# Trifluorinated Tetralins via I(I)/I(III)‐Catalysed Ring Expansion: Programming Conformation by [CH_2_CH_2_] → [CF_2_CHF] Isosterism

**DOI:** 10.1002/anie.202102222

**Published:** 2021-05-01

**Authors:** Jessica Neufeld, Timo Stünkel, Christian Mück‐Lichtenfeld, Constantin G. Daniliuc, Ryan Gilmour

**Affiliations:** ^1^ Organisch-Chemisches Institut Westfälische Wilhelms-Universität Münster Corrensstraße 36 48149 Münster Germany

**Keywords:** fluorination, iodine(III), organocatalysis, ring expansion, tetralin

## Abstract

Saturated, fluorinated carbocycles are emerging as important modules for contemporary drug discovery. To expand the current portfolio, the synthesis of novel trifluorinated tetralins has been achieved. Fluorinated methyleneindanes serve as convenient precursors and undergo efficient difluorinative ring expansion with in situ generated *p*‐TolIF_2_ (>20 examples, up to >95 %). A range of diverse substituents are tolerated under standard catalysis conditions and this is interrogated by Hammett analysis. X‐ray analysis indicates a preference for the CH−F bond to occupy a *pseudo*‐axial orientation, consistent with stabilising σ_C−H_→σ_C−F_* interactions. The replacement of the symmetric [CH_2_−CH_2_] motif by [CF_2_−CHF] removes the conformational degeneracy intrinsic to the parent tetralin scaffold leading to a predictable half‐chair. The conformational behavior of this novel structural balance has been investigated by computational analysis and is consistent with stereoelectronic theory.

## Graphical abstract

To expand the current portfolio of saturated, fluorinated carbocycles for drug discovery, a main‐group catalysis‐based synthesis of novel, trifluorinated tetralins is disclosed. Isosteric replacement of [CH_2_−CH_2_] by [CF_2_−CHF] removes the conformational degeneracy intrinsic to the parent tetralin, since the CH−F bond occupies a *pseudo*‐axial orientation (σ_C−H_→σ_C−F_*). This provides pre‐organised module for contemporary medicinal chemistry.

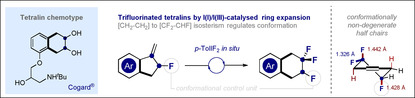

Carbocycles with tailored fluorination patterns are garnering considerable attention in the design of functional materials and therapeutics.^[1]^ Fluorination at multiple sites provides a molecular basis from which to modulate the physicochemical characteristics of the molecule without causing excessive alterations to the steric signature.^[2]^ Striking examples include the Janus ring systems developed by O′Hagan and co‐workers, which range from trifluorinated cyclopropanes (**1**)^[3]^ through to the venerable all‐*syn*‐hexafluorocyclohexane motif (**2**) (Figure 1, top):^[4]^ This latter example is noteworthy as the fluorination pattern induces the highest calculated dipole of an organic molecule to date (6.2 D). A more recent comparison of the effects of selective tetrafluorination (**3** versus **4**)^[5]^ in significantly lowering log *P* provides compelling evidence for the efficiency of this approach to drug discovery. These examples build upon the venerable history of mono‐fluorination in medicinal chemistry, as is exemplified by Fried's discovery that fluorinated cortisone exhibits enhanced efficacy relative to the parent systems,^[6]^ and the continued success of fluorinated steroid derivatives, such as dexamethasone (**5**) and diflupredante (**6**).^[7]^ Intriguingly, only three sites of fluorination are commonly explored which, in turn, are dictated by preparative considerations.^[8]^ The two most common are positions on the B‐ring of the tetralin‐derived fragment. Interestingly, the dihydroxytetralin motif constitutes the core of the β‐blocker Nadolol (Cogard^®^) (**7**), further underscoring the importance of electronegative substituents at the saturated periphery of the tetrahydronaphthalene core (Figure 1, centre). Motivated by the pallet of opportunities that multiply fluorinated carbocycles offer, the success of H/OH → F bioisosterism,[^1^, ^2^] and the prevalence of tetralin‐derivatives in nature,[^9^, ^10^] a route to generate fluorinated tetralin motifs would expand the portfolio of novel motifs for molecular design.^[11]^ Specifically, the trifluoro motif resulting from isosteric replacement of the symmetric [CH_2_−CH_2_] motif distal to the aryl ring by [CF_2_−CHF] would circumvent the conformational lability intrinsic to the parent tetralin scaffold: the introduction of hyperconjugative interactions (σ_C−H_→σ_C−F_*) would render the two half chairs non‐degenerate. To access this novel class of fluorinated heterocycles, fluorinated methyleneindanes (**8**) were selected as substrates. It was envisaged that exposure to in situ generated *p*‐TolIF_2_ ^[12]^ under the auspices of I(I)/I(III) catalysis[^13^, ^14^, ^15^] would induce a fluorinative ring expansion via an ephemeral, tricyclic phenonium ion^[16]^ to liberate the desired product (**9**) (Figure 1, bottom).

**Figure 1:**
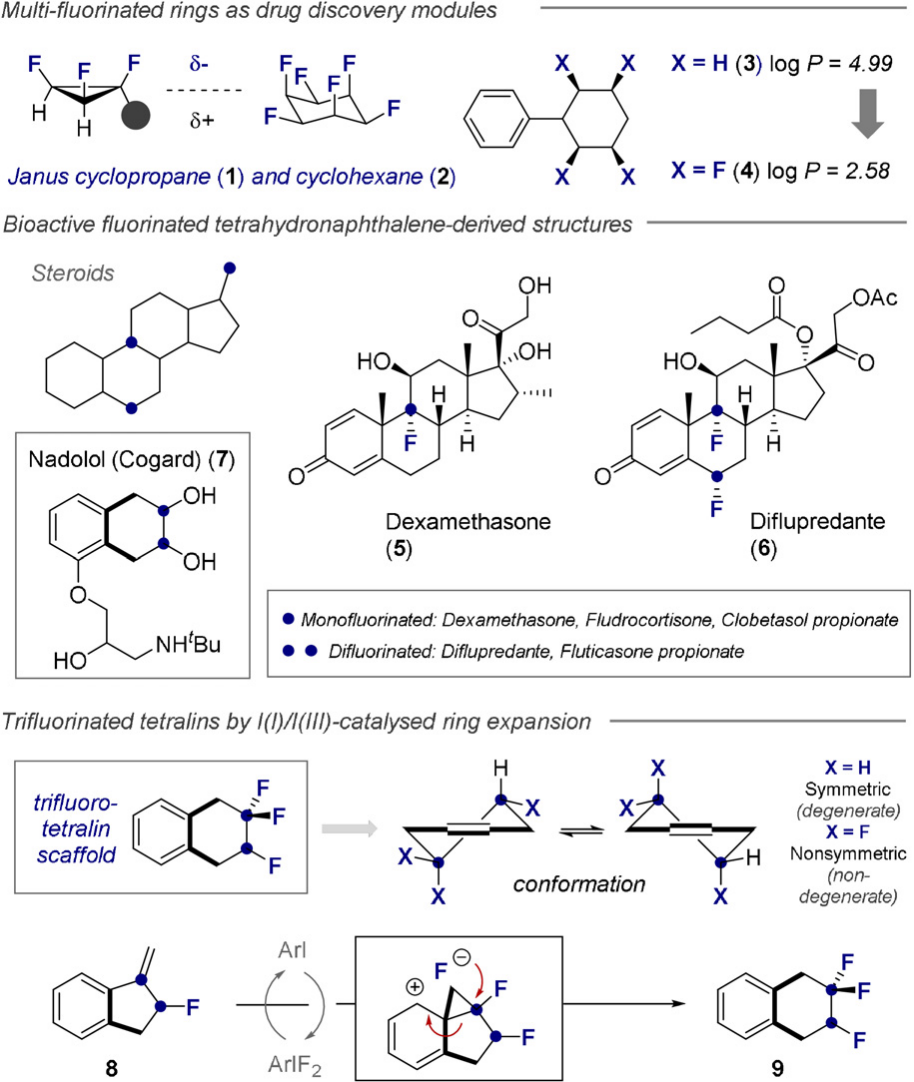
Top: Fluorinated carbocycles for contemporary drug discovery (**1**, **2** and **4**). Centre: Fluorination sites in common steroids (**5** and **6**), and the β‐blocker Nadolol (**7**). Bottom: An I(I)/I(III) paradigm to access the novel trifluorotetralin scaffold (**8 → 9**).

Confidence in the feasibility of this catalysis‐based strategy stemmed from a plenum of stoichiometric ring expansion processes. Pertinent examples include the generation of difluoro ethers from aryl‐substituted ketones using XeF_2_ by Zupan and co‐workers.^[17]^ Furthermore, the ability of hypervalent iodine reagents to induce ring expansion with Pd and Cu or BF_3_⋅OEt_2_, has been elegantly demonstrated by the groups of Szabó^[14e]^ and Murphy,[^14g^, ^14j^] respectively. To devise a catalysis‐based platform to access novel, trifluorinated tetralins, 2‐fluoro‐methyleneindane (**8**) was selected as a model substrate for reaction optimisation (Table 1). It was envisaged that this allyl fluoride would engage with *p*‐TolIF_2_, generated by in situ oxidation from inexpensive *p*‐TolI, to forge the desired carbocycle **9**, where the CHF unit would function as a conformational control unit.

**Table 1:** Optimisation of the reaction conditions. 
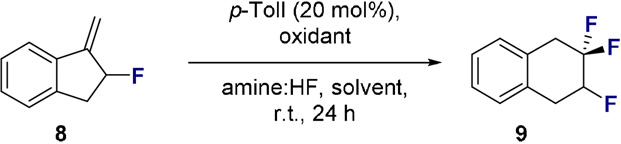

entry^[a]^	oxidant	amine:HF^[b]^	solvent	yield^[c]^
1	Selectfluor	1:3.0	CHCl_3_	7 %
2	Selectfluor	1:4.5	CHCl_3_	61 %
3	Selectfluor	1:6.0	CHCl_3_	47 %
4	Selectfluor	1:4.5	CH_2_Cl_2_	71 %
**5**	**Selectfluor**	**1:4.5**	**DCE**	**74 %**
6	Selectfluor	1:4.5	HFIP	48 %
7	Selectfluor	1:4.5	ETFA	56 %
8	Selectfluor	1:4.5	toluene	54 %
9	Selectfluor	1:4.5	CH_3_CN	28 %
10	*m‐*CPBA	1:4.5	DCE	63 %
11	Oxone	1:4.5	DCE	39 %
12^[d]^	Selectfluor	1:4.5	DCE	65 %
13^[e]^	Selectfluor	1:4.5	DCE	29 %
14^[f]^	Selectfluor	1:4.5	DCE	54 %
15^[g]^	Selectfluor	1:4.5	DCE	<5 %

[a] Standard reaction conditions: **8** (0.2 mmol), *p*‐TolI (20 mol %), oxidant (1.5 equiv.), solvent (0.5 mL), amine:HF (0.5 mL), ambient temperature, 24 h. [b] See supporting information for the exact calculation of the amine:HF mixtures. [c] Determined by ^19^F NMR analysis of the crude reaction mixture using ethyl fluoroacetate as internal standard. [d] Reaction was performed at 50 °C. [e] Reaction was performed at 0 °C. [f] Reaction was performed with 10 mol % catalyst. [g] Reaction was performed without catalyst.

Initially, the transformation was investigated using Selectfluor as the terminal oxidant in CHCl_3_ at ambient temperature using HF as a convenient fluoride source. Given the importance of Brønsted acidity in ArIX_2_ mediated processes,[^15d^, ^18^] variation of the amine:HF ratio was explored using mixtures of commercially available NEt_3_:HF 1:3 and pyr:HF 1:9.2 (Olah's reagent). Initial attempts to induce difluorinative ring expansion with an amine:HF ratio of 1:3 generated the desired *geminal* fluorinated tetralin **9** in only 7 % yield (Table 1, entry 1). However, adjusting the ratio to 1:4.5 led to a significant enhancement in efficiency (61 %, entry 2). Further increasing the amine:HF ratio to 1:6 proved to be detrimental (47 %, entry 3) and thus the remainder of the study was conducted with amine:HF 1:4.5. A solvent screen (entries 4–9) identified DCE as being the optimal reaction medium for the title transformation (74 % yield, entry 5). Fluorinated solvents such as hexafluoroisopropanol (HFIP) and ethyl trifluoroacetate (ETFA) led to moderate yields (48 % and 56 %, respectively, entries 6–7). Furthermore, non‐halogenated solvents proved to be detrimental (entries 8–9). Replacing Selectfluor with *m*‐CPBA (entry 10) or oxone (entry 11) did not lead to an improvement, nor did increasing or decreasing temperature (entries 12 and 13). Lowering the catalyst loading led to a slight decrease in yield, and the control experiment without *p*‐TolI supports the involvement of an I(I)/I(III) catalysis paradigm (54 % and <5 %, respectively, entries 14–15).

Having established suitable reaction conditions for the difluorinative ring expansion (Table 1, entry 5) the scope and the limitations of the transformation were explored (Scheme 1). Initially, the parent scaffold **9** was prepared under the standard reaction conditions, and the process could be scaled up to 1 mmol without loss of catalytic efficiency. Halogens were found to be compatible with this protocol as is exemplified by products **10**–**14** (up to >95 % yield), enabling the regioisomeric bromides **12**–**14** to be prepared as synthetically‐versatile coupling partners for downstream manipulation. Although it was possible to generate the methyl‐derivative **15**, the disparity in yield when compared with electron deficient systems prompted a more detailed Hammett analysis (vide infra). Electron‐deficient substrates proved to be highly competent precursors as exemplified by the triflate (**16**), trifluoromethyl (**17**) and cyano (**18**) species (up to 92 % yield). Phthalimide **19** was smoothly generated to provide access to masked aniline derivatives, and a substrate with a pendant α,β‐unsaturated ester (**20**) demonstrates the chemoselectivity of the transformation. The addition of substituents on the saturated ring system was tolerated (**21** and **22**), and catalysis enabled the formation of the tetrafluorinated compound **23** and mixed halogen system **24**. In all cases, reactions performed in the absence of *p*‐TolI led to <5 % yield for **9**–**24**. To correlate the electronic signature of the aryl fragment with catalysis efficiency, the Hammett values *σ_p_* and *σ_m_* of several electronically diverse compounds were plotted against the ^19^F NMR yields (Scheme 1, bottom). The plot underscores the fact that strong electron‐withdrawing groups on the aryl fragment facilitate difluorinative ring expansion.

**Scheme 1:**
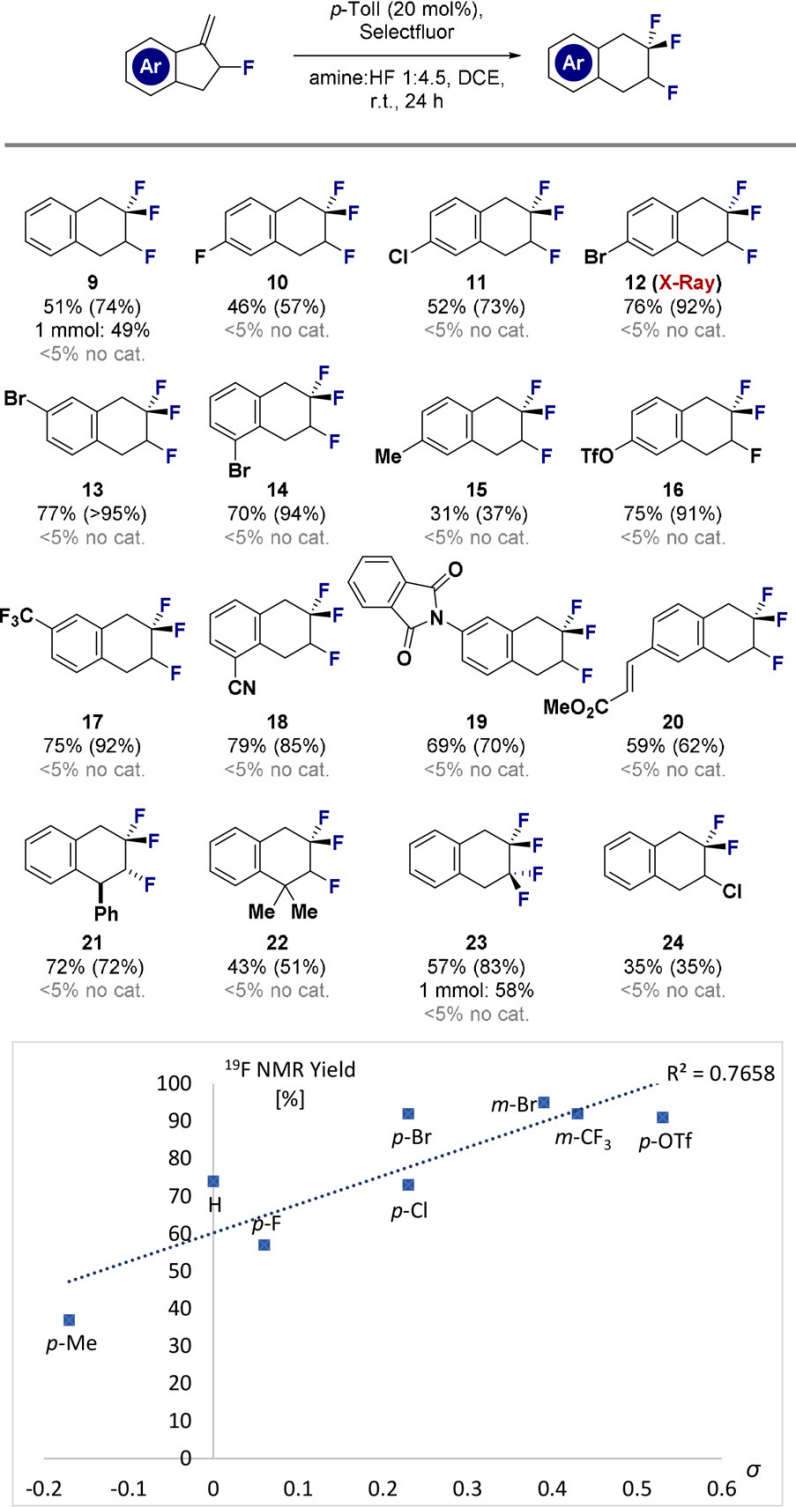
Top: Exploring the scope of the reaction. Standard reaction conditions: substrate (0.2 mmol), *p*‐TolI (20 mol %), Selectfluor (1.5 equiv.), DCE (0.5 mL), amine:HF 1:4.5 (0.5 mL), ambient temperature, 24 h. Yields refer to isolated products while ^19^F NMR yields are given in parentheses (determined by ^19^F NMR analysis of the crude reaction mixture using ethyl fluoroacetate as internal standard). Bottom: Effect of arene electron density on reaction efficiency. N.B. **Care must be taken during isolation due to the volatility of the products**.

To confirm that electron‐rich groups suppress catalysis (see Scheme 1, lower), the difluorinative ring expansion to generate **25** was attempted, but led to degradation of the starting material (Scheme 2). Furthermore, the requirement for fluorinated methyleneindanes is demonstrated through the failed attempts to generate **26** and **27**. The latter example, in which the allyl fluoride is essential, distinguishes this catalysis‐based platform from stoichiometric examples using the non‐fluorinated methyleneindane.[^14j^, ^19^]

**Scheme 2:**
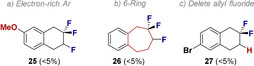
Control reactions. Standard reaction conditions: substrate (0.2 mmol), *p*‐TolI (20 mol %), Selectfluor (1.5 equiv.), DCE (0.5 mL), amine:HF 1:4.5 (0.5 mL), ambient temperature, 24 h. Yields refer to ^19^F NMR yields (determined by ^19^F NMR analysis of the crude reaction mixture using ethyl fluoroacetate as internal standard).

Given the plenum of methods to enable the enantioselective fluorination of β‐ketoesters,^[20]^ which can be processes to optically active precursors, it was envisaged that ring expansion would provide a route to access enantio‐enriched trifluorinated products (Scheme 3). Despite the addition of an additional electron‐withdrawing group, catalysis was observed under the standard conditions reported (**28**–**32**, up to 57 % yield). The ester and C(sp^2^)−Br motifs in compound **30** render it a convenient linchpin for bidirectional functionalisation. Moreover, the optical purity of the products was not compromised under the standard catalysis conditions (100 % *es*).

**Scheme 3:**
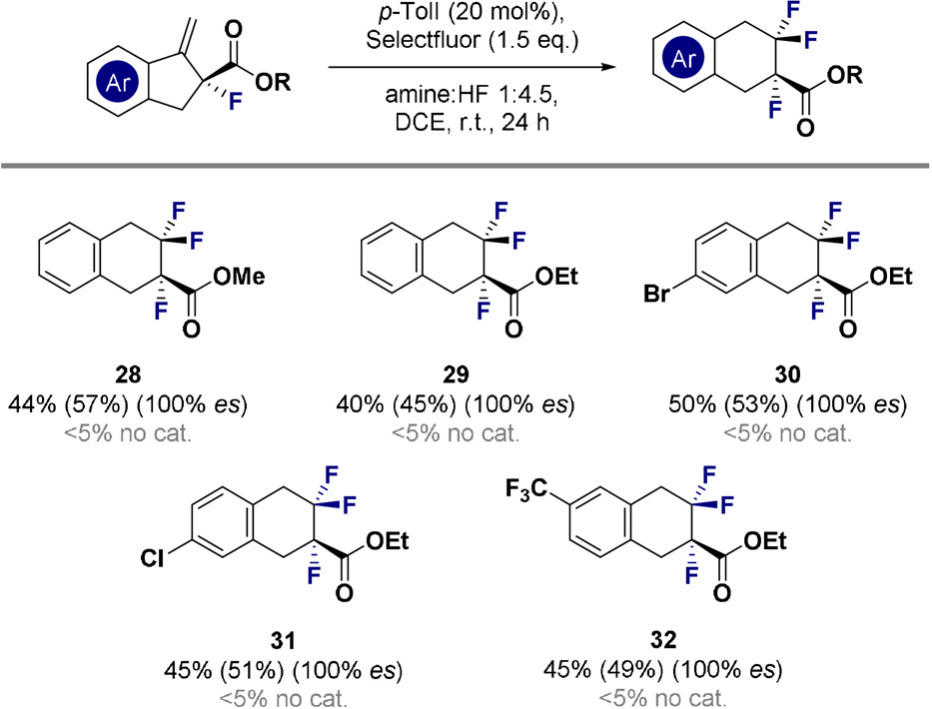
Exploring the scope of the reaction. Standard reaction conditions: substrate (0.2 mmol), *p*‐TolI (20 mol %), Selectfluor (1.5 equiv.), DCE (0.5 mL), amine:HF 1:4.5 (0.5 mL), ambient temperature, 24 h. Yields refer to isolated products while ^19^F NMR yields are given in parentheses (determined by ^19^F NMR analysis of the crude reaction mixture using ethyl fluoroacetate as internal standard). N.B. **Care must be taken during isolation due to the volatility of the products**.

To explore the conformational consequences of replacing the symmetric [CH_2_−CH_2_] motif by an isosteric non‐symmetric [CF_2_−CHF] group in tetralins [*V*_vdW_ 138 Å^3^ versus 156 Å^3^ for tetralin and **9**, respectively],^[21]^ X‐ray analysis of a representative example was conducted. It was envisaged that isosteric replacement of [CH_2_−CH_2_] by [CF_2_−CHF] would bias the conformation, thereby bypassing the degeneracy of the two half chairs in the parent system (Figure 2). In the case of compound **12**, the conformer in which the C(sp^3^)−F of the CHF unit adopts a *quasi*‐axial orientation is observed.^[22]^ This allows for stabilising hyperconjugative [σ_C−H_→σ_C−F_*] interactions, as is evident from the difference in C−F_ax._ and C−F_eq._ bond lengths (1.442 and 1.428 versus 1.326 Å, respectively, Δ*d*_C−F(ax.‐eq.)_ 0.1 Å).

**Figure 2:**
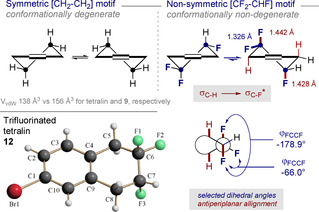
X‐ray crystal structure of tetralin **12**.^[22]^

To quantify this interaction, a conformational analysis of compound **12** was conducted at the DFT level of theory (Please see the Supporting Information for full details). The optimised molecular structures (TPSS‐D3/def2‐TZVP) of the two half chair conformers **12‐a** and **12‐b** (Figure 3), confirm a preference for the pseudo‐*axial* species (ΔΔ*G*_298_ +1.0 kcal mol^−1^), and the calculated bond lengths are in good agreement with the experimental values. A NBO second order perturbation analysis reveals that the largest contribution arises from *vicinal* σ_C−H_→σ_C−F_* interactions, which is fully in line with the working hypothesis.

**Figure 3:**
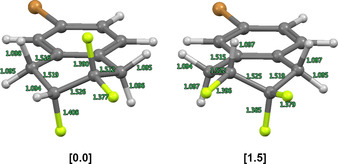
Optimised molecular structure (TPSS‐D3/def2‐TZVP) of **12‐a** (left) and alternative conformer **12‐b** (right). Internuclear distances are given in Å. In square brackets: relative free energies Δ*G*_298_ (PW6B95‐D3//TPSS‐D3/def2‐TZVP) in kcal mol^−1^.

In conclusion, a main group catalysis‐based strategy has been leveraged to access novel trifluorinated tetralins by difluorinative ring expansion of fluorinated methyleneindanes. Isosteric replacement of the symmetric [CH_2_−CH_2_] motif by a non‐symmetric [CF_2_−CHF] group [Δ*V*_vdW_=ca. 13 %] at the distal edge of the saturated carbocycle removes the conformational degeneracy inherent to the parent system. The stabilising hyperconjugative interactions [σ_C−H_→σ_C−F_*] that underpin this effect manifest themselves in the X‐ray crystal structure analysis (Δ*d*_C−F(ax.‐eq.)_ 0.1 Å) and have been interrogated by DFT analysis. It is envisaged that these novel tetralins, and the stereoelectronic bias that governs pre‐organisation, will find application in focused medicinal chemistry and molecular design in a broader sense.

## Conflict of interest

The authors declare no conflict of interest.

## Supporting information

As a service to our authors and readers, this journal provides supporting information supplied by the authors. Such materials are peer reviewed and may be re‐organized for online delivery, but are not copy‐edited or typeset. Technical support issues arising from supporting information (other than missing files) should be addressed to the authors.

Supplementary
