# Supplementary material for: Trifluorinated Tetralins via I(I)/I(III)‐Catalysed Ring Expansion: Programming Conformation by [CH2CH2] → [CF2CHF] Isosterism
Source: Angew Chem Int Ed Engl. 2021 May 1;60(24):13647–51. doi: 10.1002/anie.202102222 (PMC8251640; doi:10.1002/anie.202102222)
Supplement: Supplementary file 1 — Supplementary [file ANIE-60-13647-s001.pdf]

Angewandte Chemie
Eine Zeitschrift der Gesellschaft Deutscher Chemiker

Supporting Information

Trifluorinated Tetralins via I(I)/I(III)-Catalysed Ring Expansion: Programming Conformation by [CH_2CH_2] → [CF_2CHF] Isosterism

*Jessica Neufeld, Timo Stünkel, Christian Mück-Lichtenfeld, Constantin G. Daniliuc, and Ryan Gilmour**

anie_202102222_sm_miscellaneous_information.pdf

Table of Contents

I.	General Information	3
II.	Optimisation	5
III.	Synthesis of Starting Materials	7
IV.	Synthesis of Trifluorinated Tetralins	48
V.	HPLC Traces	65
VI.	X-Ray Crystallographic Data	80
VII.	DFT Calculations	82
VIII.	NMR Spectra of Unreported Starting Materials	90
IX.	NMR Spectra of Trifluorinated Tetralins	152
X.	References	195

I. General Information

All chemicals were purchased as reagent grade and used as received unless otherwise stated. Solvents for extraction or purification were purchased as technical grade and distilled on the rotary evaporator prior to use. Dry solvents were dried by a Grubbs-type purification system including columns packed with molecular sieves and aluminium oxide. Column chromatography was performed using silica gel (40-63 *µ*m; *VWR Chemicals*) as stationary phase. Reaction monitoring was achieved by analytical thin layer chromatography (TLC) on aluminum foil pre-coated with silica gel 60 F_{254} (*Merck*). Compounds were visualised with UV light (254 nm) or by chemical staining using a solution of $KMnO_4$ ($KMnO_4$ (10 g), K_2CO_3 (65 g), NaOH (1 N, 15 mL) in water (1 L)) followed by heating. Concentration *in vacuo* was performed at ~10 mbar at 40 °C unless otherwise stated. NMR spectra were measured by the NMR service in the Organisch-Chemisches Institut, Westfälische Wilhelms-Universität Münster on a *Bruker Avance II 300*, a *Bruker Avance II 400,* an *Agilent DD2 500* or an *Agilent DD2 600* spectrometer. Chemical shifts are given relative to TMS and are referenced to the residual solvent peak as internal standard. ^{1}H NMR spectra are reported as follows: chemical shift *δ* in ppm (multiplicity, coupling constant J_{FH} and J_{HH} in Hz, number of protons, assignment of proton). ^{13}C NMR spectra are reported as follows: chemical shift *δ* in ppm (multiplicity, coupling constant J_{FC} in Hz, number of carbons, assignment of carbon). ^{19}F NMR spectra are reported as follows: chemical shift *δ* in ppm (multiplicity, coupling constant J_{FH} in Hz, number of fluorines, assignment of fluorine). The resonance multiplicity is abbreviated as s (singlet), d (doublet), t (triplet), q (quartet) or m (multiplet). Assignments of unknown compounds are based on COSY, HSQC and HMBC spectra. Melting points were measured on a *Büchi B-545* melting point apparatus in open capillaries and are uncorrected. IR spectra were recorded on a *Perkin-Elmer 100 FT-IR* spectrometer. Absorption bands are reported in wave numbers $\tilde{\upsilon}$ (cm^{-1}) and the intensities are reported as w (weak), m (medium), s (strong). Mass spectra were measured by the MS service of the Organisch-Chemisches Institut, Westfälische Wilhelms-Universität Münster on a *Bruker Daltonics MicroTof* (HRMS-ESI), a *Triplequad TSQ 7000* (MS-EI), *Triplequad Quattro Micro GC* (GC-EI-MS), a *Qp5050 Single Quad* (GC-EI-MS) or a *LTQ Orbitap LTQ XL* (HRMS-APCI).

Amine:HF mixtures

Amine:HF mixtures were premixed in stock solutions (stored under -20 °C) from commercially available NEt_3:HF 1:3 and pyr:HF 1:9.23 (Olah's reagent). Different stock solutions were prepared as follows:

A mixture of amine:HF 1:3 was obtained by using 0.50 mL of NEt_3:HF 1:3.

A mixture of amine:HF 1:4.5 was obtained by mixing 0.34 mL of NEt_3:HF 1:3 and 0.16 mL of pyr:HF 1:9.2.

A mixture of amine:HF 1:6 was obtained by mixing 0.21 mL of NEt_3:HF 1:3 and 0.29 mL of pyr:HF 1:9.23.

II. Optimisation

General procedure A for the optimisation

2-Fluoro-1-methylene-2,3-dihydro-1*H*-indene (**8**) (30 mg, 0.2 mmol, 1.0 eq.) and *p*-TolI (8.7 mg, 40 μmol, 20 mol%) were dissolved in the stated solvent (0.5 mL) in a Teflon® screw cap vial. The stated amine:HF mixture (0.5 mL) and oxidant (0.3 mmol, 1.5 eq.) were added and the reaction mixture was stirred for 24 h at ambient temperature. The reaction mixture was poured into a saturated solution of $NaHCO_3$ and the aqueous layer was extracted with CH_2Cl_2 (3x). The combined organic layers were dried over $MgSO_4$, filtered and the solvent was removed at max. 300 mbar. The yield was determined by ^{19}F NMR analysis of the crude reaction mixture using ethyl fluoroacetate as internal standard (optimised ^{19}F delay time for quantitative analysis).

Caution: Fluorinated product is volatile and starts to sublimate under high vacuum.

Table S1: Reaction optimisation for the synthesis of trifluorinated tetralin **9**.

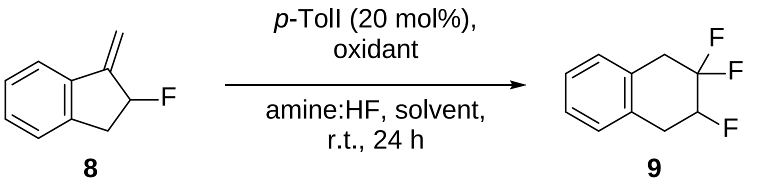

entry	oxidant	amine:HF	solvent	yield
1	Selectfluor	1:3	$CHCl_3$	7%
2	Selectfluor	1:4.5	$CHCl_3$	61%
3	Selectfluor	1:6	$CHCl_3$	47%
4	Selectfluor	1:4.5	CH_2Cl_2	71%
**5**	**Selectfluor**	**1:4.5**	**DCE**	**74%**
6	Selectfluor	1:4.5	HFIP	48%
7	Selectfluor	1:4.5	ETFA	56%
8	Selectfluor	1:4.5	toluene	54%
9	Selectfluor	1:4.5	CH_3CN	28%
10	*m*-CPBA	1:4.5	DCE	63%
11	Oxone	1:4.5	DCE	39%
12[a]	Selectfluor	1:4.5	DCE	65%
13[b]	Selectfluor	1:4.5	DCE	29%
14[c]	Selectfluor	1:4.5	DCE	54%
15[d]	Selectfluor	1:4.5	DCE	<5%

[a]Reaction was performed at 50 °C. [b]Reaction was performed at 0 °C. [c]Reaction was performed with 10 mol% catalyst. [d]Control reaction without catalyst.

III. Synthesis of Starting Materials

General procedure B for the synthesis of *α*-fluoro indanones

According to a modified procedure[1] the corresponding indanone (1.0 eq.) and Selectfluor (1.2 eq.) were refluxed in MeOH for the indicated time. The white suspension was filtered, the solvent was removed *in vacuo* and the crude mixture was purified by column chromatography.

General procedure C for the synthesis of 2-fluoro-1-methylene-2,3-dihydro-1*H*-indenes

According to a modified procedure[2] a Schlenk tube was charged with Ph_3PMeBr (1.5 eq.) and dry THF. KO^{t}Bu (1.5 eq.) was added and the reaction mixture was stirred for 30 min before it was cooled to 0 °C. The corresponding *α*-fluoro indanone (1.0 eq.) was added and the mixture was stirred for the indicated time at room temperature. The reaction mixture was diluted with H_2O and EtOAc, and the aqueous layer was extracted with EtOAc (3x). The combined organic layers were washed with brine, dried over $MgSO_4$ and filtered. The solvent was removed *in vacuo* and the crude mixture was purified by column chromatography.
Caution: Products are heat and light sensitive after column chromatography. The solvent was removed at room temperature after column chromatography and the products were stored in the dark.

2-Fluoro-2,3-dihydro-1*H*-inden-1-one (S1)

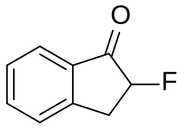

According to **general procedure B**, indanone (1.6 g, 12 mmol, 1.0 eq.) and Selectfluor (5.2 g, 15 mmol, 1.2 eq.) were refluxed in MeOH (90 mL) for 24 h. Column chromatography purification (*n*-pentane:EtOAc 20:1) afforded product **S1** as white solid (1.5 g, 9.9 mmol, 83%).

R$_f$ (*n*-pentane:EtOAc 10:1) = 0.31. **M.p.** 58 – 59 °C. **^{1}H NMR** (300 MHz, $CDCl_3$, 299 K) *δ* [ppm] = 7.81 (m, 1H), 7.67 (m, 1H), 7.51 – 7.38 (m, 2H), 5.28 (ddd, *J* = 51.0, 7.7, 4.3 Hz, 1H), 3.63 (ddd, *J* = 17.1, 7.5, 7.3 Hz, 1H), 3.24 (ddd, *J* = 22.2, 17.1, 4.4 Hz, 1H). **^{19}F NMR** (282 MHz, $CDCl_3$, 299 K) *δ* [ppm] = -194.1 (ddd, *J* = 50.9, 23.2, 7.1 Hz, 1F). **^{19}F{^{1}H} NMR** (282 MHz, $CDCl_3$, 299 K) *δ* [ppm] = -194.1 (s, 1F). **GC-**

MS (EI) *m/z* $[M]^+$, calcd. for $C_9H_7FO^+$: 150.05, found: 150.10. Analytic data in agreement with the literature.[3]

2-Fluoro-1-methylene-2,3-dihydro-1*H*-indene (8)

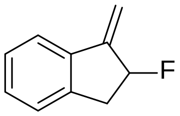

According to **general procedure C**, Ph_3PMeBr (4.1 g, 12 mmol, 1.5 eq.) and KO^tBu (1.3 g, 12 mmol, 1.5 eq.) were dissolved in THF (30 mL). 2-Fluoro-2,3-dihydro-1*H*-inden-1-one (**S1**) (1.1 g, 7.5 mmol, 1.0 eq.) was added and the reaction mixture was stirred for 16 h. Column chromatography purification (*n*-pentane) afforded product **8** as colourless oil (0.94 g, 6.4 mmol, 85%).

R_f (*n*-pentane) = 0.33. **^{1}H NMR** (400 MHz, $CDCl_3$, 299 K) δ [ppm] = 7.52 (m, 1H), 7.26 (m, 3H), 5.77 (dd, *J* = 5.4, 1.6 Hz, 1H), 5.65 (dddt, *J* = 55.2, 6.7, 2.8, 1.5 Hz, 1H), 5.50 (dd, *J* = 5.4, 1.4 Hz, 1H), 3.33 (ddd, *J* = 23.7, 17.5, 6.7 Hz, 1H), 3.16 (ddd, *J* = 28.8, 17.5, 2.5 Hz, 1H). **^{19}F NMR** (376 MHz, $CDCl_3$, 299 K) δ [ppm] = -167.2 (ddddd, *J* = 54.2, 28.8, 23.3, 5.5, 5.4 Hz, 1F). **^{19}F{^{1}H} NMR** (376 MHz, $CDCl_3$, 299 K) δ [ppm] = -167.2 (s, 1F). **GC-MS** (EI) *m/z* $[M]^+$, calcd. for $C_{10}H_9F^+$: 148.07, found: 148.11. Analytic data in agreement with the literature.[4]

2,5-Difluoro-2,3-dihydro-1*H*-inden-1-one (S2)

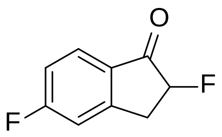

According to **general procedure B**, 5-fluoro-1-indanone (1.5 g, 10 mmol, 1.0 eq.) and Selectfluor (4.2 g, 12 mmol, 1.2 eq.) were refluxed in MeOH (70 mL) for 16 h. Column chromatography purification (*n*-pentane:EtOAc 20:1) afforded product **S2** as white solid (0.83 g, 4.9 mmol, 49%).

R_f (*n*-pentane:EtOAc 10:1) = 0.31. **M.p.** 57 – 58 °C. **^{1}H NMR** (400 MHz, $CDCl_3$, 299 K) δ [ppm] = 7.83 (m, 1H), 7.18 – 7.10 (m, 2H), 5.27 (ddd, *J* = 50.9, 7.7, 4.2 Hz, 1H), 3.62 (ddd, *J* = 17.2, 7.7, 7.5 Hz, 1H), 3.23 (ddd, *J* = 22.3, 17.6, 4.2 Hz, 1H). **^{19}F NMR** (376 MHz, $CDCl_3$, 299 K) δ [ppm] = -99.1 (tdd, *J* = 8.4, 5.1, 2.3 Hz, 1F), -192.7 (dddd, *J* = 50.9, 23.3, 7.6, 2.4 Hz, 1F). **^{19}F{^{1}H} NMR** (376 MHz, $CDCl_3$, 299 K) δ [ppm] = -99.1 (s, 1F), -192.7 (s, 1F). **GC-MS** (EI) *m/z* $[M]^+$, calcd. for $C_9H_6F_2O^+$: 168.04, found: 168.09. Analytic data in agreement with the literature.[5]

2,5-Difluoro-1-methylene-2,3-dihydro-1*H*-indene (S3)

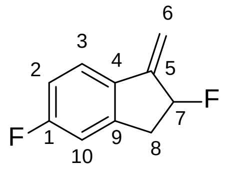

According to **general procedure C**, Ph_3PMeBr (2.2 g, 6.0 mmol, 1.5 eq.) and KO^{t}Bu (0.67 g, 6.0 mmol, 1.5 eq.) were dissolved in THF (15 mL). 2,5-Difluoro-2,3-dihydro-1*H*-inden-1-one (**S2**) (0.67 g, 4.0 mmol, 1.0 eq.) was added and the reaction mixture was stirred for 16 h. Column chromatography purification (*n*-pentane) afforded product **S3** as colourless oil (0.45 g, 2.7 mmol, 68%).

R$_f$ (*n*-pentane) = 0.34. **^{1}H NMR** (500 MHz, $CDCl_3$, 299 K) δ [ppm] = 7.47 (m, 1H, H3), 6.95 (m, 2H, H2, H10), 5.72 – 5.55 (m, 2H, H6^a, H7), 5.46 (dt, $^4J_{FH}$ = 5.5 Hz, J = 1.1 Hz, 1H, H6^b), 3.31 (ddd, $^3J_{FH}$ = 23.8 Hz, $^2J_{HH}$ = 17.8 Hz, J = 6.6 Hz, 1H, H8^a), 3.14 (dddd, $^3J_{FH}$ = 28.5 Hz, $^2J_{HH}$ = 17.7 Hz, J = 2.4, 1.1 Hz, 1H, H8^b). **^{13}C NMR** (126 MHz, $CDCl_3$, 299 K) δ [ppm] = 163.8 (d, $^1J_{FC}$ = 248.0 Hz, 1C, C1), 147.3 (d, $^2J_{FC}$ = 14.0 Hz, 1C, C5), 144.3 (d, $^3J_{FC}$ = 8.7 Hz, 1C, C9), 134.3 (dd, $^3J_{FC}$ = 2.5 Hz, $^4J_{FC}$ = 2.3 Hz, 1C, C4), 122.7 (d, $^3J_{FC}$ = 9.1 Hz, 1C, C3), 114.9 (d, $^2J_{FC}$ = 23.3 Hz, 1C, C2), 112.5 (dd, $^2J_{FC}$ = 22.6 Hz, $^4J_{FC}$ = 1.6 Hz, 1C, C10), 109.2 (dd, $^3J_{FC}$ = 7.9 Hz, $^6J_{FC}$ = 2.6 Hz, 1C, C6), 94.2 (d, $^1J_{FC}$ = 178.2 Hz, 1C, C7), 38.2 (dd, $^2J_{FC}$ = 23.4 Hz, $^4J_{FC}$ = 2.0 Hz, 1C, C8). **^{19}F NMR** (470 MHz, $CDCl_3$, 299 K) δ [ppm] = -112.0 (ddd, J = 8.9, 8.7, 5.2 Hz, 1F, F1), -166.4 (dddd, $^2J_{FH}$ = 56.9 Hz, $^3J_{FH}$ = 28.7, 23.4 Hz, $^4J_{FH}$ = 5.4 Hz, 1F, F7). **^{19}F{^{1}H} NMR** (470 MHz, $CDCl_3$, 299 K) δ [ppm] = -112.0 (s, 1F, F1), -166.4 (s, 1F, F7). **IR** (ATR) $\tilde{\upsilon}$ [cm^{-1}] = 2958 (w), 1648 (w), 1612 (m), 1593 (m), 1481 (s), 1422 (w), 1322 (m), 1244 (s), 1167 (w), 1132 (m), 1081 (w), 1016 (m), 1000 (m), 934 (m), 897 (m), 835 (s), 820 (s), 760 (w), 663 (w). **GC-MS** (EI) *m/z* [M]$^+$, calcd. for $C_{10}H_8F_2$: 166.0589, found: 166.0586.

5-Chloro-2-fluoro-2,3-dihydro-1*H*-inden-1-one (S4)

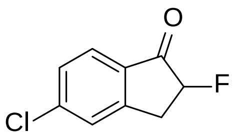

According to **general procedure B**, 5-chloro-1-indanone (1.7 g, 10 mmol, 1.0 eq.) and Selectfluor (4.2 g, 12 mmol, 1.2 eq.) were refluxed in MeOH (70 mL) for 16 h. Column chromatography purification (*n*-pentane:EtOAc 20:1) afforded product **S4** as pale yellow solid (0.90 g, 4.9 mmol, 49%).

R$_f$ (*n*-pentane:EtOAc 10:1) = 0.40. **M.p.** 119 – 120 °C. **^{1}H NMR** (400 MHz, $CDCl_3$, 299 K) δ [ppm] = 7.74 (m, 1H), 7.47 (m, 1H), 7.42 (m, 1H), 5.27 (ddd, J = 50.9, 7.7, 4.3 Hz, 1H), 3.61 (ddd, J = 15.9, 7.6, 7.5 Hz,

1H), 3.22 (ddd, J = 23.2, 17.3, 4.3 Hz, 1H). **^{19}F NMR** (377 MHz, $CDCl_3$, 299 K) δ [ppm] = -193.1 (ddd, J = 50.9, 23.2, 7.7 Hz, 1F). **^{19}F{^{1}H} NMR** (377 MHz, $CDCl_3$, 299 K) δ [ppm] = -193.1 (s, 1F). **GC-MS** (EI) *m/z* $[M]^+$, calcd. for $C_9H_6ClFO^+$: 184.01, found: 184.08. Analytic data in agreement with the literature.[5]

5-Chloro-2-fluoro-1-methylene-2,3-dihydro-1*H*-indene (S5)

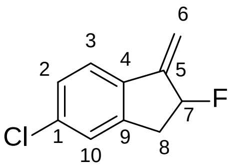

According to **general procedure C**, Ph_3PMeBr (2.2 g, 6.0 mmol, 1.5 eq.) and KO^tBu (0.67 g, 6.0 mmol, 1.5 eq.) were dissolved in THF (12 mL). 5-Chloro-2-fluoro-2,3-dihydro-1*H*-inden-1-one (**S4**) (0.74 g, 4.0 mmol, 1.0 eq.) was added and the reaction mixture was stirred for 16 h. Column chromatography purification (*n*-pentane) afforded product **S5** as colourless oil (0.43 g, 2.4 mmol, 59%).

R_f (*n*-pentane) = 0.34. **^{1}H NMR** (500 MHz, $CDCl_3$, 299 K) δ [ppm] = 7.43 (m, 1H, H3), 7.26 (m, 1H, H10), 7.23 (m, 1H, H2), 5.76 (dd, $^4J_{FH}$ = 5.5 Hz, $^4J_{HH}$ = 1.6 Hz, 1H, H6^a), 5.64 (dddt, $^2J_{FH}$ = 55.0 Hz, $^3J_{HH}$ =6.6, 2.6 Hz, $^4J_{HH}$ = 1.5 Hz, 1H, H7), 5.52 (dd, $^4J_{FH}$ = 5.4 Hz, $^4J_{HH}$ = 1.3 Hz, 1H, H6^b), 3.30 (ddd, $^3J_{FH}$ = 24.1 Hz, $^2J_{HH}$ = 17.8 Hz, $^3J_{HH}$ = 6.9 Hz, 1H, H8^a), 3.14 (ddt, $^3J_{FH}$ = 28.5 Hz, $^2J_{HH}$ =17.7 Hz, $^3J_{HH}$ =2.6 Hz, J = 0.9 Hz, 1H, H8^b). **^{13}C NMR** (126 MHz, $CDCl_3$, 299 K) δ [ppm] = 147.4 (d, $^2J_{FC}$ = 13.9 Hz, 1C, C5), 143.8 (1C, C9), 136.8 (d, $^3J_{FC}$ = 2.3 Hz, 1C, C4), 135.0 (1C, C1), 127.8 (1C, C2), 125.8 (d, $^4J_{FC}$ = 1.8 Hz, 1C, C10), 122.3 (1C, C3), 110.4 (d, $^3J_{FC}$ = 8.0 Hz, 1C, C6), 93.9 (d, $^1J_{FC}$ = 178.3 Hz, 1C, C7), 38.1 (d, $^2J_{FC}$ = 23.4 Hz, 1C, C8). **^{19}F NMR** (470 MHz, $CDCl_3$, 299 K) δ [ppm] = -166.5 (dddt, $^2J_{FH}$ = 55.1 Hz, $^3J_{FH}$ = 28.8, 23.5 Hz, $^4J_{FH}$ = 5.4 Hz, 1F, F7). **^{19}F{^{1}H} NMR** (470 MHz, $CDCl_3$, 299 K) δ [ppm] = -166.5 (s, 1F, F7). **IR** (ATR) $\tilde{\nu}$ [cm^{-1}] = 2980 (w), 1647 (w), 1599 (m), 1572 (w), 1470 (m), 1420 (w), 1317 (m), 1213 (w), 1100 (w), 1068 (m), 1016 (s), 999 (s), 899 (m), 876 (s), 832 (s), 822 (s), 758 (w), 745 (w). **GC-MS** (EI) *m/z* $[M]^+$, calcd. for $C_{10}H_8ClF^+$: 182.0293, found: 182.0292.

5-Bromo-2-fluoro-2,3-dihydro-1*H*-inden-1-one (S6)

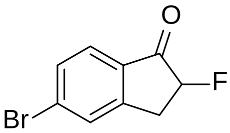

According to **general procedure B**, 5-bromo-1-indanone (1.0 g, 5.0 mmol, 1.0 eq.) and Selectfluor (2.1 g, 6.0 mmol, 1.2 eq.) were refluxed in MeOH (35 mL) for 18 h.

Column chromatography purification (*n*-pentane:EtOAc 20:1) afforded product **S6** as pale yellow solid (0.87 g, 3.8 mmol, 76%).

R$_f$ (*n*-pentane:EtOAc 10:1) = 0.43. **M.p.** 132 – 133 °C. **^{1}H NMR** (400 MHz, $CDCl_3$, 299 K) δ [ppm] = 7.66 (m, 2H), 7.58 (m, 1H), 5.26 (ddd, J = 50.9, 7.8, 4.3 Hz, 1H), 3.61 (ddd, J = 16.3, 7.7, 7.5 Hz, 1H), 3.23 (ddd, J = 23.2, 17.3, 4.2 Hz, 1H). **^{19}F NMR** (376 MHz, $CDCl_3$, 299 K) δ [ppm] = -193.3 (ddd, J = 50.9, 23.2, 7.7 Hz, 1F). **^{19}F{^{1}H} NMR** (376 MHz, $CDCl_3$, 299 K) δ [ppm] = -193.3 (s, 1F). **GC-MS** (EI) *m/z* $[M]^+$, calcd. for $C_9H_6BrFO^+$: 228.00, found: 228.00. Analytic data in agreement with the literature.[6]

5-Bromo-2-fluoro-1-methylene-2,3-dihydro-1*H*-indene (S7)

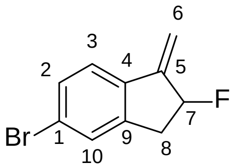

According to **general procedure C**, Ph_3PMeBr (1.4 g, 3.8 mmol, 1.5 eq.) and KO^tBu (0.46 g, 3.8 mmol, 1.5 eq.) were dissolved in THF (15 mL). 5-Bromo-2-fluoro-2,3-dihydro-1*H*-inden-1-one (**S6**) (0.57 g, 2.5 mmol, 1.0 eq.) was added and the reaction mixture was stirred for 16 h. Column chromatography purification (*n*-pentane) afforded product **S7** as colourless oil (0.27 g, 1.2 mmol, 48%).

R$_f$ (*n*-pentane) = 0.31. **^{1}H NMR** (500 MHz, $CDCl_3$, 299 K) δ [ppm] = 7.42 (m, 1H, H10), 7.37 (m, 2H, H2, H3), 5.77 (dd, $^4J_{FH}$ = 5.4 Hz, $^4J_{HH}$ = 1.6 Hz, 1H, H6^a), 5.62 (dddt, $^2J_{FH}$ = 55.0 Hz, $^3J_{HH}$ = 6.7, 2.7 Hz, $^4J_{HH}$ = 1.5 Hz, 1H, H7), 5.53 (dd, $^4J_{FH}$ = 5.4 Hz, $^4J_{HH}$ =1.4 Hz, 1H, H6^b), 3.30 (dddd, $^3J_{FH}$ = 23.6 Hz, $^2J_{HH}$ = 17.7 Hz, $^3J_{HH}$ = 6.6 Hz, J = 1.1 Hz, 1H, H8^a), 3.13 (dddd, $^3J_{FH}$ = 28.5 Hz, $^2J_{HH}$ = 17.7 Hz, $^3J_{HH}$ = 2.5 Hz, J = 1.0 Hz, 1H, H8^b). **^{13}C NMR** (126 MHz, $CDCl_3$, 299 K) δ [ppm] = 147.5 (d, $^2J_{FC}$ = 13.8 Hz, 1C, C5), 144.1 (1C, C9), 137.2 (d, $^3J_{FC}$ = 2.4 Hz, 1C, C4), 130.7 (1C, C2), 128.7 (d, $^4J_{FC}$ = 1.6 Hz, 1C, C10), 123.2 (1C, C3), 122.6 (1C, C1), 110.6 (d, $^3J_{FC}$ = 7.7 Hz, 1C, C6), 93.8 (d, $^1J_{FC}$ = 178.4 Hz, 1C, C7), 38.0 (d, $^2J_{FC}$ = 23.4 Hz, 1C, C8). **^{19}F NMR** (470 MHz, $CDCl_3$, 299 K) δ [ppm] = -166.5 (dddt, $^2J_{FH}$ = 55.0 Hz, $^3J_{FH}$ = 28.8, 23.6 Hz, $^4J_{FH}$ = 5.4 Hz, 1F, F7). **^{19}F{^{1}H} NMR** (470 MHz, $CDCl_3$, 299 K) δ [ppm] = -166.5 (s, 1F, F7). **IR** (ATR) $\tilde{\upsilon}$ [cm^{-1}] = 2981 (m), 1649 (w), 1586 (m), 1469 (m),1412 (w), 1317 (w), 1129 (w), 1095 (m), 1059 (m), 1015 (s), 998 (s), 899 (m), 862 (s), 830 (s), 821 (s), 756 (m), 744 (w). **GC-MS** (EI) *m/z* $[M]^+$, calcd. for $C_{10}H_8BrF^+$: 225.9788, found: 225.9788.

6-Bromo-2-fluoro-2,3-dihydro-1*H*-inden-1-one (S8)

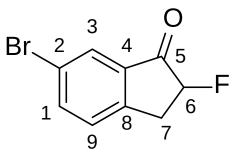

According to **general procedure B**, 6-bromo-1-indanone (2.1 g, 10 mmol, 1.0 eq.) and Selectfluor (4.2 g, 12 mmol, 1.2 eq.) were refluxed in MeOH (70 mL) for 16 h. Column chromatography purification (*n*-pentane:EtOAc 20:1) afforded product **S8** as pale yellow solid (0.52 g, 2.5 mmol, 25%).

R_f (*n*-pentane:EtOAc 10:1) = 0.31. **M.p.** 120 – 122 °C. **^{1}H NMR** (500 MHz, $CDCl_3$, 299 K) δ [ppm] = 7.93 (d, $^4J_{HH}$ = 1.9 Hz, 1H, H3), 7.77 (dd, $^3J_{HH}$ = 8.2 Hz, $^4J_{HH}$ = 2.0 Hz, 1H, H1), 7.36 (dd, $^3J_{HH}$ = 8.2 Hz, $^4J_{HH}$ = 1.2 Hz, 1H, H9), 5.27 (ddd, $^2J_{FH}$ = 50.8 Hz, $^3J_{HH}$ = 7.7, 4.3 Hz, 1H, H6), 3.58 (ddd, $^2J_{HH}$ = 17.0 Hz, $^3J_{HH}$ = 7.7 Hz, $^3J_{FH}$ = 7.5 Hz, 1H, H7^a), 3.17 (dddd, $^3J_{FH}$ = 22.8 Hz, $^2J_{HH}$ = 17.2 Hz, $^3J_{HH}$ = 4.4 Hz, $^4J_{HH}$ = 1.2 Hz, 1H, H7^b). **^{13}C NMR** (126 MHz, $CDCl_3$, 299 K) δ [ppm] = 198.6 (d, $^2J_{FC}$ = 15.0 Hz, 1C, C5), 148.2 (d, $^3J_{FC}$ = 5.7 Hz, 1C, C4), 139.3 (1C, C1), 135.8 (1C, C8), 128.5 (d, $^4J_{FC}$ = 1.8 Hz, 1C, C9), 127.8 (d, $^4J_{FC}$ = 1.4 Hz, 1C, C3), 122.8 (1C, C2), 90.5 (d, $^1J_{FC}$ = 191.6 Hz, 1C, C6), 33.3 (d, $^2J_{FC}$ = 21.8 Hz, 1C, C7). **^{19}F NMR** (470 MHz, $CDCl_3$, 299 K) δ [ppm] = -193.4 (ddd, $^2J_{FH}$ = 50.9 Hz, $^3J_{FH}$ = 23.2, 7.6 Hz, 1F, F6). **^{19}F{^{1}H} NMR** (470 MHz, $CDCl_3$, 299 K) δ [ppm] = -193.4 (s, 1F, F6). **IR** (ATR) $\tilde{\nu}$ [cm^{-1}] = 3085 (w), 3057 (w), 1717 (s), 1599 (m), 1470 (m), 1429 (m), 1417 (m), 1263 (m), 1240 (m), 1196 (s), 1186 (s), 1121 (w), 1077 (s), 1006 (s), 900 (m), 844 (m), 826 (s), 796 (s), 735 (s), 684 (w). **GC-MS** (EI) *m/z* [M]$^+$, calcd. for C_9H_6OBrF: 227.9581, found: 227.9580.

6-Bromo-2-fluoro-1-methylene-2,3-dihydro-1*H*-indene (S9)

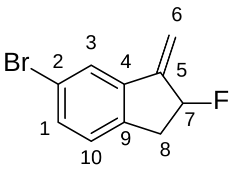

According to **general procedure C**, Ph_3PMeBr (1.1 g, 3.0 mmol, 1.5 eq.) and KO^{t}Bu (0.34 g, 3.0 mmol, 1.5 eq.) were dissolved in THF (7 mL). 6-Bromo-2-fluoro-2,3-dihydro-1*H*-inden-1-one (**S8**) (0.46 g, 2.0 mmol, 1.0 eq.) was added and the reaction mixture was stirred for 16 h. Column chromatography purification (*n*-pentane) afforded product **S9** as colourless oil (0.24 g, 1.1 mmol, 52%).

R_f (*n*-pentane) = 0.31. **^{1}H NMR** (500 MHz, $CDCl_3$, 299 K) δ [ppm] = 7.63 (d, $^4J_{HH}$ = 1.9 Hz, 1H, H3), 7.38 (dd, $^3J_{HH}$ = 8.1 Hz, $^4J_{HH}$ = 1.9 Hz, 1H, H1), 7.14 (dd, $^3J_{HH}$ = 8.0 Hz, $^4J_{HH}$ = 1.0 Hz, 1H, H10), 5.77 (dd, $^4J_{FH}$ = 5.4 Hz, $^4J_{HH}$ = 1.6 Hz, 1H, H6^a), 5.63 (dddt, $^2J_{FH}$ = 54.9 Hz, $^3J_{HH}$ = 6.7, 2.6 Hz, $^4J_{HH}$ = 1.5 Hz, 1H,

H7), 5.54 (dd, $^4J_{FH}$ = 5.3 Hz, $^4J_{HH}$ = 1.4 Hz, 1H, H6[b]), 3.26 (ddd, $^3J_{FH}$ = 24.1 Hz, $^2J_{HH}$ = 17.7 Hz, $^3J_{HH}$ = 6.6 Hz, 1H, H8[a]), 3.09 (dddd, $^3J_{FH}$ = 28.5 Hz, $^2J_{HH}$ = 17.6 Hz, $^3J_{HH}$ = 2.6, $^4J_{HH}$ = 1.0 Hz, 1H, H8[b]). **^{13}C NMR** (126 MHz, $CDCl_3$, 299 K) δ [ppm] = 147.4 (d, $^2J_{FC}$ = 13.9 Hz, 1C, C5), 140.9 (d, $^3J_{FC}$ = 0.9 Hz, 1C, C9), 140.4 (d, $^3J_{FC}$ = 2.3 Hz, 1C, C4), 132.2 (1C, C1), 127.1 (d, $^4J_{FC}$ = 1.6 Hz, H10), 124.4 (1C, C3), 121.4 (1C, C2), 111.2 (d, $^3J_{FC}$ = 7.8 Hz, 1C, C6), 93.9 (d, $^1J_{FC}$ = 178.5 Hz, 1C, C7), 37.9 (d, $^2J_{FC}$ = 23.3 Hz, 1C, C8). **^{19}F NMR** (470 MHz, $CDCl_3$, 299 K) δ [ppm] = -166.7 (dddt, $^2J_{FH}$ = 54.8 Hz, $^3J_{FH}$ = 28.7, 23.3 Hz, $^4J_{FH}$ = 5.4 Hz, 1F, F7). **^{19}F{^{1}H} NMR** (470 MHz, $CDCl_3$, 299 K) δ [ppm] = -166.7 (s, 1F, F7). **IR** (ATR) $\tilde{\upsilon}$ [cm^{-1}] = 2958 (w), 1650 (w), 1596 (w), 1569 (w), 1466 (m), 1424 (m), 1316 (w), 1271 (m), 1255 (w), 1214 (w), 1096 (s), 1060 (m), 1015 (s), 1000 (s), 902 (m), 876 (m), 853 (m), 804 (s), 769 (w), 697 (m). **GC-MS** (EI) *m/z* $[M]^+$, calcd. for $C_{10}H_8BrF^+$: 225.9788, found: 225.9790.

4-Bromo-2-fluoro-1,1-dimethoxy-2,3-dihydro-1H-indene (S10)

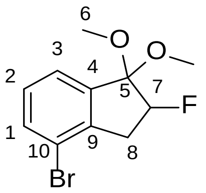

According to **general procedure B**, 4-bromo-2,3-dihydro-1H-inden-1-one (2.1 g, 10 mmol, 1.0 eq.) and Selectfluor (4.2 g, 12 mmol, 1.2 eq.) were refluxed in MeOH (70 mL) for 16 h. Column chromatography purification (*n*-pentane:EtOAc 19:1) afforded product **S10** as colourless oil (1.5 g, 5.6 mmol, 56%).

R_f (*n*-pentane:EtOAc 9:1) = 0.89. **^{1}H NMR** (599 MHz, CD_2Cl_2, 299 K) δ [ppm] = 7.53 (d, $^3J_{HH}$ = 8.0 Hz, 1H, H1), 7.38 (d, $^3J_{HH}$ = 7.5 Hz, 1H, H3), 7.18 (dd, $^3J_{HH}$ = 8.0, 7.5 Hz, 1H, H2), 5.16 (ddt, $^2J_{FH}$ = 52.4 Hz, $^3J_{HH}$ = 4.6, 1.1 Hz, 1H, H7), 3.46 (s, 3H, H6[a]), 3.26 (ddd, $^3J_{FH}$ = 30.5 Hz, $^2J_{HH}$ = 17.3 Hz, $^3J_{HH}$ = 4.6 Hz, 1H, H8[a]), 3.16 (s, 3H, H6[b]), 3.05 (ddd, $^3J_{FH}$ = 22.6 Hz, $^2J_{HH}$ = 17.3 Hz, $^3J_{HH}$ = 1.1 Hz, 1H, H8[b]). **^{13}C NMR** (151 MHz, CD_2Cl_2, 299 K) δ [ppm] = 141.0 (d, $^3J_{FC}$ = 0.9 Hz, 1C, C9), 140.8 (1C, C4), 133.3 (1C, C1), 128.8 (1C, C2), 124.7 (1C, C3), 121.3 (1C, C10), 109.0 (d, $^2J_{FC}$ = 20.0 Hz, 1C, C5), 94.6 (d, $^1J_{FC}$ = 187.2 Hz, 1C, C7), 50.9 (d, $^4J_{FC}$ = 2.0 Hz, 1C, C6[a]), 49.7 (d, $^4J_{FC}$ = 2.0 Hz, 1C, C6[b]), 38.0 (d, $^2J_{FC}$ = 23.3 Hz, 1C, C8). **^{19}F NMR** (564 MHz, CD_2Cl_2, 299 K) δ [ppm] = -191.1 (ddd, $^2J_{FH}$ = 52.7 Hz, $^3J_{FH}$ = 30.5, 22.5 Hz, 1F, F7). **^{19}F{^{1}H} NMR** (564 MHz, CD_2Cl_2, 299 K) δ [ppm] = -191.1 (s, 1F, F7). **IR** (ATR) $\tilde{\upsilon}$ [cm^{-1}] = 2942 (w), 2836 (w), 1737 (w), 1574 (w), 1455 (m), 1419 (w), 1313 (m), 1270 (w), 1213 (m), 1195 (m), 1183 (w), 1157 (m), 1143 (s), 1114 (m), 1079 (s), 1061 (s), 991 (m), 966 (m), 866 (w),

835 (w), 783 (s), 755 (w), 723 (m), 755 (w), 723 (m), 691 (w), 665 (m). **HRMS** (ESI) *m/z* $[M+Na]^+$, calcd. for $C_{11}H_{12}O_2BrFNa^+$: 296.9897, found: 296.9898.

4-Bromo-2-fluoro-1-methylene-2,3-dihydro-1*H*-indene (S11)

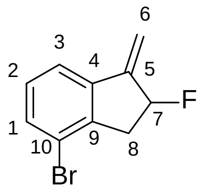

4-Bromo-2-fluoro-1,1-dimethoxy-2,3-dihydro-1H-indene (**S10**) (1.4 g, 5.0 mmol, 1.0 eq.) was dissolved in MeOH (14 mL) and conc. HCl (9 mL) was added dropwise. The mixture was stirred for 1 h. H_2O was added and the aqueous layer was extracted with Et_2O (3x). The combined organic layers were washed with sat. $NaHCO_3$, dried over $MgSO_4$ and filtered. The solvent was removed *in vacuo* to afford 4-bromo-2-fluoro-2,3-dihydro-1H-inden-1-one as white solid, which was used without purification in the next step.

According to **general procedure C**, Ph_3PMeBr (2.8 g, 7.5 mmol, 1.5 eq.) and KO^tBu (0.84 g, 7.5 mmol, 1.5 eq.) were dissolved in THF (15 mL). 4-Bromo-2-fluoro-2,3-dihydro-1*H*-inden-1-one was added and the reaction mixture was stirred for 16 h. Column chromatography purification (*n*-pentane) afforded product **S11** as white solid (0.52 g, 2.3 mmol, 46% over two steps).

R_f (*n*-pentane:EtOAc 10:1) = 0.33. **M.p.** 61 – 62 °C. **^{1}H NMR** (599 MHz, $CDCl_3$, 299 K) δ [ppm] = 7.45 (d, $^3J_{HH}$ = 7.7 Hz, 1H, H1), 7.43 (d, $^3J_{HH}$ = 8.0 Hz, 1H, H3), 7.14 (dd, $^3J_{HH}$ = 8.0, 7.7 Hz, 1H, H2), 5.79 (dd, $^4J_{FH}$ = 5.6 Hz, $^4J_{HH}$ = 1.6 Hz, 1H, H6^a), 5.64 (dddt, $^2J_{FH}$ = 55.0 Hz, $^3J_{HH}$ = 6.7, 1.9 Hz, $^4J_{HH}$ = 1.4 Hz, 1H, H7), 5.54 (dd, $^4J_{FH}$ = 5.6 Hz, $^4J_{HH}$ = 1.3 Hz, 1H, H6^b), 3.31 (ddd, $^3J_{FH}$ = 23.9 Hz, $^2J_{HH}$ = 18.0 Hz, $^3J_{HH}$ = 6.7 Hz, 1H, H8^a), 3.16 (ddd, $^3J_{FH}$ = 29.0 Hz, $^2J_{HH}$ = 18.0 Hz, $^3J_{HH}$ = 2.2 Hz, 1H, H8^b). **^{13}C NMR** (151 MHz, $CDCl_3$, 299 K) δ [ppm] = 148.4 (d, $^2J_{FC}$ = 13.7 Hz, 1C, C5), 142.5 (1C, C9), 140.1 (d, $^3J_{FC}$ = 2.3 Hz, 1C, C4), 132.1 (1C, C3), 129.2 (1C, C2), 120.8 (d, $^4J_{FC}$ = 1.7 Hz, 1C, C10), 120.0 (1C, C1), 111.8 (d, $^3J_{FC}$ = 7.9 Hz, 1C, C6), 92.9 (d, $^1J_{FC}$ = 178.0 Hz, 1C, C7), 39.7 (d, $^2J_{FC}$ = 23.9 Hz, 1C, C8). **^{19}F NMR** (564 MHz, $CDCl_3$, 299 K) δ [ppm] = -165.9 (dddt, $^2J_{FH}$ = 54.8 Hz, $^3J_{FH}$ = 29.0, 23.5 Hz, $^4J_{FH}$ = 5.6 Hz, 1F, F7). **^{19}F{^{1}H} NMR** (564 MHz, $CDCl_3$, 299 K) δ [ppm] = -165.9 (s, 1F, F7). **IR** (ATR) $\tilde{\nu}$ [cm^{-1}] = 2976 (w), 2928 (w), 1931 (w), 1868 (w), 1832 (w), 1808 (w), 1691 (w), 1645 (w), 1560 (m), 1462 (m), 1453 (m), 1414 (m), 1336 (w), 1318 (w), 1286 (m), 1248 (m), 1179 (m), 1170 (m), 1139 (w),

1099 (m), 1054 (w), 1016 (m), 1003 (s), 966 (m), 935 (m), 914 (s), 837 (s), 800 (s), 785 (s), 735 (s), 696 (s), 670 (m). **GC-MS** (EI) *m/z* $[M]^+$, calcd. for $C_{10}H_8BrF^+$: 225.9788, found: 225.9793.

2-Fluoro-5-methyl-2,3-dihydro-1*H*-inden-1-one (S12)

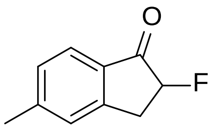

According to **general procedure B**, 5-methyl-1-indanone (1.5 g, 10 mmol, 1.0 eq.) and Selectfluor (4.2 g, 12 mmol, 1.2 eq.) were refluxed in MeOH (70 mL) for 16 h. Column chromatography purification (*n*-pentane:EtOAc 20:1) afforded product **S12** as pale yellow solid (1.4 g, 8.3 mmol, 83%).

R_f (*n*-pentane:EtOAc 10:1) = 0.34. **M.p.** 83 – 84 °C. **^{1}H NMR** (400 MHz, $CDCl_3$, 299 K) δ [ppm] = 7.69 (m, 1H), 7.28 – 7.20 (m, 2H), 5.25 (ddd, J = 51.0, 7.7, 4.3 Hz, 1H), 3.57 (ddd, J = 17.0, 7.5, 7.3 Hz, 1H), 3.18 (ddd, J = 22.1, 17.1, 4.3 Hz, 1H), 2.46 (s, 3H). **^{19}F NMR** (377 MHz, $CDCl_3$, 299 K) δ [ppm] = -193.4 (ddd, J = 51.1, 23.4, 7.3 Hz, 1F). **^{19}F{^{1}H} NMR** (377 MHz, $CDCl_3$, 299 K) δ [ppm] = -193.4 (s, 1F). **HRMS** (ESI) *m/z* $[M+Na]^+$, calcd. for $C_{10}H_9OFNa^+$: 187.0530, found: 187.0527. Analytic data in agreement with the literature.[5]

2-Fluoro-5-methyl-1-methylene-2,3-dihydro-1*H*-indene (S13)

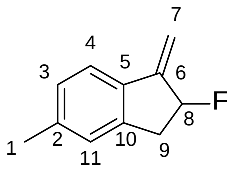

According to **general procedure C**, Ph_3PMeBr (3.3 g, 9.0 mmol, 1.5 eq.) and KO^{t}Bu (1.0 g, 9.0 mmol, 1.5 eq.) were dissolved in THF (20 mL). 2-Fluoro-5-methyl-2,3-dihydro-1*H*-inden-1-one (**S12**) (0.99 g, 6.0 mmol, 1.0 eq.) was added and the reaction mixture was stirred for 16 h. Column chromatography purification (*n*-pentane) afforded product **S13** as colourless oil (0.82 g, 5.0 mmol, 84%).

R_f (*n*-pentane) = 0.23. **^{1}H NMR** (500 MHz, $CDCl_3$, 299 K) δ [ppm] = 7.41 (d, $^3J_{HH}$ = 7.8 Hz, 1H, H4), 7.08 (s, 1H, H11), 7.07 (d, $^3J_{HH}$ = 7.8 Hz, 1H, H3), 5.73 – 5.52 (m, 2H, H7^a/H8), 5.43 (dd, $^4J_{FH}$ = 5.4, J = 1.3 Hz, 1H, H7^b), 3.29 (ddd, $^3J_{FH}$ = 23.5 Hz, $^2J_{HH}$ = 17.4 Hz, J = 6.8 Hz, 1H, H9^a), 3.11 (ddd, $^3J_{FH}$ = 28.9 Hz, $^2J_{HH}$ = 17.5 Hz, J = 2.5 Hz, 1H, H9^b), 2.36 (s, 3H, H1). **^{13}C NMR** (126 MHz, $CDCl_3$, 299 K) δ [ppm] = 148.4 (d, $^2J_{FC}$ = 13.5 Hz, 1C, C6), 142.3 (1C, C2), 139.6 (1C, C10), 135.6 (d, $^3J_{FC}$ = 2.2 Hz, 1C, C5), 128.4 (1C, C3), 126.1 (d, $^4J_{FC}$ = 1.6 Hz, 1C, C11), 121.0 (1C, C4), 108.4 (d, $^3J_{FC}$ = 7.9 Hz, 1C, C7), 94.4 (d,

${}^{1}J_{FC}$ = 177.4 Hz, 1C, C8), 38.1 (d, ${}^{2}J_{FC}$ = 22.9 Hz, 1C, C9), 21.7 (1C, C1). **^{19}F NMR** (470 MHz, $CDCl_3$, 299 K) δ [ppm] = -167.1 (dddt, J = 54.9 Hz, ${}^{3}J_{FH}$ = 28.5, 22.6 Hz, ${}^{4}J_{FH}$ = 5.5 Hz, 1F, F8). **^{19}F{^{1}H} NMR** (470 MHz, $CDCl_3$, 299 K) δ [ppm] = -167.1 (s, 1F, F8). **IR** (ATR) $\tilde{\nu}$ [cm^{-1}] = 2955 (w), 1647 (w), 1613 (w), 1488 (w), 1422 (w), 1320 (m), 1169 (w), 1143 (w), 1087 (w), 1016 (m), 932 (w), 890 (m), 820 (s), 759 (w), 751 (w), 672 (w). **GC-MS** (EI) *m/z* $[M]^+$, calcd. for $C_{11}H_{11}F^+$: 162.0839, found: 162.0835.

1-Oxo-2,3-dihydro-1*H*-inden-5-yl trifluoromethanesulfonate (S14)

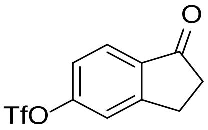

According to a modified procedure[7], to an oven-dried Schlenk flask was added 5-hydroxy-2,3-dihydro-1*H*-inden-1-one (2.2 g, 15 mmol, 1.0 eq.) and pyridine (15 mL) under argon. The mixture was cooled to 0 °C and trifluoromethanesulfonic anhydride (2.7 mL, 17 mmol, 1.2 eq.) was added dropwise. The mixture was slowly warmed to room temperature and was stirred for 15 h. The mixture was diluted with Et_2O and washed with sat. aq. $CuSO_4$ (3x), H_2O and brine. The organic layer was dried over $MgSO_4$, filtered and the solvent was removed *in vacuo*. Column chromatography purification (*n*-pentane:EtOAc 14:1) afforded product **S14** as yellow solid (3.1 g, 11 mmol, 74%).

R_f (*n*-pentane:EtOAc 9:1) = 0.44. **M.p.** 45 – 46 °C. **^{1}H NMR** (400 MHz, $CDCl_3$, 299 K) δ [ppm] = 7.83 (d, J = 8.4 Hz, 1H), 7.41 (d, J = 2.2 Hz, 1H), 7.27 (dd, J = 8.5, 2.2 Hz, 1H), 3.54 – 2.94 (m, 2H), 3.14 – 2.44 (m, 2H). **^{19}F NMR** (377 MHz, $CDCl_3$, 299 K) δ [ppm] = -72.8 (s, 3F). **^{19}F{^{1}H} NMR** (377 MHz, $CDCl_3$, 299 K) δ [ppm] = -72.8 (s, 3F). **HRMS** (ESI) *m/z* $[M+H]^+$, calcd. for $C_{10}H_7O_4SF_3H^+$: 281.0090, found: 281.0090. Analytic data in agreement with the literature.[8]

2-Fluoro-1,1-dimethoxy-2,3-dihydro-1*H*-inden-5-yl trifluoromethanesulfonate (S15)

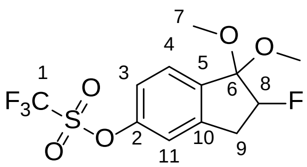

According to **general procedure B**, 1-oxo-2,3-dihydro-1H-inden-5-yl trifluoromethanesulfonate (**S14**) (2.8 g, 10 mmol, 1.0 eq.) and Selectfluor (4.2 g, 12 mmol, 1.2 eq.) were refluxed in MeOH (70 mL) for 16 h. Column chromatography purification (*n*-pentane:EtOAc 19:1) afforded product **S15** as white solid (2.7 g, 7.8 mmol, 78%).

R_f (*n*-pentane:EtOAc 9:1) = 0.63. **M.p.** 45 – 46 °C. **^{1}H NMR** (599 MHz, CD_2Cl_2, 299 K) *δ* [ppm] = 7.50 (d, $^3J_{HH}$ = 8.1 Hz, 1H, H4), 7.21 (d, $^4J_{HH}$ = 2.3 Hz, 1H, H11), 7.20 (dd, $^3J_{HH}$ = 8.1 Hz, $^4J_{HH}$ = 2.3 Hz, 1H, H3), 5.17 (ddd, $^2J_{FH}$ = 52.4 Hz, $^3J_{HH}$ = 4.5, 1.7 Hz, 1H, H8), 3.46 (s, 3H, H7[a]), 3.31 (ddd, $^3J_{FH}$ = 31.1 Hz, $^2J_{HH}$ = 17.1 Hz, $^3J_{HH}$ = 4.6 Hz, 1H, H9[a]), 3.17 (s, 3H, H7[b]), 3.05 (ddd, $^3J_{FH}$ = 21.9 Hz, $^2J_{HH}$ = 17.3 Hz, $^3J_{HH}$ = 1.7 Hz, 1H, H9[b]). **^{13}C NMR** (151 MHz, CD_2Cl_2, 299 K) *δ* [ppm] = 151.1 (1C, C2), 143.4 (1C, C10), 139.2 (1C, C5), 127.4 (1C, C4), 120.1 (1C, C3), 119.5 (1C, C11), 118.3 (q, $^1J_{FC}$ = 320.8 Hz, 1C, C1), 107.7 (d, $^2J_{FC}$ = 20 Hz, 1C, C6), 95.4 (d, $^1J_{FC}$ = 187.7 Hz, 1C, C8), 50.9 (d, $^4J_{FC}$ = 2.0 Hz, 1C, C7[a]), 49.7 (d, $^4J_{FC}$ = 2.0 Hz, 1C, C7[b]), 36.8 (d, $^2J_{FC}$ = 23.0 Hz, 1C, C9). **^{19}F NMR** (564 MHz, CD_2Cl_2, 299 K) *δ* [ppm] = -73.2 (s, 3F, F1), -191.6 (ddd, $^2J_{FH}$ = 52.8 Hz, $^3J_{FH}$ = 31.1, 21.8 Hz, 1F, F8). **^{19}F{^{1}H} NMR** (564 MHz, CD_2Cl_2, 299 K) *δ* [ppm] = -73.2 (s, 3F, F1), -191.6 (s, 1F, F8). **IR** (ATR) $\tilde{\nu}$ [cm^{-1}] = 3069 (w), 2975 (w), 2949 (w), 2839 (w), 1731 (w), 1616 (w), 1595 (w), 1482 (w), 1456 (w), 1414 (m), 1343 (w), 1317 (m), 1289 (w), 1251 (m), 1208 (s), 1139 (s), 1111 (m), 1092 (m), 1072 (m), 1054 (s), 995 (w), 975 (m), 957 (m), 931 (s), 892 (m), 865 (s), 837 (m), 829 (m), 775 (m), 765 (m), 741 (m), 729 (m), 654 (s). **HRMS** (ESI) *m/z* [M+Na]$^+$, calcd. for $C_{12}H_{12}O_5SF_4Na^+$: 367.0233, found: 367.0234.

2-Fluoro-1-oxo-2,3-dihydro-1*H*-inden-5-yl trifluoromethanesulfonate (S16)

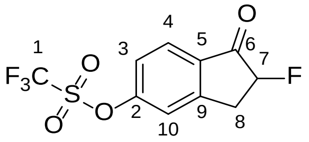

2-Fluoro-1,1-dimethoxy-2,3-dihydro-1*H*-inden-5-yl trifluoromethanesulfonate (**S15**) (2.4 g, 7.0 mmol, 1.0 eq.) was dissolved in MeOH (20 mL) and conc. HCl (13 mL) was added dropwise. The mixture was stirred for 45 min. H_2O was added and the aqueous layer was extracted with Et_2O (3x). The combined organic layers were washed with sat. $NaHCO_3$ and dried over $MgSO_4$. The solvent was removed *in vacuo* to afford product **S16** as white solid (2.1 g, 7.0 mmol, quant.).

R_f (*n*-pentane:EtOAc 9:1) = 0.45. **M.p.** 78 – 79 °C. **^{1}H NMR** (599 MHz, $CDCl_3$, 299 K) *δ* [ppm] = 7.91 (d, $^3J_{HH}$ = 8.4 Hz, 1H, H4), 7.41 (d, $^4J_{HH}$ = 1.9 Hz 1H, H10), 7.35 (dd, $^3J_{HH}$ = 8.3 Hz, $^4J_{HH}$ = 2.0 Hz, 1H, H3), 5.31 (ddd, $^2J_{FH}$ = 50.6 Hz, $^3J_{HH}$ = 7.8, 4.2 Hz, 1H, H7), 3.69 (ddd, $^2J_{HH}$ = 17.5 Hz, $^3J_{HH}$ = 7.8 Hz, $^3J_{FH}$ = 7.7 Hz, 1H, H8[a]), 3.30 (ddd, $^3J_{FH}$ = 22.6 Hz, $^2J_{HH}$ = 17.5 Hz, $^3J_{HH}$ = 4.3 Hz, 1H, H8[b]). **^{13}C NMR** (151 MHz, $CDCl_3$, 299 K) *δ* [ppm] = 197.9 (d, $^2J_{FC}$ = 15.0 Hz, 1C, C6), 154.7 (1C, C2), 152.1 (d,

$^3J_{FC}$ = 5.6 Hz, 1C, C9), 133.8 (d, $^3J_{FC}$ = 1.6 Hz, 1C, C5), 127.3 (d, $^4J_{FC}$ = 1.4 Hz, 1C, C4), 122.3 (1C, C3), 120.1 (d, $^4J_{FC}$ = 1.7 Hz, 1C, C10), 118.8 (q, $^1J_{FC}$ = 320.8 Hz, 1C, C1), 90.1 (d, $^1J_{FC}$ = 191.8 Hz, 1C, C7), 33.6 (d, $^2J_{FC}$ = 22.3 Hz, 1C, C8). **^{19}F NMR** (564 MHz, $CDCl_3$, 299 K) δ [ppm] = -72.7 (s, 3F, F1), -193.0 (ddd, $^2J_{FH}$ = 50.8 Hz, $^3J_{FH}$ = 23.0, 7.7 Hz, 1F, F7). **^{19}F{^{1}H} NMR** (564 MHz, $CDCl_3$, 299 K) δ [ppm] = -72.7 (s, 3F, F1), -193.0 (s, 1F, F7). **IR** (ATR) $\tilde{\nu}$ [cm^{-1}] = 3090 (w), 2981 (w), 1730 (m), 1616 (m), 1589 (m), 1475 (w), 1421 (s), 1356 (w), 1321 (m), 1270 (w), 1201 (s), 1129 (s), 1081 (s), 996 (m), 974 (w), 934 (s), 909 (m), 885 (m), 838 (s), 794 (m), 747 (m), 666 (m). **HRMS** (ESI) *m/z* [M+Na]$^+$, calcd. for $C_{10}H_6O_4SF_4Na^+$: 320.9815, found 320.9816.

2-Fluoro-1-methylene-2,3-dihydro-1*H*-inden-5-yl trifluoromethanesulfonate (S17)

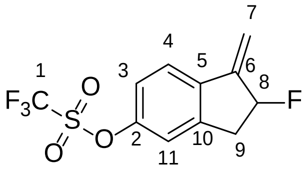

According to **general procedure C**, Ph_3PMeBr (2.8 g, 7.5 mmol, 1.5 eq.) and KO^{t}Bu (0.84 g, 7.5 mmol, 1.5 eq.) were dissolved in THF (15 mL). 2-Fluoro-1-oxo-2,3-dihydro-1*H*-inden-5-yl trifluoromethanesulfonate (**S16**) (1.5 g, 5.0 mmol, 1.0 eq.) was added and the reaction mixture was stirred for 16 h. Column chromatography purification (*n*-pentane:EtOAc 100:3) afforded product **S17** as yellow oil (0.33 g, 1.1 mmol, 22%).

R_f (*n*-pentane:EtOAc 9:1) = 0.43. **^{1}H NMR** (599 MHz, $CDCl_3$, 299 K) δ [ppm] = 7.56 (d, $^3J_{HH}$ = 8.4 Hz, 1H, H4), 7.19 (s, 1H, H11), 7.16 (d, $^3J_{HH}$ = 8.4 Hz, 1H, H3), 5.82 (dd, $^4J_{FH}$ = 5.5 Hz, $^4J_{HH}$ = 1.6 Hz, 1H, H7^a), 5.66 (dddt, $^2J_{FH}$ = 54.7 Hz, $^3J_{HH}$ = 6.6, 1.8 Hz, $^4J_{HH}$ = 1.6 Hz, 1H, H8), 5.60 (dd, $^4J_{FH}$ = 5.6 Hz, $^4J_{HH}$ = 1.6 Hz, 1H, H7^b), 3.36 (ddd, $^3J_{FH}$ = 24.0 Hz, $^2J_{HH}$ = 17.8 Hz, $^3J_{HH}$ = 6.7 Hz, 1H, H9^a), 3.19 (ddd, $^3J_{FH}$ = 28.3 Hz, $^2J_{HH}$ = 18.0 Hz, $^3J_{HH}$ = 1.8 Hz, 1H, H9^b). **^{13}C NMR** (151 MHz, $CDCl_3$, 299 K) δ [ppm] = 150.1 (1C, C2), 146.7 (d, $^2J_{FC}$ = 13.9 Hz, 1C, C6), 144.4 (1C, C10), 138.5 (d, $^3J_{FC}$ = 2.3 Hz, 1C, C5), 122.8 (1C, C4), 120.7 (1C, C3), 118.9 (q, $^1J_{FC}$ = 320.8 Hz, 1C, C1), 118.7 (d, $^4J_{FC}$ = 1.5 Hz, 1C, C11), 112.0 (d, $^3J_{FC}$ = 7.8 Hz, 1C, C7), 93.7 (d, $^1J_{FC}$ = 178.9 Hz, 1C, C8), 38.2 (d, $^2J_{FC}$ = 23.7 Hz, 1C, C9). **^{19}F NMR** (564 MHz, $CDCl_3$, 299 K) δ [ppm] = -72.8 (s, 3F, F1), -166.2 (dddt, $^2J_{FH}$ = 54.7 Hz, $^3J_{FH}$ = 28.3, 23.8 Hz, $^4J_{FH}$ = 5.4 Hz, 1F, F8). **^{19}F{^{1}H} NMR** (564 MHz, $CDCl_3$, 299 K) δ [ppm] = -72.8 (s, 3F, F1), -166.2 (s, 1F, F8). **IR** (ATR) $\tilde{\nu}$ [cm^{-1}] = 2979 (w), 1611 (w), 1587 (w), 1476 (m), 1418 (s), 1323

(w), 1250 (m), 1205 (s), 1137 (s), 1112 (s), 1086 (m), 1017 (m), 929 (s), 877 (m), 850 (s), 837 (s), 762 (w), 726 (m). **GC-MS** (EI) *m/z* $[M]^+$, calcd. for $C_{11}H_8F_4O_3S^+$: 296.0125, found: 296.0124.

2-Fluoro-1,1-dimethoxy-6-(trifluoromethyl)-2,3-dihydro-1*H*-indene (S18)

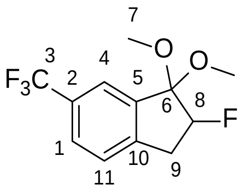

According to **general procedure B**, 6-(trifluoromethyl)-2,3-dihydro-1*H*-inden-1-one (1.7 g, 8.6 mmol, 1.0 eq.) and Selectfluor (3.6 g, 10 mmol, 1.2 eq.) were refluxed in MeOH (70 mL) for 16 h. Column chromatography purification (*n*-pentane:Et_2O 19:1) afforded product **S18** as yellow liquid (2.0 g, 7.5 mmol, 87%).

R$_f$ (*n*-pentane:Et_2O 19:1) = 0.90. **^{1}H NMR** (599 MHz, CD_2Cl_2, 299 K) δ [ppm] = 7.67 (s, 1H, H4), 7.62 (d, $^3J_{HH}$ = 7.9 Hz, 1H, H1), 7.42 (d, $^3J_{HH}$ = 7.9 Hz, 1H, H11), 5.19 (ddd, $^2J_{FH}$ = 52.5 Hz, $^3J_{HH}$ = 4.6, 1.4 Hz, 1H, H8), 3.47 (s, 3H, 7^a), 3.33 (ddd, $^3J_{FH}$ = 31.4 Hz, $^2J_{HH}$ = 17.2 Hz, $^3J_{HH}$ = 4.6 Hz, 1H, $H9^a$), 3.18 (s, 3H, 7^b), 3.07 (ddd, $^3J_{FH}$ = 21.8 Hz, $^2J_{HH}$ = 17.2 Hz, $^3J_{HH}$ = 1.4 Hz 1H, $H9^b$). **^{13}C NMR** (151 MHz, CD_2Cl_2, 299 K) δ [ppm] = 144.9 (1C, C10), 139.8 (1C, C5), 129.3 (q, $^2J_{FC}$ = 32.3 Hz, 1C, C2), 127.4 (q, $^3J_{FC}$ = 3.8 Hz, 1C, C1), 126.9 (1C, C11), 124.9 (q, $^1J_{FC}$ = 272.0 Hz, 1C, C3), 122.6 (q, $^3J_{FC}$ = 3.9 Hz, 1C, C4), 108.1 (d, $^2J_{FC}$ = 20.0 Hz, 1C, C6), 95.5 (d, $^1J_{FC}$ = 187.3 Hz, 1C, C8), 50.9 (d, $^4J_{FC}$ = 2.0 Hz, 1C, $C7^b$), 49.7 (d, $^4J_{FC}$ = 2.0 Hz, 1C, $C7^a$), 36.9 (d, $^2J_{FC}$ = 22.8 Hz, 1C, C9). **^{19}F NMR** (564 MHz, CD_2Cl_2, 299 K) δ [ppm] = -62.5 (s, 3F, F3), -191.9 (ddd, $^2J_{FH}$ = 52.8 Hz, $^3J_{FH}$ = 31.4, 21.8 Hz, 1F, F8). **^{19}F{^{1}H} NMR** (564 MHz, CD_2Cl_2, 299 K) δ [ppm] = -62.5 (s, 3F, F3), -191.9 (s, 1F, F8). **IR** (ATR) $\tilde{\nu}$ [cm^{-1}] = 2947 (w), 2838 (w), 1626 (w), 1439 (w), 1418 (w), 1337 (m), 1306 (m), 1263 (m), 1214 (m), 1159 (m), 1120 (s), 1106 (s), 1082 (m), 1057 (s), 985 (m), 970 (m), 904 (m), 877 (w), 826 (m), 770 (w), 758 (w), 729 (m), 702 (m), 654 (m). **HRMS** (ESI) *m/z* $[M+Na]^+$, calcd. for $C_{12}H_{12}O_2F_4Na^+$: 287.0666, found: 287.0665.

2-Fluoro-6-(trifluoromethyl)-2,3-dihydro-1*H*-inden-1one (S19)

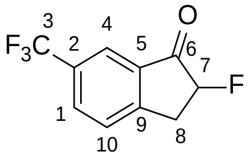

2-Fluoro-1,1-dimethoxy-6-(trifluoromethyl)-2,3-dihydro-1*H*-indene (**S18**) (1.8 g, 7.0 mmol, 1.0 eq.) was dissolved in MeOH (20 mL) and conc. HCl (13 mL) was added dropwise. The mixture was stirred for 60 min. H_2O was added and the aqueous layer was extracted with Et_2O (3x). The combined organic layers were washed with sat.

$NaHCO_3$, dried over $MgSO_4$ and filtered. The solvent was removed *in vacuo* to afford product **S19** as pale yellow solid (1.5 g, 7.0 mmol, quant.).

R_f (*n*-pentane:EtOAc 9:1) = 0.31. **M.p.** 52 – 53 °C. **^{1}H NMR** (599 MHz, $CDCl_3$, 299 K) δ [ppm] = 8.02 (d, $^4J_{HH}$ = 1.9 Hz, 1H, H4), 7.90 (dd, $^3J_{HH}$ = 8.1 Hz, $^4J_{HH}$ = 1.8 Hz, 1H, H1), 7.62 (d, $^3J_{HH}$ = 8.1 Hz, 1H, H10), 5.30 (ddd, $^2J_{FH}$ = 50.7 Hz, $^3J_{HH}$ = 7.8, 4.3 Hz, 1H, H7), 3.70 (ddd, $^2J_{HH}$ = 17.6 Hz, $^3J_{HH}$ = 7.8 Hz, $^3J_{FH}$ = 7.7 Hz, 1H, H8^a), 3.28 (ddd, $^3J_{FH}$ = 22.5 Hz, $^2J_{HH}$ = 17.4 Hz, $^3J_{HH}$ = 4.3 Hz, 1H, H8^b). **^{13}C NMR** (151 MHz, $CDCl_3$, 299 K) δ [ppm] = 198.8 (d, $^2J_{FC}$ = 15.1 Hz, 1C, C6), 153.1 (m, 1C, C9), 134.4 (d, $^3J_{FC}$ = 1.5 Hz, C5), 132.8 (q, $^3J_{FC}$ = 3.4 Hz, 1C, C1), 131.4 (q, $^2J_{FC}$ = 33.3 Hz, 1C, C2), 127.8 (d, $^4J_{FC}$ = 1.7 Hz, 1C, C10), 123.5 (q, $^1J_{FC}$ = 272.7 Hz, 1C, C3), 122.0 (qd, $^3J_{FC}$ = 3.9, $^4J_{FC}$ = 1.5 Hz, 1C, C4), 90.3 (d, $^1J_{FC}$ = 191.4 Hz, 1C, C7), 33.6 (d, $^2J_{FC}$ = 21.9 Hz, 1C, C8). **^{19}F NMR** (564 MHz, $CDCl_3$, 299 K) δ [ppm] = -62.8 (s, 3F, F3), -193.6 (ddd, $^2J_{FH}$ = 50.8 Hz, $^3J_{FH}$ = 23.3, 7.7 Hz, 1F, F7). **^{19}F{^{1}H} NMR** (564 MHz, $CDCl_3$, 299 K) δ [ppm] = -62.8 (s, 3F, F3), -193.6 (s, 1F, F7). **IR** (ATR) $\tilde{\nu}$ [cm^{-1}] = 3093 (w), 3057 (w), 1735 (s), 1625 (m), 1583 (w), 1445 (w), 1357 (w), 1330 (s), 1272 (s), 1233 (m), 1197 (m), 1180 (m), 1116 (s), 1090 (s), 1052 (s), 1006 (m), 987 (w), 908 (w), 929 (w), 852 (m), 798 (m), 765 (w), 726 (m), 704 (s). **GC-MS** (EI) *m/z* [M]$^+$, calcd. for $C_{10}H_6OF_4^+$: 218.0349, found 218.0342.

2-Fluoro-1-methylene-6-(trifluoromethyl)-2,3-dihydro-1*H*-indene (S20)

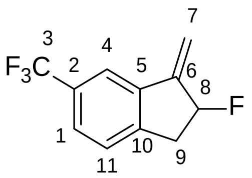

According to **general procedure C**, Ph_3PMeBr (1.9 g, 5.3 mmol, 1.5 eq.) and KO^{t}Bu (0.59 g, 5.3 mmol, 1.5 eq.) were dissolved in THF (11 mL). 2-Fluoro-6-(trifluoromethyl)-2,3-dihydro-1*H*-inden-1one (**S19**) (0.76 g, 3.5 mmol, 1.0 eq.) was added and the reaction mixture was stirred for 3 days. Column chromatography purification (*n*-pentane) afforded product **S20** as colourless liquid (0.23 g, 1.1 mmol, 31%).

R_f (*n*-pentane) = 0.27. **^{1}H NMR** (599 MHz, $CDCl_3$, 299 K) δ [ppm] = 7.75 (s, 1H, H4), 7.53 (d, $^3J_{HH}$ = 7.9 Hz, 1H, H1), 7.38 (d, $^3J_{HH}$ = 7.9 Hz, 1H, H11), 5.88 (dd, $^4J_{FH}$ = 5.5 Hz, $^4J_{HH}$ = 1.5 Hz, 1H, H7^a), 5.68 (ddtd, J = 55.0 Hz, $^3J_{HH}$ = 6.6, $^4J_{HH}$ = 1.5 Hz, $^3J_{HH}$ = 1.3 Hz, 1H, H8), 5.62 (dd, J = 5.3 Hz, $^4J_{HH}$ = 1.5 Hz, 1H, H7^b), 3.36 (ddd, J = 24.2 Hz, $^2J_{HH}$ = 17.8 Hz, $^3J_{HH}$ = 6.7 Hz, 1H, H9^a), 3.20 (ddd, J = 28.8, $^2J_{HH}$ = 18.3 Hz, $^3J_{HH}$ = 1.3 Hz, 1H, H9^b). **^{13}C NMR** (151 MHz, $CDCl_3$, 299 K) δ [ppm] = 147.3 (d, $^2J_{FC}$ = 13.9 Hz, 1C, C6),

145.8 (1C, C10), 138.9 (d, $^3J_{FC}$ = 2.3 Hz, 1C, C5), 130.1 (q, $^2J_{FC}$ = 32.4 Hz, 1C, C2), 126.1 (q, $^3J_{FC}$ = 3.8 Hz, 1C, C1), 126.0 (d, $^4J_{FC}$ = 1.5 Hz, 1C, C11), 124.3 (q, $^1J_{FC}$ = 272.2 Hz, 1C, C3), 118.3 (q, $^3J_{FC}$ = 3.9 Hz, 1C, C4), 111.9 (d, $^3J_{FC}$ = 7.8 Hz, 1C, C7), 93.7 (d, $^1J_{FC}$ = 178.5 Hz, 1C, C8), 38.3 (d, $^2J_{FC}$ = 23.4 Hz, 1C, C9). **^{19}F NMR** (564 MHz, $CDCl_3$, 299 K) *δ* [ppm] = -62.4 (s, 3F, F3), -166.3 – -166.6 (m, 1F, F8). **^{19}F{^{1}H} NMR** (564 MHz, $CDCl_3$, 299 K) *δ* [ppm] = -62.4 (s, 3F, F3), -166.5 (s, 1F, F8). **IR** (ATR) $\tilde{\nu}$ [cm^{-1}] = 2963 (w), 1622 (w), 1446 (w), 1343 (m), 1322 (s), 1276 (s), 1162 (m), 1118 (s), 1095 (s), 1060 (m), 1016 (m), 958 (w), 897 (m), 821 (m), 741 (w), 694 (m). **GC-MS** (EI) *m/z* [M]$^+$, calcd. for $C_{11}H_8F_4^+$: 216.0557, found 216.0552.

2-Fluoro-1,1-dimethoxy-2,3-dihydro-1*H*-indene-4-carbonitrile (S21)

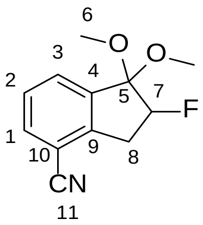

According to **general procedure B**, 1-oxo-2,3-dihydro-1*H*-indene-4-carbonitrile (1.6 g, 10 mmol, 1.0 eq.) and Selectfluor (4.2 g, 12 mmol, 1.2 eq.) were refluxed in MeOH (70 mL) for 16 h. Column chromatography purification (*n*-pentane:EtOAc 14:1) afforded product **S21** as white solid (1.8 g, 8.3 mmol, 83%).

R_f (*n*-pentane:EtOAc 9:1) = 0.40. **M.p.** 51 – 52 °C. **^{1}H NMR** (599 MHz, CD_2Cl_2, 299 K) *δ* [ppm] = 7.65 (d, $^3J_{HH}$ = 7.6 Hz, 1H, H1), 7.64 (d, $^3J_{HH}$ = 7.6 Hz, 1H, H3), 7.41 (dd, $^3J_{HH}$ = 7.6, 7.7 Hz, 1H, H2), 5.21 (ddd, $^2J_{FH}$ = 52.2 Hz, $^3J_{HH}$ = 4.5, 1.5 Hz, 1H, H7), 3.47 (s, 3H, H6^a), 3.42 (ddd, $^3J_{FH}$ = 31.1 Hz, $^2J_{HH}$ = 17.5 Hz, $^3J_{HH}$ = 4.5 Hz, 1H, H8^a), 3.21 (ddd, $^3J_{FH}$ = 21.9 Hz, $^2J_{HH}$ = 17.6 Hz, $^3J_{HH}$ = 1.5 Hz, 1H, H8^b), 3.16 (s, 3H, H6^b). **^{13}C NMR** (151 MHz, CD_2Cl_2, 299 K) *δ* [ppm] = 144.8 (1C, C9), 140.4 (1C, C4), 133.7 (1C, C1), 130.0 (1C, C3), 127.8 (1C, C2), 117.4 (1C, C11), 110.8 (1C, C10), 108.3 (d, $^2J_{FC}$ = 20.1 Hz, 1C, C5), 94.9 (d, $^1J_{FC}$ = 188.1 Hz, 1C, C7), 51.0 (d, $^4J_{FC}$ = 2.0 Hz, 1C, C6^a), 49.7 (d, $^4J_{FC}$ = 2.0 Hz, 1C, C6^b), 36.5 (d, $^2J_{FC}$ = 23.2 Hz, 1C, C8). **^{19}F NMR** (564MHz, CD_2Cl_2, 299 K) *δ* [ppm] = -191.6 (ddd, $^2J_{FH}$ = 52.5 Hz, $^3J_{FH}$ = 31.0, 21.7 Hz, 1F, F7). **^{19}F{^{1}H} NMR** (564MHz, CD_2Cl_2, 299 K) *δ* [ppm] = -191.6 (s, 1F, F7). **IR** (ATR) $\tilde{\nu}$ [cm^{-1}] = 2944 (w), 2837 (w), 2228 (w), 1595 (w), 1471 (w), 1448 (w), 1418 (w), 1319 (m), 1276 (w), 1213 (m), 1188 (m), 1142 (s), 1085 (s), 1058 (s), 1002 (m), 968 (s), 923 (w), 866 (m), 798 (s), 729 (s), 687 (m). **GC-MS** (EI) *m/z* [M]$^+$, calcd. for $C_{11}H_9NOF^+$: 190.0663, found: 190.0668.

2-Fluoro-1-methylene-2,3-dihydro-1*H*-indene-5-carbonitrile (S22)

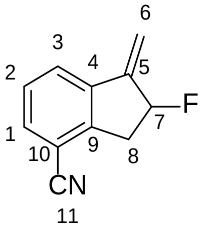

2-Fluoro-1,1-dimethoxy-2,3-dihydro-1*H*-indene-4-carbonitrile (**S21**) (1.5 g, 7.0 mmol, 1.0 eq.) was dissolved in MeOH (16 mL) and conc. HCl (12 mL) was added dropwise. The mixture was stirred for 2.5 h. H_2O was added and the aqueous layer was extracted with Et_2O (3x). The combined organic layers were washed with sat. $NaHCO_3$, dried over $MgSO_4$ and filtered. The solvent was removed *in vacuo* to afford 2-fluoro-1-oxo-2,3-dihydro-1*H*-indene-4-carbonitrile as white solid and was used without any purification in the next step.

According to **general procedure C**, Ph_3PMeBr (3.8 g, 11 mmol, 1.5 eq.) and KO^{t}Bu (1.2 g, 11 mmol, 1.5 eq.) were dissolved in THF (22 mL). 2-Fluoro-1-oxo-2,3-dihydro-1*H*-indene-4-carbonitrile was added and the reaction mixture was stirred for 16 h. Column chromatography purification (*n*-pentane:EtOAc 19:1) afforded product **S22** as white solid (67 mg, 0.36 mmol, 5% over two steps).

R$_f$ (*n*-pentane:EtOAc 9:1) = 0.54. **M.p.** 85 – 86 °C. **^{1}H NMR** (500 MHz, $CDCl_3$, 299 K) δ [ppm] = 7.71 (dd, $^3J_{HH}$ = 7.9 Hz, $^5J_{HH}$ = 0.5 Hz, 1H, H3), 7.56 (dt, $^3J_{HH}$ = 7.7 Hz, $^5J_{HH}$ = 0.8 Hz, 1H, H1), 7.37 (ddt, $^3J_{HH}$ = 7.7, 7.9 Hz, $^6J_{HH}$ = 0.9 Hz, 1H, H2), 5.88 (dd, $^4J_{FH}$ = 5.5 Hz, $^4J_{HH}$ = 1.5 Hz, 1H, H6^a), 5.69 (dddt, $^2J_{FH}$ = 54.5 Hz, $^3J_{HH}$ = 6.6, 2.2 Hz, $^4J_{HH}$ = 1.4 Hz, 1H, H7), 5.65 (dd, $^4J_{FH}$ = 5.6 Hz, $^4J_{HH}$ = 1.3 Hz, 1H, H6^b), 3.48 (ddddd, $^3J_{FH}$ = 23.9 Hz, $^2J_{HH}$ = 18.3 HZ, $^3J_{HH}$ = 6.6 Hz, $^5J_{HH}$ = 0.7, 0.8 Hz, 1H, H8^a), 3.34 (dddd, $^3J_{FH}$ = 28.0 Hz, $^2J_{HH}$ = 18.3 Hz, $^3J_{HH}$ = 2.7 Hz, $^6J_{HH}$ = 0.9 Hz, 1H, H8^b). **^{13}C NMR** (126 MHz, $CDCl_3$, 299 K) δ [ppm] = 146.9 (d, $^2J_{FC}$ = 14.2 Hz, 1C, C5), 146.0 (1C, C9), 139.6 (d, $^3J_{FC}$ = 2.3 Hz, 1C, C4), 132.5 (1C, C1), 128.2 (1C, C2), 125.4 (1C, C3), 117.1 (1C, C11), 112.8 (d, $^3J_{FC}$ = 8.0 Hz, 1C, C6), 110.1 (d, $^4J_{FC}$ = 1.5 Hz, 1C, C10), 92.9 (d, $^1J_{FC}$ = 179.4 Hz, 1C, C7), 37.9 (d, $^2J_{FC}$ = 24.2 Hz, 1C, C8). **^{19}F NMR** (470 MHz, $CDCl_3$, 299 K) δ [ppm] = -165.9 (ddt, $^2J_{FH}$ = 54.5 Hz, $^3J_{FH}$ = 28.0, 23.9 Hz, $^4J_{FH}$ = 5.5 Hz, 1F, F7). **^{19}F{^{1}H} NMR** (470 MHz, $CDCl_3$, 299 K) δ [ppm] = -165.9 (s, 1F, F7). **IR** (ATR) $\tilde{\nu}$ [cm^{-1}] = 2925 (w), 2226 (m), 1843 (w), 1720 (w), 1652 (w), 1472 (w), 1437 (w), 1415 (m), 1326 (m), 1287 (m), 1253 (m), 1180 (m), 1009 (s), 980 (w), 936 (m), 924 (m), 913 (m), 840 (m), 820 (m), 803 (s), 744 (m), 703 (m), 664 (m). **GC-MS** (EI) *m/z* [M]$^+$, calcd. for $C_{11}H_8NF^+$: 173.0636, found: 173.0632.

2-(3-Oxo-2,3-dihydro-1*H*-inden-5-yl)isoindoline-1,3-dione (S23)

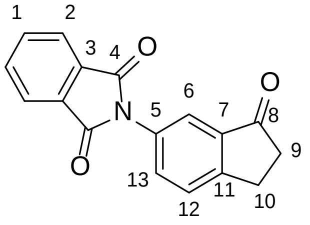

According to a modified procedure [9], 6-aminoindanone (2.2 g, 15 mmol, 1.2 eq.) was dissolved in CH_2Cl_2 (80 mL) and phthalic anhydride (1.9 g, 13 mmol, 1.0 eq.) was added. The mixture was stirred for 30 min and filtered. The solid residue was dried *in vacuo* and dissolved in Ac_2O (50 mL). The mixture was heated to 90 °C and NaOAc (0.16 g, 1.9 mmol, 15 mol%) was added. The reaction was stirred for additional 4 h. The solvent was removed *in vacuo* and the residue suspended in CH_2Cl_2 and filtered to remove NaOAc. The solvent was removed *in vacuo* and the crude product was recrystallised from MeOH to afford product **S23** as green crystals (2.7 g, 9.9 mmol, 79%).

R_f (CH_2Cl_2:EtOAc 19:1) = 0.29. **M.p.** 226 – 227 °C. **^{1}H NMR** (500 MHz, $CDCl_3$, 299 K) δ [ppm] = 8.03 – 7.93 (m, 2H, H2), 7.84 (dd, $^4J_{HH}$ = 2.0 Hz, $^5J_{HH}$ = 0.7 Hz, 1H, H6), 7.85 – 7.75 (m, 2H, H1), 7.67 (dd, $^3J_{HH}$ = 8.1 Hz, $^4J_{HH}$ = 2.0 Hz, 1H, H13), 7.62 (dd, $^3J_{HH}$ = 8.2 Hz, $^5J_{HH}$ = 0.8 Hz, 1H, H12), 3.41 – 3.05 (m, 2H, H9), 2.94 – 2.62 (m, 2H, H10). **^{13}C NMR** (126 MHz, $CDCl_3$, 299 K) δ [ppm] = 205.8 (1C, C8), 167.1 (2C, C4), 154.6 (1C, C11), 138.2 (1C, C7), 134.7 (2C, C1), 132.8 (C1, C13), 131.8 (2C, C3), 131.3 (1C, C5), 127.6 (1C, C12), 124.1 (2C, C2), 122.1 (1C, C6), 36.8 (1C, C9), 25.9 (1C, C10). **IR** (ATR) $\tilde{\nu}$ [cm^{-1}] = 3106 (w), 1775, (w), 1703 (s), 1617 (m), 1585 (m), 1491 (m), 1470 (m), 1441 (m), 1405 (w), 1358 (s), 1327 (m), 1278 (m), 1251 (m), 1217 (m), 1182 (m), 1127 (m), 1068 (s), 1034 (m), 875 (m), 848 (s), 794 (m), 752 (w), 714 (s), 708 (s). **HRMS** (ESI) *m/z* [M+Na]$^+$, calcd. for $C_{17}H_{11}NO_3Na^+$: 300.0631, found: 300.0631.

2-(2-Fluoro-3-oxo-2,3-dihydro-1*H*-inden-5-yl)isoindoline-1,3-dione (S24)

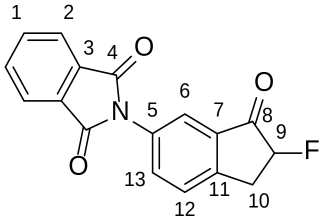

According to **general procedure B**, 2-(3-oxo-2,3-dihydro-1*H*-inden-5-yl)isoindoline-1,3-dione (**S23**) (1.4 g, 5.0 mmol, 1.0 eq.) and Selectfluor (2.1 g, 6.0 mmol, 1.2 eq.) were refluxed in CH_3CN (35 mL) for 16 h. Column chromatography purification (CH_2Cl_2:EtOAc 34:1) afforded product **S24** as yellow solid (0.54 g, 1.8 mmol, 37%).

R$_f$ (CH_2Cl_2:EtOAc 19:1) = 0.63. **M.p.** 227 – 228 °C (decomposition). **^{1}H NMR** (500 MHz, $CDCl_3$, 299 K) δ [ppm] = 7.98 (m, 2H, H2), 7.92 (d, $^4J_{HH}$ = 2.0 Hz, 1H, H6), 7.83 (m, 2H, H1), 7.78 (dd, $^3J_{HH}$ = 8.2 Hz, $^4J_{HH}$ = 2.0 Hz, 1H, H13), 7.61 (dd, $^3J_{HH}$ = 8.2 Hz, $^4J_{HH}$ = 0.9 Hz, 1H, H12), 5.33 (ddd, $^2J_{FH}$ = 50.9 Hz, $^3J_{HH}$ = 7.8, 4.4 Hz, 1H, H9), 3.68 (ddd, $^2J_{HH}$ = 17.2 Hz, $^3J_{HH}$ = 7.8 Hz, $^3J_{FH}$ = 7.2 Hz, 1H, H10^a), 3.29 (dddd, $^3J_{FH}$ = 22.8 Hz, $^2J_{HH}$ = 17.1 Hz, $^3J_{HH}$ = 4.4 Hz, $^4J_{HH}$ = 1.1 Hz, 1H, H10^b). **^{13}C NMR** (126 MHz, $CDCl_3$, 299 K) δ [ppm] = 199.0 (d, $^2J_{FC}$ = 15.0 Hz, 1C, C8), 166.9 (2C, C4), 148.9 (d, $^3J_{FC}$ = 5.7 Hz, 1C, C11), 134.9 (1C, C5), 134.9 (2C, C1) 134.3 (1C, C13), 132.3 (2C, C3), 131.7 (1C, C7), 127.7 (d, $^4J_{FC}$ = 1.8 Hz, 1C, C12), 124.2 (2C, C2), 122.8 (d, $^4J_{FC}$ = 1.4 Hz, 1C, C6), 90.7 (d, $^1J_{FC}$ = 191.5 Hz, 1C, C9), 33.5 (d, $^2J_{FC}$ = 21.7 Hz, 1C, C10). **^{19}F NMR** (470 MHz, $CDCl_3$, 299 K) δ [ppm] = -193.5 (ddd, $^2J_{FH}$ = 50.9 Hz, $^3J_{FH}$ = 23.1, 7.2 Hz, 1F, F9). **^{19}F{^{1}H} NMR** (470 MHz, $CDCl_3$, 299 K) δ [ppm] = -193.5 (s, 1F, F9). **IR** (ATR) $\tilde{\nu}$ [cm^{-1}] = 3107 (w), 3045 (w), 1776 (w), 1703 (s), 1617 (w), 1492 (m), 1442 (m), 1406 (w), 1368 (s), 1328 (m), 1279 (m), 1252 (w), 1218 (w), 1182 (m), 1128 (w), 1091 (m), 1070 (m), 1035 (w), 876 (m), 849 (m), 794 (w), 715 (s), 709 (s). **HRMS** (ESI) *m/z* [M+Na]$^+$, calcd. for $C_{17}H_{10}NO_3FNa^+$: 318.0537, found: 318.0538.

2-(2-Fluoro-3-methylene-2,3-dihydro-1*H*-inden-5-yl)isoindoline-1,3-dione (S25)

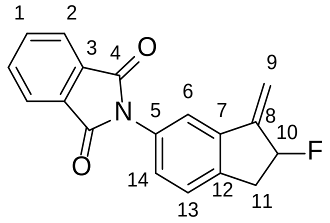

According to **general procedure C**, Ph_3PMeBr (1.1 g, 3.2 mmol, 2.0 eq.) and KO^{t}Bu (0.36 g, 3.2 mmol, 2.0 eq.) were dissolved in THF (8 mL). 2-(2-Fluoro-3-oxo-2,3-dihydro-1*H*-inden-5-yl)isoindoline-1,3-dione (**S24**) (0.47 g, 1.6 mmol, 1.0 eq.) was added and the reaction mixture was stirred for 3 days. Column chromatography purification (CH_2Cl_2) afforded product **S25** as white solid (0.21 g, 0.72 mmol, 45%).

R$_f$ (CH_2Cl_2) = 0.36. **M.p.** 145 – 146 °C (decomposition). **^{1}H NMR** (500 MHz, $CDCl_3$, 299 K) δ [ppm] = 7.96 (m, 2H, H2), 7.80 (m, 2H, H1), 7.56 (d, $^4J_{HH}$ = 1.9 Hz, 1H, H6), 7.40 (dd, $^3J_{HH}$ = 8.1 Hz, $^4J_{HH}$ = 0.9 Hz, 1H, H13), 7.33 (dd, $^3J_{HH}$ = 8.0 Hz, $^4J_{HH}$ = 1.9 Hz, 1H, H14), 5.80 (dd, $^4J_{FH}$ = 5.3 Hz, $^4J_{HH}$ = 1.6 Hz, 1H, H9^a), 5.69 (dddt, $^2J_{FH}$ = 55.0 Hz, $^3J_{HH}$ = 6.7, 2.8 Hz, $^4J_{HH}$ = 1.5 Hz, 1H, H10), 5.56 (dd, $^4J_{FH}$ = 5.3 Hz, $^4J_{HH}$ = 1.3 Hz, 1H, H9^b), 3.37 (ddd, $^3J_{FH}$ = 23.1 Hz, $^2J_{HH}$ = 17.7 Hz, $^3J_{HH}$ = 6.7 Hz, 1H, H11^a), 3.20 (dddd,

$^3J_{FH}$ = 28.6 Hz, $^2J_{HH}$ = 17.7 Hz, $^3J_{HH}$ = 2.7 Hz, $^4J_{HH}$ = 0.9 Hz, 1H, H11[b]). **^{13}C NMR** (126 MHz, $CDCl_3$, 299 K) δ [ppm] = 167.4 (2C, C4), 147.7 (d, $^2J_{FC}$ = 14.1 Hz, 1C, C8), 142.1 (1C, C12), 139.4 (d, $^3J_{FC}$ = 2.4 Hz, 1C, C7), 134.6 (2C, C1), 131.9 (2C, C3), 131.1 (1C, C5), 127.7 (1C, C14), 126.2 (d, $^4J_{FC}$ = 1.5 Hz, 1C, C13), 123.9 (2C, C2), 119.7 (1C, C6), 111.1 (d, $^3J_{FC}$ = 7.7 Hz, 1C, C9), 94.1 (d, $^1J_{FC}$ = 178.6 Hz, 1C, C10), 38.1 (d, $^2J_{FC}$ = 23.3 Hz, 1C, C11). **^{19}F NMR** (470 MHz, $CDCl_3$, 299 K) δ [ppm] = -166.9 (dddt, $^2J_{FH}$ = 55.0 Hz, $^3J_{FH}$ = 28.4, 23.1 Hz, $^4J_{FH}$ = 5.3 Hz, 1F, F10). **^{19}F{^{1}H} NMR** (470 MHz, $CDCl_3$, 299 K) δ [ppm] = -166.9 (1F, F10). **IR** (ATR) $\tilde{\nu}$ [cm^{-1}] = 3033 (w), 1764 (w), 1775 (w), 1714 (s), 1645 (w), 1610 (w), 1583 (w), 1488 (m), 1468 (w), 1450 (m), 1386 (s), 1289 (w), 1261 (w), 1217 (w), 1175 (w), 1107 (s), 1077 (m), 1016 (s), 963 (m), 926 (w), 896 (m), 885 (m), 874 (s), 814 (m), 787 (m), 766 (w), 745 (w), 713 (s), 707 (s), 682 (m), 652 (m). **HRMS** (ESI) *m/z* $[M+Na]^+$, calcd. for $C_{18}H_{12}NO_2FNa^+$: 316.0744, found: 316.0746.

Methyl (*E*)-3-(2-fluoro-2,3-dihydrospiro[indene-1,2'-[1,3-dioxolan]-5-yl)acrylate (S26)

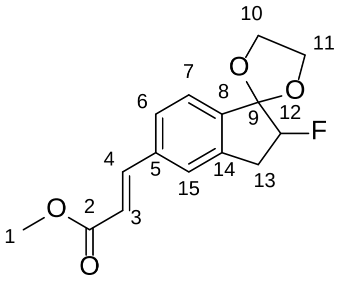

According to a modified procedure[10], 5-bromo-2-fluoro-2,3-dihydro-1*H*-inden-1-one (**S6**) (2.9 g, 13 mmol, 1.0 eq.) was added to toluene (150 mL), ethylene glycol (14 mL, 0.26 mol, 20 eq.) and *p*-toluenesulfonic acid (0.28 g, 1.5 mmol, 12 mol%) The reaction mixture was refluxed with azeotropic removal of H_2O using a Dean-Stark apparatus for 24 h. The mixture was cooled to room temperature and washed with a saturated solution of K_2CO_3 and brine. The organic layer was dried over $MgSO_4$, filtered and the solvent was removed *in vacuo.* 5-Bromo-2-fluoro-2,3-dihydrospiro[indene-1,2'-[1,3]dioxolane was obtained as the crude product and was used without further purification in the next step.

To an oven dried pressure tube was added $Pd(OAc)_2$ (0.22 g, 0.96 mmol, 7.5 mol%), PPh_3 (0.76 g, 2.9 mmol, 23 mol%) and DMF (26 mL) under argon. The mixture was heated to 90 °C and the crude 5-bromo-2-fluoro-2,3-dihydrospiro[indene-1,2'-[1,3]dioxolane, K_2CO_3 (3.5 g, 26 mmol, 2.0 eq.) and methyl acrylate (5.9 mL, 64 mmol, 5.0 eq.) were added. The mixture was stirred for 15 h, cooled to room temperature and diluted with EtOAc. The organic layer was washed with 1 M HCl, 5% aq. LiCl solution

and brine. The organic layer was dried over $MgSO_4$, filtered and the solvent was removed *in vacuo*. Column chromatography purification (*n*-pentane:EtOAc 19:1) afforded product **S26** as white solid (1.8 g, 6.5 mmol, 51% over two steps).

R$_f$ (*n*-pentane:EtOAc 9:1) = 0.16. **M.p.** 98 – 99 °C. **^{1}H NMR** (500 MHz, CD_2Cl_2, 299 K) δ [ppm] = 7.68 (d, $^3J_{HH}$ = 16.0 Hz, 1H, H4), 7.49 (d, $^3J_{HH}$ = 7.9 Hz, 1H, H6), 7.42 (s, 1H, H15), 7.41 (d, $^3J_{HH}$ = 8.1 Hz, 1H, H7), 6.47 (d, $^3J_{HH}$ = 16.0 Hz, 1H, H3), 5.05 (ddd, $^2J_{FH}$ = 52.9 Hz, $^3J_{HH}$ = 6.2, 4.5 Hz, 1H, H12), 4.32 – 4.10 (m, 4H, H10, H11), 3.78 (s, 3H, H1), 3.28 (ddd, $^3J_{FH}$ = 16.7 Hz, $^2J_{HH}$ = 16.4 Hz, $^3J_{HH}$ = 6.2 Hz, 1H, H13[a]), 3.07 (ddd, $^3J_{FH}$ = 20.9 Hz, $^2J_{HH}$ = 16.4 Hz, $^3J_{HH}$ = 4.6 Hz, 1H, H13[a]). **^{13}C NMR** (126 MHz, CD_2Cl_2, 299 K) δ [ppm] = 167.5 (1C, C2), 144.6 (1C, C4), 141.2 (d, $^3J_{FC}$ = 1.1 Hz, 1C, C8), 140.5 (d, $^3J_{FC}$ = 4.9 Hz, 1C, C14), 137.4 (d, $^5J_{HH}$ = 0.7 Hz, 1C, C5), 128.3 (1C, C6), 125.4 (d, $^4J_{FC}$ = 1.1 Hz, 1C, C15), 124.9 (d, $^4J_{FC}$ = 1.1 Hz, 1C, C7), 119.3 (1C, C3), 113.1 (d, $^2J_{FC}$ = 17.6 Hz, 1C, C9), 95.8 (d, $^1J_{FC}$ = 193.6 Hz, 1C, C12), 66.9 (d, $^4J_{FC}$ = 1.2 Hz, 1C, C10), 66.4 (C1, C11), 52.1 (1C, C1), 35.6 (d, $^2J_{FC}$ = 22.3 Hz, 1C, C13). **^{19}F NMR** (470 MHz, CD_2Cl_2, 299 K) δ [ppm] = -193.7 (ddd, $^2J_{FH}$ = 52.9 Hz, $^3J_{FH}$ = 20.5, 16.7 Hz, 1F, F12). **^{19}F{^{1}H} NMR** (470 MHz, CD_2Cl_2, 299 K) δ [ppm] = -193.7 (s, 1F, F12). **IR** (ATR) $\tilde{\nu}$ [cm^{-1}] = 3065 (w), 2949 (w), 2886 (w), 1701 (s), 1635 (s), 1579 (w), 1436 (m), 1335 (s), 1305 (s), 1274 (m), 1217 (s), 1195 (s), 1178 (s), 1162 (s), 1103 (m), 1057 (s), 1025 (m), 994 (s), 950 (s), 921 (m), 878 (w), 827 (s), 779 (w), 752 (w), 722 (m), 713 (m), 664 (w). **HRMS** (ESI) *m/z* [M+Na]$^+$, calcd. for $C_{15}H_{15}O_4FNa^+$: 301.0847, found 301.0834.

Methyl (*E*)-3-(2-fluoro-1-methylene-2,3-dihydro-1*H*-inden-5-yl)acrylate (S27)

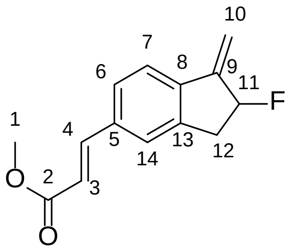

Methyl (*E*)-3-(2-fluoro-2,3-dihydrospiro[indene-1,2'-[1,3-dioxolan]-5-yl)acrylate (**S26**) (1.8 g, 6.3 mmol, 1.0 eq.) was dissolved in MeOH (25 mL) and conc. HCl (4 mL) was added dropwise. The mixture was stirred for 14 h and then filtered. The residue was dissolved in CH_2Cl_2 and washed with a saturated solution of $NaHCO_3$ and brine. The organic layer was dried over $MgSO_4$ and filtered. The solvent was removed *in vacuo* to afford methyl (*E*)-3-(2-fluoro-1-oxo-2,3-dihydro-1*H*-inden-5-yl)acrylate as white solid and was used without purification in the next step.

According to **general procedure C**, Ph_3PMeBr (3.4 g, 9.5 mmol, 1.5 eq.) and KO*t*Bu (1.1 g, 9.5 mmol, 1.5 eq.) were dissolved in THF (18 mL). Methyl (*E*)-3-(2-fluoro-1-oxo-2,3-dihydro-1*H*-inden-5-yl)acrylate was added and the reaction mixture was stirred for 21 h. Column chromatography purification (*n*-pentane:EtOAc 9:1) afforded product **S27** as white solid (0.22 g, 0.96 mmol, 15% over two steps).

R_f (*n*-pentane:EtOAc 9:1) = 0.35. **M.p.** 75 – 76 °C. **^{1}H NMR** (500 MHz, CD_2Cl_2, 299 K) δ [ppm] = 7.67 (d, $^3J_{HH}$ = 16.0 Hz, 1H, H4), 7.54 (d, $^3J_{HH}$ = 7.9 Hz, 1H, H7), 7.47 (s, 1H, H14), 7.45 (d, $^3J_{HH}$ = 8.6 Hz, 1H, H6), 6.45 (d, $^3J_{HH}$ = 16.0 Hz, 1H, H3), 5.85 (dd, $^4J_{FH}$ = 5.6 Hz, $^4J_{HH}$ = 1.6 Hz, 1H, H10^a), 5.65 (dddt, $^2J_{FH}$ = 55.1 Hz, $^3J_{HH}$ = 6.6, 2.2 Hz, $^4J_{HH}$ = 1.4 Hz, 1H, H11), 5.56 (dd, $^4J_{FH}$ = 5.5 Hz, $^4J_{HH}$ = 1.3 Hz, 1H, H10^b), 3.78 (s, 3H, H1), 3.34 (ddd, $^3J_{FH}$ = 24.4 Hz, $^2J_{HH}$ = 17.8 Hz, $^3J_{HH}$ = 6.7 Hz, 1H, H12^a), 3.14 (ddd $^3J_{FH}$ = 28.8 Hz, $^2J_{HH}$ = 17.7 Hz, $^3J_{HH}$ = 2.2 Hz, 1H, H12^b). **^{13}C NMR** (126 MHz, CD_2Cl_2, 299 K) δ [ppm] = 167.7 (C1, C2), 148.5 (d, $^2J_{FC}$ = 13.8 Hz, 1C, C9), 144.7 (1C, C4), 143.5 (1C, C13), 140.7 (d, $^3J_{FC}$ = 2.3 Hz, 1C, C8), 136.0 (1C, C5), 128.1 (1C, C6), 125.5 (d, $^4J_{FC}$ = 1.5 Hz, 1C, C14), 122.0 (1C, C7), 118.5 (1C, C3), 111.7 (d, $^3J_{FC}$ = 8.0 Hz, 1C, C10), 94.6 (d, $^1J_{FC}$ = 177.3 Hz, 1C, C11), 52.1 (1C, C1), 38.5 (d, $^2J_{FC}$ = 23.3 Hz, 1C, C12). **^{19}F NMR** (470 MHz, CD_2Cl_2, 299 K) δ [ppm] = -166.17 (dddt, $^2J_{FH}$ = 54.5 Hz, $^3J_{FH}$ = 29.5, 24.3 Hz, $^4J_{FH}$ = 5.6 Hz). **^{19}F{^{1}H} NMR** (470 MHz, CD_2Cl_2, 299 K) δ [ppm] = -166.2 (s, 1F, F11). **IR** (ATR) $\tilde{\nu}$ [cm^{-1}] = 3029 (w), 2948 (w), 1716 (s), 1633 (s), 1571 (w), 1489 (w), 1431 (m), 1344 (w), 1314 (m), 1302 (m), 1281 (m), 1260 (w), 1236 (m), 1206 (m), 1187 (m), 1170 (s), 1156 (s), 996 (s), 935 (m), 918 (w), 885 (m), 855 (m), 823 (s), 786 (m), 766 (m), 751 (w), 725 (w), 676 (w), 662 (w). **HRMS** (ESI) *m/z* $[M+Na]^+$, calcd. for $C_{14}H_{13}O_2FNa^+$: 255.0792, found: 255.0791.

2-Fluoro-3-phenyl-2,3-dihydro-1*H*-inden-1-one (S28)

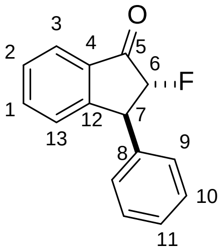

According to **general procedure B**, 3-phenyl-1-indanone (2.1 g, 10 mmol, 1.0 eq.) and Selectfluor (4.2 g, 12 mmol, 1.2 eq.) were refluxed in MeOH (70 mL) for 16 h. Column chromatography purification (*n*-pentane:EtOAc 20:1) afforded product **S28** as white solid (1.3 g, 5.5 mmol, 55%).

R_f (*n*-pentane:EtOAc 10:1) = 0.21. **M.p.** 72 – 73 °C. **^{1}H NMR** (500 MHz, $CDCl_3$, 299 K) δ [ppm] = 7.86 (m, 1H, H3), 7.67 (m, 1H, H2), 7.50 (m, 1H, H11), 7.41 (m, 2H, H10), 7.38 – 7.34 (m, 1H, H1), 7.26 –

7.22 (m, 3H, H9, H13), 5.14 (dd, $^2J_{FH}$ = 50.5 Hz, $^3J_{HH}$ = 5.0 Hz, 1H, H6), 4.55 (dd, $^3J_{FH}$ = 21.0 Hz, $^3J_{HH}$ = 5.0 Hz, 1H, H7). **^{13}C NMR** (126 MHz, $CDCl_3$, 299 K) *δ* [ppm] = 198.8 (d, $^2J_{FC}$ = 15.2 Hz, 1C, C5), 152.2 (d, $^3J_{FC}$ = 8.1 Hz, 1C, C4), 139.8 (1C, C12), 136.7 (d, $^5J_{FC}$ = 0.9 Hz. 1C, C2), 133.7 (d, $^3J_{FC}$ = 1.4 Hz, 1C, C8), 129.3 (2C, C10), 129.1 (1C, C11), 128.4 (2C, C9), 128.0 (1C, C1), 127.0 (d, $^4J_{FC}$ = 1.8 Hz, 1C, C13), 124.4 (d, $^4J_{FC}$ = 1.4 Hz, 1C, C3), 98.4 (d, $^1J_{FC}$ = 199.2 Hz, 1C, C6), 51.6 (d, $^2J_{FC}$ = 20.3 Hz, 1C, C7). **^{19}F NMR** (470 MHz, $CDCl_3$, 299 K) *δ* [ppm] = -194.0 (dd, $^2J_{FH}$ = 50.5 Hz, $^3J_{FH}$ =21.0 Hz, 1F, F6). **^{19}F{^{1}H} NMR** (470 MHz, $CDCl_3$, 299 K) *δ* [ppm] = -194.0 (s, 1F, F6). **IR** (ATR) $\tilde{\nu}$ [cm^{-1}] = 3421 (w), 2901 (w), 2901 (w), 1722 (s), 1606 (m), 1585 (m), 1564 (m), 1459 (m), 1343 (w), 1300 (w), 1206 (m), 1176 (w), 1106 (m), 1089 (s), 976 (s), 851 (w), 824 (w), 754 (s), 695 (s). **GC-MS** (EI) *m/z* [M]$^+$, calcd. for $C_{15}H_{11}FO^+$: 226.0794, found 226.0788.

2-Fluoro-1-methylene-3-phenyl-2,3-dihydro-1*H*-indene (S29)

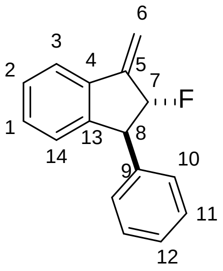

According to **general procedure C**, Ph_3PMeBr (2.2 g, 6.0 mmol, 1.5 eq.) and KO^{t}Bu (0.67 g, 6.0 mmol, 1.5 eq.) were dissolved in THF (15 mL). 2-Fluoro-3-phenyl-2,3-dihydro-1*H*-inden-1-one (**S28**) (0.91 g, 4.0 mmol, 1.0 eq.) was added and the reaction mixture was stirred for 16 h. Column chromatography purification (*n*-pentane) afforded product **S29** as pale yellow solid (0.44 mg, 2.0 mmol, 49%).

R_f (*n*-pentane) = 0.56. **M.p.** 54 – 55 °C. **^{1}H NMR** (500 MHz, $CDCl_3$, 299 K) *δ* [ppm] = 7.62 – 7.57 (m, 1H, H3), 7.36 – 7.31 (m, 3H, H2, H11), 7.29 (m, 2H, H1, H12), 7.17 – 7.14 (m, 2H, H10), 7.08 (m, 1H, H14), 5.81 (dd, $^4J_{FH}$ = 4.3 Hz, $^4J_{HH}$ = 1.9 Hz, 1H, H6[a]), 5.56 (ddt, $^2J_{FH}$ = 54.5 Hz, $^3J_{HH}$ = 3.6 Hz, $^4J_{HH}$ = 1.8 Hz, 1H, H7), 5.50 (dd, $^4J_{FH}$ = 4.2 Hz, $^4J_{HH}$ = 1.6 Hz, 1H, H6[b]), 4.52 (dd, $^3J_{FH}$ = 26.3 Hz, $^3J_{HH}$ = 3.7 Hz, 1H, H8). **^{13}C NMR** (126 MHz, $CDCl_3$, 299 K) *δ* [ppm] = 147.2 (d, $^2J_{FC}$ = 14.6 Hz, 1C, C5), 144.6 (d, $^3J_{FC}$ = 4.1 Hz, 1C, C13), 141.6 (d, $^3J_{FC}$ = 4.5 Hz, 1C, C9), 138.1 (d, $^3J_{FC}$ = 2.4 Hz, 1C, C4), 129.8 (1C, C1), 128.9 (2C, C11), 128.3 (2C, C10), 128.1 (1C, C2), 127.3 (1C, C12), 126.1 (d, $^4J_{FC}$ = 1.6 Hz, 1C, C14), 121.1 (1C, C3), 109.0 (d, $^3J_{FC}$ = 6.8 Hz, 1C, C6), 101.3 (d, $^1J_{FC}$ = 186.6 Hz, 1C, C7), 56.3 (d, $^2J_{FC}$ = 21.6 Hz, 1C, C8). **^{19}F NMR** (470 MHz, $CDCl_3$, 299 K) *δ* [ppm] = -169.9 (ddt, $^2J_{FH}$ = 54.5 Hz, $^3J_{FH}$ = 26.3 Hz, $^4J_{FH}$ = 4.2 Hz, 1F, F7). **^{19}F{^{1}H} NMR** (470 MHz, $CDCl_3$, 299 K) *δ* [ppm] = -169.9 (s, 1F, F7). **IR** (ATR)

$\tilde{\nu}$ [cm^{-1}] = 2908 (w), 1724 (s), 1603 (m), 1463 (m), 1344 (m), 1299 (w), 1241 (w), 1105 (m), 1089 (m), 976 (m), 917 (w), 861 (w). 824 (w), 748 (s), 703 (s), 694 (m). **GC-MS** (EI) *m/z* [M]$^+$, calcd. for $C_{16}H_{13}F^+$: 224.1001, found: 224.0992.

2-Fluoro-3,3-dimethyl-2,3-dihydro-1*H*-inden-1-one (S30)

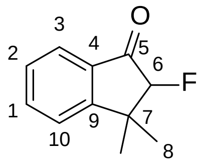

According to **general procedure B**, 3,3-dimethyl-1-indanone (1.6 g, 10 mmol, 1.0 eq.) and Selectfluor (4.2 g, 12 mmol, 1.2 eq.) were refluxed in MeOH (70 mL) for 16 h. Column chromatography purification (*n*-pentane:EtOAc 20:1) afforded product **S30** as colourless oil (1.2 g, 6.9 mmol, 69%).

R_f (*n*-pentane:EtOAc 10:1) = 0.26. **^{1}H NMR** (500 MHz, $CDCl_3$, 299 K) δ [ppm] = 7.76 (ddd, $^3J_{HH}$ = 7.7 Hz, $^4J_{HH}$ =1.3 Hz, $^5J_{HH}$ =0.8 Hz, 1H, H3), 7.70 (ddd, $^3J_{HH}$ = 7.6, 7.5 Hz, $^4J_{HH}$ =1.3 Hz, 1H, H1), 7.51 (ddd, $^3J_{HH}$ = 7.8, $^4J_{HH}$ = 0.9 Hz, $^5J_{HH}$ = 0.8 Hz, 1H, H10), 7.43 (ddd, $^3J_{HH}$ = 7.5, 7.7 Hz, $^4J_{HH}$ = 1.0 Hz, 1H, H2), 4.91 (d, $^2J_{FH}$ = 51.4 Hz, 1H, H6), 1.59 (s, 3H, H8^a), 1.26 (d, $^4J_{FH}$ = 4.0 Hz, 3H, H8^b). **^{13}C NMR** (126 MHz, $CDCl_3$, 299 K) δ [ppm] = 199.3 (d, $^2J_{FC}$ = 15.7 Hz, 1 C, C5), 158.9 (d, $^3J_{FC}$ = 5.2 Hz, 1C, C9), 136.5 (1C, C1), 131.8 (d, $^3J_{FC}$ = 2.4 Hz, 1C, C4), 128.5 (1C, C2), 124.4 (d, $^4J_{FC}$ = 1.6 Hz, 1C, C3), 123.8 (d, $^4J_{FC}$ = 2.0 Hz, 1C, C10), 99.1 (d, $^1J_{FC}$ = 201.9 Hz, 1C, C6), 42.4 (d, $^2J_{FC}$ = 17.9 Hz, 1C, C7), 25.9 (d, $^3J_{FC}$ = 9.6 Hz, 1C, C8^a), 25.49 (1C, C8^b). **^{19}F NMR** (470 MHz, $CDCl_3$, 299 K) δ [ppm] = -206.3 (dq, $^2J_{FH}$ = 51.4 Hz, $^4J_{FH}$ = 4.0 Hz, 1F, F6). **^{19}F{^{1}H} NMR** (470 MHz, $CDCl_3$, 299 K) δ [ppm] = -206.3 (s, 1F, F6). **IR** (ATR) $\tilde{\nu}$ [cm^{-1}] = 2968 (w), 2932 (w), 1726 (s), 1688 (w), 1604 (m), 1471 (w), 1369 (w), 1296 (w), 1235 (w), 1186 (w), 1124 (m), 1076 (m), 999 (m), 844 (m), 763 (s), 689 (m). **GC-MS** (EI) *m/z* [M]$^+$, calcd. for $C_{11}H_{11}OF^+$: 178.0788, found: 178.0788.

2-Fluoro-1,1-dimethyl-3-methylene-2,3-dihydro-1*H*-indene (S31)

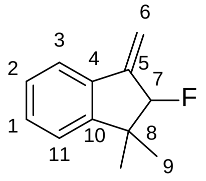

According to **general procedure C**, Ph_3PMeBr (2.8 g, 7.5 mmol, 1.5 eq.) and KO^{t}Bu (0.75 g, 7.5 mmol, 1.5 eq.) were dissolved in THF (12 mL). 2-Fluoro-3,3-dimethyl-2,3-dihydro-1*H*-inden-1-one (**S30**) (0.89 g, 5.0 mmol, 1.0 eq.) was added and the reaction

mixture was stirred for 16 h. Column chromatography purification (*n*-pentane) afforded product **S31** as colourless oil (0.58 g, 3.3 mmol, 66%).

R_f (*n*-pentane) = 0.63. **^{1}H NMR** (500 MHz, $CDCl_3$, 299 K) δ [ppm] = 7.49 – 7.46 (m, 1H, H3), 7.31 (m, 1H, H1), 7.27 – 7.25 (m, 1H, H2), 7.24 – 7.21 (m, 1H, H11), 5.70 (dd, $^4J_{FH}$ = 3.5 Hz, $^4J_{HH}$ = 1.9 Hz, 1H, H6[a]), 5.43 (dd, $^4J_{FH}$ = 3.6 Hz, $^4J_{HH}$ = 1.6 Hz, 1H, H6[b]), 5.15 (dt, $^2J_{FH}$ = 55.4 Hz, $^4J_{HH}$ = 1.8 Hz, 1H, H7), 1.33 (s, 3H, H9[a]), 1.27 (d, $^4J_{FH}$ = 3.8 Hz, 3H, H9[b]). **^{13}C NMR** (126 MHz, $CDCl_3$, 299 K) δ [ppm] = 151.2 (d, $^3J_{FC}$ = 2.3 Hz, 1C, C10), 146.8 (d, $^2J_{FC}$ = 15.4 Hz, 1C, C5), 136.1 (d, $^3J_{FC}$ = 3.4 Hz, 1C, C4), 129.7 (1C, C1), 127.5 (d, $^5J_{FC}$ = 0.8 Hz, 1C, C2), 122.9 (d, $^4J_{FC}$ = 1.7 Hz, 1C, C11), 121.3 (1C, C3), 108.3 (d, $^3J_{FC}$ = 6.3 Hz, 1C, C6), 101.7 (d, $^1J_{FC}$ = 189.7 Hz, 1C, C7), 45.5 (d, $^2J_{FC}$ = 18.3 Hz, 1C, C8), 26.9 (d, $^3J_{FC}$ = 2.1 Hz, 1C, C9[a]), 22.5 (d, $^3J_{FC}$ = 11.7 Hz, 1C, C9[b]). **^{19}F NMR** (470 MHz, $CDCl_3$, 299 K) δ [ppm] = -187.3 (dqt, $^2J_{FH}$ = 55.4 Hz, $^4J_{FH}$ = 3.8, 3.5 Hz, 1F, F7). **^{19}F{^{1}H} NMR** (470 MHz, $CDCl_3$, 299 K) δ [ppm] = -187.3 (s, 1F, F7). **IR** (ATR) $\tilde{\upsilon}$ [cm^{-1}] = 2963 (w), 2869 (w), 1654 (w), 1470 (m), 1410 (w), 1365 (w), 1321 (w), 1193 (w), 1130 (w), 1047 (m), 1019 (m), 986 (m), 889 (m), 785 (m), 764 (s), 679 (w), 652 (m). **GC-MS** (EI) *m/z* [M]$^+$, calcd. for $C_{12}H_{13}F^+$: 176.0996, found: 176.0995.

2,2-Difluoro-2,3-dihydro-1*H*-inden-1-one (S32)

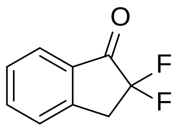

According to a modified procedure[11], indanone (1.3 g, 10 mmol, 1.0 eq.) was dissolved in cyclohexane (20 mL). To this mixture *n*-butylamine (1.2 mL, 12 mmol, 1.2 eq.) and TFA (5 drops) were added. The reaction mixture was refluxed with azeotropic removal of H_2O using a Dean-Stark apparatus for 6 h. The solvent was removed and Et_2O was added to the crude reaction mixture. A saturated solution of $NaHCO_3$ was added and the aqueous solution was extracted with Et_2O (3x). The combined organic layers were washed with brine, dried over $MgSO_4$, filtered and the solvent was removed *in vacuo*. *N*-Butyl-2,3-dihydro-1*H*-inden-1-imine was obtained as the crude product and was used without purification in the next step.

According to a modified procedure[11], crude *N*-butyl-2,3-dihydro-1*H*-inden-1-imine was dissolved in CH_3CN (90 mL). Na_2SO_4 (2.1 g, 15 mmol, 1.5 eq.) and Selectfluor (11 g, 30 mmol, 3.0 eq.) were added and the reaction mixture was refluxed for 16 h. Conc. HCl was added and the mixture was stirred for

additional 15 min. The solvent was removed and Et_2O was added to the crude reaction mixture. A saturated solution of $NaHCO_3$ was added and the aqueous solution was extracted with Et_2O (3x). The combined organic layers were washed with brine, dried over $MgSO_4$, filtered and the solvent was removed *in vacuo*. Column chromatography purification (*n*-pentane:EtOAc 20:1) afforded product **S32** as pale yellow oil (0.51 g, 3.0 mmol, 30% over two steps).

R$_f$ (*n*-pentane:EtOAc 10:1) = 0.31. **^{1}H NMR** (400 MHz, $CDCl_3$, 299 K) δ [ppm] = 7.87 (m, 1H), 7.74 (m, 1H), 7.49 (m, 2H), 3.57 (t, J = 12.7 Hz, 2H). **^{19}F NMR** (377 MHz, $CDCl_3$, 299 K) δ [ppm] = -111.6 (t, J = 12.8, 2F). **^{19}F{^{1}H} NMR** (377 MHz, $CDCl_3$, 299 K) δ [ppm] = -111.6 (s, 2F). **GC-MS** (EI) *m/z* $[M]^+$, calcd. for $C_9H_6OF_2^+$: 168.0382, found: 168.0379. Analytic data in agreement with the literature.[11]

2,2-Difluoro-1-methylene-2,3-dihydro-1*H*-indene (S33)

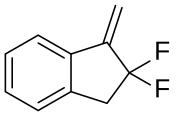

According to **general procedure C**, Ph_3PMeBr (1.1 g, 3.0 mmol, 1.5 eq.) and KO^{t}Bu (0.34 g, 3.0 mmol, 1.5 eq.) were dissolved in THF (6 mL). 2,2-Difluoro-2,3-dihydro-1*H*-inden-1-one (**S32**) (0.34 g, 2.0 mmol, 1.0 eq.) was added and the reaction mixture was stirred for 16 h. Column chromatography purification (*n*-pentane) afforded product **S33** as colourless oil (0.22 g, 1.3 mmol, 66%).

R$_f$ (*n*-pentane) = 0.55. **^{1}H NMR** (400 MHz, $CDCl_3$, 299 K) δ [ppm] = 7.55 – 7.47 (m, 1H), 7.37 – 7.21 (m, 3H), 5.80 (t, J = 3.5 Hz, 1H), 5.67 (t, J = 3.3 Hz, 1H), 3.45 (t, J = 14.2 Hz, 2H). **^{19}F NMR** (377 MHz, $CDCl_3$, 299 K) δ [ppm] = -93.5 (tt, J = 14.3, 3.3 Hz, 2F). **^{19}F{^{1}H} NMR** (377 MHz, $CDCl_3$, 299 K) δ [ppm] = -93.5 (s, 2F). **GC-MS** (EI) *m/z* $[M]^+$, calcd. for $C_{10}H_8F_2^+$: 166.0589, found: 166.0583. Analytic data in agreement with the literature.[12]

2-Chloro-2,3-dihydro-1*H*-inden-1-one (S34)

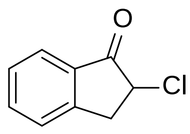

According to a modified procedure[13], indanone (0.66 g, 5.0 mmol, 1.0 eq.), Oxone (3.7 g, 6.0 mmol, 1.2 eq.) and NH_4Cl (0.54 g, 10 mmol, 2.0 eq.) were dissolved in MeOH (20 mL) and the reaction mixture was stirred for 16 h. The white suspension was

filtered and the solvent was removed *in vacuo*. Column chromatography purification (*n*-pentane:EtOAc 20:1) afforded product **S34** as pale yellow solid (0.67 g, 4.0 mmol, 80%).

R$_f$ (*n*-pentane:EtOAc 10:1) = 0.37. **M.p.** 37 – 38 °C. **^{1}H NMR** (300 MHz, $CDCl_3$, 299 K) δ [ppm] = 7.88 – 7.79 (m, 1H), 7.67 (m, 1H), 7.49 – 7.38 (m, 2H), 4.57 (dd, J = 7.8, 4.1 Hz, 1H), 3.79 (dd, J = 17.6, 7.8 Hz, 1H), 3.30 (dd, J = 17.6, 4.1 Hz, 1H). **GC-MS** (EI) *m/z* [M]$^+$, calcd. for $C_9H_7OCl^+$: 166.02, found: 166.09. Analytic data in agreement with the literature.[14]

2-Chloro-1-methylene-2,3-dihydro-1*H*-indene (S35)

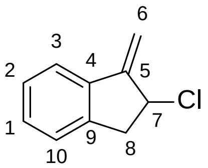

According to **general procedure C**, Ph_3PMeBr (1.7 g, 4.5 mmol, 1.5 eq.) and KO^{t}Bu (0.51 g, 4.5 mmol, 1.5 eq.) were dissolved in THF (7 mL). 2-Chloro-2,3-dihydro-1*H*-inden-1-one (**S34**) (0.50 g, 3.0 mmol, 1.0 eq.) was added and the reaction mixture was stirred for 16 h. Column chromatography purification (*n*-pentane) afforded product **S35** as colourless oil (74 mg, 0.45 mmol, 15%).

R$_f$ (*n*-pentane) = 0.58. **^{1}H NMR** (500 MHz, $CDCl_3$, 299 K) δ [ppm] = 7.50 (m, 1H, H1), 7.29 – 7.22 (m, 3H, H2, H3, H10), 5.68 (d, $^4J_{HH}$ = 1.8 Hz, 1H, H6^a), 5.43 (d, $^4J_{HH}$ = 1.5 Hz, 1H, H6^b), 5.05 (ddt, $^3J_{HH}$ = 7.3, 3.4 Hz, $^4J_{HH}$ = 1.7 Hz, 1H, H7), 3.54 (dd, $^2J_{HH}$ = 17.3 Hz, $^3J_{HH}$ = 7.3 Hz, 1H, H8^a), 3.23 (dd, $^2J_{HH}$ = 17.2 Hz, $^3J_{HH}$ = 3.4 Hz, 1H, H8^b). **^{13}C NMR** (126 MHz, $CDCl_3$, 299 K) δ [ppm] = 150.9 (1C, C9), 142.0 (1C, C5), 138.1 (1C, C4), 129.4 (1C, C3), 127.5 (1C, C2), 125.3 (1C, C10), 121.3 (1C, C1), 108.2 (1C, C6), 59.5 (1C, C7), 42.2 (1C, C8). **IR** (ATR) $\tilde{\nu}$ [cm^{-1}] = 2976 (w), 1645 (w), 1463 (w), 1426 (w), 1225 (w), 1141 (w), 953 (w), 908 (m), 889 (m), 776 (s), 732 (m), 716 (m). **GC-MS** (EI) *m/z* [M]$^+$, calcd. for $C_{10}H_9Cl^+$: 164.0387, found: 164.0387.

Dimethyl 2,2'-(2-iodo-1,3-phenylene)bis(oxy))(2*R*, 2'*R*)-dipropionate (S36)

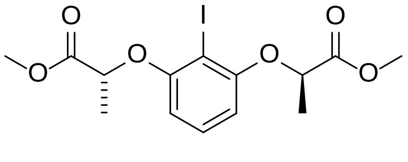

According to a modified procedure[15], 2-iodobenzene-1,3-diol (3.5 g, 15 mmol, 1.0 eq.) and PPh_3 (9.0 g, 35 mmol, 2.3 eq.) were dissolved in dry THF (30 mL). Methyl-(*S*)-(-)-lactate (3.2 mL, 33 mmol, 2.2 eq.) was added to the reaction mixture. The mixture was cooled to 0 °C and DIAD (7.3 g, 36 mmol, 2.4 eq.)

was added dropwise and the reaction mixture was stirred for 20 h at ambient temperature. The solvent was removed *in vacuo* and column chromatography purification (cyclohexane:EtOAc 5:1) afforded product **S36** as pale yellow oil (4.9 g, 12 mmol, 81%).

$[\alpha]_D^{25}$ = -18.8° (*c* = 1.00, $CHCl_3$). **R$_f$** (*n*-pentane:EtOAc 10:1) = 0.10. **^{1}H NMR** (400 MHz, $CDCl_3$, 299 K) *δ* [ppm] = 7.16 (t, *J* = 8.2 Hz, 1H), 6.39 (d, *J* = 8.3 Hz, 2H), 4.79 (q, *J* = 6.8 Hz, 2H), 3.77 (s, 6H), 1.72 (d, *J* = 6.8 Hz, 6H). **HRMS** (ESI) *m/z* $[M+Na]^+$, calcd. for $C_{14}H_{17}O_6INa^+$: 430.9962, found: 430.9967. Analytic data in agreement with the literature.[15]

(1*S*, 2*R*, 5*S*)-2-Isopropyl-5-methylcyclohexyl (*R*)-2-(2-iodo-3-(((*R*)-1-(((1*R*, 2*R*, 5*S*)-2-isopropyl-5-methylcyclohexyl)oxy)-1-oxopropan-2-yl)oxy)phenoxy)-propanoate (S37)

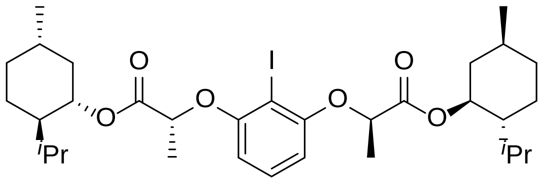

According to a modified procedure[15], dimethyl 2,2'-(2-iodo-1,3-phenylene)bis(oxy))(2*R*, 2'*R*)-dipropionate (**S36**) (4.1 g, 10 mmol, 1.0 eq.) was dissolved in THF (35 mL) and MeOH (35 mL). NaOH (2 M, 28 mL) was added and the reaction mixture was stirred for 18 h. HCl (1 M) was added and the aqueous solution was extracted with EtOAc (3x). The combined organic layers were washed with H_2O (1x) and brine (1x), dried over $MgSO_4$, filtered and the solvent was removed *in vacuo*. (2*R*, 2'*R*)-2,2'-((2-iodo-1,3-phenylene)bis(oxy))dipropionic acid was obtained and used without purification in the next step.

According to a modified procedure[16], (2*R*, 2'*R*)-2,2'-((2-iodo-1,3-phenylene)bis(oxy))di-propionic acid (3.3 g, 8.8 mmol, 1.0 eq.), *D*-menthol (3.4 g, 22 mmol, 2.5 eq.) and 4-(dimethylamino)-pyridine (0.76 mg, 6.2 mmol, 0.7 eq.) were dissolved in CH_2Cl_2 (70 mL). The reaction mixture was cooled to 0 °C and *N*,*N'*-dicyclohexylcarbodiimide (4.5 g, 22 mmol, 2.5 eq.) in CH_2Cl_2 (15 mL) was added. The reaction was slowly warmed to room temperature and was stirred for 16 h. The reaction was quenched with HCl (1 M) and the aqueous solution was extracted with CH_2Cl_2 (3x). The combined organic layers were dried over $MgSO_4$, filtered and the solvent was removed *in vacuo*. The crude reaction mixture was purified by column chromatography (*n*-pentane:EtOAc 50:1) to afford product **S37** as white solid (3.9 g, 5.9 mmol, 67% over two steps).

$[\alpha]_D^{25}$ = +11.9° (c = 1.00, $CHCl_3$). **R$_f$** (*n*-pentane:EtOAc 10:1) = 0.49. **M.p.** 99 – 100 °C. **^{1}H NMR** (400 MHz, $CDCl_3$, 299 K) δ [ppm] = 7.08 (t, J = 8.3 Hz, 1H), 6.35 (d, J = 8.2 Hz, 2H), 4.78 – 4.66 (m, 4H), 1.96 – 1.81 (m, 4H), 1.73 – 1.62 (m, 10H), 1.51 – 1.35 (m, 4H), 1.12 – 0.96 (m, 2H), 0.90 (d, J = 7.0 Hz, 6H), 0.87 (d, J = 4.9 Hz, 10H), 0.75 (d, J = 7.0 Hz, 6H). **HRMS** (ESI) *m/z* $[M+Na]^+$, calcd. for $C_{32}H_{49}O_6INa^+$: 679.2466, found: 679.2456. Analytic data in agreement with the literature.[17]

Methyl 1-oxo-2,3-dihydro-1*H*-indene-2-carboxylate (S38)

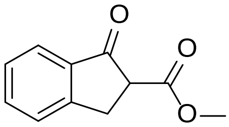

According to a modified procedure[18], a Schlenk tube was charged with NaH (60% w/w in mineral oil, 1.6 g, 40 mmol, 2.0 eq.), dry THF (50 mL) and dimethyl carbonate (8.5 mL, 100 mmol, 5.0 eq.). To the stirred suspension, indanone (2.6 g, 20 mmol, 1.0 eq.) in THF (20 mL) was slowly added and the reaction mixture was stirred for 16 h. The reaction was quenched with HCl (1 M) and the aqueous layer was extracted with Et_2O (3x). The combined organic layers were washed with brine (1x), dried over $MgSO_4$, filtered and the solvent was removed *in vacuo*. Column chromatography purification (*n*-pentane:EtOAc 10:1) afforded product **S38** as a mixture of keto and enol tautomer (ratio 85:15 by ^{1}H NMR) as pale yellow solid (3.2 g, 17 mmol, 83%).

R$_f$ (*n*-pentane:EtOAc 10:1) = 0.23. **M.p.** 57 – 59 °C. **^{1}H NMR** (400 MHz, $CDCl_3$, 299 K) δ [ppm] = 7.78 (d, J = 7.7 Hz, 1H), 7.67 – 7.59 (m, 1H), 7.51 (dt, J = 7.8, 0.9 Hz, 1H), 7.44 – 7.36 (m, 1H), 3.80 (s, 3H), 3.74 (dd, J = 8.3, 4.1 Hz, 1H), 3.57 (dd, J = 17.3, 4.2 Hz, 1H), 3.38 (dd, J = 17.3, 8.2 Hz, 1H). Minor peaks due to enol form observed at 10.37 (b, 1H), 3.86 (s, 3H), 3.52 (s, 2H). **HRMS** (ESI) *m/z* $[M+Na]^+$, calcd. for $C_{11}H_{10}O_3Na^+$: 213.0522, found: 213.0519. Analytic data in agreement with the literature.[19]

Methyl (*R*)-2-fluoro-1-oxo-2,3-dihydro-1*H*-indene-2-carboxylate (S39)

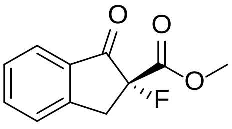

According to a modified procedure,[17] methyl 1-oxo-2,3-dihydro-1*H*-indene-2-carboxylate (**S38**) (0.95 g, 5.0 mmol, 1.0 eq.) and catalyst **S37** (0.33 g, 0.50 mmol, 10 mol%) were dissolved in $CHCl_3$ (50 mL) in a Teflon® screw cap vial. NEt_3:HF 1:5 (5 mL) and *m*-CPBA (1.3 g, 7.5 mmol, 1.5 eq.) were added and the reaction mixture was stirred for 18 h. The reaction mixture was poured into a saturated solution of $NaHCO_3$ and the aqueous layer was

extracted with CH_2Cl_2 (3x). The combined organic layers were dried over $MgSO_4$, filtered and the solvent was removed *in vacuo*. The crude reaction mixture was purified by column chromatography (*n*-pentane:EtOAc 10:1) to afford product **S39** as pale yellow solid (0.75 g, 3.6 mmol, 72%, 73:27 e.r.).
CAUTION: For safety reasons the reaction was prepared in five batches of a 1.0 mmol scale and were combined after quenching.
The e.r. of the product was determined by HPLC analysis using a Reporsil Chiral-OM column (10 μm, 250 mm x 4.6 mm) as the stationary phase and hexane:^{i}PrOH (95:5) as eluent system at a flow rate of 1.0 mL/min. Detection took place at λ = 254 nm with t_R(major) = 13.59 min; t_R(minor) = 15.89 min. $[\alpha]_D^{25}$ = -14.0° (*c* = 1.00, $CHCl_3$). **R$_f$** (*n*-pentane:EtOAc 10:1) = 0.10. **M.p.** 83 – 85 °C. **^{1}H NMR** (400 MHz, $CDCl_3$, 299 K) δ [ppm] = 7.85 (d, *J* = 7.7 Hz, 1H), 7.71 (td, *J* = 7.5, 1.3 Hz, 1H), 7.58 – 7.42 (m, 2H), 3.87 – 3.74 (m, 4H), 3.44 (dd, *J* = 23.3, 17.6 Hz, 1H). **^{19}F NMR** (377 MHz, $CDCl_3$, 299 K) δ [ppm] = -164.5 (dd, *J* = 23.3, 11.2 Hz, 1F). **^{19}F{^{1}H} NMR** (377 MHz, $CDCl_3$, 299 K) δ [ppm] = -164.5 (s, 1F). **HRMS** (ESI) *m/z* $[M+Na]^+$, calcd. for $C_{11}H_9O_3FNa^+$: 231.0428, found: 231.0426. Analytic data in agreement with the literature.[20]

Methyl (*R*)-2-fluoro-1-methylene-2,3-dihydro-1*H*-indene-2-carboxylate (S40)

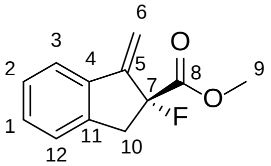

According to **general procedure C**, Ph_3PMeBr (1.4 g, 3.8 mmol, 1.5 eq.) and KO^{t}Bu (0.43 g, 3.8 mmol, 1.5 eq.) were dissolved in THF (15 mL). Methyl (*R*)-2-fluoro-1-oxo-2,3-dihydro-1*H*-indene-2-carboxylate (**S39**) (0.52 g, 2.5 mmol, 1.0 eq.) was added and the reaction mixture was stirred for 16 h. Column chromatography purification (*n*-pentane:EtOAc 40:1) afforded product **S40** as white solid (0.25 g, 1.2 mmol, 49%, 74:26 e.r.).
The e.r. of the product was determined by HPLC analysis using a Reporsil Chiral-OM column (10 μm, 250 mm x 4.6 mm) as the stationary phase and hexane:^{i}PrOH (98:2) as eluent system at a flow rate of 1.0 mL/min. Detection took place at λ = 254 nm with t_R(major) = 7.59 min; t_R(minor) = 8.60 min. $[\alpha]_D^{25}$ = -32.9° (*c* = 1.00, $CHCl_3$). **R$_f$** (*n*-pentane:EtOAc 10:1) = 0.34. **M.p.** 45 – 46 °C. **^{1}H NMR** (500 MHz, $CDCl_3$, 299 K) δ [ppm] = 7.55 – 7.44 (m, 1H, H1), 7.34 – 7.26 (m, 3H, H2, H3, H12), 5.80 (dd, $^4J_{FH}$ = 5.0 Hz, $^2J_{HH}$ = 0.9 Hz, 1H, H6^a), 5.49 (dd, $^4J_{FH}$ = 4.7 Hz, $^2J_{HH}$ = 0.9 Hz, 1H, H6^b), 3.81 (s, 3H, H9),

3.75 (dd, $^3J_{FH}$ = 17.8 Hz, $^2J_{HH}$ = 17.5 Hz, 1H, H10^a), 3.38 (dd, $^3J_{FH}$ = 27.0 Hz, $^2J_{HH}$ = 17.5 Hz, 1H, H10^b). **^{13}C NMR** (126 MHz, $CDCl_3$, 299 K) δ [ppm] = 170.7 (d, $^2J_{FC}$ = 31.2 Hz, 1C, C8), 148.5 (d, $^2J_{FC}$ = 17.4 Hz, 1C, C5), 141.1 (d, $^3J_{FC}$ = 1.7 Hz, 1C, C11), 137.3 (d, $^3J_{FC}$ = 2.2 Hz, 1C, C4), 129.9 (1C, C3), 127.7 (d, $^5J_{FC}$ = 0.8 Hz, 1C, C2), 125.3 (d, $^4J_{FC}$ = 1.6 Hz, 1C, C12), 121.3 (1C, C1), 109.5 (d, $^3J_{FC}$ = 6.3 Hz, 1C, C6), 98.5 (d, $^1J_{FC}$ = 194.9 Hz, 1C, C7), 53.0 (d, $^4J_{FC}$ = 1.0 Hz, 1C, C9), 42.1 (d, $^2J_{FC}$ = 24.4 Hz, 1C, C10). **^{19}F NMR** (470 MHz, $CDCl_3$, 299 K) δ [ppm] = -143.8 (ddt, $^3J_{FH}$ = 27.3, 17.8 Hz, $^4J_{FH}$ = 4.8 Hz, 1F, F7). **^{19}F{^{1}H} NMR** (470 MHz, $CDCl_3$, 299 K) δ [ppm] = -143.8 (s, 1F, F7). **IR** (ATR) $\tilde{\nu}$ [cm^{-1}] = 2956 (w), 1754 (s), 1741 (s), 1644 (w), 1476 (w), 1437 (m), 1295 (m), 1270 (s), 1222 (m), 1193 (s), 1093 (m), 1061 (s), 942 (w), 894 (m), 811 (m), 777 (s), 694 (m). **HRMS** (ESI) *m/z* [M+Na]$^+$, calcd. for $C_{12}H_{11}O_2FNa^+$: 229.0635, found: 229.0635.

Ethyl 1-oxo-2,3-dihydro-1*H*-indene-2-carboxylate (S41)

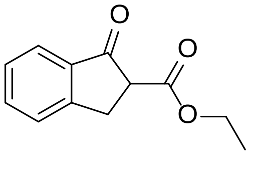

According to a modified procedure[21], a solution of indanone (2.6 g, 20 mmol, 1.0 eq.) in dry THF (20 mL) was added dropwise to a solution of diethyl carbonate (12 mL, 100 mmol, 5.0 eq.) and NaH (60% w/w in mineral oil, 1.6 g, 40 mmol, 2.0 eq.) in dry THF (50 mL). The reaction mixture was refluxed for 5 h and then cooled to 0 °C before the addition of HCl (1 M). The aqueous solution was extracted with Et_2O (3x) and the combined organic layers were washed with brine (1x), dried over $MgSO_4$, filtered and the solvent was removed. Column chromatography purification (*n*-pentane:EtOAc 10:1) afforded product **S41** as a mixture of keto and enol tautomer (ratio 85:15 by ^{1}H NMR) as yellow oil (3.6 g, 17 mmol, 89%).

R_f (*n*-pentane:EtOAc 10:1) = 0.26. **^{1}H NMR** (400 MHz, $CDCl_3$, 299 K) δ [ppm] = 7.82 – 7.72 (m, 1H), 7.66 – 7.60 (m, 1H), 7.51 (dt, *J* = 7.7, 1.0 Hz, 1H), 7.43 – 7.35 (m, 1H), 4.25 (q, *J* = 7.2, 2H), 3.72 (dd, *J* = 8.3, 4.1 Hz, 1H), 3.56 (dd, *J* = 17.3, 4.1 Hz, 1H), 3.37 (dd, *J* = 17.2, 8.3 Hz, 1H), 1.31 (t, *J* = 7.1 Hz, 3H). Minor peaks due to enol form observed at 10.45 (b, 1H), 3.52 (s, 2H), 1.37 (t, *J* = 7.1 Hz, 3H). **HRMS** (ESI) *m/z* [M+Na]$^+$, calcd. for $C_{12}H_{12}O_3Na^+$: 227.0679, found: 227.0678. Analytic data in agreement with the literature.[19]

Ethyl (*R*)-2-fluoro 1-oxo-2,3-dihydro-1*H*-indene-2-carboxylate (S42)

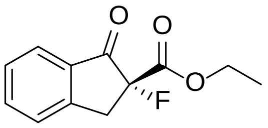

According to a modified procedure,[17] ethyl 1-oxo-2,3-dihydro-1*H*-indene-2-carboxylate (**S41**) (0.82 g, 4.0 mmol, 1.0 eq.) and catalyst **S37** (0.26 g, 0.40 mmol, 10 mol%) were dissolved in $CHCl_3$ (40 mL) in a Teflon® screw cap vial. NEt_3:HF 1:5 (4 mL) and *m*-CPBA (1.0 g, 6.0 mmol, 1.5 eq.) were added and the reaction mixture was stirred for 18 h. The reaction mixture was poured into a saturated solution of $NaHCO_3$ and the aqueous layer was extracted with CH_2Cl_2 (3x). The combined organic layers were dried over $MgSO_4$, filtered and the solvent was removed *in vacuo*. The crude reaction mixture was purified by column chromatography (*n*-pentane:EtOAc 15:1) to afford product **S42** as pale yellow oil (0.71 g, 3.2 mmol, 80%, 84:16 e.r.). CAUTION: For safety reasons the reaction was prepared in four batches of a 1.0 mmol scale and were combined after quenching.

The e.r. of the product was determined by HPLC analysis using a Reporsil Chiral-OM column (10 μm, 250 mm x 4.6 mm) as the stationary phase and hexane:^{i}PrOH (95:5) as eluent system at a flow rate of 1.0 mL/min. Detection took place at λ = 254 nm with t_R(major) = 10.44 min; t_R(minor) = 12.00 min. $[\alpha]_D^{25}$ = -12.0° (*c* = 1.00, $CHCl_3$). **R_f** (*n*-pentane:EtOAc 10:1) = 0.11. **^{1}H NMR** (400 MHz, $CDCl_3$, 299 K) δ [ppm] = 7.84 (d, *J* = 7.7 Hz, 1H), 7.70 (td, *J* = 7.5, 1.3 Hz, 1H), 7.54 – 7.41 (m, 2H), 4.28 (q, *J* = 7.1 Hz, 2H), 3.79 (dd, *J* = 17.6, 11.5 Hz, 1H), 3.44 (dd, *J* = 23.3, 17.6 Hz, 1H), 1.26 (t, *J* = 7.1 Hz, 3H). **^{19}F NMR** (376 MHz, $CDCl_3$, 299 K) δ [ppm] = -164.5 (dd, *J* = 23.3, 11.5 Hz, 1F). **^{19}F{^{1}H} NMR** (470 MHz, $CDCl_3$, 299 K) δ [ppm] = -164.5 (s, 1F). **HRMS** (ESI) *m/z* $[M+Na]^+$, calcd. for $C_{12}H_{11}O_3FNa^+$: 245.0584, found: 245.0581. Analytic data in agreement with the literature.[17]

Ethyl (*R*)-2-fluoro 1-methylene-2,3-dihydro-1*H*-indene-2-carboxylate (S43)

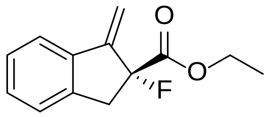

According to **general procedure C**, Ph_3PMeBr (1.7 g, 4.5 mmol, 1.5 eq.) and KO^{t}Bu (0.51 g, 4.5 mmol, 1.5 eq.) were dissolved in THF (6 mL). Ethyl (*R*)-2-fluoro 1-oxo-2,3-dihydro-1*H*-indene-2-carboxylate (**S42**) (0.67 g, 3.0 mmol, 1.0 eq.) was added and the reaction mixture was stirred for 16 h. Column chromatography purification (*n*-pentane:EtOAc 40:1) afforded product **S43** as yellow oil (0.43 g, 2.0 mmol, 65%, 84:16 e.r.).

The e.r. of the product was determined by HPLC analysis using a Reporsil Chiral-OM column (10 *μ*m, 250 mm x 4.6 mm) as the stationary phase and hexane:^{i}PrOH (98:2) as eluent system at a flow rate of 1.0 mL/min. Detection took place at *λ* = 254 nm with t_R(major) = 6.52 min; t_R(minor) = 7.31 min. $[\alpha]_D^{25}$ **=** -36.8° (*c* = 1.00, $CHCl_3$). **R$_f$** (*n*-pentane:EtOAc 10:1) = 0.51. **^{1}H NMR** (400 MHz, $CDCl_3$, 299 K) *δ* [ppm] = 7.54 – 7.48 (m, 1H), 7.36 – 7.25 (m, 3H), 5.80 (d, *J* = 5.0 Hz, 1H), 5.49 (d, *J* = 4.8 Hz, 1H), 4.38 – 4.20 (m, 2H), 3.76 (dd, *J* = 18.1, 17.5 Hz, 1H), 3.37 (dd, *J* = 27.1, 17.6 Hz, 1H), 1.29 (t, *J* = 7.1 Hz, 3H). **^{19}F NMR** (470 MHz, $CDCl_3$, 299 K) *δ* [ppm] = -143.4 (ddt, *J* = 27.0, 18.7, 4.9 Hz, 1F). **^{19}F{^{1}H} NMR** (470 MHz, $CDCl_3$, 299 K) *δ* [ppm] = -143.4 (s, 1F). **HRMS** (ESI) *m/z* $[M+Na]^+$, calcd. for $C_{13}H_{13}O_2FNa^+$: 243.0792, found: 243.0791. Analytic data in agreement with the literature.[17]

Ethyl 5-bromo-1-oxo-2,3-dihydro-1*H*-indene-2-carboxylate (S44)

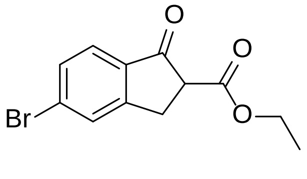

According to a modified procedure[21], a solution of 5-bromo-indanone (4.2 g, 20 mmol, 1.0 eq.) in dry THF (20 mL) was added dropwise to a solution of diethyl carbonate (12 mL, 100 mmol, 5.0 eq.) and NaH (60% w/w in mineral oil, 1.6 g, 40 mmol, 2.0 eq.) in dry THF (50 mL). The reaction mixture was stirred for 18 h and then cooled to 0 °C before the addition of HCl (1 M). The aqueous solution was extracted with Et_2O (3x) and the combined organic layers were washed with brine (1x), dried over $MgSO_4$, filtered and the solvent was removed. Column chromatography purification (*n*-pentane:EtOAc 10:1) afforded product **S44** as a mixture of keto and enol tautomer (ratio 75:25 by ^{1}H NMR) as pale yellow solid (4.0 g, 14 mmol, 71%).
R$_f$ (*n*-pentane:EtOAc 10:1) = 0.34. **M.p.** 49 – 50 °C. **^{1}H NMR** (400 MHz, $CDCl_3$, 299 K) *δ* [ppm] = 7.69 (d, *J* = 1.5 Hz, 1H), 7.62 (s, 1H), 7.59 – 7.46 (m, 1H), 4.25 (q, *J* = 7.5 Hz, 2H), 3.71 (dd, *J* = 8.3, 4.0 Hz, 1H), 3.55 (dd, *J* = 17.5, 4.1 Hz, 1H), 3.35 (dd, *J* = 17.5, 8.2 Hz, 1H), 1.31 (t, *J* = 7.1 Hz, 3H). Minor peaks due to enol form observed at 10.39 (s, 1H), 7.64 (s, 1H), 4.32 (q, *J* = 7.1 Hz, 2H), 3.50 (s, 2H), 1.37 (t, *J* = 7.1 Hz, 3H). **HRMS** (ESI) *m/z* $[M+Na]^+$, calcd. for $C_{12}H_{11}O_3BrNa^+$: 304.9784, found: 304.9782. Analytic data in agreement with the literature.[17]

Ethyl (*R*)-5-bromo-2-fluoro 1-oxo-2,3-dihydro-1*H*-indene-2-carboxylate (S45)

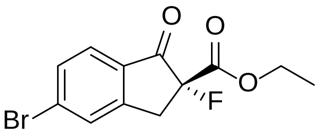

According to a modified procedure,[17] ethyl 5-bromo-1-oxo-2,3-dihydro-1*H*-indene-2-carboxylate (**S44**) (1.4 g, 5.0 mmol, 1.0 eq.) and catalyst **S37** (0.33 g, 0.50 mmol, 10 mol%) were dissolved in $CHCl_3$ (50 mL) in a Teflon® screw cap vial. NEt_3:HF 1:5 (5 mL) and *m*-CPBA (1.3 g, 7.5 mmol, 1.5 eq.) were added and the reaction mixture was stirred for 18 h. The reaction mixture was poured into a saturated solution of $NaHCO_3$ and the aqueous layer was extracted with CH_2Cl_2 (3x). The combined organic layers were dried over $MgSO_4$, filtered and the solvent was removed *in vacuo*. The crude reaction mixture was purified by column chromatography (*n*-pentane:EtOAc 20:1) to afford product **S45** as pale yellow solid (1.1 g, 3.5 mmol, 70%, 86:14 e.r.).

CAUTION: For safety reasons the reaction was prepared in five batches of a 1.0 mmol scale and were combined after quenching.

The e.r. of the product was determined by HPLC analysis using a Reporsil Chiral-OM column (10 μm, 250 mm x 4.6 mm) as the stationary phase and hexane:^{i}PrOH (95:5) as eluent system at a flow rate of 1.0 mL/min. Detection took place at λ = 253 nm with t_R(major) = 11.86 min; t_R(minor) = 14.00 min. $[\alpha]_D^{25}$ = -43.3° (c = 1.00, $CHCl_3$). $\mathbf{R_f}$ (*n*-pentane:EtOAc 10:1) = 0.39. **M.p.** 72 – 75 °C. **^{1}H NMR** (400 MHz, $CDCl_3$, 299 K) δ [ppm] = 7.73 – 7.68 (m, 2H), 7.65 – 7.59 (m, 1H), 4.29 (q, J = 7.1 Hz, 2H), 3.84 – 3.71 (m, 1H), 3.55 – 3.32 (m, 1H), 1.27 (t, J = 7.1 Hz, 3H). **^{19}F NMR** (376 MHz, $CDCl_3$, 299 K) δ [ppm] = -164.0 (dd, J = 22.9, 11.2 Hz, 1F). **^{19}F{^{1}H} NMR** (376 MHz, $CDCl_3$, 299 K) δ [ppm] = -164.0 (s, 1F). **HRMS** (ESI) *m/z* $[M+Na]^+$, calcd. for $C_{12}H_{10}O_3BrFNa^+$: 322.9690, found: 322.9689. Analytic data in agreement with the literature.[17]

Ethyl (*R*)-5-bromo-2-fluoro 1-methylene-2,3-dihydro-1*H*-indene-2-carboxylate (S46)

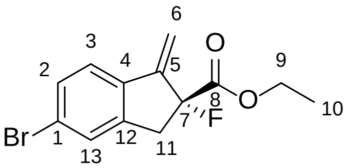

According to **general procedure C**, Ph_3PMeBr (1.4 g, 3.8 mmol, 1.5 eq.) and KO^{t}Bu (0.42 g, 3.8 mmol, 1.5 eq.) were dissolved in THF (15 mL). Ethyl (*R*)-5-bromo-2-fluoro 1-oxo-2,3-dihydro-1*H*-indene-2-carboxylate (**S45**) (0.75 g, 2.5 mmol, 1.0 eq.) was added and the reaction mixture was stirred for 16 h. Column

chromatography purification (*n*-pentane:EtOAc 20:1) afforded product **S46** as white solid (0.59 g, 2.0 mmol, 78%, 85:15 e.r.).

The e.r. of the product was determined by HPLC analysis using a Reporsil Chiral-OM column (10 μm, 250 mm x 4.6 mm) as the stationary phase and hexane:^{i}PrOH (99.5:0.5) as eluent system at a flow rate of 1.0 mL/min. Detection took place at λ = 254 nm with t_R(major) = 10.18 min; t_R(minor) = 10.94 min. $[\alpha]_D^{25}$ = -47.7° (*c* = 1.00, $CHCl_3$). **R_f** (*n*-pentane:EtOAc 10:1) = 0.68. **M.p.** 50 – 52 °C. **^{1}H NMR** (599 MHz, $CDCl_3$, 299 K) δ [ppm] = 7.46 – 7.42 (m, 1H, H13), 7.43 – 7.38 (m, 1H, H2), 7.36 (d, *J* = 8.2 Hz, 1H, H3), 5.80 (dd, $^4J_{FH}$ = 4.9 Hz, $^2J_{HH}$ = 1.1 Hz, 1H, H6^a), 5.53 (dd, $^4J_{FH}$ = 4.6 Hz, $^2J_{HH}$ = 1.0 Hz, 1H, H6^b), 4.34 – 4.23 (m, 2H, H9), 3.73 (dd, $^3J_{FH}$ = 17.9 Hz, $^2J_{HH}$ = 17.7 Hz 1H, H11^a), 3.34 (dd, $^3J_{FH}$ = 26.6 Hz, $^2J_{HH}$ = 17.7 Hz, 1H, H11^b), 1.29 (t, *J* = 7.1 Hz, 3H, H10). **^{13}C NMR** (151 MHz, $CDCl_3$, 299 K) δ [ppm] = 169.9 (d, $^2J_{FC}$ = 30.6 Hz, 1C, C8), 147.6 (d, $^2J_{FC}$ = 17.6 Hz, 1C, C5), 143.2 (d, $^3J_{FC}$ = 1.6 Hz, 1C, C12), 136.4 (d, $^3J_{FC}$ = 2.3 Hz, 1C, C4), 131.0 (1C, C2), 128.5 (d, $^4J_{FC}$ = 1.7 Hz, 1C, C13), 123.8 (1C, C1), 122.7 (1C, C3), 110.2 (d, $^3J_{FC}$ = 6.2 Hz, 1C, C6), 98.2 (d, $^1J_{FC}$ = 196.0 Hz, 1C, C7), 62.3 (1C, C9), 41.7 (d, $^2J_{FC}$ = 24.8 Hz, 1C, C11), 14.2 (1C, C10). **^{19}F NMR** (564 MHz, $CDCl_3$, 299 K) δ [ppm] = -143.5 (ddt, $^3J_{FH}$ = 27.0, 17.9 Hz, $^4J_{FH}$ = 4.8 Hz, 1F, F7). **^{19}F{^{1}H} NMR** (470 MHz, $CDCl_3$, 299 K) δ [ppm] = -143.5 (1F, F7). **IR** (ATR) $\tilde{\nu}$ [cm^{-1}] = 2970 (w), 1753 (s), 1595 (w), 1471 (w), 1407 (w), 1299 (m), 1279 (m), 1261 (m), 1222 (m), 1188 (s), 1134 (w), 1056 (s), 1023 (m), 913 (m), 868 (m), 859 (s), 753 (m), 713 (w). **GC-MS** (EI) *m/z* $[M]^+$, calcd. for $C_{13}H_{12}O_2BrF^+$: 298.0000, found: 298.0000.

Ethyl 5-chloro-1-oxo-2,3-dihydro-1*H*-indene-2-carboxylate (S47)

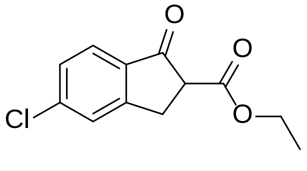

According to a modified procedure[21], a solution of 5-chloro-indanone (3.3 g, 20 mmol, 1.0 eq.) in dry THF (20 mL) was added dropwise to a solution of diethyl carbonate (12 mL, 100 mmol, 5.0 eq.) and NaH (60% w/w in mineral oil, 1.6 g, 40 mmol, 2.0 eq.) in dry THF (50 mL). The reaction mixture was stirred for 18 h and was then cooled to 0 °C before the addition of HCl (1 M). The aqueous solution was extracted with Et_2O (3x) and the combined organic layers were washed with brine (1x), dried over $MgSO_4$, filtered and the solvent

was removed. Column chromatography purification (*n*-pentane:EtOAc 10:1) afforded product **S47** as a mixture of keto and enol tautomer (ratio 77:23 by ^{1}H NMR) as yellow solid (2.8 g, 12 mmol, 58%).

R$_f$ (*n*-pentane:EtOAc 10:1) = 0.58. **M.p.** 54 – 55 °C. **^{1}H NMR** (400 MHz, $CDCl_3$, 299 K) δ [ppm] = 7.70 (d, *J* = 8.2 Hz, 1H), 7.50 (m, 1H), 7.40 – 7.33 (m, 1H), 4.28 – 4.22 (m, 2H), 3.73 (dd, *J* = 8.3, 4.0 Hz, 1H), 3.54 (dd, *J* = 17.6, 4.2 Hz, 1H), 3.35 (dd, *J* = 17.5, 8.2 Hz, 1H), 1.31 (t, *J* = 7.1 Hz, 3H). Minor peaks due to enol form observed at 10.40 (s, 1H), 7.55 (dd, *J* = 8.2, 0.6 Hz, 1H), 7.45 (d, *J* = 0.7 Hz, 1H), 4.32 (q, *J* = 7.1 Hz, 2H), 3.51 (s, 1H). **HRMS** (ESI) *m/z* $[M+Na]^+$, calcd. for $C_{12}H_{11}O_3ClNa^+$: 261.0289, found: 261.0287. Analytic data in agreement with the literature.[17]

Ethyl (*R*)-5-chloro-2-fluoro 1-oxo-2,3-dihydro-1*H*-indene-2-carboxylate (S48)

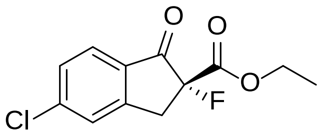

According to a modified procedure,[17] ethyl 5-chloro1-oxo-2,3-dihydro-1*H*-indene-2-carboxylate (**S47**) (0.96 g, 4.0 mmol, 1.0 eq.) and catalyst **S37** (0.26 g, 0.40 mmol, 10 mol%) were dissolved in $CHCl_3$ (40 mL) in a Teflon® screw cap vial. NEt_3:HF 1:5 (4 mL) and *m*-CPBA (1.0 g, 6.0 mmol, 1.5 eq.) were added and the reaction mixture was stirred for 18 h. The reaction mixture was poured into a saturated solution of $NaHCO_3$ and the aqueous layer was extracted with CH_2Cl_2 (3x). The combined organic layers were dried over $MgSO_4$, filtered and the solvent was removed *in vacuo*. The crude reaction mixture was purified by column chromatography (*n*-pentane:EtOAc 20:1) to afford product **S48** as pale yellow solid (0.90 g, 3.5 mmol, 88%, 87:13 e.r.).

CAUTION: For safety reasons the reaction was prepared in four batches of a 1.0 mmol scale and were combined after quenching.

The e.r. of the product was determined by HPLC analysis using a Reporsil Chiral-OM column (10 μm, 250 mm x 4.6 mm) as the stationary phase and hexane:^{i}PrOH (95:5) as eluent system at a flow rate of 1.0 mL/min. Detection took place at λ = 254 nm with t_R(major) = 11.31 min; t_R(minor) = 13.01 min. $[\alpha]_D^{25}$ = -38.9° (*c* = 1.00, $CHCl_3$). **R$_f$** (*n*-pentane:EtOAc 10:1) = 0.35. **M.p.** 54 – 55 °C. **^{1}H NMR** (400 MHz, $CDCl_3$, 299 K) δ [ppm] = 7.78 (d, *J* = 8.2 Hz, 1H), 7.51 (d, *J* = 1.0 Hz, 1H), 7.49 – 7.40 (m, 1H), 4.29 (q, *J* = 7.1 Hz, 2H), 3.77 (dd, *J* = 17.8, 11.2 Hz, 1H), 3.41 (dd, *J* = 22.8, 17.8 Hz, 1H), 1.27 (t, *J* = 7.1 Hz,

3H). **^{19}F NMR** (377 MHz, $CDCl_3$, 299 K) *δ* [ppm] = -163.9 (dd, *J* = 22.9, 11.3 Hz, 1F). **^{19}F{^{1}H} NMR** (377 MHz, $CDCl_3$, 299 K) *δ* [ppm] = -163.9 (s, 1F). **HRMS** (ESI) *m/z* $[M+Na]^+$, calcd. for $C_{12}H_{10}O_3ClFNa^+$: 279.0195, found: 279.0193. Analytic data in agreement with the literature.[17]

Ethyl (*R*)-5-chloro-2-fluoro 1-methylene-2,3-dihydro-1*H*-indene-2-carboxylate (S49)

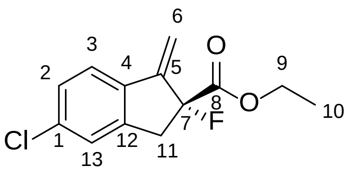

According to **general procedure C**, Ph_3PMeBr (1.4 g, 3.8 mmol, 1.5 eq.) and KO^{t}Bu (0.43 g, 3.8 mmol, 1.5 eq.) were dissolved in THF (15 mL). Ethyl (*R*)-5-chloro-2-fluoro 1-oxo-2,3-dihydro-1*H*-indene-2-carboxylate (**S48**) (0.64 g, 2.5 mmol, 1.0 eq.) was added and the reaction mixture was stirred for 16 h. Column chromatography purification (*n*-pentane:EtOAc 30:1) afforded product **S49** as colourless oil (0.47 mg, 1.8 mmol, 73%, 86:14 e.r.).

The e.r. of the product was determined by HPLC analysis using a Reporsil Chiral-AM column (10 *µ*m, 250 mm x 4.6 mm) as the stationary phase and hexane:^{i}PrOH (99.8:0.2) as eluent system at a flow rate of 1.0 mL/min. Detection took place at *λ* = 254 nm with t_R(minor) = 8.76 min; t_R(major) = 10.26 min. $[\alpha]_D^{25}$ = -52.7° (*c* = 1.00, $CHCl_3$). **R_f** (*n*-pentane:EtOAc 10:1) = 0.65. **^{1}H NMR** (599 MHz, $CDCl_3$, 299 K) *δ* [ppm] = 7.42 (d, *J* = 8.2 Hz, 1H, H2), 7.29 – 7.25 (m, 1H, H13), 7.28 – 7.23 (m, 1H, H3), 5.78 (dd, $^4J_{FH}$ = 5.0 Hz, $^2J_{HH}$ = 1.1 Hz, 1H, H6^a), 5.52 (dd, $^4J_{FH}$ = 4.6 Hz, $^2J_{HH}$ = 1.1 Hz, 1H, H6^b), 4.40 – 4.16 (m, 2H, H9), 3.73 (dd, $^3J_{FH}$ = 17.9 Hz, $^2J_{HH}$ = 17.7 Hz, 1H, H11^a), 3.34 (dd, $^3J_{FH}$ = 26.5 Hz, $^2J_{HH}$ = 17.7 Hz, 1H, H11^b), 1.28 (d, *J* = 7.1 Hz, 3H, H10). **^{13}C NMR** (151 MHz, $CDCl_3$, 299 K) *δ* [ppm] = 169.9 (d, $^2J_{FC}$ = 30.6 Hz, 1C, C8), 147.5 (d, $^2J_{FC}$ = 17.5 Hz, 1C, C5), 142.9 (d, $^3J_{FC}$ = 1.7 Hz, 1C, C12), 136.0 (d, $^3J_{FC}$ = 2.2 Hz, 1C, C4), 135.6 (1C, C1), 128.2 (1C, C2), 125.5 (d, $^4J_{FC}$ = 1.7 Hz, 1C, C13), 122.4 (1C, C3), 110.0 (d, $^3J_{FC}$ = 6.2 Hz, 1C, C6), 98.3 (d, $^1J_{FC}$ = 196.0 Hz, 1C, C7), 62.3 (1C, C9), 41.8 (d, $^2J_{FC}$ = 24.7 Hz, 1C, C11), 14.2 (1C, C10). **^{19}F NMR** (564MHz, $CDCl_3$, 299 K) *δ* [ppm] = -143.5 (ddt, $^3J_{FH}$ = 27.0, 17.8 Hz, $^4J_{FH}$ = 4.8 Hz, 1F, F6). **^{19}F{^{1}H} NMR** (470 MHz, $CDCl_3$, 299 K) *δ* [ppm] = -143.5 (s, 1F, F6). **IR** (ATR) $\tilde{\nu}$ [cm^{-1}] = 2983 (w), 1752 (s), 1737 (s), 1599 (w), 1472 (m), 1326 (w), 1301 (m), 1279 (m), 1218 (m), 1107 (m), 885 (m), 862 (m), 823 (m), 713 (w). **GC-MS** (EI) *m/z* $[M]^+$, calcd. for $C_{13}H_{12}O_2ClF^+$: 254.0504, found: 254.0503.

Ethyl 1-oxo-6-(trifluoromethyl)-2,3-dihydro-1*H*-indene-2-carboxylate (S50)

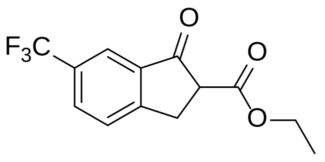

According to a modified procedure[21], a solution of 6-trifluoromethyl-indanone (2.0 g, 10 mmol, 1.0 eq.) in dry THF (10 mL) was added dropwise to a solution of diethyl carbonate (6.1 mL, 50 mmol, 5.0 eq.) and NaH (60% w/w in mineral oil, 0.80 g, 20 mmol, 2.0 eq.) in dry THF (25 mL). The reaction mixture was stirred for 18 h and was then cooled to 0 °C before the addition of HCl (1 M). The aqueous solution was extracted with Et_2O (3x) and the combined organic layers were washed with brine (1x), dried over $MgSO_4$, filtered and the solvent was removed. Column chromatography purification (*n*-pentane:EtOAc 10:1) afforded product **S50** as a mixture of keto and enol tautomer (ratio 35:65 by ^{1}H NMR) as brown solid (1.0 g, 3.8 mmol, 38%).

R_f (*n*-pentane:EtOAc 10:1) = 0.48. **M.p.** 64 – 65 °C. **^{1}H NMR** (400 MHz, $CDCl_3$, 299 K) δ [ppm] = 8.02 (s, 1H), 7.86 (d, J = 9.8 Hz, 1H), 7.69 – 7.62 (m, 1H), 4.26 (q, J = 7.1 Hz, 2H), 3.78 (dd, J = 8.3, 4.0 Hz, 1H), 3.63 (dd, J = 17.8, 4.1 Hz, 1H), 3.43 (dd, J = 17.7, 8.3 Hz, 1H), 1.31 (t, J = 7.1 Hz, 3H). Major peaks due to enol form observed at 10.35 (b, 1H), 7.88 (s, 1H), 7.69 – 7.62 (m, 1H), 7.56 (d, J = 7.9 Hz, 1H), 4.33 (q, J = 7.1 Hz, 2H), 3.57 (s, 2H), 1.37 (t, J = 7.1 Hz, 3H). **^{19}F NMR** (377 MHz, $CDCl_3$, 299 K) δ [ppm] = -62.1 (s, 3F). Major peak due to enol observed at -62.5 (s, 3F). **^{19}F{^{1}H} NMR** (377 MHz, $CDCl_3$, 299 K) δ [ppm] = -62.1 (s, 3F). Major peak due to enol observed at -62.5 (s, 3F). **HRMS** (ESI) *m/z* $[M+Na]^+$, calcd. for $C_{13}H_{11}O_3F_3Na^+$: 295.0563, found: 295.0550. Analytic data in agreement with the literature.[17]

Ethyl (*R*)-2-fluoro 1-oxo-6-(trifluoromethyl)-2,3-dihydro-1*H*-indene-2-carboxylate (S51)

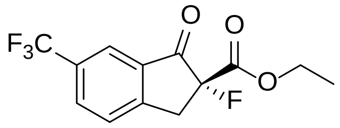

According to a modified procedure,[17] ethyl 1-oxo-6-(trifluoromethyl)-2,3-dihydro-1*H*-indene-2-carboxylate (**S50**) (0.82 g, 3.0 mmol, 1.0 eq.) and catalyst **S37** (0.20 g, 0.30 mmol, 10 mol%) were dissolved in $CHCl_3$ (30 mL) in a Teflon® screw cap vial. NEt_3:HF 1:5 (3 mL) and *m*-CPBA (0.78 g, 4.5 mmol, 1.5 eq.) were added and the reaction mixture was stirred for 18 h. The reaction mixture was poured into a saturated solution of $NaHCO_3$ and the aqueous layer was extracted with CH_2Cl_2 (3x). The combined organic layers were dried over $MgSO_4$, filtered and the solvent was removed *in vacuo*. The crude reaction mixture was purified by column chromatography (*n*-pentane:EtOAc 20:1) to afford product **S51** as pale yellow oil (0.66 g, 2.3 mmol, 76%, 87:13 e.r.).

CAUTION: For safety reasons the reaction was prepared in three batches of a 1.0 mmol scale and were combined after quenching.

The e.r. of the product was determined by HPLC analysis using a Reporsil Chiral-AM column (10 *µ*m, 250 mm x 4.6 mm) as the stationary phase and hexane:^{i}PrOH (98:2) as eluent system at a flow rate of 1.0 mL/min. Detection took place at $\lambda = 254$ nm with t_R(major) = 7.82 min; t_R(minor) = 9.58 min. $[\alpha]_D^{25}$ = -4.8° (*c* = 1.00, $CHCl_3$). **R_f** (*n*-pentane:EtOAc 10:1) = 0.19. **^{1}H NMR** (400 MHz, $CDCl_3$, 299 K) *δ* [ppm] = δ 8.10 (s, 1H), 7.98 – 7.91 (m, 1H), 7.67 (d, *J* = 8.0 Hz, 1H), 4.29 (q, *J* = 7.1 Hz, 2H), 3.86 (dd, *J* = 18.0, 11.2 Hz, 1H), 3.50 (dd, *J* = 22.9, 18.0, 1H), 1.27 (t, *J* = 7.1 Hz, 3H). **^{19}F NMR** (377 MHz, $CDCl_3$, 299 K) *δ* [ppm] = -62.8 (s, 3F), -164.1 (dd, *J* = 22.8, 11.3 Hz, 1F). **^{19}F{^{1}H} NMR** (377 MHz, $CDCl_3$, 299 K) *δ* [ppm] = -62.8 (s, 3F), -164.1 (s,1F). **HRMS** (ESI) *m/z* $[M+Na]^+$, calcd. for $C_{13}H_{10}O_3F_4Na^+$: 313.0458, found: 313.0456. Analytic data in agreement with the literature.[17]

Ethyl (*R*)-2-fluoro 1-methylene-6-(trifluoromethyl)-2,3-dihydro-1*H*-indene-2-carboxylate (S52)

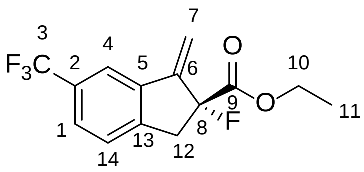

According to **general procedure C**, Ph_3PMeBr (1.0 g, 2.7 mmol, 1.5 eq.) and KO^{t}Bu (0.31 g, 2.7 mmol, 1.5 eq.) were dissolved in THF (10 mL). Ethyl (*R*)-2-fluoro 1-oxo-6-(trifluoromethyl)-2,3-dihydro-1*H*-indene-2-carboxylate (**S51**) (0.53 g, 1.8 mmol, 1.0 eq.) was added and the reaction mixture was stirred for 16 h. Column chromatography purification (*n*-pentane:EtOAc 20:1) afforded product **S52** as colourless oil (0.30 g, 1.0 mmol, 57%, 87:13 e.r.).

The e.r. of the product was determined by HPLC analysis using a Reporsil Chiral-OM column (10 *µ*m, 250 mm x 4.6 mm) as the stationary phase and hexane:^{i}PrOH (99.5:0.5) as eluent system at a flow rate of 0.5 mL/min. Detection took place at $\lambda = 254$ nm with t_R(minor) = 16.70 min; t_R(major) = 17.74 min. $[\alpha]_D^{25}$ = -21.2° (*c* = 1.00, $CHCl_3$). **R_f** (*n*-pentane:EtOAc 10:1) = 0.37. **^{1}H NMR** (599 MHz, $CDCl_3$, 299 K) *δ* [ppm] = 7.74 (s, 1H, H4), 7.56 (d, $^3J_{HH}$ = 8.8 Hz, 1H, H1), 7.40 (d, $^3J_{HH}$ = 8.9 Hz, 1H, H14), 5.91 (dd, $^4J_{FH}$ = 4.9 Hz, $^2J_{HH}$ = 1.2 Hz, 1H, H7^a), 5.62 (dd, $^4J_{FH}$ = 4.6 Hz, $^2J_{FH}$ = 1.2 Hz, 1H, H7^b), 4.36 – 4.24 (m, 2H, H10), 3.80 (dd, $^3J_{FH}$ = 18.0 Hz, $^2J_{HH}$ = 17.8 Hz, 1H, H12^a), 3.41 (dd, $^3J_{FH}$ = 26.4 Hz, $^2J_{HH}$ = 17.7 Hz, 1H, H12^b), 1.29 (t, *J* = 7.1 Hz, 3H, H11). **^{13}C NMR** (151 MHz, $CDCl_3$, 299 K) *δ* [ppm] = 169.7 (d,

$^2J_{FC}$ = 30.6 Hz, 1C, C9), 147.5 (d, $^2J_{FC}$ = 17.8 Hz, 1C, C6), 144.9 (m, 1C, C13), 138.0 (d, $^3J_{FC}$ = 2.3 Hz, 1C, C5), 130.4 (q, $^2J_{FC}$ = 31.5 Hz, 1C, C2), 126.6 (q, $^3J_{FC}$ = 3.7 Hz, 1C, C1), 125.8 (d, $^4J_{FC}$ = 1.5 Hz, 1C, C14), 124.2 (q, $^1J_{FC}$ = 272.2 Hz, 1C, C3), 118.4 (q, $^3J_{FC}$ = 4.0 Hz, 1C, C4), 111.5 (d, $^3J_{FC}$ = 6.1 Hz, 1C, C7), 98.2 (d, $^1J_{FC}$ = 196.3 Hz, 1C, C8), 62.4 (1C, C10), 42.0 (d, $^2J_{FC}$ = 24.8 Hz, 1C, C12), 14.2 (1C, C11). **^{19}F NMR** (564 MHz, $CDCl_3$, 299 K) δ [ppm] = -62.5 (s, 3F, F3), -143.6 (ddt, $^3J_{FH}$ = 27.0, 18.0 Hz, $^4J_{FH}$ = 4.7 Hz, 1F, F6). **^{19}F{^{1}H} NMR** (470 MHz, $CDCl_3$, 299 K) δ [ppm] = -62.5 (s, 3F, F3), -143.6 (s, 1F, F6). **IR** (ATR) $\tilde{\nu}$ [cm^{-1}] = 2985 (w), 1754 (m), 1740 (m), 1622 (w), 1446 (w), 1324 (s), 1274 (s), 1220 (m), 1189 (m), 1164 (m), 1120 (s), 1105 (s), 1060 (s), 897 (m), 825 (m), 765 (w), 687 (w). **HRMS** (ESI) *m/z* $[M+Na]^+$, calcd. for $C_{14}H_{12}O_2F_4Na^+$: 311.0666, found: 311.0665.

2-Fluoro-6-methoxy-2,3-dihydro-1*H*-inden-1-one (S53)

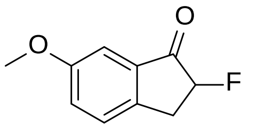

According to **general procedure B**, 6-methoxyindanone (0.81 g, 5.0 mmol, 1.0 eq.) and Selectfluor (2.1 g, 6.0 mmol, 1.2 eq.) were refluxed in MeOH (35 mL) for 16 h. Column chromatography purification (*n*-pentane:EtOAc 20:1) afforded product **S53** as white solid (0.64 g, 3.5 mmol, 71%).

R_f (*n*-pentane:EtOAc 10:1) = 0.27. **M.p.** 65 – 66 °C. **^{1}H NMR** (400 MHz, $CDCl_3$, 299 K) δ [ppm] = 7.35 (dt, *J* = 8.5, 0.8 Hz, 1H), 7.28 – 7.21 (m, 2H), 5.27 (ddd, *J* = 50.9, 7.7, 4.3 Hz, 1H), 3.84 (s, 3H), 3.62 – 3.50 (m, 1H), 3.22 – 3.07 (m, 1H). **^{19}F NMR** (376 MHz, $CDCl_3$, 299 K) δ [ppm] = -193.2 (ddd, *J* = 51.3, 22.8, 6.7 Hz, 1F). **^{19}F{^{1}H} NMR** (376 MHz, $CDCl_3$, 299 K) δ [ppm] = -193.2 (s, 1F). **HRMS** (ESI) *m/z* $[M+Na]^+$, calcd. for $C_{10}H_9O_2FNa^+$: 203.0479, found: 203.0479. Analytic data in agreement with the literature.[5]

2-Fluoro-6-methoxy-1-methylene-2,3-dihydro-1*H*-indene (S54)

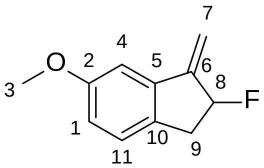

According to **general procedure C**, Ph_3PMeBr (1.4 g, 3.8 mmol, 1.5 eq.) and KO^{t}Bu (0.46 g, 3.8 mmol, 1.5 eq.) were dissolved in THF (5 mL). 2-Fluoro-6-methoxy-2,3-dihydro-1*H*-inden-1-one (**S53**) (0.45 g, 2.5 mmol, 1.0 eq.) was

added and the reaction mixture was stirred for 16 h. Column chromatography purification (*n*-pentane) afforded product **S54** as colourless oil (0.29 g, 1.6 mmol, 64%).

R$_f$ (*n*-pentane) = 0.11. **^{1}H NMR** (500 MHz, $CDCl_3$, 299 K) δ [ppm] = 7.16 (d, $^3J_{HH}$ = 8.7 Hz, 1H, H11), 7.01 (d, $^4J_{HH}$ = 2.5 Hz, 1H, H4), 6.86 (dd, $^3J_{HH}$ = 8.3 Hz, $^4J_{HH}$ = 2.5 Hz, 1H, H1), 5.73 (dd, $^4J_{FH}$ = 5.3 Hz, $^4J_{HH}$ = 1.6 Hz, 1H, H7^a), 5.65 (dddt, $^2J_{FH}$ = 55.4 Hz, $^3J_{HH}$ = 6.7, 2.9 Hz, $^4J_{HH}$ = 1.5 Hz, 1H, H8), 5.48 (dd, $^4J_{FH}$ = 5.3 Hz, $^4J_{HH}$ = 1.4 Hz, 1H, H7^b), 3.83 (s, 3H, H3), 3.27 (ddd, $^3J_{FH}$ = 22.3 Hz, $^2J_{HH}$ = 17.0 Hz, $^3J_{HH}$ = 6.5 Hz, 1H, H9^a), 3.08 (ddd, $^3J_{FH}$ = 28.7 Hz, $^2J_{HH}$ = 17.1 Hz, $^3J_{HH}$ = 2.3 Hz, 1H, H9^b). **^{13}C NMR** (126 MHz, $CDCl_3$, 299 K) δ [ppm] = 159.5 (1C, C2), 148.7 (d, $^2J_{FC}$ = 13.9 Hz, 1C, C6), 139.4 (d, $^3J_{FC}$ = 2.3 Hz, 1C, C5), 134.3 (d, $^3J_{FC}$ = 1.2 Hz, 1C, C10), 126.3 (d, $^4J_{FC}$ = 1.7 Hz, 1C, C11), 116.7 (1C, C1), 109.4 (d, $^3J_{FC}$ = 7.7 Hz, 1C, C7), 105.3 (1C, C4), 94.7 (d, $^1J_{FC}$ = 177.8 Hz, 1C, C8), 55.6 (1C, C3), 37.5 (d, $^2J_{FC}$ = 22.7 Hz, 1C, C9). **^{19}F NMR** (470 MHz, $CDCl_3$, 299 K) δ [ppm] = -167.2 (dddt, $^2J_{HH}$ = 55.4 Hz, $^3J_{HH}$ = 28.7, 22.3 Hz, $^4J_{HH}$ = 5.7 Hz, 1F, F8). **^{19}F{^{1}H} NMR** (470 MHz, $CDCl_3$, 299 K) δ [ppm] = -167.2 (s, 1F, F8). **IR** (ATR) $\tilde{\upsilon}$ [cm^{-1}] = 2957 (w), 1648 (w), 1580 (w), 1486 (s), 1434 (w), 1302 (m), 1290 (m), 1228 (s), 1179 (m), 1079 (w), 1028 (m), 1016 (m), 896 (m), 848 (m), 806 (m), 727 (m), 661 (m). **GC-MS** (EI) *m/z* [M]$^+$, calcd. for $C_{11}H_{11}OF^+$: 178.0788, found: 178.0782.

2-Fluoro-3,4-dihydronapthalen-1(2*H*)-one (S55)

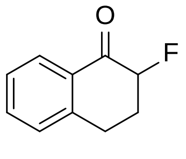

According to **general procedure B**, 3,4-dihydronapthalen-1(2*H*)-one (1.5 g, 9.1 mmol, 1.0 eq.) and Selectfluor (4.3 g, 12 mmol, 1.2 eq.) were refluxed in MeOH (70 mL) for 16 h. Column chromatography purification (*n*-pentane:EtOAc 20:1) afforded product **S55** as white solid (1.5 g, 9.1 mmol, 91%).

R$_f$ (*n*-pentane:EtOAc 10:1) = 0.32. **M.p.** 42 – 43 °C. **^{1}H NMR** (400 MHz, $CDCl_3$, 299 K) δ [ppm] = 8.06 (dd, *J* = 8.0, 1.5 Hz, 1H), 7.53 (td, *J* = 7.5, 1.5 Hz, 1H), 7.38 – 7.33 (m, 1H), 7.30 – 7.24 (m, 1H), 5.15 (ddd, *J* = 48.1, 12.7, 5.2 Hz, 1H), 3.18 – 3.09 (m, 2H), 2.58 (dddt, *J* = 12.5, 10.3, 5.2, 4.1 Hz, 1H), 2.44 – 2.27 (m, 1H). **^{19}F NMR** (376 MHz, $CDCl_3$, 299 K) δ [ppm] = -190.2 – -190.4 (m, 1F). **^{19}F{^{1}H} NMR** (376 MHz, $CDCl_3$, 299 K) δ [ppm] = -190.3 (s, 1F). **HRMS** (ESI) *m/z* [M+Na]$^+$, calcd. for $C_{10}H_9O_1FNa^+$: 187.0530, found: 187.0529. Analytic data in agreement with the literature.[5]

2-Fluoro-1-methylene-1,2,3,4-tetrahydronapthelene (S56)

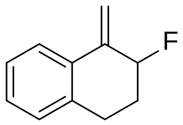

According to **general procedure C**, Ph_3PMeBr (2.7 g, 7.5 mmol, 1.5 eq.) and KO*t*Bu (0.84 g, 7.5 mmol, 1.5 eq.) were dissolved in THF (10 mL). 2-Fluoro-3,4-dihydronapthalen-1(2*H*)-one (**S55**) (0.82 g, 5.0 mmol, 1.0 eq.) was added and the reaction mixture was stirred for 16 h. Column chromatography purification (*n*-pentane) afforded product **S56** as colourless oil (0.65 g, 4.0 mmol, 80%).

R_f (*n*-pentane) = 0.37. **^{1}H NMR** (400 MHz, $CDCl_3$, 299 K) δ [ppm] = 7.67 – 7.64 (m, 1H), 7.24 – 7.18 (m, 2H), 7.14 (ddt, *J* = 6.2, 2.3, 0.9 Hz, 1H), 5.74 (s, 1H), 5.40 – 5.22 (m, 2H), 3.14 – 3.04 (m, 1H), 2.86 (dt, *J* = 16.8, 6.0 Hz, 1H), 2.31 – 2.20 (m, 1H), 2.20 – 2.03 (m, 1H). **^{19}F NMR** (376 MHz, $CDCl_3$, 299 K) δ [ppm] = -175.3 (ddd, *J* = 50.3, 29.8, 8.0 Hz, 1F). **^{19}F{^{1}H} NMR** (376 MHz, $CDCl_3$, 299 K) δ [ppm] = -175.3 (s, 1F). Analytic data in agreement with the literature.[4]

5-Bromo-1-methylene-2,3-dihydro-1*H*-indene (S57)

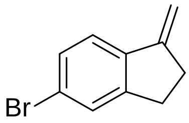

According to **general procedure C**, Ph_3PMeBr (1.1 g, 3.0 mmol, 1.5 eq.) and KO*t*Bu (0.34 g, 3.0 mmol, 1.5 eq.) were dissolved in THF (5 mL). 5-Bromo-indanone (0.42 g, 2.0 mmol, 1.0 eq.) was added and the reaction mixture was stirred for 16 h. Column chromatography purification (*n*-pentane) afforded product **S57** as colourless oil (0.34 g, 1.6 mmol, 81%).

R_f (*n*-pentane) = 0.57. **^{1}H NMR** (400 MHz, $CDCl_3$, 299 K) δ [ppm] = 7.41 – 7.39 (m, 1H), 7.36 – 7.29 (m, 2H), 5.44 (t, *J* = 2.5 Hz, 1H), 5.05 (t, *J* = 2.2 Hz, 1H), 3.00 – 2.93 (m, 2H), 2.84 – 2.75 (m, 2H). **GC-MS** (EI) *m/z* $[M]^+$, calcd. for $C_{10}H_9Br^+$: 207.9882, found: 207.9885. Analytic data in agreement with the literature.[22]

IV. Synthesis of Trifluorinated Tetralins

General procedure D for the difluorinative ring expansion

The corresponding 2-fluoro-methylen-dihydroindene (0.2 mmol, 1.0 eq.) and *p*-TolI (8.7 mg, 40 *µ*mol, 20 mol%) were dissolved in DCE (0.5 mL) in a Teflon® screw cap vial. Amine:HF 1:4.5 (0.5 mL) and Selectfluor (106 mg, 0.3 mmol, 1.5 eq.) were added and the reaction mixture was stirred for 24 h at ambient temperature. The reaction mixture was poured into a saturated solution of $NaHCO_3$ and the aqueous layer was extracted with CH_2Cl_2 (3x). The combined organic layers were dried over $MgSO_4$, filtered and the solvent was removed at max. 300 mbar. The yield was determined by ^{19}F NMR analysis of the crude reaction mixture using ethyl fluoroacetate as internal standard. The crude reaction mixture was purified by column chromatography to afford the desired product.

Caution: Fluorinated products might be volatile.

2,2,3-Trifluoro-1,2,3,4-tetrahydronaphthalene (9)

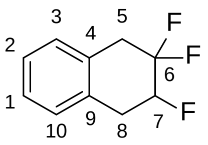

The reaction was performed according to **general procedure D** using 2-fluoro-1-methylene-2,3-dihydro-1*H*-indene (**8**) (30 mg, 0.2 mmol, 1.0 eq.). Column chromatography purification (*n*-pentane) afforded product **9** as white solid (19 mg, 0.10 mmol, 51%; ^{19}F NMR yield: 74%).

For a 1.0 mmol scale:

The reaction was performed according to **general procedure D** using 2-fluoro-1-methylene-2,3-dihydro-1*H*-indene **8** (0.15 g, 1.0 mmol, 1.0 eq.), *p*-TolI (44 mg, 0.2 mmol, 20 mol%), Selectfluor (0.53 g, 1.5 mmol, 1.5 eq.), amine:HF 1:4.5 (2.5 mL) and DCE (2.5 mL). Column chromatography purification (*n*-pentane) afforded product **9** as white solid (92 mg, 0.49 mmol, 49%).

R_f (*n*-pentane) = 0.32. **M.p.** 62 – 63 °C. **^{1}H NMR** (599 MHz, $CDCl_3$, 299 K) δ [ppm] = 7.24 – 7.20 (m, 2H, H1, H2), 7.19 – 7.14 (m, 1H, H10), 7.13 – 7.11 (m, 1H, H3), 4.97 (ddtt, J = 50.8, 10.1, 4.1, 1.5 Hz, 1H, H7), 3.48 (dddd, J = 28.1, 16.7, 11.3 Hz, 4.9 Hz, 1H, H5^a), 3.40-3.22 (m, 3H, H5^b/H8). **^{13}C NMR** (151 MHz, $CDCl_3$, 299 K) δ [ppm] = 130.4 (dd, $^3J_{FC}$ = 8.6, 2.6 Hz, 1C, C4), 130.1 – 130.0 (m, 1C, C9), 129.2 (1C, C10), 129.1 (d, $^4J_{FC}$ = 1.5 Hz, 1C, C3), 127.3 (1C, C1), 127.1 (1C, C2), 119.8 (ddd, $^1J_{FC}$ = 247.9, 239.7 Hz,

$^2J_{FC}$ = 25.0 Hz, 1C, C6), 86.7 (ddd, $^1J_{FC}$ = 180.1 Hz, $^2J_{FC}$ = 34.9, 25.4 Hz, 1C, C7), 34.7 – 34.3 (m, 1C, C5), 33.7 (ddd, $^2J_{FC}$ = 21.5 Hz, $^3J_{FC}$ = 5.1, 1.7 Hz, 1C, C8). **^{19}F NMR** (564 MHz, $CDCl_3$, 299 K) δ [ppm] = -109.3 – -109.8 (m, 1F, F6^a), -110.0 – -110.6 (m, 1F, F6^b), -196.9 – -197.2 (m, 1F, F7). **^{19}F{^{1}H} NMR** (564 MHz, $CDCl_3$, 299 K) δ [ppm] = -109.6 (dd, $^2J_{FF}$ = 251.8 Hz, $^3J_{FF}$ = 14.7 Hz, 1F, F6^a), -110.4 (dd, $^2J_{FF}$ = 251.9 Hz, $^3J_{FF}$ = 5.9 Hz, 1F, F6^b), -197.1 (dd, $^3J_{FF}$ = 14.7, 5.9 Hz, 1F, F7). **IR** (ATR) $\tilde{\nu}$ [cm^{-1}] = 2956 (w), 1608 (w), 1499 (w), 1459 (w), 1417 (m), 1389 (w), 1360 (w), 1295 (w), 1228 (m), 1197 (w), 1128 (m), 1097 (m), 1070 (s), 1047 (s), 1004 (s), 964 (w), 939 (w), 926 (w), 894 (w), 854 (m), 744 (s), 696 (m), 672 (m). **GC-MS** (EI) *m/z* [M]$^+$, calcd. for $C_{10}H_9F_3^+$: 186.0651, found: 186.0651.

2,2,3,6-Tetrafluoro-1,2,3,4-tetrahydronaphthalene (10)

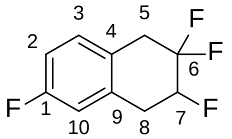

The reaction was performed according to **general procedure D** using 2,5-difluoro-1-methylene-2,3-dihydro-1*H*-indene (**S3**) (33 mg, 0.2 mmol, 1.0 eq.). Column chromatography purification (*n*-pentane) afforded product **10** as colourless oil (19 mg, 0.09 mmol, 46%; ^{19}F NMR yield: 57%).

R$_f$ (*n*-pentane) = 0.33. **^{1}H NMR** (600 MHz, $CDCl_3$, 299 K) δ [ppm] = 7.08 (dd, $^3J_{HH}$ = 8.5 Hz, $^4J_{FH}$ = 5.6 Hz, 1H, H3), 6.93 (ddd, $^3J_{HH}$ = 8.4 Hz, $^3J_{FH}$ = 8.4 Hz, $^4J_{HH}$ = 2.7 Hz, 1H, H2), 6.87 (dd, $^3J_{FH}$ = 9.3 Hz, $^4J_{HH}$ = 2.7 Hz, 1H, H10), 5.03 – 4.88 (m, 1H, H7), 3.50 – 3.29 (m, 2H, H5^a, H8^a), 3.29 – 3.18 (m, 2H, H5^b, H8^b). **^{13}C NMR** (151 MHz, $CDCl_3$, 299 K) δ [ppm] = 161.9 (d, $^1J_{FC}$ = 245.7 Hz, 1C, C1), 132.1 (d, $^3J_{FC}$ = 8.5 Hz, 1C, C9), 130.6 (dd, $^3J_{FC}$ = 8.2 Hz, $^5J_{FC}$ = 1.8 Hz, 1C, C3), 126.1 (dd, $^3J_{FC}$ = 7.1, 2.7 Hz, 1C, C4), 119.7 (ddd, $^1J_{FC}$ = 248.7, 239.4 Hz, $^2J_{FC}$ = 25.3 Hz, 1C, C6), 115.6 (dd, $^2J_{FC}$ = 21.7 Hz, $^4J_{FC}$ = 0.9 Hz, 1C, C10), 114.4 (dd, $^2J_{FC}$ = 21.6 Hz, $^5J_{FC}$ = 0.7 Hz, 1C, C2), 86.2 (ddd, $^1J_{FC}$ = 180.2 Hz, $^2J_{FC}$ = 36.0, 25.4 Hz, 1C, C7), 34.2 – 33.6 (m, 1C, C5), 33.71 (dddd, $^2J_{FC}$ = 21.7 Hz, $^3J_{FC}$ = 5.5, 1.5 Hz, $^4J_{FC}$ = 1.5 Hz, 1C, C8). **^{19}F NMR** (564 MHz, $CDCl_3$, 299 K) δ [ppm] = -109.1 – -109.8 (m, 1F, F6^a), -110.3 – -110.9 (m, 1F, F6^b), -115.6 (ddd, $^3J_{FH}$ = 9.0, 8.6 Hz, $^4J_{FH}$ = 5.5 Hz, 1F, F1), -196.6 – -196.9 (m, 1F, F7). **^{19}F{^{1}H} NMR** (564 MHz, $CDCl_3$, 299 K) δ [ppm] = -109.4 (dd, $^2J_{FF}$ = 253.1 Hz, $^3J_{FF}$ = 15.2 Hz, 1F, F6^a), -110.6 (dd, $^2J_{FF}$ = 253.2 Hz, $^3J_{FF}$ = 5.1 Hz, 1F, F6^b), -115.6 (s, 1F, F1), -196.8 (dd, $^3J_{FF}$ = 15.2, 5.1 Hz, 1F, F7). **IR** (ATR) $\tilde{\nu}$ [cm^{-1}] = 2924 (w), 1618 (w), 1595 (w), 1504 (m), 1433 (w), 1391 (w), 1237 (s), 1144 (m), 1126

(s), 1077 (s), 1055 (w), 1011 (m), 954 (m), 891 (w), 840 (m), 809 (s), 744 (w), 669 (m). **GC-MS** (EI) *m/z* [M]$^+$, calcd. for $C_{10}H_8F_4^+$: 204.0557, found: 204.0557.

6-Chloro-2,2,3-trifluoro-1,2,3,4-tetrahydronaphthalene (11)

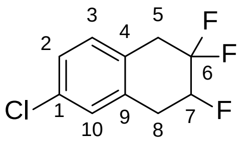

The reaction was performed according to **general procedure D** using 5-chloro-2-fluoro-1-methylene-2,3-dihydro-1*H*-indene (**S5**) (37 mg, 0.2 mmol, 1.0 eq.). Column chromatography purification (*n*-pentane) afforded product **11** as colourless oil (23 mg, 0.10 mmol, 52%; ^{19}F NMR yield: 73%).

R$_f$ (*n*-pentane) = 0.36. **^{1}H NMR** (500 MHz, $CDCl_3$, 299 K) δ [ppm] = 7.19 (dd, $^3J_{HH}$ = 8.2 Hz, $^4J_{HH}$ = 2.2 Hz, 1H, H2), 7.16 (d, $^4J_{HH}$ = 2.5 Hz, 1H, H10), 7.05 (d, $^3J_{HH}$ = 8.2 Hz, 1H, H3), 4.96 (ddtt, J = 49.2, 10.2, 4.0, 1.6 Hz, 1H, H7), 3.49 – 3.29 (m, 2H, H5^a, H8^a), 3.29 – 3.19 (m, 2H, H5^b, H8^b). **^{13}C NMR** (126 MHz, $CDCl_3$, 299 K) δ [ppm] = 133.1 (1C, C1), 132.0 – 131.6 (m, 1C, C9), 130.4 (d, $^4J_{FC}$ = 1.8 Hz, 1C, C3), 129.1 (d, $^4J_{FC}$ = 1.1 Hz, 1C, C10), 128.9 (dd, $^3J_{FC}$ = 9.3, 1.9 Hz, 1C, C4), 127.4 (d, $^5J_{FC}$ = 0.9 Hz, 1C, C2), 119.5 (ddd, $^1J_{FC}$ = 248.7, 239.4 Hz, $^2J_{FC}$ = 25.5 Hz, 1C, C6), 86.2 (ddd, $^1J_{FC}$ = 180.2 Hz, $^2J_{FC}$ = 36.0, 25.5 Hz, 1C, C7), 34.0 (ddd, $^2J_{FC}$ = 25.7, 24.6 Hz, $^3J_{FC}$ = 1.0 Hz, 1C, C5), 33.5 (ddd, $^2J_{FC}$ = 21.7 Hz, $^3J_{FC}$ = 5.3, 1.4 Hz, 1C, C8). **^{19}F NMR** (470 MHz, $CDCl_3$, 299 K) δ [ppm] = -108.9 – -109.6 (m, 1F, F6^a), -110.0 – -110.7 (m, 1F, F6^b), -196.6 – -196.9 (m, 1F, F7). **^{19}F{^{1}H} NMR** (470 MHz, $CDCl_3$, 299 K) δ [ppm] = -109.2 (dd, $^2J_{FF}$ = 253.7 Hz, $^3J_{FF}$ = 14.9 Hz, 1F, F6^a), -110.4 (dd, $^2J_{FF}$ = 253.7 Hz, $^3J_{FF}$ = 5.4 Hz, 1F, F6^b), -196.8 (dd, $^3J_{FF}$ = 14.9, 5.3 Hz, 1F, F7). **IR** (ATR) $\tilde{\nu}$ [cm^{-1}] = 2925 (w), 1600 (w), 1490 (w), 1420 (w), 1390 (w), 1338 (w), 1229 (m), 1179 (m), 1138 (m), 1078 (s), 1054 (s), 1009 (m), 930 (m), 844 (w), 805 (s), 708 (w), 667 (w). **GC-MS** (EI) *m/z* [M]$^+$, calcd. for $C_{10}H_8ClF_3^+$: 220.0261, found: 220.0261.

6-Bromo-2,2,3-trifluoro-1,2,3,4-tetrahydronaphthalene (12)

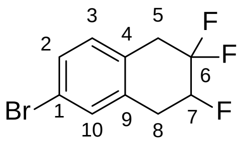

The reaction was performed according to **general procedure D** using 5-bromo-2-fluoro-1-methylene-2,3-dihydro-1*H*-indene (**S7**) (45 mg, 0.2 mmol, 1.0 eq.). Column chromatography purification (*n*-pentane) afforded product **12** as white solid (36 mg, 0.13 mmol, 76%; ^{19}F NMR yield: 92%).

R$_f$ (*n*-pentane) = 0.32. **M.p.** 38 – 39 °C. **^{1}H NMR** (500 MHz, $CDCl_3$, 299 K) δ [ppm] = 7.34 (dd, $^3J_{HH}$ = 8.1 Hz, $^4J_{HH}$ = 2.1 Hz, 1H, H2), 7.32 (d, $^4J_{HH}$ = 1.8 Hz, 1H, H10), 6.99 (d, $^3J_{HH}$ = 8.1 Hz, 1H, H3), 4.95 (ddtt, *J* = 49.2, 10.2, 3.8, 1.3 Hz, 1H, H7), 3.47 – 3.29 (m, 2H, H5^a, H8^a), 3.27 – 3.17 (m, 2H, H5^b, H8^b). **^{13}C NMR** (126 MHz, $CDCl_3$, 299 K) δ [ppm] = 132.2 (1C, C9), 132.0 (d, $^4J_{FC}$ = 1.1 Hz, 1C, C10), 130.7 (d, $^4J_{FC}$ = 1.7 Hz, 1C, C3), 130.2 (d, $^5J_{FC}$ = 0.9 Hz, 1C, C2), 129.5 (dd, $^3J_{FC}$ = 9.6 Hz, 1.7 Hz, 1C, C4), 121.0 (1C, C1), 118.7 (ddd, $^1J_{FC}$ = 248.9, 239.2 Hz, $^2J_{FC}$ = 25.8 Hz, 1C, C6), 86.2 (ddd, $^1J_{FC}$ = 180.3 Hz, $^2J_{FC}$ = 36.1, 25.5 Hz, 1C, C7), 34.1 (ddd, $^2J_{FC}$ = 25.7, 24.7, $^3J_{FC}$ = 0.9 Hz, 1C, C5), 33.4 (ddd, $^2J_{FC}$ = 21.7, $^3J_{FC}$ = 5.3, 1.4 Hz, 1C, C8). **^{19}F NMR** (470 MHz, $CDCl_3$, 299 K) δ [ppm] = -108.9 – -109.6 (m, 1F, F6^a), -110.0 – -110.7 (m, 1F, F6^b), -196.6 – -196.9 (m, 1F, F7). **^{19}F{^{1}H} NMR** (470 MHz, $CDCl_3$, 299 K) δ [ppm] = -109.2 (dd, $^2J_{FF}$ = 253.8 Hz, $^3J_{FF}$ = 15.0 Hz), -1104 (dd, $^2J_{FF}$ = 253.9 Hz, $^3J_{FF}$ = 5.4 Hz), -196.7 (dd, $^3J_{FF}$ = 14.9, 5.4 Hz). **IR** (ATR) $\tilde{\nu}$ [cm^{-1}] = 2958 (w), 1590 (w), 1488 (m), 1420 (w), 1389 (w), 1337 (w), 1289 (w), 1213 (w), 1138 (m), 1077 (s), 1056 (s), 1008 (m), 962 (w), 896 (w), 847 (w), 804 (m), 710 (w). **GC-MS** (EI) *m/z* [M]$^+$, calcd. for $C_{10}H_8BrF_3^+$: 263.9756, found: 263.9757.

7-Bromo-2,2,3-trifluoro-1,2,3,4-tetrahydronaphthalene (13)

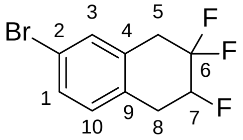

The reaction was performed according to **general procedure D** using 6-bromo-2-fluoro-1-methylene-2,3-dihydro-1*H*-indene (**S9**) (45 mg, 0.2 mmol, 1.0 eq.). Column chromatography purification (*n*-pentane) afforded product **13** as white solid (41 mg, 0.15 mmol, 77%; ^{19}F NMR yield: >95%).

R$_f$ (*n*-pentane) = 0.31. **M.p.** 29 – 30 °C. **^{1}H NMR** (599 MHz, $CDCl_3$, 299 K) δ [ppm] = 7.34 (dd, $^3J_{HH}$ = 8.3 Hz, $^4J_{HH}$ = 2.1 Hz, 1H, H1), 7.28 (d, $^4J_{HH}$ = 2.1 Hz, 1H, H3), 7.03 (d, $^3J_{HH}$ = 8.2 Hz, 1H, H10), 4.96 (ddtt, *J* = 49.0, 10.1, 4.4, 1.4 Hz, 1H, H7), 3.45 (dddd, *J* = 28.2, 17.7, 10.5, 4.9 Hz, 1H, H5^a), 3.29 – 3.21 (m, 3H, H5^b, H8). **^{13}C NMR** (151 MHz, $CDCl_3$, 299 K) δ [ppm] = 132.7 (dd, $^3J_{FC}$ = 9.0, 2.3 Hz, 1C, C4), 131.9 (d, $^4J_{FC}$ = 1.7 Hz, 1C, C3), 130.8 (d, $^4J_{FC}$ = 1.1 Hz, 1C, C10), 130.5 (1C, C1), 129.1 – 128.9 (m, 1C, C9), 120.8 (d, $^5J_{FC}$ = 1.2 Hz, 1C, C2), 119.3 (ddd, $^1J_{FC}$ = 248.5, 239.8 Hz, $^2J_{FC}$ = 25.5 Hz, 1C, C6), 86.3 (ddd, $^1J_{FC}$ = 180.2 Hz, $^2J_{FC}$ = 35.5, 25.6 Hz, 1C, C7), 34.2 (ddd, $^2J_{FC}$ = 25.9, 24.8 Hz, $^3J_{FC}$ = 1.1 Hz, 1C, C5), 33.2 (ddd, $^2J_{FC}$ = 21.7 Hz, $^3J_{FC}$ = 5.2, 1.5 Hz, 1C, C8). **^{19}F NMR** (564 MHz, $CDCl_3$, 299 K)

δ [ppm] = -109.2 – -109.8 (m, 1F, F6[a]), -110.0 – -110.6 (m, 1F, F6[b]), -196.5 – -197.1 (m, 1F, F7). **^{19}F{^{1}H} NMR** (564 MHz, $CDCl_3$, 299 K) *δ* [ppm] = -109.5 (dd, $^{2}J_{FF}$ = 253.8 Hz, $^{3}J_{FF}$ = 14.7 Hz, 1F, F6[a]), -110.3 (dd, $^{2}J_{FF}$ = 253.9 Hz, $^{3}J_{FF}$ = 5.7 Hz), -196.81 (dd, $^{3}J_{FH}$ = 14.7, 5.6 Hz, 1C, C7). **IR** (ATR) $\tilde{\upsilon}$ [cm^{-1}] = 2958 (w), 1574 (w), 1488 (m), 1388 (w), 1340 (w), 1285 (w), 1107 (m), 1079 (s), 1055 (s), 1006 (m), 962 (w), 804 (m), 708 (w). **GC-MS** (EI) *m/z* [M]$^{+}$, calcd. for $C_{10}H_8BrF_3^{+}$: 263.9756, found: 263.9757.

5-Bromo-2,2,3-trifluoro-1,2,3,4-tetrahydronaphthalene (14)

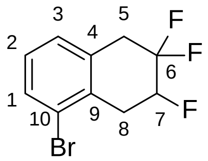

The reaction was performed according to **general procedure D** using 4-bromo-2-fluoro-1-methylene-2,3-dihydro-1*H*-indene (**S11**) (45 mg, 0.2 mmol, 1.0 eq.). Column chromatography purification (*n*-pentane) afforded product **14** as white solid (38 mg, 0.14 mmol, 70%; ^{19}F NMR yield: 94%).

R_f (*n*-pentane) = 0.36. **M.p.** 42 – 43 °C. **^{1}H NMR** (599 MHz, $CDCl_3$, 299 K) *δ* [ppm] = 7.49 (dd, *J* = 6.6, 2.4 Hz, 1H, H1), 7.12 – 7.06 (m, 2H, H2, H3), 5.00 (ddt, *J* = 48.9, 10.3, 4.1 Hz, 1H, H7), 3.57 – 3.38 (m, 2H, H5[a], H8[a]), 3.30 – 3.14 (m, 2H, H5[b], H8[b]). **^{13}C NMR** (151 MHz, $CDCl_3$, 299 K) *δ* [ppm] = 132.7 (dd, $^{3}J_{FC}$ = 7.9, 1.6 Hz, 1C, C4), 131.6 (1C, C1), 130.1 (m, 2C, C2, C3), 128.4 (d, $^{3}J_{FC}$ = 1.1 Hz, 1C, C9), 125.4 (d, $^{4}J_{FC}$ = 1.2 Hz, 1C, C10), 119.9 (ddd, $^{1}J_{FC}$ = 248.8, 238.6 Hz, $^{2}J_{FC}$ = 25.7 Hz, 1C, C6), 86.3 (ddd, $^{1}J_{FC}$ = 179.3 Hz, $^{2}J_{FC}$ = 36.8, 25.2 Hz, 1C, C7), 35.1 (ddd, $^{2}J_{FC}$ = 22.1 Hz, $^{3}J_{FC}$ = 5.5, 1.1 Hz, 1C, C8), 34.3 (ddd, $^{2}J_{FC}$ = 25.6, 24.4 Hz, $^{3}J_{FC}$ = 1.2 Hz, 1C, C5). **^{19}F NMR** (564 MHz, $CDCl_3$, 299 K) *δ* [ppm] = -110.1 – -110.7 (m, 1F, F6[a]), -111.3 – -111.5 (m, 1F, F6[b]), -194.2 – -194.5 (m, 1F, F7). **^{19}F{^{1}H} NMR** (564 MHz, $CDCl_3$, 299 K) *δ* [ppm] = -110.4 (dd, $^{2}J_{FF}$ = 253.8 Hz, $^{3}J_{FF}$ = 15.4 Hz, F6[a]), -111.6 (dd, $^{2}J_{FF}$ = 253.7 Hz, $^{3}J_{FF}$ = 5.1 Hz, F6[b]), -194.4 (dd, $^{3}J_{FF}$ = 15.3, 5.1 Hz, F7). **IR** (ATR) $\tilde{\upsilon}$ [cm^{-1}] = 2960 (w), 2924 (w), 1597 (w), 1566 (m), 1460 (m), 1446 (m), 1425 (m), 1414 (m), 1387 (m), 1347 (m), 1317 (w), 1287 (w), 1223 (m), 1184 (w), 1175 (w), 1139 (m), 1107 (s), 1072 (s), 1053 (s), 1004 (m), 934 (m), 904 (m), 883 (w), 862 (m), 840 (m), 773 (s), 694 (m). **GC-MS** (EI) *m/z* [M]$^{+}$, calcd. for $C_{10}H_8BrF_3^{+}$: 263.9756, found: 263.9763.

2,2,3-Trifluoro-6-methyl-1,2,3,4-tetrahydronaphthalene (15)

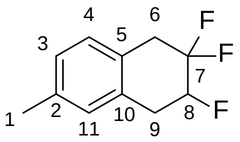

The reaction was performed according to **general procedure D** using 2-fluoro-5-methyl-1-methylene-2,3-dihydro-1*H*-indene (**S13**) (32 mg, 0.2 mmol, 1.0 eq.). Column chromatography purification (*n*-pentane) afforded product **15** as colourless oil (12 mg, 0.06 mmol, 31%; ^{19}F NMR yield: 37%).

R$_f$ (*n*-pentane) = 0.23. **^{1}H NMR** (599 MHz, $CDCl_3$, 299 K) δ [ppm] = 7.04 – 6.99 (m, 2H, H3, H4), 6.97 (s, 1H, H11), 5.01 – 4.88 (m, 1H, H8), 3.42 (dddd, *J* = 28.2, 17.1, 11.1, 4.9 Hz, 1H, H6^a), 3.35 – 3.19 (m, 3H, H6^b, H9), 2.31 (s, 3H, H1). **^{13}C NMR** (151 MHz, $CDCl_3$, 299 K) δ [ppm] = 137.0 (1C, C2), 129.9 – 129.8 (m, 1C, C10), 129.7 (1C, C11), 128.9 (d, $^4J_{FC}$ = 1.6 Hz, 1C, C4), 127.9 (1C, C3), 127.3 (dd, $^3J_{FC}$ = 7.7, 2.3 Hz, 1C, C5), 121.8 – 117.8 (m, 1C, C7), 86.8 (ddd, $^1J_{FC}$ = 180.2 Hz, $^2J_{FC}$ = 34.8, 25.4 Hz, 1C, C8), 34.4 (dd, $^2J_{FC}$ = 24.9, 26.0 Hz, 1C, C6), 33.6 (ddd, $^2J_{FC}$ = 21.5 Hz, $^3J_{FC}$ = 5.0, 1.7 Hz, 1C, C9), 21.1 (1C, C1). **^{19}F NMR** (564 MHz, $CDCl_3$, 299 K) δ [ppm] = -109.4 – -109.9 (m, 1F, F7^a), -110.2 – -110.7 (m, 1F, F7^b), -197.0 – -197.4 (m, 1F, F8). **^{19}F{^{1}H} NMR** (564 MHz, $CDCl_3$, 299 K) δ [ppm] = -109.6 (dd, $^2J_{FF}$ = 251.3 Hz, $^3J_{FF}$ = 14.8 Hz, 1F, F7^a), -110.5 (dd, $^2J_{FF}$ = 251.3 Hz, $^3J_{FF}$ = 5.9 Hz, 1F, F7^b), -197.2 (dd, $^3J_{FF}$ = 14.7, 5.9 Hz, 1F, F8). **IR** (ATR) $\tilde{\nu}$ [cm^{-1}] = 2924 (w), 1508 (w), 1422 (w), 1227 (m), 1131 (m), 1104 (m), 1079 (s), 1056 (m), 1017 (w), 806 (m), 677 (w). **GC-MS** (EI) *m/z* [M]$^+$, calcd. for $C_{11}H_{11}F_3^+$: 200.0807, found: 200.0807.

6,6,7-Trifluoro-5,6,7,8-tetrahydronaphthalen-2-yl trfluoromethanesulfonate (16)

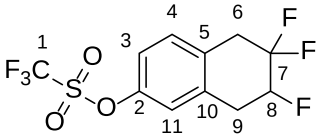

The reaction was performed according to **general procedure D** using 2-fluoro-1-methylene-2,3-dihydro-1*H*-inden-5-yl trifluoromethanesulfonate (**S17**) (59 mg, 0.2 mmol, 1.0 eq.). Column chromatography purification (*n*-pentane:EtOAc 25:1) afforded product **16** as colourless oil (51 mg, 0.15 mmol, 75%; ^{19}F NMR yield: 91%).

R$_f$ (*n*-pentane:EtOAc 25:1) = 0.38. **^{1}H NMR** (599 MHz, $CDCl_3$, 299 K) δ [ppm] = 7.21 (d, $^3J_{HH}$ = 8.5 Hz, 1H, H4), 7.14 (dd, $^3J_{HH}$ = 8.5 Hz, $^4J_{HH}$ = 2.6 Hz, 1H, H3), 7.09 (d, $^4J_{HH}$ = 2.6 Hz, 1H, H11), 4.99 (dddd, *J* = 48.9, 9.9, 4.3, 3.8 Hz, 1H, H8), 3.48 (dddd, *J* = 30.8, 17.4, 9.5, 4.6 Hz, 1H, H6^a), 3.39 – 3.25 (m, 3H,

H6[b], H9). **^{13}C NMR** (151 MHz, $CDCl_3$, 299 K) δ [ppm] = 148.6 (1C, C2), 132.8 (1C, C10), 131.1 (dd, $^{3}J_{FC}$ = 9.7, 1.4 Hz, 1C, C5), 131.0 (d, $^{4}J_{FC}$ = 1.8 Hz, 1C, C4), 121.9 (1C, C11), 120.1 (1C, C3), 119.2 (ddd, $^{1}J_{FC}$ = 249.3, 239.1 Hz, $^{2}J_{FC}$ = 25.8 Hz, 1C, C7), 118.9 (q, $^{1}J_{FC}$ = 320.7 Hz, 1C, C1), 85.9 (ddd, $^{1}J_{FC}$ = 180.3 Hz, $^{2}J_{FC}$ = 36.7, 25.5 Hz, 1C, C8), 34.0 (t, $^{2}J_{FC}$ = 25.5 Hz, 1C, C6), 33.7 (ddd, $^{2}J_{FC}$ = 21.7 Hz, $^{3}J_{FC}$ = 5.5, 1.3 Hz, 1C, C9). **^{19}F NMR** (564 MHz, $CDCl_3$, 299 K) δ [ppm] = -72.9 (s, 3F, F1), -108.5 – -109.7 (m, 1F, F7[a]), -109.8 – -110.9 (m, 1F, F7[b]), -196.3 – -196.8 (m, 1F, F8). **^{19}F{^{1}H} NMR** (564 MHz, $CDCl_3$, 299 K) δ [ppm] = -72.9 (s, 3F, F1), -109.2 (dd, $^{2}J_{FF}$ = 255.3 Hz, $^{3}J_{FF}$ = 15.2 Hz, 1F, F7[a]), -110.3 (dd, $^{2}J_{FF}$ = 255.3 Hz, $^{3}J_{FF}$ = 5.1 Hz, 1F, F7[b]), -196.5 (dd, $^{3}J_{FF}$ = 15.2, 5.1 Hz, 1F, F8). **IR** (ATR) $\tilde{\upsilon}$ [cm^{-1}] = 1613 (w), 1497 (m), 1420 (s), 1342 (w), 1249 (m), 1216 (s), 1138 (s), 1084 (s), 1061 (m), 1011 (m), 942 (m), 919 (m), 882 (m), 843 (m), 813 (m), 785 (m), 761 (w), 725 (w). **GC-MS** (EI) *m/z* $[M]^+$, calcd. for $C_{11}H_8O_3SF_6^+$: 334.0094, found: 334.0090.

2,2,3Trifluoro-7-(trifluoromethyl)-1,2,3,4-tetrahydronaphthalene (17)

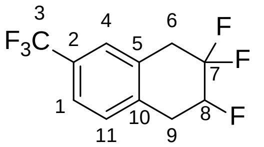

The reaction was performed according to **general procedure D** using 2-fluoro-1-methylene-6-(trifluoromethyl)-2,3-dihydro-1*H*-indene (**S20**) (43 mg, 0.2 mmol, 1.0 eq.). Column chromatography purification (*n*-pentane) afforded product **17** as colourless oil (38 mg, 0.15 mmol, 75%; ^{19}F NMR yield: 92%).

R_f (*n*-pentane) = 0.25. **^{1}H NMR** (500 MHz, $CDCl_3$, 299 K) δ [ppm] = 7.52 (dd, $^{3}J_{HH}$ = 8.1 Hz, $^{4}J_{HH}$ = 2.1 Hz, 1H, H1), 7.44 (d, $^{4}J_{HH}$ = 2.1 Hz, 1H, H4), 7.35 (d, $^{3}J_{HH}$ = 8.1 Hz, 1H, H11), 5.13 – 4.98 (m, 1H, H8), 3.55 (dddd, *J* = 26.0, 17.9, 13.0, 4.9 Hz, 1H, H6[a]), 3.46 – 3.32 (m, 3H, H6[b], H9). **^{13}C NMR** (126 MHz, $CDCl_3$, 299 K) δ [ppm] = 135.0 (1C, C10), 131.9 (dd, $^{3}J_{FC}$ = 8.4 Hz, $^{3}J_{FC}$ = 3.2 Hz, 1C, C5), 130.3 (d, $^{4}J_{FC}$ = 0.9 Hz, 1C, C11), 129.7 (q, $^{2}J_{FC}$ = 32.5 Hz, 1C, C2), 126.4 (qd, $^{3}J_{FC}$ = 3.7 Hz, $^{4}J_{FC}$ = 1.5 Hz, 1C, C4), 124.7 (q, $^{1}J_{FC}$ = 271.9 Hz, 1C, C3), 124.5 (q, $^{3}J_{FC}$ = 3.8 Hz, 1C, C1), 119.9 (ddd, $^{1}J_{FC}$ = 247.0, 240.6 Hz, $^{2}J_{FC}$ = 25.3 Hz, 1C, C7), 86.8 (ddd, $^{1}J_{FC}$ = 179.7 Hz, $^{2}J_{FC}$ = 34.1, 26.8 Hz, 1C, C8), 34.9 (ddd, $^{2}J_{FC}$ = 26.1, 25.0 Hz, $^{3}J_{FC}$ = 1.0 Hz, 1C, C6), 34.0 (ddd, $^{2}J_{FC}$ = 21.7 Hz, $^{3}J_{FC}$ = 4.7, 2.1 Hz, 1C, C9). **^{19}F NMR** (470 MHz, $CDCl_3$, 299 K) δ [ppm] = -63.0 (s, 3F, F3), -109.7 – -110.5 (m, 1F, F7[a]), -110.5 – -111.3 (m, 1F, F7[b]), -197.0 – -197.5 (m, 1F, F8). **^{19}F{^{1}H} NMR** (470 MHz, $CDCl_3$, 299 K) δ [ppm] = -63.0 (s, 3F,

F3), -110.1 (dd, $^2J_{FF}$ = 253.3 Hz, $^3J_{FF}$ = 13.6 Hz, 1F, F7[a]), -110.8 (dd, $^2J_{FF}$ = 253.4 Hz, $^3J_{FF}$ = 6.9 Hz, 1F, F7[b]), -197.3 (dd, $^3J_{FF}$ = 13.6, 6.9 Hz, 1F, F8). **IR** (ATR) $\tilde{\nu}$ [cm^{-1}] = 2971 (w), 1625 (w), 1435 (m), 1391 (m), 1330 (s), 1278 (m), 1263 (m), 1232 (m), 1215 (w), 1163 (m), 1120 (s), 1075 (s), 1055 (s), 1006 (m), 964 (w), 948 (m), 912 (m), 892 (m), 871 (w), 849 (m), 817 (s), 794 (w), 758 (w), 733 (w), 713 (m). **GC-MS** (EI) *m/z* [M]$^+$, calcd. for $C_{11}H_8F_6^+$: 254.0525, found: 254.0524.

6,6,7-Trifluoro-5,6,7,8-tetrahydronaphthalene-2-carbonitrile (18)

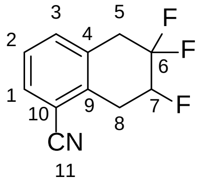

The reaction was performed according to **general procedure D** using 2-fluoro-1-methylene-2,3-dihydro-1*H*-indene-5-carbonitrile (**S22**) (34 mg, 0.2 mmol, 1.0 eq.). Column chromatography purification (*n*-pentane:Et_2O 19:1) afforded product **18** as white solid (33 mg, 0.16 mmol, 79%; ^{19}F NMR yield: 85%).

R$_f$ (*n*-pentane:Et_2O 9:1) = 0.47. **M.p.** 41 – 42 °C. **^{1}H NMR** (599 MHz, CD_2Cl_2, 299 K) δ [ppm] = 7.62 (d, $^3J_{HH}$ = 7.0 Hz, 1H, H1), 7.41 (d,$^3J_{HH}$ = 7.8 Hz, 1H, H3), 7.37 (dd, $^3J_{HH}$ = 7.7, 7.2 Hz, 1H, H2), 5.09 (ddt, J = 48.7, 9.9, 3.8 Hz, 1H, H7), 3.64 – 3.29 (m, 4H, H5, H8). **^{13}C NMR** (151 MHz, CD_2Cl_2, 299 K) δ [ppm] = 134.2 (dd, $^3J_{FC}$ = 1.6 Hz, $^4J_{FC}$ = 1.2 Hz, 1C, C9), 134.1 (d, $^4J_{FC}$ = 1.5 Hz, 1C, C3), 132.7 (dd, $^3J_{FC}$ = 9.6, 2.0 Hz, 1C, C4), 132.4 (1C, C1), 128.0 (1C, C2), 119.7 (ddd, $^1J_{FC}$ = 248.5, 239.1 Hz, $^2J_{FC}$ = 25.7 Hz, 1C, C6), 117.6 (1C, C11), 113.7 (d, $^4J_{FC}$ = 1.1 Hz, 1C, C10), 86.2 (ddd, $^1J_{FC}$ = 179.8 Hz, $^2J_{FC}$ = 36.4, 25.7 Hz, 1C, C7), 34.5 (ddd, $^2J_{FC}$ = 26.0, 24.7 Hz, $^3J_{FC}$ = 1.1 Hz, 1C, C5), 33.2 (ddd, $^2J_{FC}$ = 22.0 Hz, $^3J_{FC}$ = 5.5, 1.3 Hz, 1C, C8). **^{19}F NMR** (564 MHz, CD_2Cl_2, 299 K) δ [ppm] = -109.3 – -110.8 (m, 1F, F6[a]), -110.8 – -111.8 (m, 1F, F6[b]), -195.7 – -196.7 (m, 1F, F7). **^{19}F{^{1}H} NMR** (564 MHz, CD_2Cl_2, 299 K) δ [ppm] = -110.3 (dd, $^2J_{FF}$ = 254.6 Hz, $^3J_{FF}$ = 15.0 Hz, 1F, F6[a]), -111.2 (dd, $^2J_{FF}$ = 254.6 Hz, $^3J_{FF}$ = 5.3 Hz, 1F, F6[b]), -196.3 (dd, $^3J_{FF}$ = 15.1, 5.3 Hz, 1F, F7). **IR** (ATR) $\tilde{\nu}$ [cm^{-1}] = 2944 (w), 2232 (m), 1731 (w), 1592 (w), 1467 (m), 1429 (w), 1414 (m), 1394 (m), 1355 (w), 1320 (w), 1293 (m), 1225 (m), 1199 (w), 1186 (w), 1170 (w), 1123 (s), 1075 (s), 1051 (s), 1003 (s), 979 (m), 935 (w), 903 (w), 875 (w), 844 (m), 893 (s), 707 (m), 697 (m), 665 (w). **GC-MS** (EI) *m/z* [M]$^+$, calcd. for $C_{11}H_8NF_3^+$: 211.0603, found: 211.0596.

2-(6,7,7-Trifluoro-5,6,7,8-tetrahydronaphthalen-2-yl)isoindoline-1,3-dione (19)

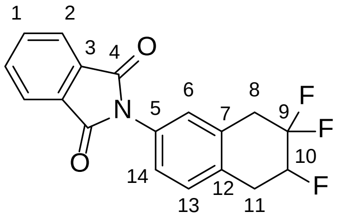

The reaction was performed according to **general procedure D** using 2-(2-fluoro-3-methylene-2,3-dihydro-1*H*-inden-5-yl)isoindoline-1,3-dione (**S25**) (59 mg, 0.2 mmol, 1.0 eq.). Column chromatography purification (CH_2Cl_2:*n*-pentane 1:1) afforded product **19** as white solid (46 mg, 0.14 mmol, 69%; ^{19}F NMR yield: 70%).

R$_f$ (CH_2Cl_2) = 0.60. **M.p.** 122 – 123 °C. **^{1}H NMR** (500 MHz, $CDCl_3$, 299 K) δ [ppm] = 7.95 (m, 2H, H2), 7.80 (m, 2H, H1), 7.34 – 7.27 (m, 2H, H13, H14), 7.22 (s, 1H, H6), 4.99 (ddd, J = 49.1, 8.6, 3.5 Hz, 1H, H10), 3.54 (dddd, J = 25.8, 17.7, 13.1, 4.7 Hz, 1H, H8^a), 3.46 – 3.21 (m, 3H, H8^b, H11). **^{13}C NMR** (126 MHz, $CDCl_3$, 299 K) δ [ppm] = 167.3 (2C, C4), 134.6 (2C, C1), 131.8 (2C, C3), 131.6 (dd, $^3J_{FC}$ = 8.2, 3.2 Hz, 1C, C7), 130.6 (1C, C5), 130.2 (1C, C12), 130.0 (1C, C13), 127.0 (d, $^4J_{FC}$ = 1.4 Hz, 1C, C6), 125.6 (1C, C14), 123.9 (2C, C2), 119.5 (ddd, $^1J_{FC}$ = 247.2, 240.9 Hz, $^2J_{FC}$ = 25.3 Hz, 1C, C9), 86.4 (ddd, $^1J_{FC}$ = 180.4 Hz, $^2J_{FC}$ = 33.9, 26.7 Hz, 1C, C10), 34.5 (t, $^2J_{FC}$ = 25.2 Hz, 1C, C8), 33.5 (ddd, $^2J_{FC}$ = 21.5 Hz, $^3J_{FC}$ = 4.6, 1.7 Hz, 1C, C11). **^{19}F NMR** (470 MHz, $CDCl_3$, 299 K) δ [ppm] = -109.0 – -109.9 (m, 1F, F9^a), -109.9 – -111.0 (m, 1F, F9^b), -195.9 – -198.6 (m, 1F, F10). **^{19}F{^{1}H} NMR** (470 MHz, $CDCl_3$, 299 K) δ [ppm] = -109.5 (dd, $^2J_{FF}$ = 253.2 Hz, $^3J_{FF}$ = 13.6 Hz, 1F, F9^a), -110.3 (dd, $^2J_{FF}$ = 252.5 Hz, $^3J_{FF}$ = 7.2 Hz, 1F, F9^b), -196.8 (dd, $^3J_{FF}$ = 13.6, 7.0 Hz, 1F, F10). **IR** (ATR) $\tilde{\nu}$ [cm^{-1}] = 2926 (w), 1782 (w), 1716 (s), 1611 (w), 1586 (w), 1506 (m), 1467 (w), 1421 (w), 1376 (s), 1291 (m), 1227 (m), 1176 (w), 1130 (w), 1113 (m), 1097 (m), 1077 (s), 1052 (s), 1007 (m), 963 (w), 946 (m), 907 (m), 885 (m), 845 (w), 812 (m), 792 (m), 715 (s), 681 (m). **HRMS** (ESI) *m/z* [M+Na]$^+$, calcd. for $C_{18}H_{12}NO_2F_3Na^+$: 354.0712, found: 354.0714.

Methyl (*E*)-3-(6,6,7-trifluoro-5,6,7,8-tetrahydronaphthalen-2-yl)acrylate (20)

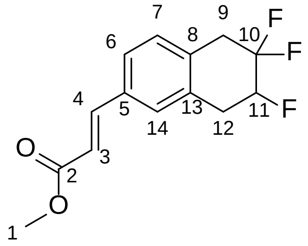

The reaction was performed according to **general procedure D** using methyl (*E*)-3-(2-fluoro-1-methylene-2,3-dihydro-1*H*-inden-5-yl)acrylate (**S27**) (46 mg, 0.2 mmol, 1.0 eq.). Column chromatography purification (*n*-pentane:EtOAc 20:1) afforded product **20** as colourless oil (32 mg, 0.12 mmol, 59%; ^{19}F NMR yield: 62%).

R_f (*n*-pentane:EtOAc 10:1) = 0.36. **^{1}H NMR** (500 MHz, $CDCl_3$, 299 K) δ [ppm] = 7.64 (d, $^3J_{HH}$ = 16.0 Hz, 1H, H4), 7.38 (dd, $^3J_{HH}$ = 8.1 Hz, $^4J_{HH}$ = 1.9 Hz, 1H, H6), 7.30 (d, $^4J_{HH}$ = 1.8 Hz, 1H, H14), 7.14 (d, $^3J_{HH}$ = 7.9 Hz, 1H, H7), 6.42 (d, $^3J_{HH}$ = 16.0 Hz, 1H, H3), 5.07 – 4.90 (m, 1H, H11), 3.81 (s, 3H, H1), 3.55 – 3.38 (m, 1H, H9^a), 3.38 – 3.23 (m, 3H, H9^b, H12). **^{13}C NMR** (126 MHz, $CDCl_3$, 299 K) δ [ppm] = 167.4 (1C, C2), 144.1 (1C, C4), 133.7 (1C, C5), 133.3 – 132.5 (m, 1C, C8), 130.8 (1C, C13), 129.7 (d, $^4J_{FC}$ = 1.5 Hz, 1C, C7), 129.0 (d, $^4J_{FC}$ = 0.9 Hz, 1C, C14), 126.5 (1C, C6), 119.5 (ddd, $^1J_{FC}$ = 247.6, 239.4 Hz, $^2J_{FC}$ = 24.5 Hz, 1C, C10), 118.2 (1C, C3), 86.4 (ddd, $^1J_{FC}$ = 180.1 Hz, $^2J_{FC}$ = 35.4, 25.7 Hz, 1C, C11), 51.9 (1C, C1), 34.5 (dd, $^2J_{FC}$ = 25.8, 24.6 Hz, 1C, C9), 33.6 (ddd, $^2J_{FC}$ = 21.6 Hz, $^3J_{FC}$ = 5.2, 1.6 Hz, 1C, C12). **^{19}F NMR** (470 MHz, $CDCl_3$, 299 K) δ [ppm] = -108.8 – -109.5 (m, 1F, F10^a), -109.7 – -110.5 (m, 1F, F10^b), -196.5 – -196.9 (m, 1F, F11). **^{19}F{^{1}H} NMR** (470 MHz, $CDCl_3$, 299 K) δ [ppm] = -109.2 (dd, $^2J_{FF}$ = 253.6 Hz, $^3J_{FF}$ = 14.6 Hz, 1F, F10^a), -110.1 (dd, $^2J_{FF}$ = 253.6 Hz, $^3J_{FF}$ = 5.8 Hz, 1F, F10^b), -196.8 (dd, $^3J_{FF}$ = 14.6, 5.7 Hz, 1F, F11). **IR** (ATR) $\tilde{\nu}$ [cm^{-1}] = 2954 (w), 1713 (s), 1638 (m), 1575 (w), 1504 (w), 1437 (m), 1390 (w), 1325 (m), 1277 (m), 1230 (m), 1208 (s), 1193 (m), 1173 (s), 1108 (s), 1078 (s), 1056 (s), 983 (m), 860 (w), 815 (m), 721(w). **HRMS** (ESI) *m/z* $[M+Na]^+$, calcd. for $C_{14}H_{13}O_2F_3Na^+$: 293.0760, found: 293.0761.

2,2,3-Trifluoro-1-phenyl-1,2,3,4-tetrahydronaphthalene (21)

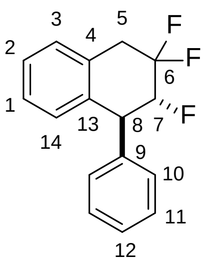

The reaction was performed according to **general procedure D** using 2-fluoro-1-methylene-3-phenyl-2,3-dihydro-1*H*-indene (**S29**) (45 mg, 0.2 mmol, 1.0 eq.). Preparative thin layer chromatography purification (*n*-pentane:Et_2O 20:1) afforded product **21** as a pale yellow solid (41 mg, 0.14 mmol, 72%; ^{19}F NMR yield: 72%).

R_f (*n*-pentane:EtOAc 20:1) = 0.68. **M.p.** 88 – 90 °C. **^{1}H NMR** (599 MHz, $CDCl_3$, 299 K) δ [ppm] = 7.39 – 7.35 (m, 2H, H11), 7.34 – 7.30 (m, 1H, H12), 7.27 – 7.22 (m, 1H, H2), 7.22 – 7.19 (m, 2H, H10), 7.18 – 7.14 (m, 2H, H1, H3), 6.86 (d, *J* = 7.8 Hz, 1H, H14), 4.91 (dddd, *J* = 48.5, 17.8, 8.5, 2.0 Hz, 1H, H7), 4.54 (dd, *J* = 18.7, 8.5 Hz, 1H, H8), 3.59 – 3.40 (m, 2H, H5). **^{13}C NMR** (151 MHz, $CDCl_3$, 299 K) δ [ppm] = 140.4 (dd, $^3J_{FC}$ = 2.6 Hz, $^4J_{FC}$ = 1.2 Hz, 1C, C9), 135.0 (dd, $^3J_{FC}$ = 7.3 Hz, $^4J_{FC}$ = 1.5 Hz, 1C, C13), 130.5 (ddd, $^3J_{FC}$ = 8.3, 2.6 Hz, $^4J_{FC}$ = 1.4 Hz, 1C, C4), 130.1 (d, $^4J_{FC}$ = 1.8 Hz, 1C, C14), 129.6

(2C, C10), 128.9 (2C, C11), 128.8 (d, $^{4}J_{FC}$ = 1.2 Hz, 1C, C3), 127.7 (1C, C12), 127.5 (1C, C2), 127.5 (d, $^{5}J_{FC}$ = 1.1 Hz, 1C, C1), 119.4 (td, $^{1}J_{FC}$ = 244.7 Hz, $^{2}J_{FC}$ = 18.8 Hz, 1C, C6), 91.9 (ddd, $^{1}J_{FC}$ = 190.1 Hz, $^{2}J_{FC}$ = 23.4, 22.4 Hz, 1C, C7), 50.8 (dt, $^{2}J_{FC}$ = 21.0 Hz, $^{3}J_{FC}$ = 3.2 Hz, 1C, C8), 37.1 (ddd, $^{2}J_{FC}$ = 25.7, 23.9 Hz, $^{3}J_{FC}$ = 2.0 Hz, 1C, C5). **^{19}F NMR** (564 MHz, $CDCl_3$, 299 K) δ [ppm] = -108.0 – -110.2 (m, 1F, F6^{a}), -115.2 – -117.1 (m, 1F, F6^{b}), -199.1 – -201.3 (m, 1F, F7). **^{19}F{^{1}H} NMR** (564 MHz, $CDCl_3$, 299 K) δ [ppm] = -108.9 (dd, $^{2}J_{FF}$ = 243.2 Hz, $^{3}J_{FF}$ = 12.1 Hz, 1F, F6^{a}), -116.2 (dd, $^{2}J_{FF}$ = 243.2 Hz, $^{3}J_{FF}$ = 15.0 Hz), -200.4 (dd, $^{3}J_{FF}$ = 15.0, 12.1 Hz, 1F, F7). **IR** (ATR) $\tilde{\nu}$ [cm^{-1}] = 3030 (w), 2918 (w), 1496 (w), 1455 (w), 1352 (w), 1297 (w), 1132 (m), 1068 (s), 1043 (m), 903 (w), 886 (w), 856 (w), 748 (s), 700 (s), 676 (m). **GC-MS** (EI) *m/z* [M]$^{+}$, calcd. for $C_{16}H_{13}F_3^{+}$: 262.0964, found: 262.0966.

2,2,3-Trifluoro-1,1-dimethyl-1,2,3,4-tetrahydronaphthalene (22)

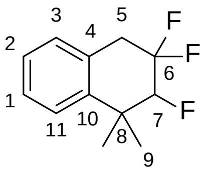

The reaction was performed according to **general procedure D** using 2-fluoro-1-methylene-3,3-dimethyl-2,3-dihydro-1*H*-indene (**S31**) (35 mg, 0.2 mmol, 1.0 eq.). Column chromatography purification (*n*-pentane) afforded product **22** as a colourless oil (19 mg, 0.09 mmol, 43%; ^{19}F NMR yield: 51%).

R$_f$ (*n*-pentane) = 0.34. **^{1}H NMR** (500 MHz, $CDCl_3$, 299 K) δ [ppm] = 7.35 (dd, $^{3}J_{HH}$ = 7.9 Hz, $^{4}J_{HH}$ = 1.4 Hz, 1H, H11), 7.29 (ddd, $^{3}J_{HH}$ = 7.6, 7.4, $^{4}J_{HH}$ = 1.4 Hz, 1H, H1), 7.20 (ddd, $^{3}J_{HH}$ = 7.8, 7.4 Hz, $^{4}J_{HH}$ = 1.4 Hz, 1H, H2), 7.08 (dd, $^{3}J_{HH}$ = 7.6 Hz, $^{4}J_{HH}$ = 1.3 Hz, 1H, H3), 4.52 (dddd, *J* = 47.9, 10.2, 4.3, 1.2 Hz, 1H, H7), 3.58 – 3.44 (m, 1H, H5^{a}), 3.32 – 3.22 (m, 1H, H5^{b}), 1.47 (dd, *J* = 3.9, 1.2 Hz, 3H, H9^{a}), 1.40 (s, 3H, H9^{b}). **^{13}C NMR** (126 MHz, $CDCl_3$, 299 K) δ [ppm] = 140.8 (1C, C10), 129.1 (d, $^{4}J_{FC}$ = 1.2 Hz, 1C, C3), 128.9 (dd, $^{3}J_{FC}$ = 6.9, 3.9 Hz, 1C, C4), 127.8 (1C, C1), 127.0 (1C, C2), 126.8 (1C, C11), 119.9 (ddd, $^{1}J_{FC}$ = 246.5, 242.4 Hz, $^{2}J_{FC}$ = 25.6 Hz, 1C, C6), 93.6 (ddd, $^{1}J_{FC}$ = 184.3 Hz, $^{2}J_{FC}$ = 29.0, 23.6 Hz, 1C, C7), 40.5 (dd, $^{2}J_{FC}$ = 19.1 Hz, $^{3}J_{FC}$ = 2.5 Hz, 1C, C8), 35.7 (ddd, $^{2}J_{FC}$ = 24.9, 25.1 Hz, $^{3}J_{FC}$ = 1.6 Hz, 1C, C5), 29.3 – 28.9 (m, 1C, C9^{b}), 25.8 (dd, $^{3}J_{FC}$ = 10.8 Hz, $^{4}J_{FC}$ = 2.6 Hz, 1C, C9^{a}). **^{19}F NMR** (470 MHz, $CDCl_3$, 299 K) δ [ppm] = -104.3 – -105.0 (m, 1F, F6^{a}), -105.0 – -105.7 (m, 1F, F6^{b}), -199.6 – -199.9 (m, 1F, F7). **^{19}F{^{1}H} NMR** (470 MHz, $CDCl_3$, 299 K) δ [ppm] = -104.7 (dd, $^{2}J_{FF}$ = 253.9 Hz, $^{3}J_{FF}$ = 13.0 Hz, 1F, F6^{a}), -105.4 (dd, $^{2}J_{FF}$ = 253.9 Hz, $^{3}J_{FF}$ = 7.1 Hz, 1F, F6^{b}), -199.7 (dd, $^{3}J_{FF}$ = 13.0, 6.4 Hz, 1F, F7). **IR** (ATR)

$\tilde{\nu}$ [cm^{-1}] = 2977 (w), 1492 (w), 1451 (w), 1380 (m), 1327 (w), 1292 (w), 1198 (w), 1101 (m), 1072 (s), 1052 (s), 1009 (w), 837 (w), 760 (s), 721 (m). 674 (w). **GC-MS** (EI) *m/z* [M]$^+$, calcd. for $C_{12}H_8F_4^+$: 214.0964, found: 214.0965.

2,2,3,3-Tetrafluoro-1,2,3,4-tetrahydronaphthalene (23)

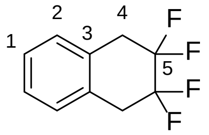

The reaction was performed according to **general procedure D** using 2,2-difluoro-1-methylene-2,3-dihydro-1*H*-indene (**S33**) (33 mg, 0.2 mmol, 1.0 eq.). Column chromatography purification (*n*-pentane) afforded product **23** as white solid (23 mg, 0.11 mmol, 57%; ^{19}F NMR yield: 83%).

R$_f$ (*n*-pentane) = 0.51. **M.p.** 72 – 73 °C. **^{1}H NMR** (599 MHz, $CDCl_3$, 299 K) δ [ppm] = 7.25 (dd, $^3J_{HH}$ = 5.6 Hz, $^4J_{HH}$ = 3.5 Hz, 2H, H1), 7.16 (dd, $^3J_{HH}$ = 5.6 Hz, $^4J_{HH}$ = 3.5 Hz, 2H, H2), 3.50 (tt, $^3J_{FH}$ = 11.2 Hz, $^4J_{FH}$ = 6.5 Hz, 4H, H4). **^{13}C NMR** (151 MHz, $CDCl_3$, 299 K) δ [ppm] = 129.4 – 129.3 (m, 2C, C3), 128.9 (2C, C2), 127.4 (2C, C1), 116.2 (tt, $^1J_{FC}$ = 251.4 Hz, $^2J_{FC}$ = 30.8 Hz, 2C, C5), 36.5 (tt, $^2J_{FC}$ = 25.3 Hz, $^3J_{FC}$ = 8.6 Hz, 2C, C4). **^{19}F NMR** (564 MHz, $CDCl_3$, 299 K) δ [ppm] = -120.2 (tt, $^3J_{FH}$ = 11.3 Hz, $^4J_{FH}$ = 7.1 Hz, 4F, F5). **^{19}F{^{1}H} NMR** (564 MHz, $CDCl_3$, 299 K) δ [ppm] = -120.2 (s, 4F, F5). **IR** (ATR) $\tilde{\nu}$ [cm^{-1}] = 2960 (w), 1500 (w), 1459 (w), 1421 (m), 1372 (w), 1239 (m), 1205 (m), 1146 (m), 1125 (s), 1083 (s), 1034 (s), 892 (m), 782 (w), 750 (s), 690 (s). **GC-MS** (EI) *m/z* [M]$^+$, calcd. for $C_{10}H_8F_4^+$: 204.0557, found: 204.0554.

3-Chloro-2,2-difluoro-1,2,3,4-tetrahydronaphthalene (24)

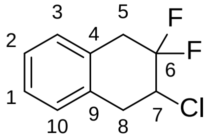

The reaction was performed according to **general procedure D** using 2-chloro-1-methylene-2,3-dihydro-1*H*-indene (**S35**) (33 mg, 0.2 mmol, 1.0 eq.). Column chromatography purification (*n*-pentane) afforded product **24** as pale yellow oil (15 mg, 0.07 mmol, 35%; ^{19}F NMR yield: 35%).

R$_f$ (*n*-pentane) = 0.39. **^{1}H NMR** (599 MHz, $CDCl_3$, 299 K) δ [ppm] = 7.25 – 7.20 (m, 2H, H1, H2), 7.16 – 7.13 (m, 1H, H10), 7.13 – 7.10 (m, 1H, H3), 4.43 – 4.38 (m, 1H, H7), 3.64 – 3.49 (m, 2H, H5^a, H8^a), 3.35 – 3.23 (m, 2H, H5^b, H8^b). **^{13}C NMR** (151 MHz, $CDCl_3$, 299 K) δ [ppm] = 130.9 (1C, C9), 130.5 (dd,

$^{3}J_{FC}$ = 6.6, 4.1 Hz, 1C, C4), 129.1 (1C, C3), 128.9 (1C, C10), 127.3 (1C, C2), 127.2 (1C, C1), 120.6 (dd, $^{1}J_{FC}$ = 247.9, 241.6 Hz, 1C, C6), 55.5 (dd, $^{2}J_{FC}$ = 30.2, 24.5 Hz, 1C, C7), 37.2 (dd, $^{3}J_{FC}$ = 3.9, 2.5 Hz, 1C, C8), 35.8 (dd, $^{2}J_{FC}$ = 25.8, 25.4 Hz, 1C, C5). **^{19}F NMR** (564 MHz, $CDCl_3$, 299 K) δ [ppm] = -100.9 (dddt, $^{2}J_{FF}$ = 239.8 Hz, J = 22.4, 13.6, 3.3 Hz, 1F, F6[a]), -107.2 (dtdd, $^{2}J_{FF}$ = 239.7 Hz, J = 13.7, 10.7, 2.8 Hz, 1F, F6[b]). **^{19}F{^{1}H} NMR** (564 MHz, $CDCl_3$, 299 K) δ [ppm] = -100.9 (d, $^{2}J_{FF}$ = 239.9 Hz, 1F, F6[a]), -107.2 (d, $^{2}J_{FF}$ = 239.7 Hz, 1F, F6[b]). **IR** (ATR) $\tilde{\nu}$ [cm^{-1}] = 2942 (w), 1497 (w), 1426 (w), 1372 (w), 1279 (m), 1166 (m), 1128 (m), 1091 (m), 1068 (s), 976 (w), 917 (w), 744 (s), 666 (w). **GC-MS** (EI) *m/z* [M]$^{+}$, calcd. for $C_{10}H_9ClF^{+}$: 202.0356, found: 202.0355.

Methyl (*S*)-2,3,3,-trifluoro-1,2,3,4-tetrahydronaphthalene-2-carboxylate (28)

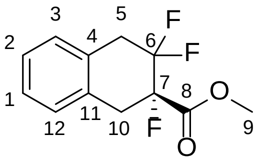

The reaction was performed according to **general procedure D** using methyl (*R*)-2-fluoro-1-methylene-2,3-dihydro-1*H*-indene-2-carboxylate **S40** (41 mg, 0.2 mmol, 1.0 eq.). Column chromatography purification (*n*-pentane:EtOAc 100:1) afforded product **31** as a colourless oil (21 mg, 0.09 mmol, 44%; ^{19}F NMR yield: 57%, 75:25 e.r., 100% e.s.). The e.r. of the product was determined by HPLC analysis using a Reporsil Chiral-OM column (10 μm, 250 mm x 4.6 mm) as the stationary phase and hexane:^{i}PrOH (98:2) as eluent system at a flow rate of 1.0 mL/min. Detection took place at λ = 210 nm with t_R(major) = 7.32 min; t_R(minor) = 8.04 min. $[\alpha]_D^{25}$ = -13.7° (c = 1.00, $CHCl_3$). **R$_f$** (*n*-pentane:EtOAc 10:1) = 0.53. **^{1}H NMR** (599 MHz, $CDCl_3$, 299 K) δ [ppm] = 7.25 – 7.21 (m, 2H, H1, H2), 7.16 – 7.11 (m, 2H, H3, H12), 3.92 (s, 3H, H9), 3.76 (ddd, J = 38.4 Hz, 17.8, 4.8 Hz, 1H, H10[a]), 3.56 (dddd, J = 31.4, 17.3, 8.8, 5.1 Hz, 1H, H5[a]), 3.42 – 3.32 (m, 2H, H5[b], H10[b]). **^{13}C NMR** (151 MHz, $CDCl_3$, 299 K) δ [ppm] = 166.7 (ddd, $^{2}J_{FC}$ = 25.4 Hz, $^{3}J_{FC}$ = 1.9, 2.2 Hz, 1C, C8), 130.0 (d, $^{3}J_{FC}$ = 9.9 Hz,1C, C4), 129.5 (d, $^{3}J_{FC}$ = 1.3 Hz, 1C, C11), 129.1 (d, $^{4}J_{FC}$ = 1.6 Hz, 1C, C3), 128.9 (d, $^{4}J_{FC}$ = 0.9 Hz, 1C, C12), 127.5 (1C, C1/C2), 127.2 (1C, C1/C2), 119.2 (ddd, $^{1}J_{FC}$ = 253.2, 247.1 Hz, $^{2}J_{FC}$ = 29.7 Hz, 1C, C6), 92.0 (ddd, $^{1}J_{FC}$ = 193.9, $^{2}J_{FC}$ = 30.8, 24.8 Hz, 1C, C7), 53.6 (1C, C9), 36.8 (ddd, $^{2}J_{FC}$ = 22.1, $^{3}J_{FC}$ = 4.0, 1.3 Hz, 1C, C10), 35.5 (ddd, $^{2}J_{FC}$ = 24.7, 25.0 Hz, $^{3}J_{FC}$ = 1.7 Hz, 1C, C5). **^{19}F NMR** (564 MHz, $CDCl_3$, 299 K) δ [ppm] = -109.01 – -109.60 (m, 1F, F6[a]), -111.20 – -111.74 (m, 1F, F6[b]), -172.35 – -172.51 (m, 1F, F7). **^{19}F{^{1}H} NMR** (564 MHz, $CDCl_3$,

299 K) δ [ppm] = -109.30 (dd, $^2J_{FF}$ = 251.2 Hz, $^3J_{FF}$ = 5.5 Hz), -111.47 (dd, $^2J_{FF}$ = 251.1 Hz, $^3J_{FF}$ = 14.9 Hz), -172.43 (dd, $^3J_{FF}$ = 14.9, 5.4 Hz). **IR** (ATR) $\tilde{\upsilon}$ [cm^{-1}] = 2959 (w), 1751 (s),1498 (w), 1458 (w), 1438 (m), 1297 (m), 1256 (m), 1205 (m), 1126 (m), 1083 (s), 1068 (s), 1017 (m), 953 (w), 881 (w), 744 (m), 691 (w).

Ethyl (*S*)-2,3,3,-trifluoro-1,2,3,4-tetrahydronaphthalene-2-carboxylate (29)

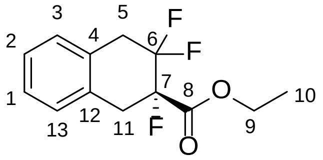

The reaction was performed according to **general procedure D** using ethyl (*R*)-2-fluoro 1-methylene-2,3-dihydro-1*H*-indene-2-carboxylate (**S43**) (44 mg, 0.2 mmol, 1.0 eq.). Column chromatography purification (*n*-pentane:EtOAc 100:1) afforded product **29** as colourless oil (20 mg, 0.08 mmol, 40%; ^{19}F NMR yield: 45%, 84:16 e.r. 100% e.s.).

The e.r. of the product was determined by HPLC analysis using a Reporsil Chiral-OM column (10 μm, 250 mm x 4.6 mm) as the stationary phase and hexane:^{i}PrOH (98:2) as eluent system at a flow rate of 1.0 mL/min. Detection took place at λ = 210 nm with t_R(major) = 6.29 min; t_R(minor) = 6.76 min. $[\alpha]_D^{25}$ = -7.5° (*c* = 0.50, $CHCl_3$). **R_f** (*n*-pentane/EtOAc 20:1) = 0.48. **^{1}H NMR** (500 MHz, $CDCl_3$, 299 K) δ [ppm] = 7.25 – 7.21 (m, 2H, H1, H2), 7.16 – 7.11 (m, 2H, H3, H13), 4.38 (qd, $^3J_{HH}$ = 7.1 Hz, *J* = 1.4 Hz, 2H, H9), 3.75 (ddd, *J* = 38.9, 18.0, 4.3 Hz, 1H, H11^a), 3.55 (ddddd, *J* = 17.9, 17.5, 13.7, 8.4, 4.2 Hz, 1H, H5^a), 3.43 – 3.29 (m, 2H, H5^b, H11^b), 1.37 (t, $^3J_{HH}$ = 7.1 Hz, 3H, H10). **^{13}C NMR** (126 MHz, $CDCl_3$, 299 K) δ [ppm] = 166.2 (d, $^2J_{FC}$ = 25.3 Hz, 1C, C8), 130.1 (d, $^4J_{FC}$ = 10.5 Hz, 1C, C12), 129.7 (d, $^3J_{FC}$ = 1.4 Hz, 1C, C4), 129.1 (d, $^4J_{Fc}$ = 1.6 Hz, 1C, C3), 128.9 (d, $^3J_{FC}$ = 1.0 Hz, 1C, C13), 127.4 (1C, C1/C2), 127.2 (d, $^5J_{FC}$ = 0.8 Hz, 1C, C1/C2), 119.2 (ddd, $^1J_{FC}$ = 253.1, 247.0 Hz, $^2J_{FC}$ = 29.5 Hz, 1C, C6), 91.8 (ddd, $^1J_{FC}$ = 193.8 Hz, $^2J_{FC}$ = 31.2, 24.7 Hz, 1C, C7), 62.9 (1C, C9), 36.7 (ddd, $^2J_{FC}$ = 22.1 Hz, $^3J_{FC}$ = 4.0, 1.3 Hz, 1C, C11), 35.5 (td, $^2J_{FC}$ = 24.7 Hz, $^3J_{FC}$ = 1.7 Hz, 1C, C5), 14.2 (1C, C10). **^{19}F NMR** (470 MHz, $CDCl_3$, 299 K) δ [ppm] = -108.8 – -110.0 (m, 1F, F6^a), -111.0 – -112.6 (m, 1F, F6^b), -172.1 – -172.8 (m, 1F, F7). **^{19}F{^{1}H} NMR** (470 MHz, $CDCl_3$, 299 K) δ [ppm] = -109.2 (dd, $^2J_{FF}$ = 251.1 Hz, $^3J_{FF}$ = 5.6 Hz), -111.6 (dd, $^2J_{FF}$ = 251.1 Hz, $^3J_{FF}$ = 14.9 Hz), -172.4 (dd, $^3J_{FF}$ = 14.8, 5.6Hz). **IR** (ATR) $\tilde{\upsilon}$ [cm^{-1}] = 2985 (w), 1759 (m),

1498 (w), 1458 (w), 1296 (m), 1254 (m), 1206 (w), 1126 (m), 1084 (s), 1068 (s), 1022 (m), 885 (w), 859 (w), 745 (m), 691 (w). **HRMS** (ESI) *m/z* $[M+Na]^+$, calcd. for $C_{13}H_{13}O_2F_3Na^+$: 281.0760, found: 281.0759.

Ethyl (*S*)-7-bromo-2,3,3,-trifluoro-1,2,3,4-tetrahydronaphthalene-2-carboxylate (30)

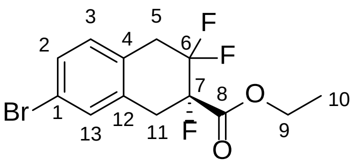

The reaction was performed according to **general procedure D** using methyl ethyl (*R*)-5-bromo-2-fluoro 1-methylene-2,3-dihydro-1*H*-indene-2-carboxylate (**S46**) (60 mg, 0.2 mmol, 1.0 eq.). Column chromatography purification (*n*-pentane:EtOAc 100:1) afforded product **30** as colourless oil (35 mg, 0.10 mmol, 50%; ^{19}F NMR yield: 53%, 86:14 e.r., 100% e.s.).

The e.r. of the product was determined by HPLC analysis using a Reporsil Chiral-NR column (10 *μ*m, 250 mm x 4.6 mm) as the stationary phase and hexane:^{i}PrOH (99.8:0.2) as eluent system at a flow rate of 1.0 mL/min. Detection took place at λ = 210 nm with t_R(minor) = 15.01 min; t_R(major) = 17.06 min. $[\alpha]_D^{25}$ = -13.2° (*c* = 1.00, $CHCl_3$). **R$_f$** (*n*-pentane:EtOAc 20:1) = 0.48. **^{1}H NMR** (599 MHz, $CDCl_3$, 299 K) δ [ppm] = 7.36 (dd, $^3J_{HH}$ = 8.2 Hz, $^4J_{HH}$ = 2.1 Hz, 1H, H2), 7.30 (d, $^4J_{HH}$ = 2.0 Hz, 1H, H13), 7.01 (d, $^3J_{HH}$ = 8.2 Hz, 1H, H3), 4.38 (qd, $^3J_{HH}$ = 7.1 Hz, *J* = 2.0 Hz, 2H, H9), 3.78 – 3.65 (m, 1H, H11^a), 3.53 – 3.40 (m, 1H, H5^a), 3.32 (m, 2H, H5^b, H11^b), 1.36 (t, *J* = 7.1 Hz, 3H, H10). **^{13}C NMR** (151 MHz, $CDCl_3$, 299 K) δ [ppm] = 165.7 (d, $^2J_{FC}$ = 25.1 Hz, 1C, C8), 131.8 – 131.7 (m, 1C, C4), 131.6 (d, $^4J_{FC}$ = 1.0 Hz, 1C, C13), 130.4 (d, $^4J_{FC}$ = 1.7 Hz, 1C, C3), 130.3 (1C, C2), 128.9 (d, $^3J_{FC}$ = 9.5 Hz, 1C, C12), 120.9 (1C, C1), 118.7 (ddd, $^1J_{FC}$ = 253.5, 247.4 Hz, $^2J_{FC}$ = 29.1 Hz, 1C, C6), 92.3 – 90.3 (m, 1C, C7), 62.9 (1C, C9), 36.2 (ddd, $^2J_{FC}$ = 22.5 Hz, $^3J_{FC}$ = 4.0, 1.3 Hz, 1C, C11), 34.9 (td, $^2J_{FC}$ = 24.7 Hz, $^3J_{FC}$ = 1.4 Hz, 1C, C5), 14.0 (1C, C10). **^{19}F NMR** (564 MHz, $CDCl_3$, 299 K) δ [ppm] = -109.0 – -109.6 (m, 1F, F6^a), -111.5 – -112.2 (m, 1F, F6^b), -172.3 – -172.5 (m, 1F, F7). **^{19}F{^{1}H} NMR** (564 MHz, $CDCl_3$, 299 K) δ [ppm] = -109.3 (dd, $^2J_{FF}$ = 252.1 Hz, $^3J_{FF}$ = 5.4 Hz, 1F, F6^a), -111.9 (dd, $^2J_{FF}$ = 251.9 Hz, $^3J_{FF}$ = 14.9 Hz, 1F, F6^b), -172.4 (dd, $^3J_{FF}$ = 14.9, 5.3 Hz, 1F, F7). **IR** (ATR) $\tilde{\nu}$ [cm^{-1}] = 2987 (w), 1759 (m), 1597 (w), 1422 (w), 1309 (m), 1285 (m), 1252 (m), 1180 (m), 1203 (w), 1126 (m), 1090 (m), 1070 (s), 1023 (m), 889 (w), 808 (w), 789 (w). **HRMS** (ESI) *m/z* $[M+Na]^+$, calcd. for $C_{13}H_{12}O_2BrF_3Na^+$: 358.9865, found: 358.9866.

Ethyl (*S*)-7-chloro-2,3,3,-trifluoro-1,2,3,4-tetrahydronaphthalene-2-carboxylate (31)

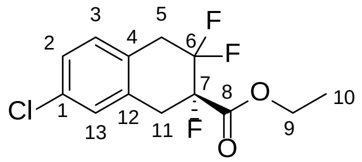

The reaction was performed according to **general procedure D** using ethyl (*R*)-5-chloro-2-fluoro 1-methylene-2,3-dihydro-1*H*-indene-2-carboxylate (**S49**) (48 mg, 0.2 mmol, 1.0 eq.). Column chromatography purification (*n*-pentane:EtOAc 100:1) afforded product **31** as colourless oil (26 mg, 0.09 mmol, 45%; ^{19}F NMR yield: 51%, 86:14 e.r., 100% e.s.).

The e.r. of the product was determined by HPLC analysis using a Reporsil Chiral-NR column (10 μm, 250 mm x 4.6 mm) as the stationary phase and hexane:^{i}PrOH (99.8:0.2) as eluent system at a flow rate of 1.0 mL/min. Detection took place at λ = 210 nm with t_R(minor) = 13.64 min; t_R(major) = 15.48 min. $[\alpha]_D^{25}$ = -7.8° (*c* = 0.50, $CHCl_3$). **R$_f$** (*n*-pentane:EtOAc 20:1) = 0.48. **^{1}H NMR** (599 MHz, $CDCl_3$, 299 K) δ [ppm] = 7.21 (dd, $^3J_{HH}$ = 8.3 Hz, $^4J_{HH}$ = 2.2 Hz, 1H, H2), 7.14 (d, $^4J_{HH}$ = 2.2 Hz, 1H, H13), 7.07 (d, $^3J_{HH}$ = 8.2 Hz, 1H, H3), 4.38 (qd, $^3J_{HH}$ = 7.1 Hz, J = 1.8 Hz, 2H, H9), 3.72 (ddd, J = 37.5, 17.8, 4.0 Hz, 1H, H11^a), 3.50 (dddd, J = 30.9, 17.2, 8.8, 5.1 Hz, 1H, H5^a), 3.38 – 3.28 (m, 2H, H5^b, H11^b), 1.37 (t, $^3J_{HH}$ = 7.1 Hz, 3H, H10). **^{13}C NMR** (151 MHz, $CDCl_3$, 299 K) δ [ppm] = 166.0 ($^2J_{FC}$ = 26.6 Hz, 1C, C8), 133.2 (1C, C1), 131.6 (1C, C12), 130.3 (d, $^4J_{FC}$ = 1.7 Hz, 1C, C3), 128.8 (d, $^4J_{FC}$ = 1.1 Hz, 1C, C13), 128.6 (d, $^3J_{FC}$ = 10.5 Hz, 1C, C4), 127.5 (1C, C2), 119.0 (ddd, $^1J_{FC}$ = 254.6, 247.2 Hz, $^2J_{FC}$ = 29.4 Hz, 1C, C6), 92.0 (ddd, $^1J_{FC}$ = 195.2 Hz, $^2J_{FC}$ = 30.9, 24.2 Hz, 1C, C7), 63.1 (1C, C9), 36.5 (dd, $^2J_{FC}$ = 22.8 Hz, $^3J_{FC}$ = 4.5 Hz, 1C, C11), 35.0 (ddd, $^2J_{FC}$ = 26.1, 25.9 Hz, $^3J_{FC}$ = 1.9 Hz, 1C, C5), 14.2 (1C, C10). **^{19}F NMR** (564 MHz, $CDCl_3$, 299 K) δ [ppm] = -109.0 – -109.6 (m, 1H, F6^a), -111.6 – -112.2 (m, 1F, F6^b), -172.3 – -172.5 (m, 1F, F7). **^{19}F{^{1}H} NMR** (564 MHz, $CDCl_3$, 299 K) δ [ppm] = -109.3 (dd, $^2J_{FF}$ = 251.9 Hz, $^3J_{FF}$ = 5.4 Hz, 1F, F6^a), -111.9 (dd, $^2J_{FF}$ = 252.0 Hz, $^2J_{FF}$ = 14.9 Hz, 1F, F6^b), -172.4 (dd, $^3J_{FF}$ = 14.8, 5.4 Hz, 1F, F7). **IR** (ATR) $\tilde{\upsilon}$ [cm^{-1}] = 2986 (w), 1745 (m), 1601 (w), 1489 (w), 1371 (w), 1301 (w), 1283 (m), 1251 (m), 1164 (m), 1127 (m), 1085 (s), 1068 (s), 1022 (m), 902 (w), 859 (w), 811 (w). **HRMS** (ESI) *m/z* [M+Na]$^+$, calcd. for $C_{13}H_{12}O_2ClF_3Na^+$: 315.0370, found: 315.0370.

Ethyl (*S*)-2,3,3,-trifluoro-6-(trifluoromethyl)-1,2,3,4-tetrahydronaphthalene-2-carboxylate (32)

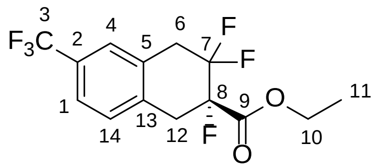

The reaction was performed according to **general procedure D** using ethyl (*R*)-2-fluoro 1-methylene-6-(trifluoromethyl)-2,3-dihydro-1*H*-indene-2-carboxylate (**S52**) (58 mg, 0.2 mmol, 1.0 eq.). Column chromatography purification (*n*-pentane:CH_2Cl_2 10:1) afforded product **32** as colourless oil (29 mg, 0.09 mmol, 45%; ^{19}F NMR yield: 49%, 88:12 e.r., 100% e.s.).

The e.r. of the product was determined by HPLC analysis using a Reporsil Chiral-NR column (10 μm, 250 mm x 4.6 mm) as the stationary phase and hexane:^{i}PrOH (99.9:0.1) as eluent system at a flow rate of 1.0 mL/min. Detection took place at λ = 210 nm with t_R(minor) = 12.34 min; t_R(minor) = 13.46 min. $[\alpha]_D^{25}$ = -8.2° (*c* = 1.00, $CHCl_3$). **R_f** (*n*-pentane:CH_2Cl_2 4:1) = 0.47. **^{1}H NMR** (599MHz, $CDCl_3$, 299 K) δ [ppm] = 7.49 (d, $^3J_{HH}$ = 8.0 Hz, 1H, H1), 7.41 (s, 1H, H4), 7.27 (d, $^3J_{HH}$ = 8.1 Hz, 1H, H14), 4.39 (qd, $^3J_{HH}$ = 7.1 Hz, J = 1.9 Hz, 2H, H10), 3.78 (ddd, J = 37.1, 18.1, 3.3 Hz, 1H, H12^a), 3.65 – 3.53 (m, 1H, H6^a), 3.48 – 3.37 (m, 2H, H6^b, H12^b), 1.37 (t, $^3J_{HH}$ = 7.1 Hz, 3H, H11). **^{13}C NMR** (151 MHz, $CDCl_3$, 299 K) δ [ppm] = 165.8 (dt, $^2J_{FC}$ = 25.1 Hz, $^3J_{FC}$ = 2.0 Hz, 1C, C9), 133.9 (d, $^3J_{FC}$ = 1.1 Hz, 1C, C13), 131.0 (d, $^3J_{FC}$ = 10.2 Hz, 1C, C5), 129.9 (q, $^2J_{FC}$ = 32.5 Hz, 1C, C2), 129.5 (1C, C14), 126.0 (qd, $^3J_{FC}$ = 3.8 Hz, $^4J_{FC}$ = 1.7 Hz, 1C, C4), 124.3 (q, $^3J_{FC}$ = 3.7 Hz, 1C, C1), 123.9 (q, $^1J_{FC}$ = 272.1 Hz, 1C, C3), 118.8 (ddd, $^1J_{FC}$ = 253.8, 247.2 Hz, $^2J_{FC}$ = 29.5, 1C, C7), 91.4 (ddd, $^1J_{FC}$ = 194.6 Hz, $^2J_{FC}$ = 30.7, 24.9 Hz, 1C, C8), 63.2 (1C, C10), 36.7 (dd, $^2J_{FC}$ = 22.9 Hz, $^3J_{FC}$ = 4.0 Hz, 1C, C12), 35.5 (td, $^2J_{FC}$ = 25.4 Hz, $^3J_{FC}$ = 1.5 Hz, 1C, C6), 14.2 (1C, C11). **^{19}F NMR** (564 MHz, $CDCl_3$, 299 K) δ [ppm] = -62.8 (s, 3F, F3), -108.9 – -109.5 (m, 1F, F7^a), -111.8 – -112.3 (m, 1F, F7^b), -172.1 – -172.4 (m, 1F, F8). **^{19}F{^{1}H} NMR** (564 MHz, $CDCl_3$, 299 K) δ [ppm] = -62.8 (s, 3F, F3), -109.2 (dd, $^2J_{FF}$ = 252.7 Hz, $^3J_{FF}$ = 5.2 Hz, 1F, F7^a), -112.1 (dd, ^3J = 252.7 Hz, $^3J_{FF}$ = 14.9 Hz, 1F, F7^b), -172.3 (dd, $^3J_{FF}$ = 14.9, 5.3 Hz, 1F, F8). **IR** (ATR) $\tilde{\nu}$ [cm^{-1}] = 2990 (w), 1761 (m), 1626 (w), 1434 (w), 1333 (s), 1299 (m), 1287 (m), 1201 (w), 1165 (m), 1128 (s), 1070 (s), 1024 (w), 916 (w), 871 (w), 774 (w); 698 (w). **HRMS** (ESI) *m/z* [M+Na]$^+$, calcd. for $C_{14}H_{12}O_2F_6Na^+$: 349.0634, found: 349.0634.

V. HPLC Traces

Methyl (*R*)-2-fluoro-1-oxo-2,3-dihydro-1*H*-indene-2-carboxylate (S39)

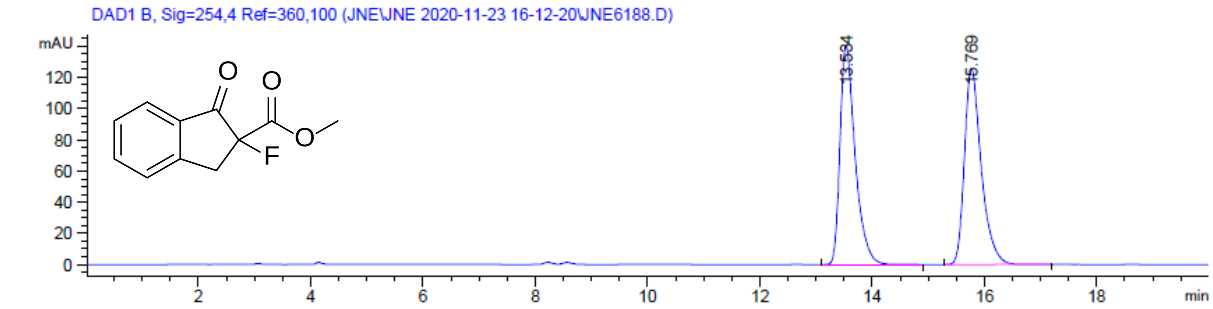

Signal 1: DAD1 B, Sig=254,4 Ref=360,100

Peak #	RetTime [min]	Type	Width [min]	Area [mAU*s]	Height [mAU]	Area %
1	13.534	BB	0.2816	2620.26294	140.50551	50.0895
2	15.769	BB	0.3124	2610.89478	125.64368	49.9105

Totals : 5231.15771 266.14919

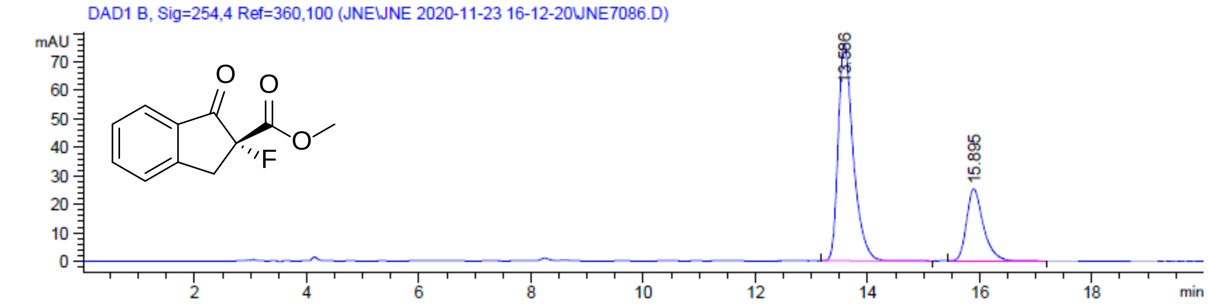

Signal 1: DAD1 B, Sig=254,4 Ref=360,100

Peak #	RetTime [min]	Type	Width [min]	Area [mAU*s]	Height [mAU]	Area %
1	13.586	BB	0.2807	1420.66736	76.48762	72.9782
2	15.895	BB	0.3144	526.03400	25.32203	27.0218

Totals : 1946.70135 101.80964

Methyl (*R*)-2-fluoro-1-methylene-2,3-dihydro-1*H*-indene-2-carboxylate (S40)

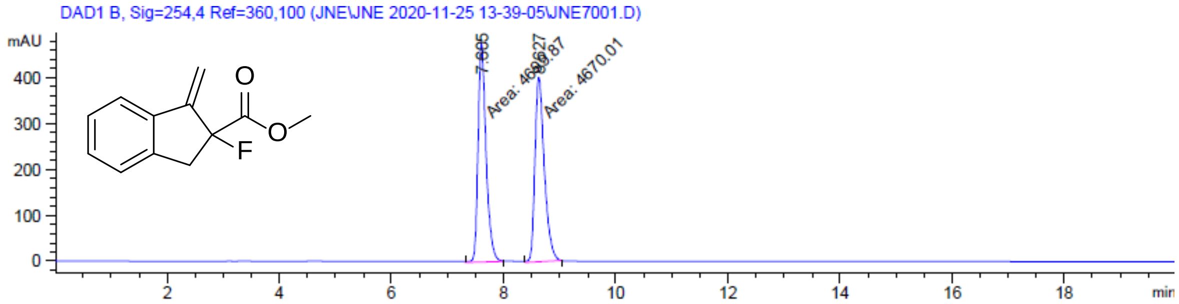

Signal 1: DAD1 B, Sig=254,4 Ref=360,100

Peak #	RetTime [min]	Type	Width [min]	Area [mAU*s]	Height [mAU]	Area %
1	7.605	MM	0.1641	4699.87402	477.21719	50.1593
2	8.627	MM	0.1935	4670.01367	402.32150	49.8407

Totals : 9369.88770 879.53870

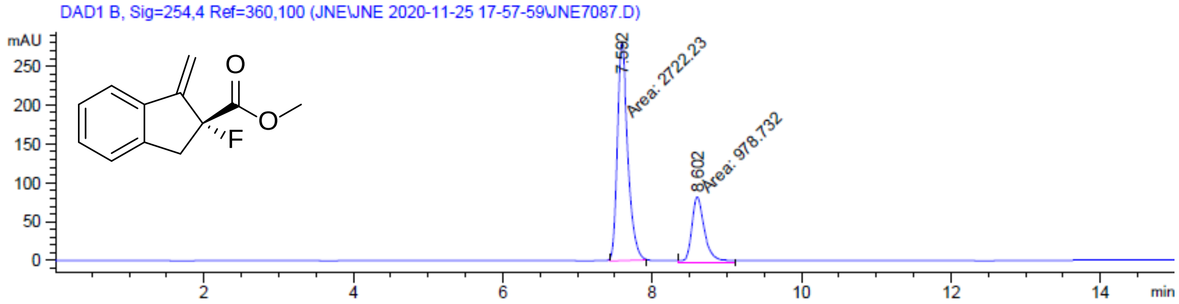

Signal 1: DAD1 B, Sig=254,4 Ref=360,100

Peak #	RetTime [min]	Type	Width [min]	Area [mAU*s]	Height [mAU]	Area %
1	7.592	MM	0.1619	2722.23242	280.31296	73.5547
2	8.602	MM	0.1954	978.73206	83.49361	26.4453

Totals : 3700.96448 363.80657

Methyl (*S*)-2,3,3,-trifluoro-1,2,3,4-tetrahydronaphthalene-2-carboxylate (28)

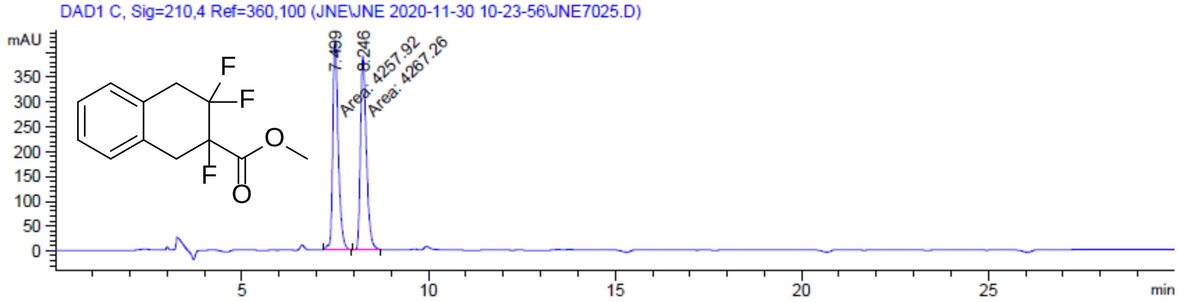

Signal 2: DAD1 C, Sig=210,4 Ref=360,100

Peak #	RetTime [min]	Type	Width [min]	Area [mAU*s]	Height [mAU]	Area %
1	7.499	MM	0.1693	4257.92285	419.23883	49.9452
2	8.246	MM	0.1819	4267.26270	390.94257	50.0548
Totals :				8525.18555	810.18140	

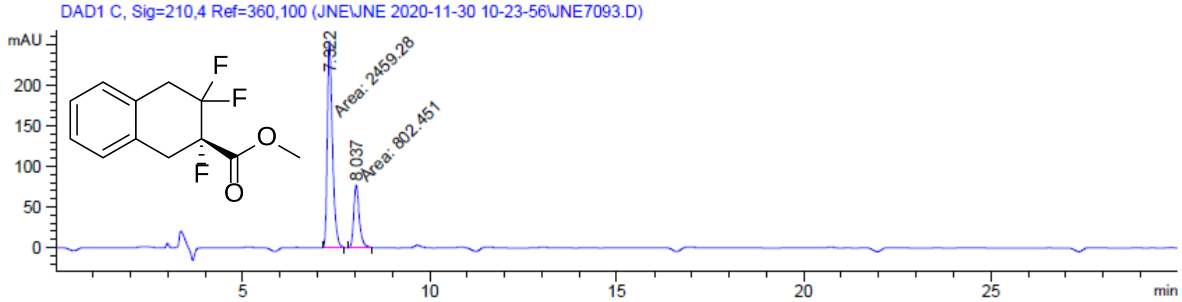

Signal 2: DAD1 C, Sig=210,4 Ref=360,100

Peak #	RetTime [min]	Type	Width [min]	Area [mAU*s]	Height [mAU]	Area %
1	7.322	MM	0.1613	2459.27930	254.05548	75.3980
2	8.037	MM	0.1726	802.45148	77.50092	24.6020
Totals :				3261.73077	331.55640	

Ethyl (*R*)-2-fluoro 1-oxo-2,3-dihydro-1*H*-indene-2-carboxylate (S42)

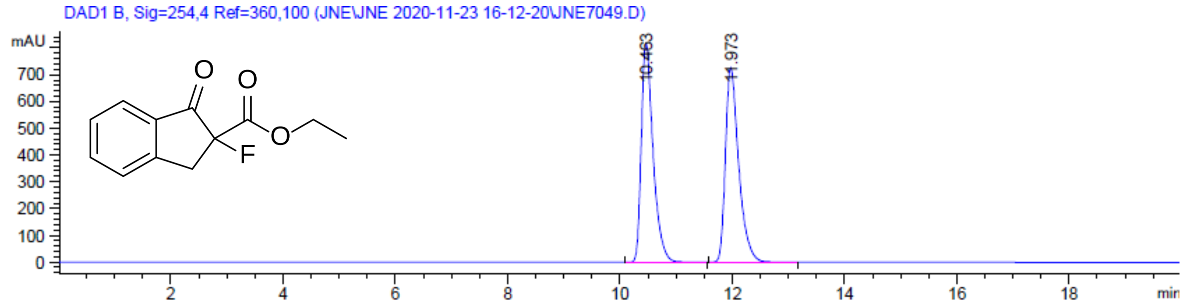

Signal 1: DAD1 B, Sig=254,4 Ref=360,100

Peak #	RetTime [min]	Type	Width [min]	Area [mAU*s]	Height [mAU]	Area %
1	10.463	BB	0.2177	1.18901e4	817.52313	50.0360
2	11.973	BB	0.2473	1.18730e4	724.13312	49.9640

Totals : 2.37631e4 1541.65625

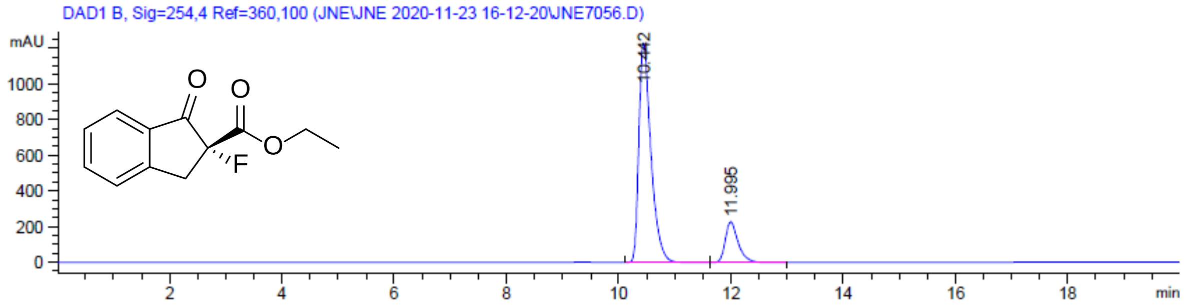

Signal 1: DAD1 B, Sig=254,4 Ref=360,100

Peak #	RetTime [min]	Type	Width [min]	Area [mAU*s]	Height [mAU]	Area %
1	10.442	BB	0.2260	1.83342e4	1229.21985	83.5801
2	11.995	BB	0.2400	3601.87720	225.89294	16.4199

Totals : 2.19361e4 1455.11279

Ethyl (*R*)-2-fluoro 1-methylene-2,3-dihydro-1*H*-indene-2-carboxylate (S43)

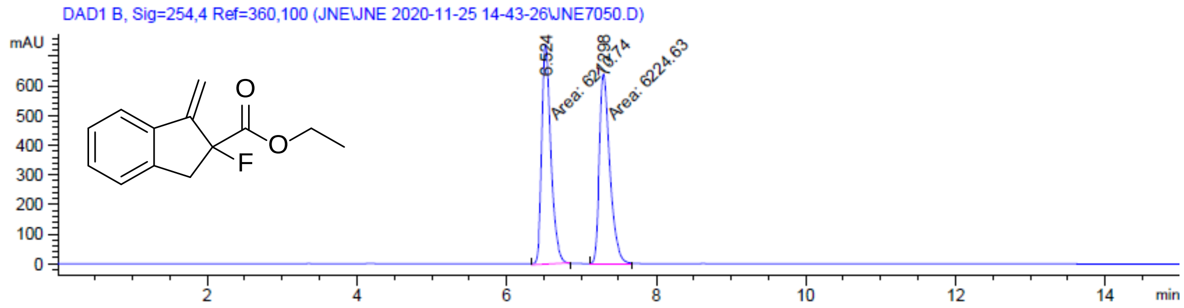

Signal 1: DAD1 B, Sig=254,4 Ref=360,100

Peak #	RetTime [min]	Type	Width [min]	Area [mAU*s]	Height [mAU]	Area %
1	6.524	MM	0.1395	6210.74316	741.97253	49.9442
2	7.298	MM	0.1619	6224.63135	640.60931	50.0558
Totals :				1.24354e4	1382.58185	

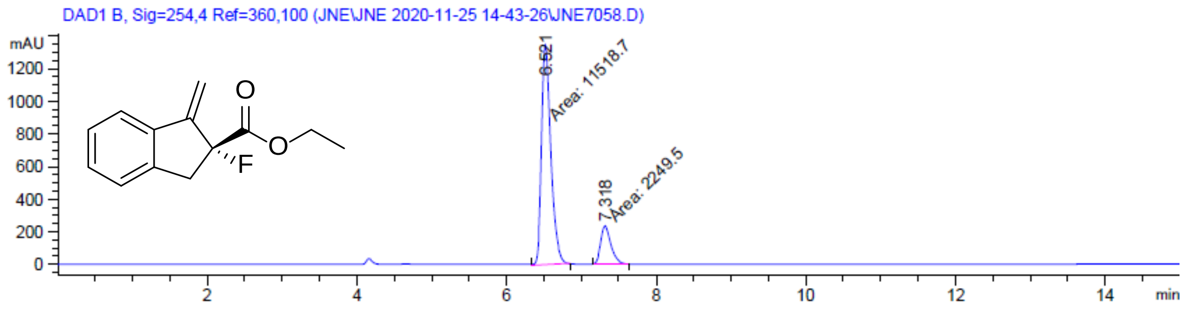

Signal 1: DAD1 B, Sig=254,4 Ref=360,100

Peak #	RetTime [min]	Type	Width [min]	Area [mAU*s]	Height [mAU]	Area %
1	6.521	MM	0.1424	1.15187e4	1347.91833	83.6617
2	7.318	MM	0.1596	2249.49707	234.87755	16.3383
Totals :				1.37682e4	1582.79588	

Ethyl (*S*)-2,3,3,-trifluoro-1,2,3,4-tetrahydronaphthalene-2-carboxylate (29)

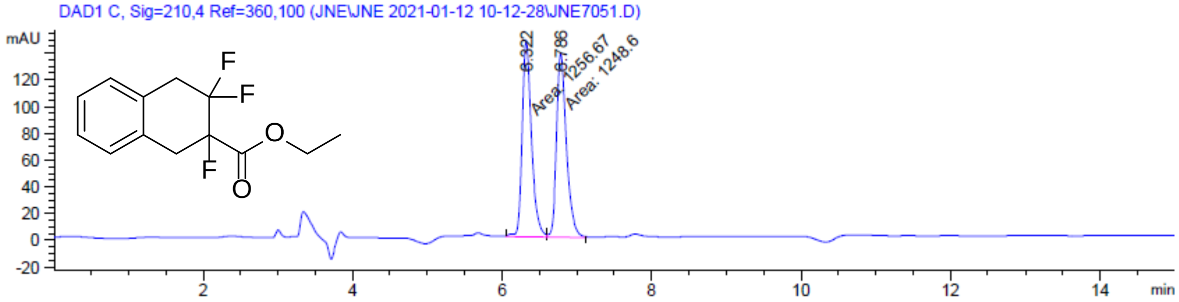

Signal 2: DAD1 C, Sig=210,4 Ref=360,100

Peak #	RetTime [min]	Type	Width [min]	Area [mAU*s]	Height [mAU]	Area %
1	6.322	MF	0.1429	1256.67358	146.61745	50.1612
2	6.786	FM	0.1514	1248.59827	137.44135	49.8388

Totals : 2505.27185 284.05879

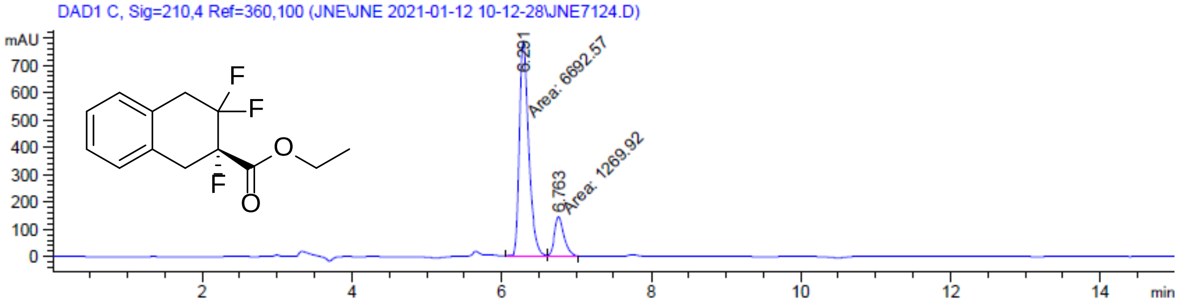

Signal 2: DAD1 C, Sig=210,4 Ref=360,100

Peak #	RetTime [min]	Type	Width [min]	Area [mAU*s]	Height [mAU]	Area %
1	6.291	MF	0.1419	6692.57227	785.89435	84.0512
2	6.763	FM	0.1466	1269.91907	144.34654	15.9488

Totals : 7962.49133 930.24089

Ethyl (*R*)-5-bromo-2-fluoro 1-oxo-2,3-dihydro-1*H*-indene-2-carboxylate (S45)

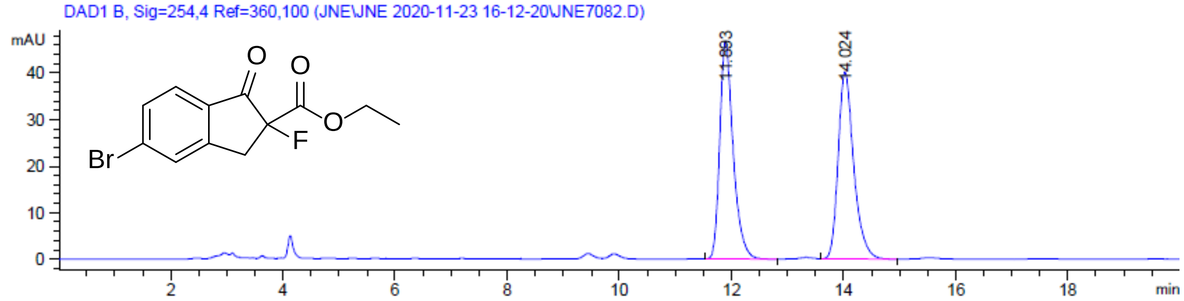

Signal 1: DAD1 B, Sig=254,4 Ref=360,100

Peak #	RetTime [min]	Type	Width [min]	Area [mAU*s]	Height [mAU]	Area %
1	11.893	BB	0.2514	775.28113	46.76352	50.0891
2	14.024	BB	0.2924	772.52435	40.15824	49.9109

Totals : 1547.80548 86.92175

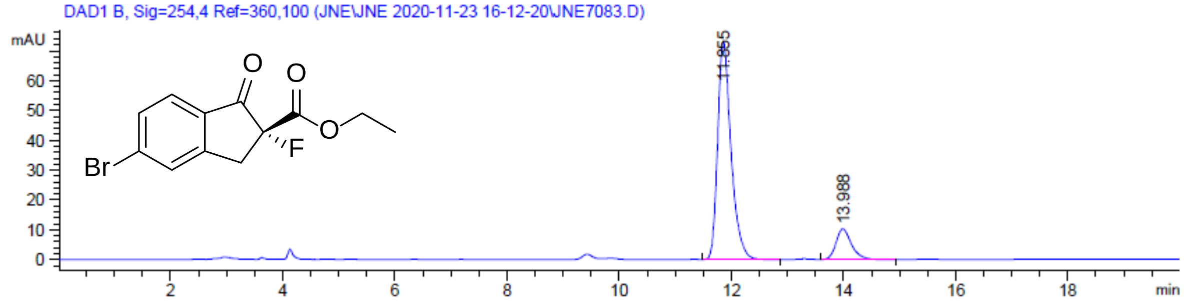

Signal 1: DAD1 B, Sig=254,4 Ref=360,100

Peak #	RetTime [min]	Type	Width [min]	Area [mAU*s]	Height [mAU]	Area %
1	11.855	BB	0.2510	1210.07019	73.16468	86.0420
2	13.988	BB	0.2873	196.30069	10.25399	13.9580

Totals : 1406.37088 83.41867

Ethyl (*R*)-5-bromo-2-fluoro 1-methylene-2,3-dihydro-1*H*-indene-2-carboxylate (S46)

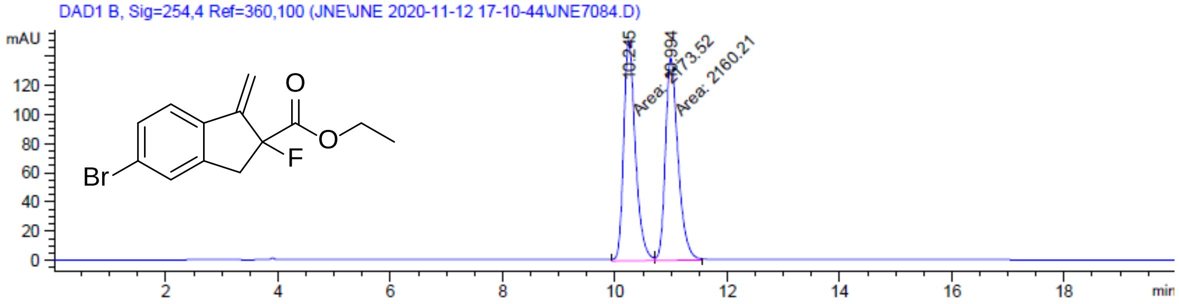

Signal 2: DAD1 B, Sig=254,4 Ref=360,100

Peak #	RetTime [min]	Type	Width [min]	Area [mAU*s]	Height [mAU]	Area %
1	10.245	MF	0.2409	2173.51709	150.35228	50.1536
2	10.994	FM	0.2605	2160.20679	138.22412	49.8464
Totals :				4333.72388	288.57640	

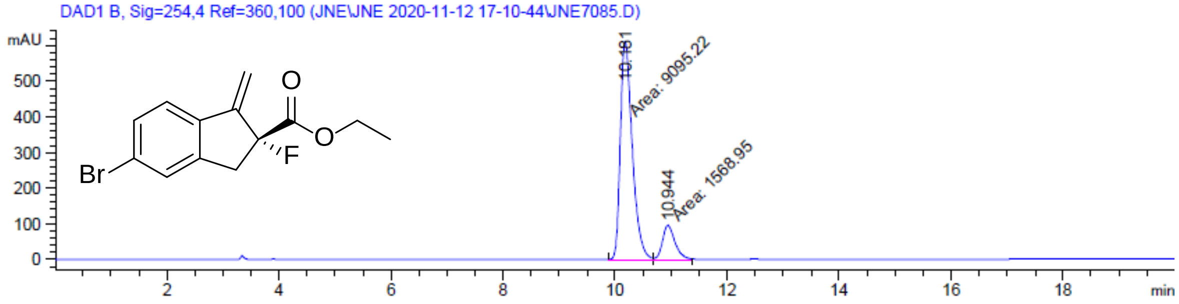

Signal 2: DAD1 B, Sig=254,4 Ref=360,100

Peak #	RetTime [min]	Type	Width [min]	Area [mAU*s]	Height [mAU]	Area %
1	10.181	MF	0.2467	9095.22461	614.52124	85.2877
2	10.944	FM	0.2673	1568.94897	97.81023	14.7123
Totals :				1.06642e4	712.33147	

Ethyl (*S*)-7-bromo-2,3,3,-trifluoro-1,2,3,4-tetrahydronaphthalene-2-carboxylate (30)

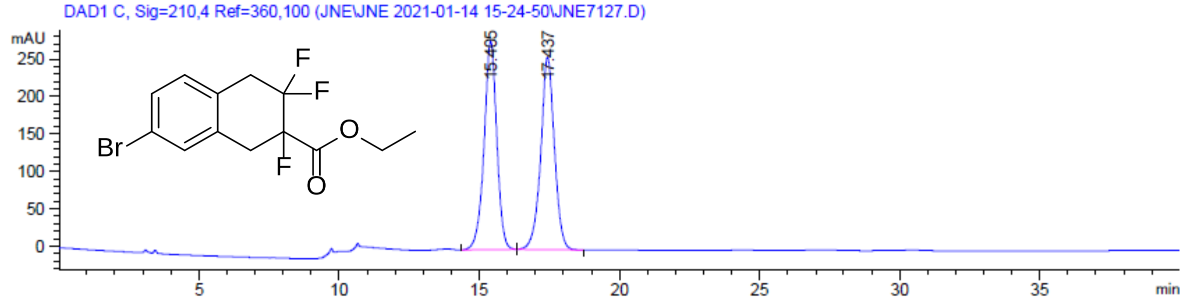

Signal 2: DAD1 C, Sig=210,4 Ref=360,100

Peak #	RetTime [min]	Type	Width [min]	Area [mAU*s]	Height [mAU]	Area %
1	15.405	BB	0.4747	8777.47754	279.24423	50.1458
2	17.437	BB	0.5057	8726.44824	257.29108	49.8542
Totals :				1.75039e4	536.53531	

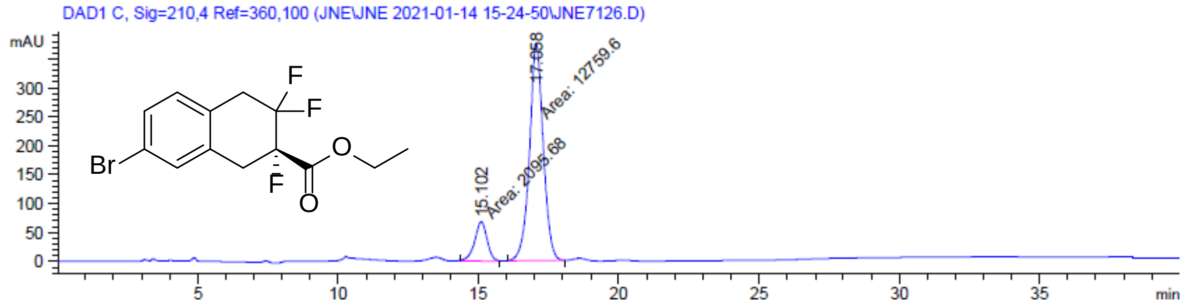

Signal 2: DAD1 C, Sig=210,4 Ref=360,100

Peak #	RetTime [min]	Type	Width [min]	Area [mAU*s]	Height [mAU]	Area %
1	15.102	MM	0.5119	2095.68091	68.23520	14.1073
2	17.058	MM	0.5661	1.27596e4	375.65759	85.8927
Totals :				1.48553e4	443.89279	

Ethyl (*R*)-5-chloro-2-fluoro 1-oxo-2,3-dihydro-1*H*-indene-2-carboxylate (S48)

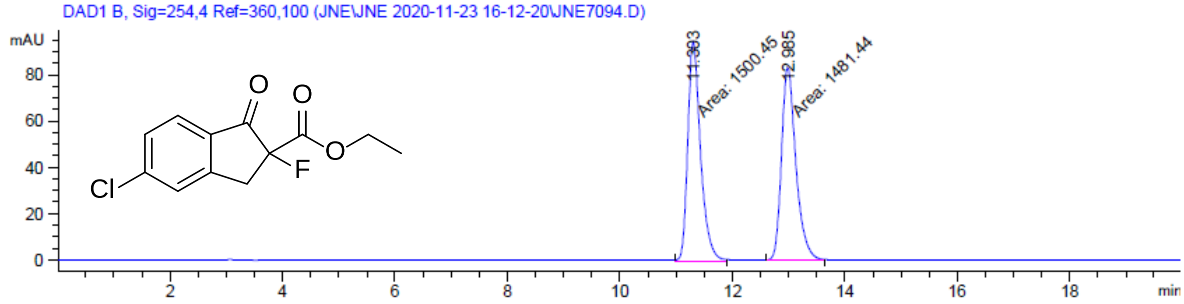

Signal 1: DAD1 B, Sig=254,4 Ref=360,100

Peak #	RetTime [min]	Type	Width [min]	Area [mAU*s]	Height [mAU]	Area %
1	11.303	MM	0.2636	1500.44678	94.85668	50.3187
2	12.985	MM	0.2971	1481.43799	83.09279	49.6813

Totals : 2981.88477 177.94947

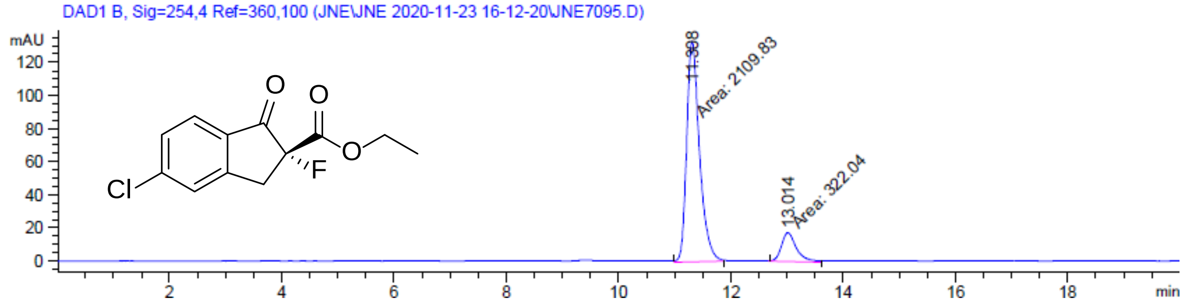

Signal 1: DAD1 B, Sig=254,4 Ref=360,100

Peak #	RetTime [min]	Type	Width [min]	Area [mAU*s]	Height [mAU]	Area %
1	11.308	MM	0.2640	2109.83057	133.17911	86.7575
2	13.014	MM	0.3082	322.03992	17.41377	13.2425

Totals : 2431.87048 150.59288

Ethyl (*R*)-5-chloro-2-fluoro 1-methylene-2,3-dihydro-1*H*-indene-2-carboxylate (S49)

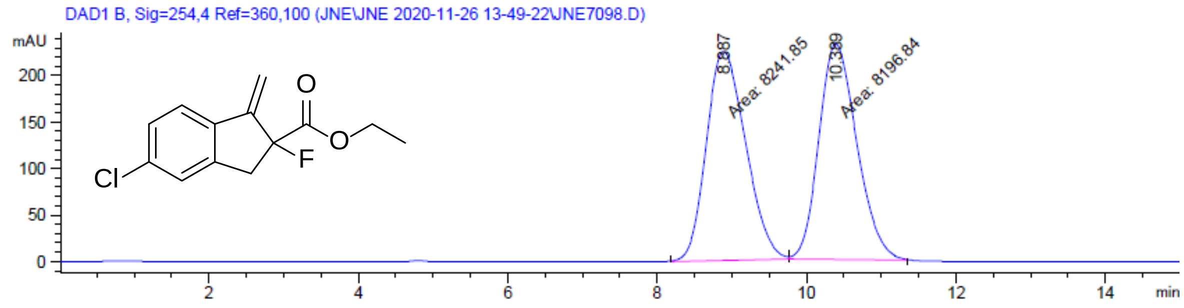

Signal 2: DAD1 B, Sig=254,4 Ref=360,100

Peak #	RetTime [min]	Type	Width [min]	Area [mAU*s]	Height [mAU]	Area %
1	8.887	MM	0.6102	8241.84668	225.12640	50.1369
2	10.389	MM	0.5871	8196.83594	232.67943	49.8631

Totals : 1.64387e4 457.80583

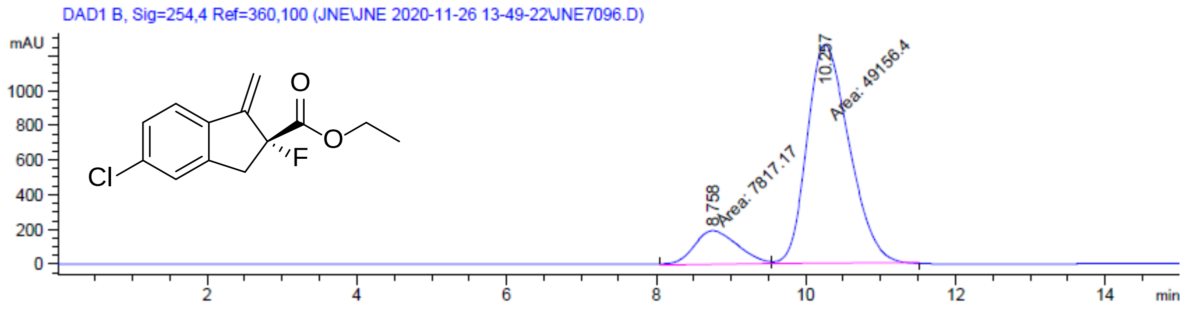

Signal 2: DAD1 B, Sig=254,4 Ref=360,100

Peak #	RetTime [min]	Type	Width [min]	Area [mAU*s]	Height [mAU]	Area %
1	8.758	MF	0.6741	7817.16992	193.26842	13.7207
2	10.257	FM	0.6499	4.91564e4	1260.58167	86.2793

Totals : 5.69736e4 1453.85008

Ethyl (*S*)-7-chloro-2,3,3,-trifluoro-1,2,3,4-tetrahydronaphthalene-2-carboxylate (31)

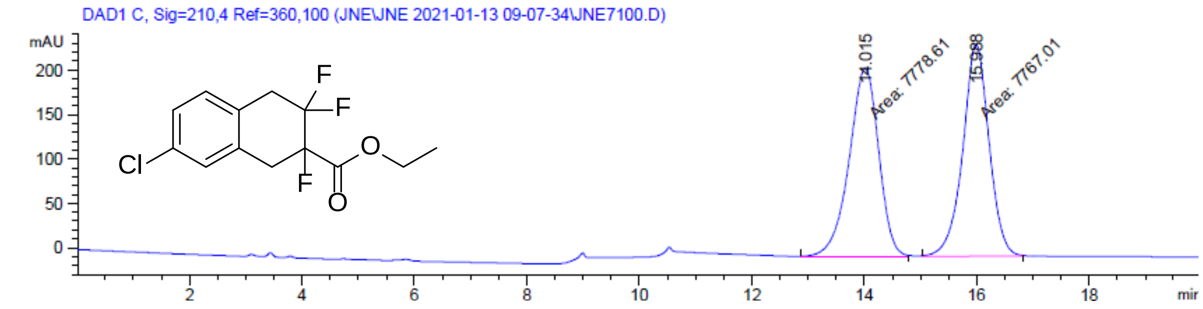


```
Signal 2: DAD1 C, Sig=210,4 Ref=360,100

Peak RetTime Type  Width     Area       Height      Area
  #   [min]        [min]   [mAU*s]      [mAU]        %
----|-------|----|-------|----------|----------|--------|
   1  14.015 MM    0.6117 7778.61182  211.94893  50.0373
   2  15.988 MM    0.5421 7767.01172  238.80028  49.9627

Totals :                  1.55456e4   450.74921
```

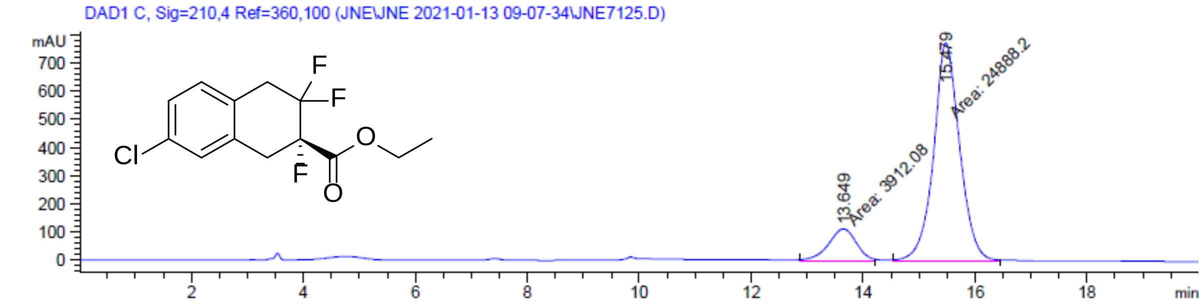


```
Signal 2: DAD1 C, Sig=210,4 Ref=360,100

Peak RetTime Type  Width     Area       Height      Area
  #   [min]        [min]   [mAU*s]      [mAU]        %
----|-------|----|-------|----------|----------|--------|
   1  13.649 MM    0.5765 3912.08398  113.09968  13.5835
   2  15.479 MM    0.5381 2.48882e4   770.80743  86.4165

Totals :                  2.88003e4   883.90711
```

Ethyl (*R*)-2-fluoro 1-oxo-6-(trifluoromethyl)-2,3-dihydro-1*H*-indene-2-carboxylate (S51)

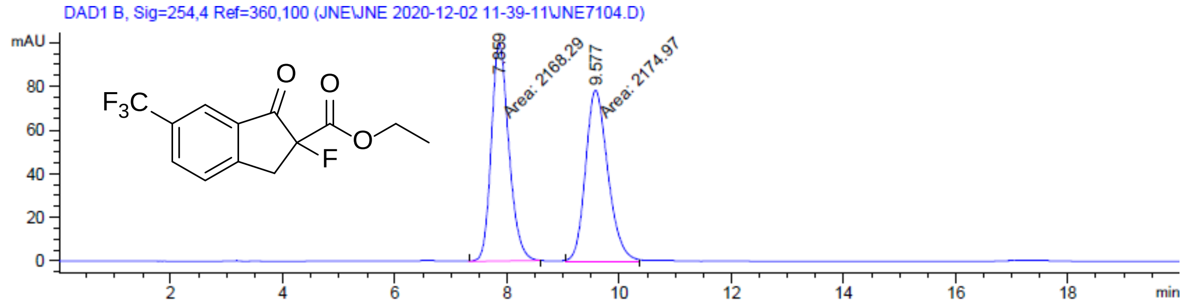

Signal 1: DAD1 B, Sig=254,4 Ref=360,100

Peak #	RetTime [min]	Type	Width [min]	Area [mAU*s]	Height [mAU]	Area %
1	7.859	MM	0.3624	2168.29004	99.73103	49.9231
2	9.577	MM	0.4631	2174.96606	78.28299	50.0769

Totals : 4343.25610 178.01402

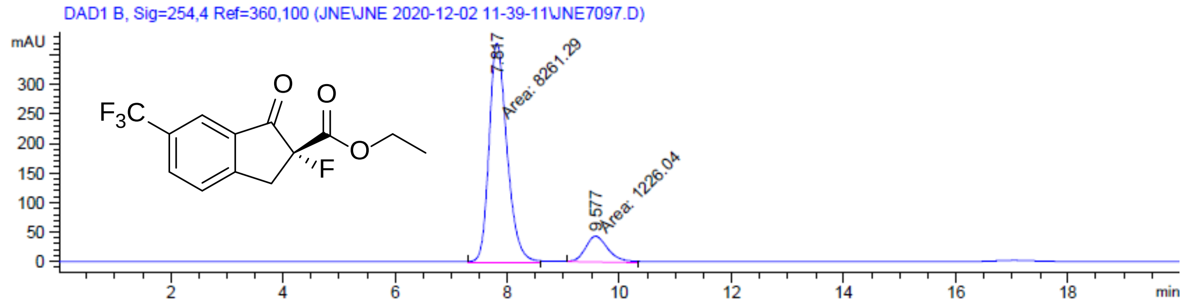

Signal 1: DAD1 B, Sig=254,4 Ref=360,100

Peak #	RetTime [min]	Type	Width [min]	Area [mAU*s]	Height [mAU]	Area %
1	7.817	MM	0.3706	8261.28906	371.56952	87.0771
2	9.577	MM	0.4683	1226.04102	43.63327	12.9229

Totals : 9487.33008 415.20279

Ethyl (*R*)-2-fluoro 1-methylene-6-(trifluoromethyl)-2,3-dihydro-1*H*-indene-2-carboxylate (S52)

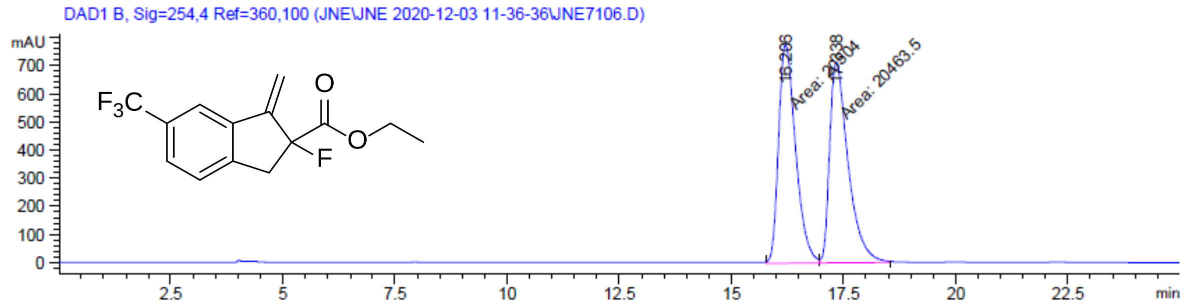

Signal 1: DAD1 B, Sig=254,4 Ref=360,100

Peak #	RetTime [min]	Type	Width [min]	Area [mAU*s]	Height [mAU]	Area %
1	16.206	MF	0.4393	2.05040e4	777.83057	50.0494
2	17.338	FM	0.4784	2.04635e4	712.89703	49.9506
Totals :				4.09675e4	1490.72760	

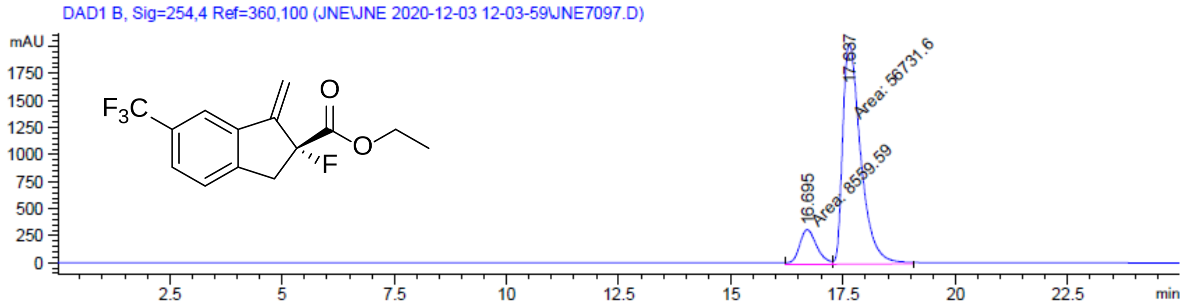

Signal 1: DAD1 B, Sig=254,4 Ref=360,100

Peak #	RetTime [min]	Type	Width [min]	Area [mAU*s]	Height [mAU]	Area %
1	16.695	MF	0.4469	8559.59180	319.23083	13.1099
2	17.637	FM	0.4645	5.67316e4	2035.38696	86.8901
Totals :				6.52912e4	2354.61780	

Ethyl (*S*)-2,3,3,-trifluoro-6-(trifluoromethyl)-1,2,3,4-tetrahydronaphthalene-2-carboxylate (32)

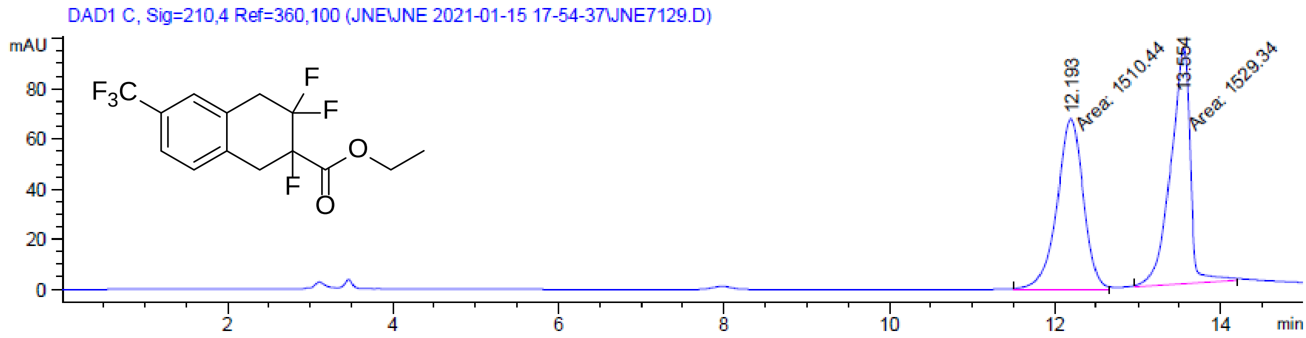

Signal 2: DAD1 C, Sig=210,4 Ref=360,100

Peak #	RetTime [min]	Type	Width [min]	Area [mAU*s]	Height [mAU]	Area %
1	12.193	MM	0.3702	1510.43994	67.99513	49.6891
2	13.554	MM	0.2714	1529.33948	93.93176	50.3109

Totals : 3039.77942 161.92690

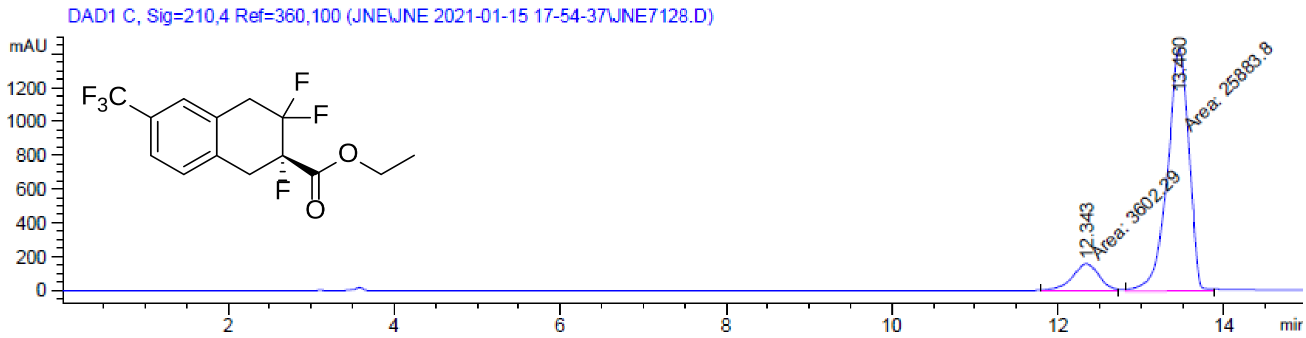

Signal 2: DAD1 C, Sig=210,4 Ref=360,100

Peak #	RetTime [min]	Type	Width [min]	Area [mAU*s]	Height [mAU]	Area %
1	12.343	MM	0.3761	3602.29321	159.64424	12.2169
2	13.460	MM	0.3004	2.58838e4	1436.02087	87.7831

Totals : 2.94861e4 1595.66512

VI. X-Ray Crystallographic Data

X-Ray diffraction: Data sets for compound **12** were collected with a Bruker D8 Venture PHOTON III diffractometer. Programs used: data collection: APEX3 V2016.1-0 (Bruker AXS Inc., **2016**); cell refinement: SAINT V8.37A (Bruker AXS Inc., **2015**); data reduction: SAINT V8.37A (Bruker AXS Inc., **2015**); absorption correction, SADABS V2014/7 (Bruker AXS Inc., **2014**); structure solution *SHELXT-2015* (Sheldrick, G. M. *Acta Cryst.,* **2015**, *A71*, 3-8); structure refinement *SHELXL-2015* (Sheldrick, G. M. *Acta Cryst.,* **2015**, *C71* (1), 3-8) and graphics, *XP* (Version 5.1, Bruker AXS Inc., Madison, Wisconsin, USA, **1998**). *R*-values are given for observed reflections, and wR^2 values are given for all reflections.

X-ray crystal structure analysis of 12 (gil9966): A colorless needle-like specimen of $C_{10}H_8BrF_3$, approximate dimensions 0.042 mm x 0.069 mm x 0.178 mm, was used for the X-ray crystallographic analysis. The X-ray intensity data were measured on a Bruker D8 Venture Bruker D8 Venture Photon III Diffractometer system equipped with a micro focus tube CuKα (CuKα, λ = 1.54178 Å) and a MX mirror monochromator. A total of 1237 frames were collected. The total exposure time was 15.70 hours. The frames were integrated with the Bruker SAINT software package using a wide-frame algorithm. The integration of the data using a monoclinic unit cell yielded a total of 13371 reflections to a maximum θ angle of 66.93° (0.84 Å resolution), of which 1674 were independent (average redundancy 7.987, completeness = 99.1%, R_{int} = 9.84%, R_{sig} = 5.87%) and 1519 (90.74%) were greater than $2\sigma(F^2)$. The final cell constants of a = 15.1288(7) Å, b = 6.0402(3) Å, c = 10.4294(5) Å, β = 93.652(2)°, volume = 951.11(8) $Å^3$, are based upon the refinement of the XYZ-centroids of 7899 reflections above 20 σ(I) with 5.853° < 2θ < 133.3°. Data were corrected for absorption effects using the Multi-Scan method (SADABS). The ratio of minimum to maximum apparent transmission was 0.561. The calculated minimum and maximum transmission coefficients (based on crystal size) are 0.4160 and 0.7880. The structure was solved and refined using the Bruker SHELXTL Software Package, using the space group $P2_1/c$, with Z = 4 for the formula unit, $C_{10}H_8BrF_3$. The final anisotropic full-matrix least-squares refinement on F^2 with 127 variables converged at R1 = 6.46%, for the observed data and wR2 = 17.68% for all data. The goodness-of-fit was 1.066. The largest peak in the final difference electron density synthesis was

1.526 $e^-/Å^3$ and the largest hole was -0.495 $e^-/Å^3$ with an RMS deviation of 0.141 $e^-/Å^3$. On the basis of the final model, the calculated density was 1.851 g/cm^3 and F(000), 520 e^-. CCDC number: 2057761.

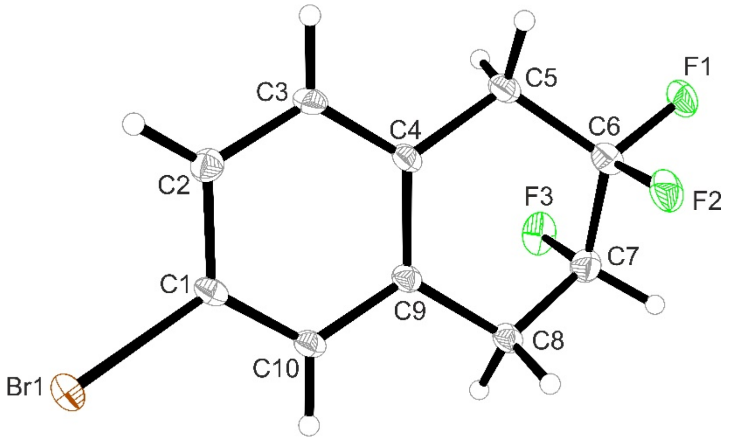

Figure S1 Crystal structure of compound **12**. Thermal ellipsoids are shown at 15% probability.

1. *APEX3* (**2016**), *SAINT* (**2015**) and *SADABS* (**2015**), Bruker AXS Inc., Madison, Wisconsin, USA.
2. Sheldrick, G. M., *SHELXT – Integrated space-group and crystal-structure determination*, *Acta Cryst.,* **2015**, *A71*, 3-8.
3. Sheldrick, G.M., *Crystal structure refinement with SHELXL*, *Acta Cryst.*, **2015**, *C71 (1)*, 3-8.
4. *XP – Interactive molecular graphics, Version 5.1,* Bruker AXS Inc., Madison, Wisconsin, USA, **1998**.

VII. DFT Calculations

Two conformers of **12** were optimised with the TPSS meta-GGA functional[1], an atom-pairwise dispersion correction (D3)[2] and the def2-TZVP basis set.[3] Gibbs free energies of translation, rotation and internal (harmonic) vibrations at 298 K (G298) were obtained with the same method. Single point calculations were performed with the hybrid functional PW6B95(-D3).[4] Geometry optimisations and energy evaluations were performed with the TURBOMOLE 7.4[5] NBO analysis[6] was done with the NBO 3.0 as implemented in Gaussian 16[7] with TPSS/def2-TZVP. Free energy of solvation (G_{solv}) in CH_2Cl_2 were calculated with the COSMO-RS method.[8]

Results

Table S2 Relative energies of **12-a** (conformer found in the solid state) and **12-b**.

Structure	E(TPSS-D3) [E_h]	G298 [kcal/mol]	E(PW6B95-D3) [E_h]	G_{solv} [kcal/mol]	ΔG_{298}(PW6) [kcal/mol]
**12-a**	-3259.883425	73.923	-3261.820809	-6.706	0.00
**12-b**	-3259.881048	73.917	-3261.818373	-7.233	1.53

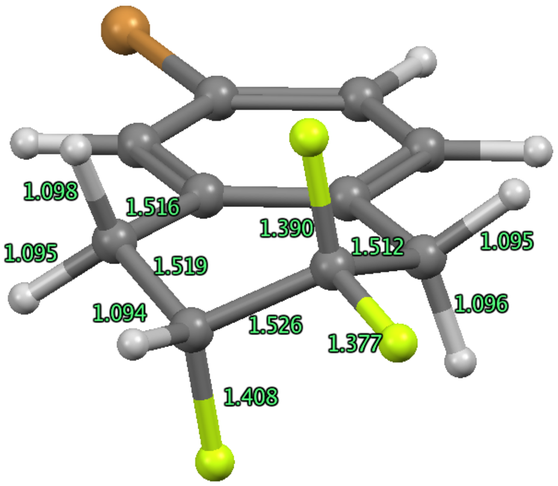

12-a

[0.0]

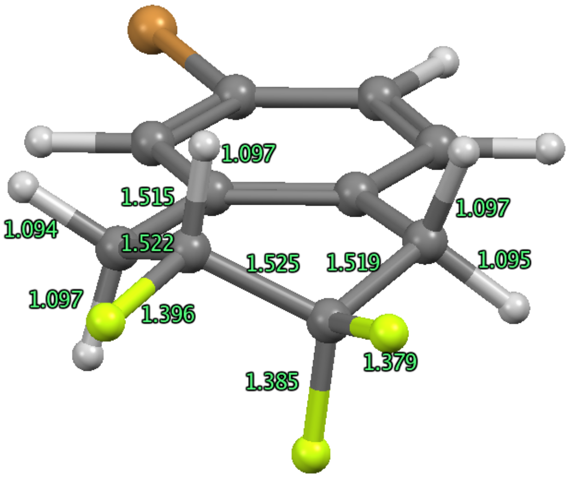

12-b

[+1.0]

Figure S2 Optimised molecular structure (TPSS-D3/def2-TZVP) of **12-a** and alternative conformer **12-b**. Internuclear distances are given in Å. In square brackets: relative free energies ΔG_{298}(PW6B95-D3//TPSS-D3/def2-TZVP) in kcal/mol.

NBO second order perturbation analysis (hyperconjugative interactions)

Only the interactions involving the C-F bonds and the C(F)-H bond are printed (in bold: σ(C-F)=BD(1), σ*(C-F) = BD*(1)). The largest contribution arises from *vicinal* σ(C-H) → σ*(C-F) interactions, which are slightly more stabilising in **12-a** than in **12-b**.

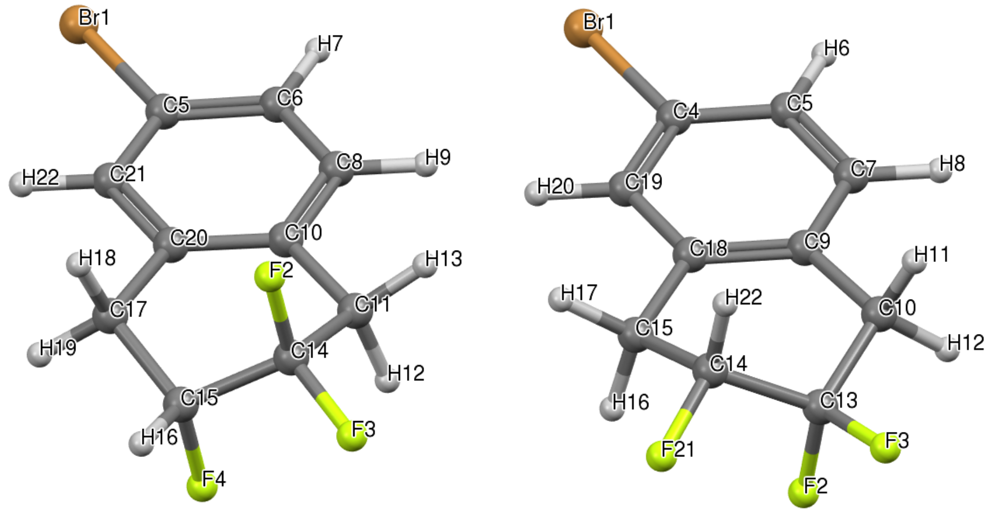

12-a **12-b**

Figure S3

12-a

2.	**BD (1) F 2 - C 14**	/477.	**BD*(1) F 4 - C 15**	1.12	1.03	0.031
2.	**BD (1) F 2 - C 14**	/489.	BD*(1) C 11 - H 12	0.75	1.20	0.027
3.	**BD (1) F 3 - C 14**	/486.	BD*(1) C 10 - C 11	1.06	1.22	0.032
3.	**BD (1) F 3 - C 14**	/494.	BD*(1) C 15 - C 17	0.77	1.18	0.027
4.	**BD (1) F 4 - C 15**	/475.	**BD*(1) F 2 - C 14**	1.26	1.00	0.032
4.	**BD (1) F 4 - C 15**	/495.	BD*(1) C 17 - H 18	0.80	1.16	0.027
13.	BD (1) C 10 - C 11	/476.	**BD*(1) F 3 - C 14**	2.47	0.76	0.039
16.	BD (1) C 11 - H 12	/475.	**BD*(1) F 2 - C 14**	**4.46**	0.63	0.048
18.	BD (1) C 11 - C 14	/493.	BD*(1) C 15 - H 16	1.19	0.90	0.029
20.	BD (1) C 15 - H 16	/491.	BD*(1) C 11 - C 14	2.56	0.77	0.040
20.	BD (1) C 15 - H 16	/497.	BD*(1) C 17 - C 20	2.81	0.82	0.043
21.	BD (1) C 15 - C 17	/476.	**BD*(1) F 3 - C 14**	2.50	0.76	0.039
22.	BD (1) C 17 - H 18	/477.	**BD*(1) F 4 - C 15**	**4.30**	0.62	0.046
24.	BD (1) C 17 - C 20	/493.	BD*(1) C 15 - H 16	1.34	0.88	0.031

12-b

2.	**BD (1) F 2 - C 13**	/488.	BD*(1) C 10 - H 11	0.76	1.19	0.027
2.	**BD (1) F 2 - C 13**	/494.	BD*(1) C 14 - H 22	0.69	1.17	0.025
3.	**BD (1) F 3 - C 13**	/485.	BD*(1) C 9 - C 10	1.09	1.21	0.033
3.	**BD (1) F 3 - C 13**	/492.	BD*(1) C 14 - C 15	0.79	1.17	0.027
12.	BD (1) C 9 - C 10	/476.	**BD*(1) F 3 - C 13**	2.31	0.76	0.038
15.	BD (1) C 10 - H 11	/475.	**BD*(1) F 2 - C 13**	**4.10**	0.64	0.046
17.	BD (1) C 10 - C 13	/493.	**BD*(1) C 14 - F 21**	2.13	0.76	0.036
19.	BD (1) C 14 - C 15	/476.	**BD*(1) F 3 - C 13**	2.62	0.75	0.040
20.	**BD (1) C 14 - F 21**	/490.	BD*(1) C 10 - C 13	0.92	1.12	0.029
20.	**BD (1) C 14 - F 21**	/497.	BD*(1) C 15 - C 18	1.10	1.18	0.032
21.	BD (1) C 14 - H 22	/475.	**BD*(1) F 2 - C 13**	**3.85**	0.66	0.046
21.	BD (1) C 14 - H 22	/495.	BD*(1) C 15 - H 16	1.99	0.82	0.036
22.	BD (1) C 15 - H 16	/494.	BD*(1) C 14 - H 22	2.58	0.75	0.039
24.	BD (1) C 15 - C 18	/493.	**BD*(1) C 14 - F 21**	2.22	0.75	0.036

Table S3 Relative Energies of cationic complexes **[8·(F-I-*p*-Tol)]⁺**

Structure	E(TPSS-D3) [E_h]	G298 [kcal/mol]	E(PW6B95-D3) [E_h]	G_{solv} [kcal/mol]	ΔG_{298}(PW6,solv) [kcal/mol]
**Cpx-a**	-1154.945495	143.206	-1155.759528	0.00	0.00
**Cpx-b**	-1154.949226	144.790	-1155.765412	-1.90	-4.01
**Cpx-c**	-1154.945056	144.564	-1155.758087	-2.83	-0.56

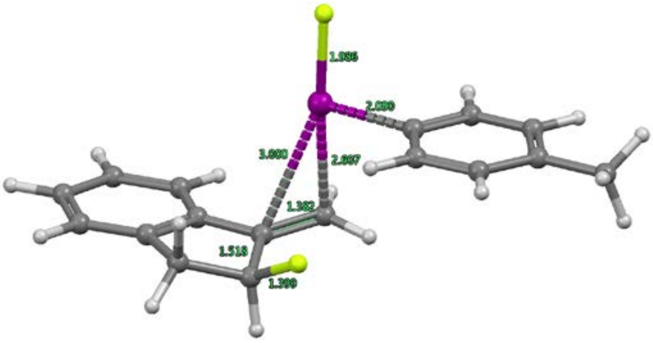

Cpx-a, (d(I···F(C_α)) = 3.49 Å)

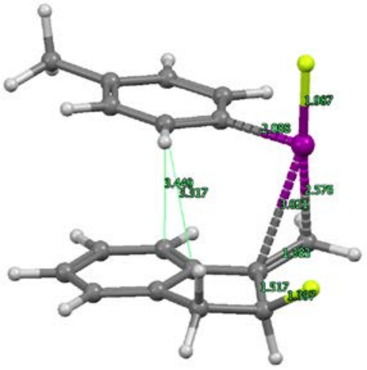

Cpx-b, (d(I···F(C_α)) = 3.33 Å)

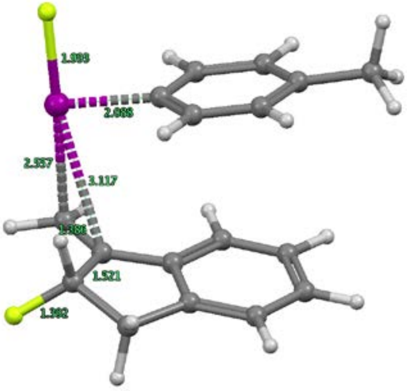

Cpx-c, (d(I···F(C_α)) = 4.43 Å)

DFT optimised (TPSS-D3/def2-TZVP) cartesian coordinates

12-a

```
E(TPSS-D3/def2-TZVP) = -3259.883424965 (conv)
Lowest Freq. =    41.73 cm^-1
22
X-a (001c1/opt)
Br    0.5505685    1.1861645    9.9979843
F     5.8296202    5.2296294    7.4922498
F     5.9719023    5.3160652    5.2863080
F     5.2403931    2.6147428    5.1571783
C     1.5734491    2.3246418    8.8557928
C     0.9748968    3.4528242    8.2985543
H    -0.0618372    3.6894371    8.5095730
C     1.7390387    4.2642676    7.4664446
H     1.2823274    5.1463166    7.0237609
C     3.0780340    3.9717576    7.1831623
C     3.8424574    4.8645206    6.2271108
H     3.5530350    4.6379864    5.1942367
H     3.6241924    5.9211834    6.4109823
C     5.3382528    4.6642487    6.3211867
C     5.7387167    3.1921520    6.3405836
H     6.8289395    3.1011181    6.3158072
C     5.1272181    2.4955561    7.5440496
H     5.7046884    2.7900245    8.4299214
H     5.2563473    1.4150099    7.4222855
C     3.6661388    2.8365196    7.7608406
C     2.9019312    2.0129914    8.5964924
H     3.3491803    1.1302164    9.0431551
```

12-b

```
E(TPSS-D3/def2-TZVP) = -3259.881048041 (conv)
Lowest Freq. =    42.24 cm^-1
22
X-b (001c2/opt)
Br    3.1531451    2.3039560   -2.6574593
F    -2.1837238   -1.7838808   -0.1422631
F    -2.2704785   -1.8144605    2.0656598
C     2.1221651    1.1559879   -1.5335558
C     2.7203524    0.0291001   -0.9724070
H     3.7609842   -0.2003892   -1.1714310
C     1.9515963   -0.7898408   -0.1522454
H     2.4088777   -1.6703000    0.2927518
C     0.6068601   -0.5063570    0.1147609
C    -0.1655420   -1.4091396    1.0553852
H     0.1509376   -1.2307562    2.0905335
H     0.0260067   -2.4645389    0.8360844
C    -1.6667252   -1.1882524    0.9958313
C    -2.0181271    0.2956517    0.9849271
C    -1.4471051    0.9520081   -0.2635948
H    -2.0280374    0.5867872   -1.1197675
H    -1.5959519    2.0342589   -0.2047914
C     0.0186264    0.6271895   -0.4676252
C     0.7888505    1.4594015   -1.2899845
H     0.3410689    2.3402148   -1.7392625
F    -3.4049694    0.4579201    1.0044429
H    -1.6235831    0.7472864    1.9034239
```

Cpx-a

```
E(TPSS-D3/def2-TZVP) = -1154.945495045 (conv)
Lowest Freq. =     2.88 cm^-1
36
Cpx-a (002c1/opt)
H     0.5365721   -5.5324824   -1.3021044
C    -0.2226892   -4.7652605   -1.4184336
C    -1.5569926   -5.1200577   -1.6332655
H    -1.8260855   -6.1707136   -1.6866603
C    -2.5628519   -4.1485397   -1.7823061
H    -3.5869566   -4.4621731   -1.9557516
C    -2.2491492   -2.7996174   -1.7249808
H    -3.0185145   -2.0462201   -1.8667336
C    -0.9066296   -2.4340065   -1.5103973
C     0.1025916   -3.4168129   -1.3442787
C     1.4381831   -2.7734789   -1.0694171
C    -0.3208313   -1.1236294   -1.4175973
C     1.1857674   -1.3043834   -1.4560868
H     2.2643122   -3.2262669   -1.6224227
H     1.6831684   -2.8194356   -0.0011836
C    -0.9460707    0.0981812   -1.2570737
H    -0.3601077    1.0103026   -1.3062632
H    -2.0176729    0.1902710   -1.4059738
F     1.8416421   -0.3910613   -0.6236235
H     1.5359734   -1.1028226   -2.4775434
C     1.7937464    3.0442400    1.8761039
C     1.2293537    1.7820615    1.7177703
C    -0.1428633    1.7099415    1.4786919
C    -0.9580011    2.8409604    1.3878194
C    -0.3612302    4.0860619    1.5502003
C     1.0169398    4.2101881    1.7937709
C     1.6461946    5.5676200    1.9448229
H     2.8594347    3.1221544    2.0717139
H     1.8397664    0.8891209    1.7804243
H    -2.0252876    2.7570403    1.2143270
H    -0.9802147    4.9772586    1.4950678
H     1.8161895    6.0173289    0.9584794
H     2.6103620    5.5055620    2.4545558
H     0.9935369    6.2445845    2.5033783
I    -1.0423675   -0.1712541    1.3343812
F    -1.3092180   -0.2446618    3.3005894
```

Cpx-b

```
E(TPSS-D3/def2-TZVP) = -1154.949226055 (conv)
Lowest Freq. =    17.58 cm^-1
36
Cpx-b (002c2/opt)
H     2.5769942   -2.4710889   -1.3798359
C     1.7469131   -1.7749535   -1.4516615
C     0.4594331   -2.2387158   -1.7365892
H     0.2998771   -3.3023490   -1.8877037
C    -0.6339392   -1.3614766   -1.8423723
H    -1.6157098   -1.7574878   -2.0802318
C    -0.4590710    0.0012735   -1.6628203
H    -1.2946370    0.6862231   -1.7634138
C     0.8316234    0.4743298   -1.3676718
C     1.9336849   -0.4118964   -1.2588994
C     3.1918904    0.3400567   -0.9034297
C     1.2763147    1.8184838   -1.1275147
C     2.7919742    1.8035304   -1.1788740
H     4.0686492    0.0190451   -1.4703773
H     3.4270498    0.2387993    0.1633789
C     0.5249291    2.9401753   -0.8298736
H     0.9995426    3.9180350   -0.8339610
H    -0.5551714    2.9207330   -0.9361288
F     3.3514605    2.7141700   -0.2799089
H     3.1142529    2.1135757   -2.1822349
C    -2.7553288    0.0515541    1.2478931
C    -1.9337900    1.1733056    1.3110918
C    -0.5744775    0.9772311    1.5592166
C    -0.0211923   -0.2906099    1.7454320
C    -0.8694596   -1.3912167    1.6817042
C    -2.2419281   -1.2419789    1.4327268
C    -3.1502978   -2.4402696    1.3935446
H    -3.8171220    0.1871124    1.0614161
H    -2.3474683    2.1681466    1.1843253
H     1.0363608   -0.4185543    1.9469526
H    -0.4547362   -2.3846597    1.8268913
H    -3.9798138   -2.2898639    0.6973471
H    -3.5832598   -2.6148645    2.3862854
H    -2.6054851   -3.3440755    1.1087843
I     0.6882087    2.6325510    1.7220911
F     0.5737293    2.5557295    3.7044216
```

Cpx-c

```
E(TPSS-D3/def2-TZVP) = -1154.945055815 (conv)
Lowest Freq. =    14.41 cm^-1
36
Cpx-c (002c3/opt)
H     2.4375633   -2.5795011    1.6343860
C     1.5513871   -1.9571470    1.5568608
C     0.2962398   -2.5382907    1.3566537
H     0.2188240   -3.6183409    1.2716269
C    -0.8755491   -1.7617544    1.2880022
H    -1.8334717   -2.2521211    1.1503330
C    -0.8087229   -0.3841711    1.3967312
H    -1.7076760    0.2195022    1.3373480
C     0.4557621    0.2136537    1.5653969
C     1.6307337   -0.5758119    1.6793691
C     2.8276746    0.2876816    1.9757999
C     0.7979753    1.5994693    1.6458578
C     2.3149026    1.7071809    1.6646963
H     3.7180446    0.0186395    1.4022827
H     3.0866705    0.2349939    3.0401009
C    -0.0471052    2.6978802    1.6525865
H    -1.1135382    2.5603095    1.8038415
H     0.3605016    3.6610770    1.9526895
F     2.7396844    2.6637709    2.5831454
H     2.6719689    2.0378635    0.6795870
C    -0.1103889   -0.8126778   -2.1327843
C     0.3216959    0.4440685   -1.7213428
C    -0.6439607    1.3922422   -1.3774975
C    -2.0124284    1.1225679   -1.4360595
C    -2.4124728   -0.1428667   -1.8554830
C    -1.4756732   -1.1268829   -2.2080026
C    -1.9265483   -2.4794967   -2.6874247
H     0.6290149   -1.5605083   -2.4052198
H     1.3797218    0.6790694   -1.6867181
H    -2.7474264    1.8771735   -1.1772208
H    -3.4742556   -0.3658511   -1.9140607
H    -1.1846484   -3.2498200   -2.4613189
H    -2.0649857   -2.4645988   -3.7756497
H    -2.8826737   -2.7632984   -2.2398651
I    -0.0169853    3.3077271   -0.8301625
F    -0.0998549    3.9082681   -2.7284849
```

1. J. Tao, J. P. Perdew, V. N. Staroverov and G. E. Scuseria, *Phys. Rev. Lett.*, **2003**, *91*, 146401.
2. a) S. Grimme, J. Antony, S. Ehrlich, H. Krieg, *J. Chem. Phys.* **2010**, *132*, 154104. b) S. Grimme, S. Ehrlich, L. Goerigk, *J. Comput. Chem.* **2011**, *32*, 1456–1465.
3. F. Weigend; R. Ahlrichs. *Phys. Chem. Chem. Phys.* **2005**, *7*, 3297–3305.
4. Y. Zhao, D. G. Truhlar, *J. Phys. Chem. A* **2005**, *109*, 5656-5667.
5. TURBOMOLE V7.4 2019, a development of University of Karlsruhe and Forschungszentrum Karlsruhe GmbH, 1989-2007, TURBOMOLE GmbH, since 2007; available from http://www.turbomole.com
6. A. E. Reed; R. B. Weinstock; F. Weinhold. *J. Chem. Phys.* **1985**, *83*, 735–746.
7. Gaussian 16, Revision A.03, M. J. Frisch, G. W. Trucks, H. B. Schlegel, G. E. Scuseria, M. A. Robb, J. R. Cheeseman, G. Scalmani, V. Barone, G. A. Petersson, H. Nakatsuji, X. Li, M. Caricato, A. V. Marenich, J. Bloino, B. G. Janesko, R. Gomperts, B. Mennucci, H. P. Hratchian, J. V. Ortiz, A. F. Izmaylov, J. L. Sonnenberg, D. Williams-Young, F. Ding, F. Lipparini, F. Egidi, J. Goings, B. Peng, A. Petrone, T. Henderson, D. Ranasinghe, V. G. Zakrzewski, J. Gao, N. Rega, G. Zheng, W. Liang, M. Hada, M. Ehara, K. Toyota, R. Fukuda, J. Hasegawa, M. Ishida, T. Nakajima, Y. Honda, O. Kitao, H. Nakai, T. Vreven, K. Throssell, J. A. Montgomery, Jr., J. E. Peralta, F. Ogliaro, M. J. Bearpark, J. J. Heyd, E. N. Brothers, K. N. Kudin, V. N. Staroverov, T. A. Keith, R. Kobayashi, J. Normand, K. Raghavachari, A. P. Rendell, J. C. Burant, S. S. Iyengar, J. Tomasi, M. Cossi, J. M. Millam, M. Klene, C. Adamo, R. Cammi, J. W. Ochterski, R. L. Martin, K. Morokuma, O. Farkas, J. B. Foresman, and D. J. Fox, Gaussian, Inc., Wallingford CT, **2016**.
8. a) A. Klamt, *J. Phys. Chem.* **1995**, *99*, 2224-2235. b) A. Klamt, V. Jonas, T. Bürger, J.C. Lohrenz, *J. Phys. Chem. A* **1998**, *102*, 5074-5085. c) F. Eckert, A. Klamt, *AIChE Journal*, **2002**, *48*, 369-385.

VIII. NMR Spectra of Unreported Starting Materials

2,5-Difluoro-1-methylene-2,3-dihydro-1*H*-indene (S3)

^{1}H NMR (500 MHz, $CDCl_3$, 299 K)

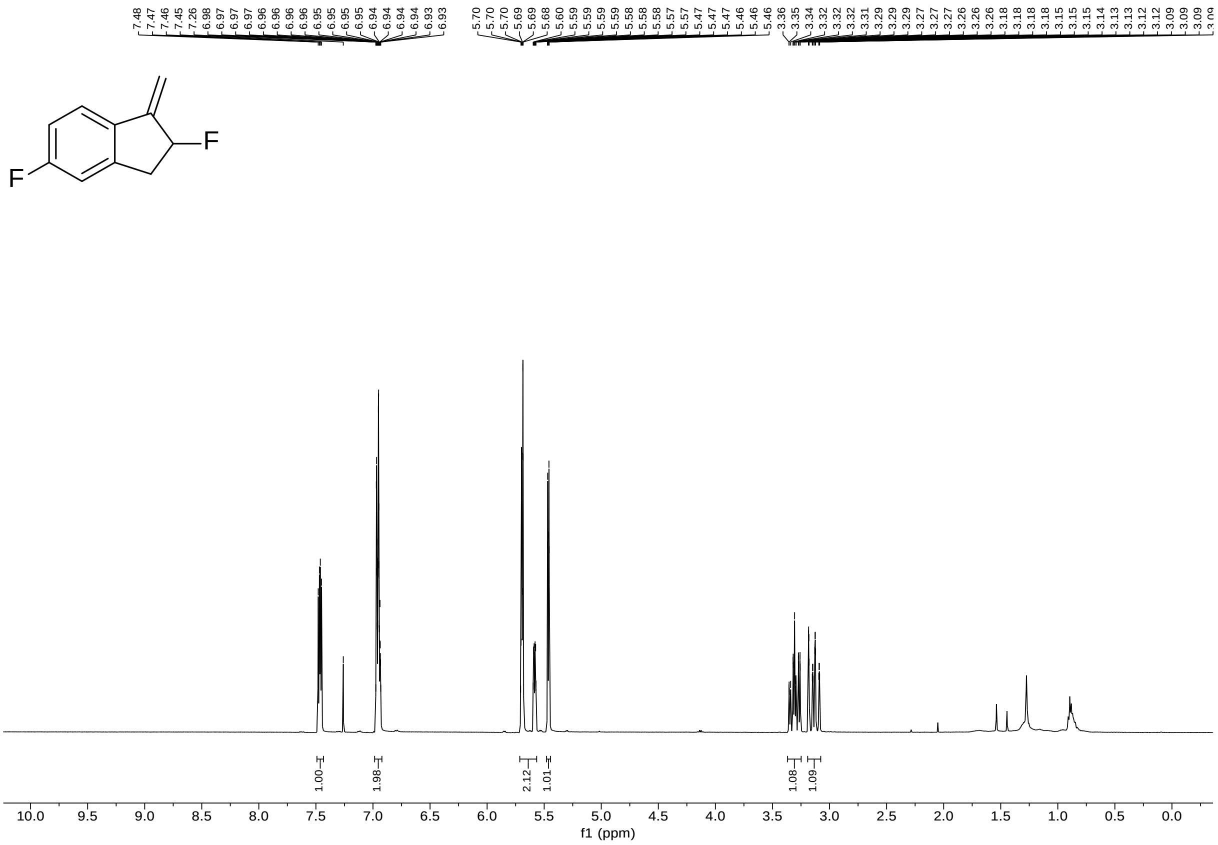

^{13}C NMR (126 MHz, $CDCl_3$, 299 K)

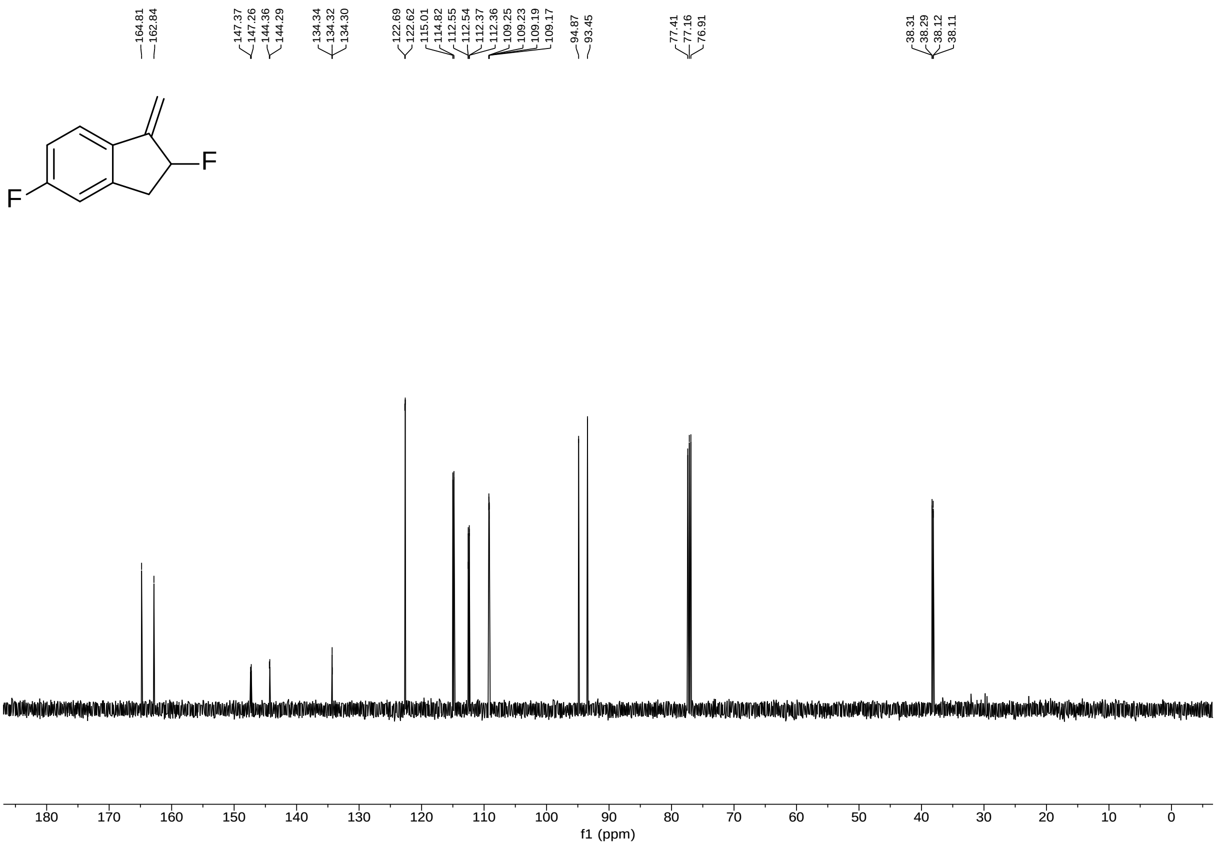

^{19}F NMR (470 MHz, $CDCl_3$, 299 K)

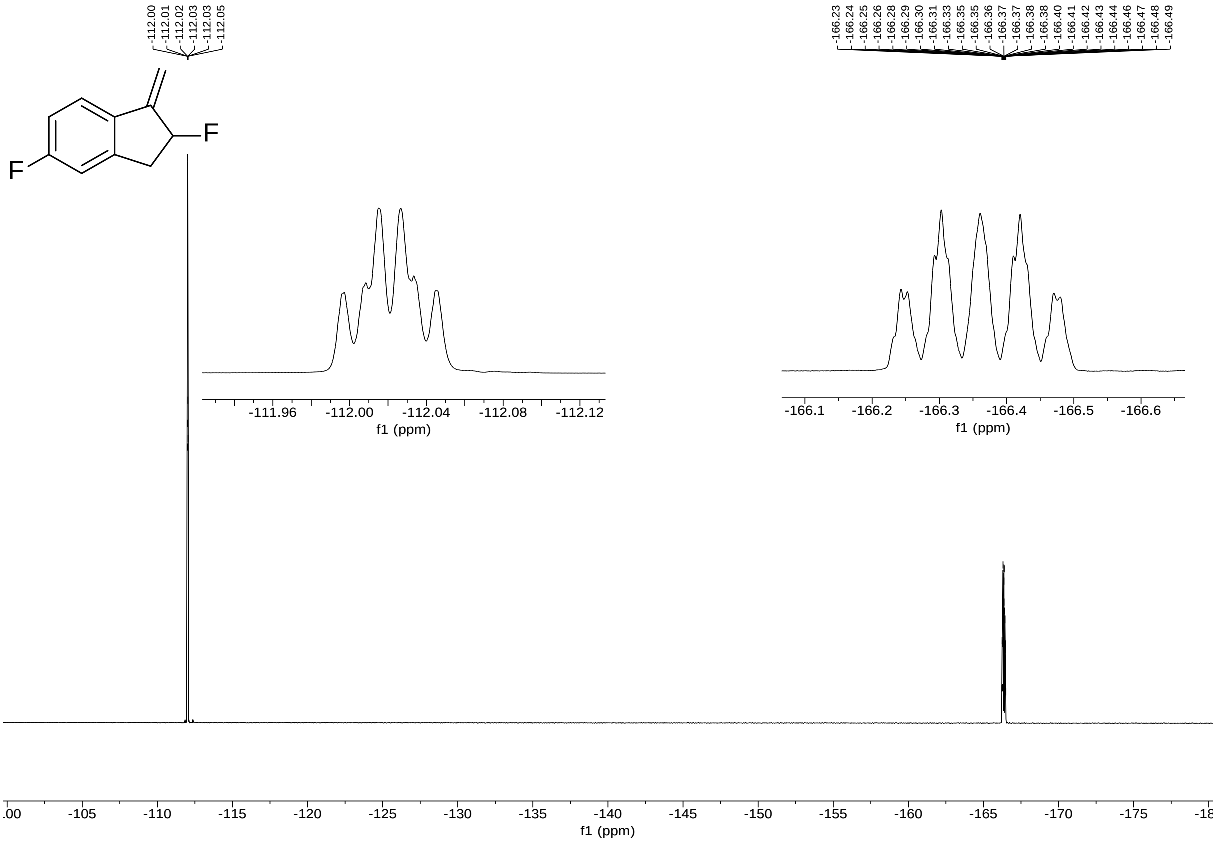

$^{19}F\{^1H\}$ NMR (470 MHz, $CDCl_3$, 299 K)

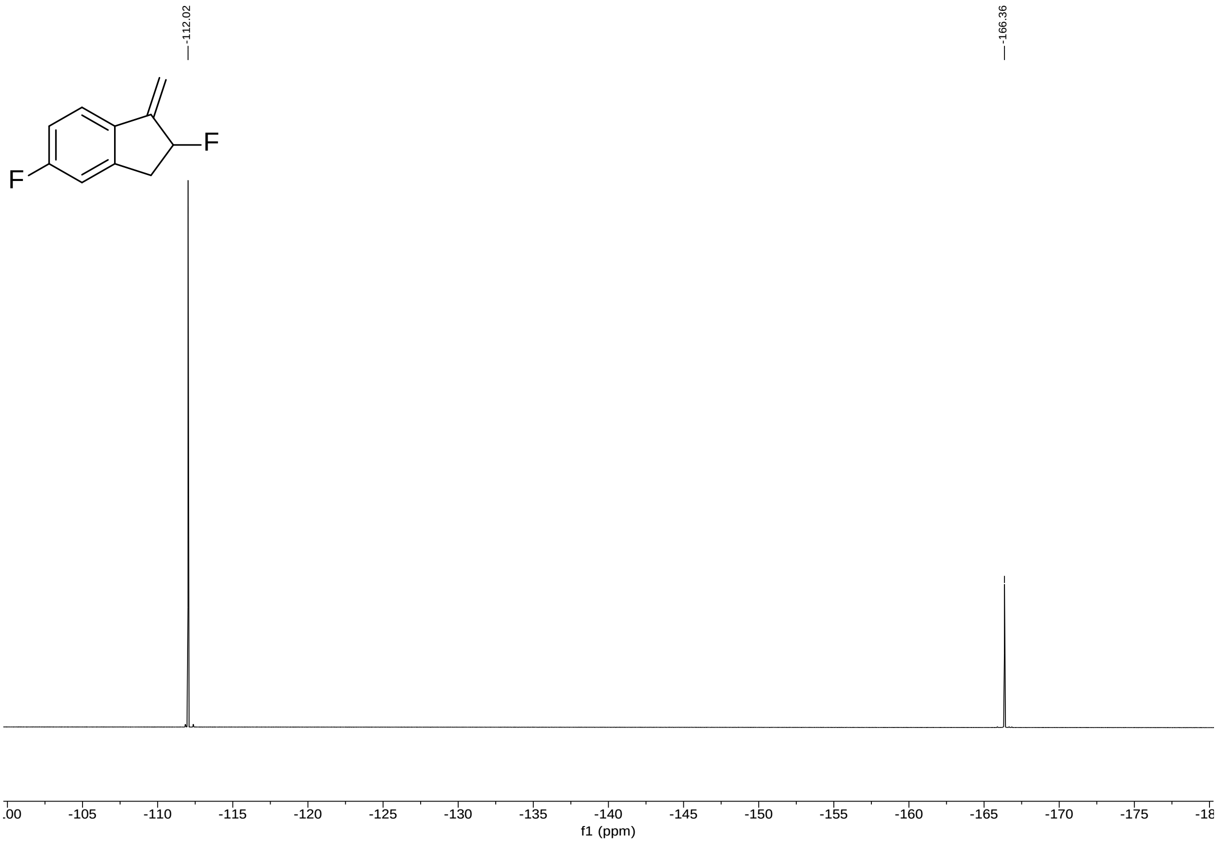

5-Chloro-2-fluoro-1-methylene-2,3-dihydro-1*H*-indene (S5)

^{1}H NMR (500 MHz, $CDCl_3$, 299 K)

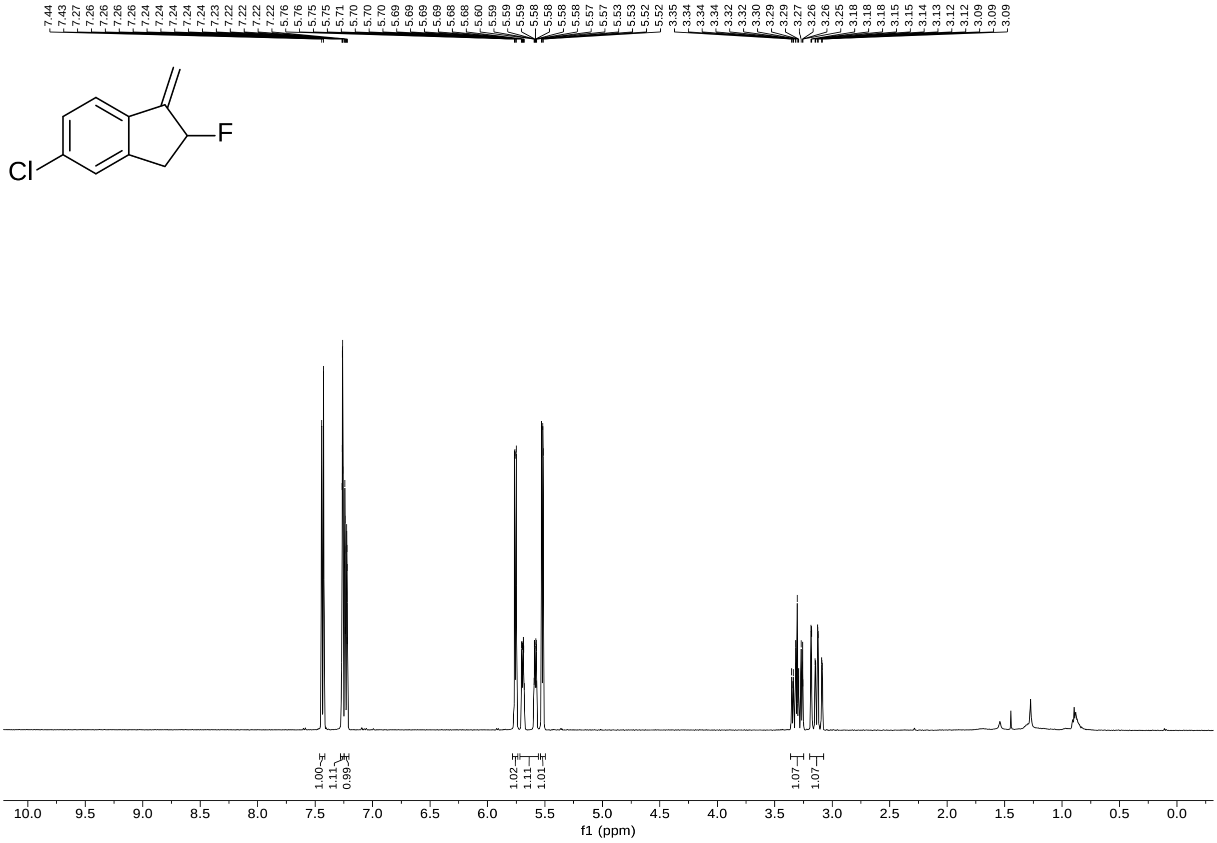

^{13}C NMR (126 MHz, $CDCl_3$, 299 K)

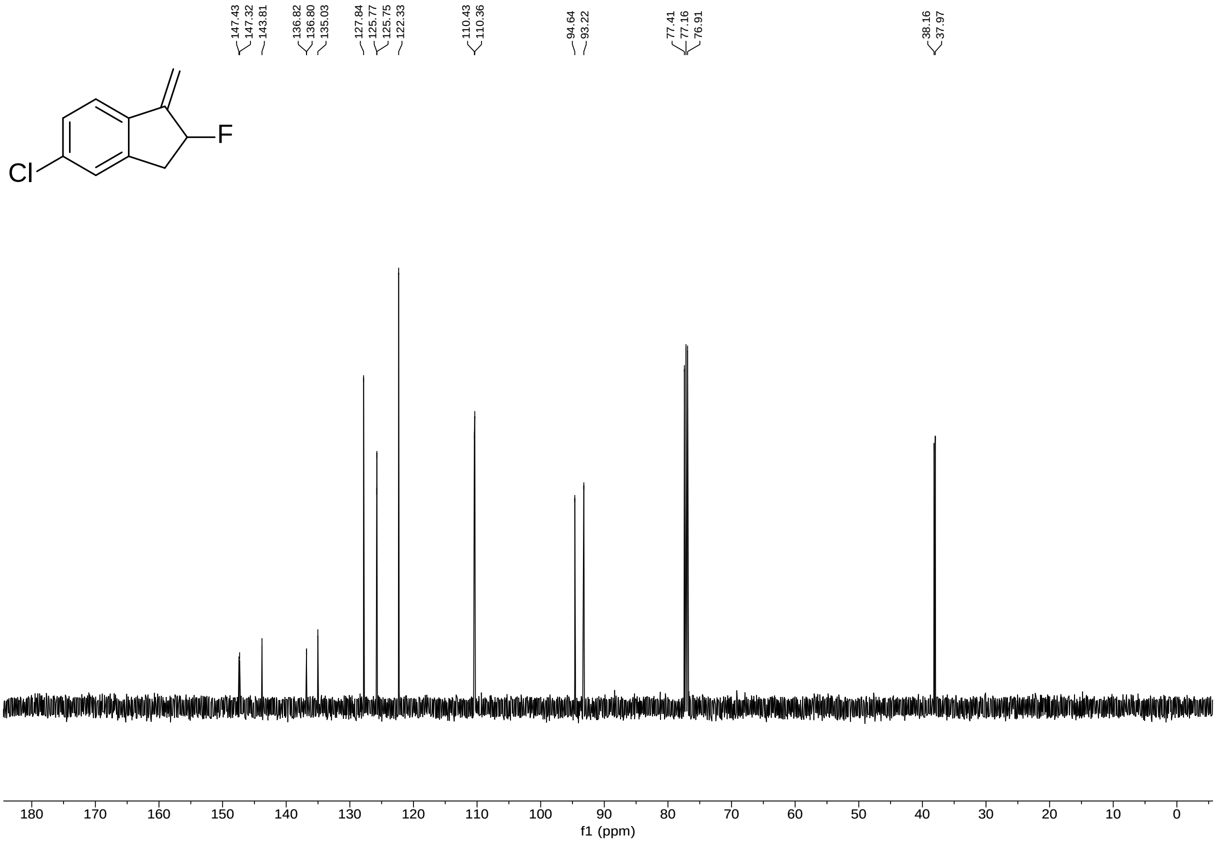

^{19}F NMR (470 MHz, $CDCl_3$, 299 K)

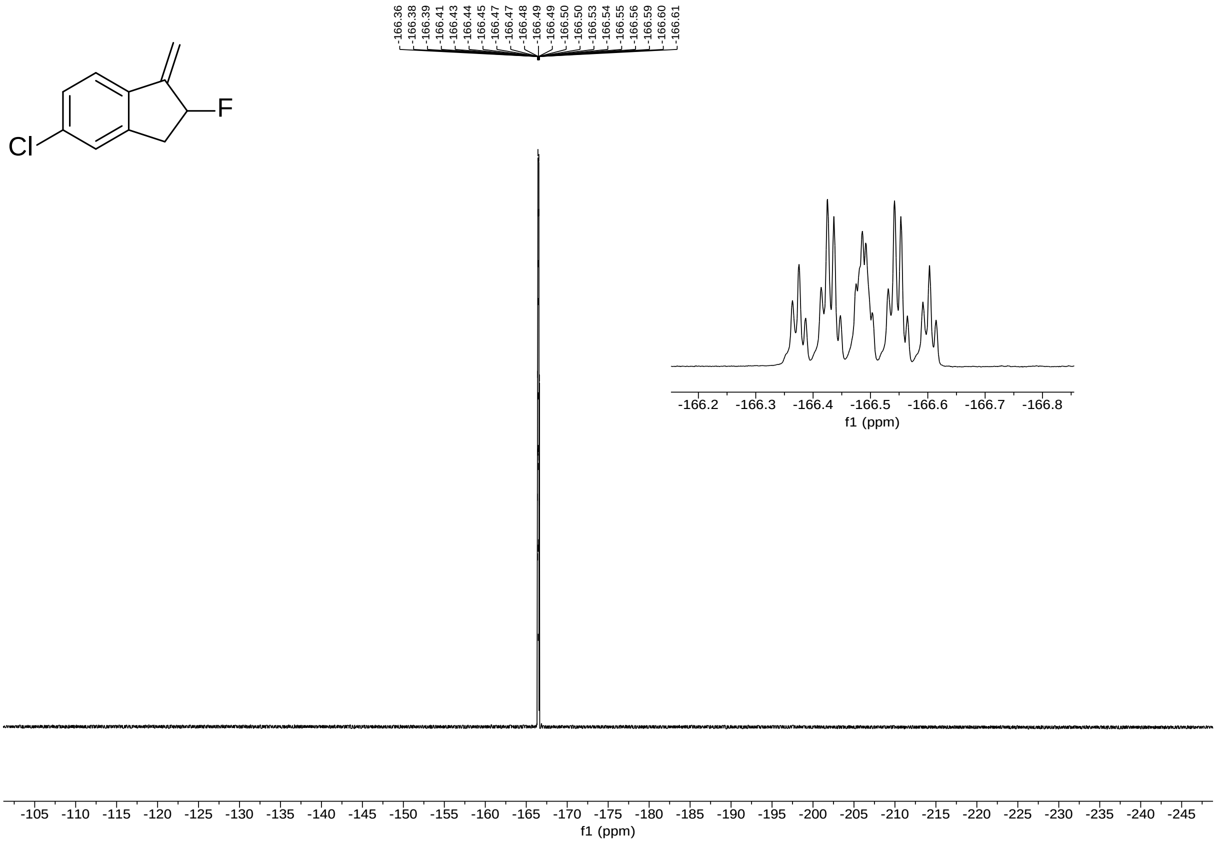

^{19}F{^{1}H} NMR (470 MHz, $CDCl_3$, 299 K)

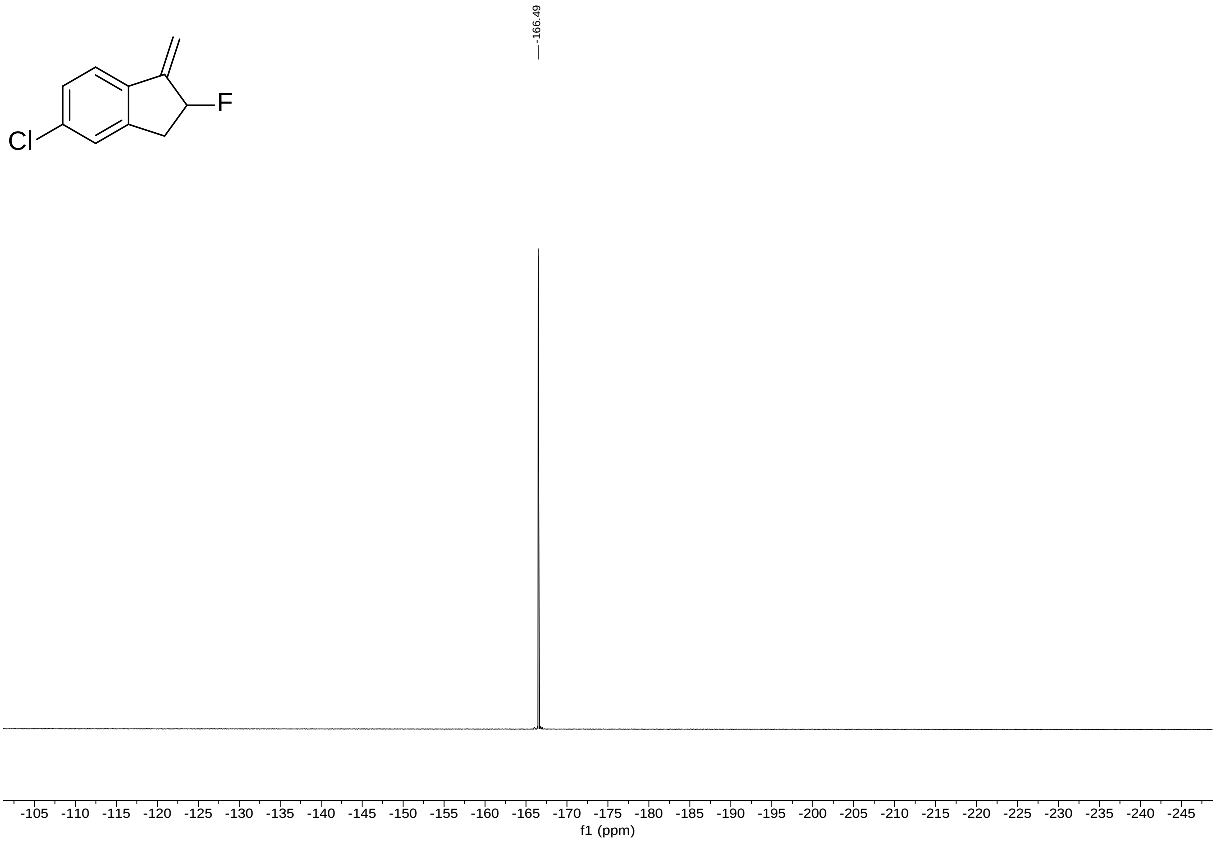

5-Bromo-2-fluoro-1-methylene-2,3-dihydro-1*H*-indene (S7)

^{1}H NMR (599 MHz, $CDCl_3$, 299 K)

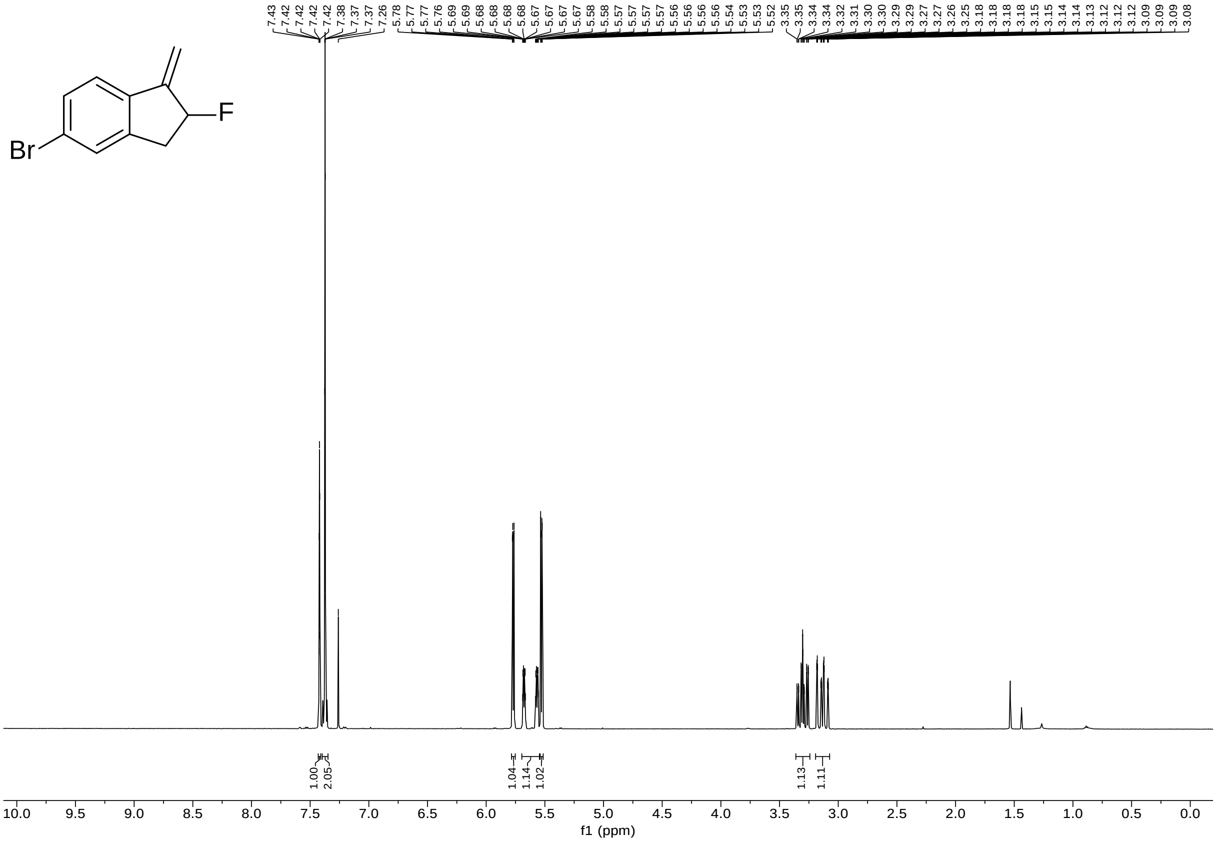

^{13}C NMR (126 MHz, $CDCl_3$, 299 K)

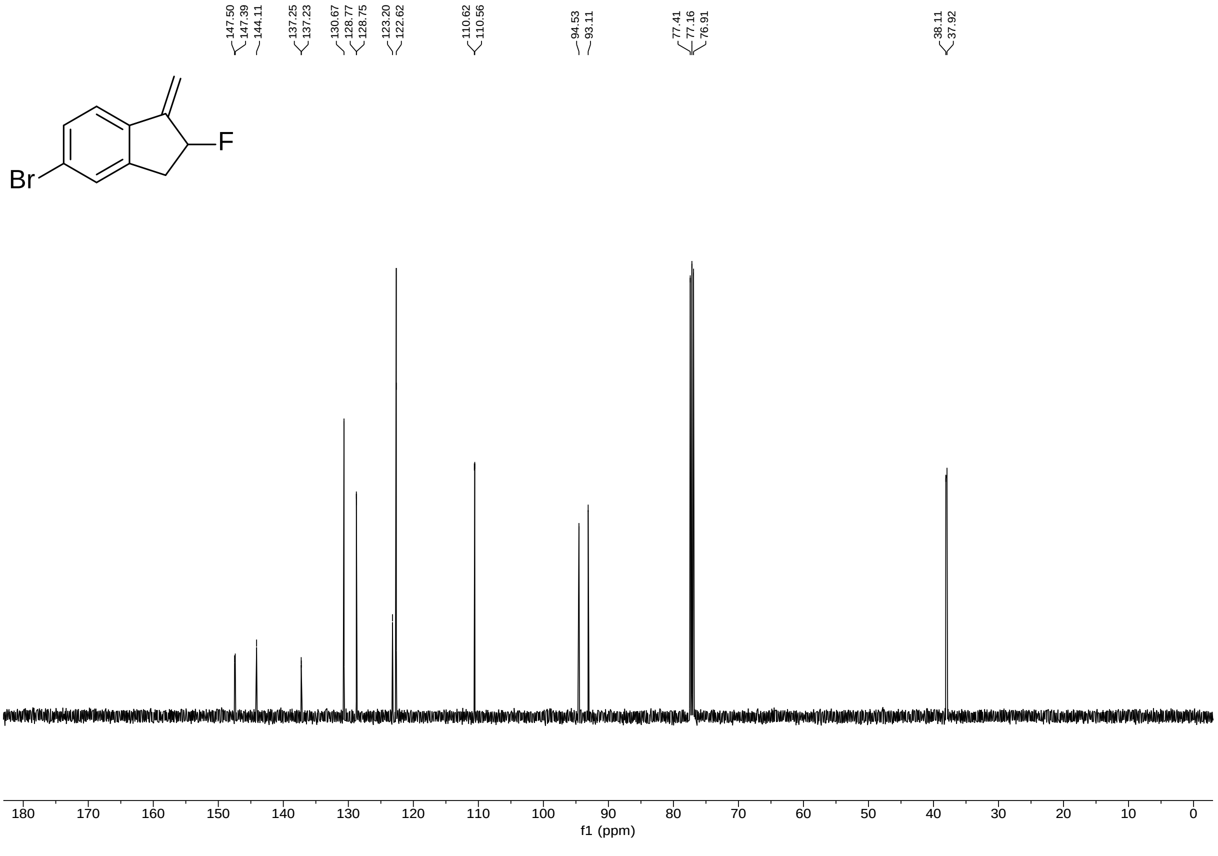

^{19}F NMR (470 MHz, $CDCl_3$, 299 K)

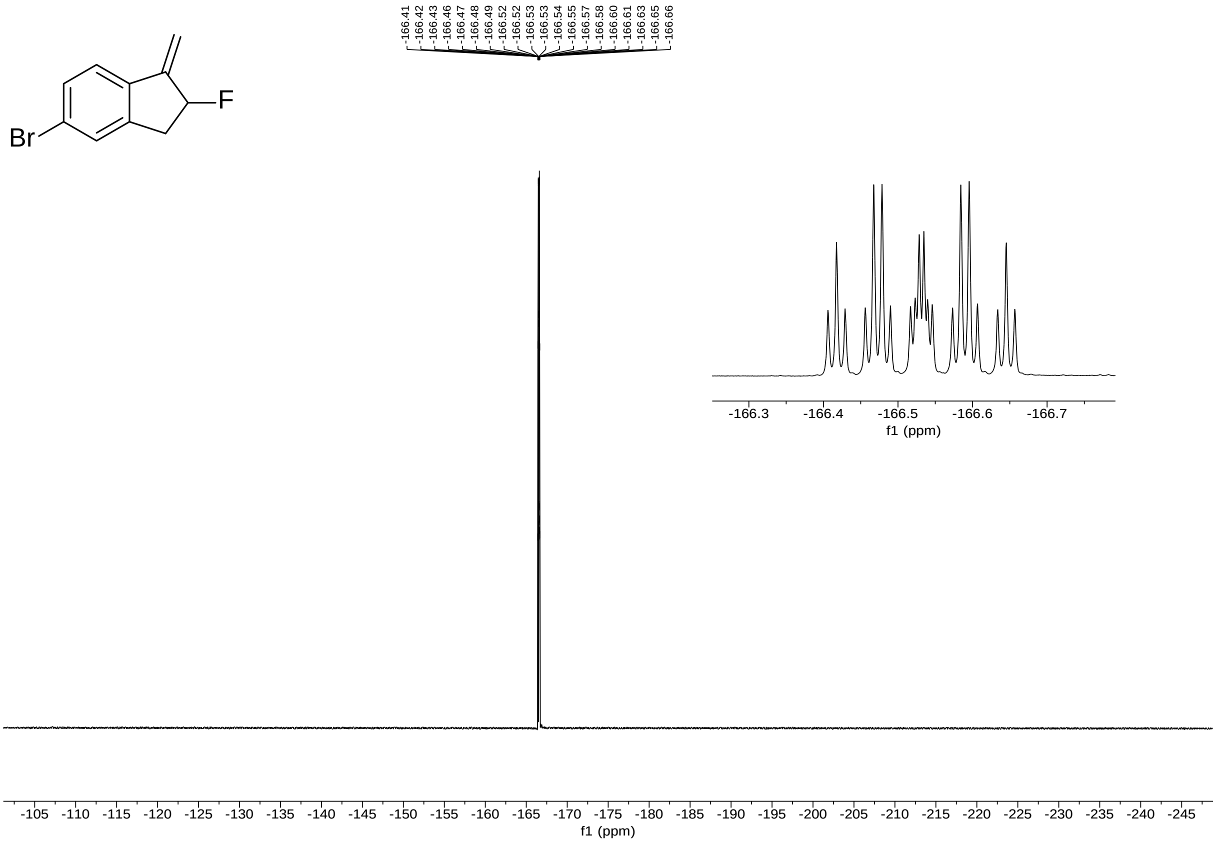

^{19}F{^{1}H} NMR (470 MHz, $CDCl_3$, 299 K)

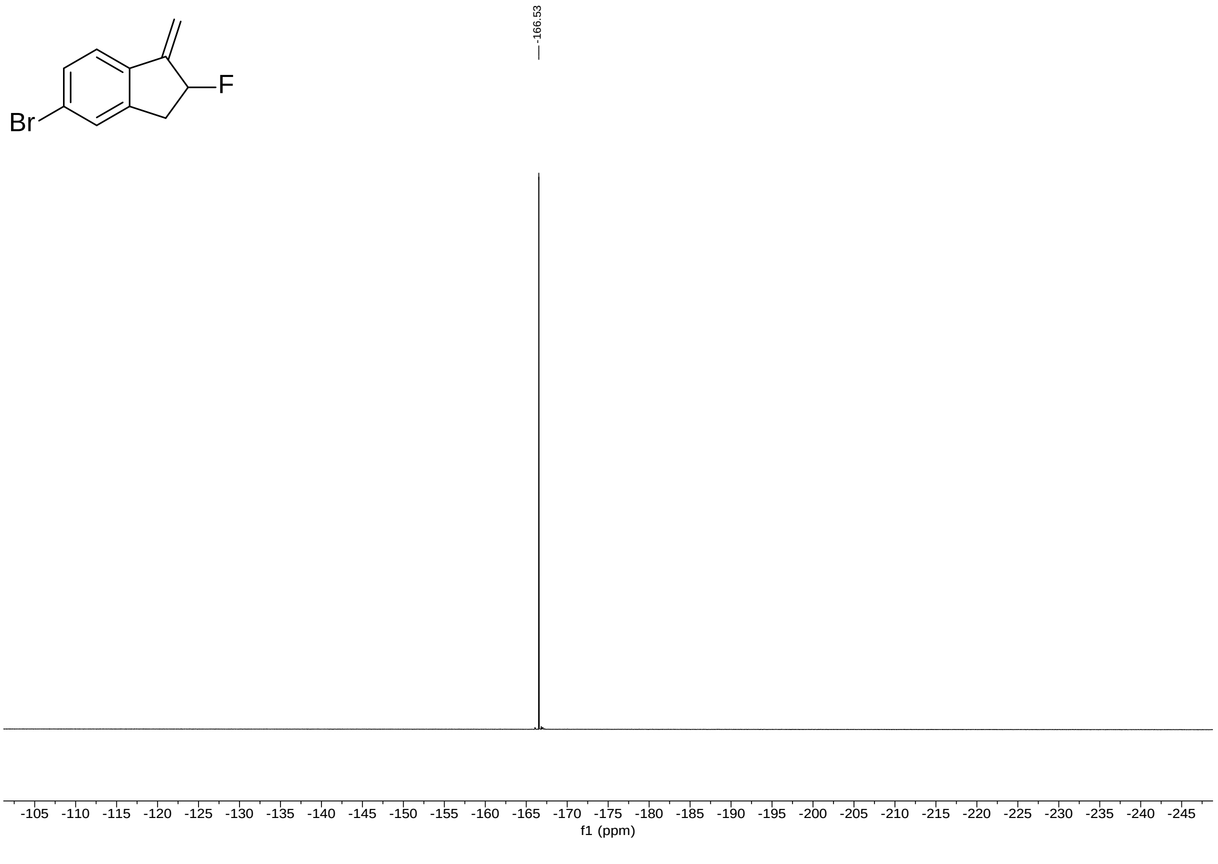

6-Bromo-2-fluoro-2,3-dihydro-1*H*-inden-1-one (S8)

^{1}H NMR (500 MHz, $CDCl_3$, 299 K)

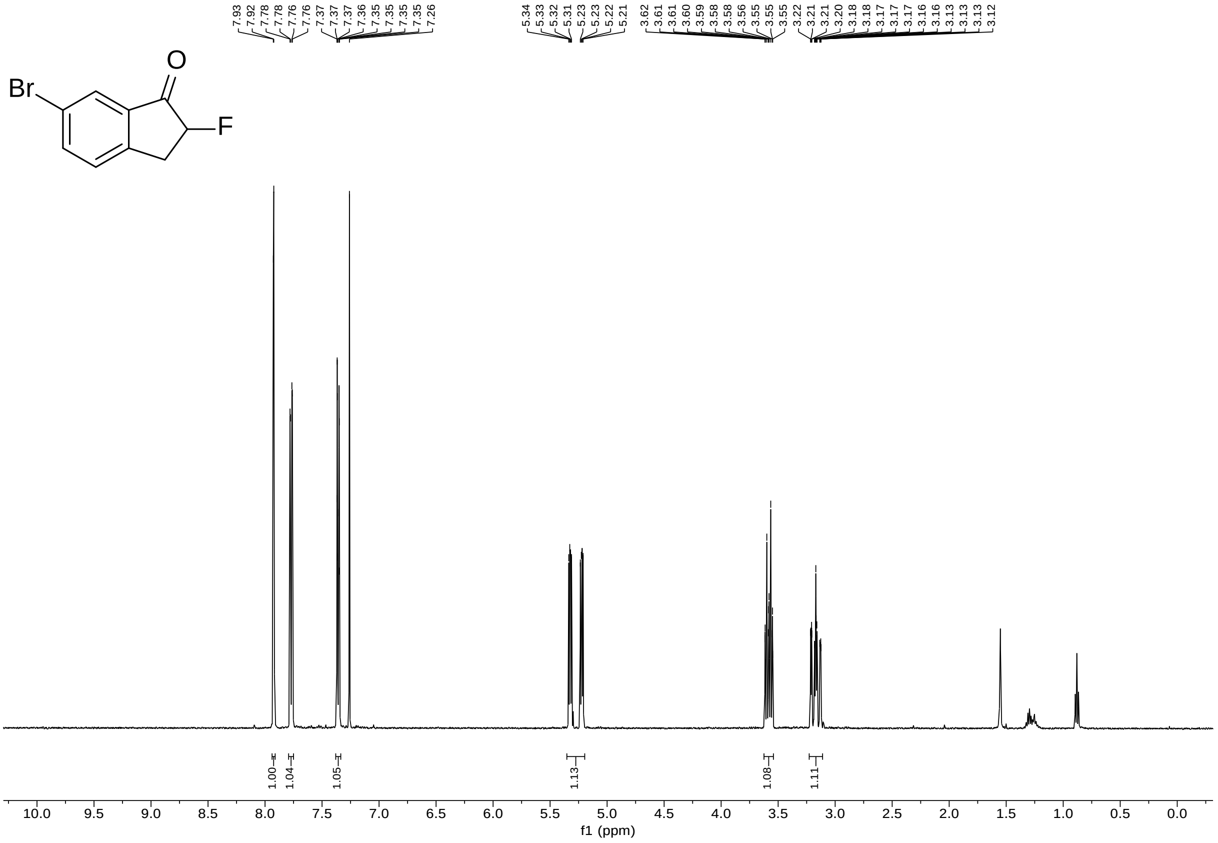

^{13}C NMR (126 MHz, $CDCl_3$, 299 K)

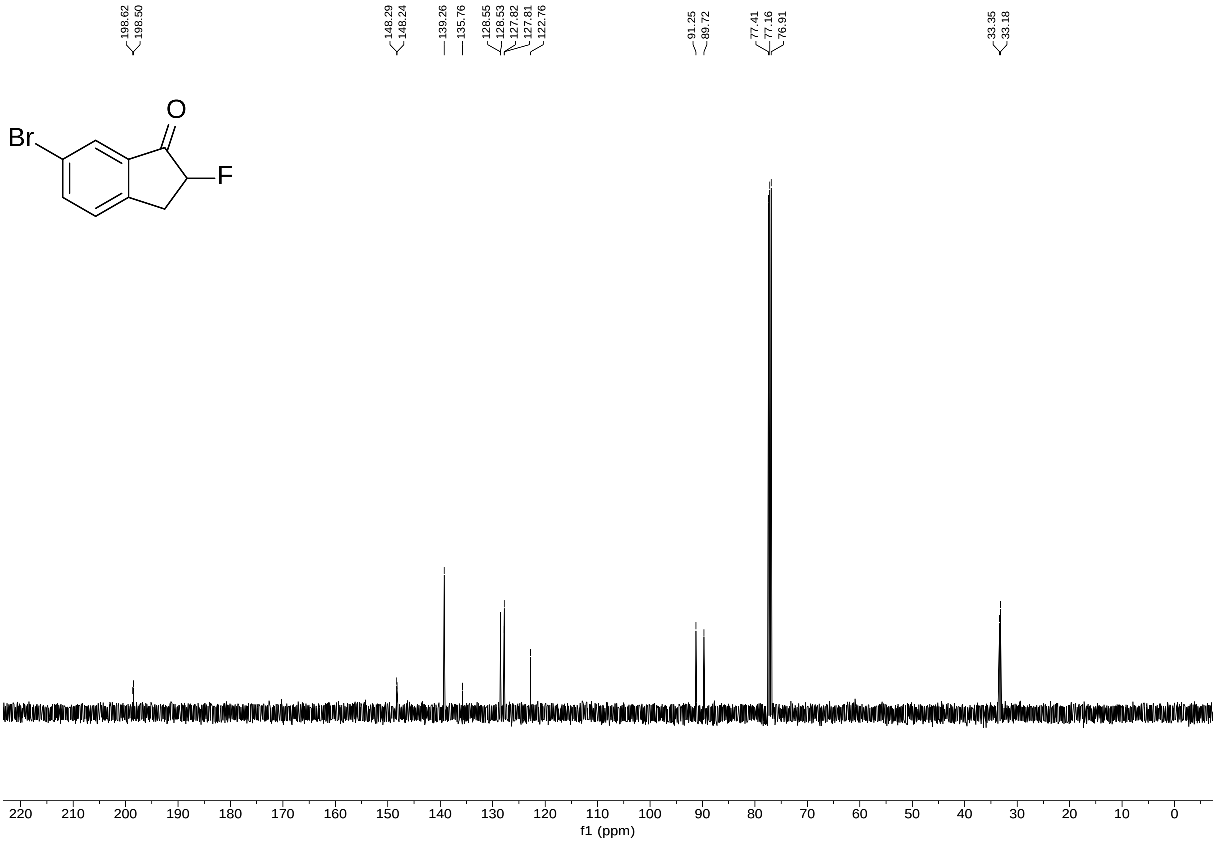

^{19}F NMR (470 MHz, $CDCl_3$, 299 K)

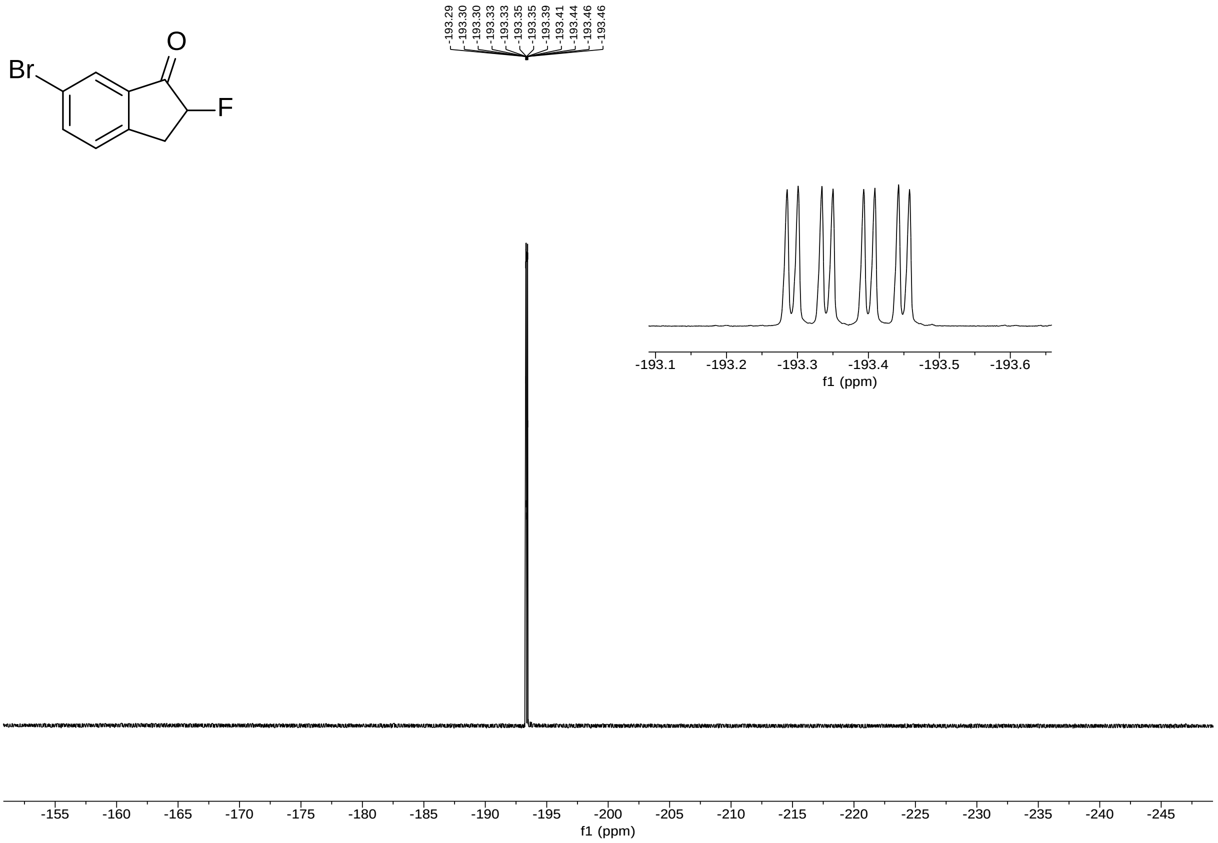

^{19}F{^{1}H} NMR (470 MHz, $CDCl_3$, 299 K)

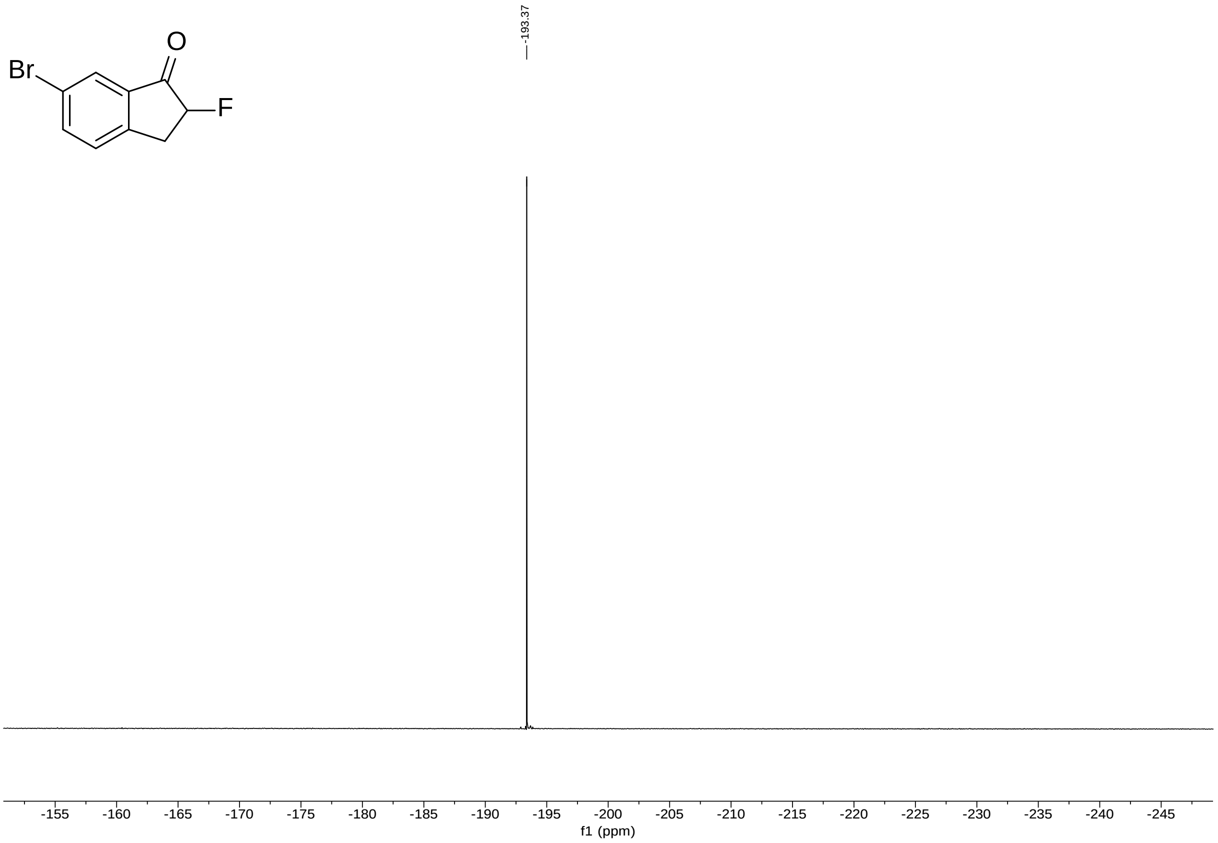

6-Bromo-2-fluoro-1-methylene-2,3-dihydro-1*H*-indene (S9)

^{1}H NMR (500 MHz, $CDCl_3$, 299 K)

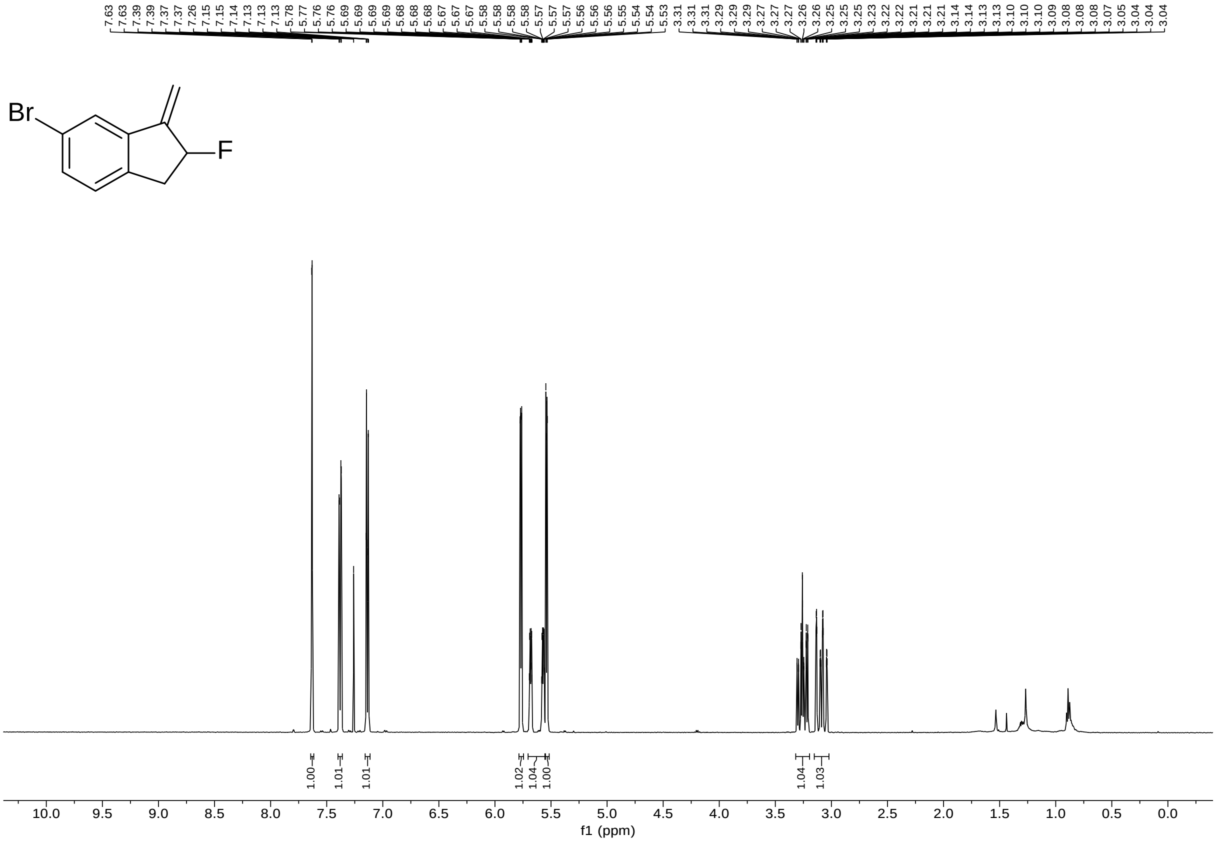

^{13}C NMR (126 MHz, $CDCl_3$, 299 K)

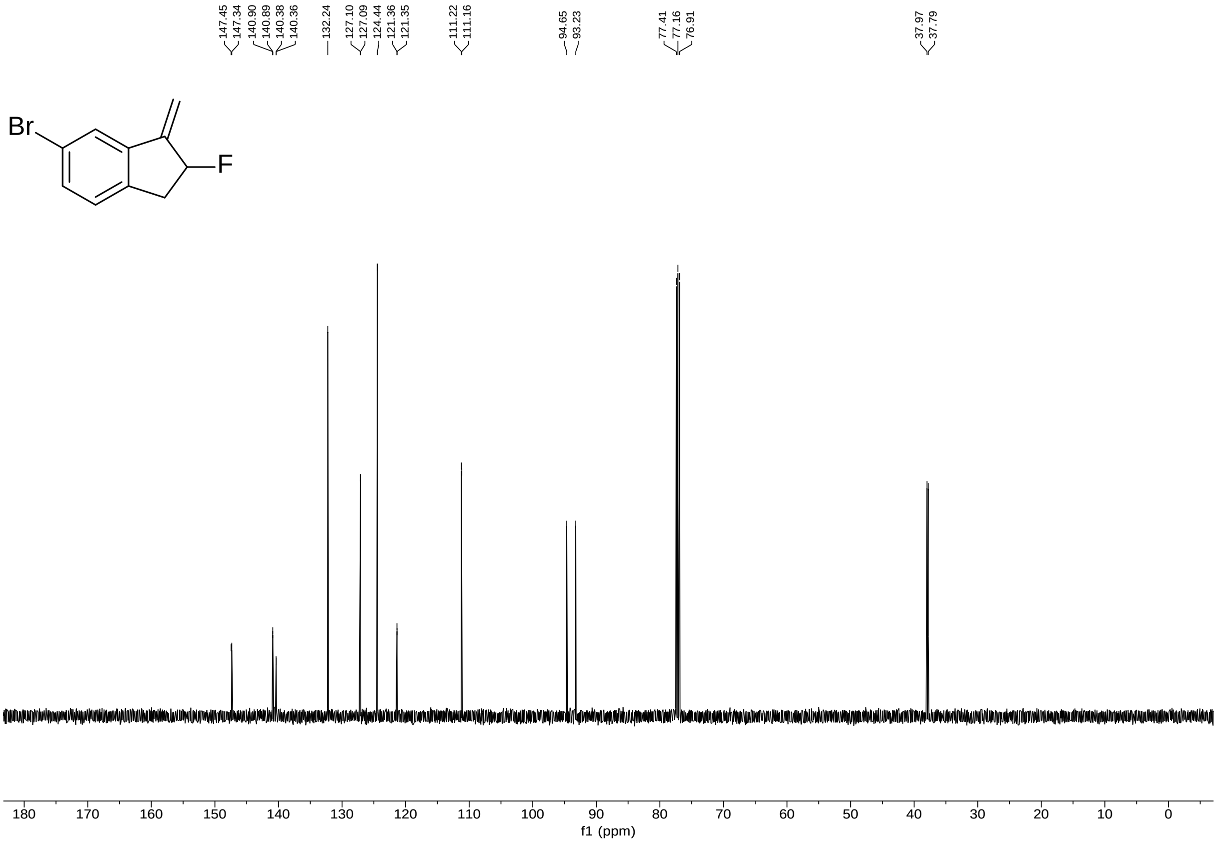

^{19}F NMR (470 MHz, $CDCl_3$, 299 K)

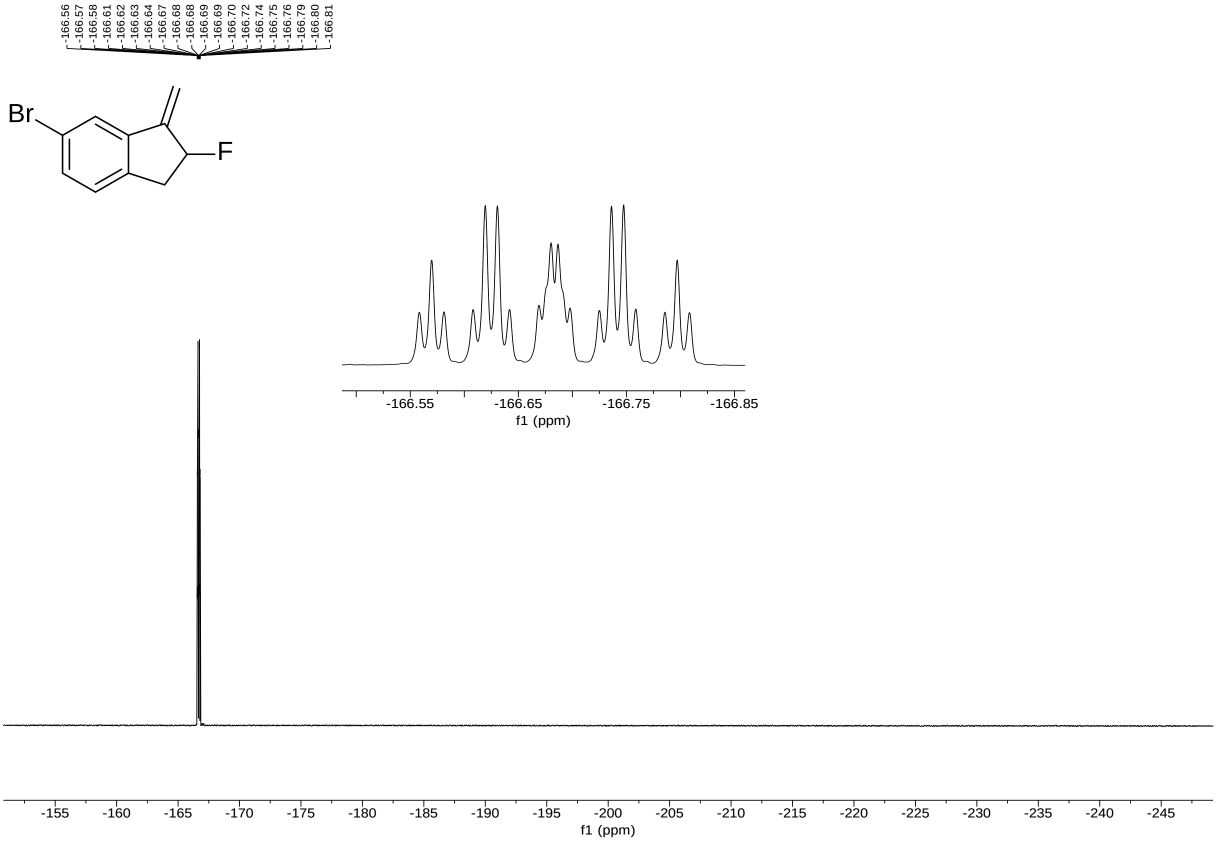

^{19}F{^{1}H} NMR (470 MHz, $CDCl_3$, 299 K)

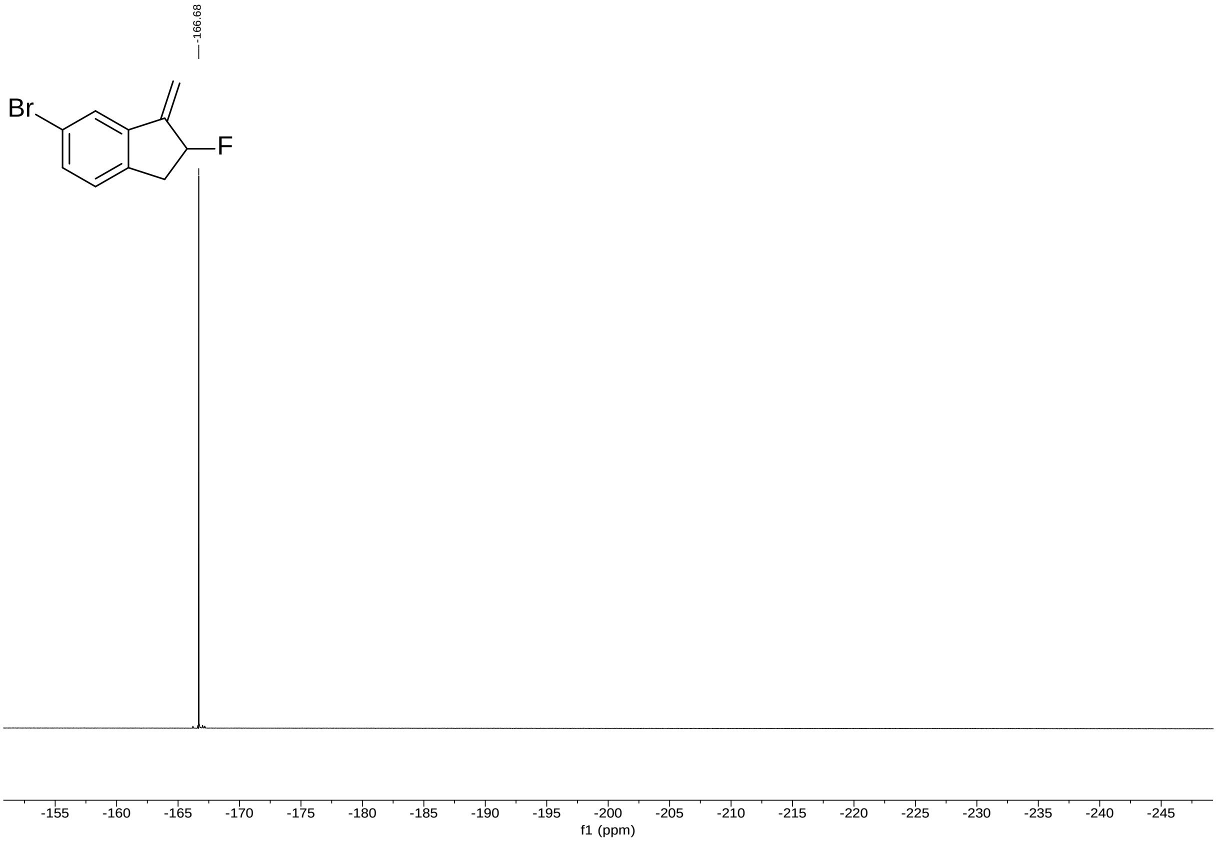

4-Bromo-2-fluoro-1,1-dimethoxy-2,3-dihydro-1H-indene (S10)

^{1}H NMR (599 MHz, CD_2Cl_2, 299 K)

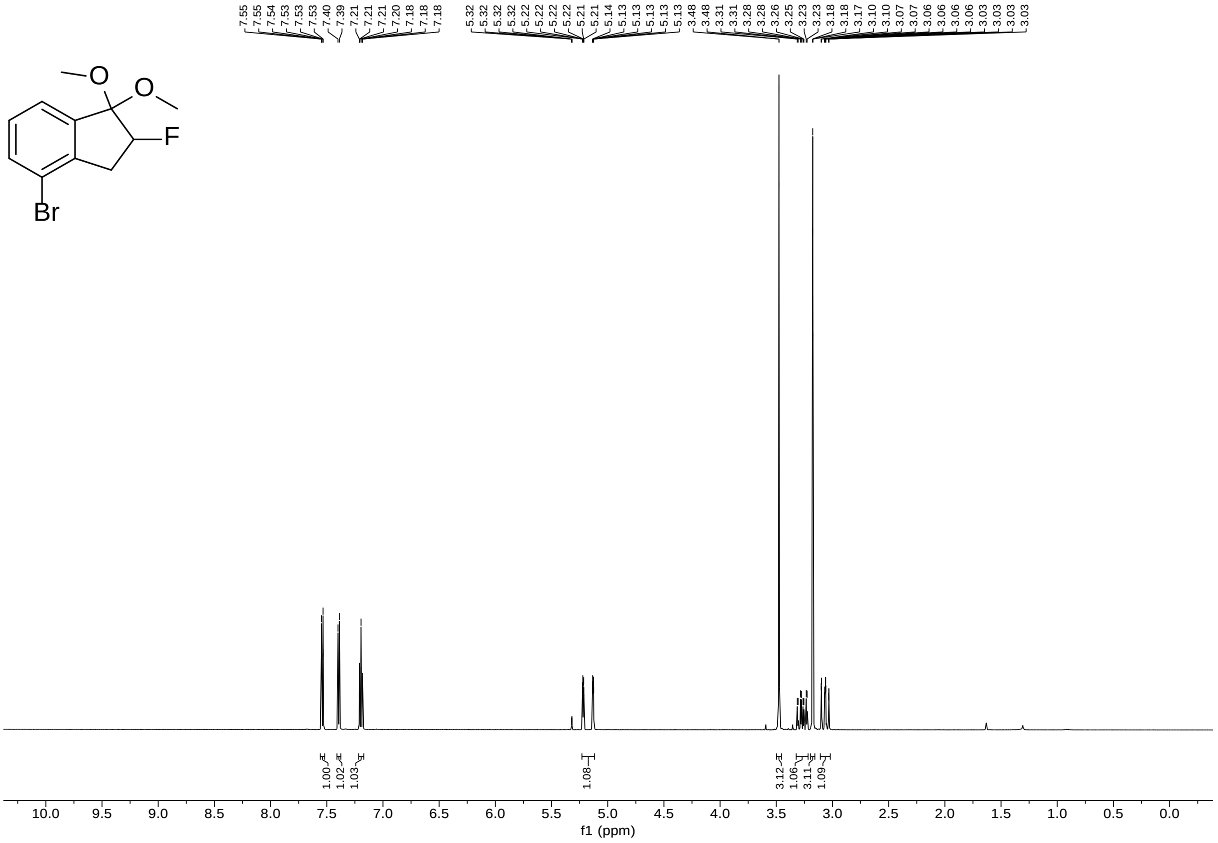

^{13}C NMR (151 MHz, CD_2Cl_2, 299 K)

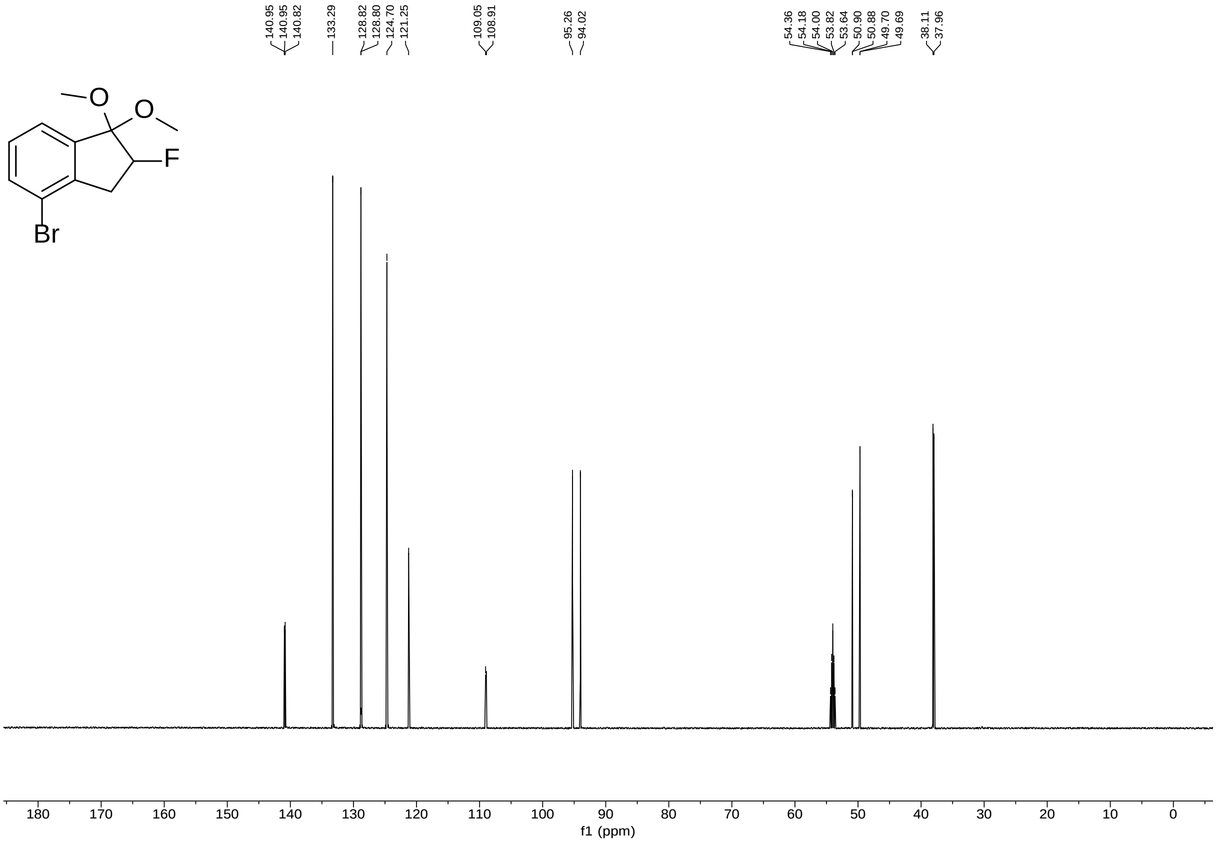

^{19}F NMR (564 MHz, CD_2Cl_2, 299 K)

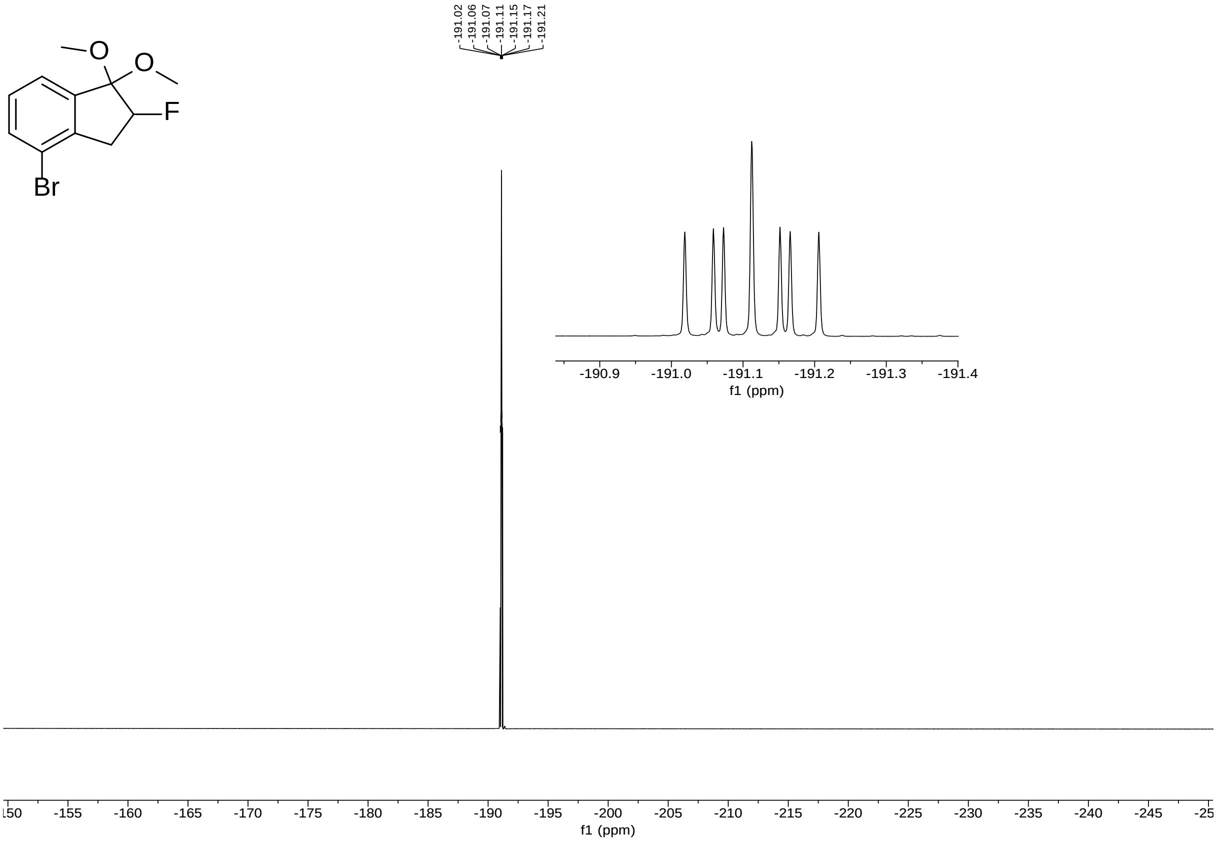

^{19}F{^{1}H} NMR (564 MHz, CD_2Cl_2, 299 K)

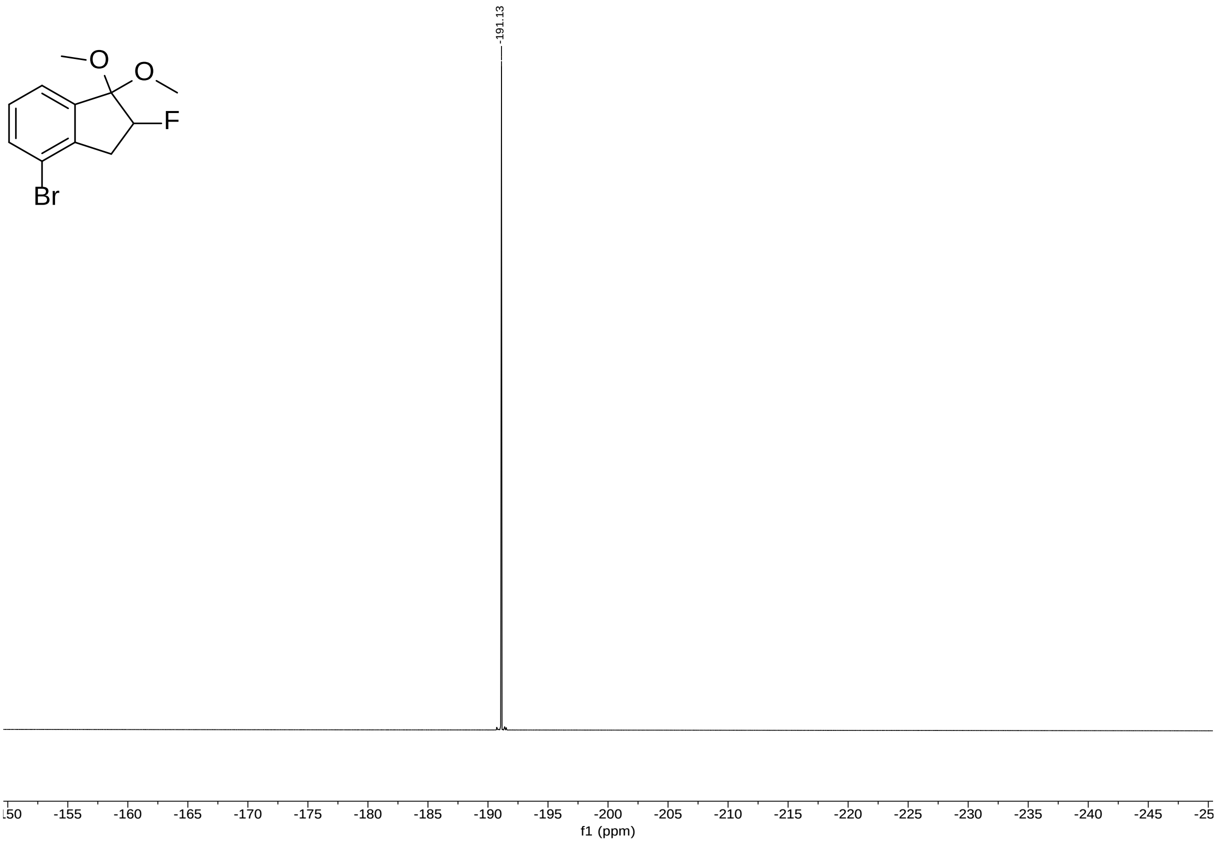

4-Bromo-2-fluoro-1-methylene-2,3-dihydro-1*H*-indene (S11)

^{1}H NMR (599 MHz, $CDCl_3$, 299 K)

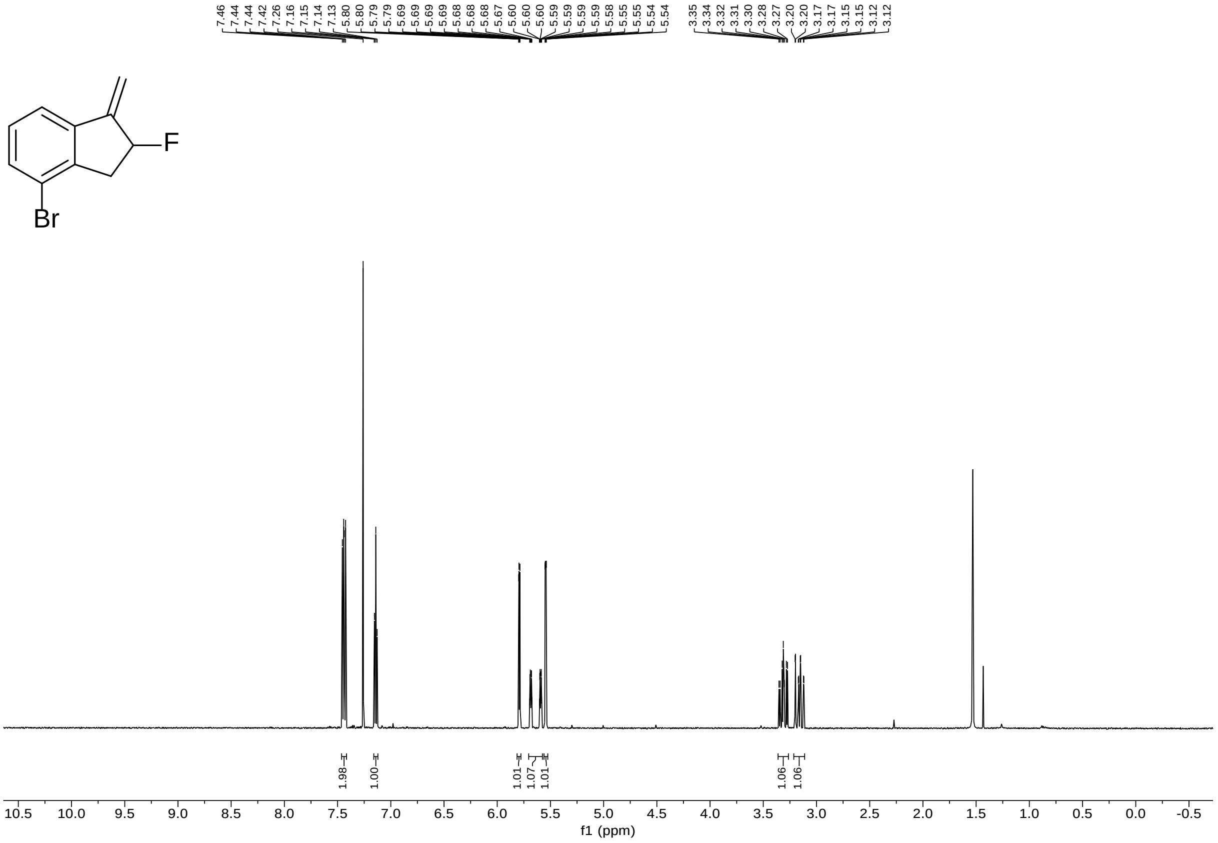

^{13}C NMR (151 MHz, $CDCl_3$, 299 K)

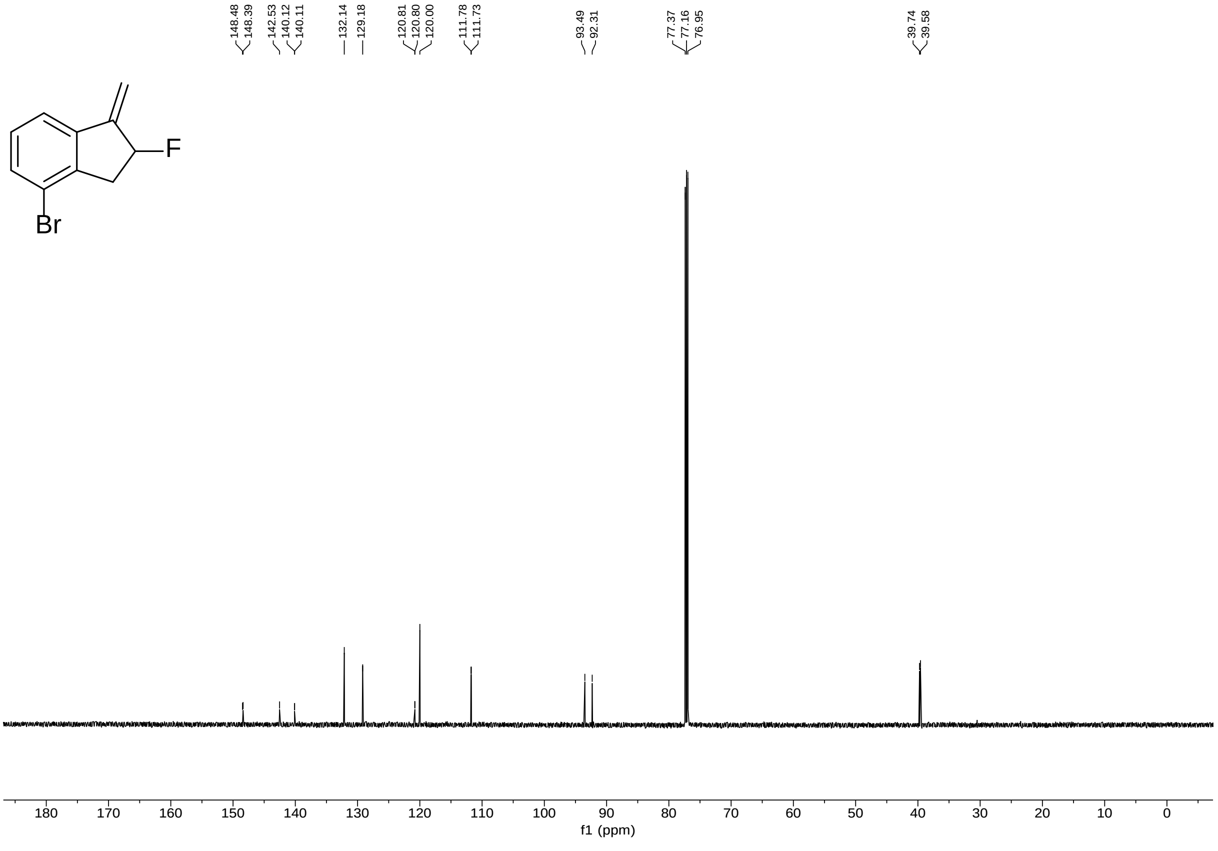

^{19}F NMR (564 MHz, $CDCl_3$, 299 K)

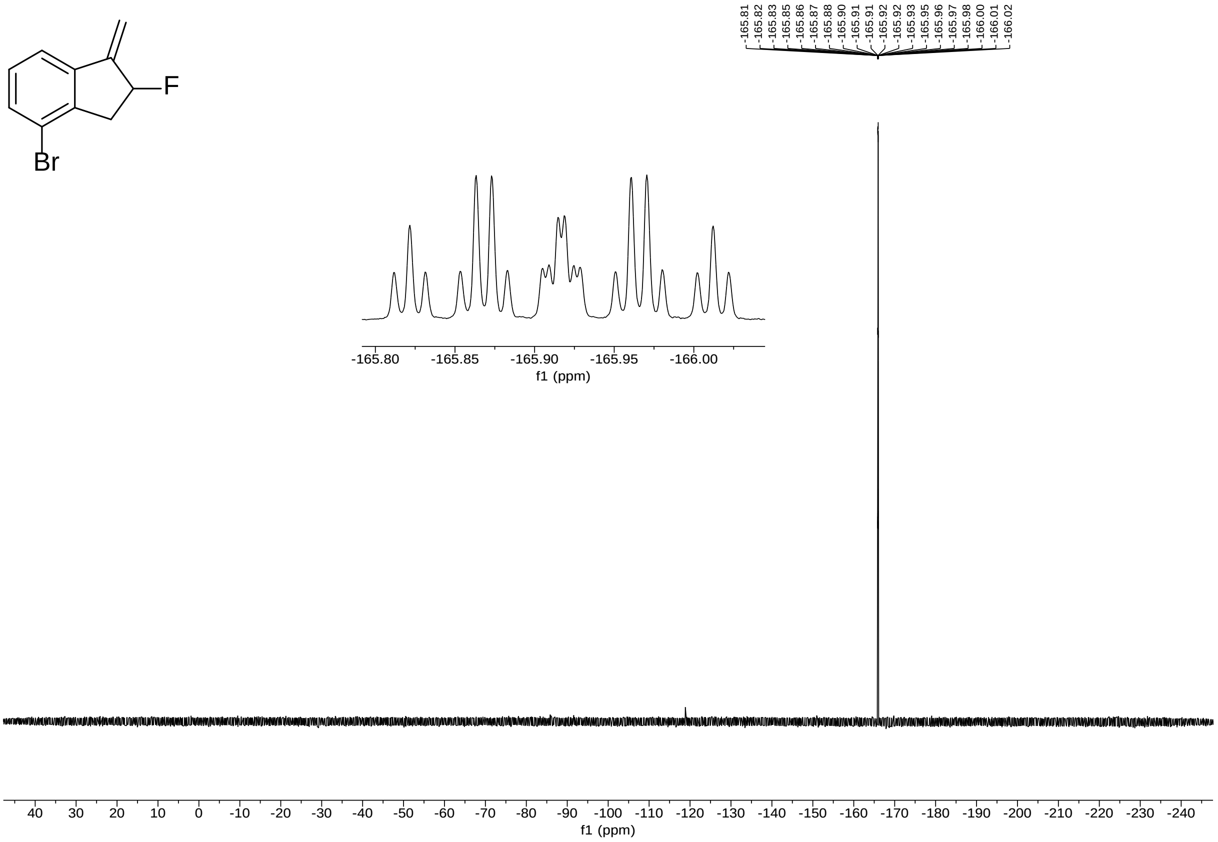

^{19}F{^{1}H} NMR (564 MHz, $CDCl_3$, 299 K)

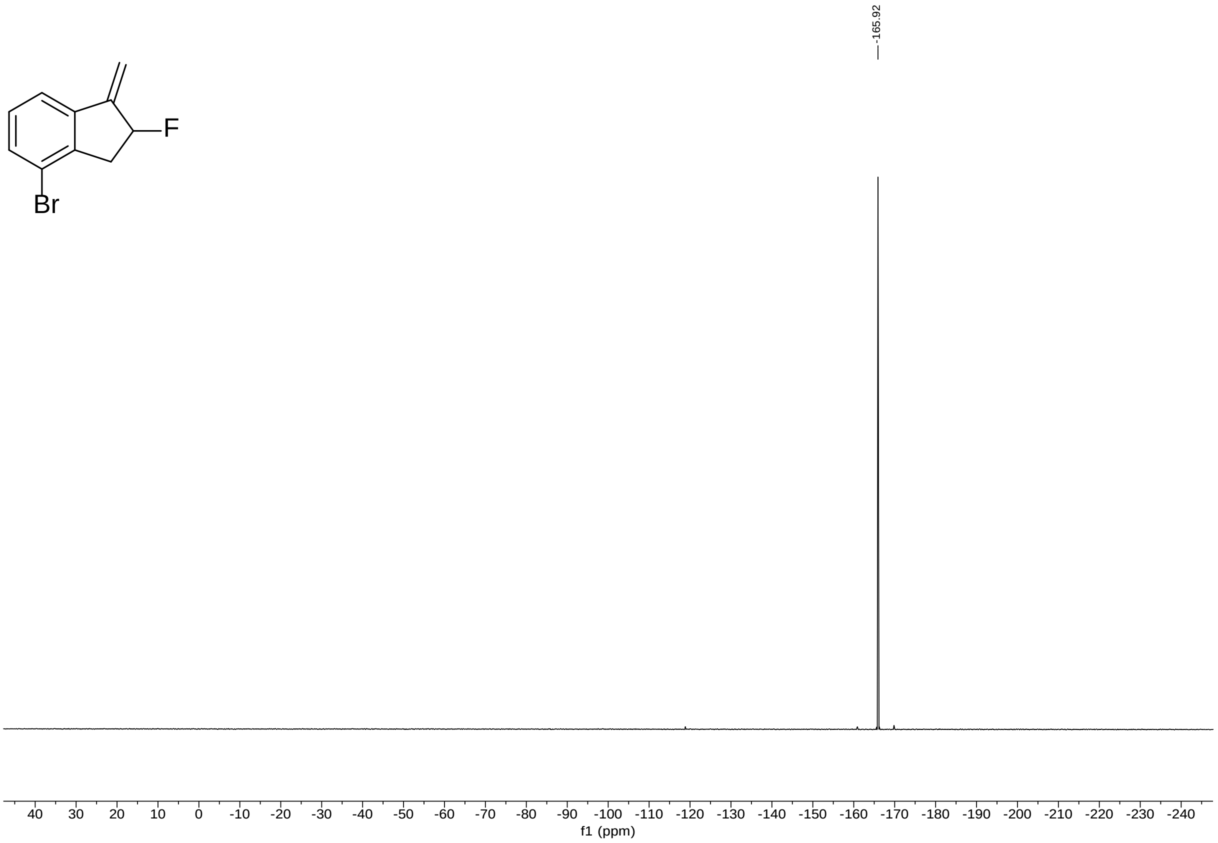

2-Fluoro-5-methyl-1-methylene-2,3-dihydro-1*H*-indene (S13)

^{1}H NMR (500 MHz, $CDCl_3$, 299 K)

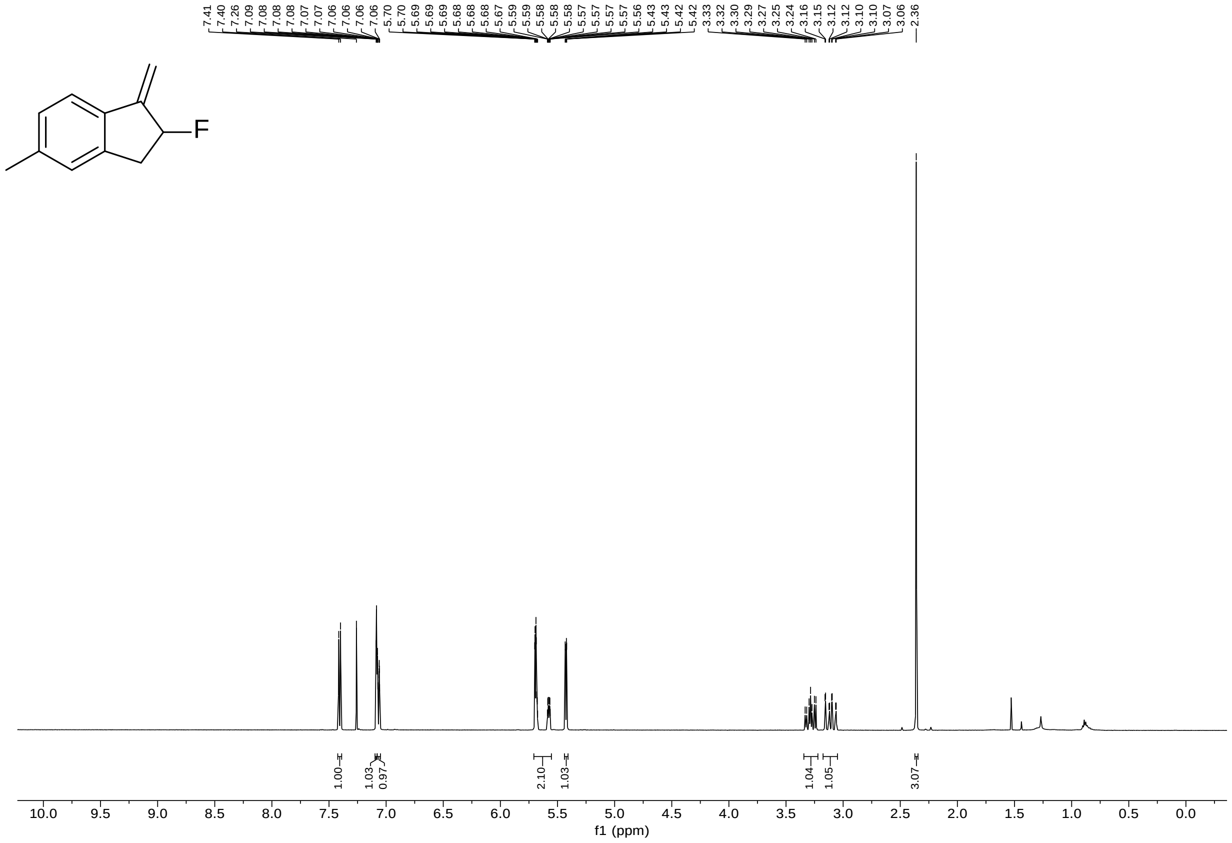

^{13}C NMR (126 MHz, $CDCl_3$, 299 K)

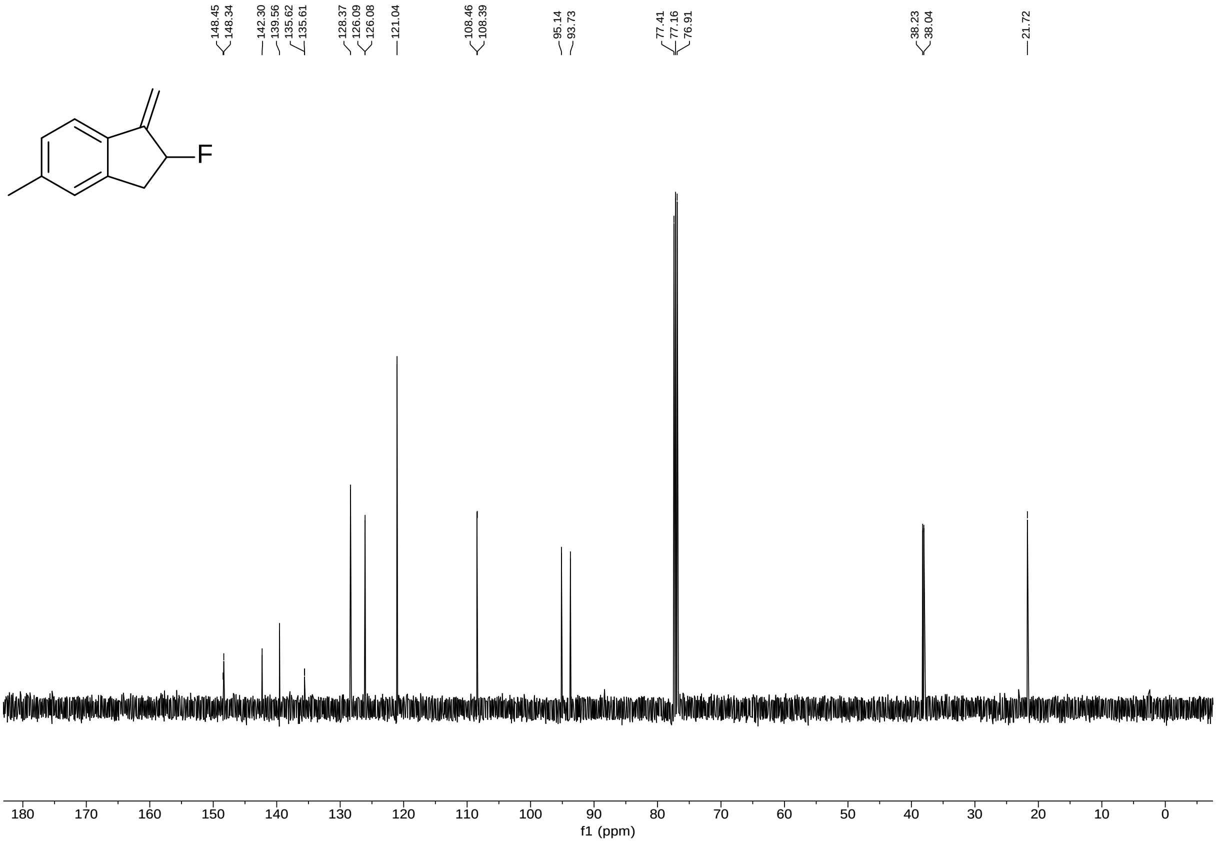

^{19}F NMR (470 MHz, $CDCl_3$, 299 K)

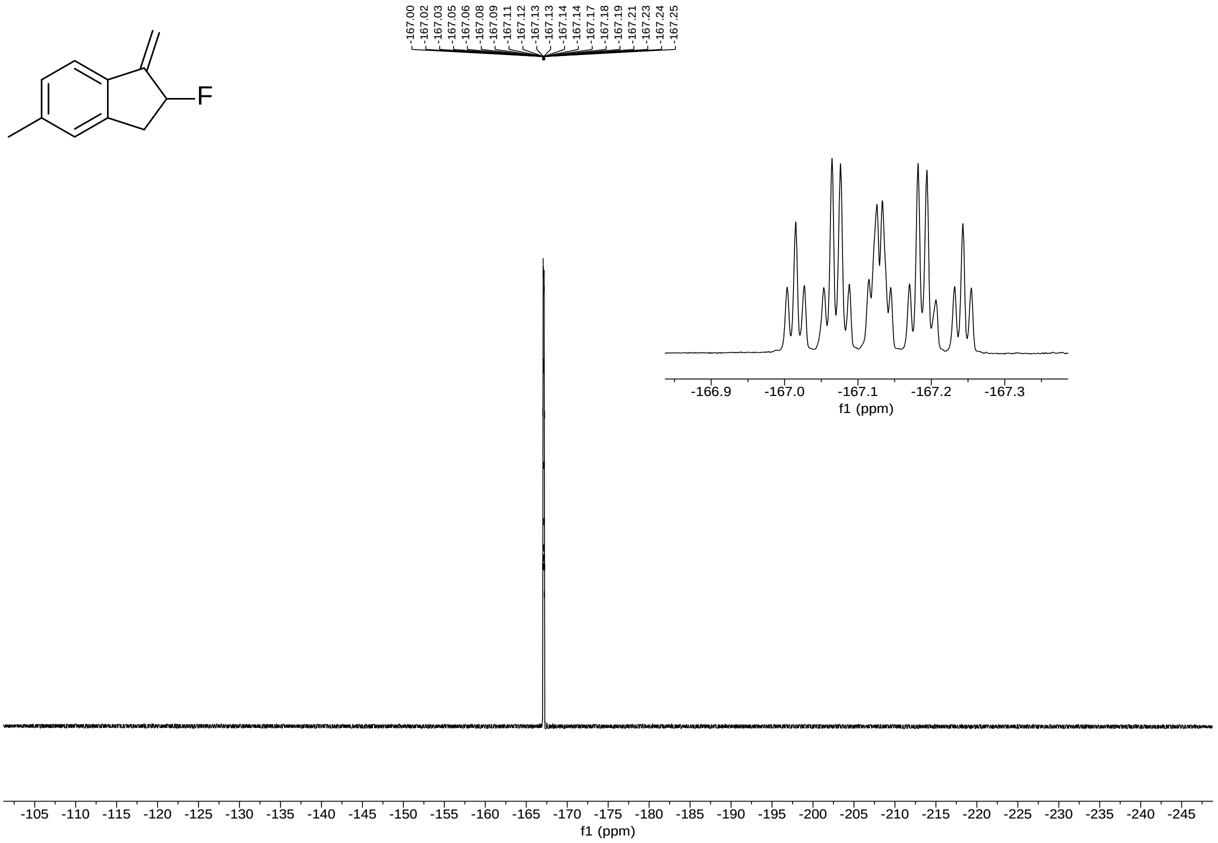

^{19}F{^{1}H} NMR (470 MHz, $CDCl_3$, 299 K)

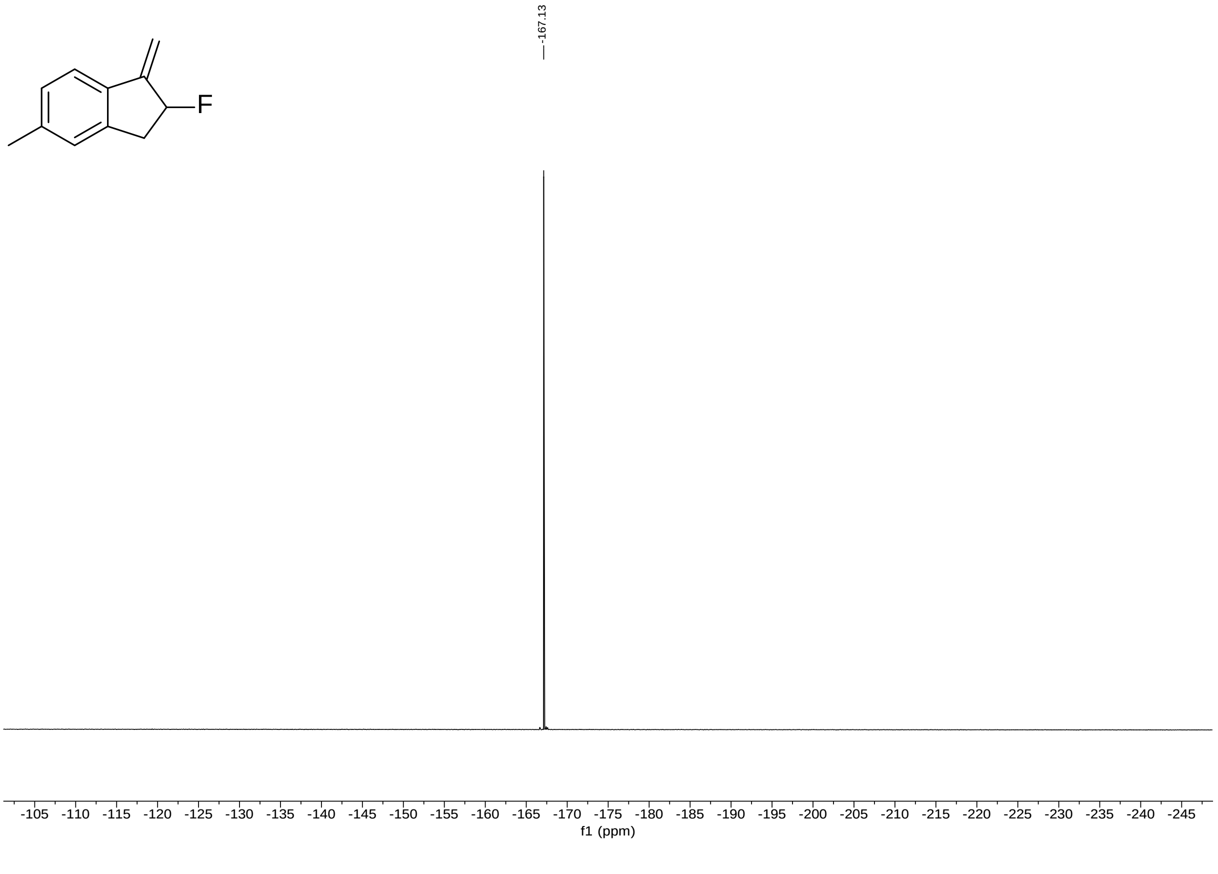

2-Fluoro-1,1-dimethoxy-2,3-dihydro-1*H*-inden-5-yltrifluoromethanesulfonate (S15)

^{1}H NMR (599 MHz, CD_2Cl_2, 299 K)

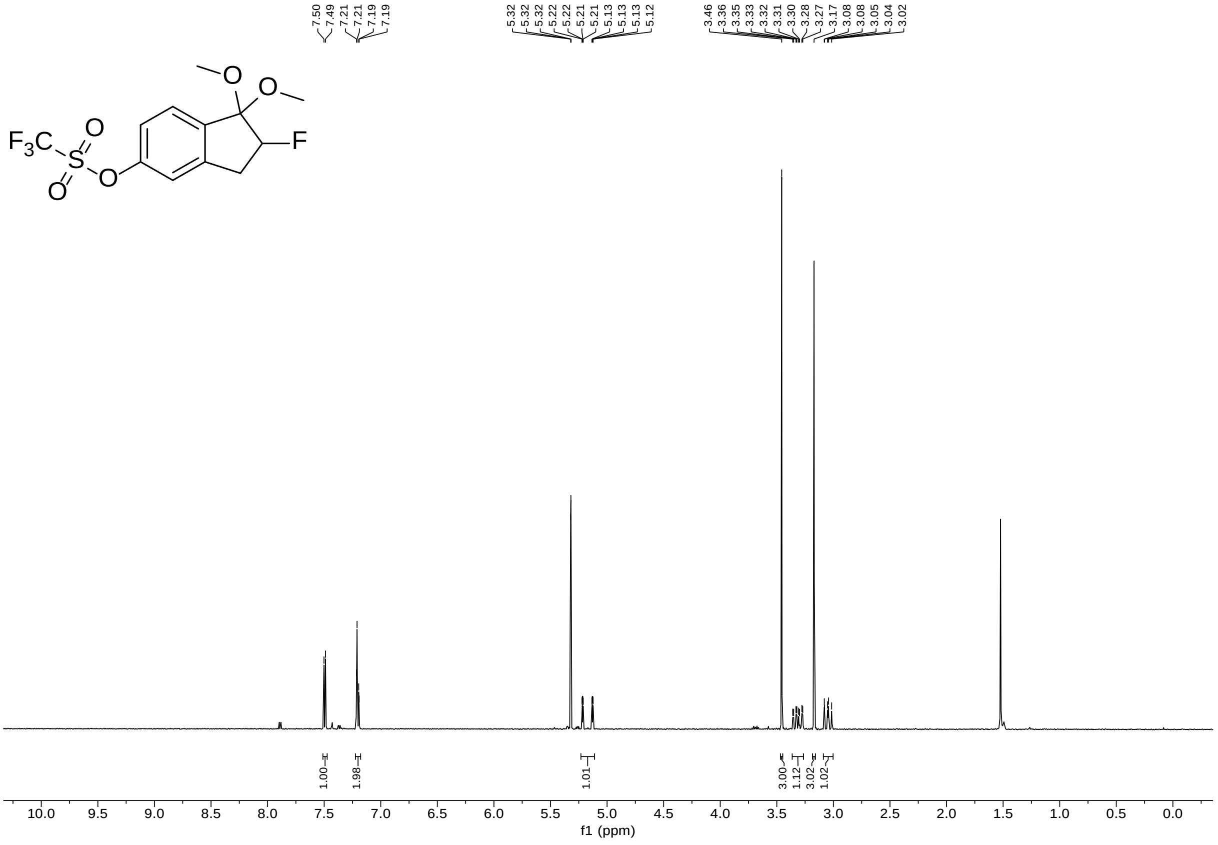

^{13}C NMR (151 MHz, CD_2Cl_2, 299 K)

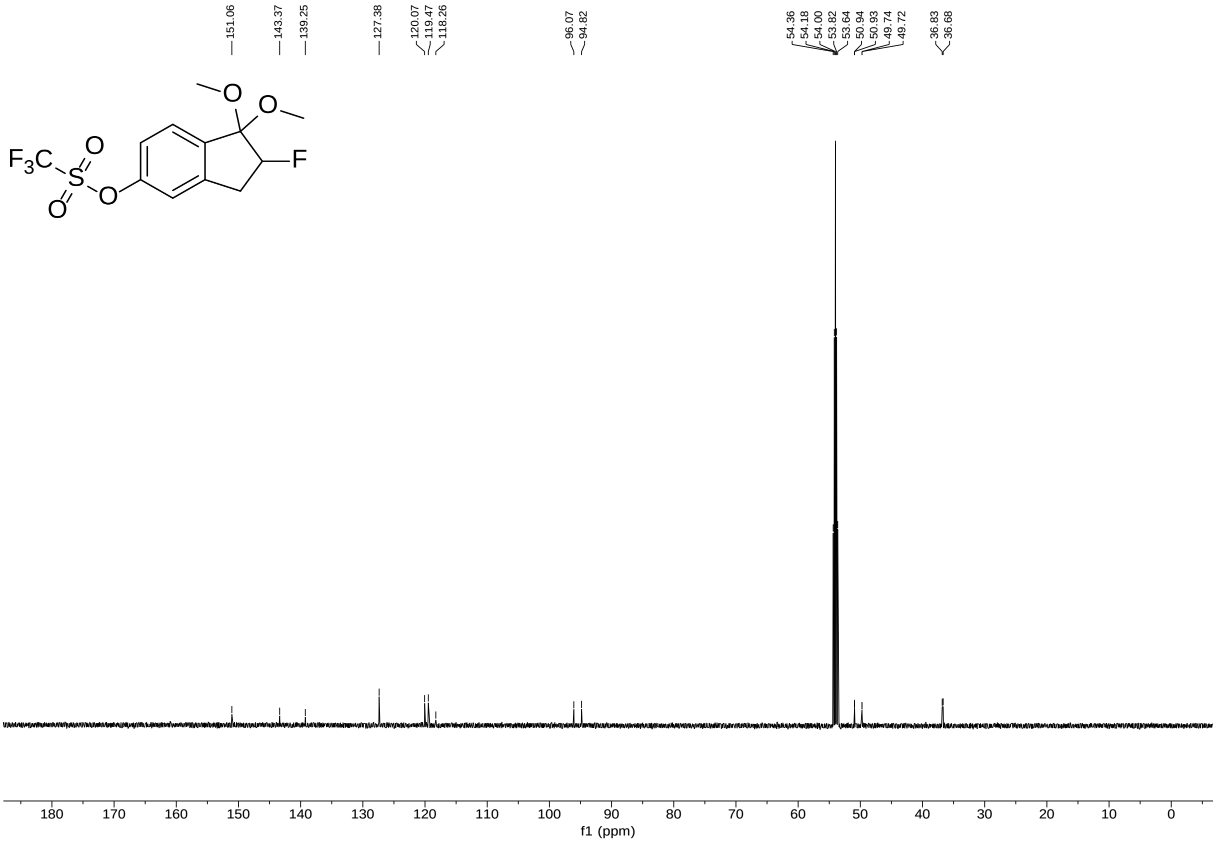

^{19}F NMR (564 MHz, CD_2Cl_2, 299 K)

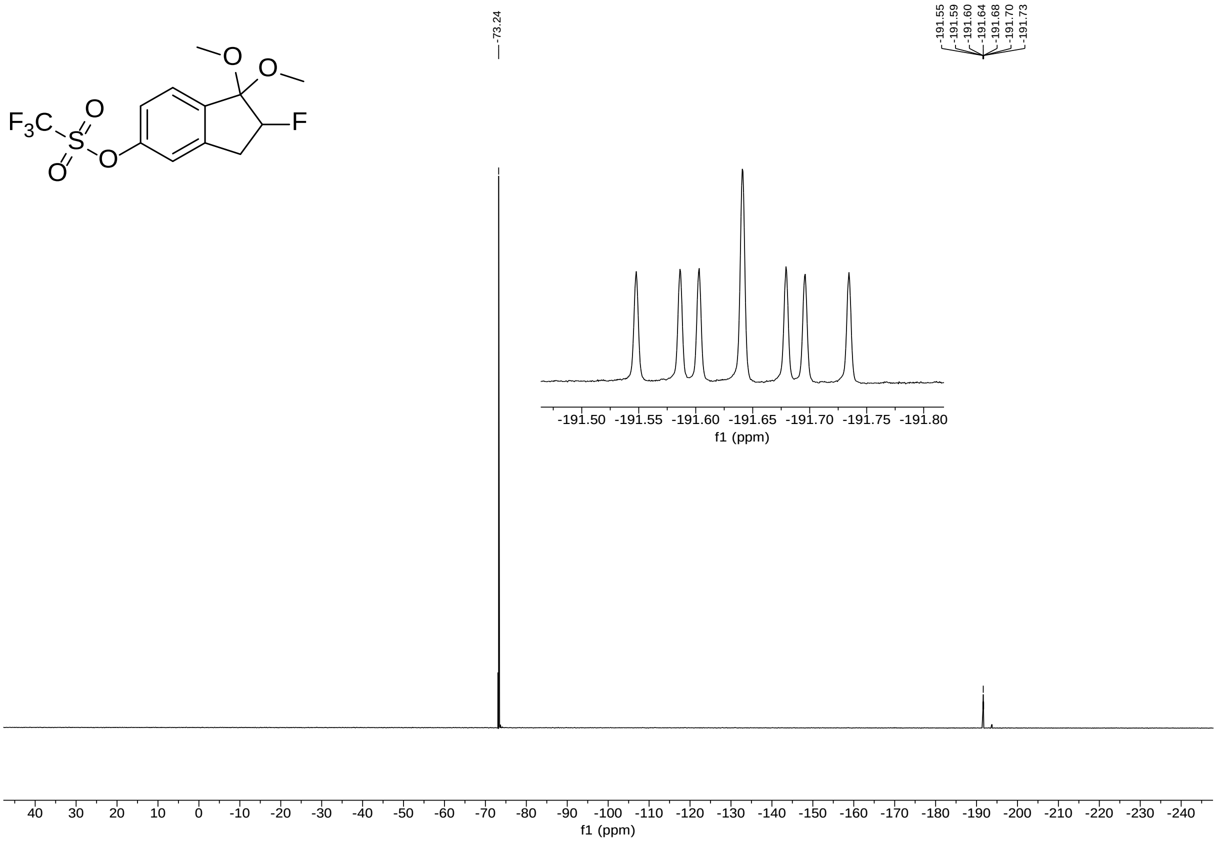

^{19}F{^{1}H} NMR (564 MHz, CD_2Cl_2, 299 K)

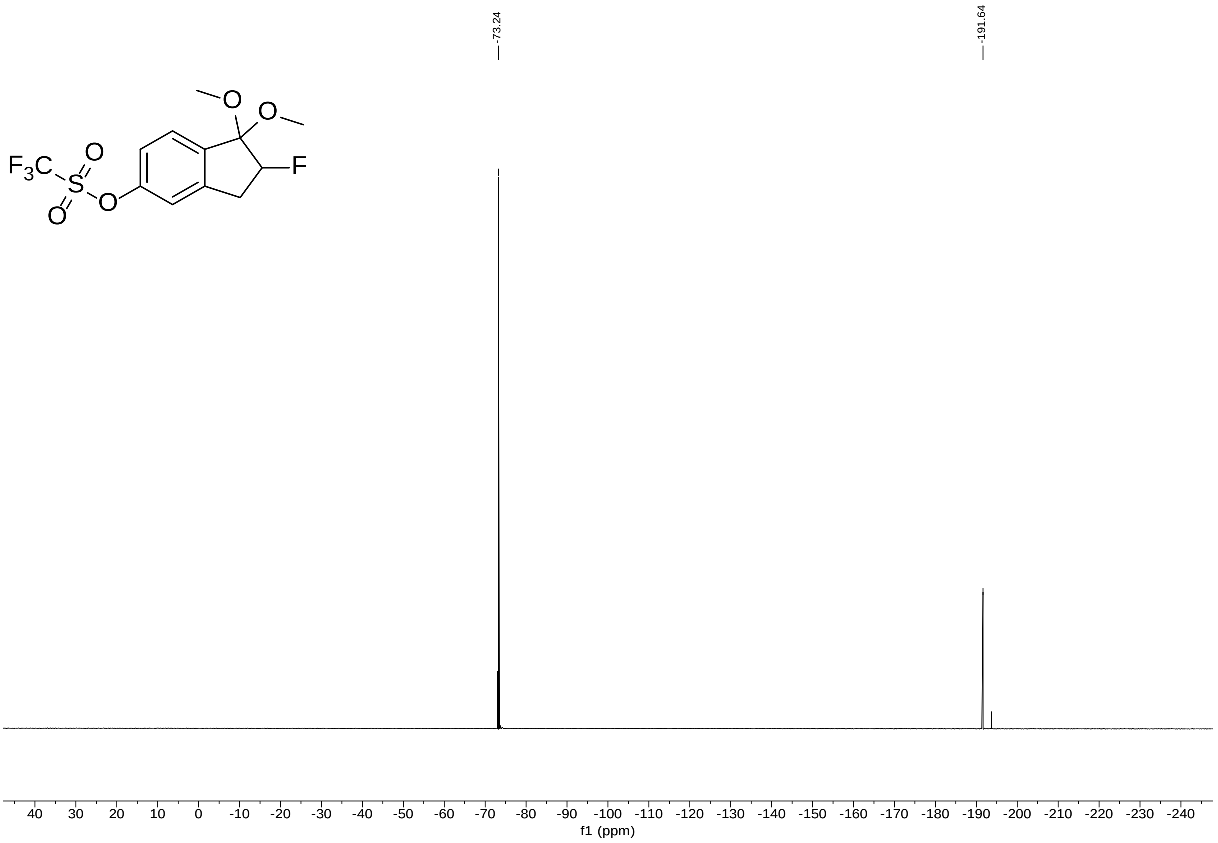

2-Fluoro-1-oxo-2,3-dihydro-1*H*-inden-5-yl trifluoromethanesulfonate (S16)

^{1}H NMR (599 MHz, CD_2Cl_2, 299 K)

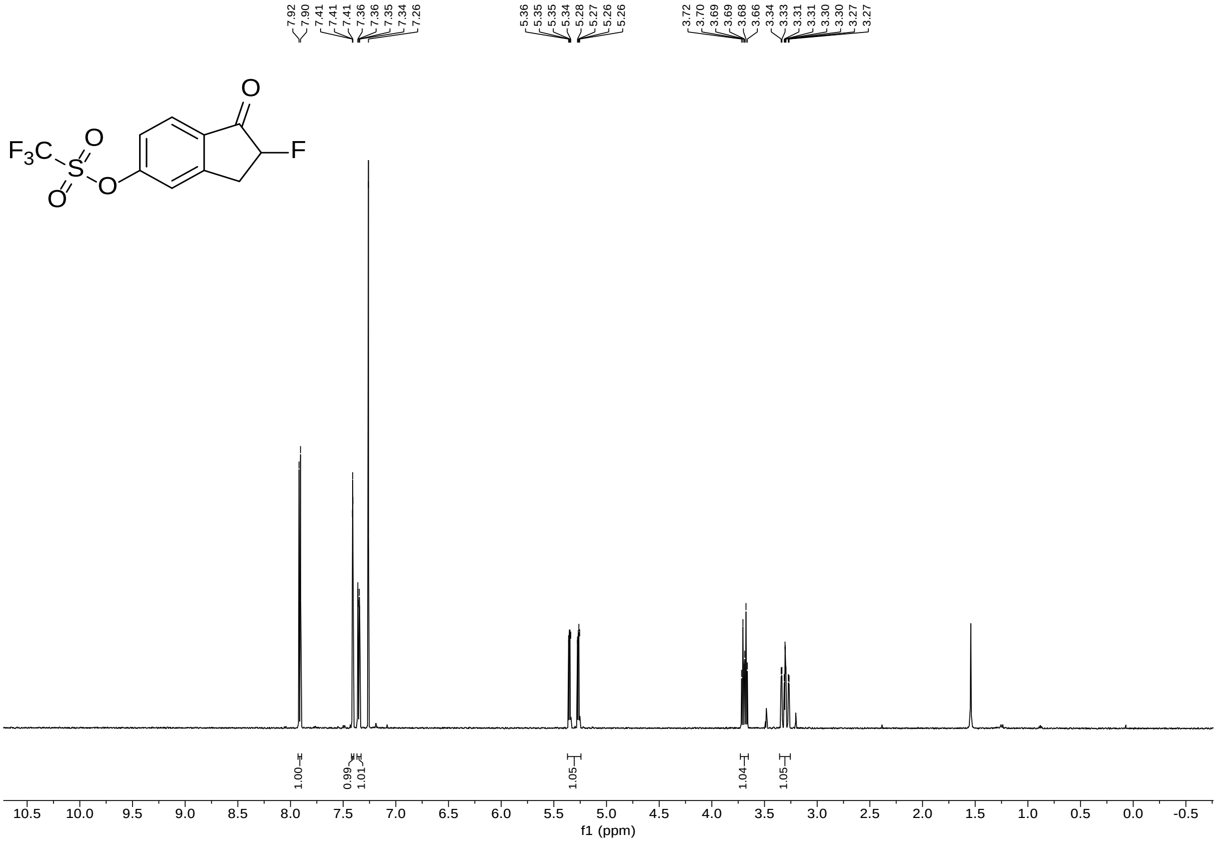

^{13}C NMR (151 MHz, $CDCl_3$, 299 K)

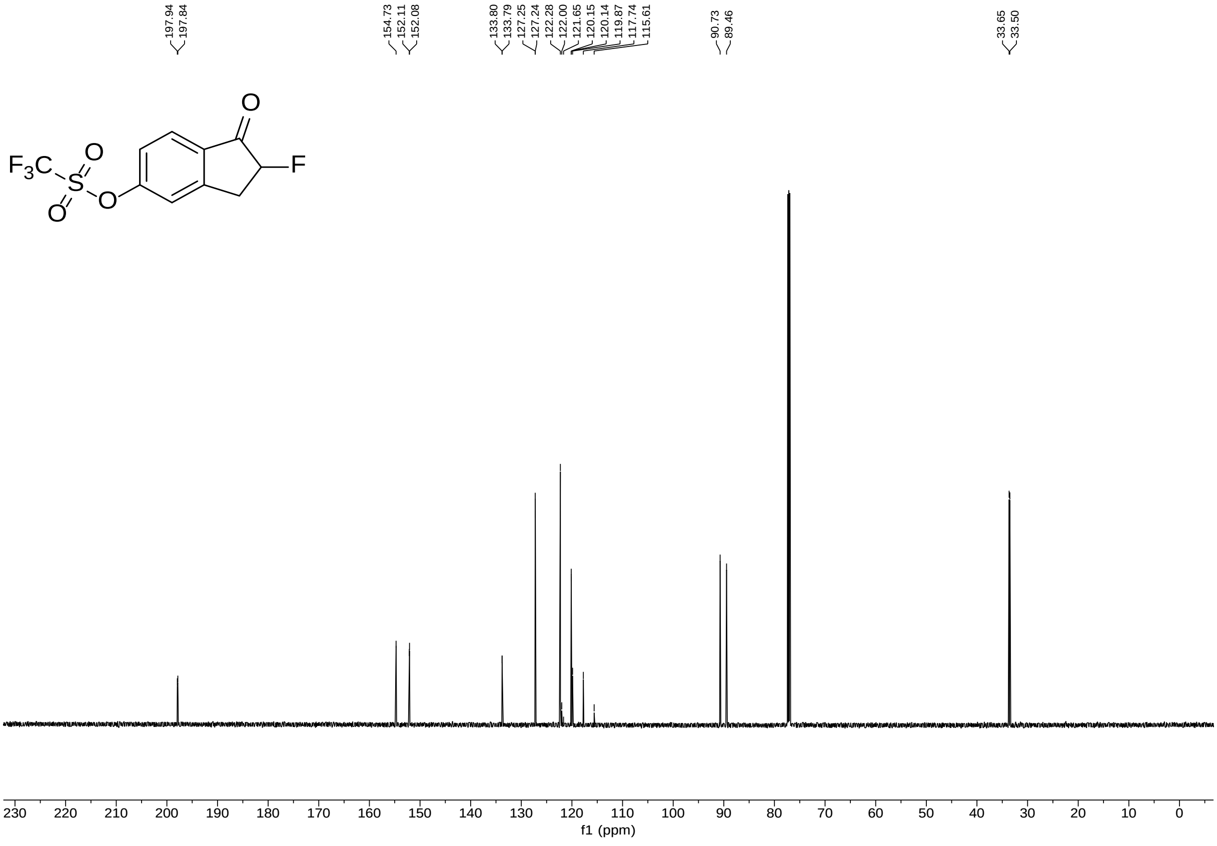

^{19}F NMR (564 MHz, $CDCl_3$, 299 K)

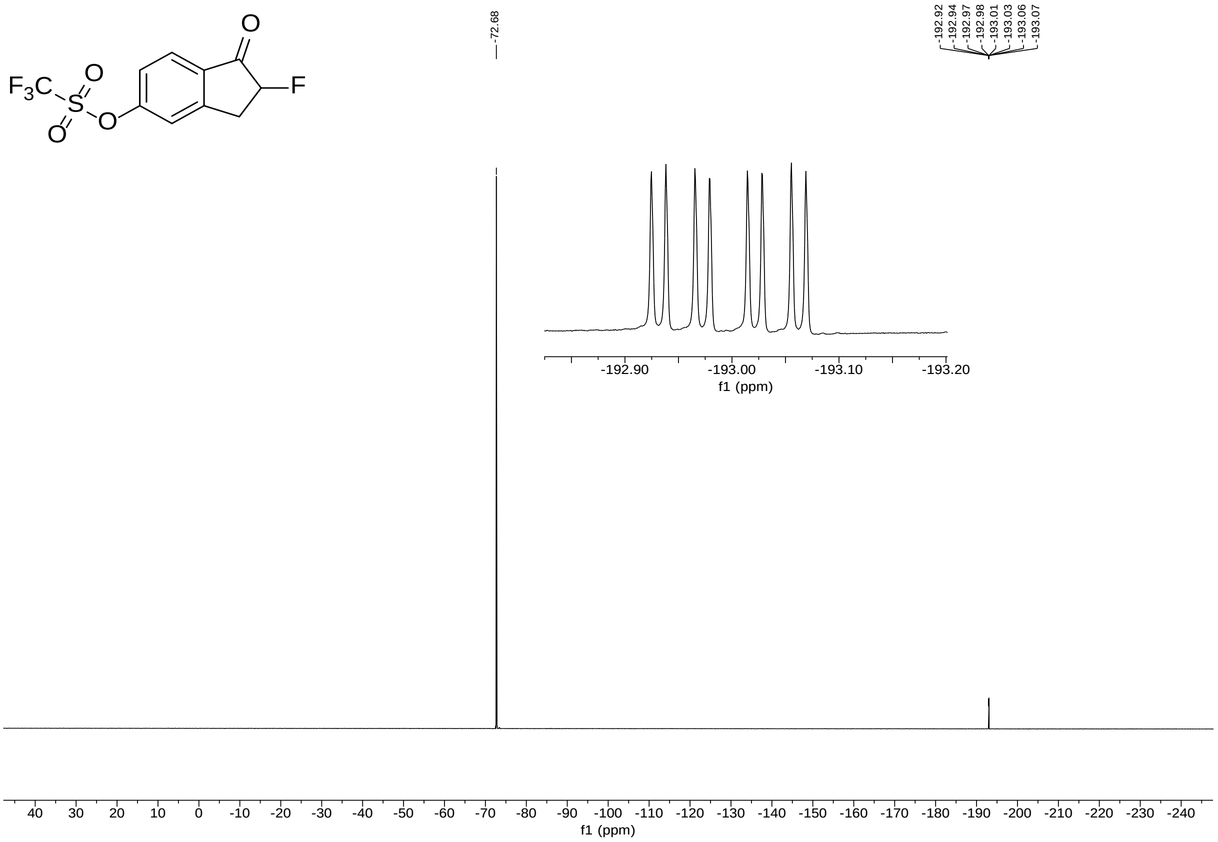

^{19}F{^{1}H} NMR (564 MHz, $CDCl_3$, 299 K)

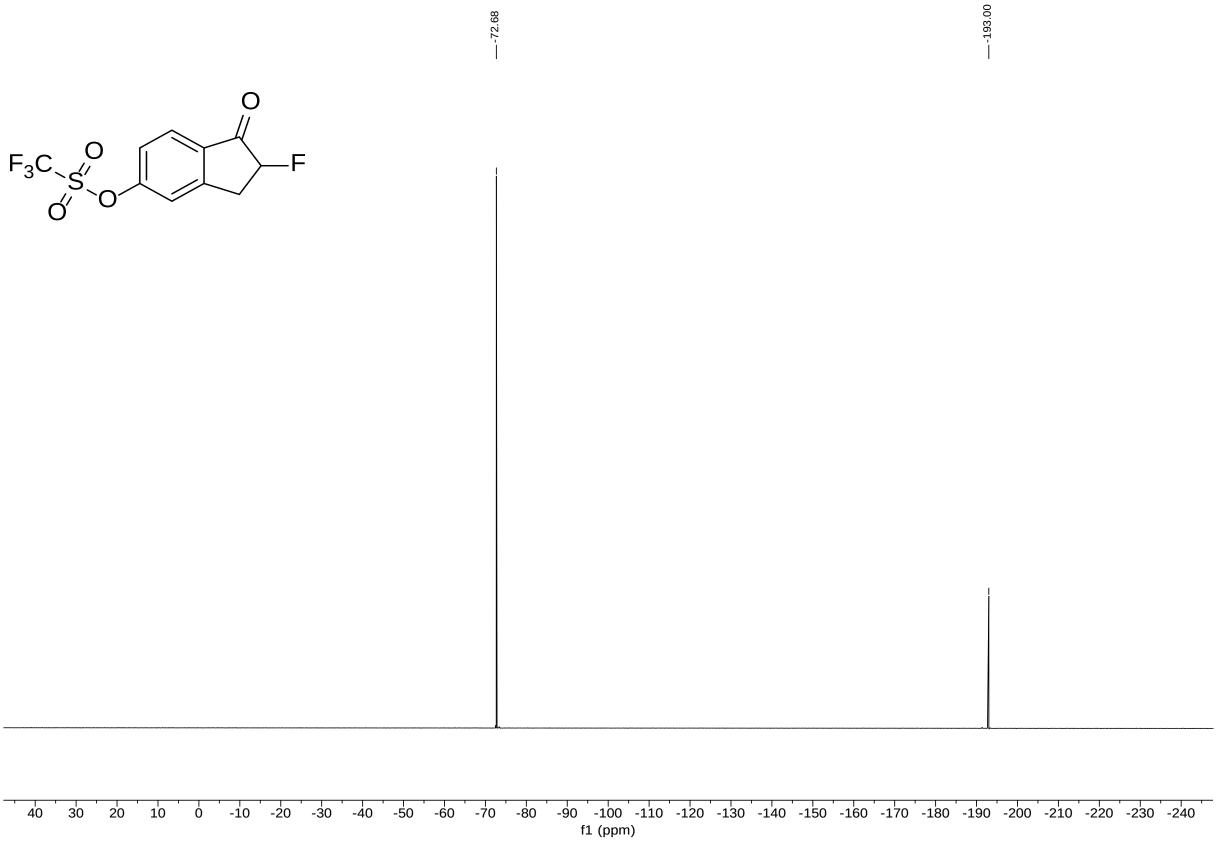

2-Fluoro-1-methylene-2,3-dihydro-1*H*-inden-5-yl trifluoromethanesulfonate (S17)

^{1}H NMR (599 MHz, $CDCl_3$, 299 K)

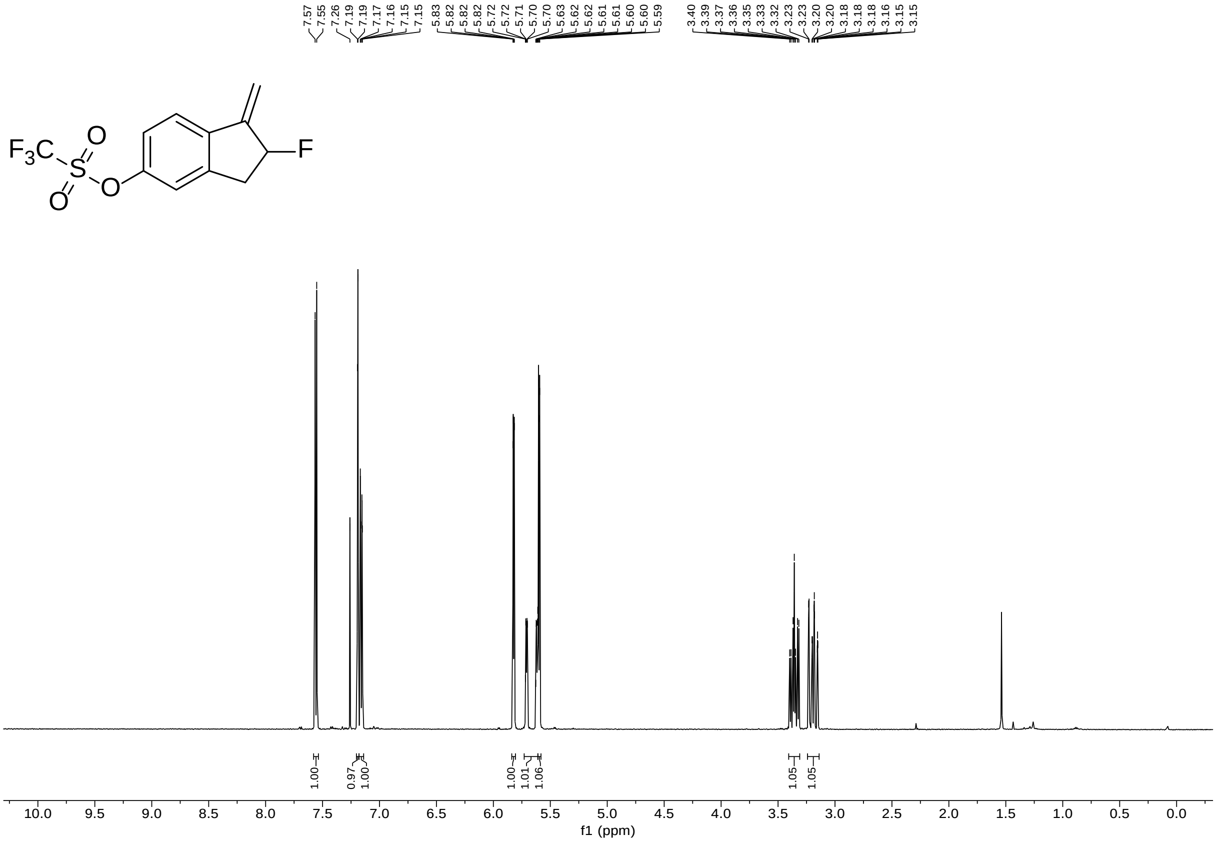

^{13}C NMR (151 MHz, $CDCl_3$, 299 K)

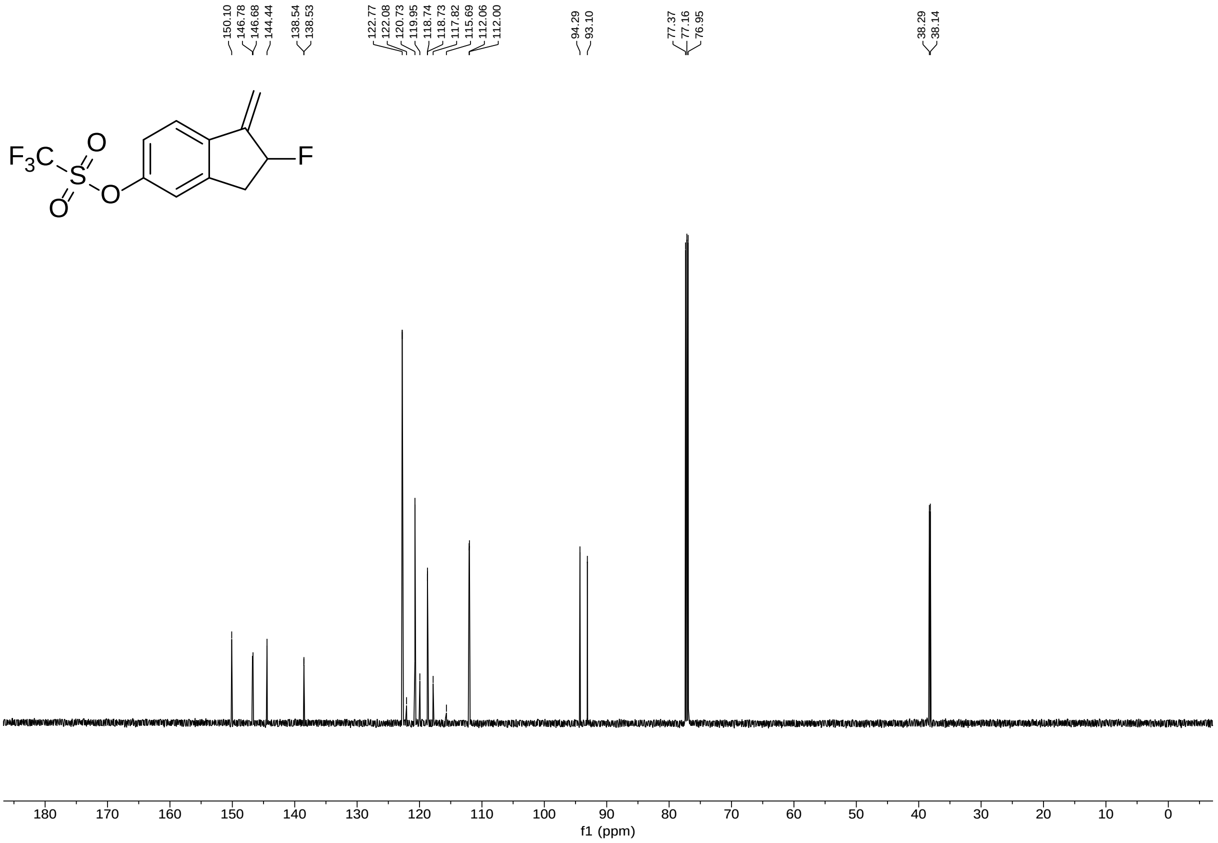

^{19}F NMR (564 MHz, $CDCl_3$, 299 K)

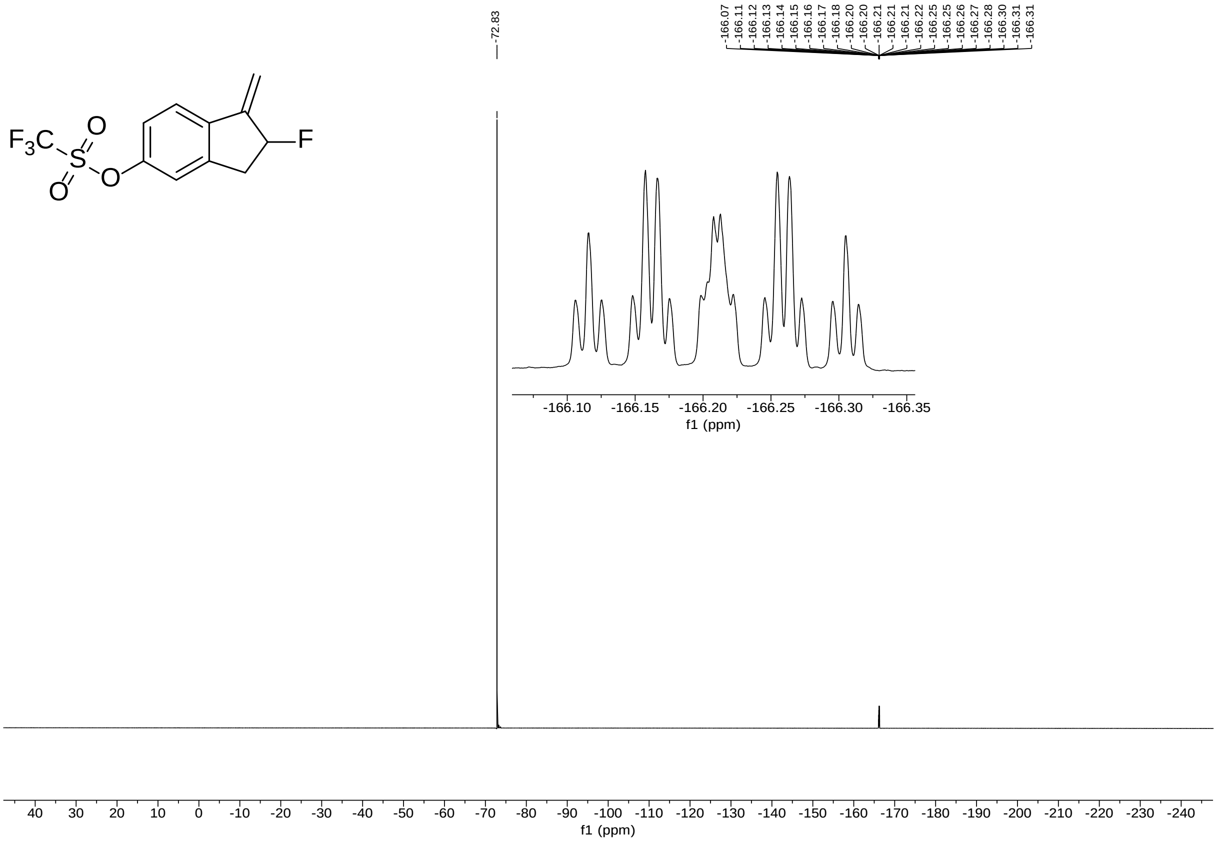

^{19}F{^{1}H} NMR (564 MHz, $CDCl_3$, 299 K)

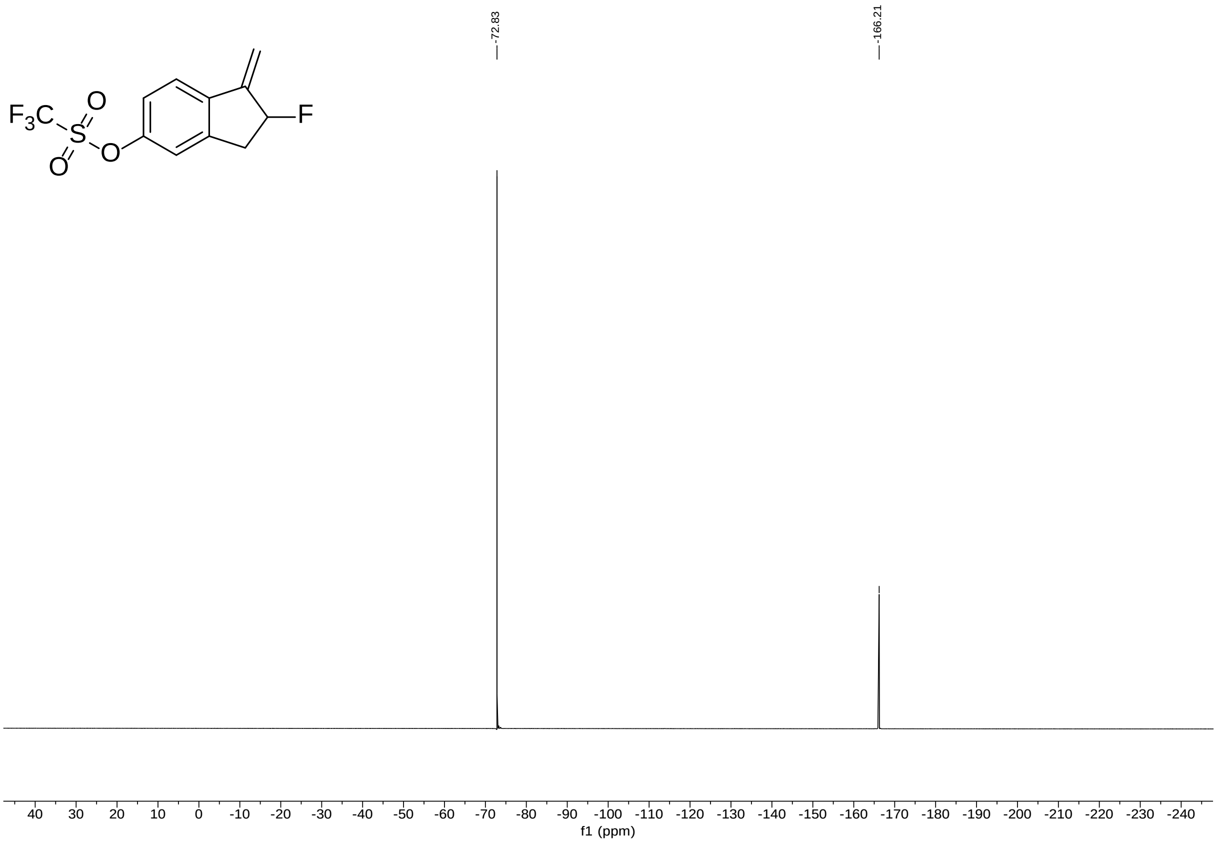

2-Fluoro-1,1-dimethoxy-6-(trifluoromethyl)-2,3-dihydro-1*H*-indene (S18)

^{1}H NMR (599 MHz, CD_2Cl_2, 299 K)

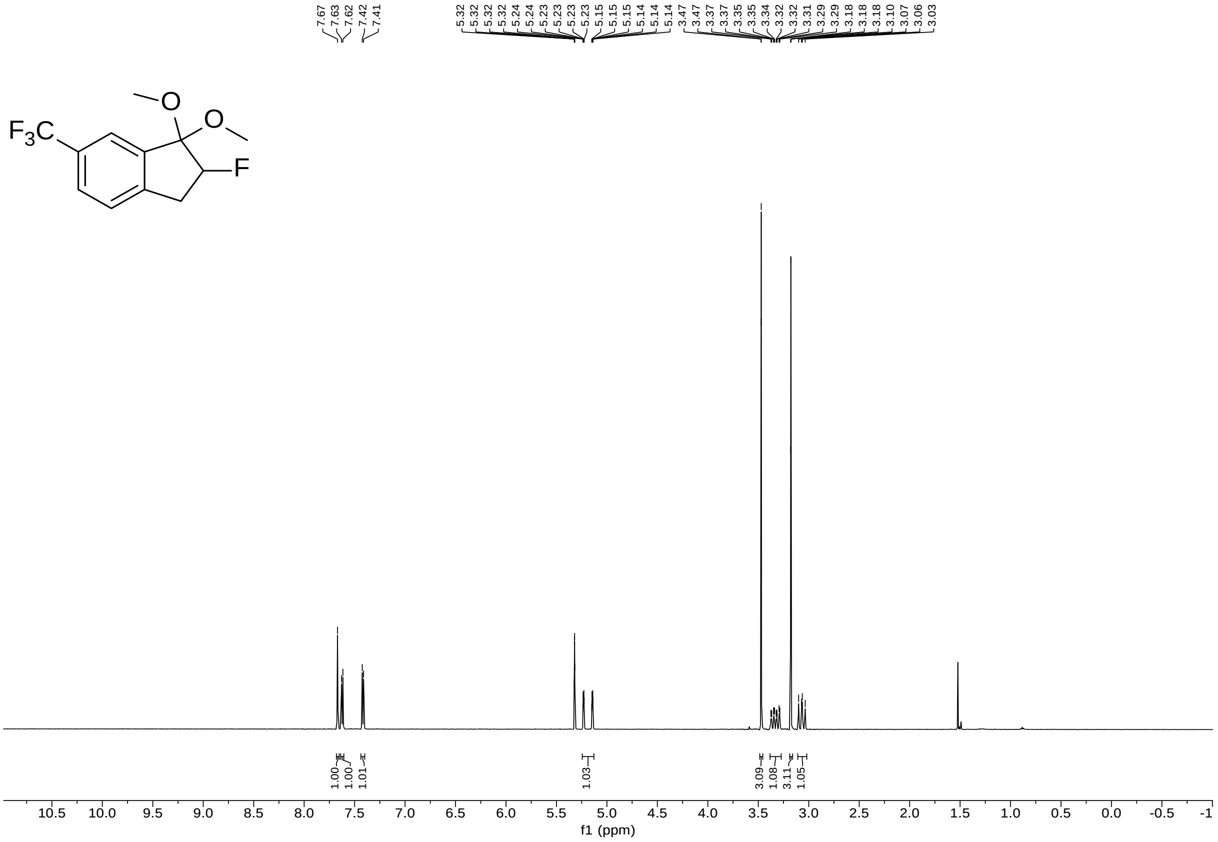

^{13}C NMR (151 MHz, CD_2Cl_2, 299 K)

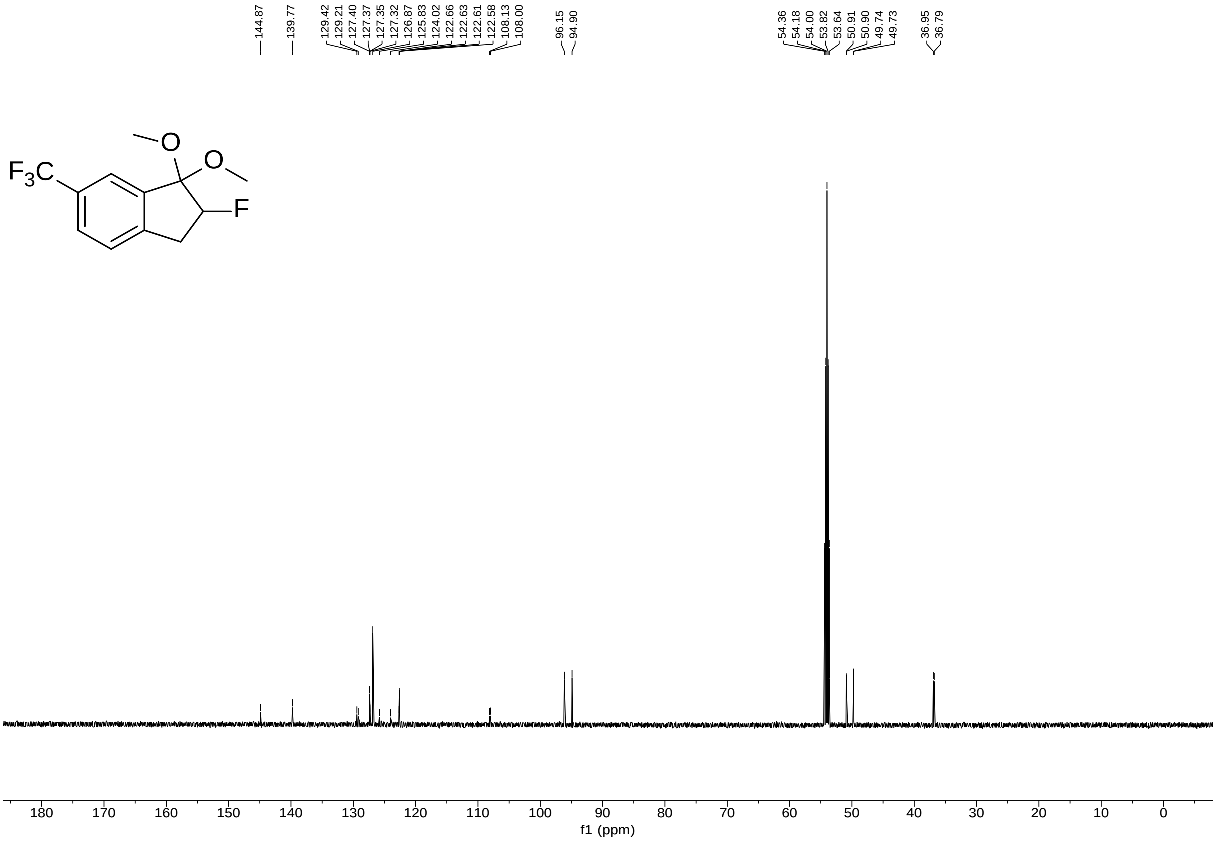

^{19}F NMR (564 MHz, CD_2Cl_2, 299 K)

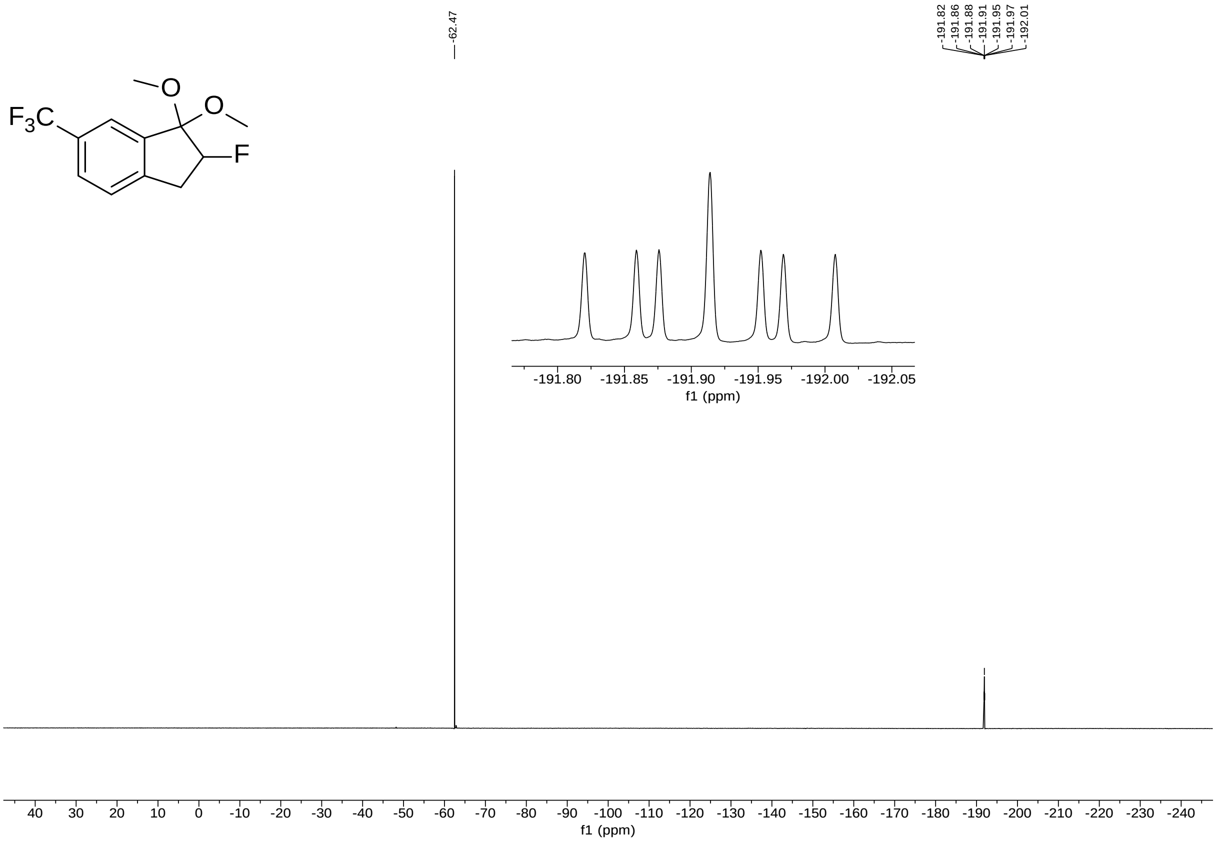

^{19}F{^{1}H} NMR (564 MHz, CD_2Cl_2, 299 K)

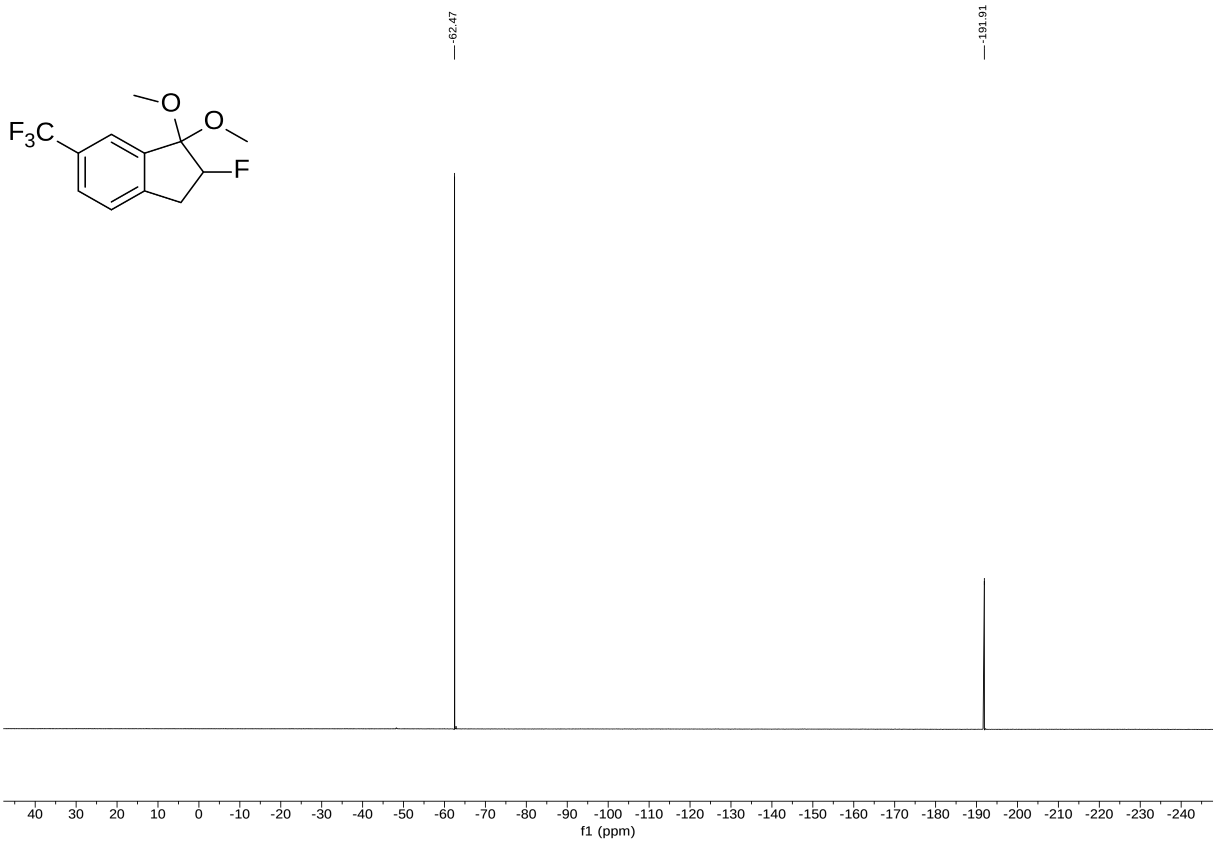

2-Fluoro-6-(trifluoromethyl)-2,3-dihydro-1*H*-inden-1one (S19)

^{1}H NMR (599 MHz, $CDCl_3$, 299 K)

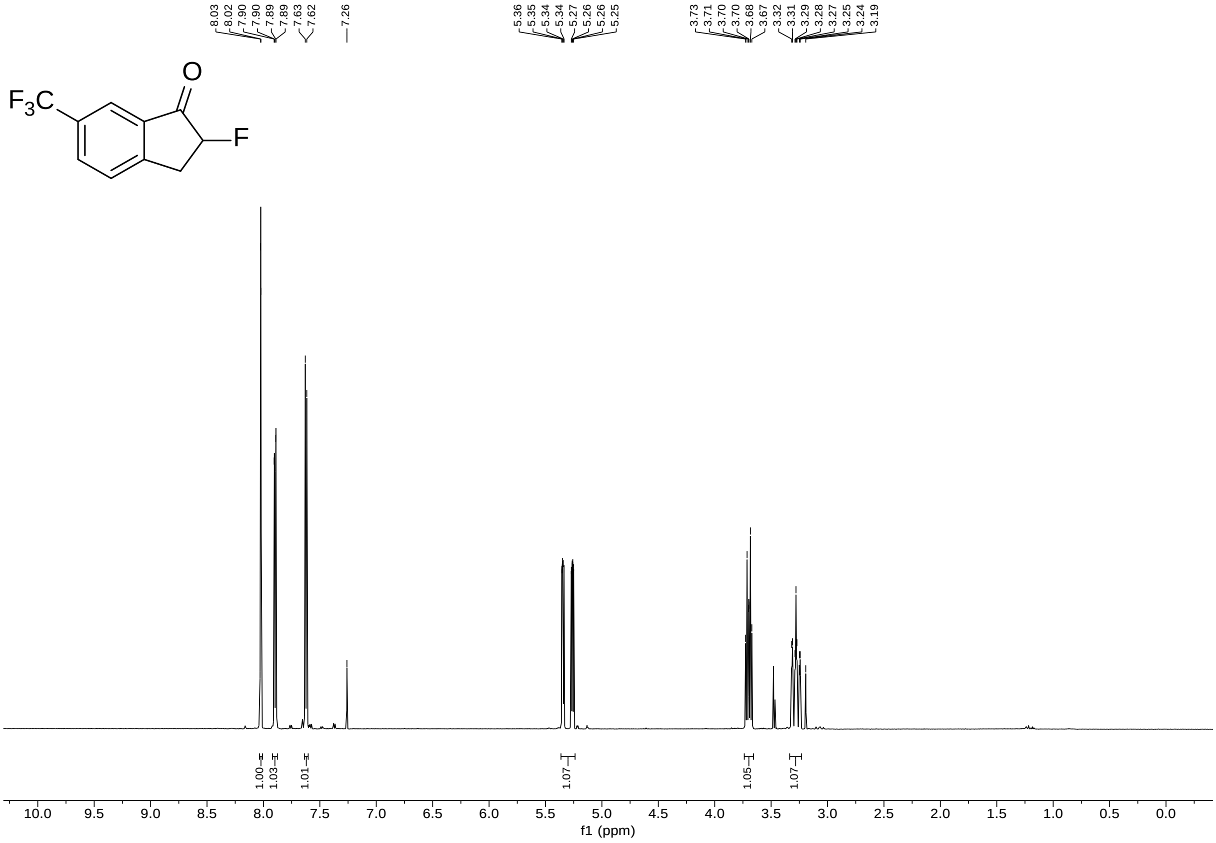

^{13}C NMR (151 MHz, $CDCl_3$, 299 K)

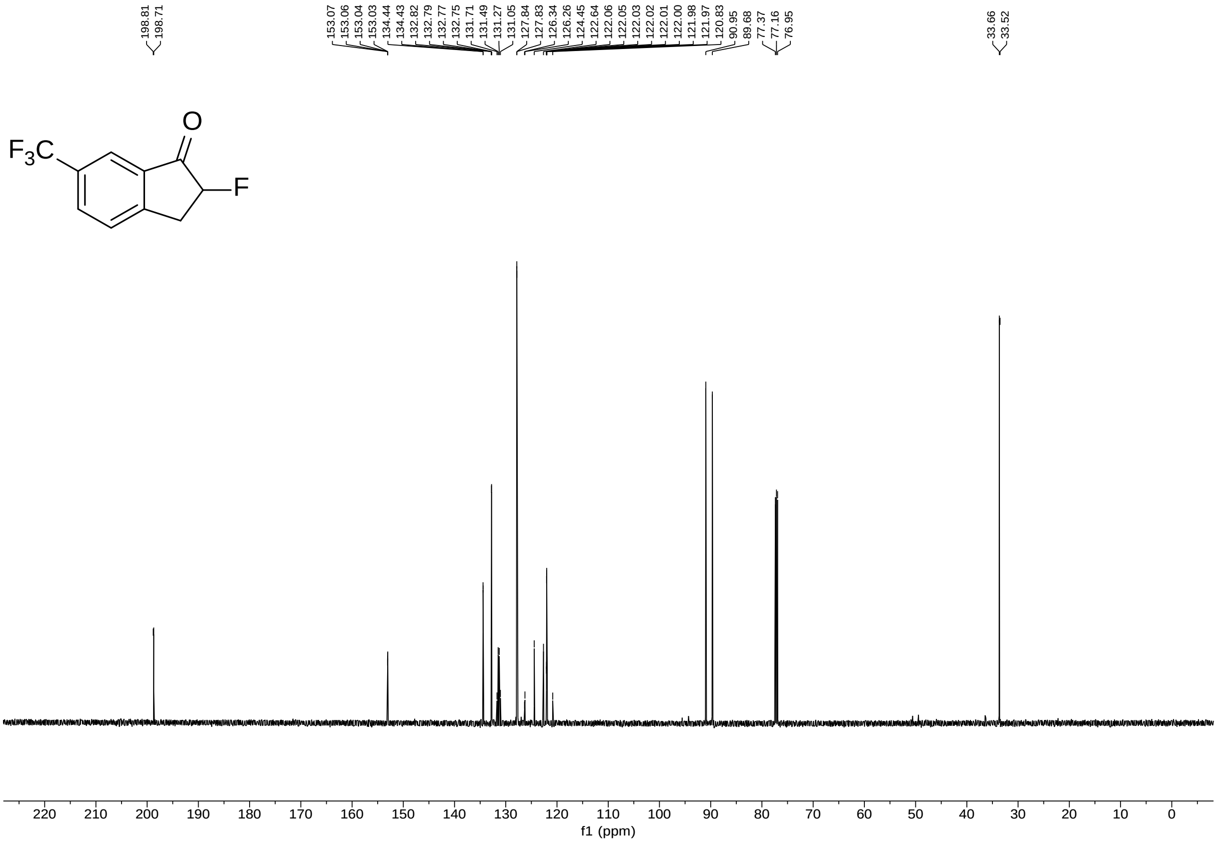

^{19}F NMR (564 MHz, $CDCl_3$, 299 K)

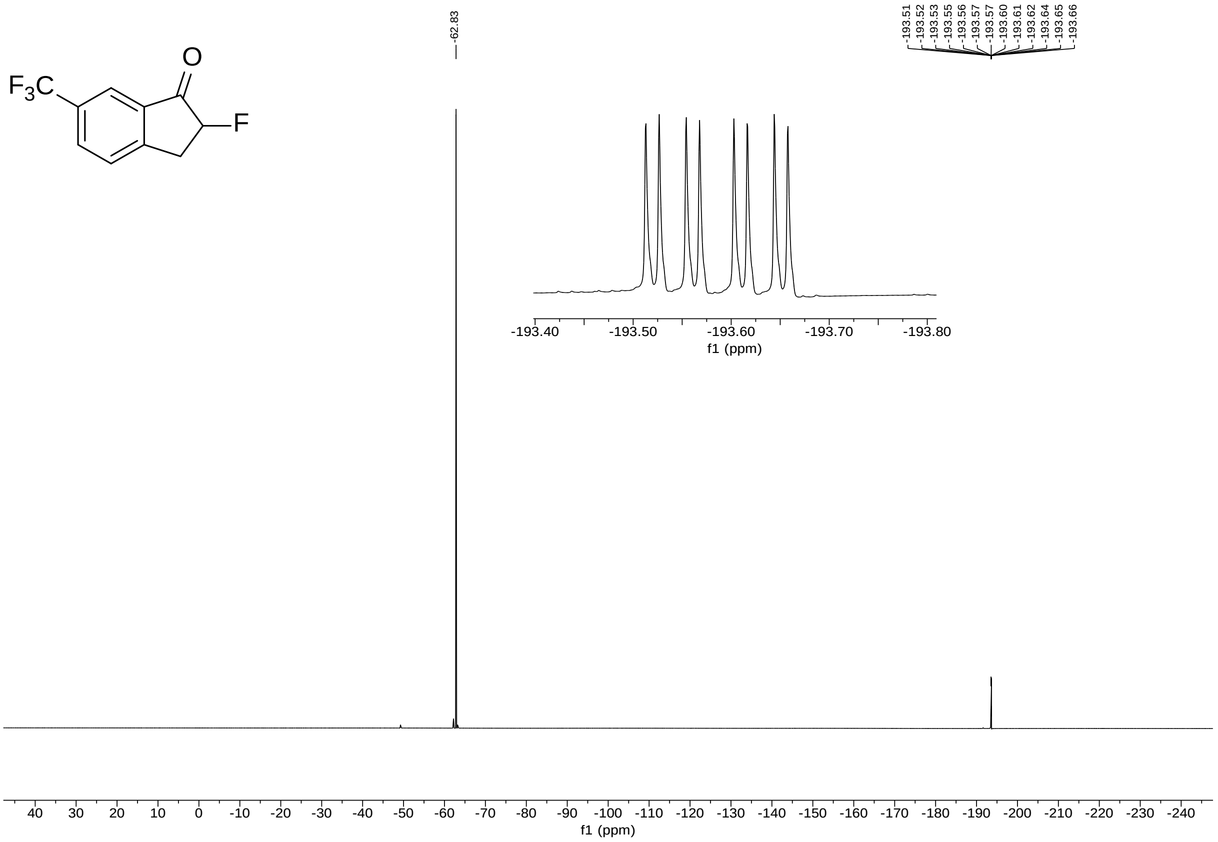

^{19}F{^{1}H} NMR (564 MHz, $CDCl_3$, 299 K)

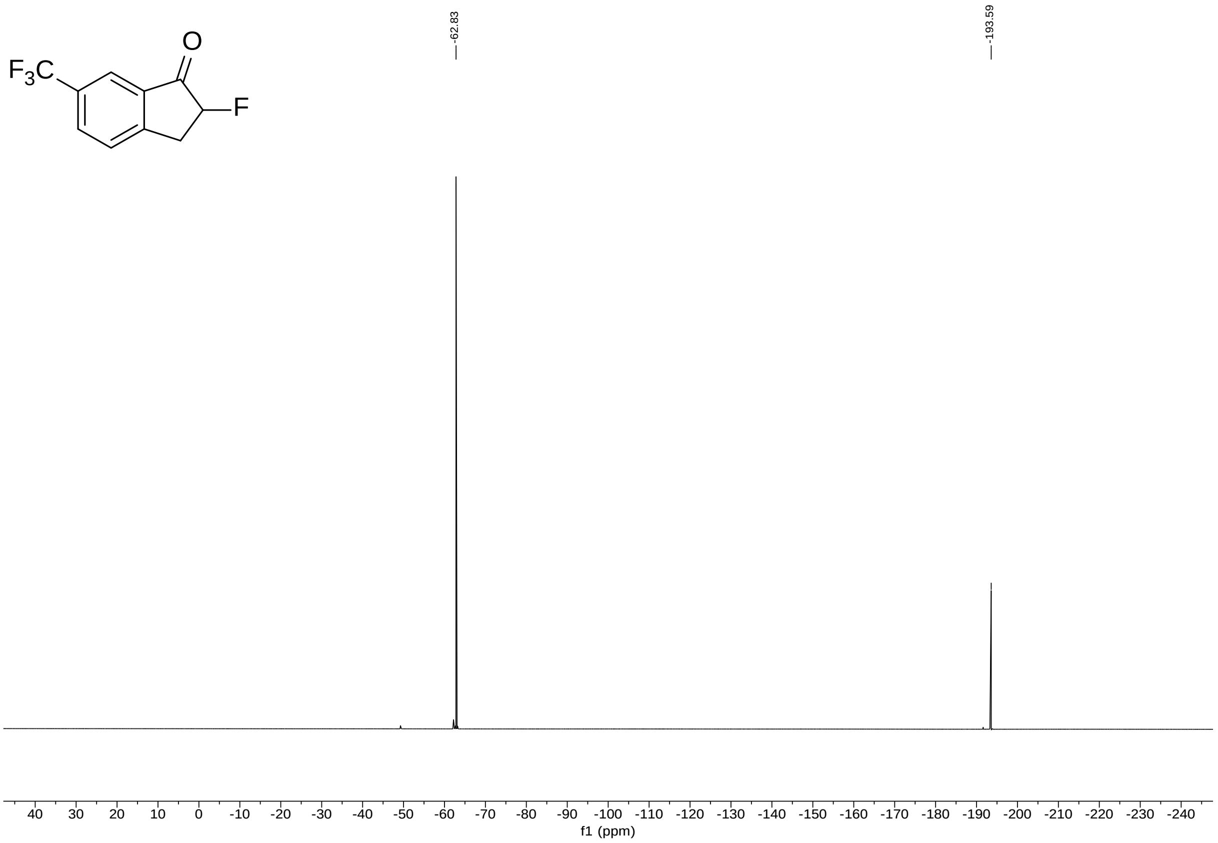

2-Fluoro-1-methylene-6-(trifluoromethyl)-2,3-dihydro-1*H*-indene (S20)

^{1}H NMR (599 MHz, $CDCl_3$, 299 K)

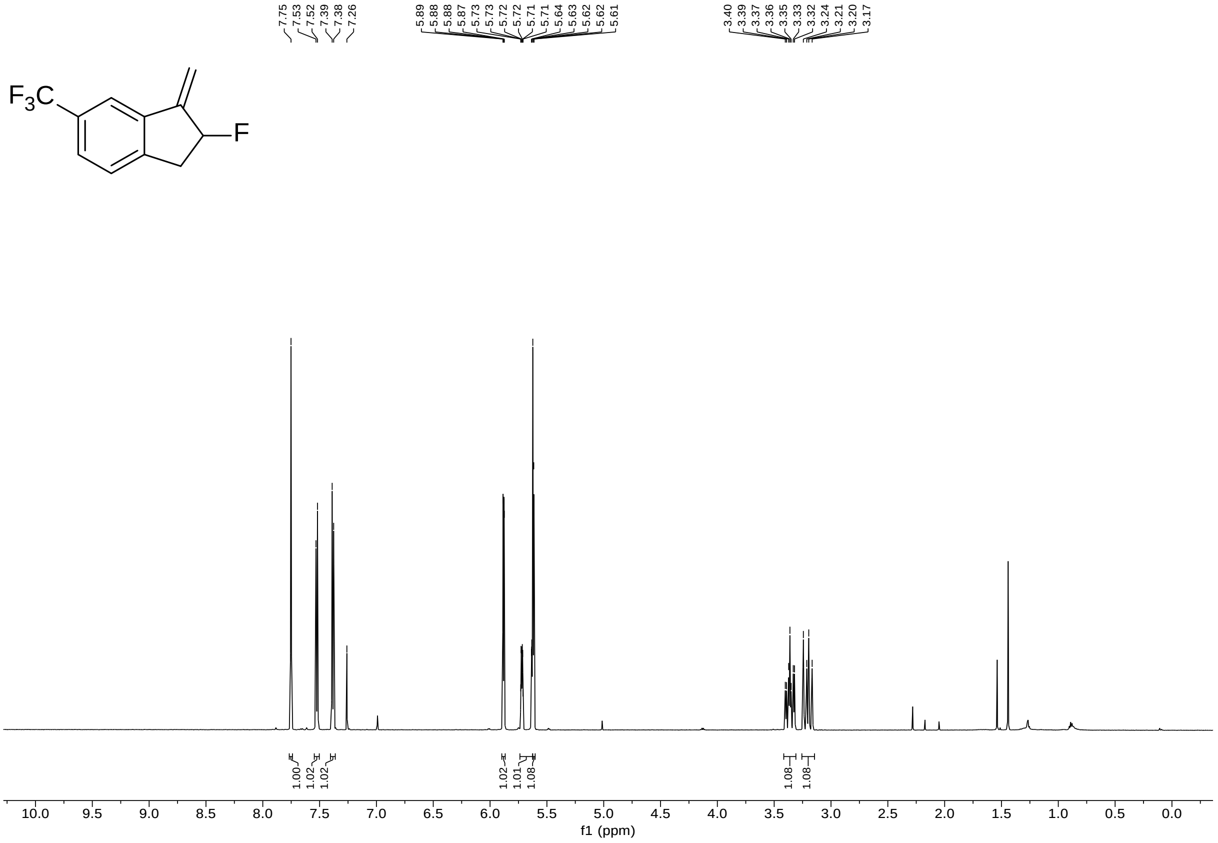

^{13}C NMR (151 MHz, $CDCl_3$, 299 K)

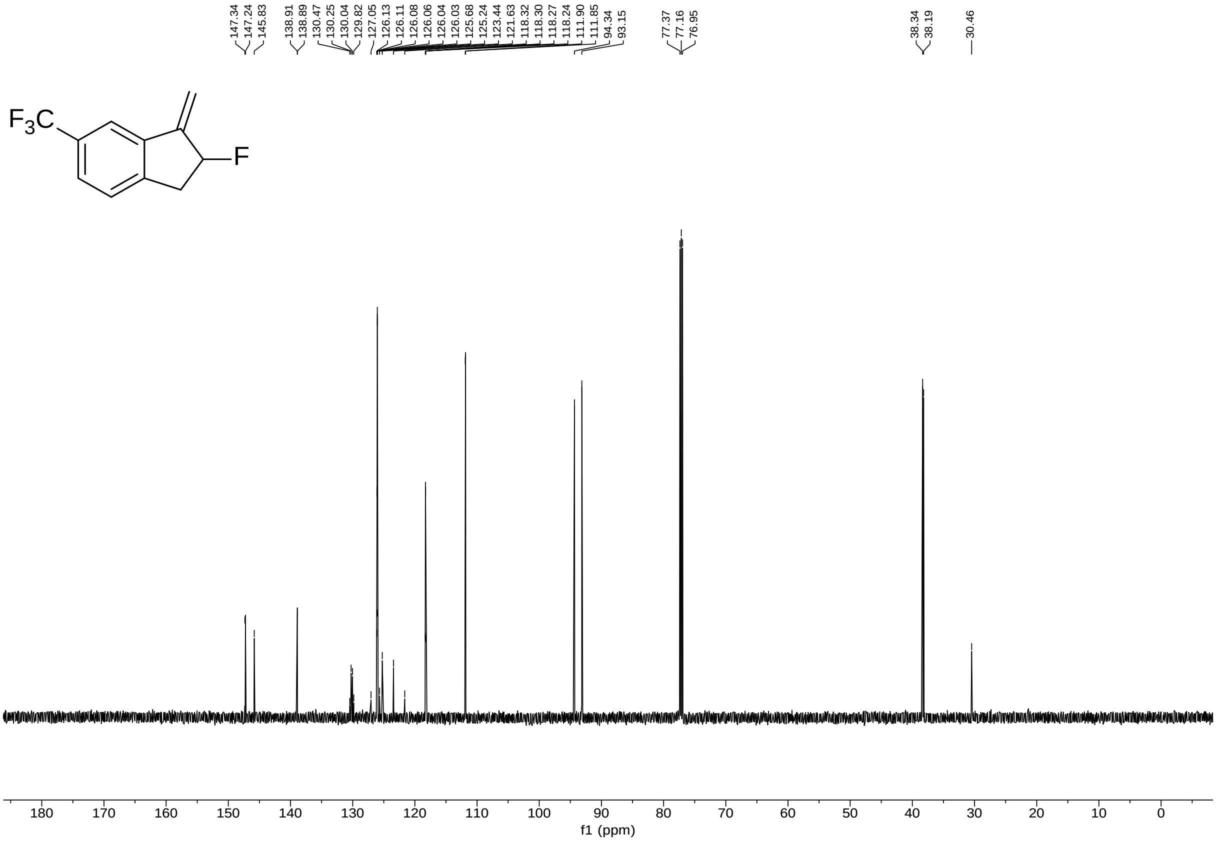

^{19}F NMR (564 MHz, $CDCl_3$, 299 K)

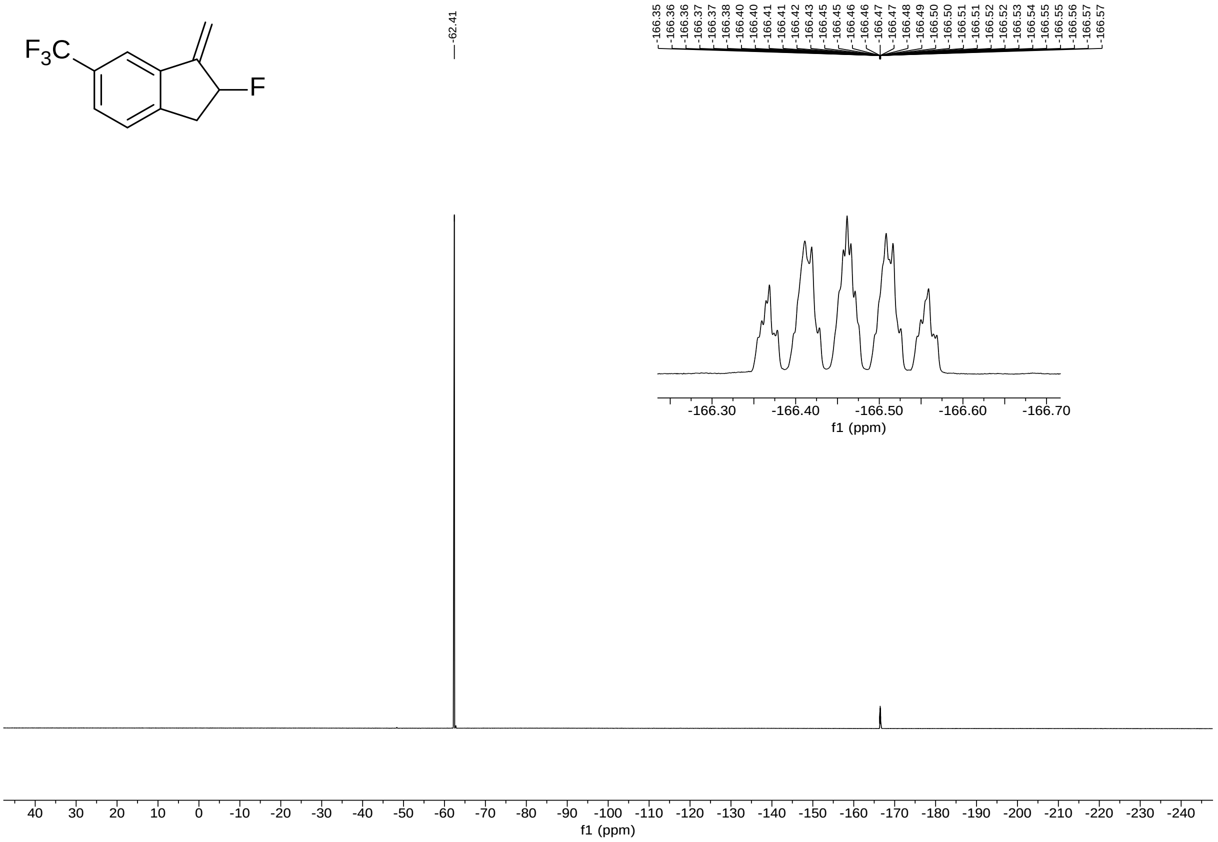

^{19}F{^{1}H} NMR (564 MHz, $CDCl_3$, 299 K)

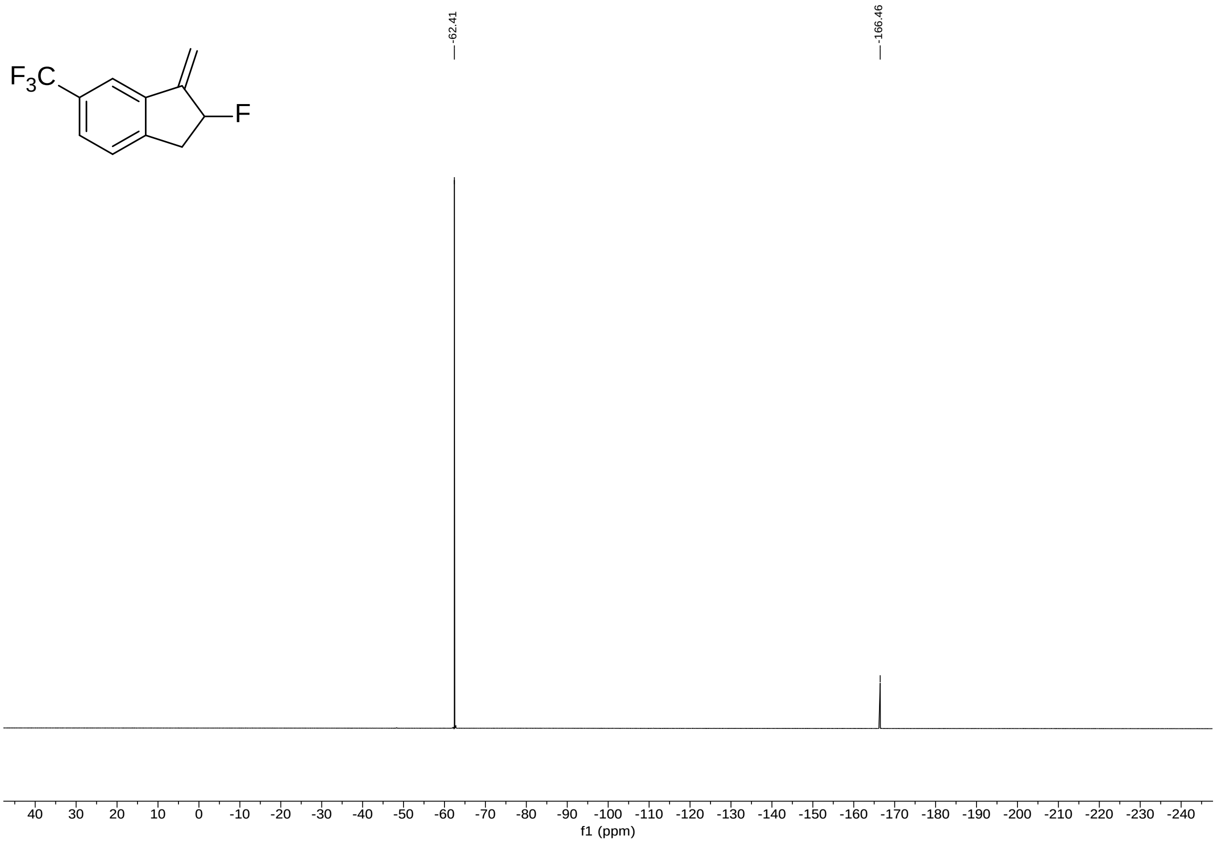

2-Fluoro-1,1-dimethoxy-2,3-dihydro-1H-indene-4-carbonitrile (S21)

^{1}H NMR (599 MHz, CD_2Cl_2, 299 K)

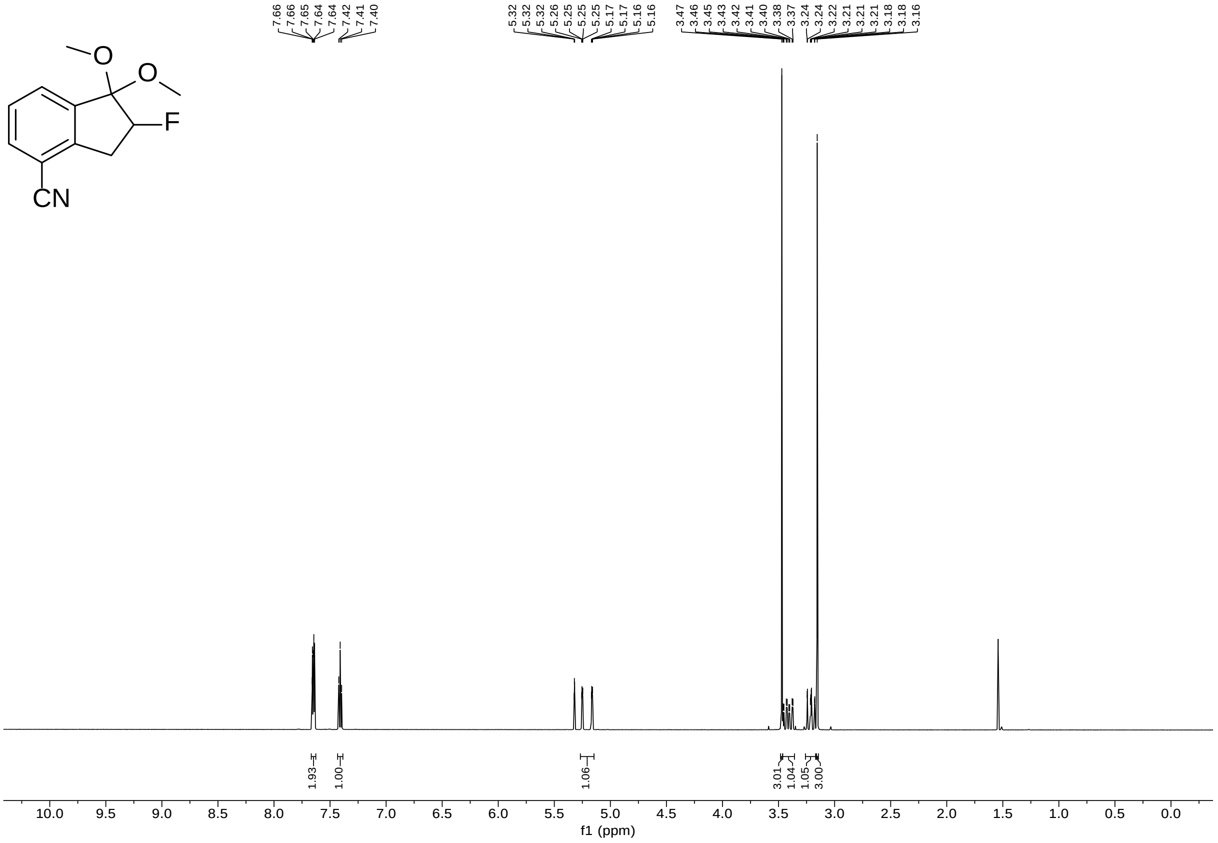

^{13}C NMR (151 MHz, CD_2Cl_2, 299 K)

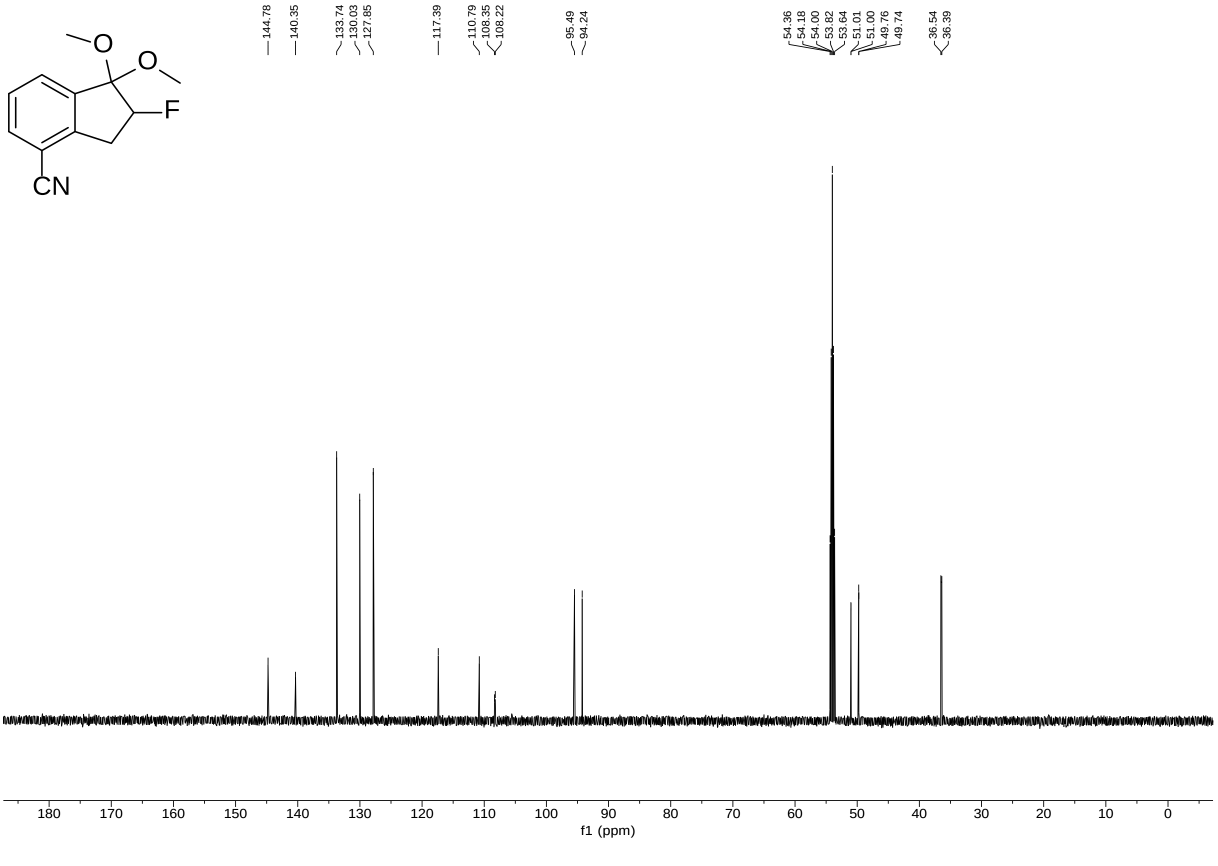

^{19}F NMR (564MHz, CD_2Cl_2, 299 K)

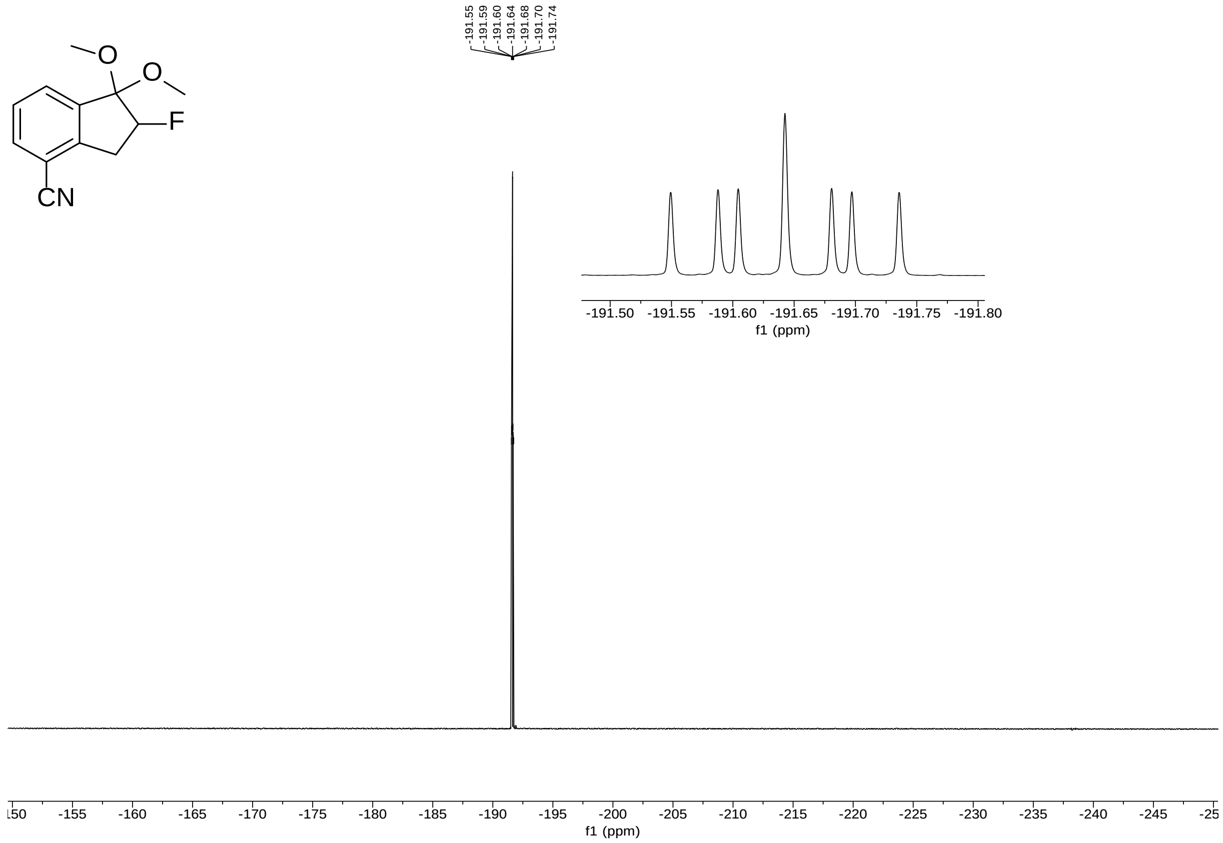

$^{19}F\{^1H\}$ NMR (564MHz, CD_2Cl_2, 299 K)

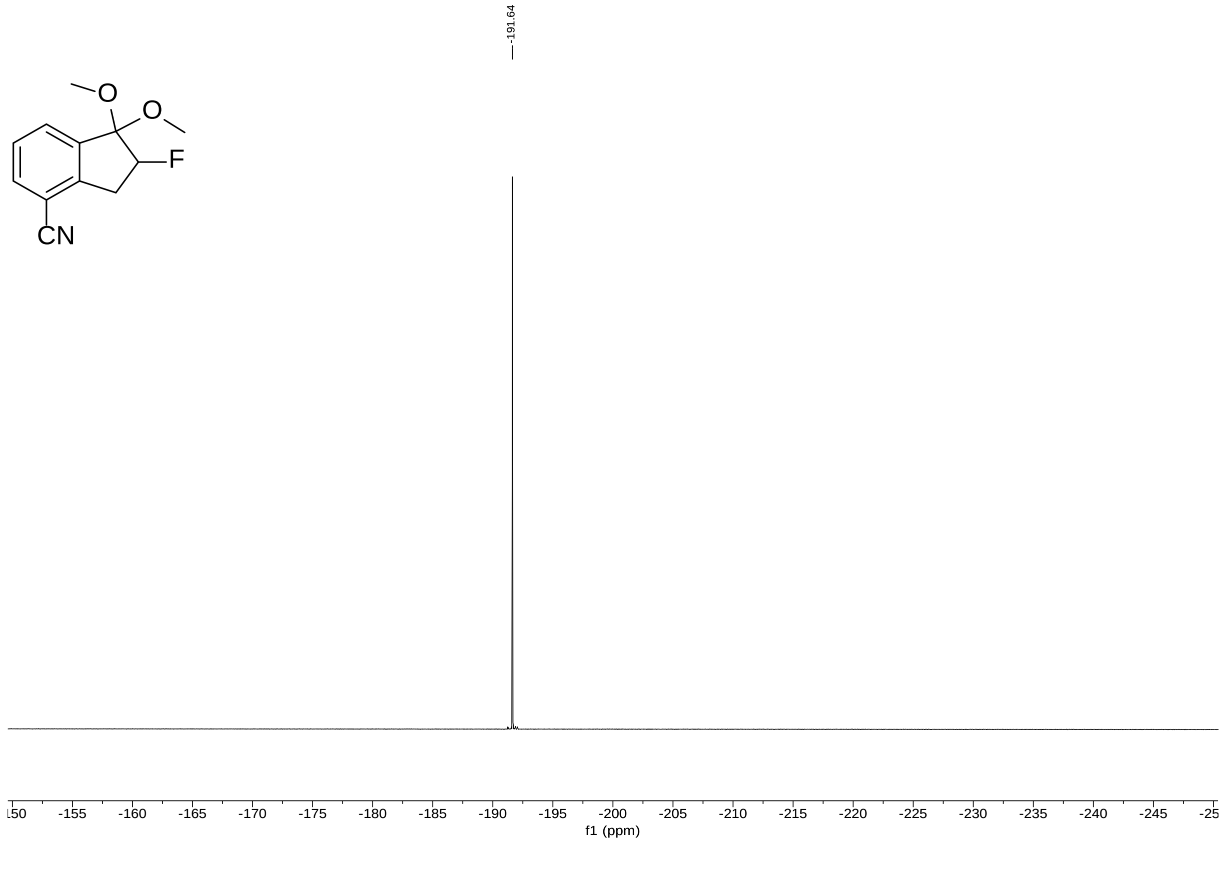

2-Fluoro-1-methylene-2,3-dihydro-1*H*-indene-5-carbonitrile (S22)

^{1}H NMR (500 MHz, $CDCl_3$, 299 K)

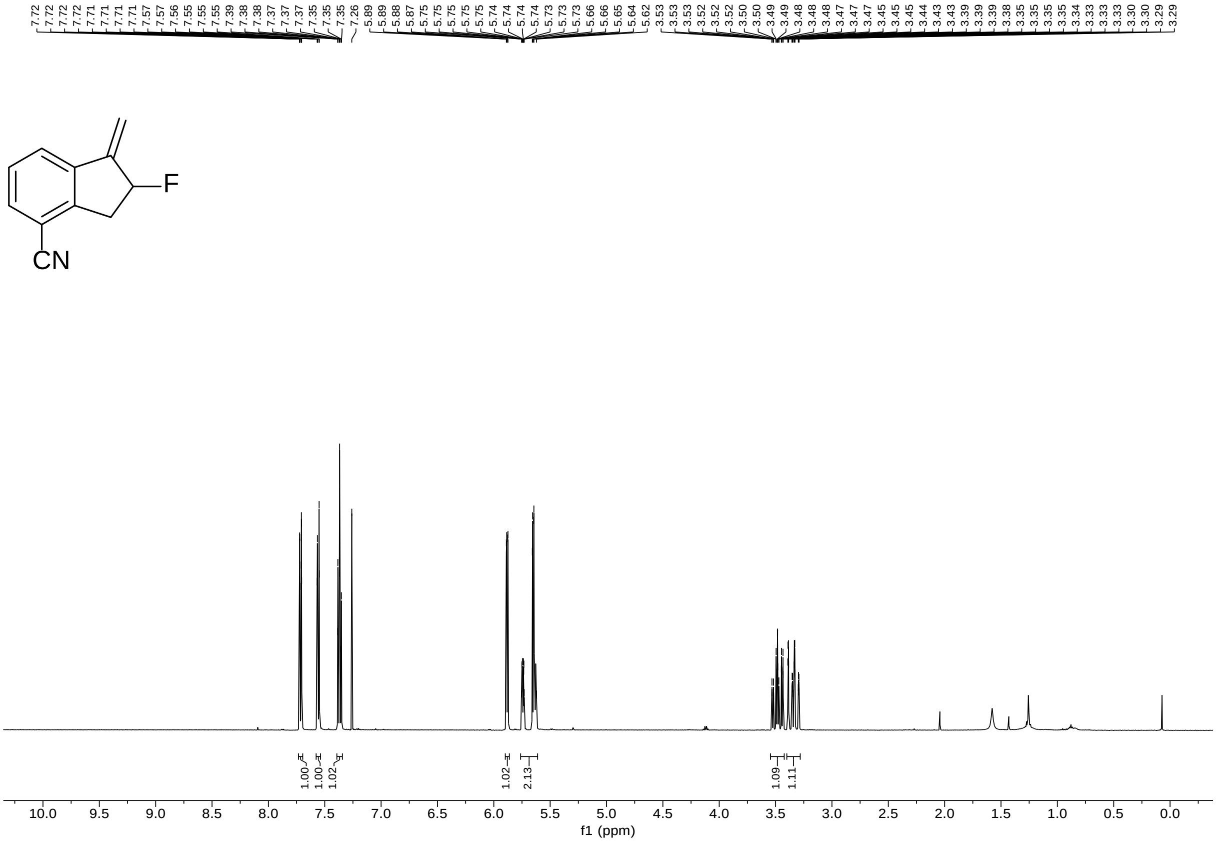

^{13}C NMR (126 MHz, $CDCl_3$, 299 K)

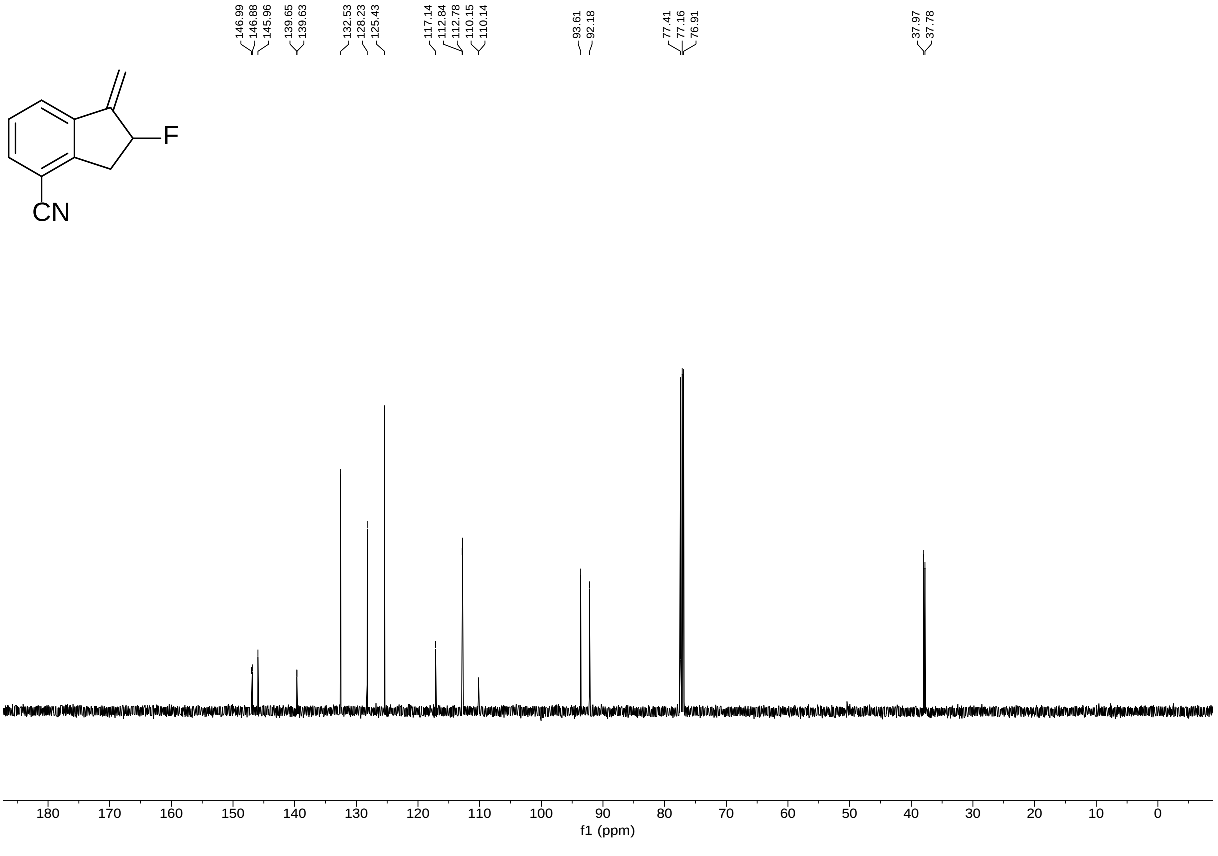

^{19}FNMR (470 MHz, $CDCl_3$, 299 K)

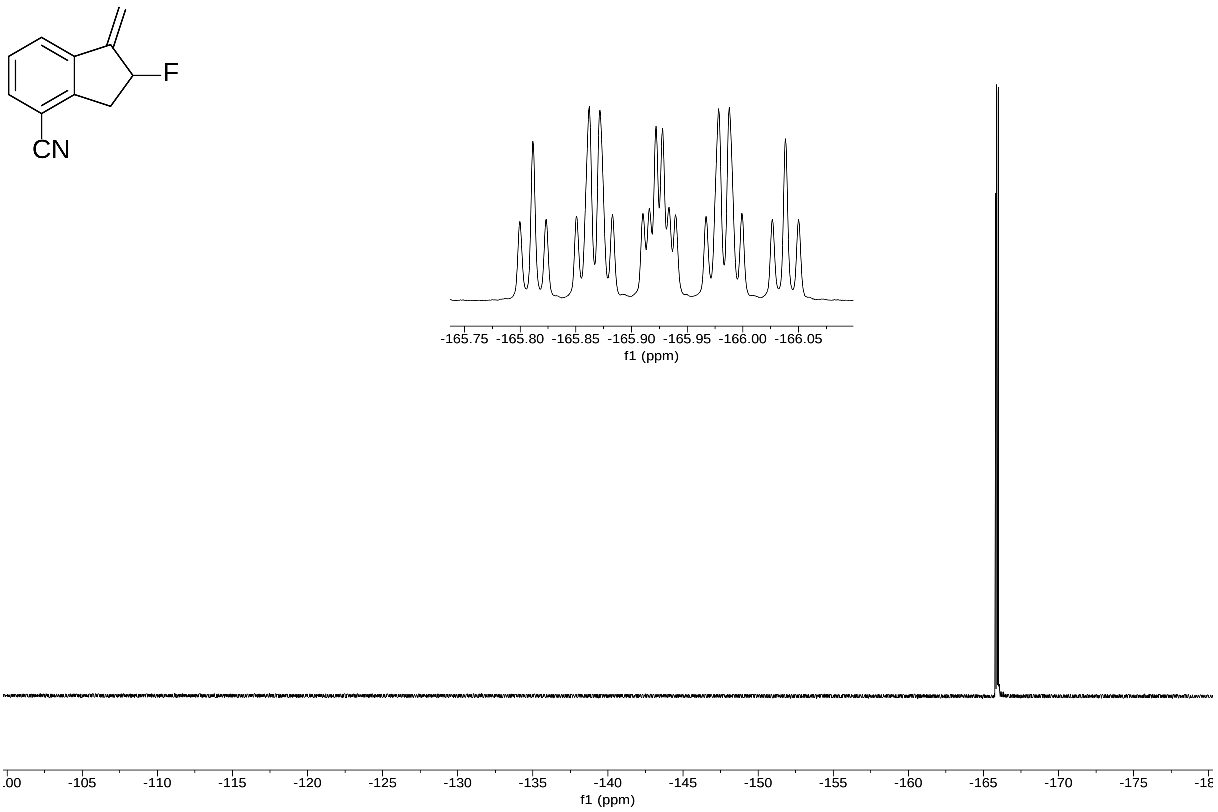

^{19}F NMR{^{1}H} (470 MHz, $CDCl_3$, 299 K)

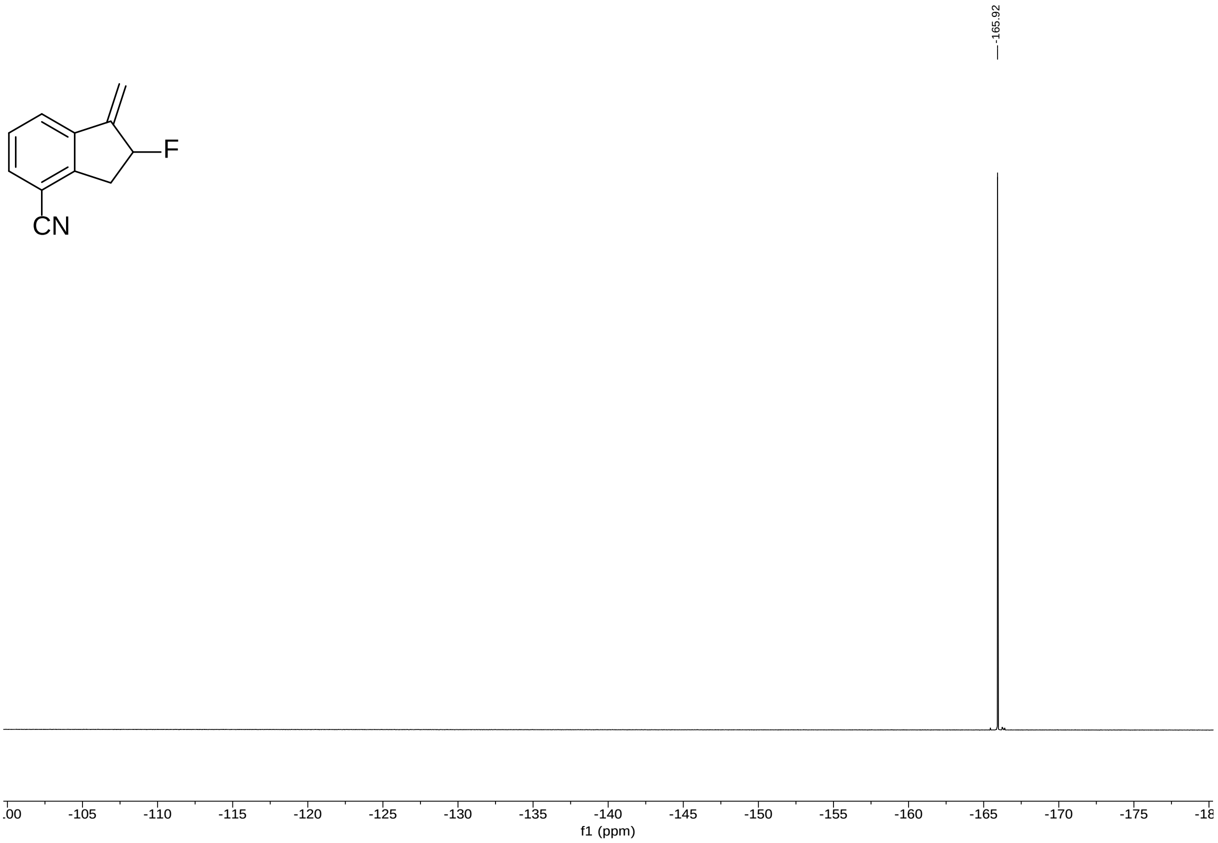

2-(3-Oxo-2,3-dihydro-1*H*-inden-5-yl)isoindoline-1,3-dione (S23)

^{1}H NMR (500 MHz, $CDCl_3$, 299 K)

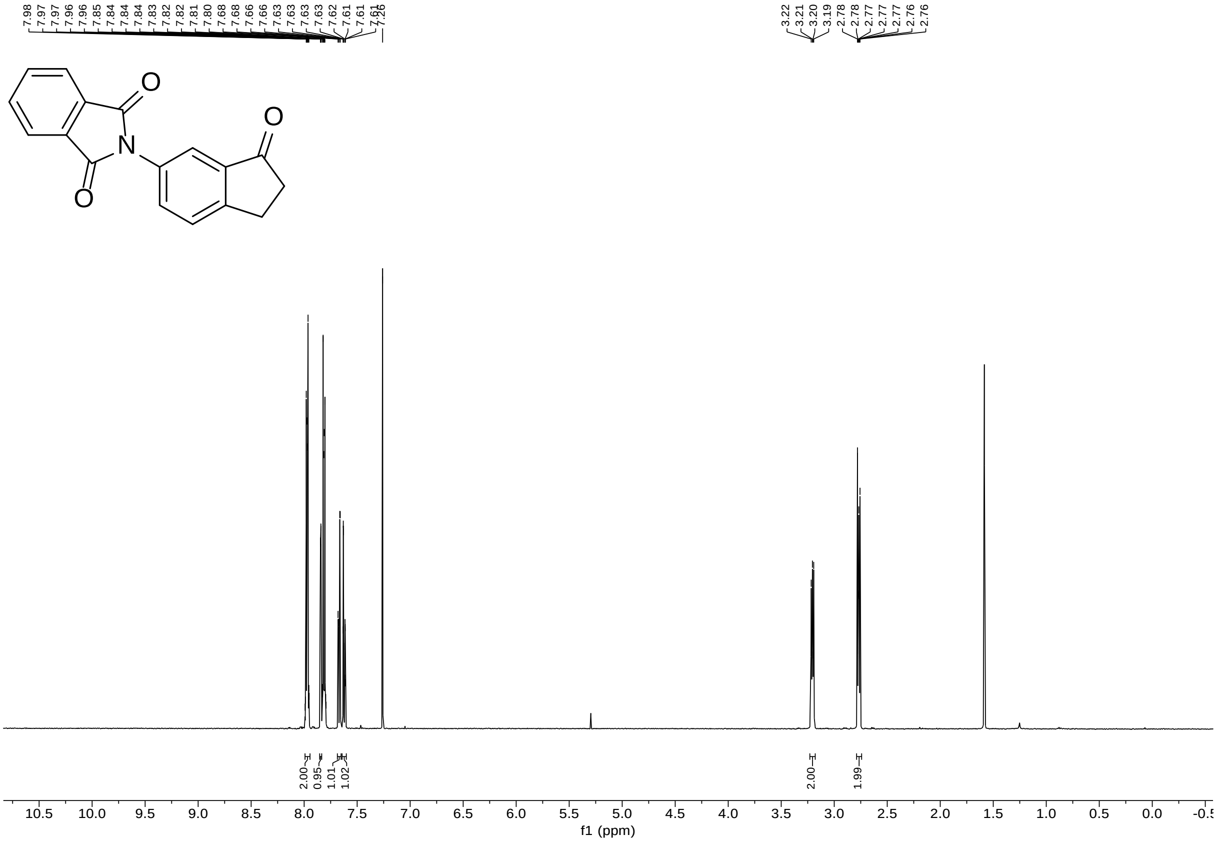

^{13}C NMR (126 MHz, $CDCl_3$, 299 K)

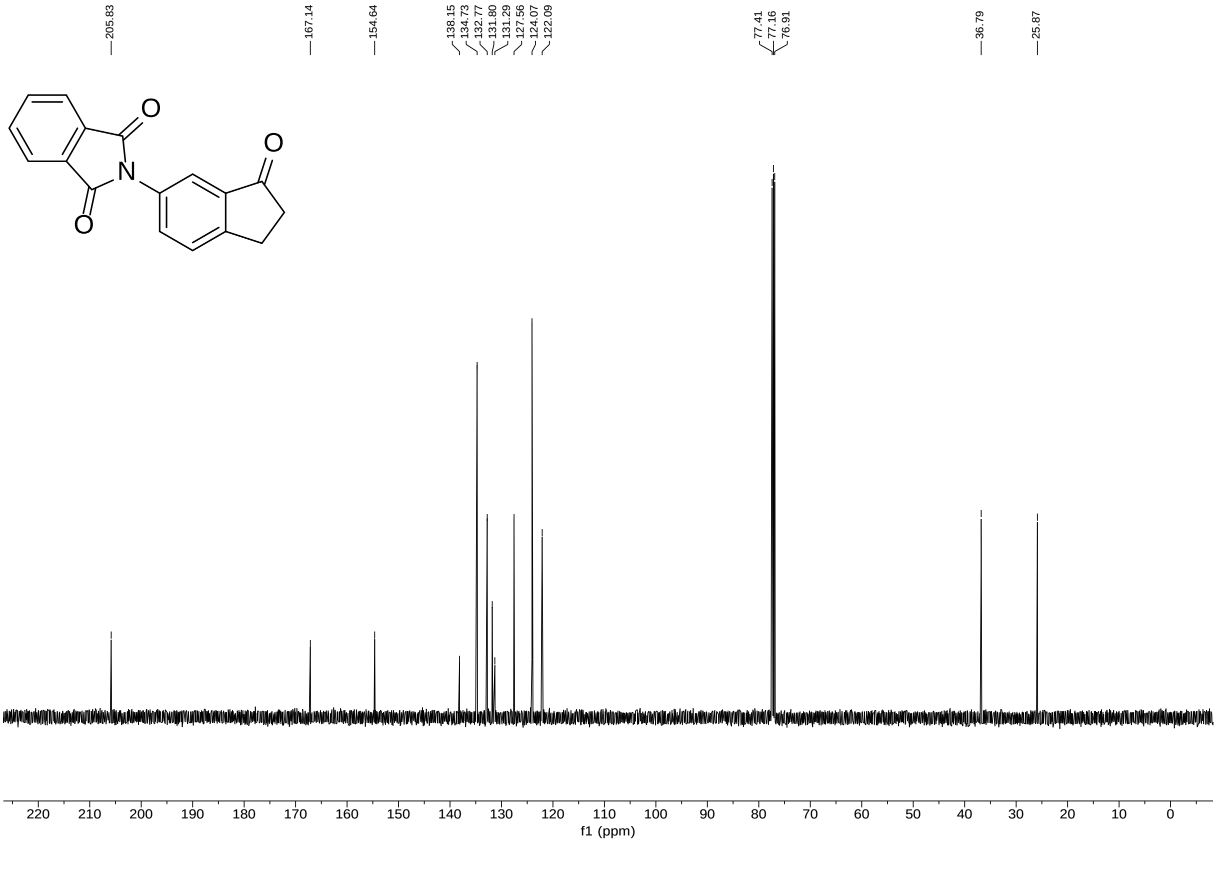

2-(2-Fluoro-3-oxo-2,3-dihydro-1*H*-inden-5-yl)isoindoline-1,3-dione (S24)

^{1}H NMR (500 MHz, $CDCl_3$, 299 K)

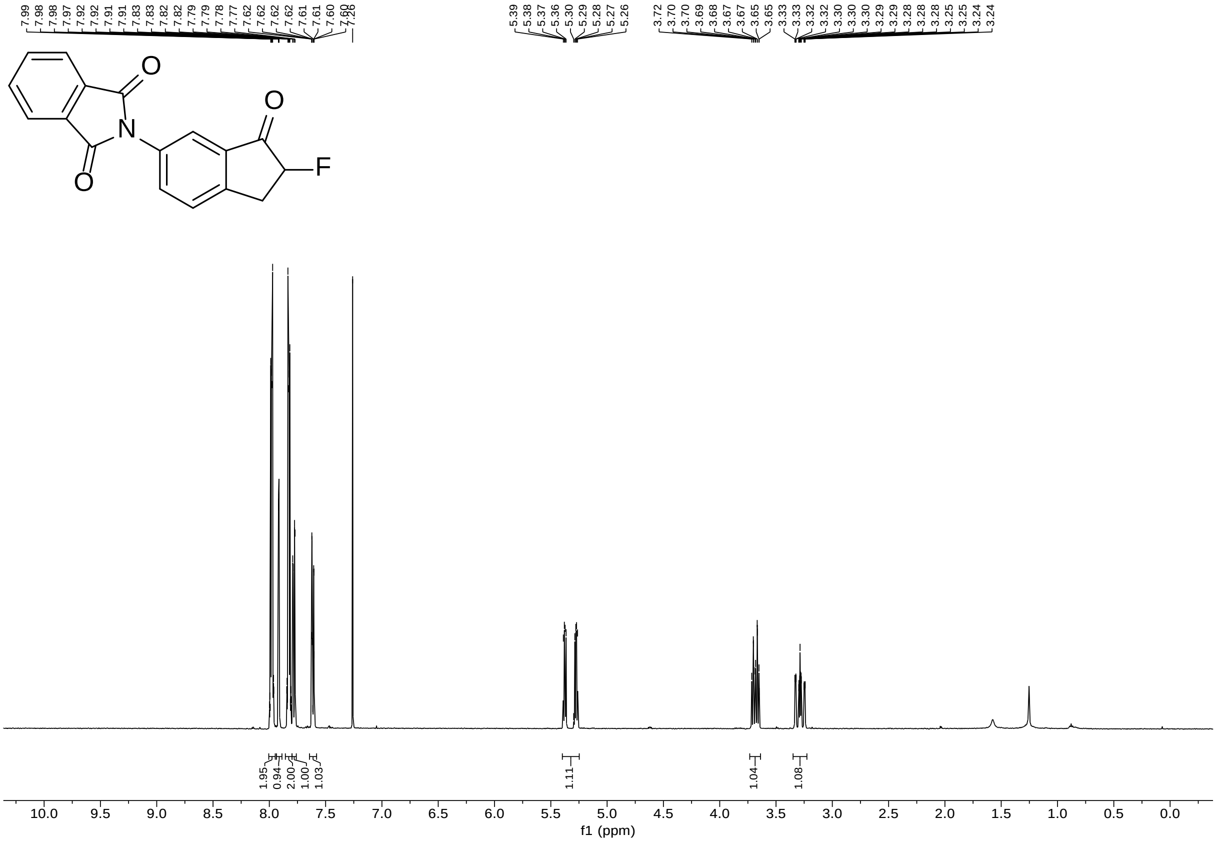

^{13}C NMR (126 MHz, $CDCl_3$, 299 K)

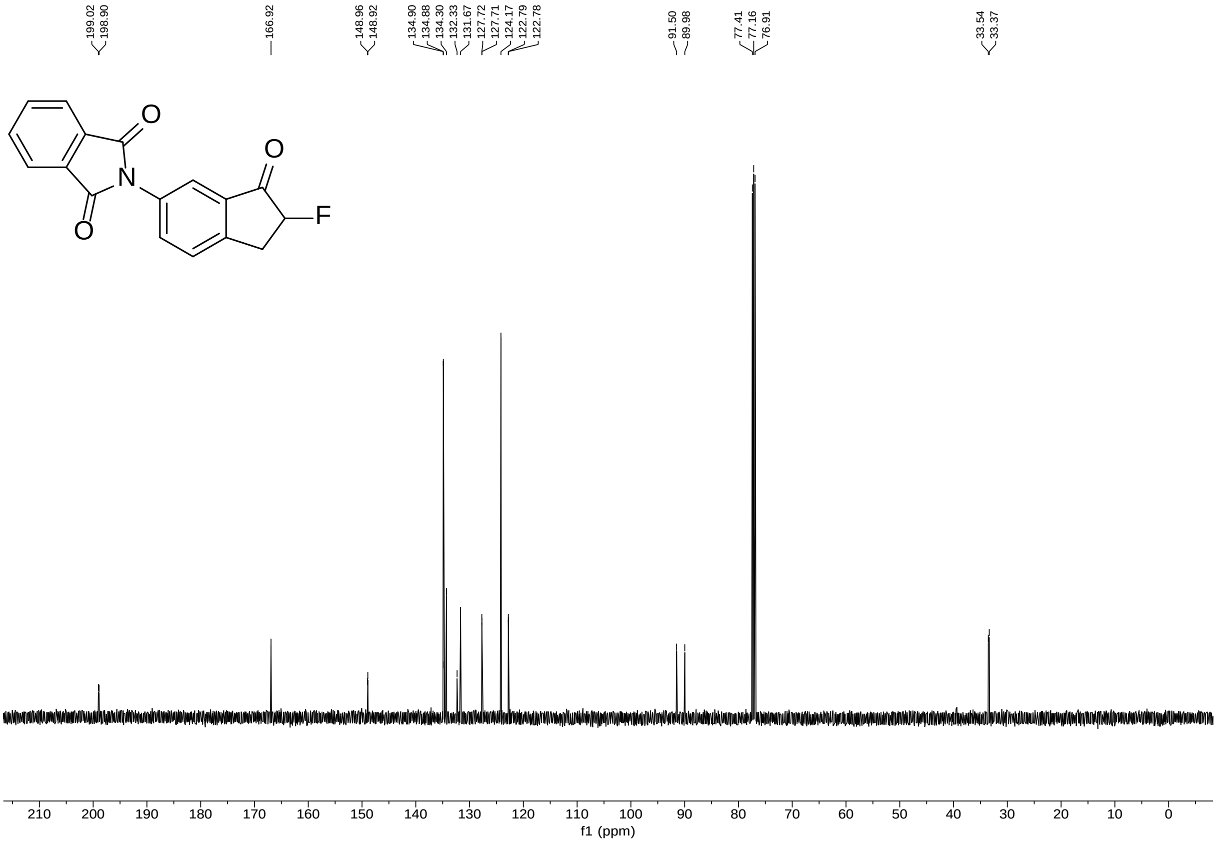

^{19}F NMR (470 MHz, $CDCl_3$, 299 K)

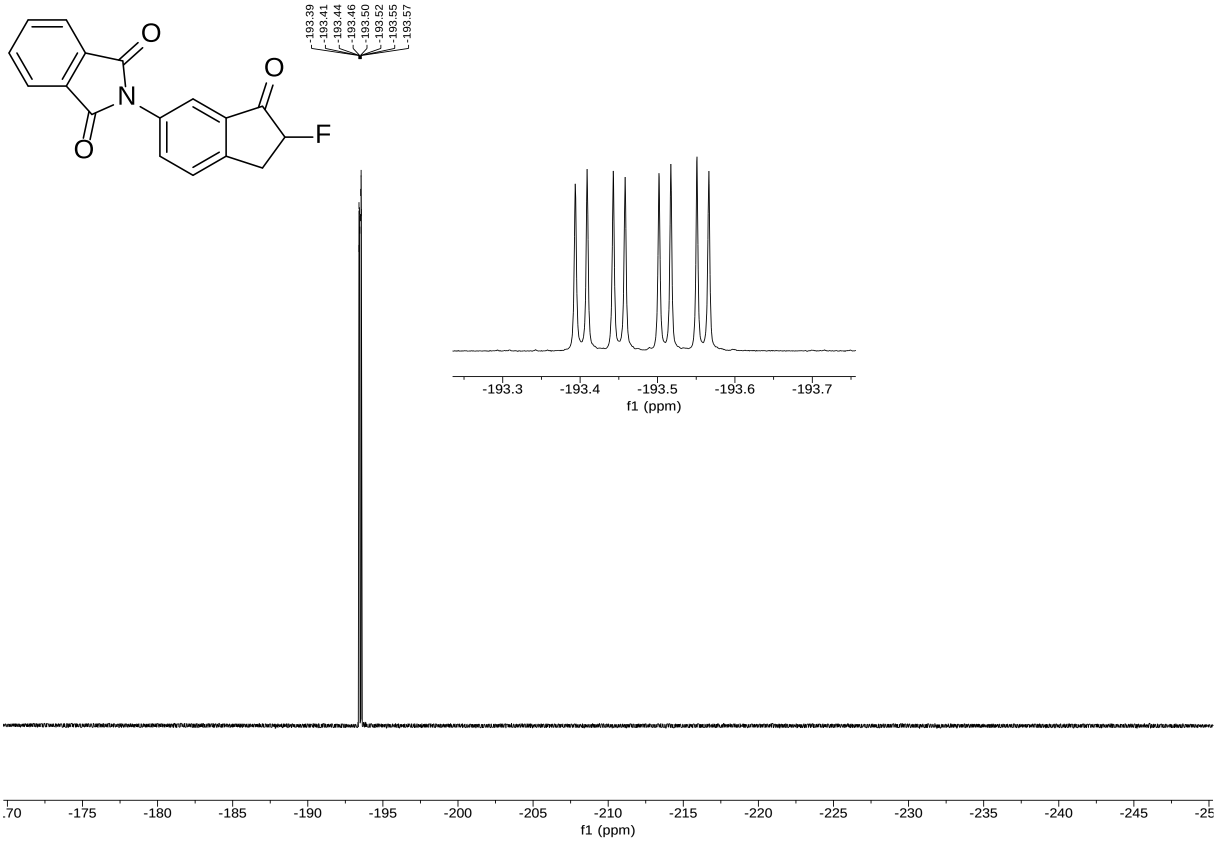

^{19}F NMR{^{1}H} (470 MHz, $CDCl_3$, 299 K)

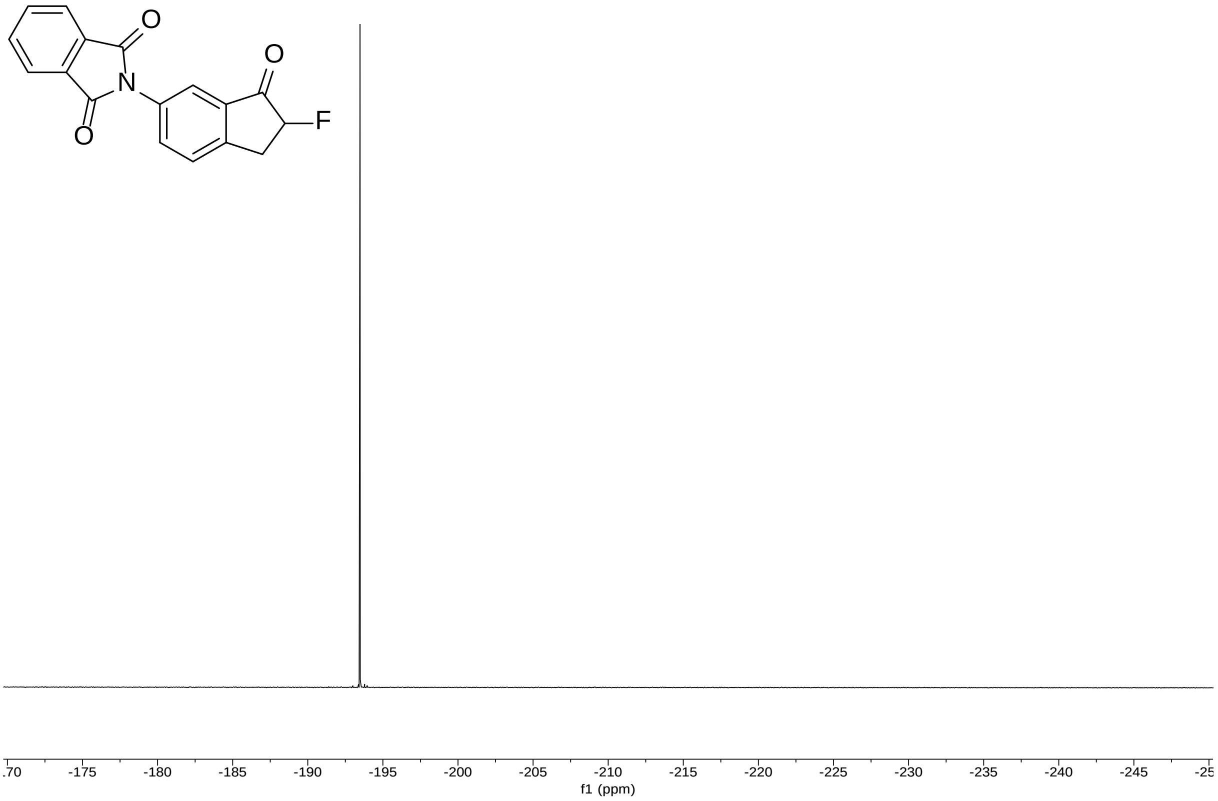

2-(2-Fluoro-3-methylene-2,3-dihydro-1*H*-inden-5-yl)isoindoline-1,3-dione (S25)

^{1}H NMR (500 MHz, $CDCl_3$, 299 K)

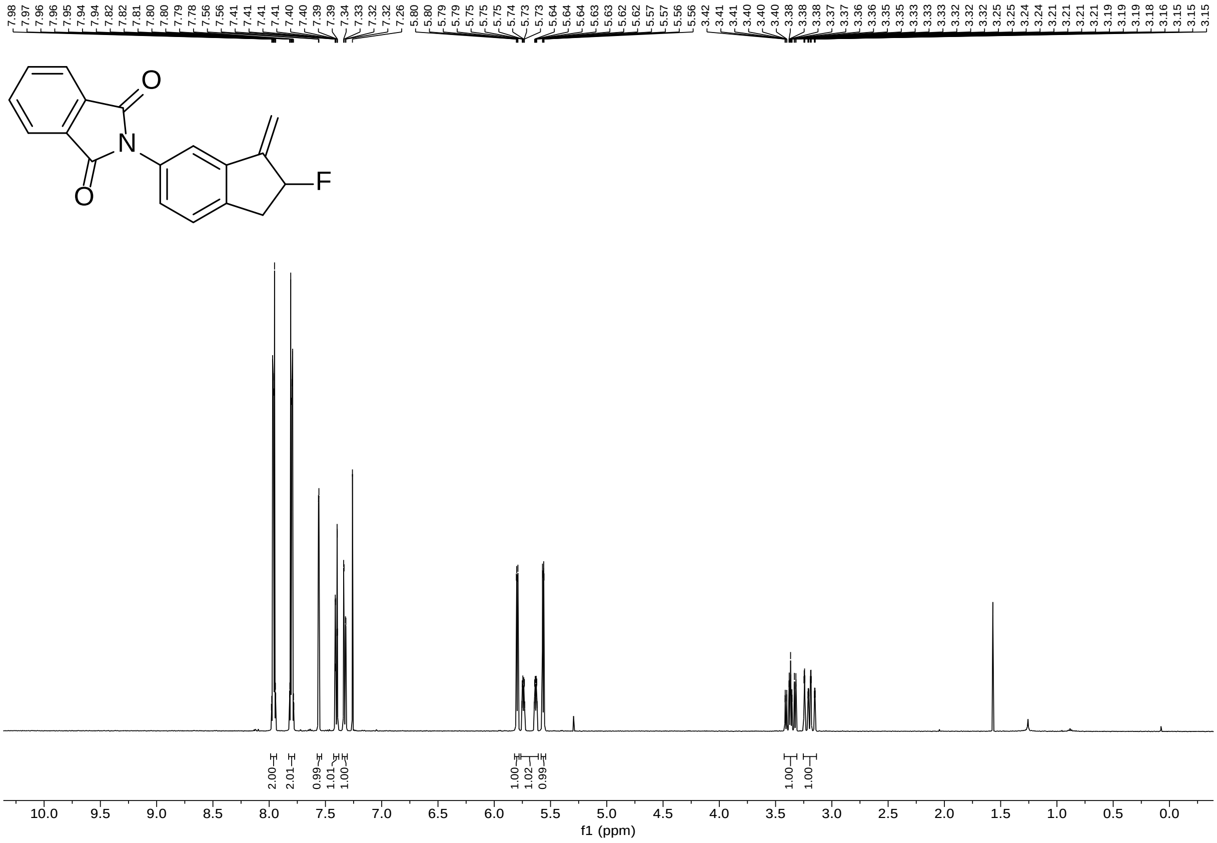

^{13}C NMR (126 MHz, $CDCl_3$, 299 K)

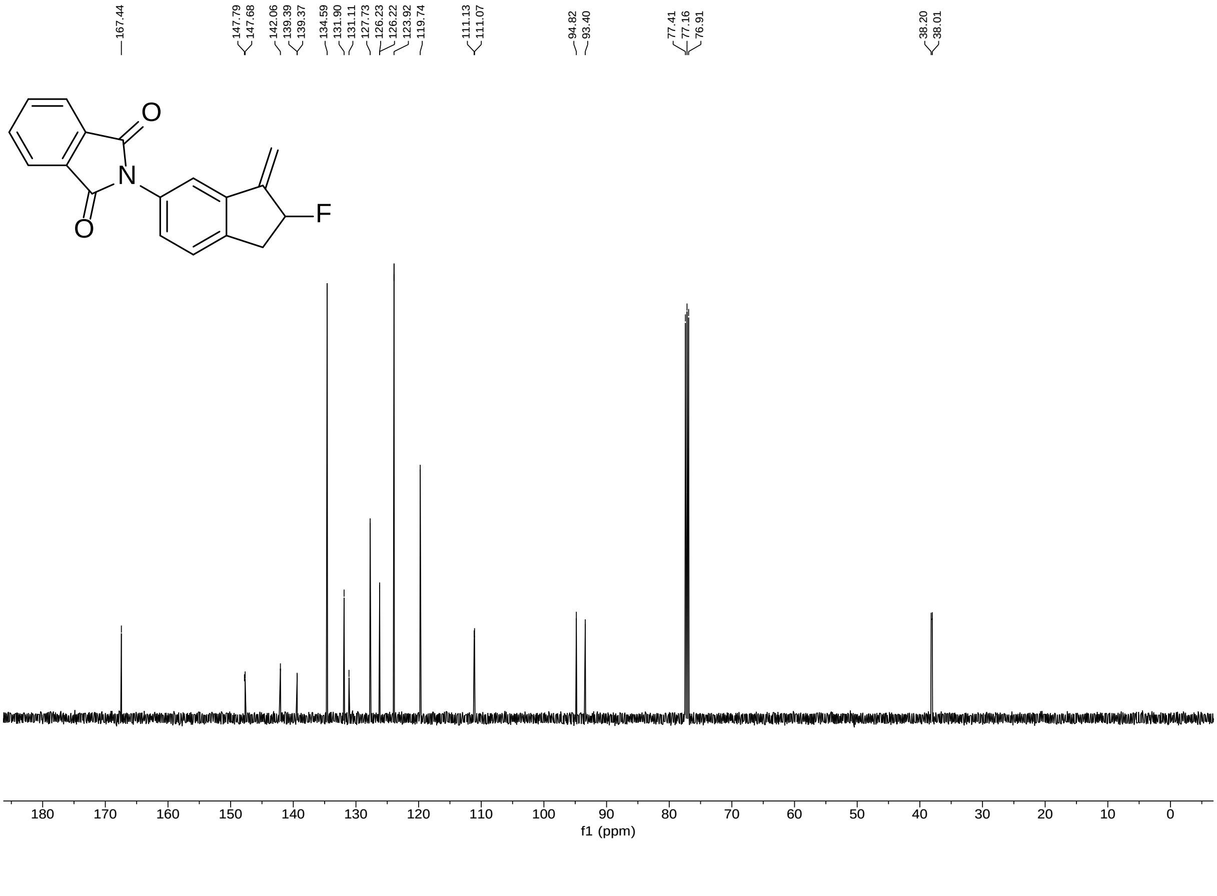

^{19}F NMR (470 MHz, $CDCl_3$, 299 K)

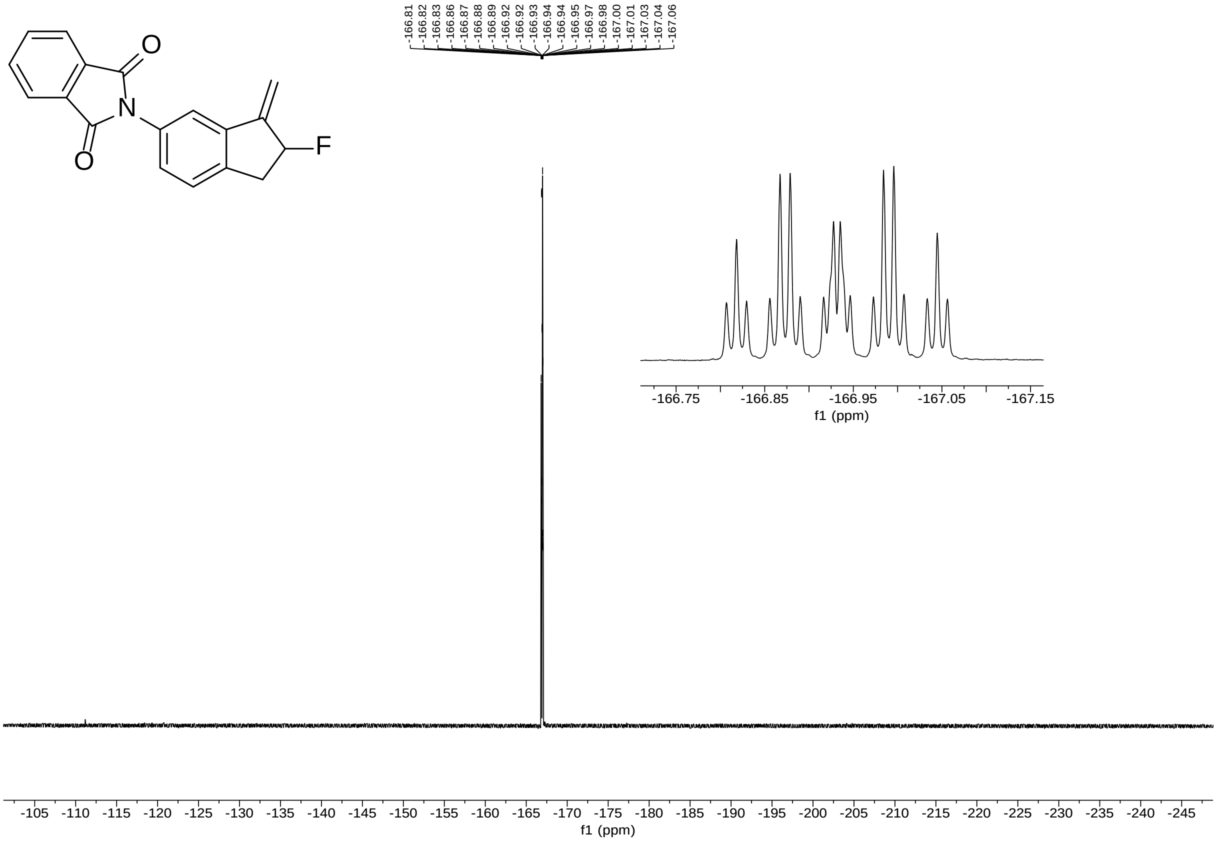

^{19}F NMR{^{1}H} (470 MHz, $CDCl_3$, 299 K)

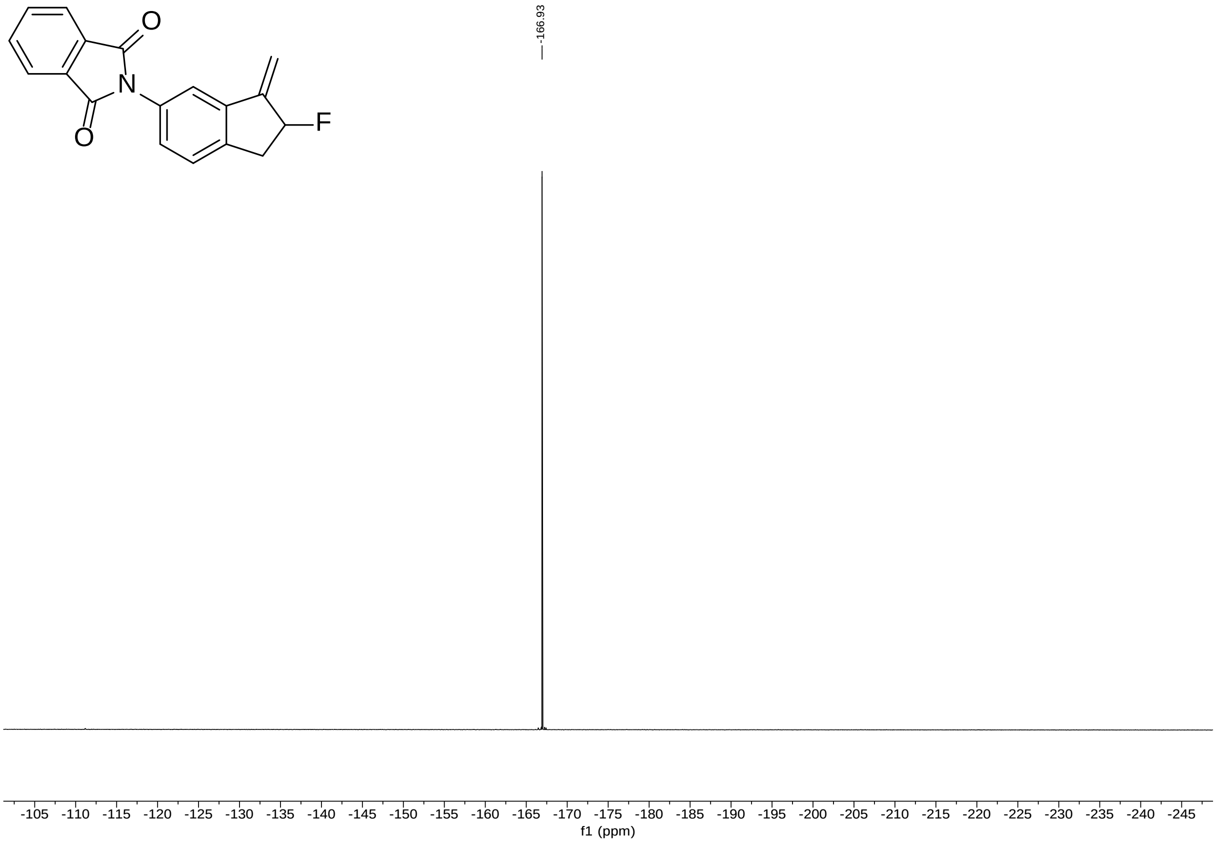

Methyl (*E*)-3-(2-fluoro-2,3-dihydrospiro[indene-1,2'-[1,3-dioxolan]-5-yl)acrylate (S26)

^{1}H NMR (500 MHz, CD_2Cl_2, 299 K)

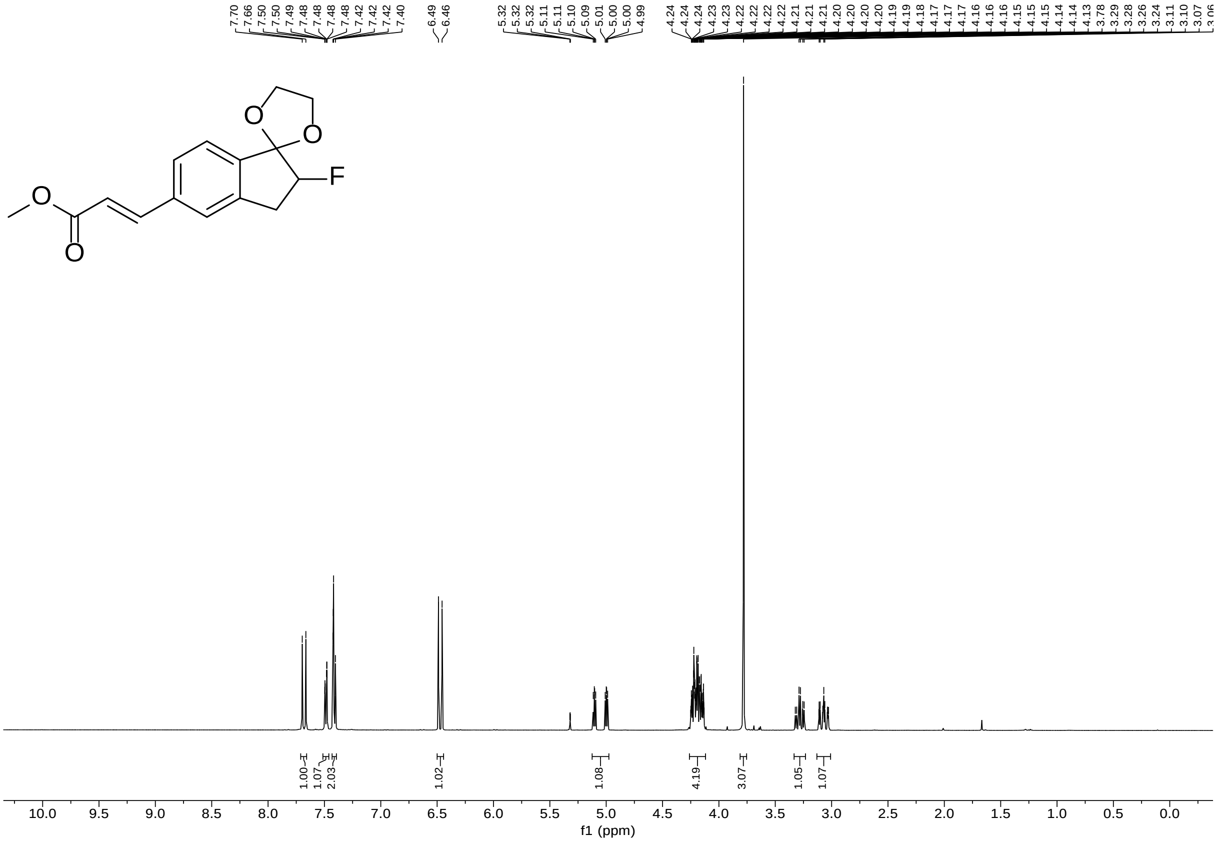

^{13}C NMR (126 MHz, CD_2Cl_2, 299 K)

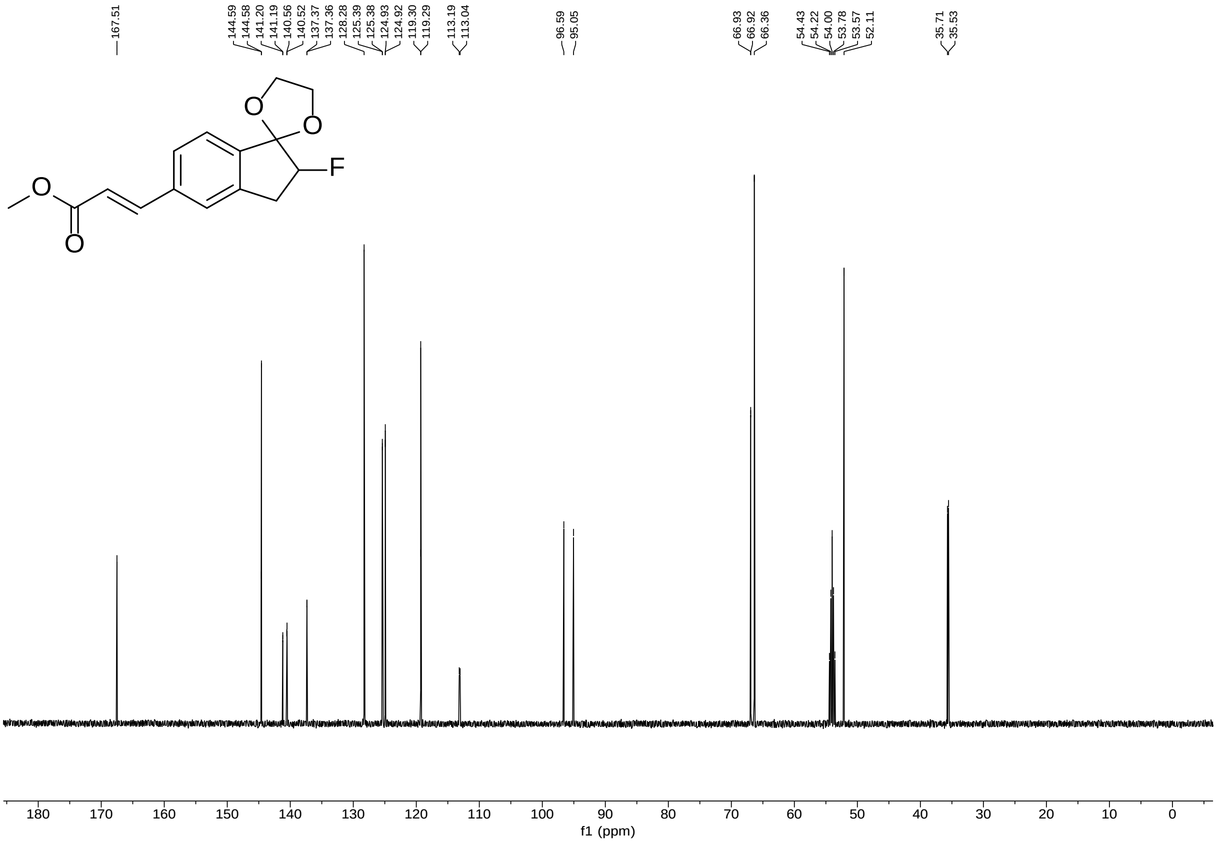

^{19}F NMR (470 MHz, CD_2Cl_2, 299 K)

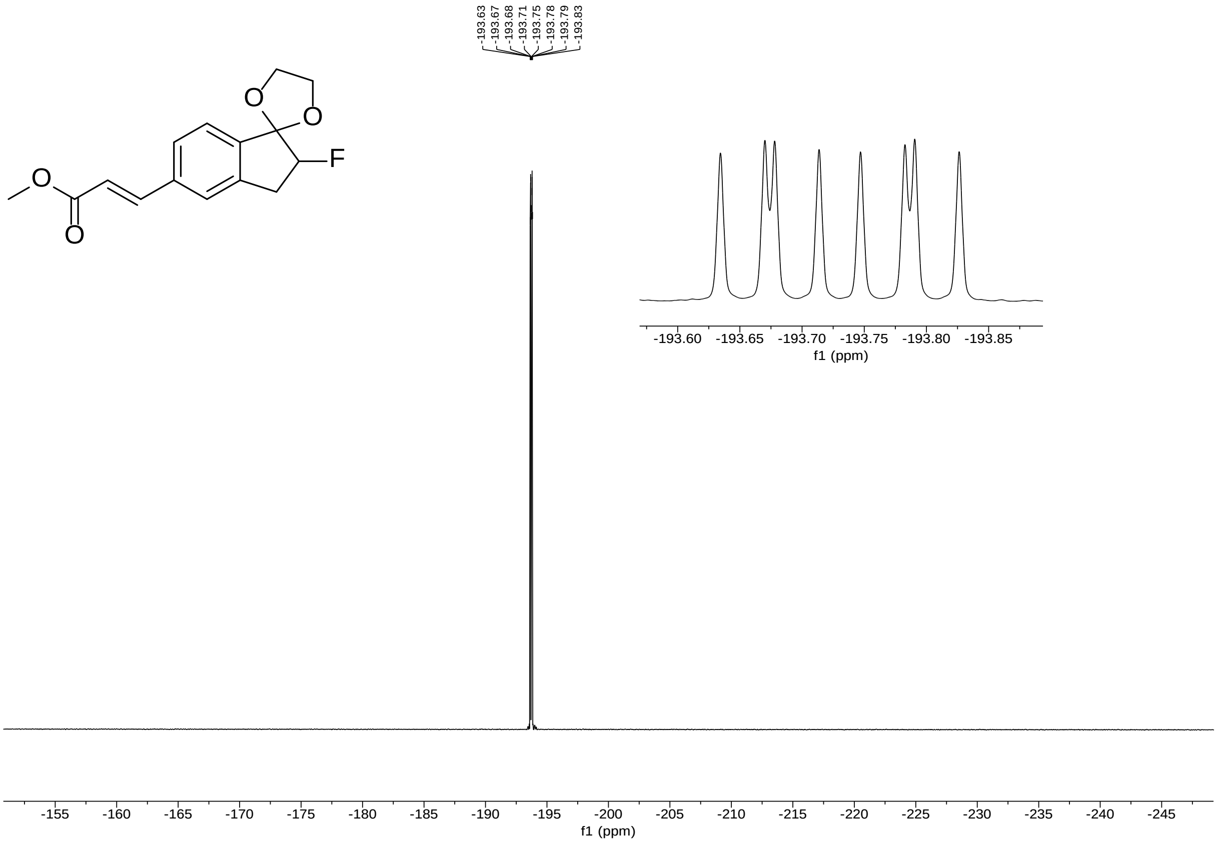

^{19}F NMR{^{1}H} (470 MHz, CD_2Cl_2, 299 K)

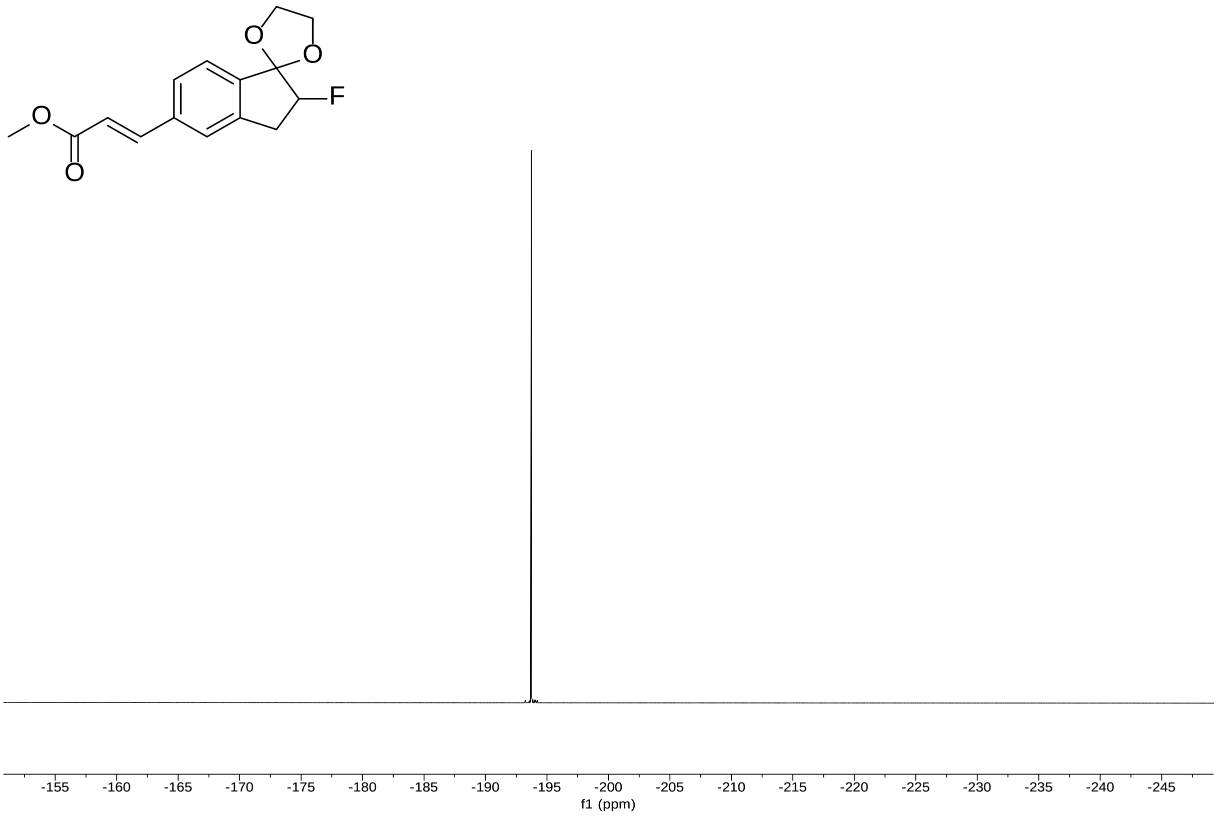

Methyl (*E*)-3-(2-fluoro-1-methylene-2,3-dihydro-1*H*-inden-5-yl)acrylate (S27)

^{1}H NMR (500 MHz, $CDCl_3$, 299 K)

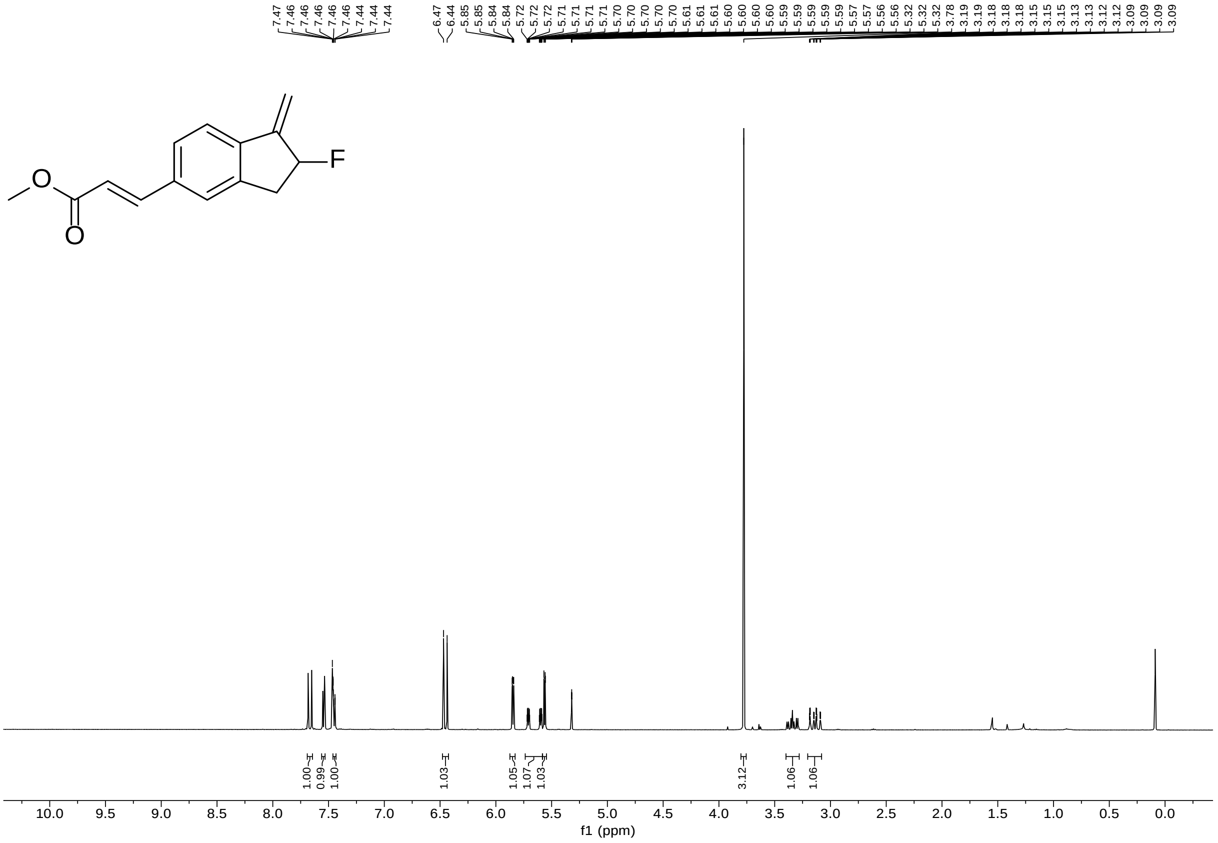

^{13}C NMR (126 MHz, CD_2Cl_2, 299 K)

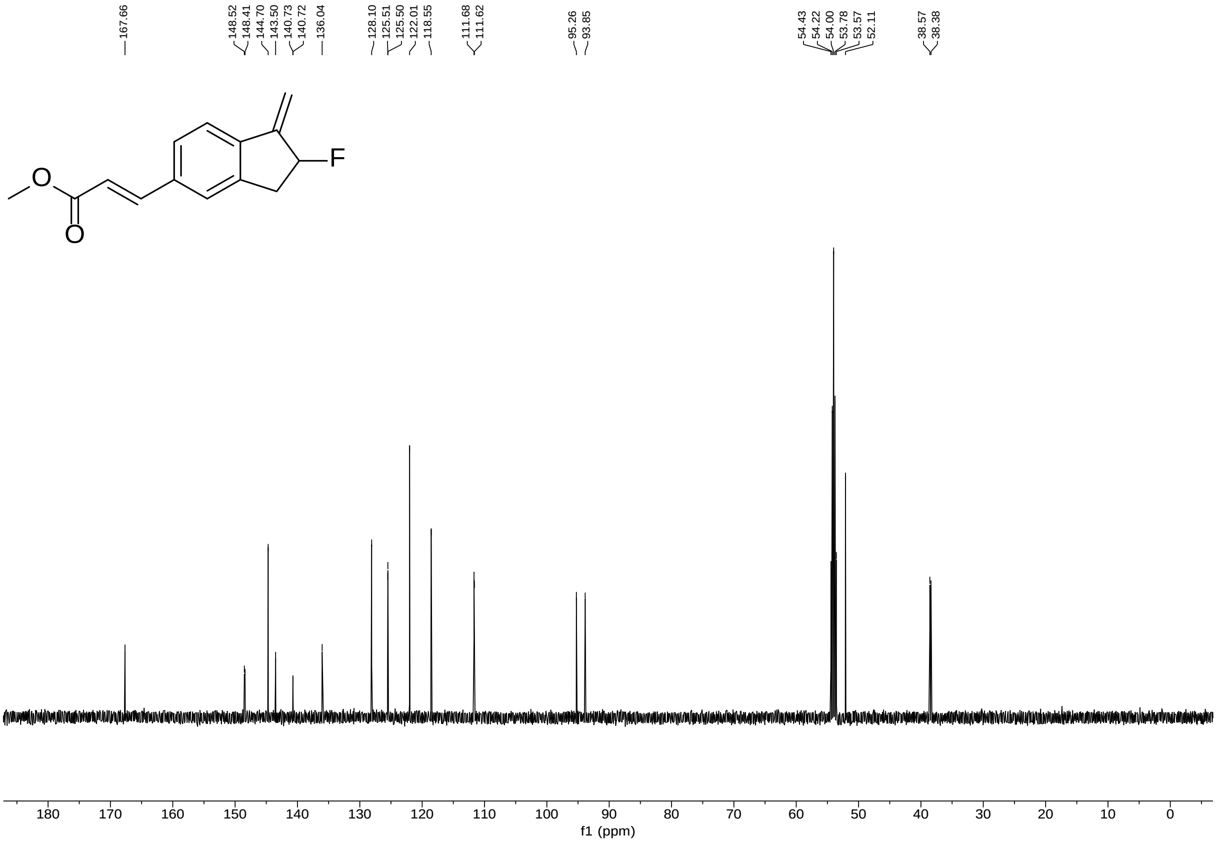

^{19}F NMR (470 MHz, CD_2Cl_2, 299 K)

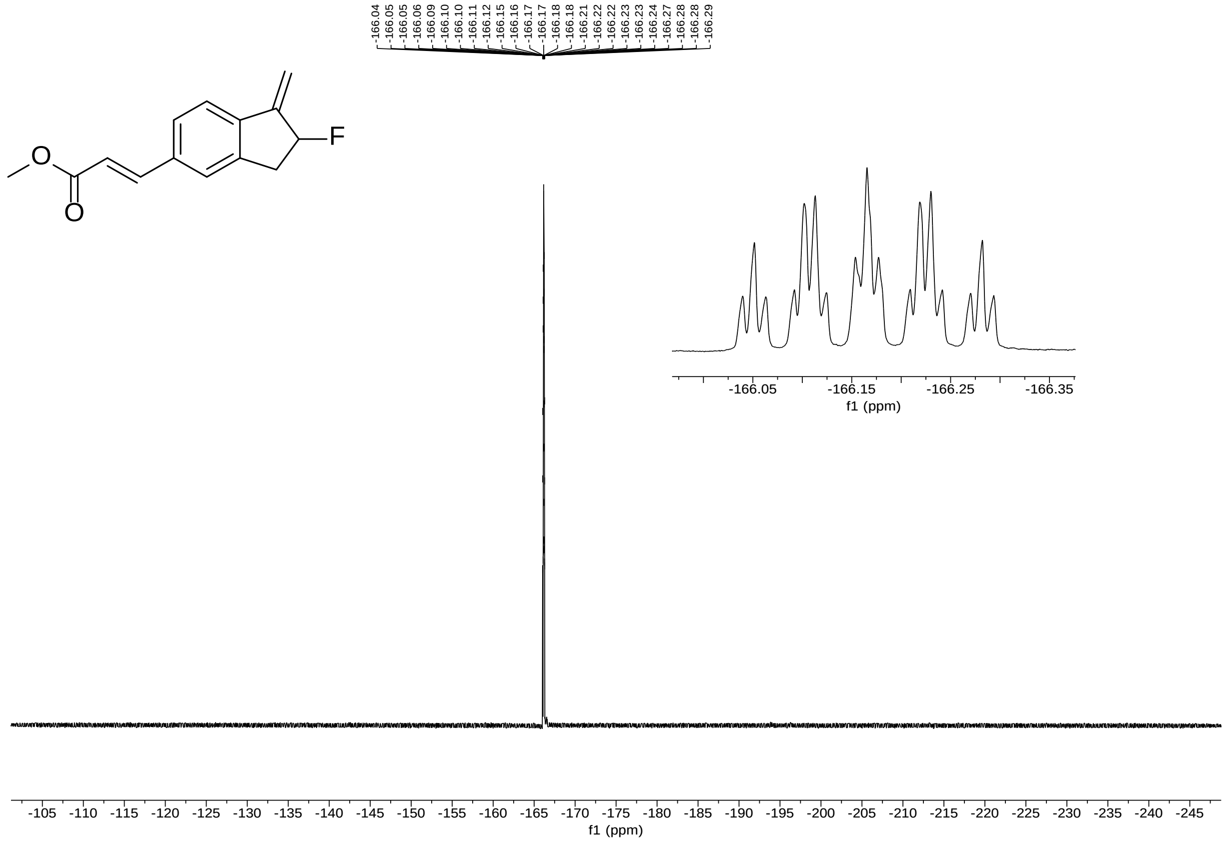

^{19}F NMR{^{1}H} (470 MHz, CD_2Cl_2, 299 K)

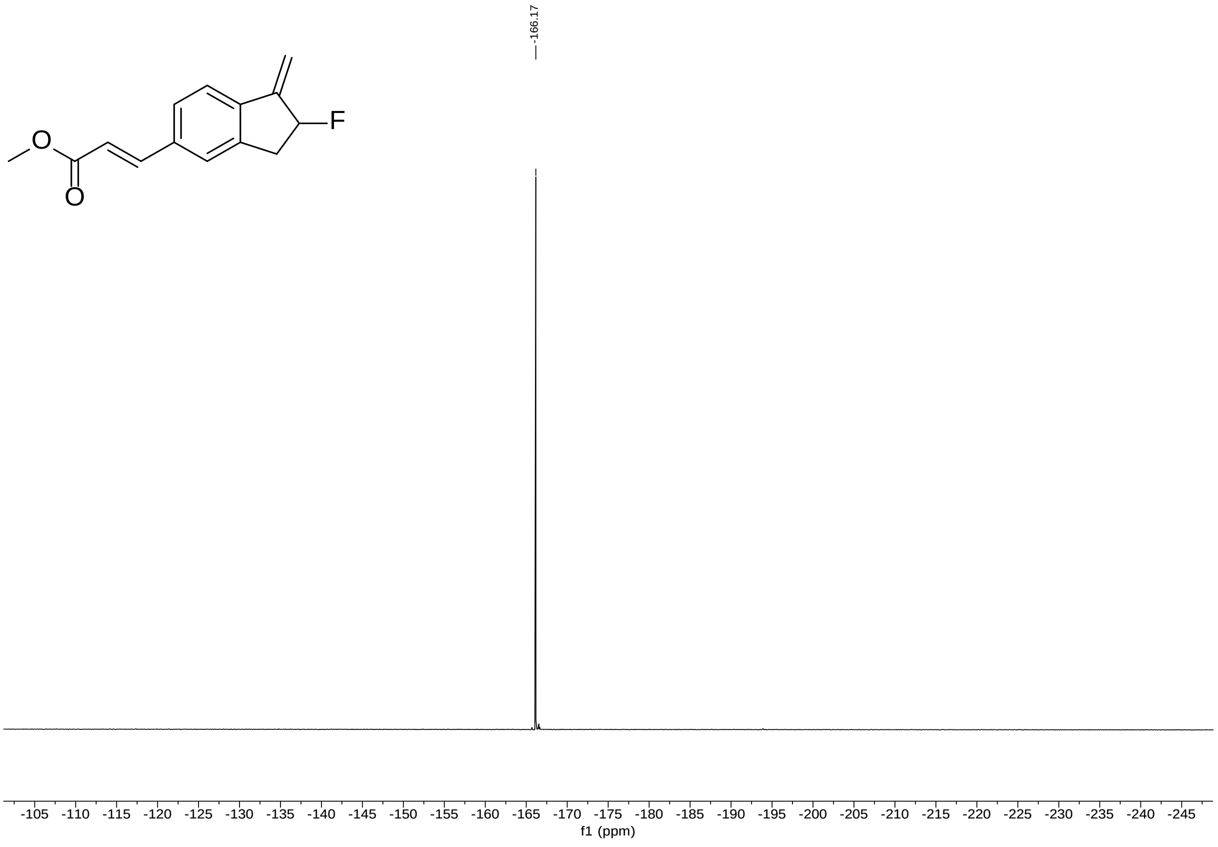

2-Fluoro-3-phenyl-2,3-dihydro-1*H*-inden-1-one (S28)

^{1}H NMR (500 MHz, $CDCl_3$, 299 K)

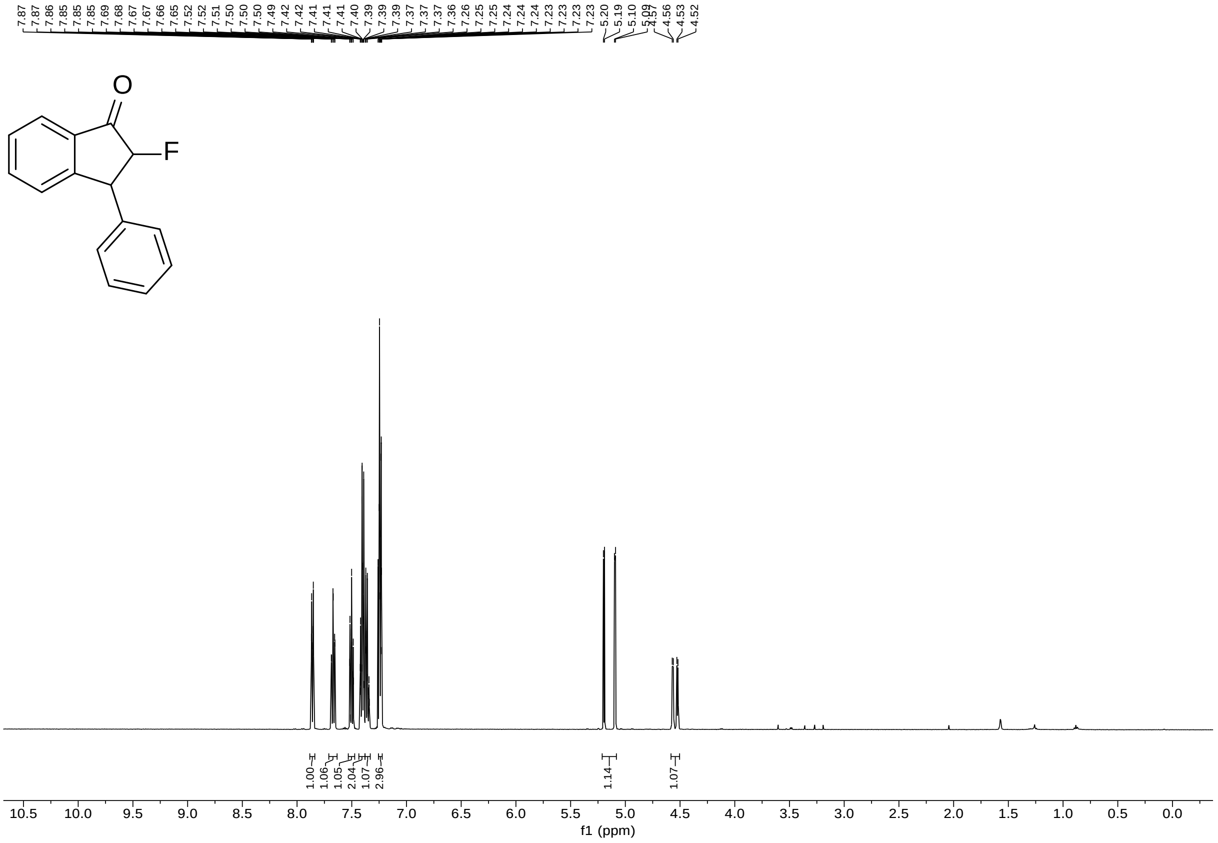

^{13}C NMR (126 MHz, $CDCl_3$, 299 K)

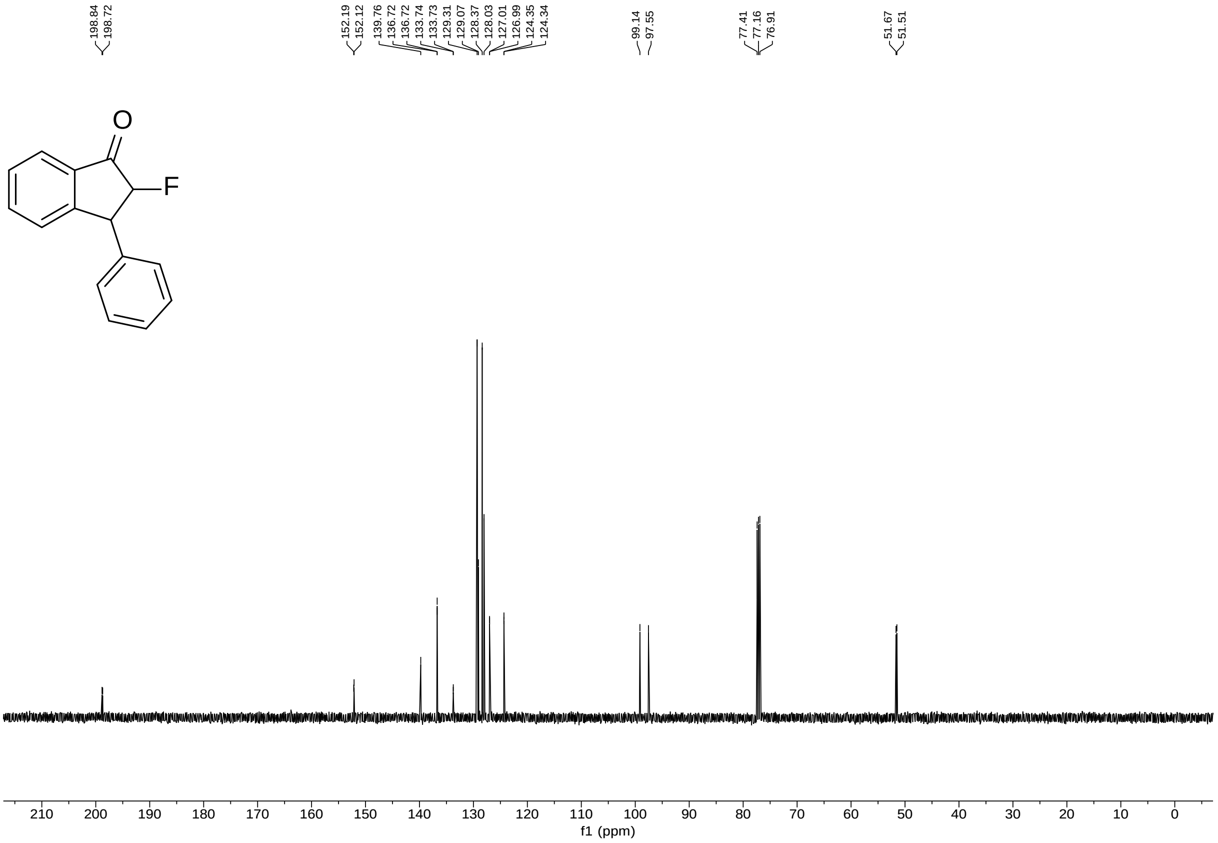

^{19}F NMR (470 MHz, $CDCl_3$, 299 K)

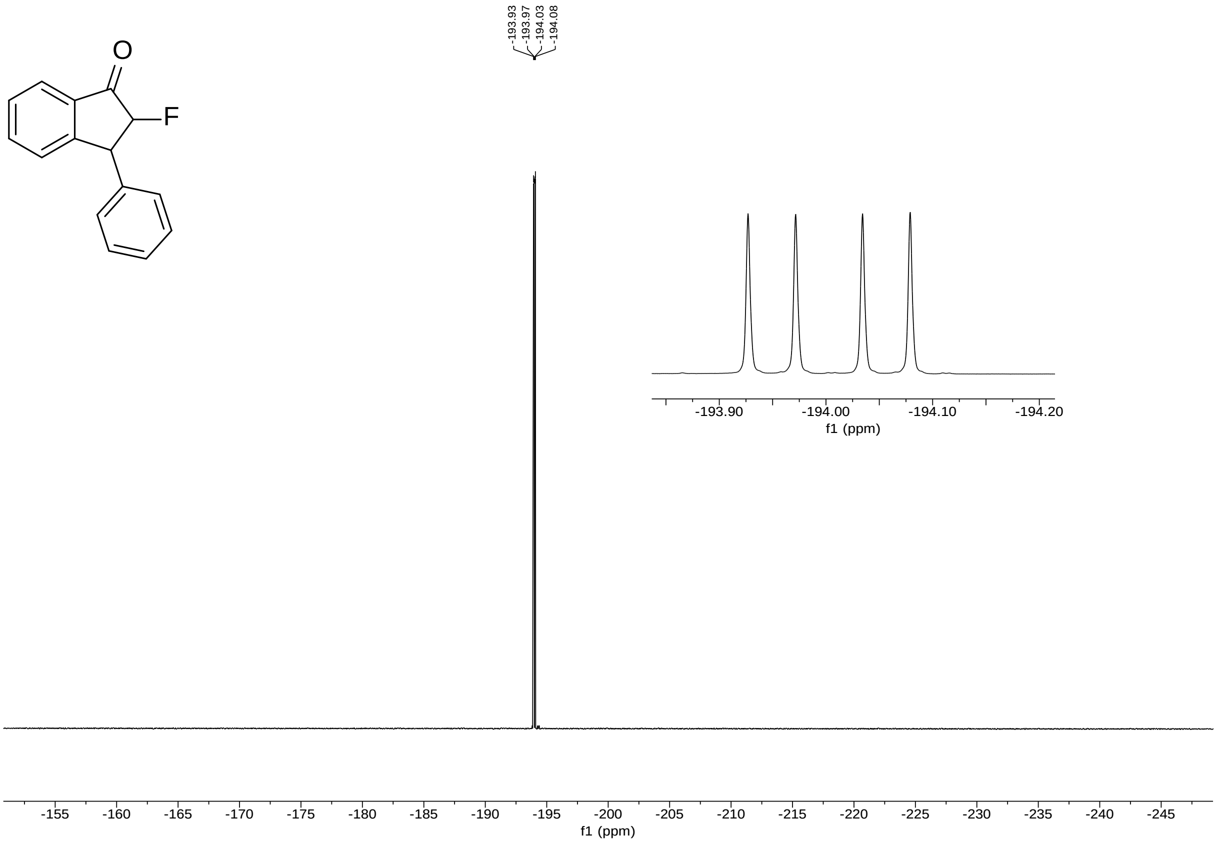

^{19}F{^{1}H} NMR (470 MHz, $CDCl_3$, 299 K)

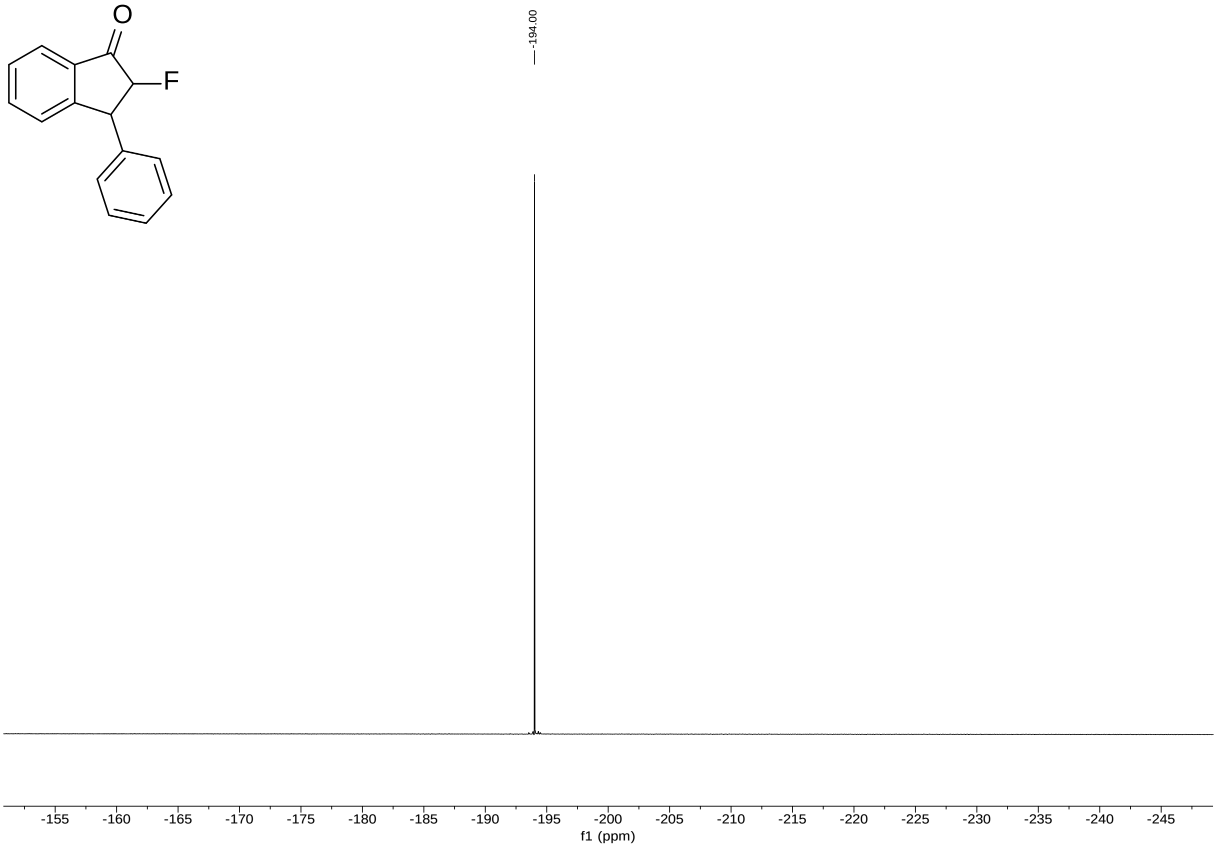

2-Fluoro-1-methylene-3-phenyl-2,3-dihydro-1*H*-indene (S29)

^{1}H NMR (500 MHz, $CDCl_3$, 299 K)

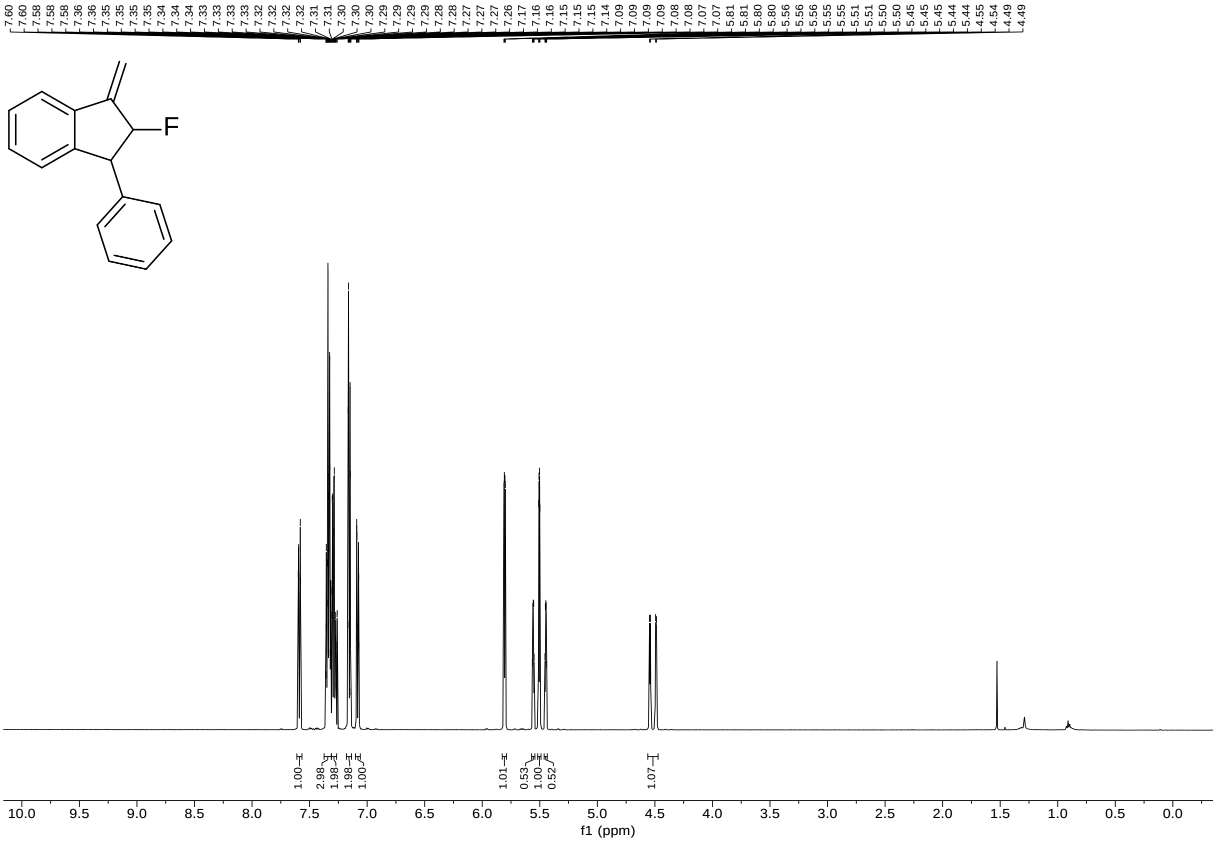

^{13}C NMR (126 MHz, $CDCl_3$, 299 K)

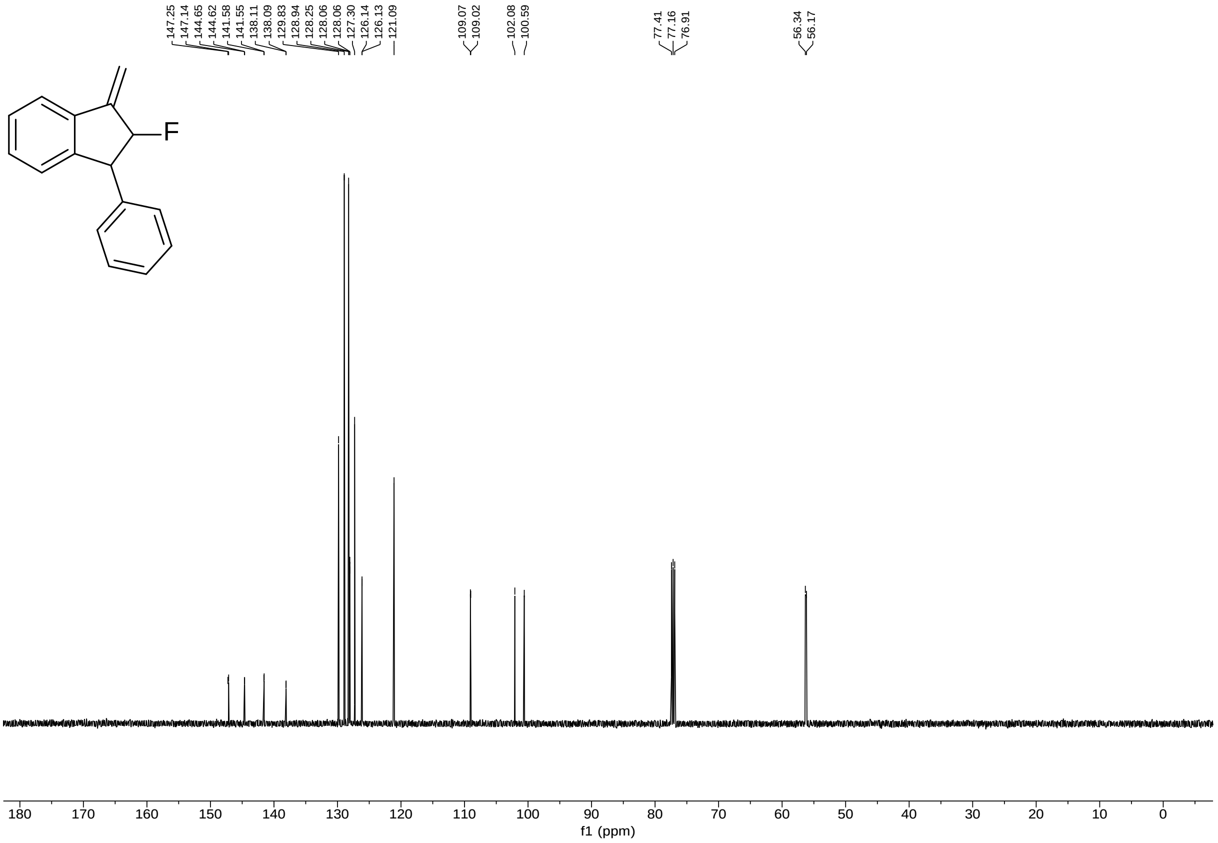

^{19}F NMR (470 MHz, $CDCl_3$, 299 K)

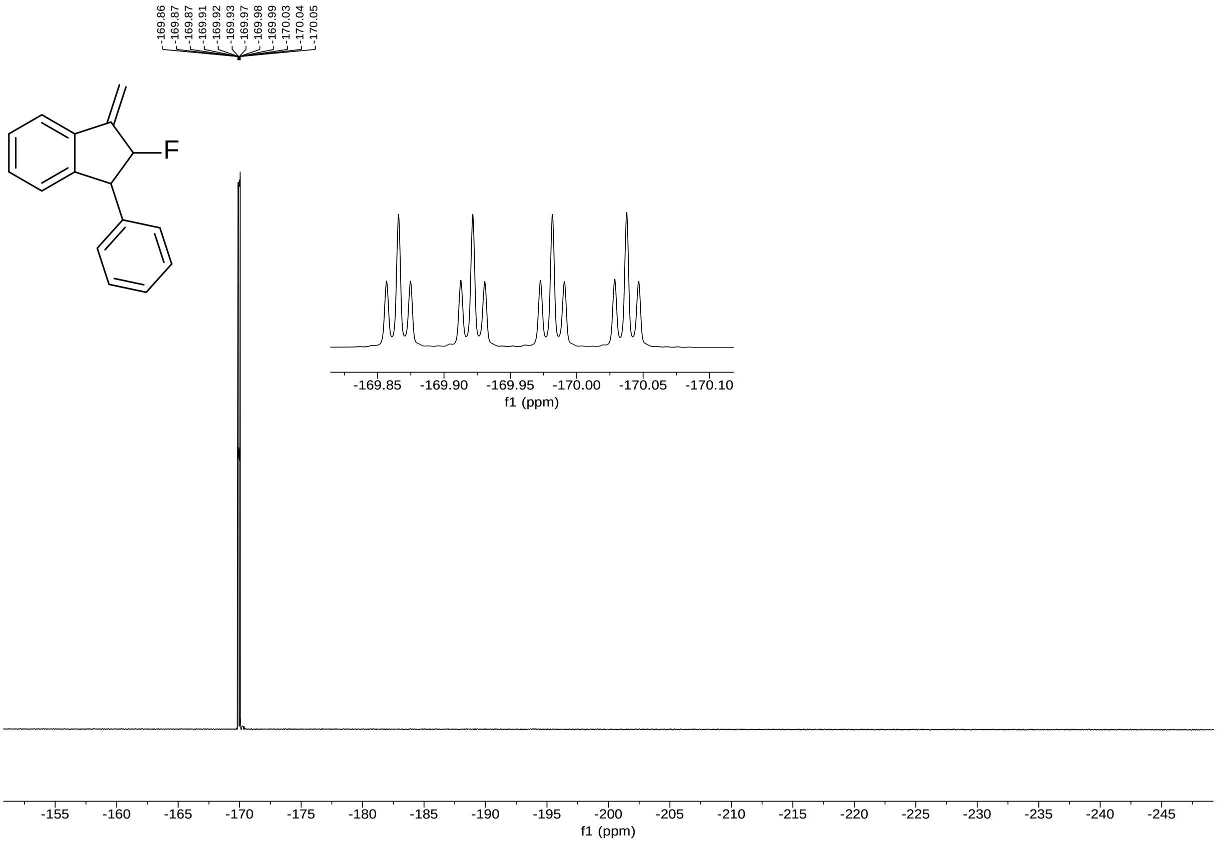

^{19}F{^{1}H} NMR (470 MHz, $CDCl_3$, 299 K)

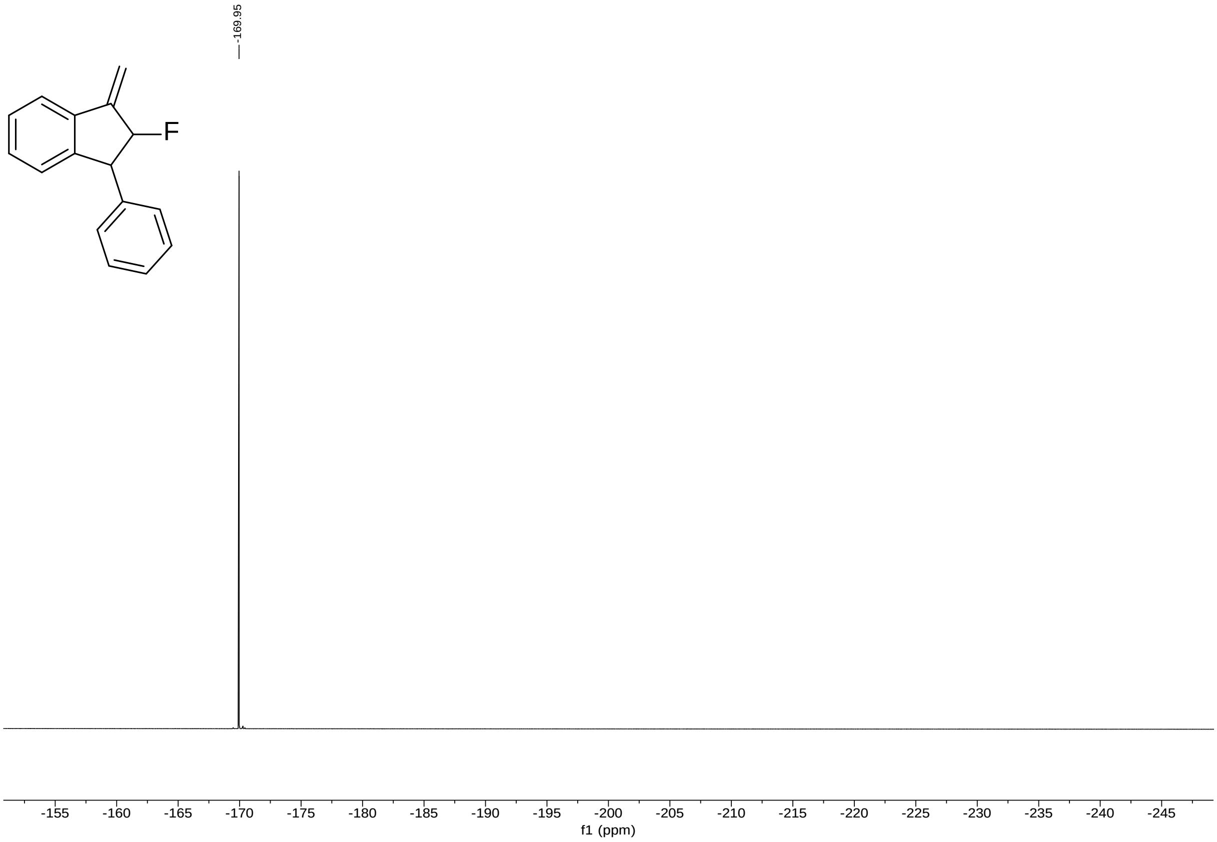

2-Fluoro-3,3-dimethyl-2,3-dihydro-1*H*-inden-1-one (S30)

^{1}H NMR (500 MHz, $CDCl_3$, 299 K)

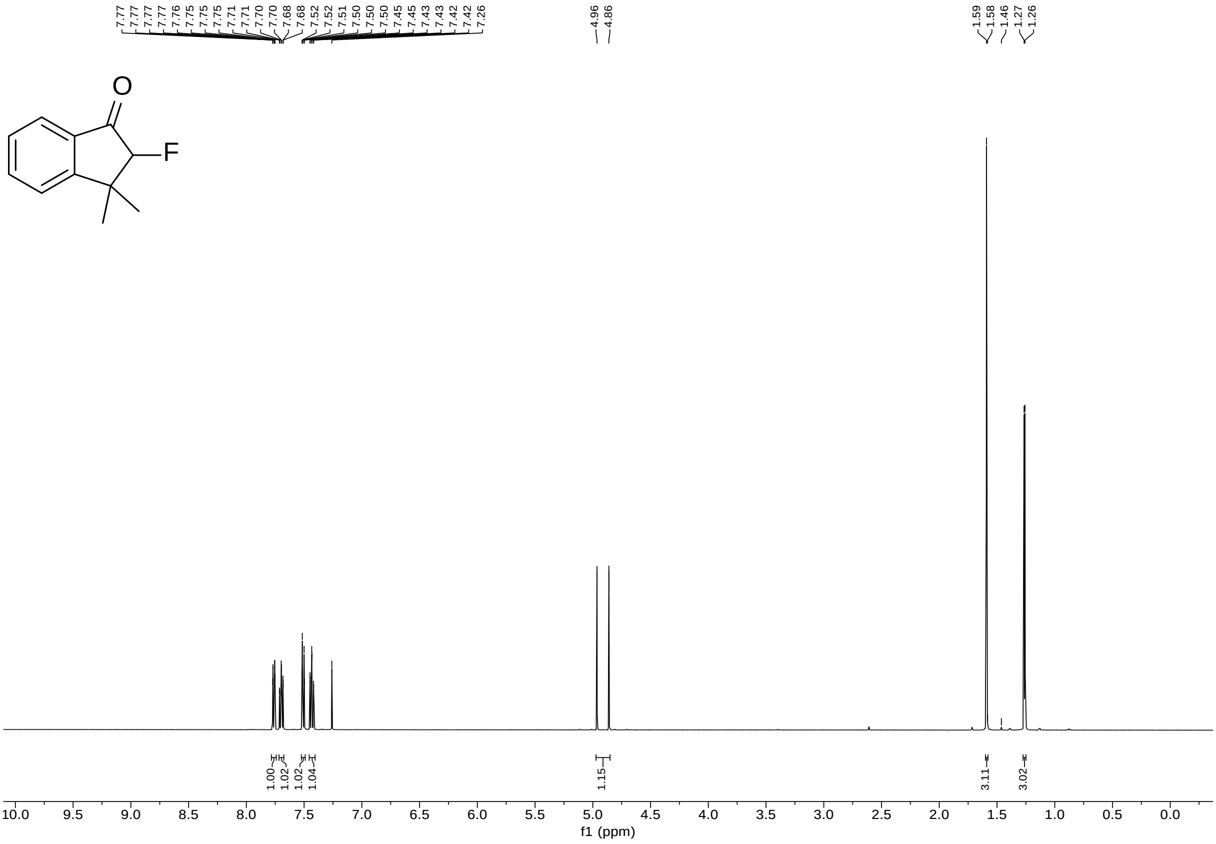

^{13}C NMR (126 MHz, $CDCl_3$, 299 K)

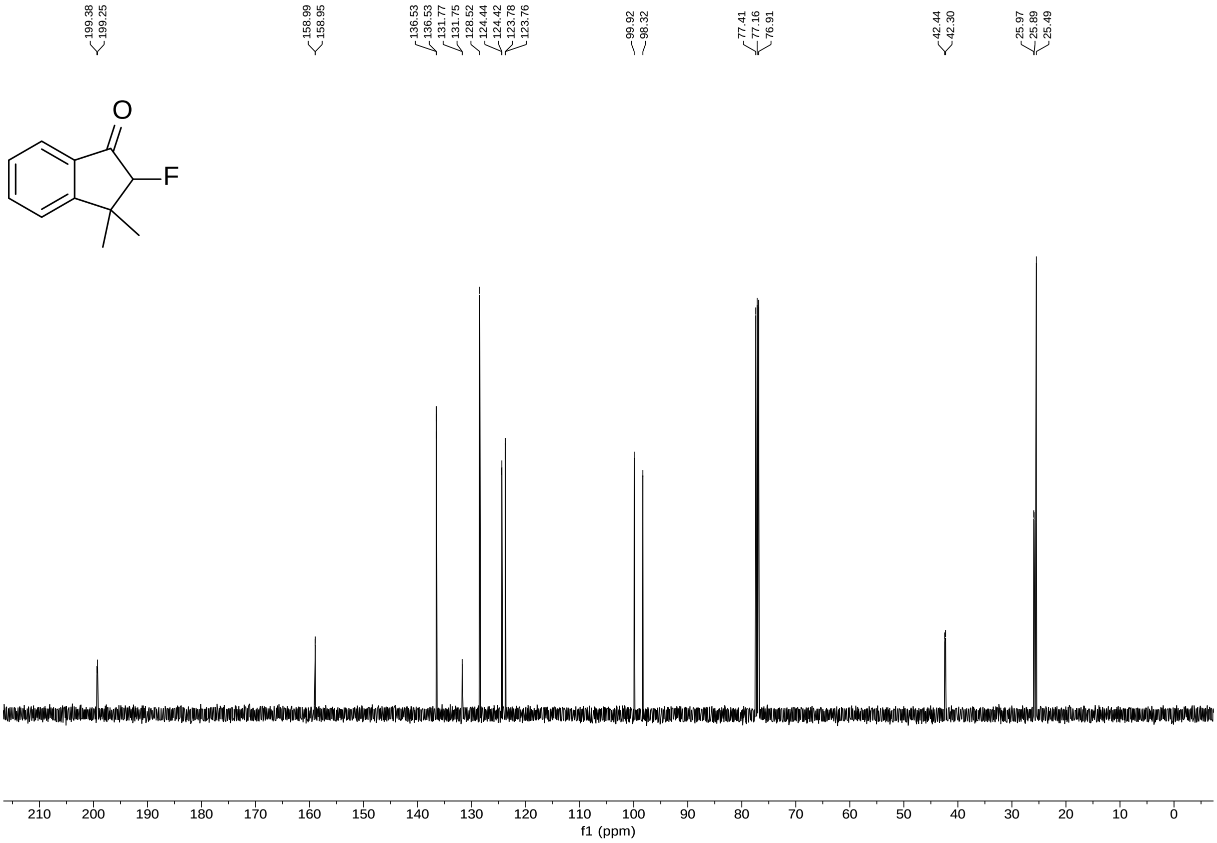

^{19}F NMR (470 MHz, $CDCl_3$, 299 K)

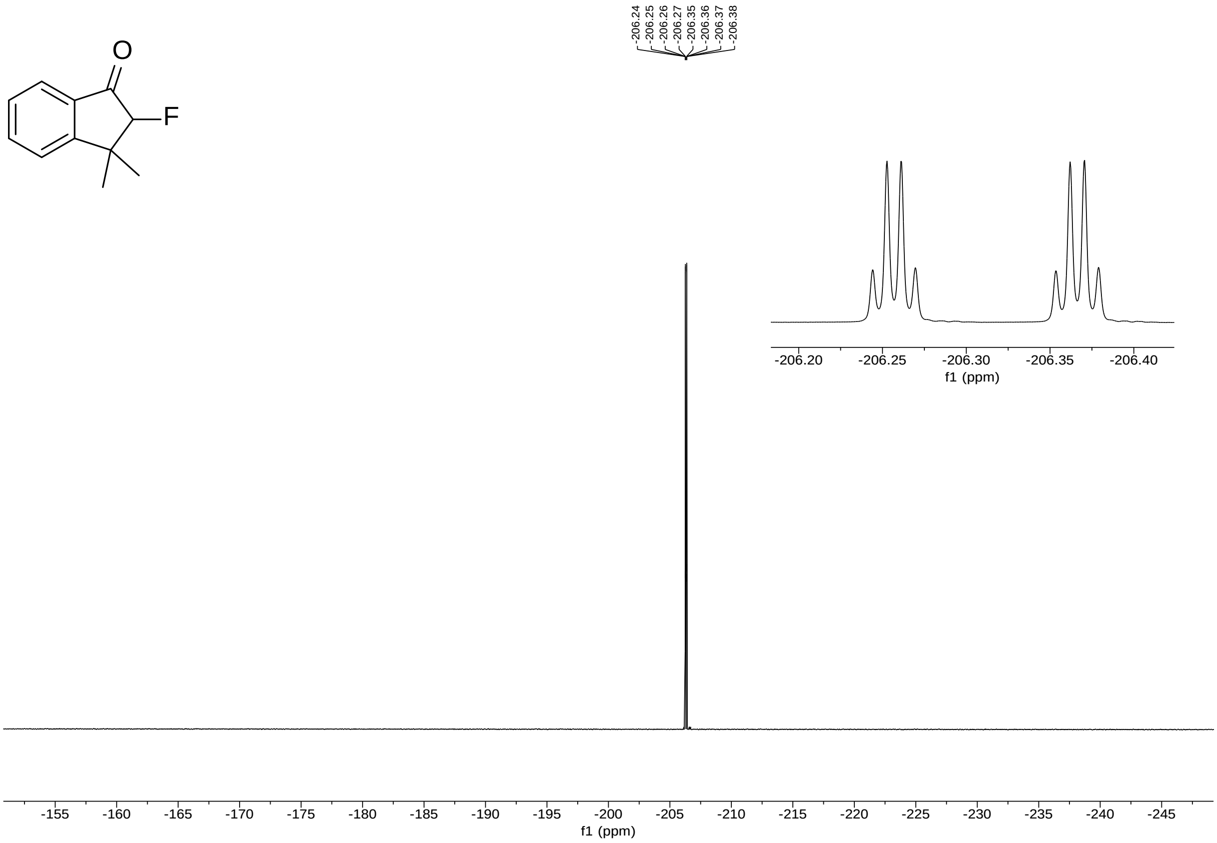

^{19}F{^{1}H} NMR (470 MHz, $CDCl_3$, 299 K)

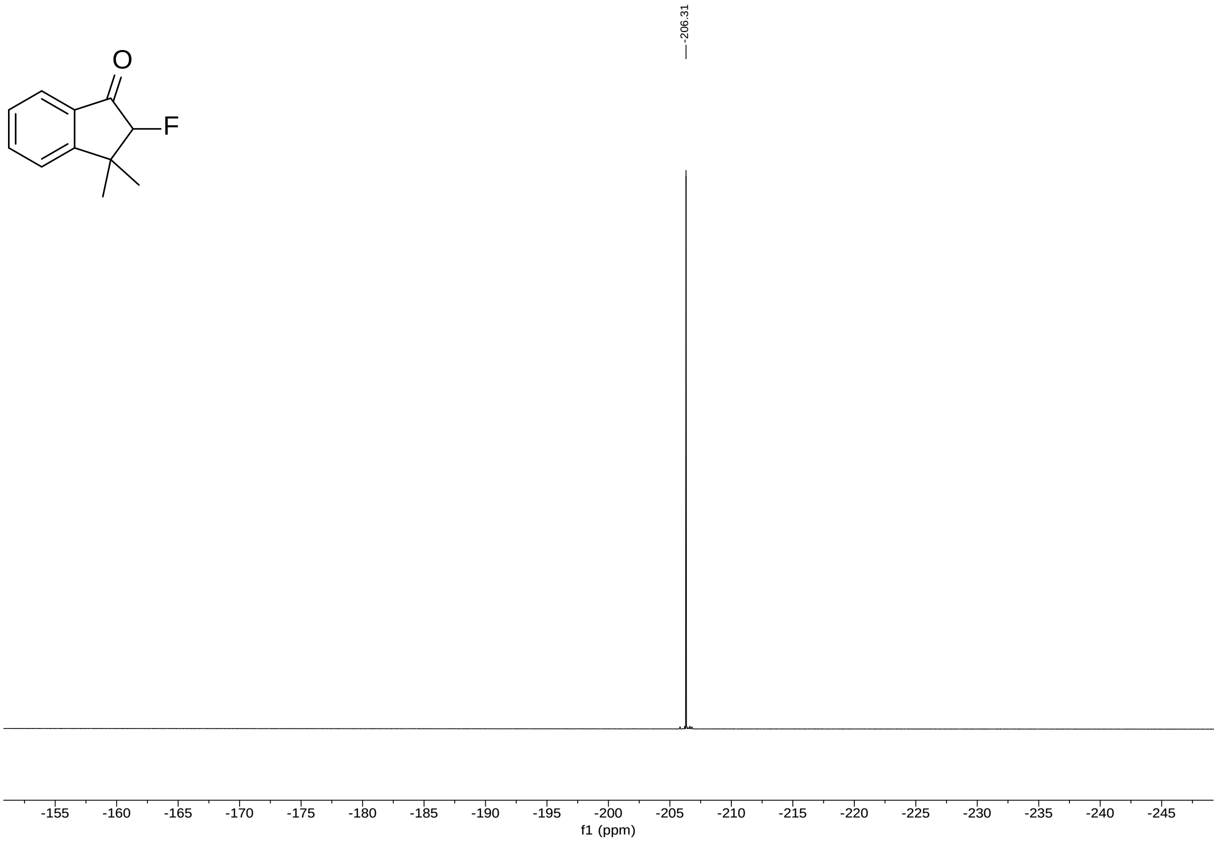

2-Fluoro-1,1-dimethyl-3-methylene-2,3-dihydro-1*H*-indene (S31)

^{1}H NMR (500 MHz, $CDCl_3$, 299 K)

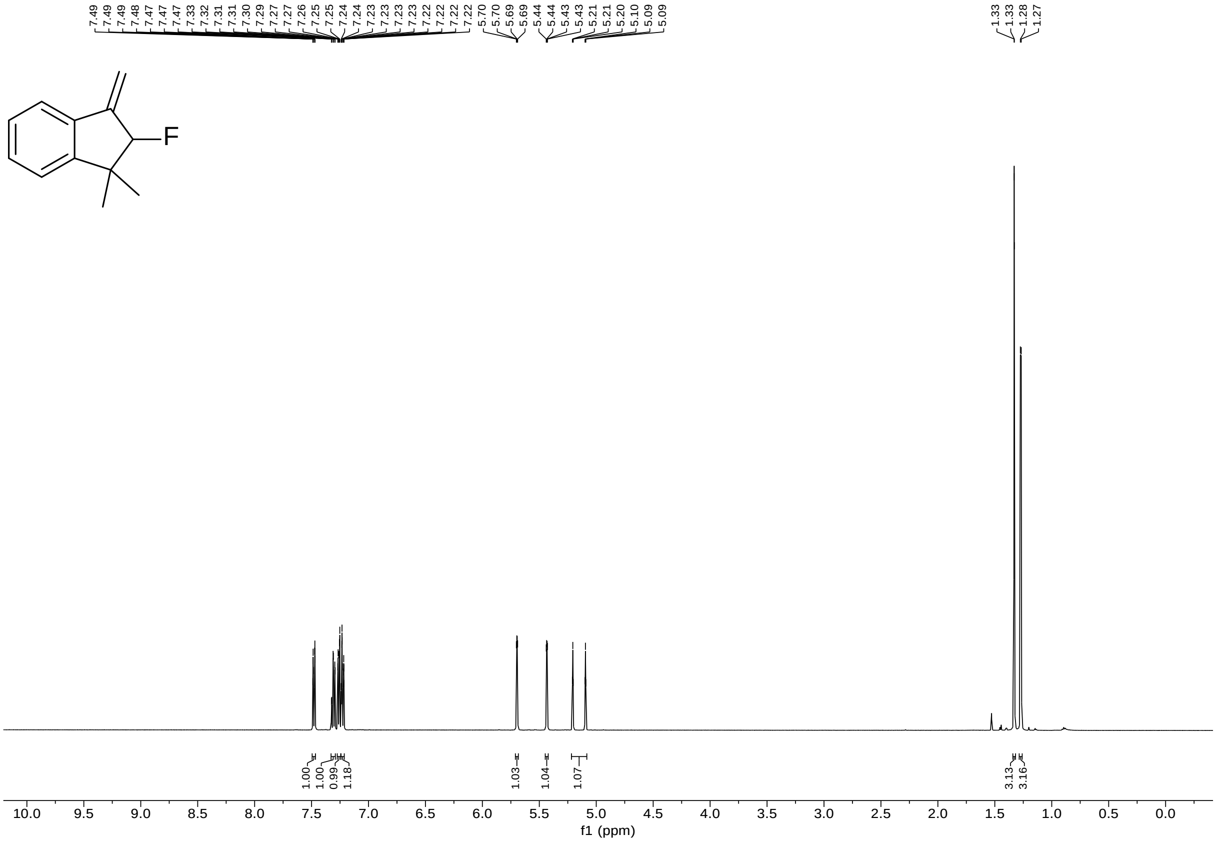

^{13}C NMR (126 MHz, $CDCl_3$, 299 K)

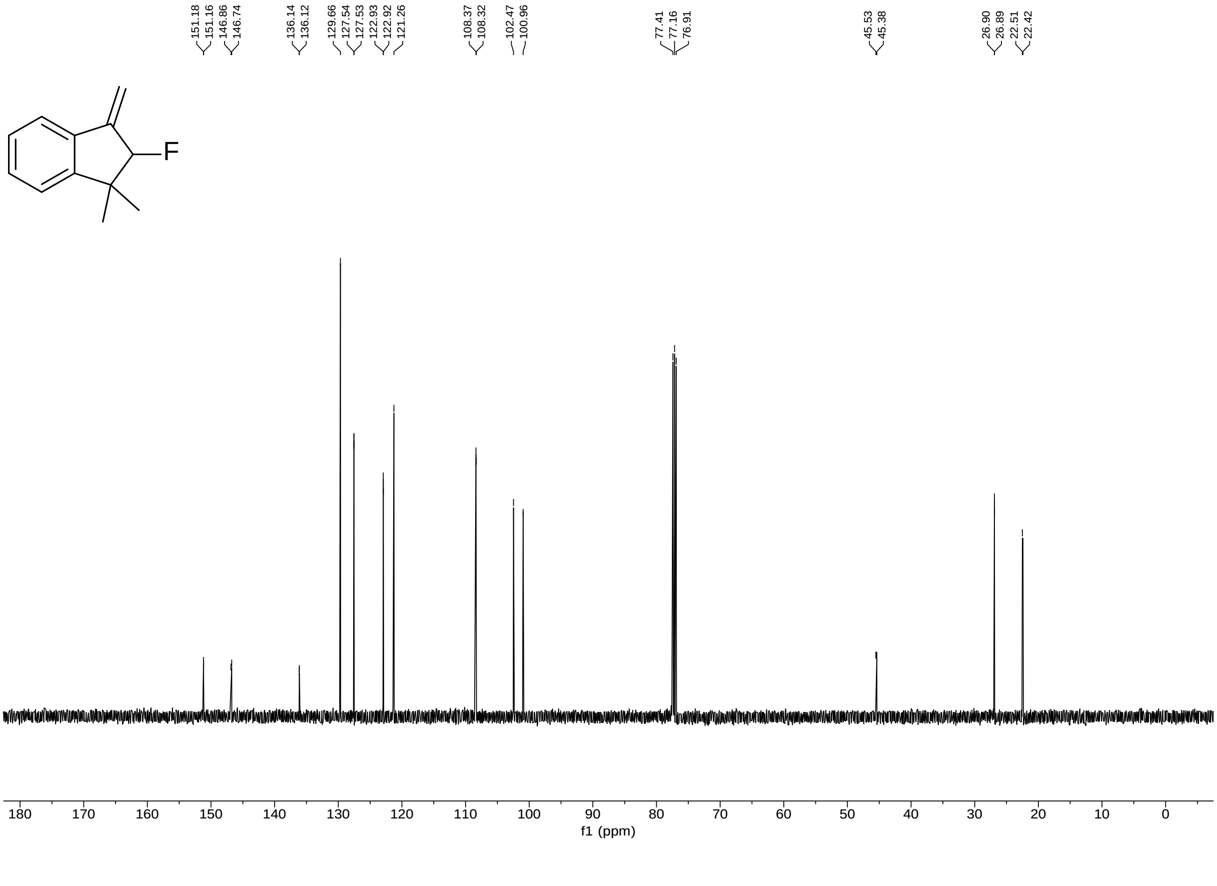

^{19}F NMR (470 MHz, $CDCl_3$, 299 K)

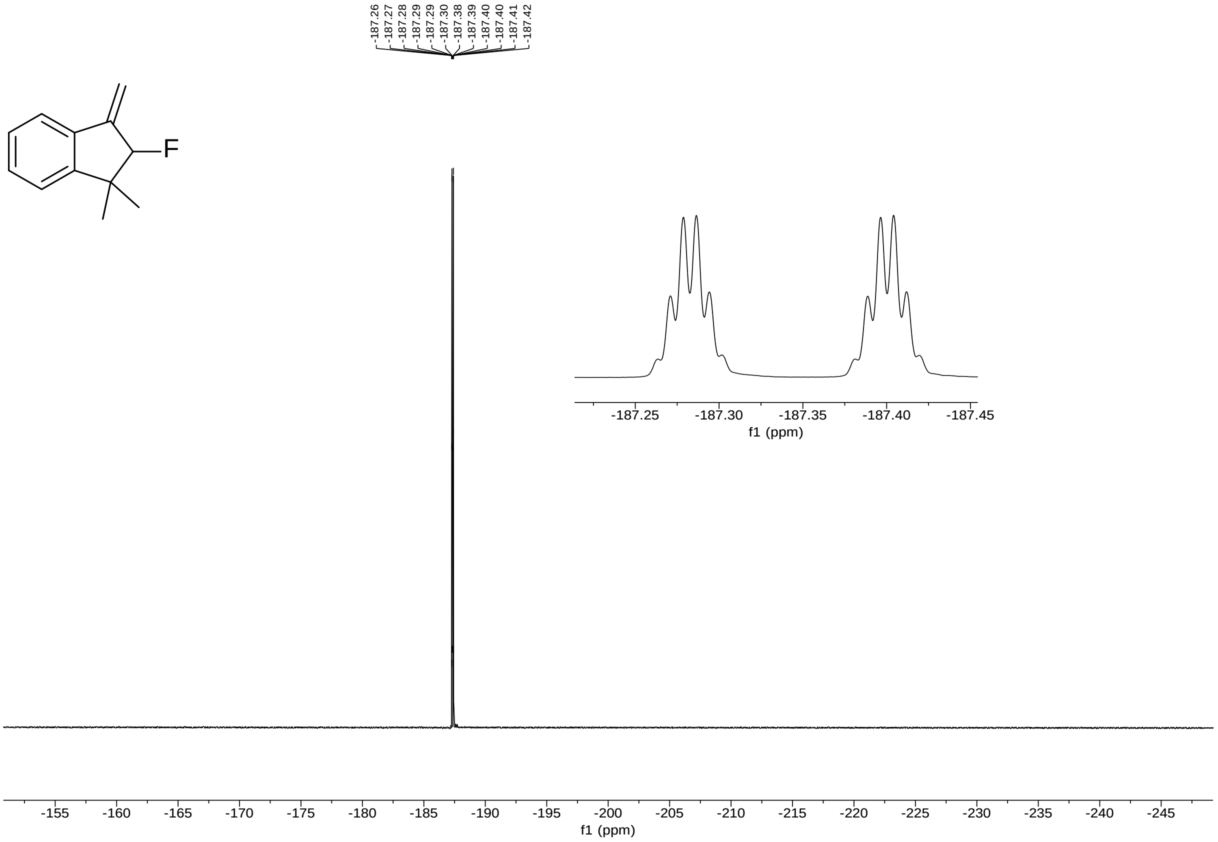

^{19}F{^{1}H} NMR (470 MHz, $CDCl_3$, 299 K)

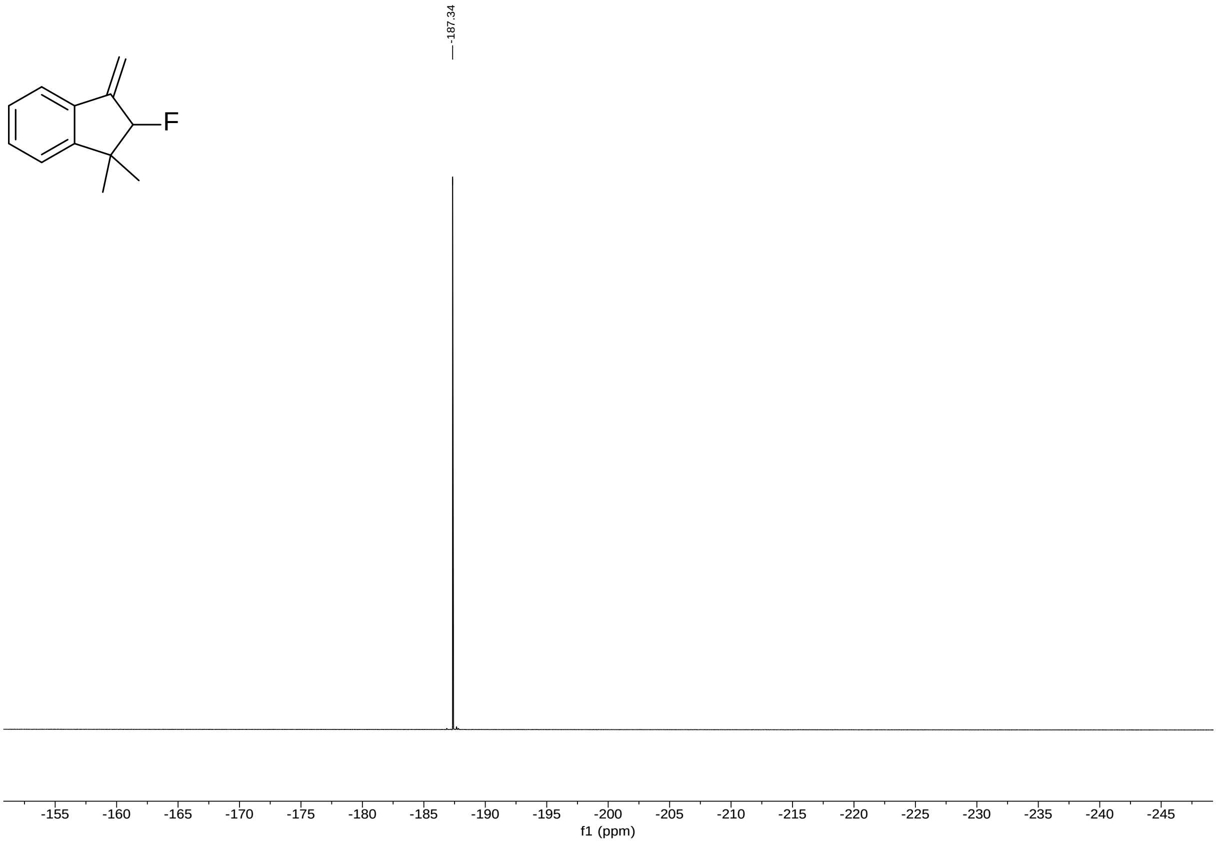

2-Chloro-1-methylene-2,3-dihydro-1*H*-indene (S35)

^{1}H NMR (500 MHz, $CDCl_3$, 299 K)

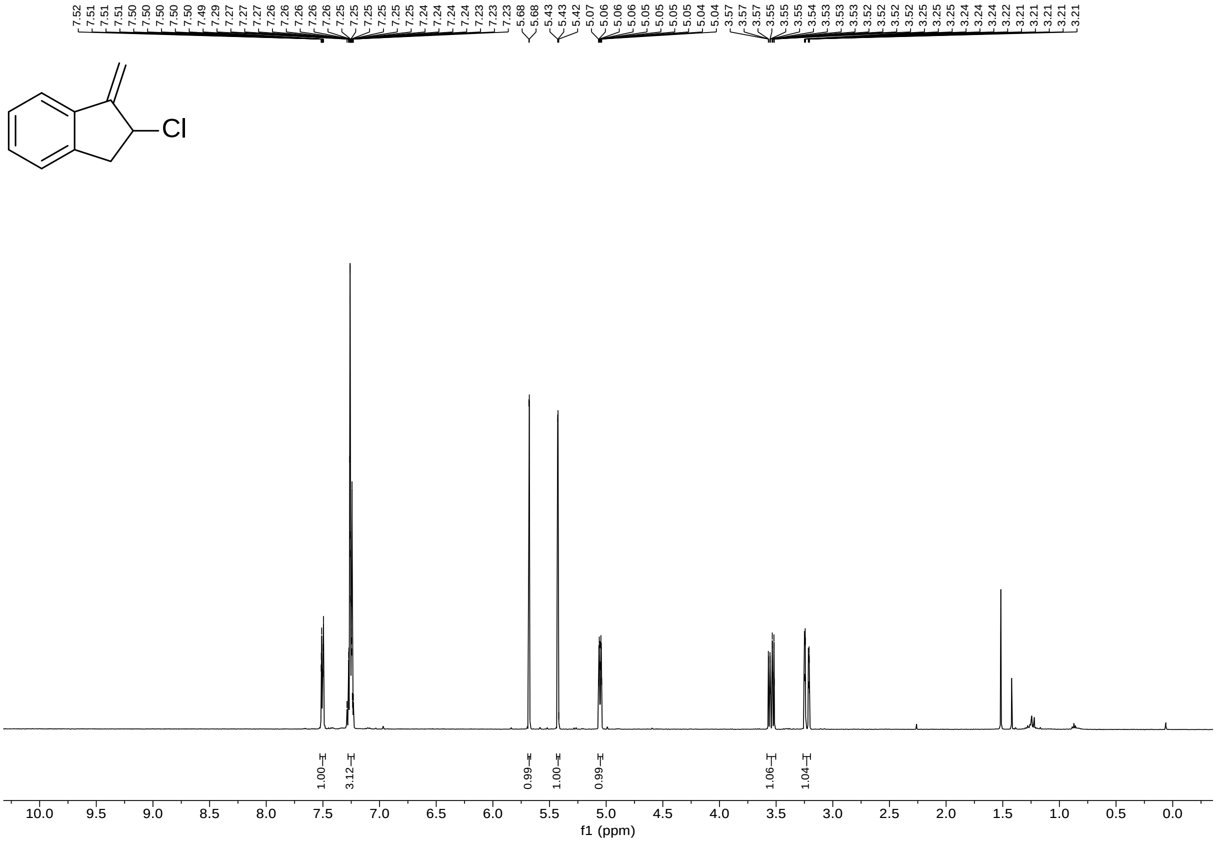

^{13}C NMR (126 MHz, $CDCl_3$, 299 K)

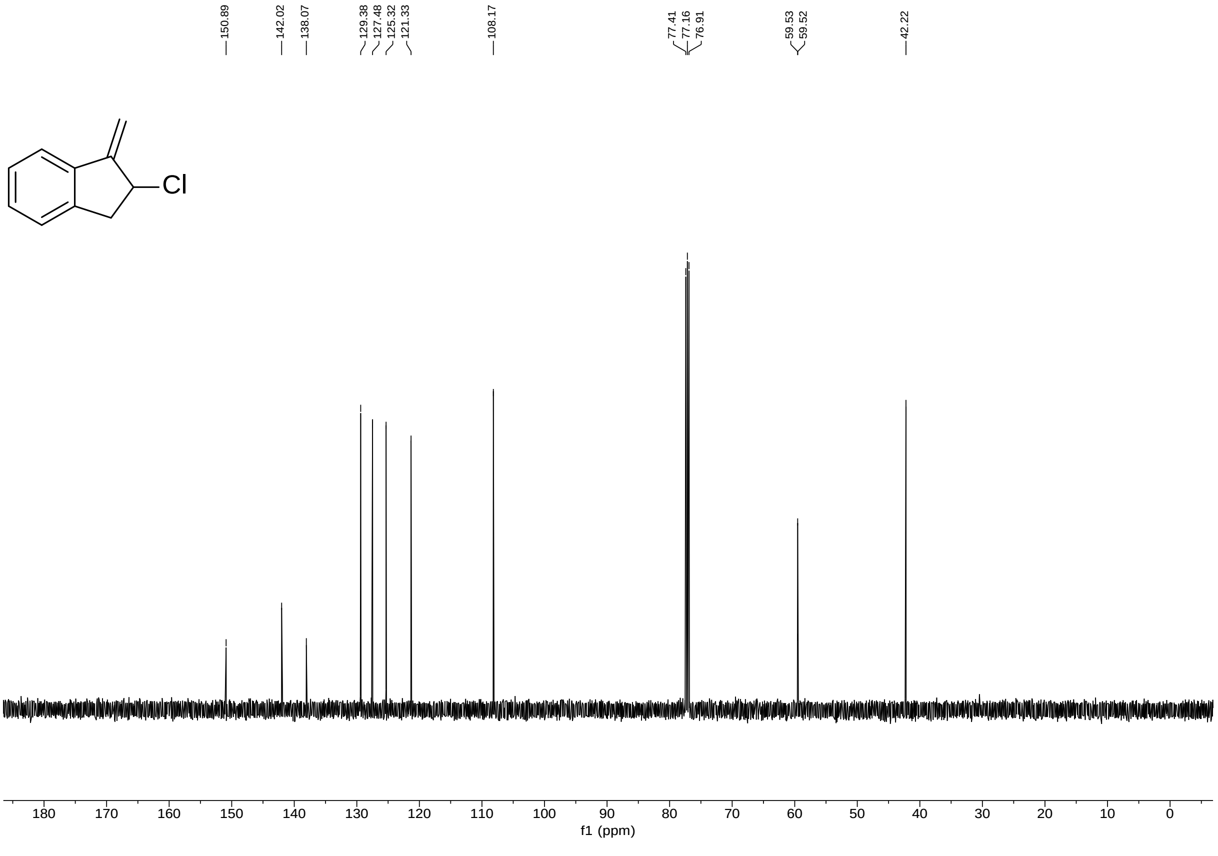

Methyl (*R*)-2-fluoro-1-methylene-2,3-dihydro-1*H*-indene-2-carboxylate (S40)

^{1}H NMR (500 MHz, $CDCl_3$, 299 K)

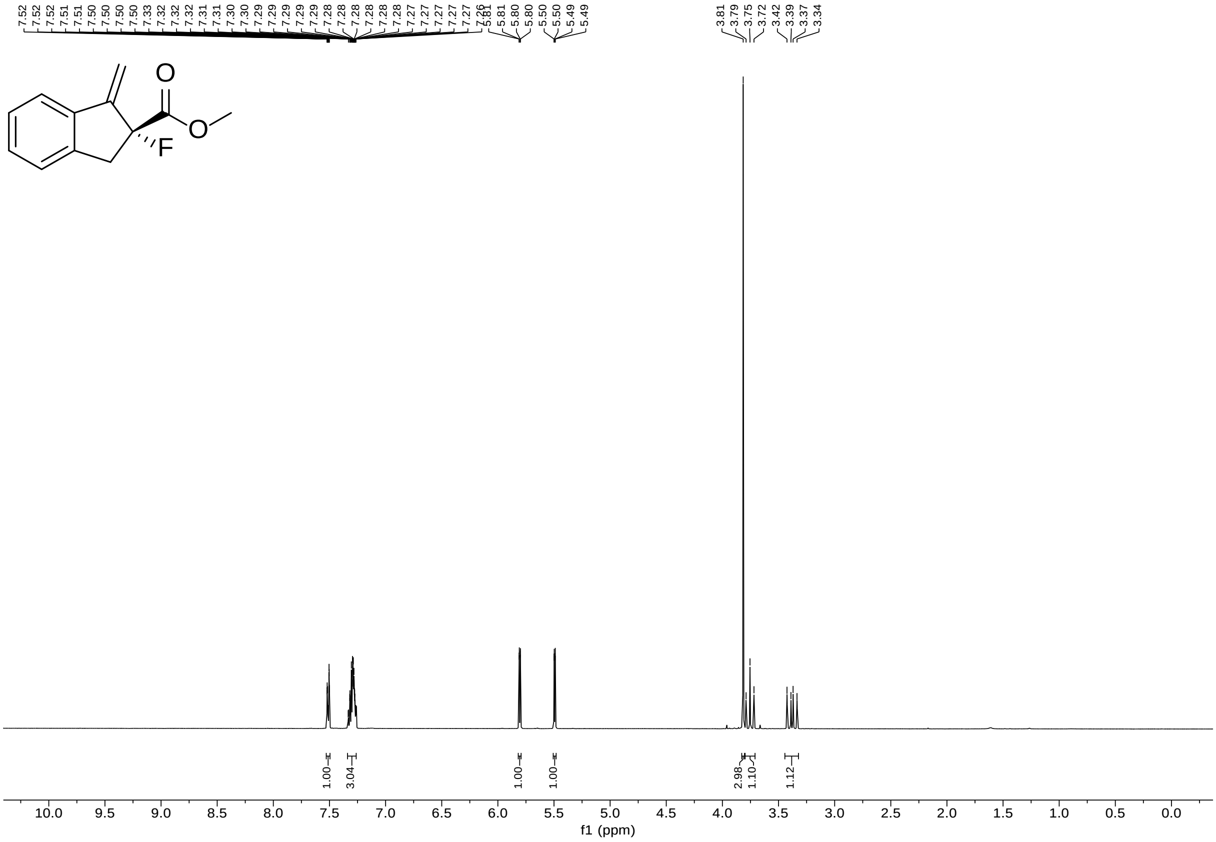

^{13}C NMR (126 MHz, $CDCl_3$, 299 K)

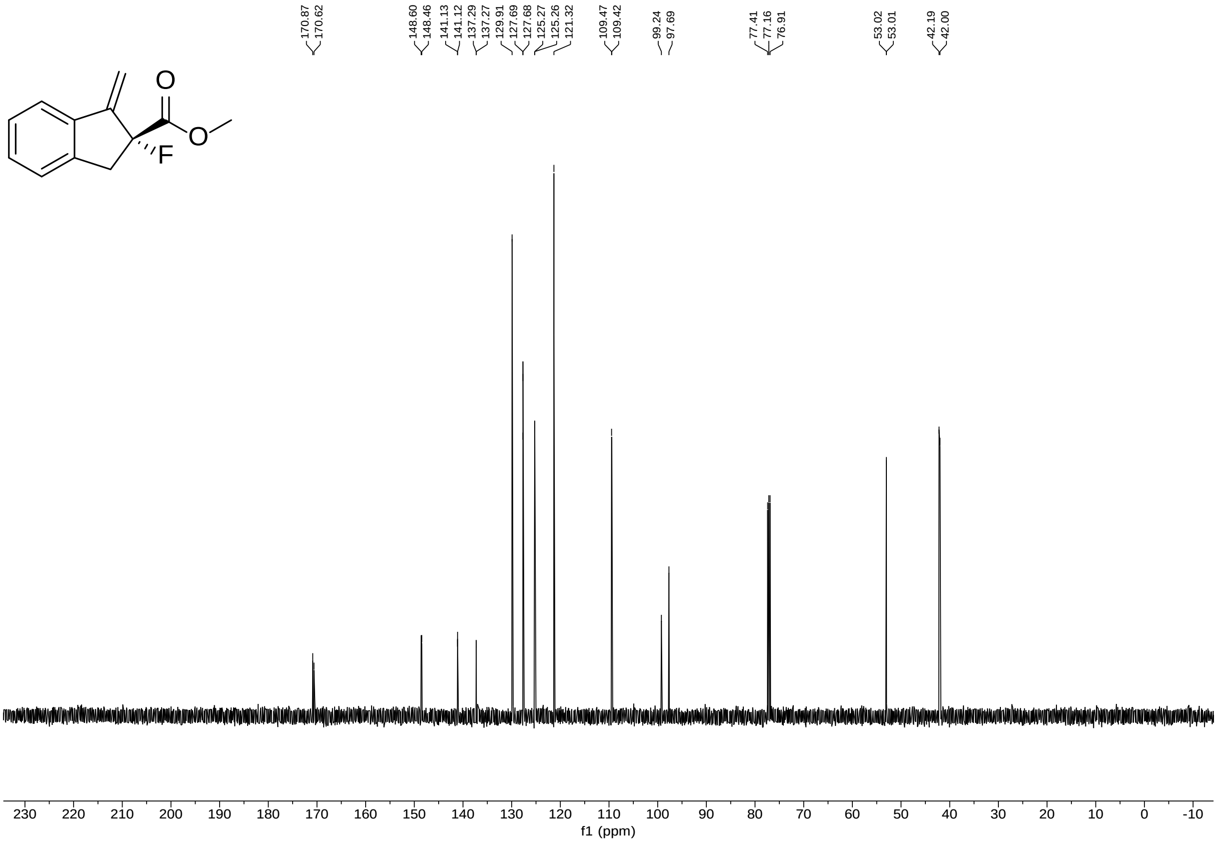

^{19}F NMR (470 MHz, $CDCl_3$, 299 K)

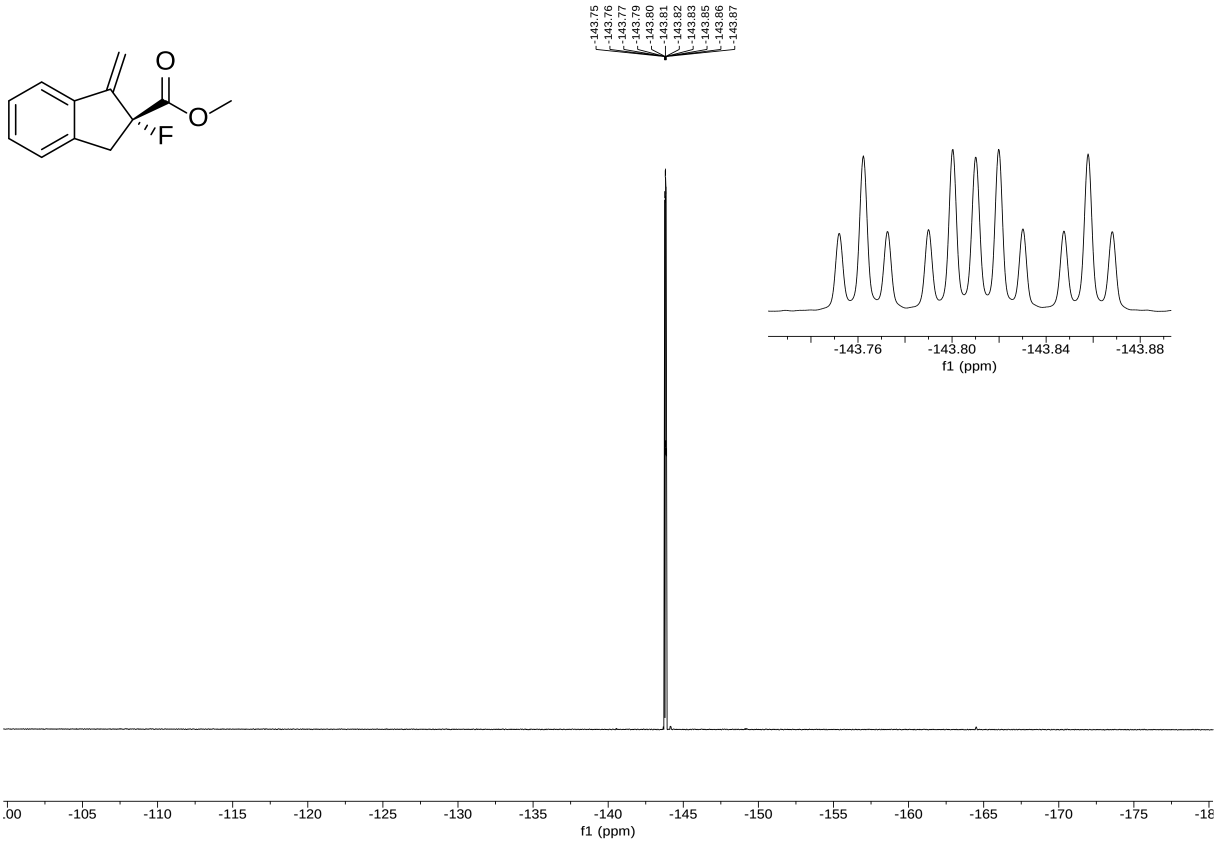

^{19}F{^{1}H} NMR (470 MHz, $CDCl_3$, 299 K)

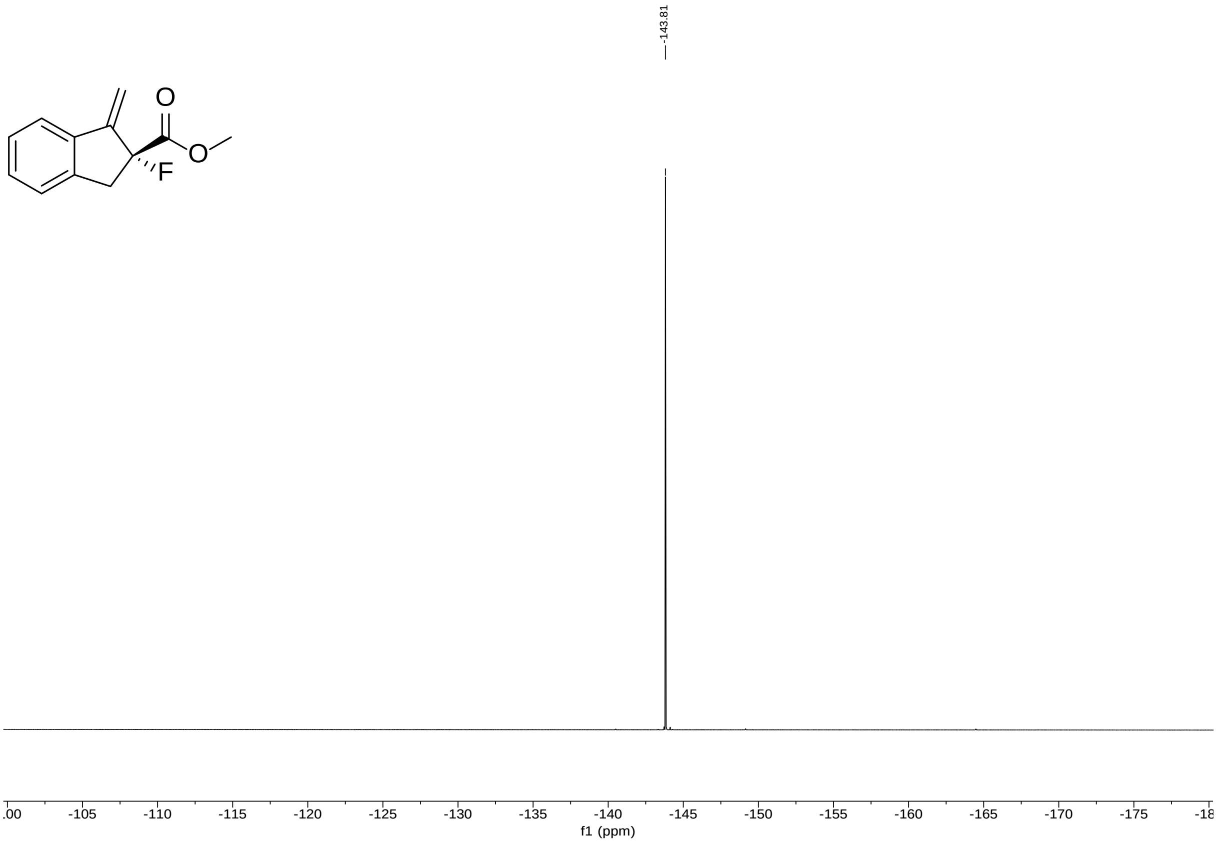

Ethyl (*R*)-5-bromo-2-fluoro 1-methylene-2,3-dihydro-1*H*-indene-2-carboxylate (S46)

^{1}H NMR (599 MHz, $CDCl_3$, 299 K)

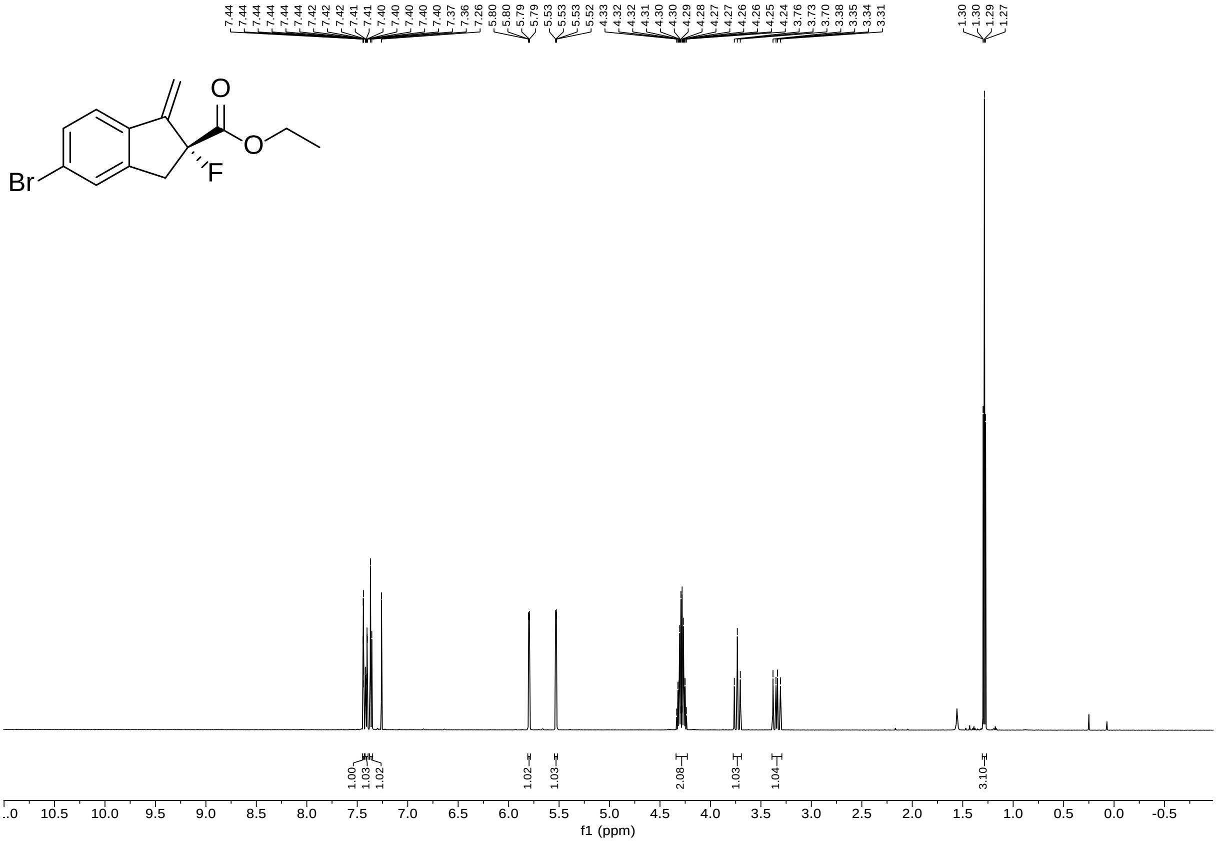

^{13}C NMR (151 MHz, $CDCl_3$, 299 K)

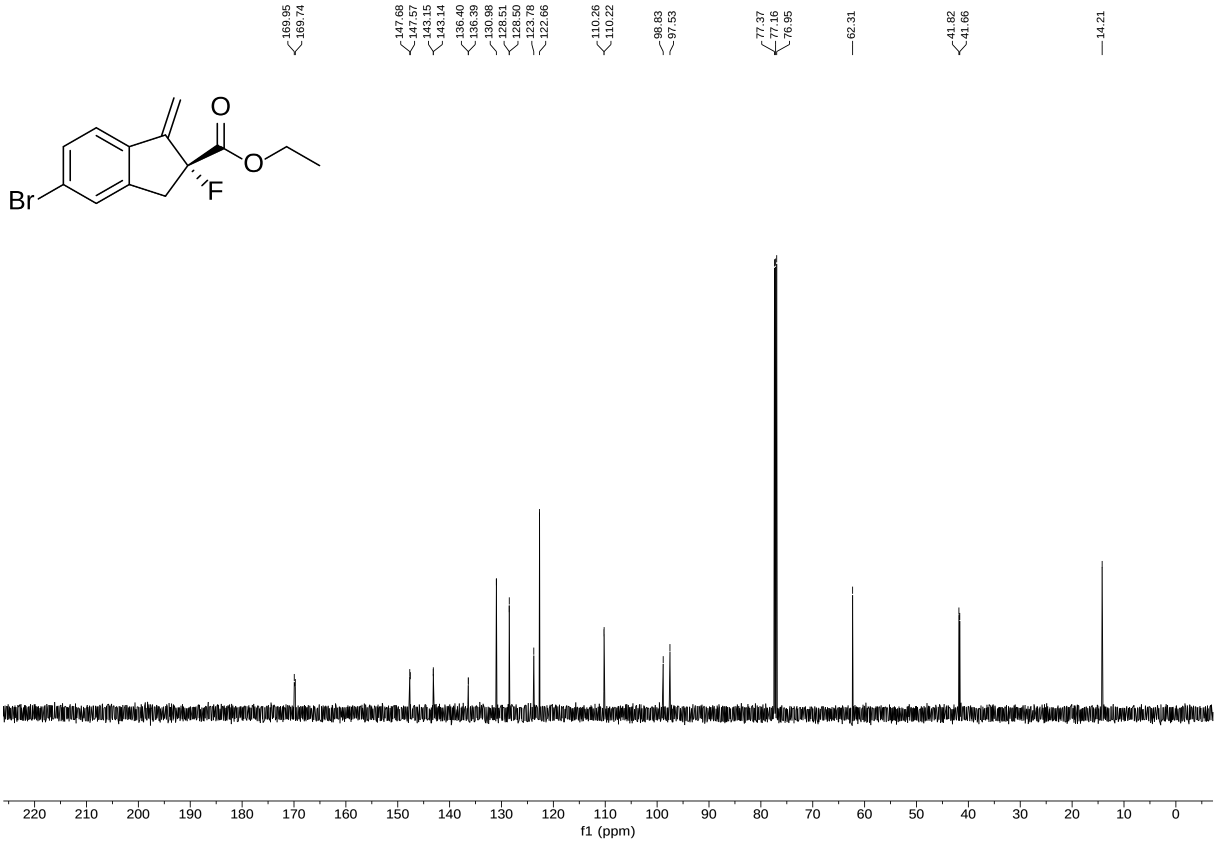

^{19}F NMR (564 MHz, $CDCl_3$, 299 K)

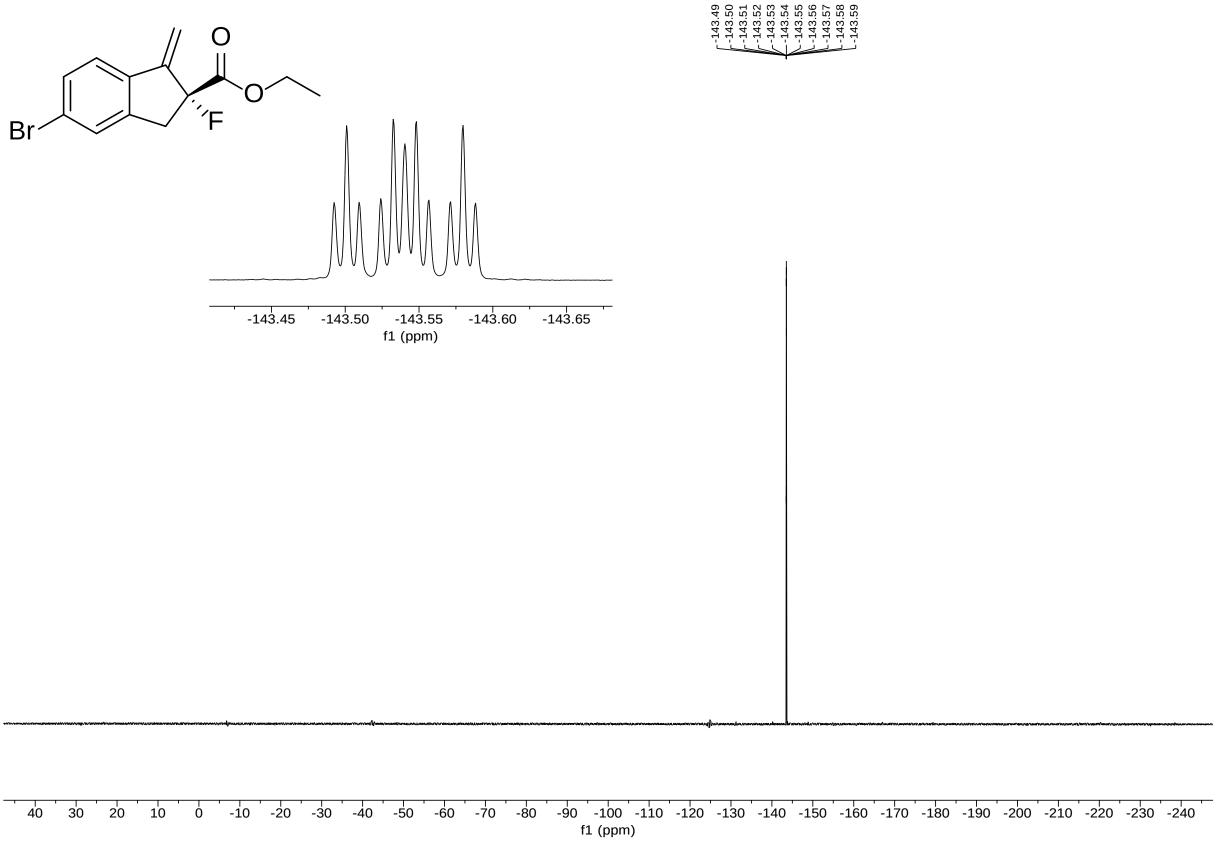

^{19}F{^{1}H} NMR (564 MHz, $CDCl_3$, 299 K

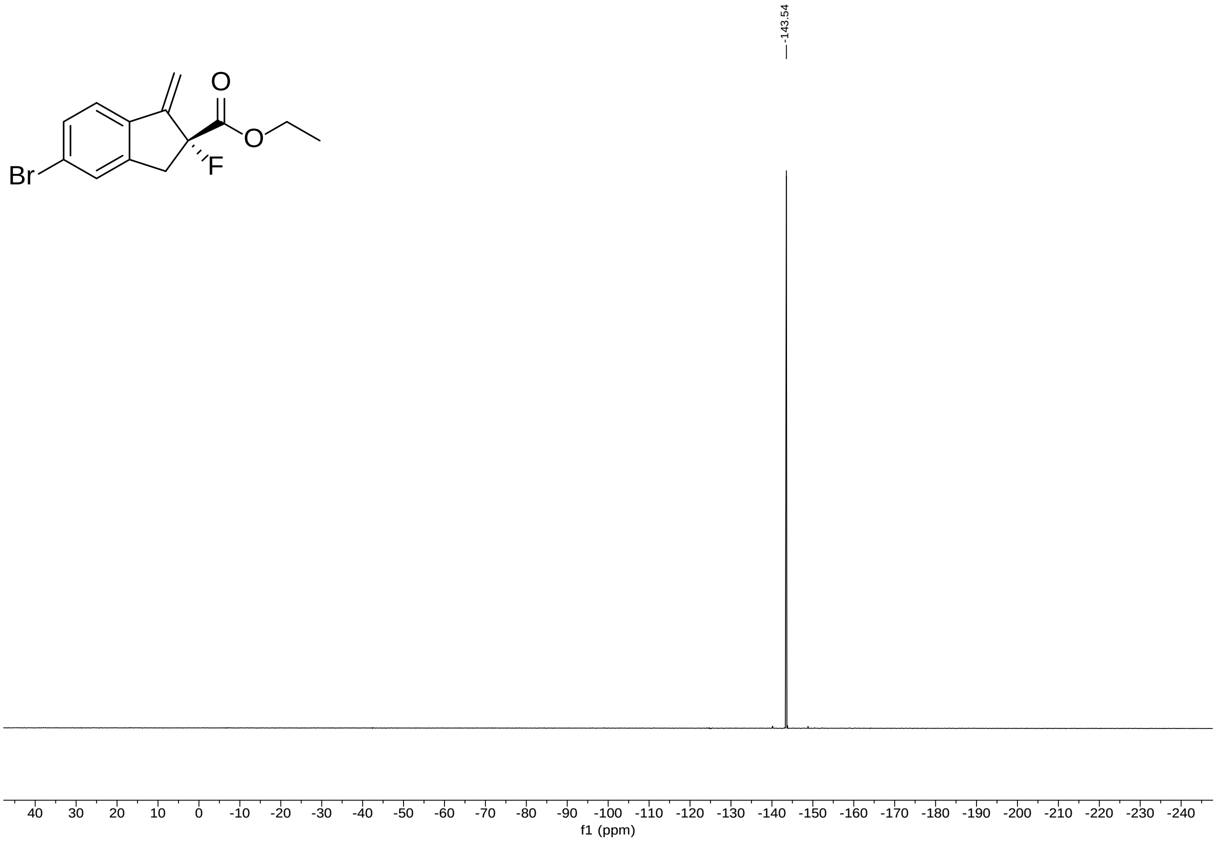

Ethyl (*R*)-5-chloro-2-fluoro 1-methylene-2,3-dihydro-1*H*-indene-2-carboxylate (S49)

^{1}H NMR (599 MHz, $CDCl_3$, 299 K)

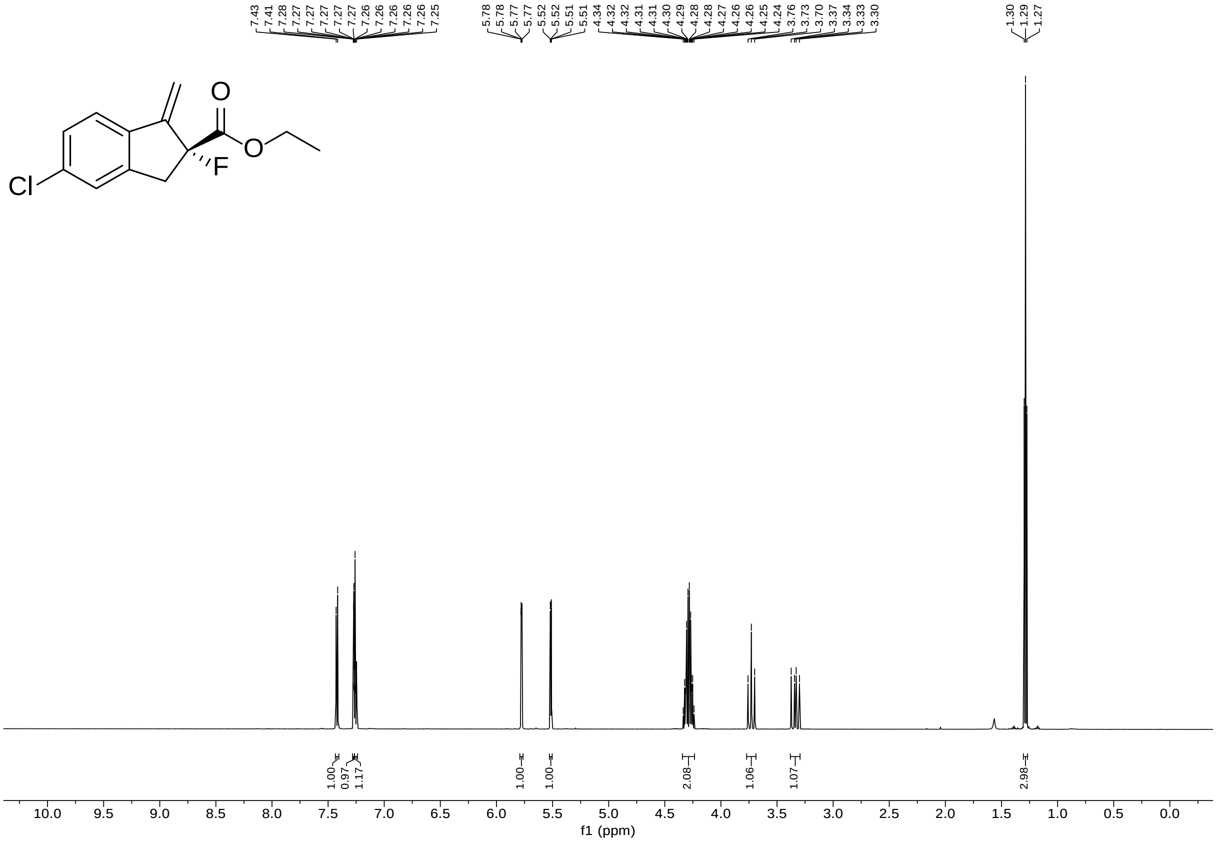

^{13}C NMR (151 MHz, $CDCl_3$, 299 K)

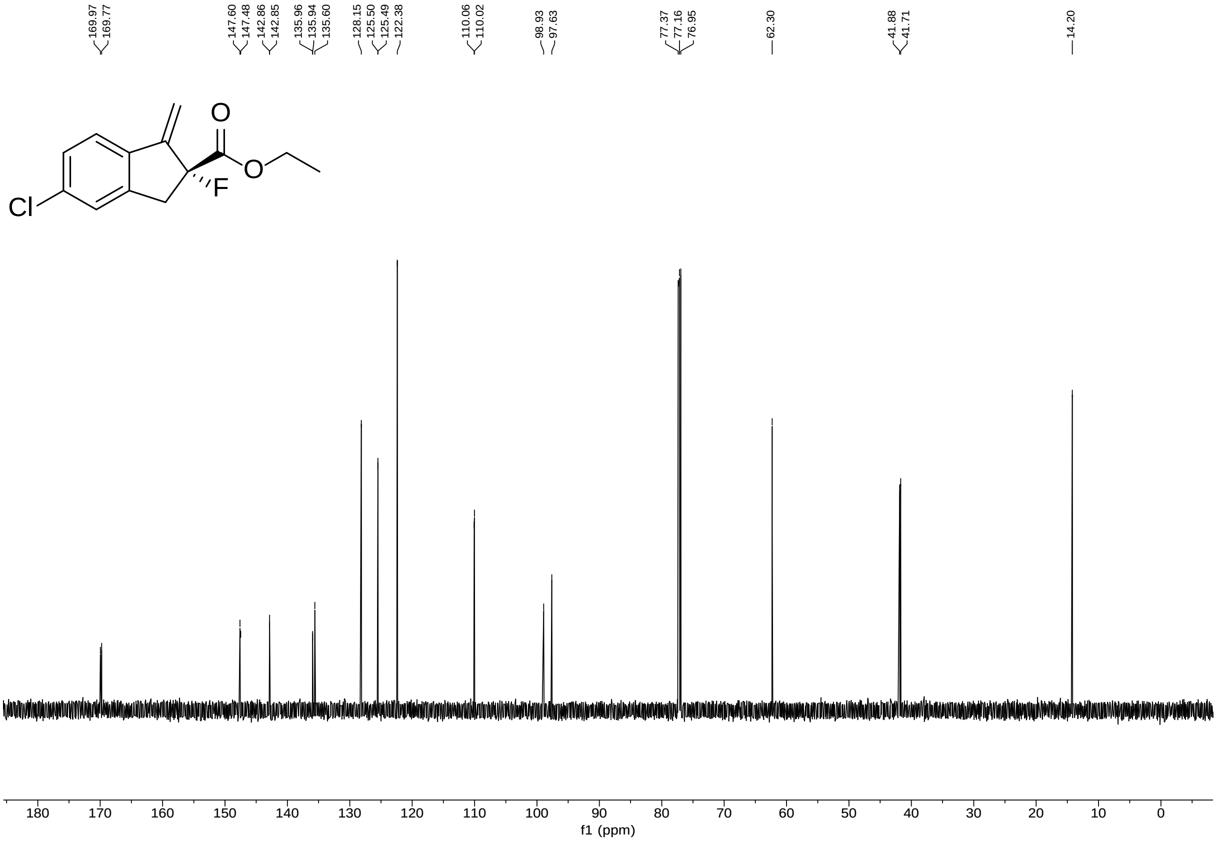

^{19}F NMR (564MHz, $CDCl_3$, 299 K)

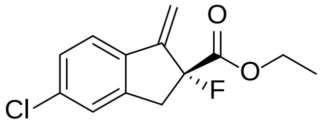

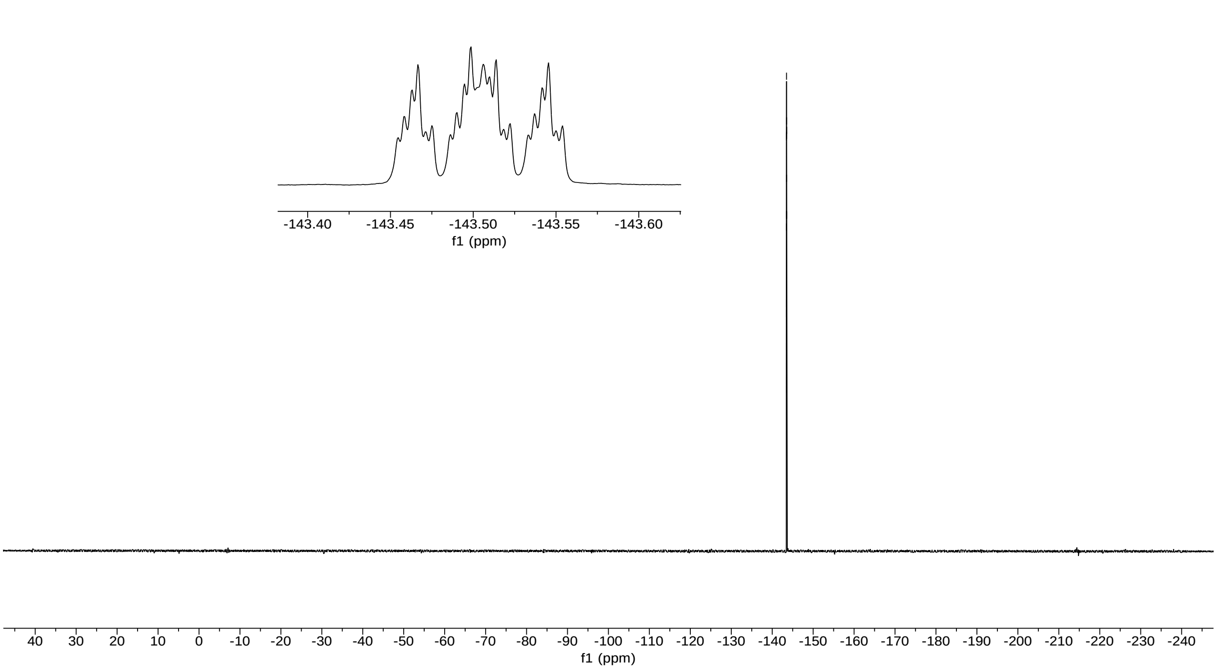

^{19}F{^{1}H} NMR (470 MHz, $CDCl_3$, 299 K)

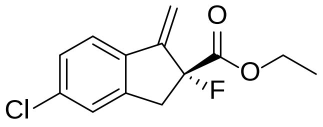

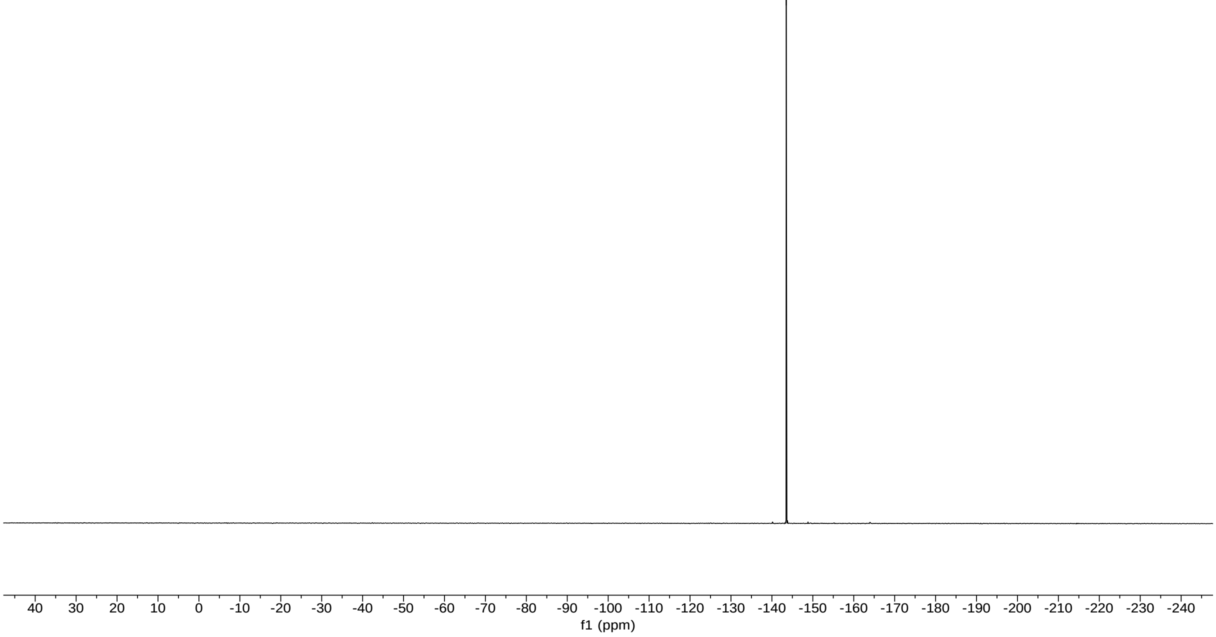

Ethyl (*R*)-2-fluoro 1-methylene-6-(trifluoromethyl)-2,3-dihydro-1*H*-indene-2-carboxylate (S52)

^{1}H NMR (599 MHz, $CDCl_3$, 299 K)

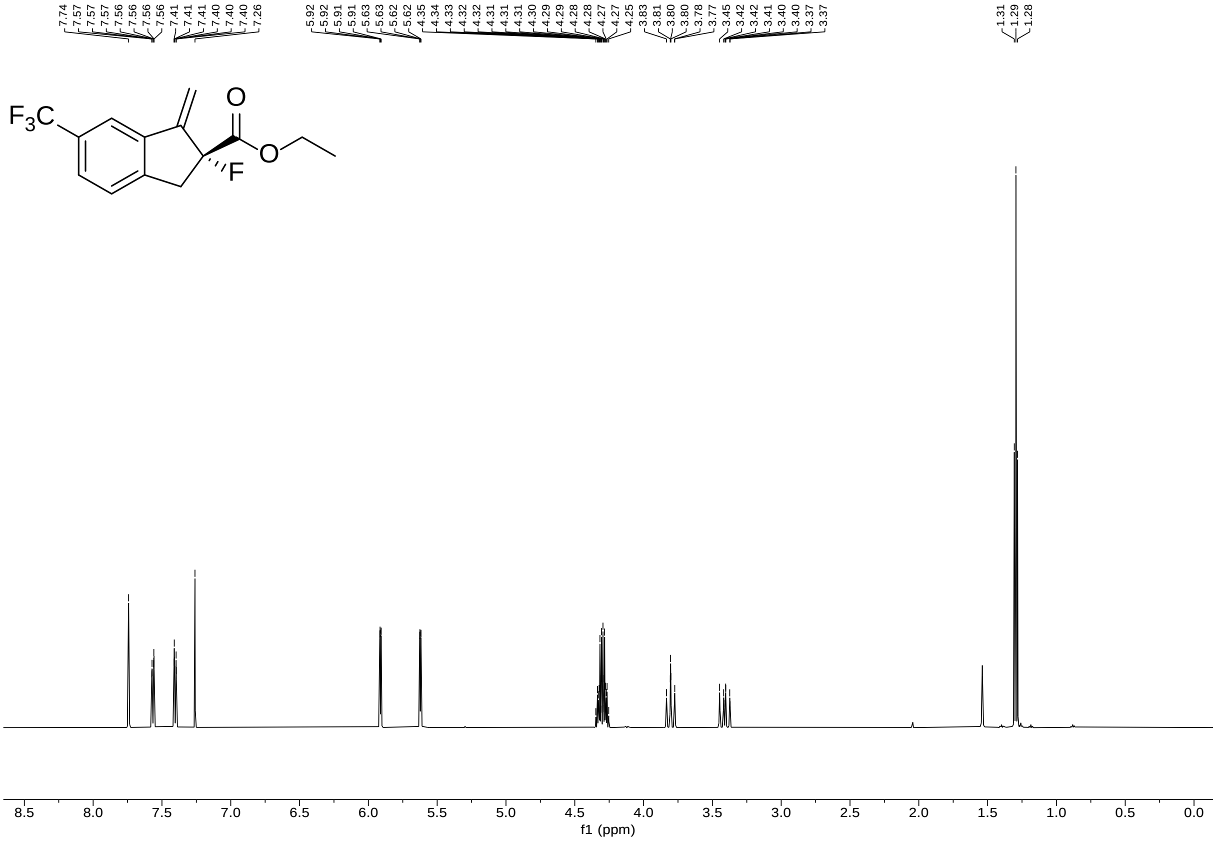

^{13}C NMR (151 MHz, $CDCl_3$, 299 K)

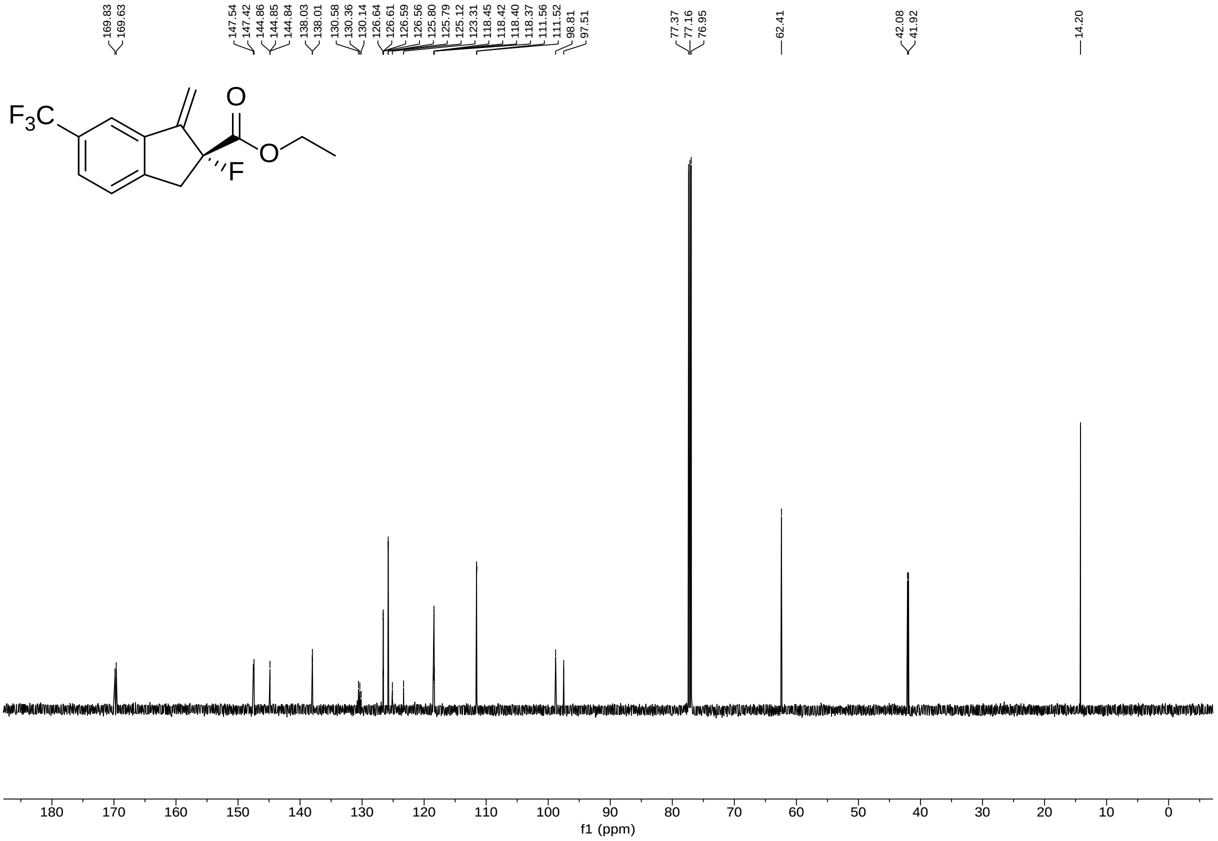

^{19}F NMR (564 MHz, $CDCl_3$, 299 K)

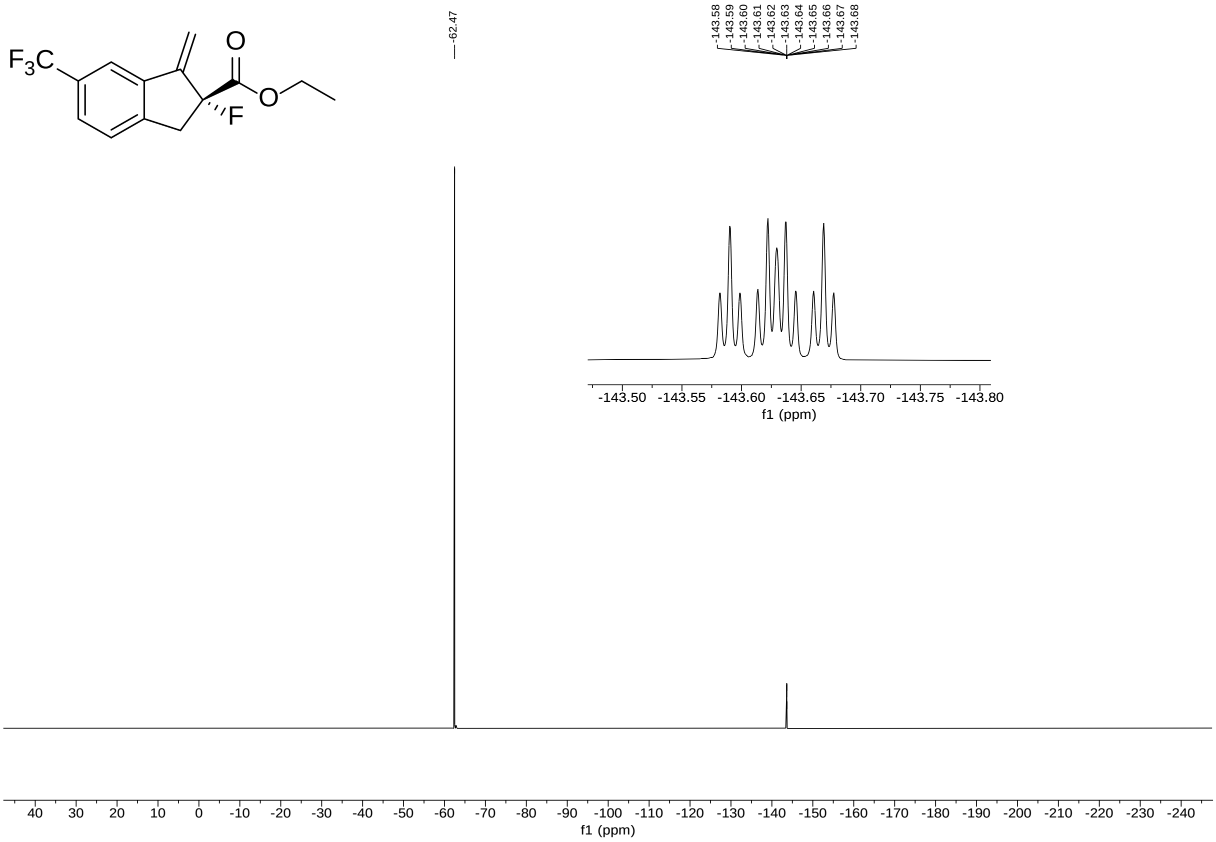

^{19}F{^{1}H} NMR (470 MHz, $CDCl_3$, 299 K)

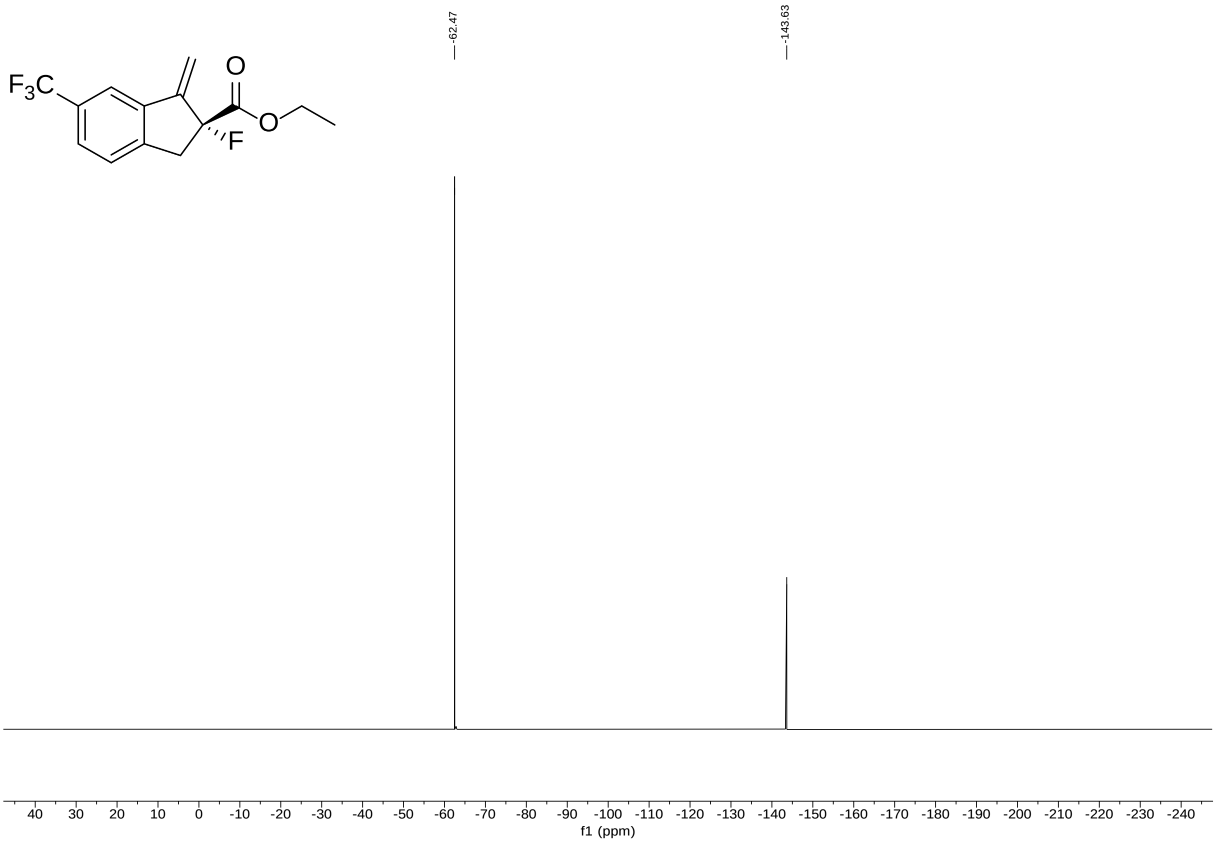

2-Fluoro-6-methoxy-1-methylene-2,3-dihydro-1*H*-indene (S54)

^{1}H NMR (500 MHz, $CDCl_3$, 299 K)

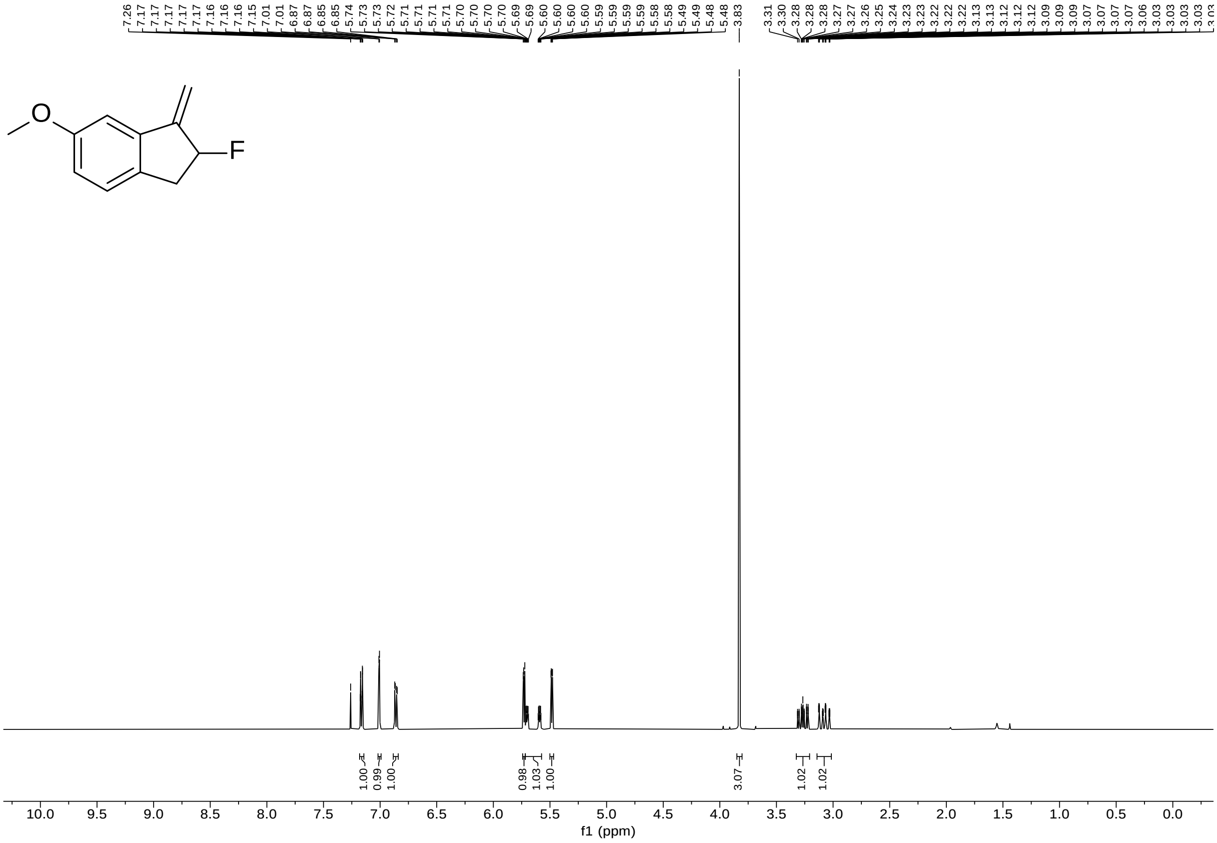

^{13}C NMR (126 MHz, $CDCl_3$, 299 K)

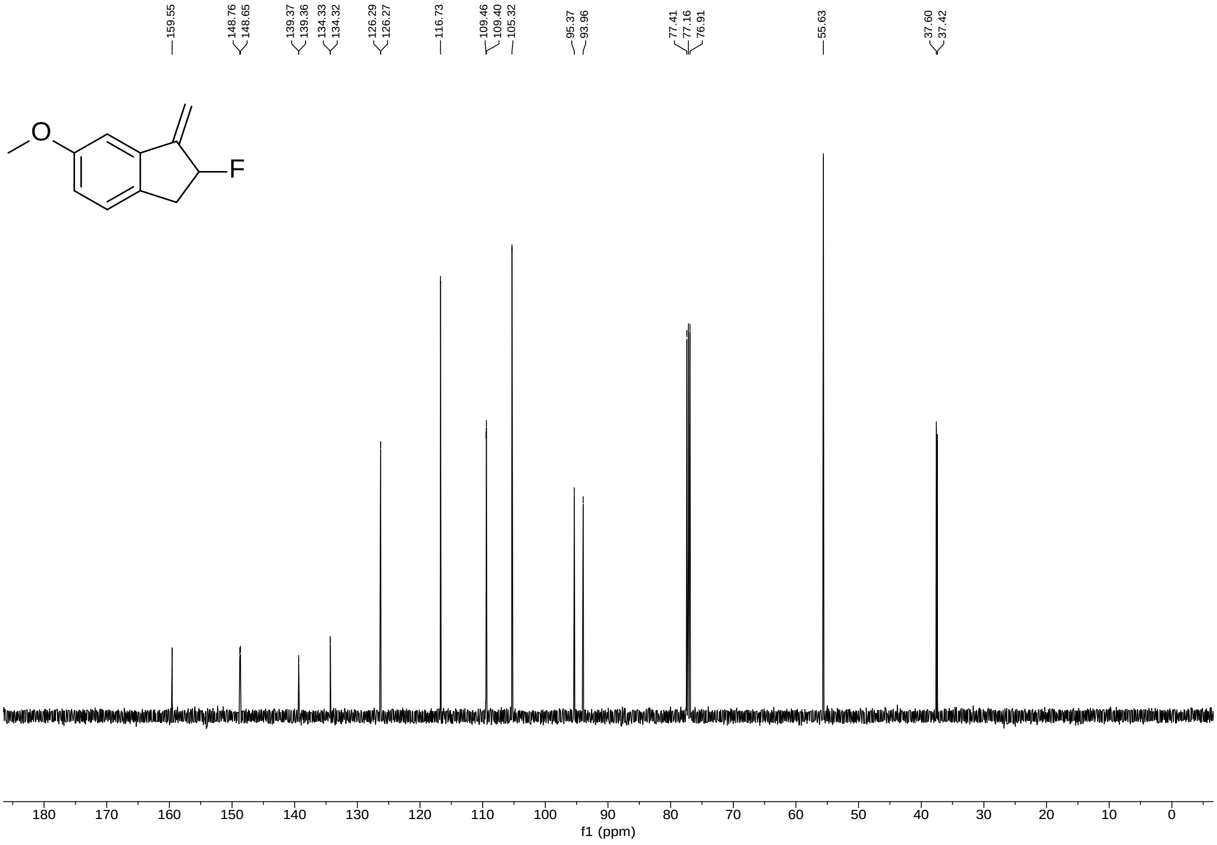

^{19}F NMR (470 MHz, $CDCl_3$, 299 K)

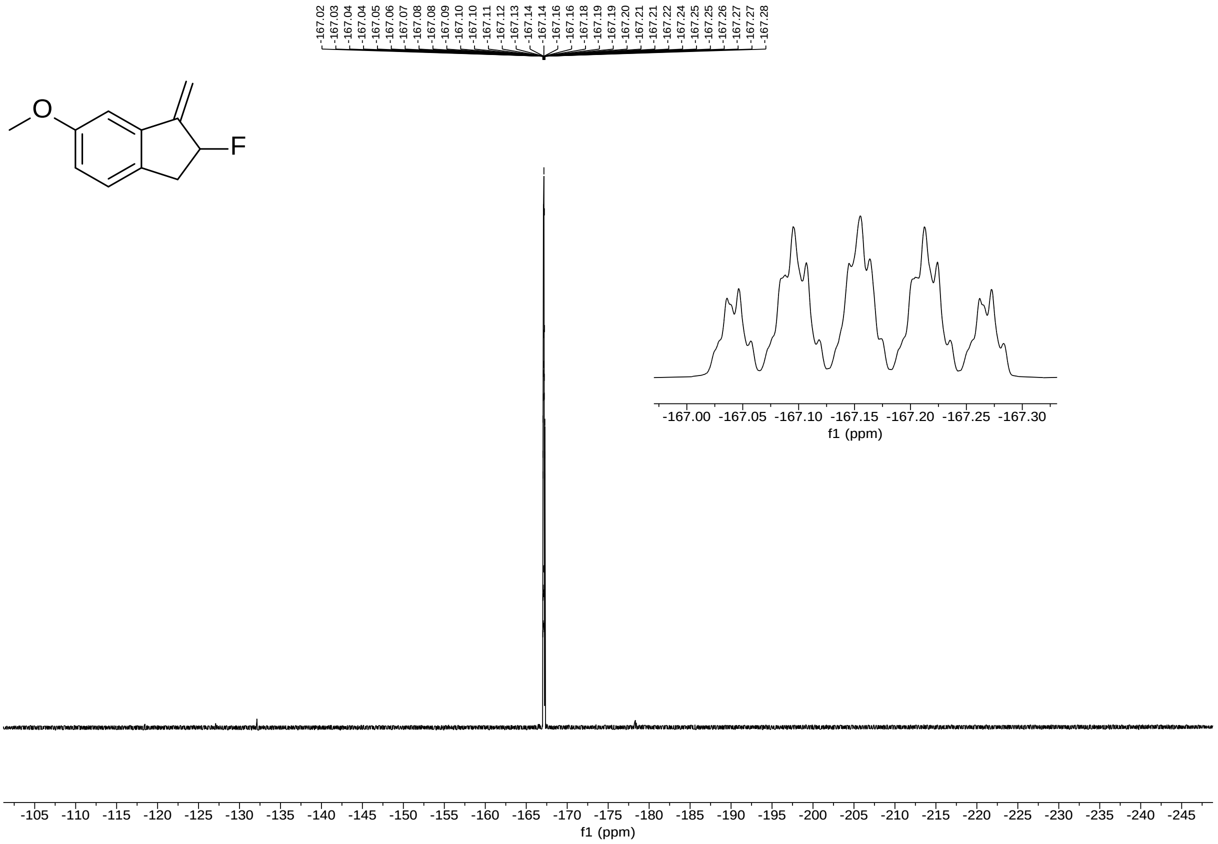

^{19}F{^{1}H} NMR (470 MHz, $CDCl_3$, 299 K)

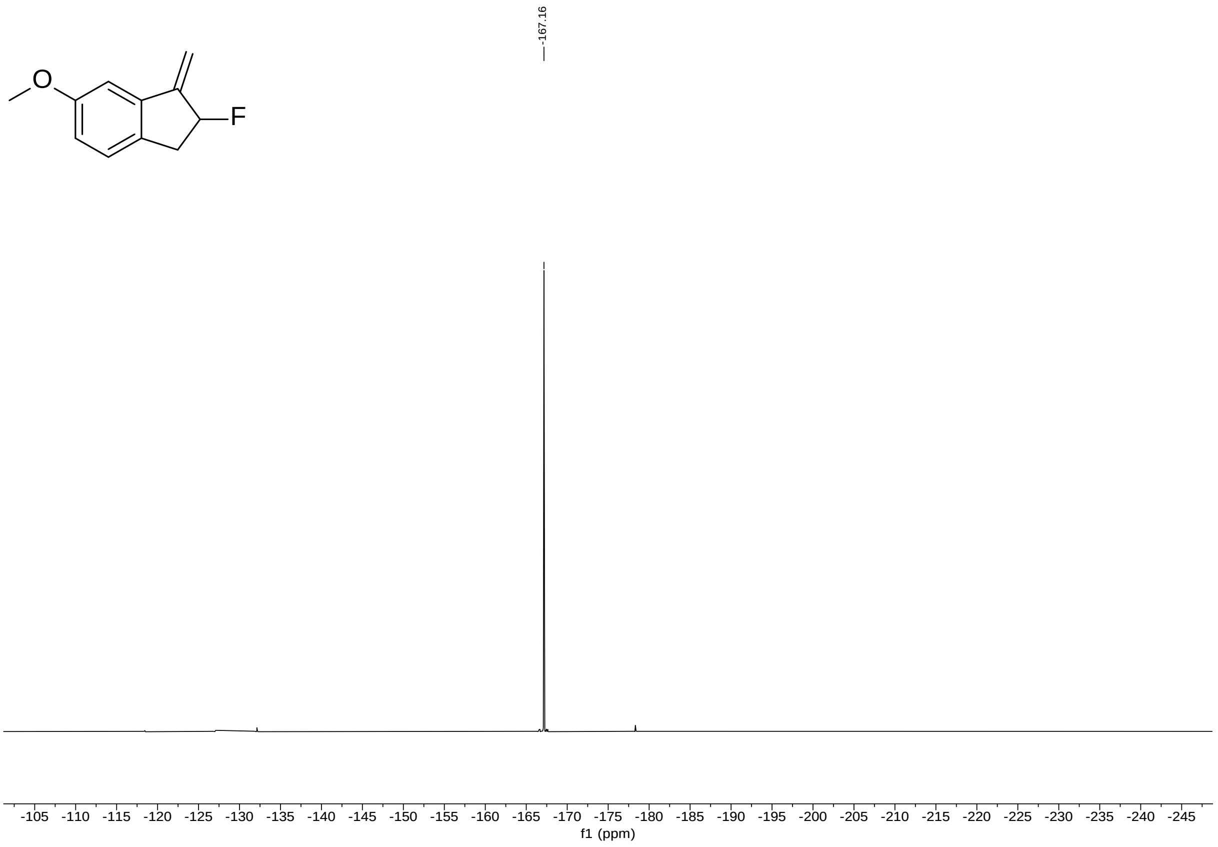

IX. NMR Spectra of Trifluorinated Tetralins

2,2,3-Trifluoro-1,2,3,4-tetrahydronaphthalene (9)

^{1}H NMR (599 MHz, $CDCl_3$, 299 K)

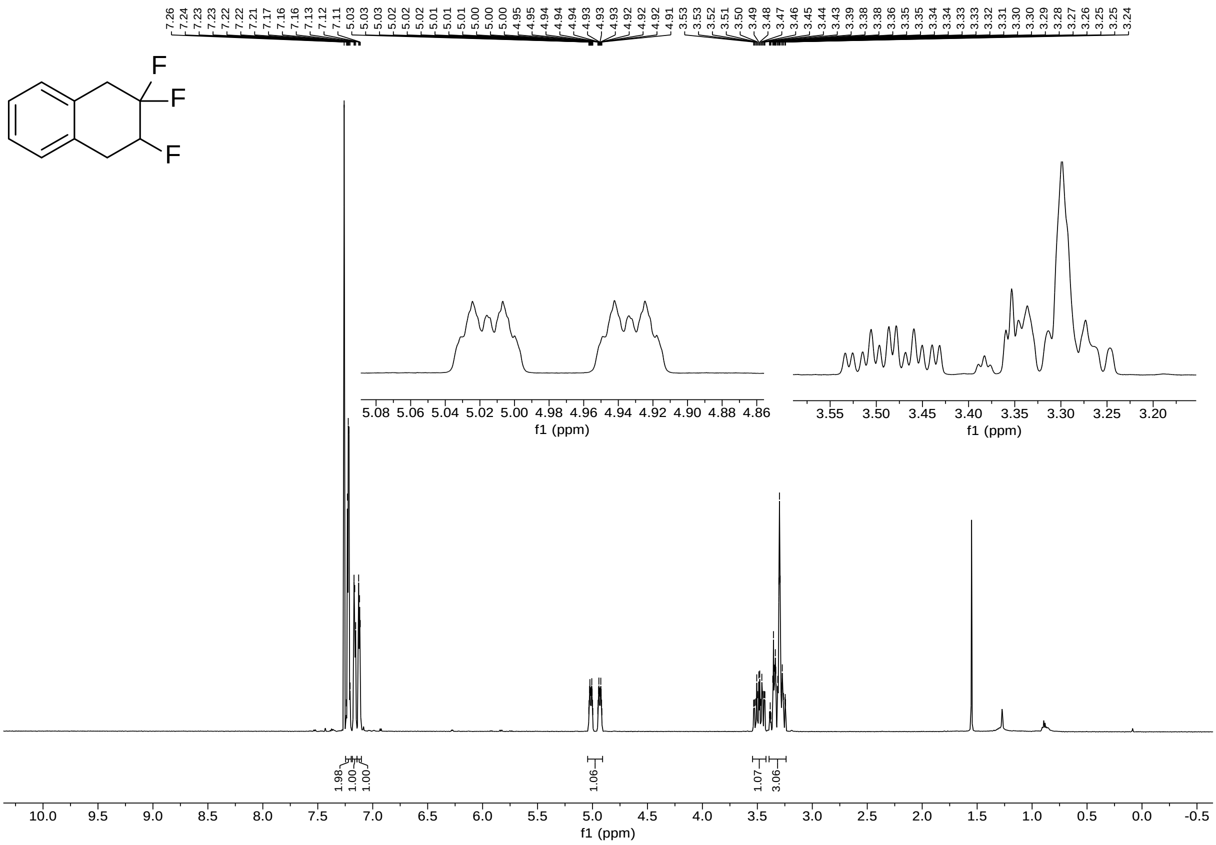

^{13}C NMR (151 MHz, $CDCl_3$, 299 K)

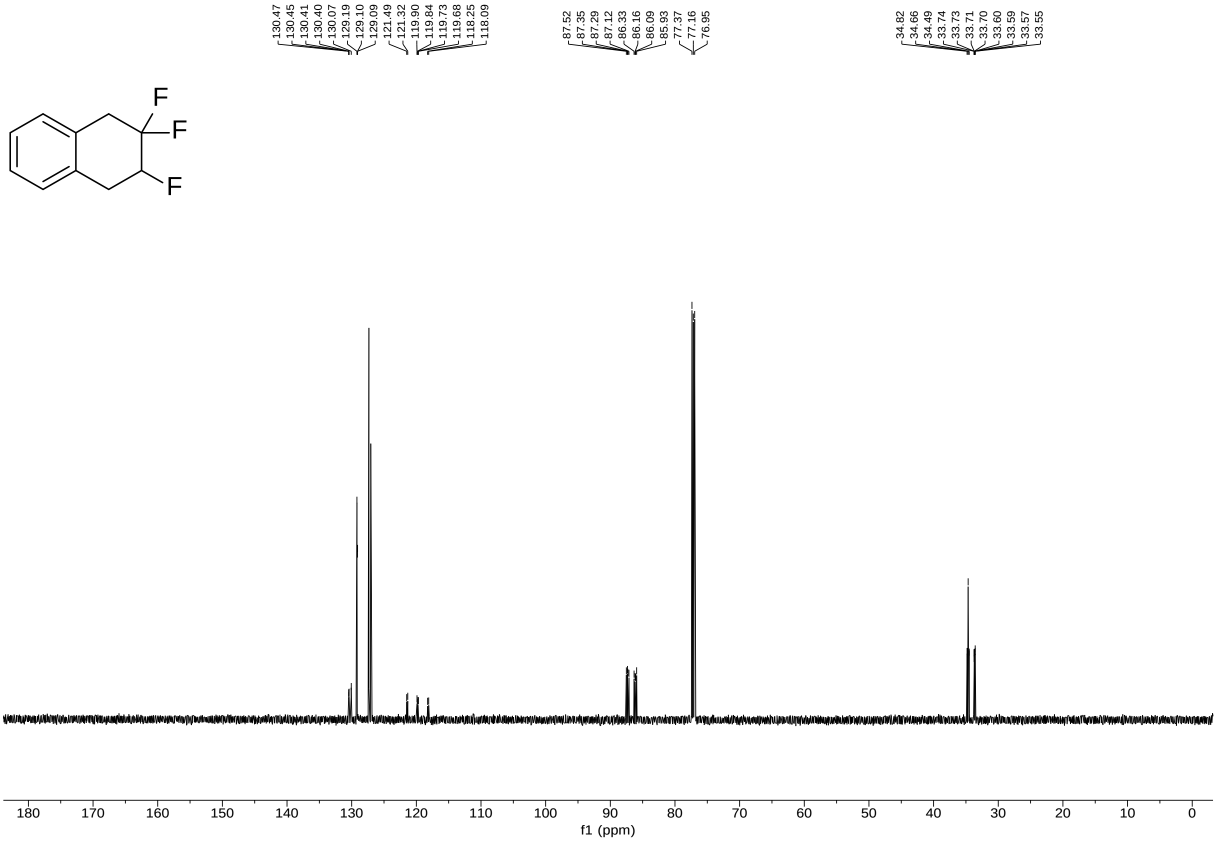

^{19}F NMR (564 MHz, $CDCl_3$, 299 K)

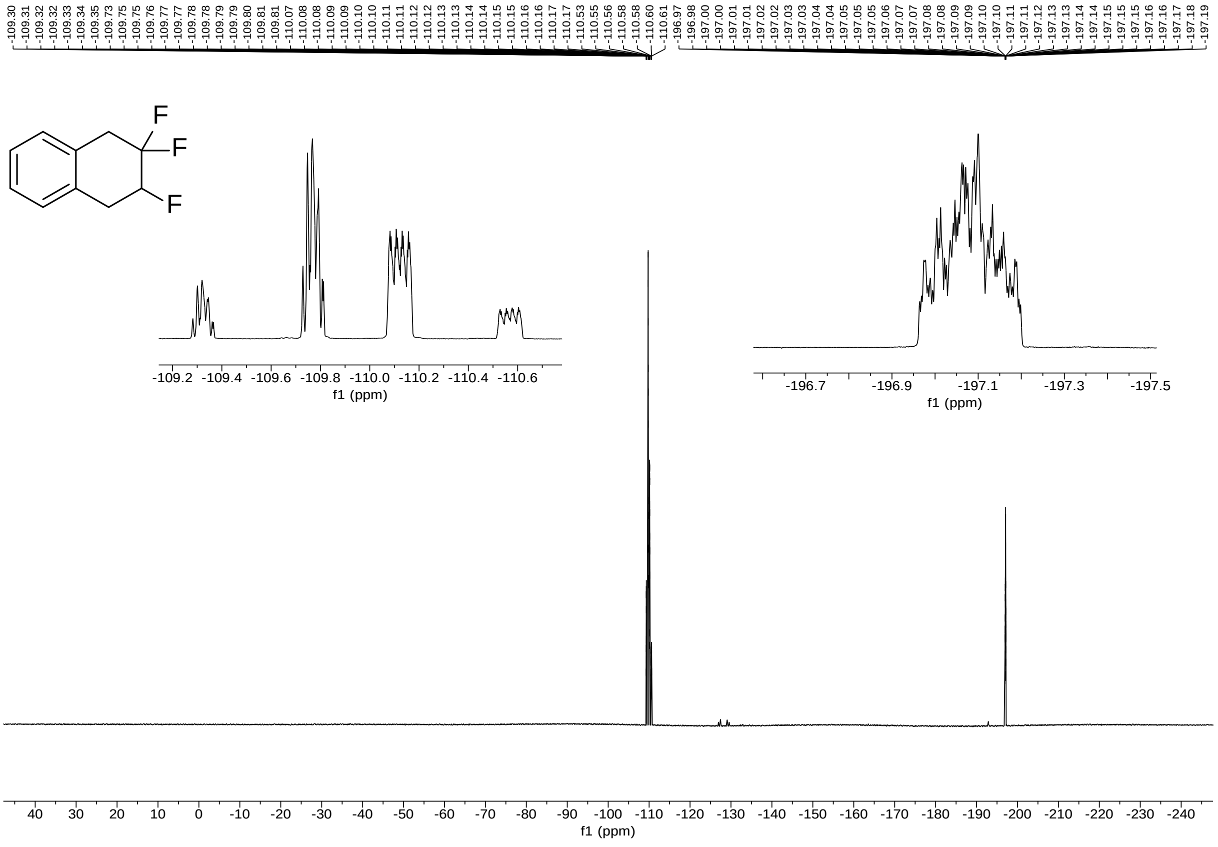

$^{19}F\{^1H\}$ NMR (564 MHz, $CDCl_3$, 299 K)

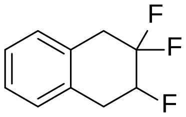

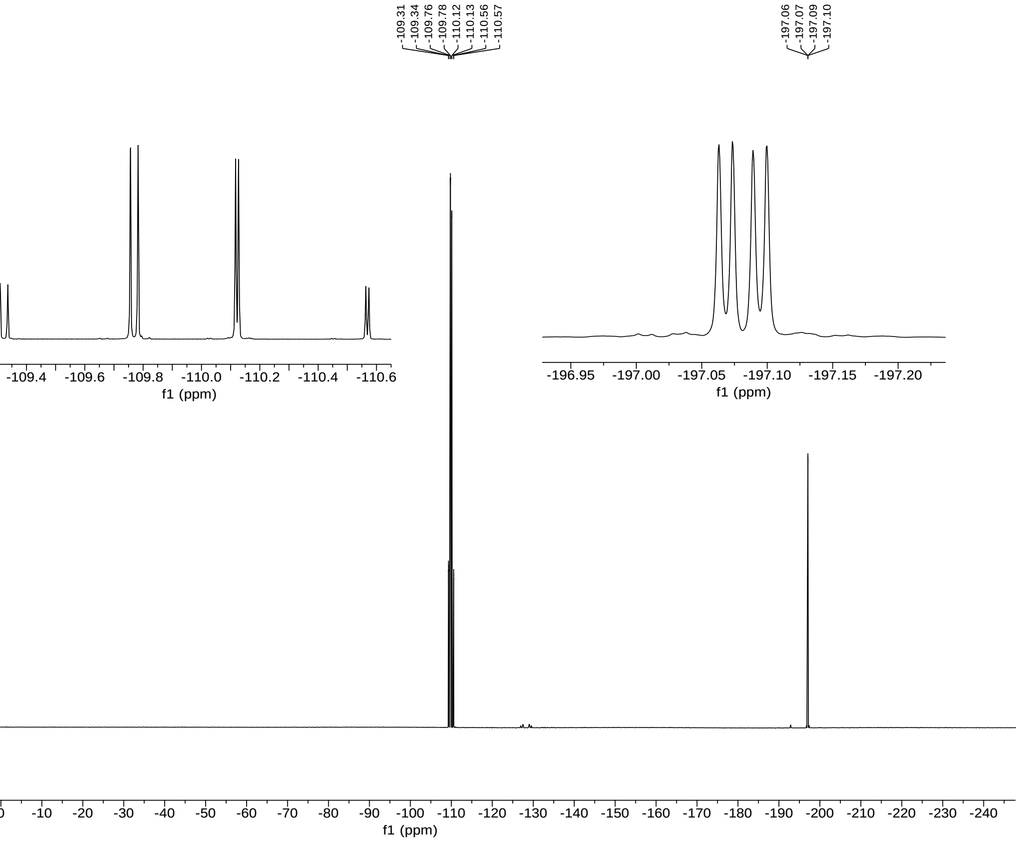

2,2,3,6-Tetrafluoro-1,2,3,4-tetrahydronaphthalene (10)

^{1}H NMR (599 MHz, $CDCl_3$, 299 K)

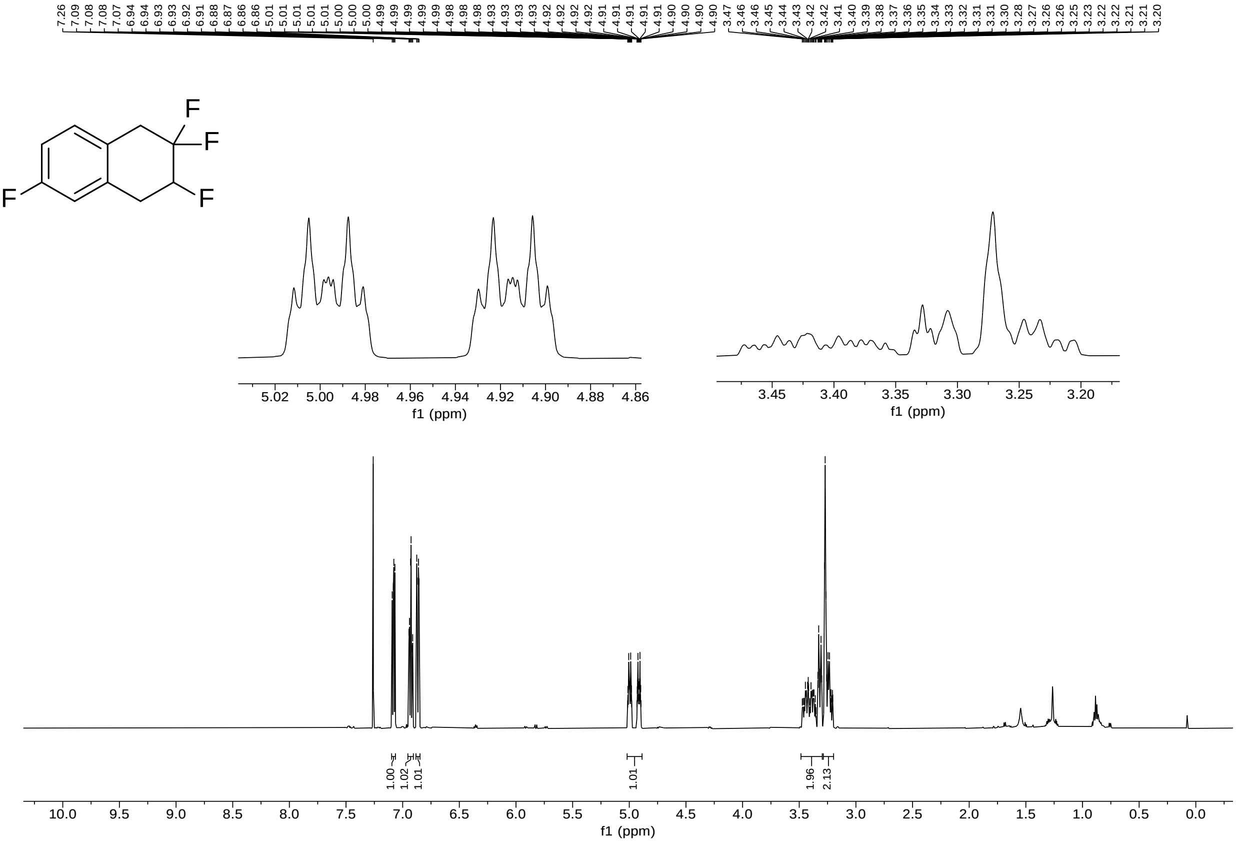

^{13}C NMR (151 MHz, $CDCl_3$, 299 K)

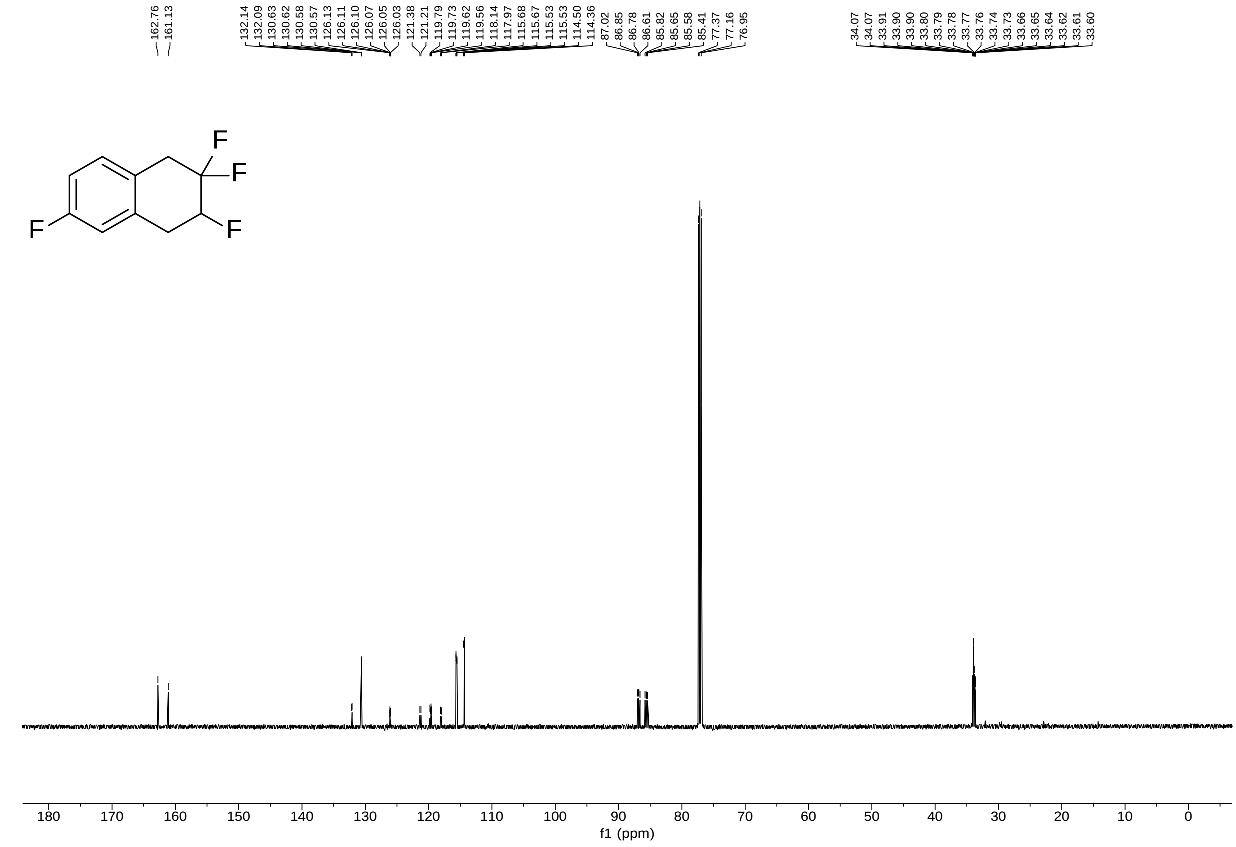

^{19}F NMR (565 MHz, $CDCl_3$, 299 K)

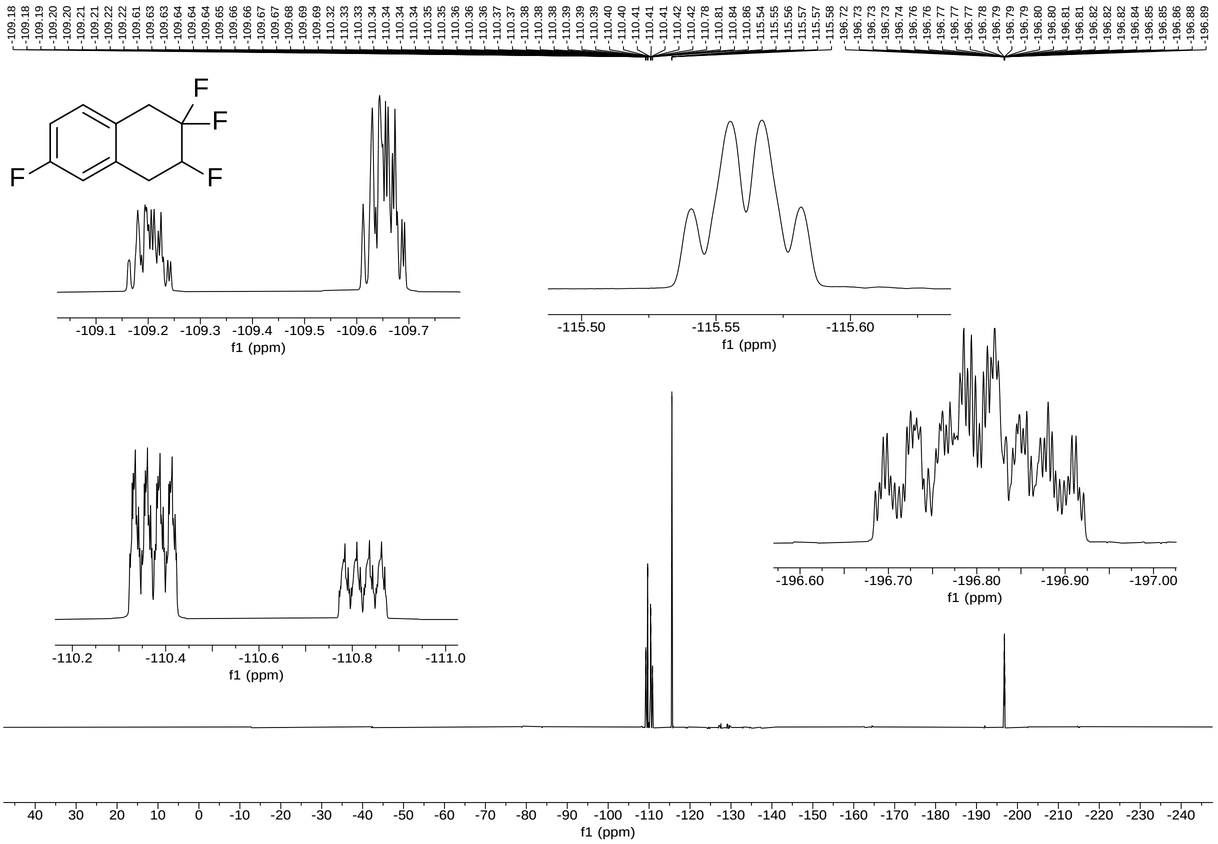

^{19}F{^{1}H} NMR (564 MHz, $CDCl_3$, 299 K)

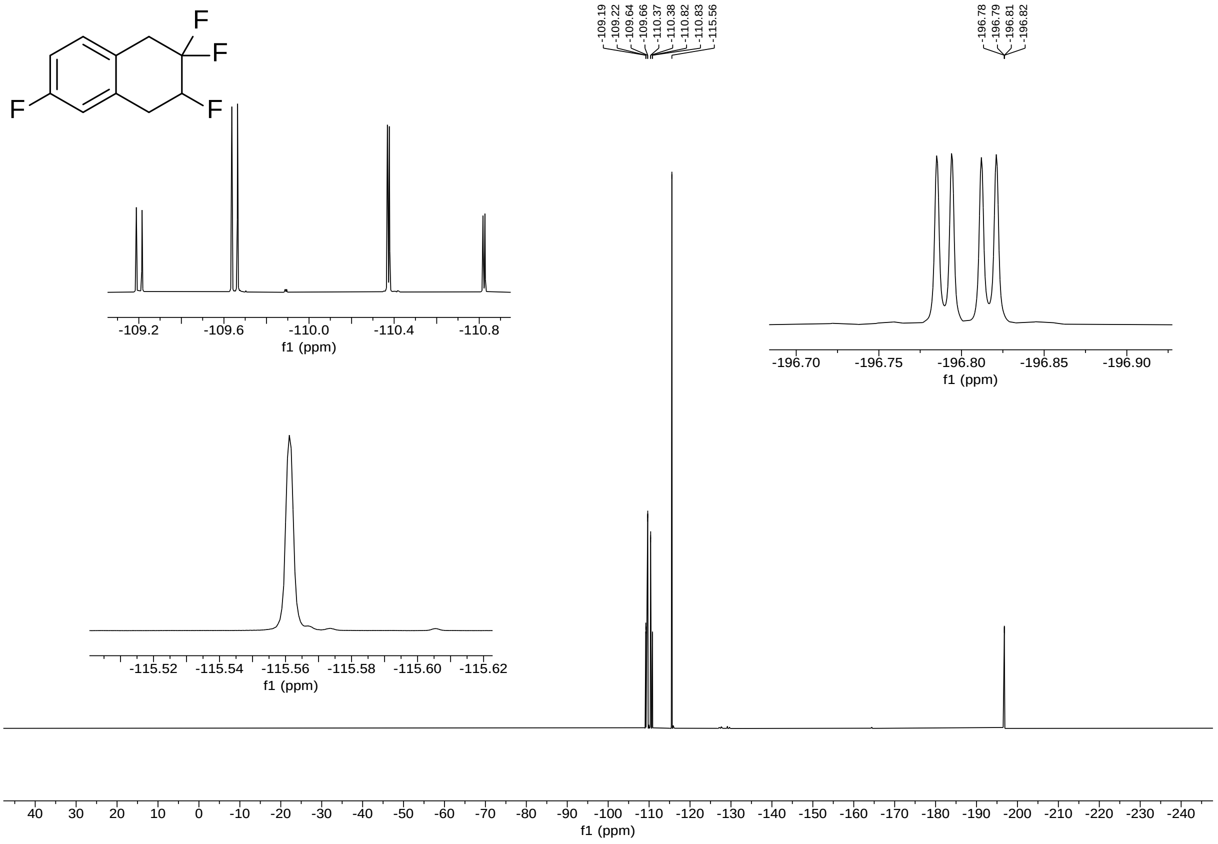

6-Chloro-2,2,3-trifluoro-1,2,3,4-tetrahydronaphthalene (11)

^{1}H NMR (500 MHz, $CDCl_3$, 299 K)

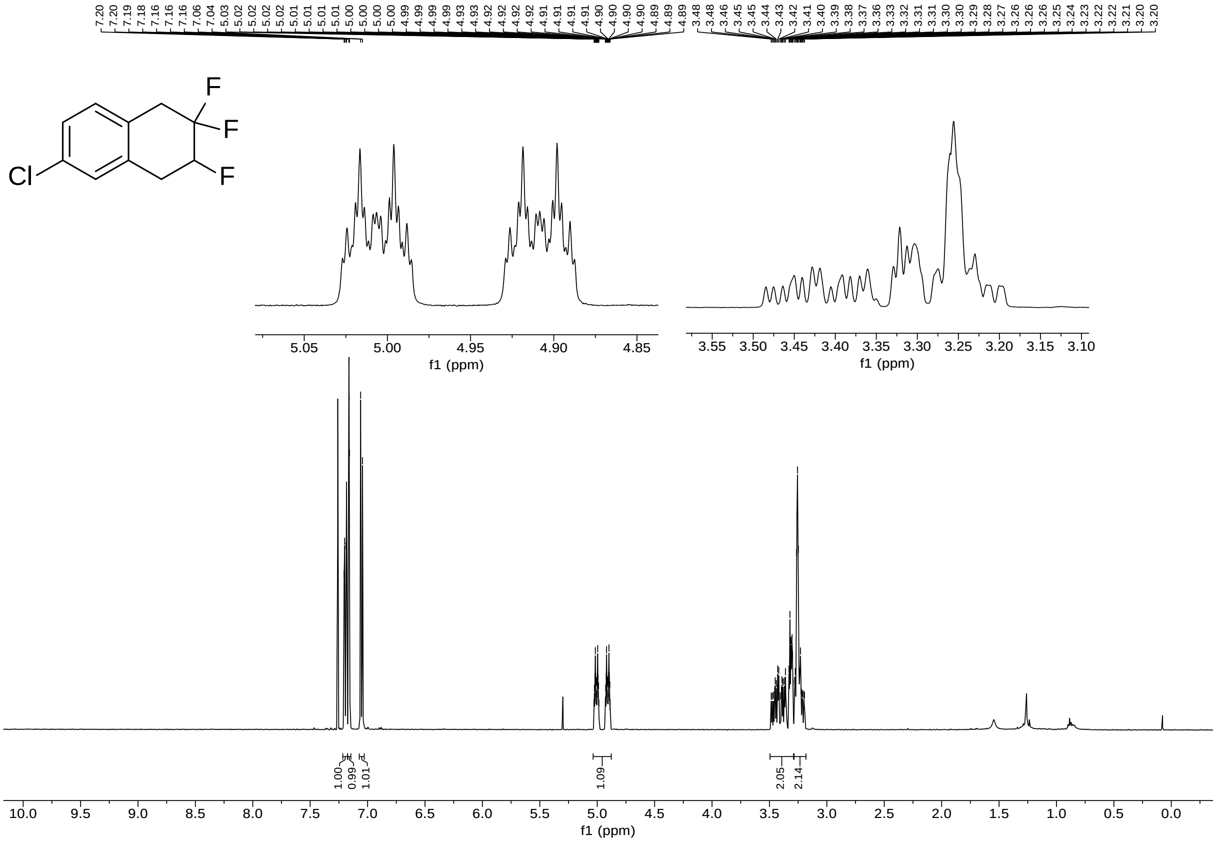

^{13}C NMR (126 MHz, $CDCl_3$, 299 K)

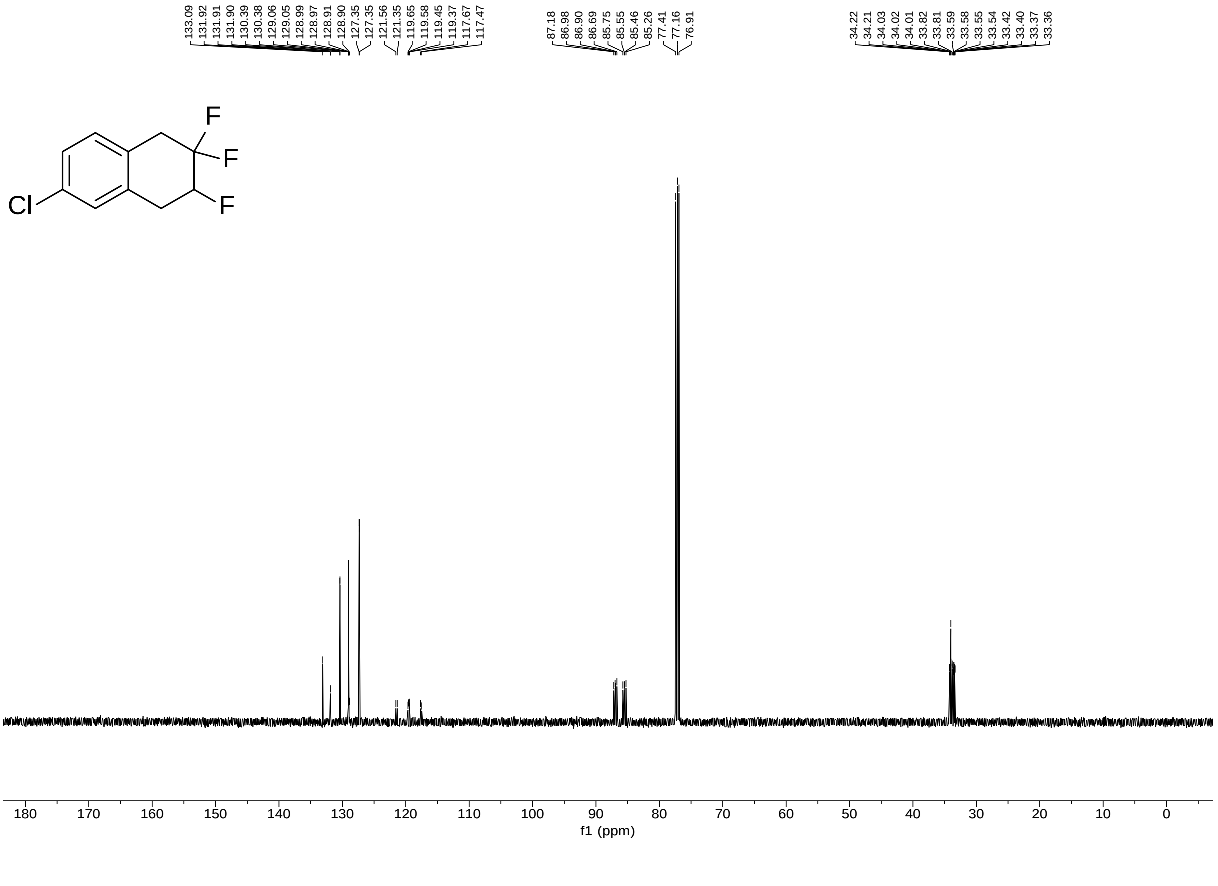

^{19}F NMR (470 MHz, $CDCl_3$, 299 K)

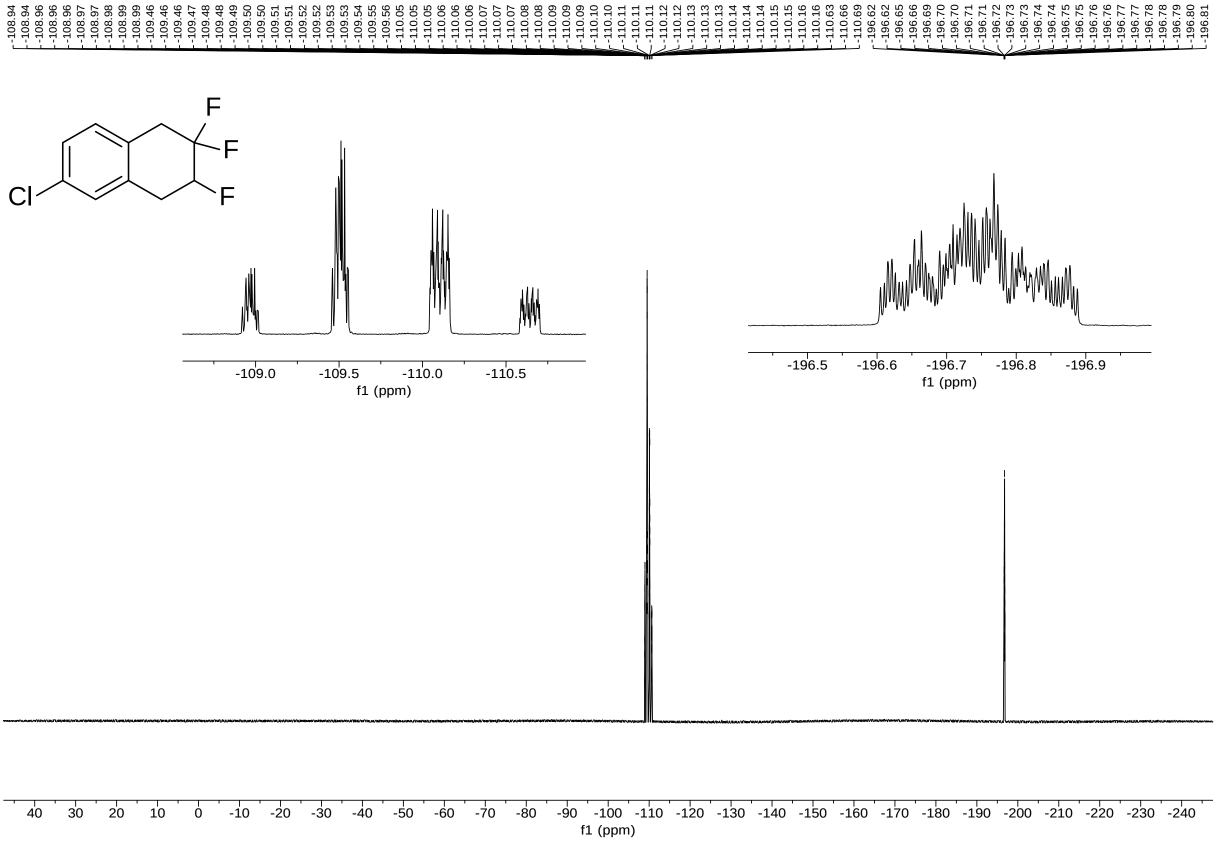

^{19}F{^{1}H} NMR (470 MHz, $CDCl_3$, 299 K)

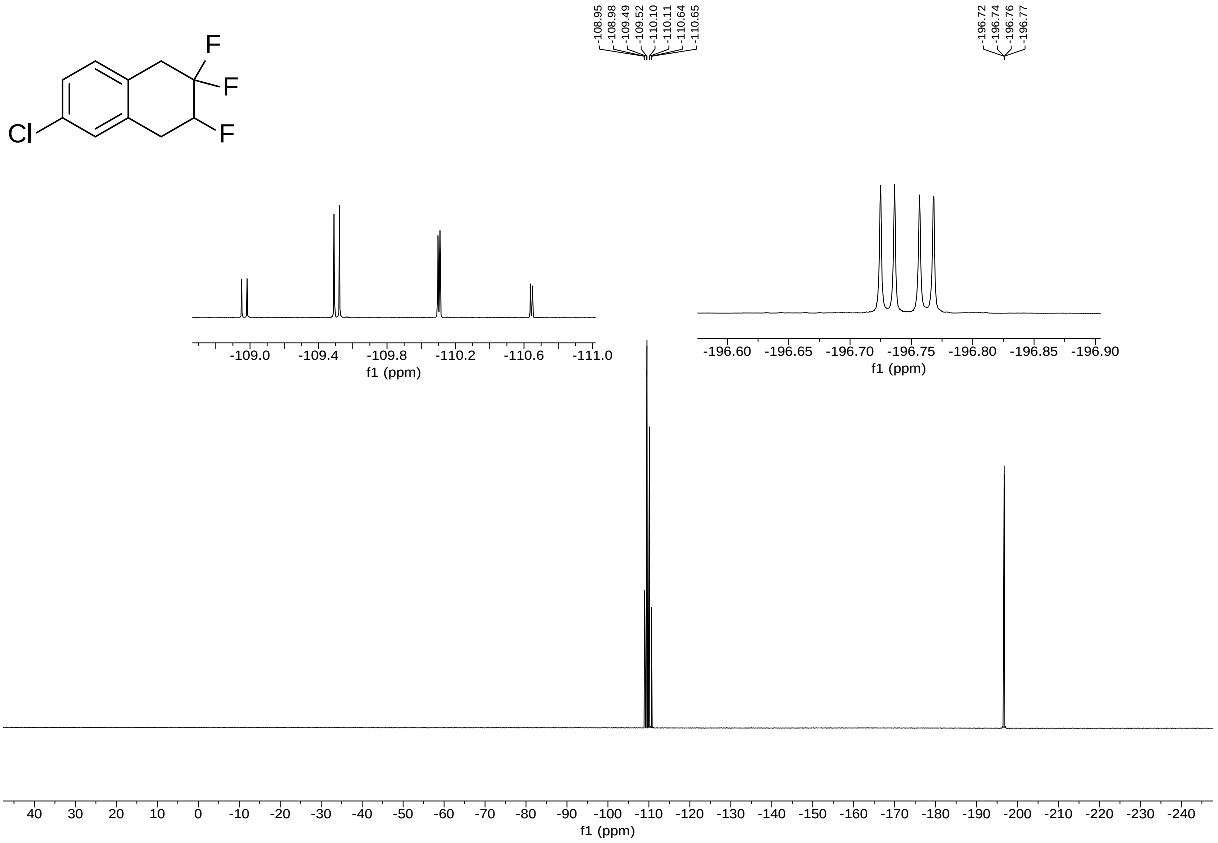

6-Bromo-2,2,3-trifluoro-1,2,3,4-tetrahydronaphthalene (12)

^{1}H NMR (500 MHz, $CDCl_3$, 299 K)

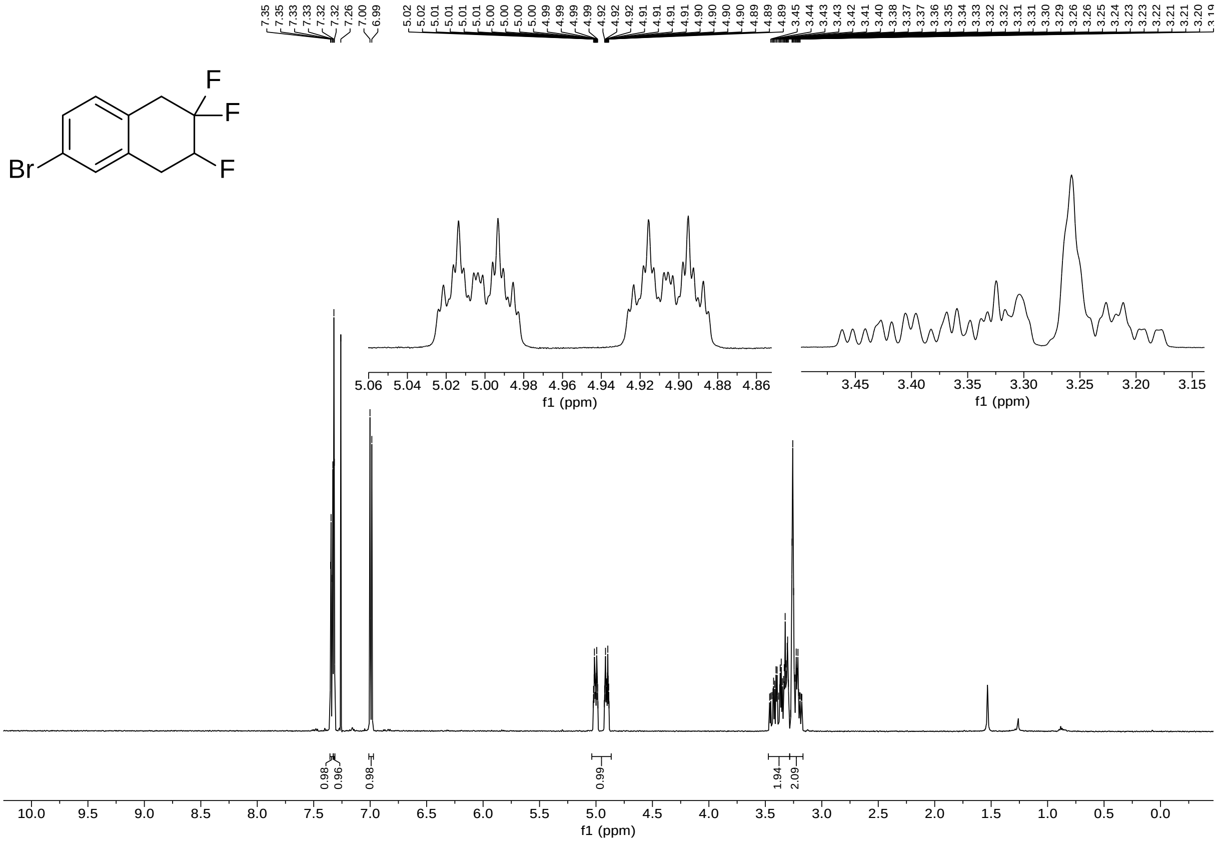

^{13}C NMR (126 MHz, $CDCl_3$, 299 K)

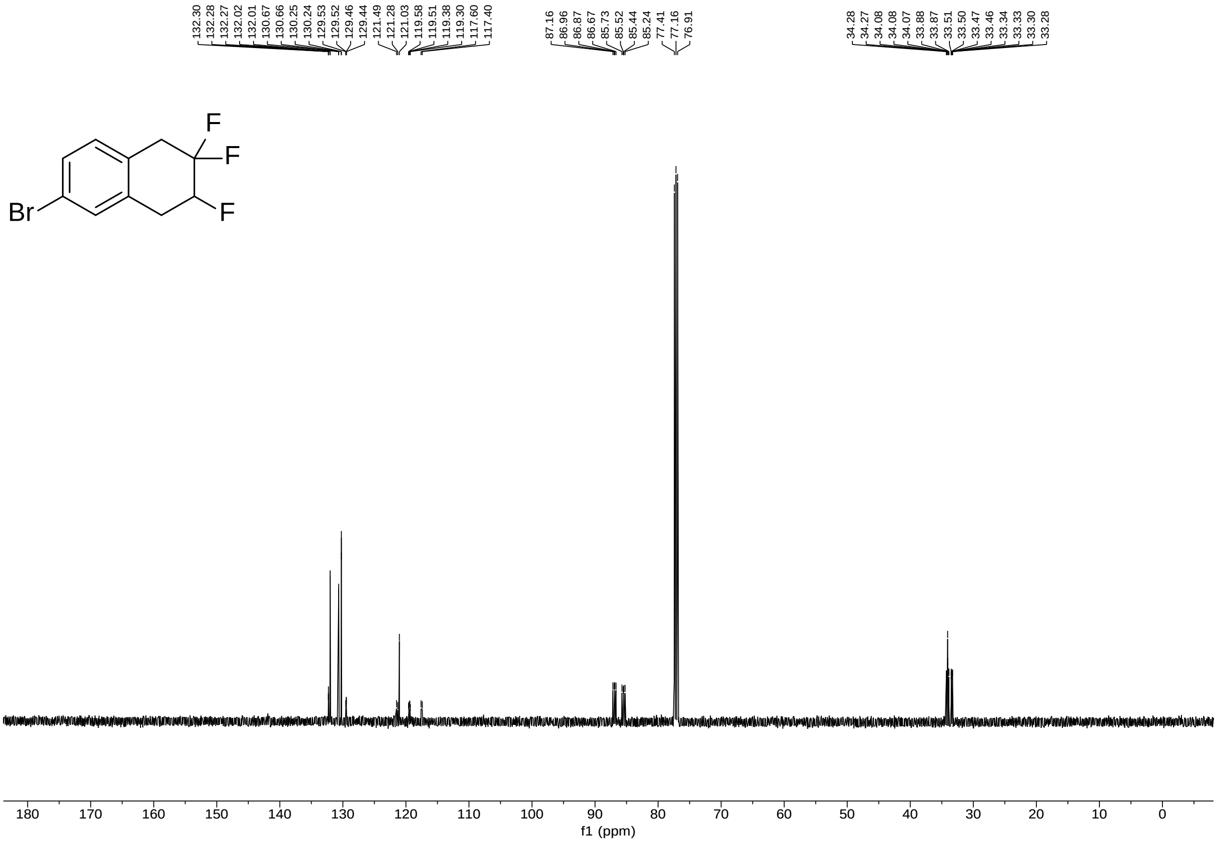

^{19}F NMR (470 MHz, $CDCl_3$, 299 K)

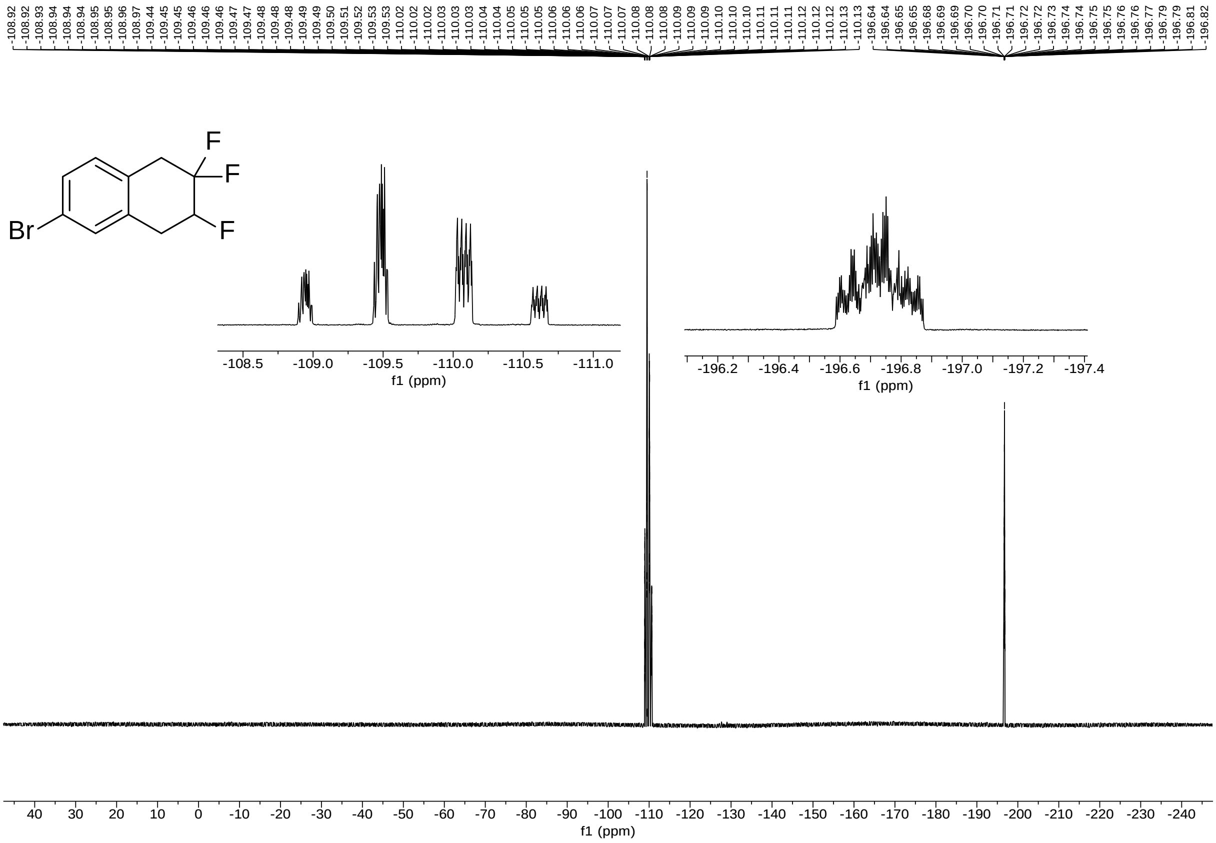

^{19}F{^{1}H} NMR (470 MHz, $CDCl_3$, 299 K)

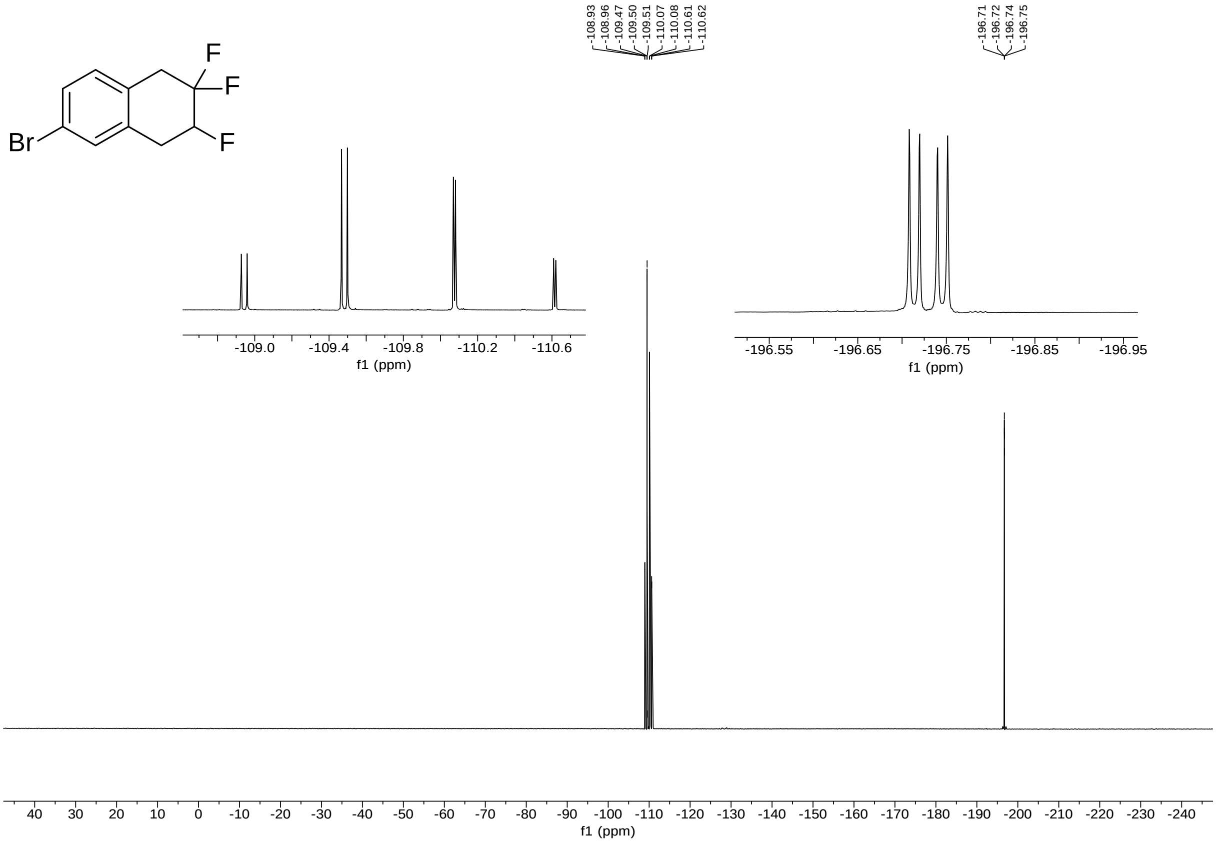

7-Bromo-2,2,3-trifluoro-1,2,3,4-tetrahydronaphthalene (13)

^{1}H NMR (599 MHz, $CDCl_3$, 299 K)

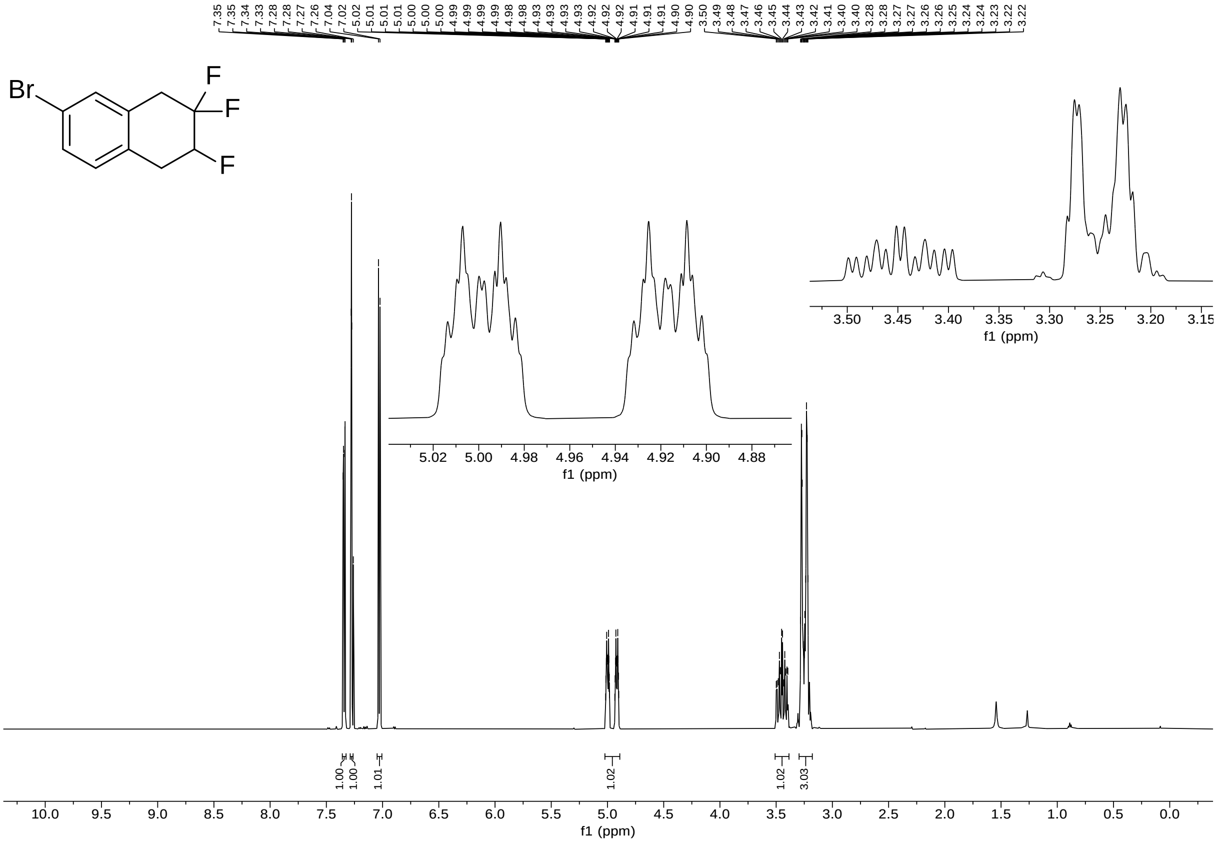

^{13}C NMR (151 MHz, $CDCl_3$, 299 K)

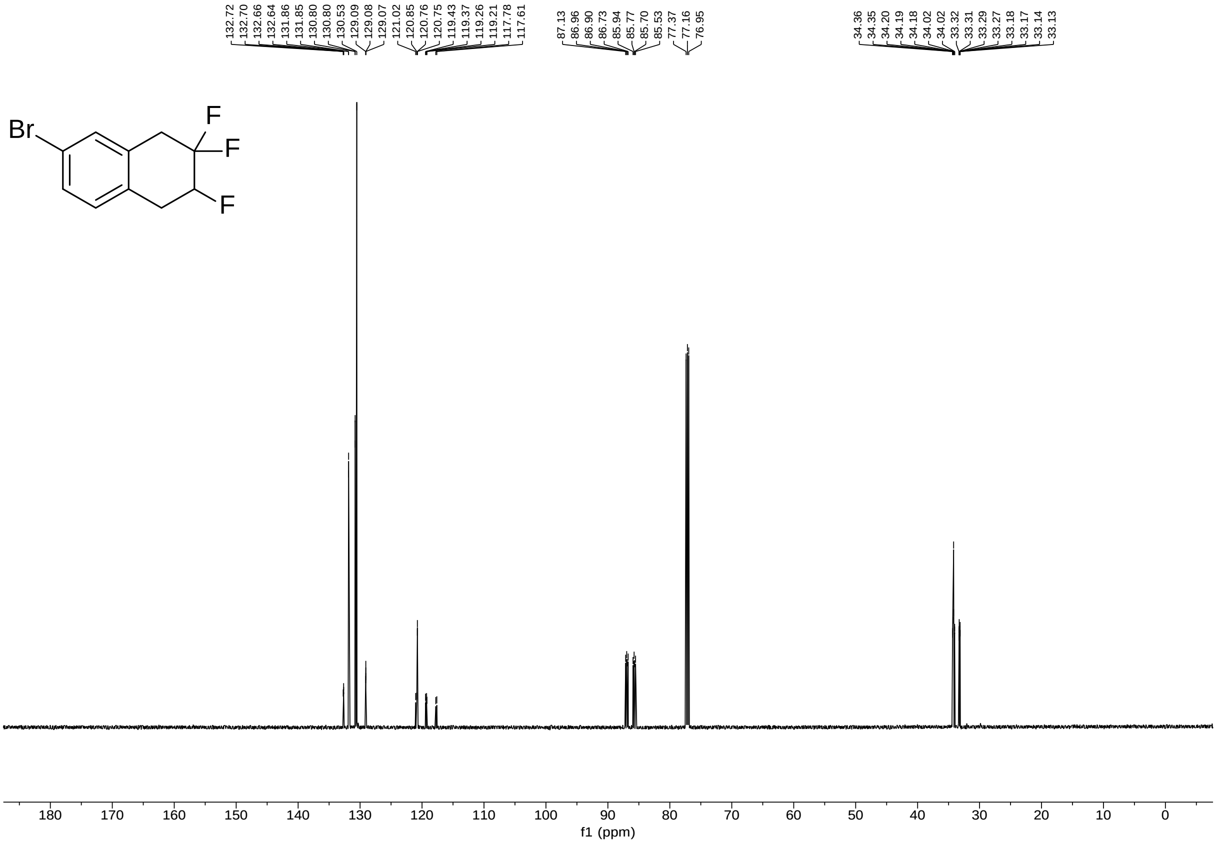

^{19}F NMR (564 MHz, $CDCl_3$, 299 K)

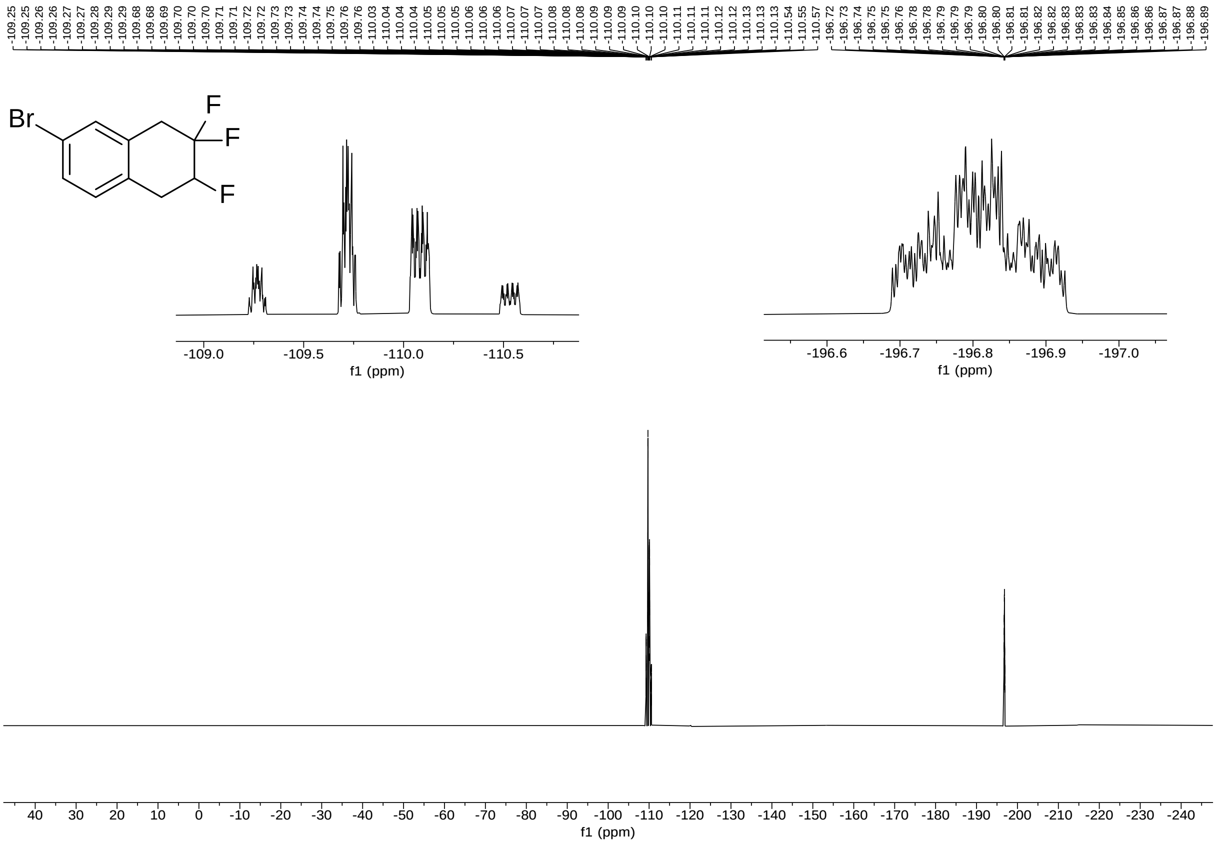

^{19}F{^{1}H} NMR (564 MHz, $CDCl_3$, 299 K)

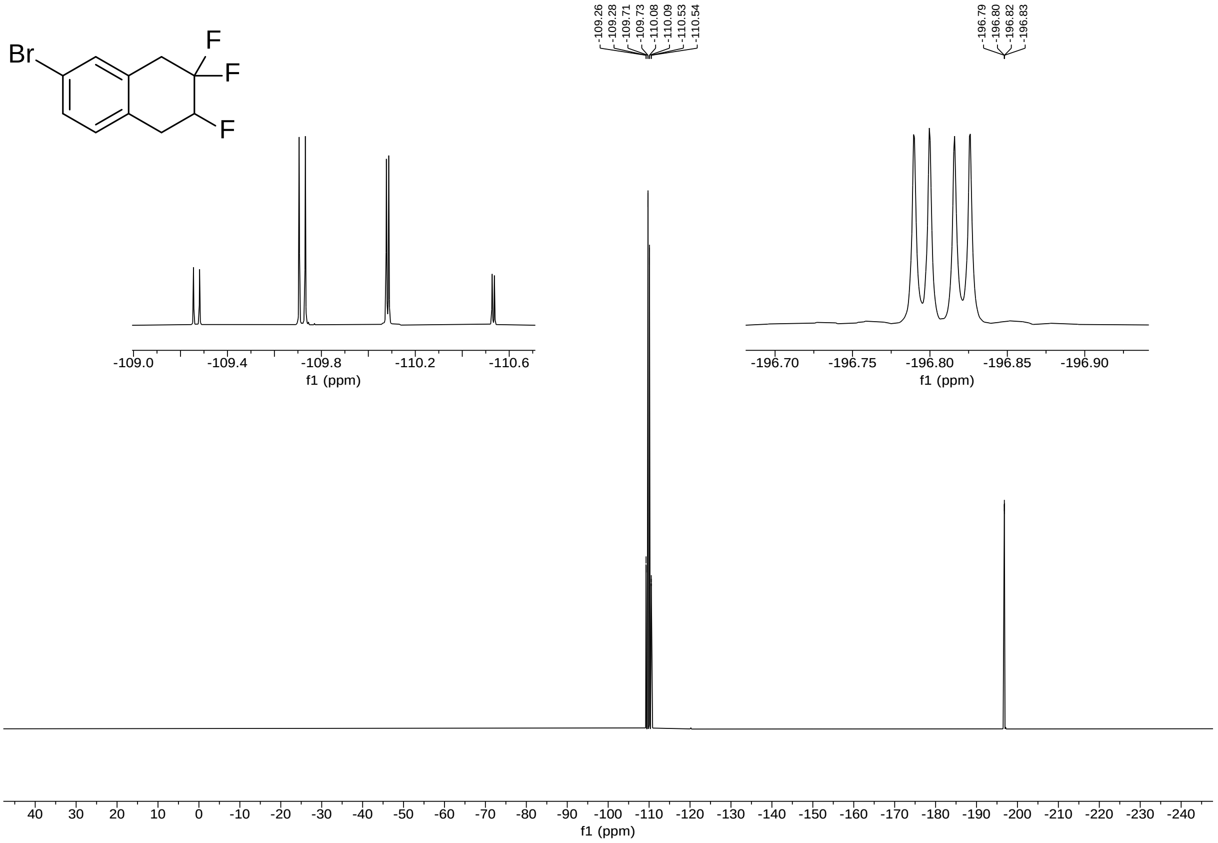

5-Bromo-2,2,3-trifluoro-1,2,3,4-tetrahydronaphthalene (14)

^{1}H NMR (599 MHz, $CDCl_3$, 299 K)

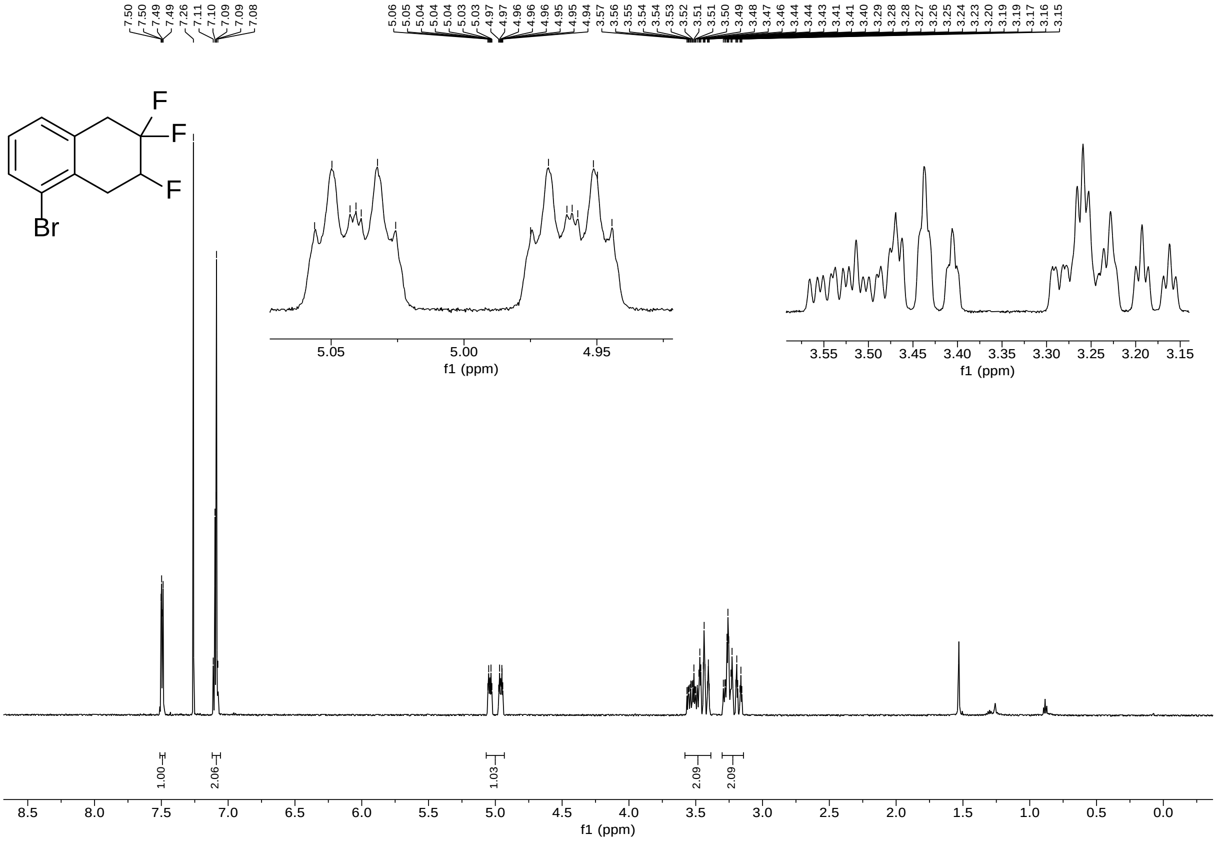

^{13}C NMR (151 MHz, $CDCl_3$, 299 K)

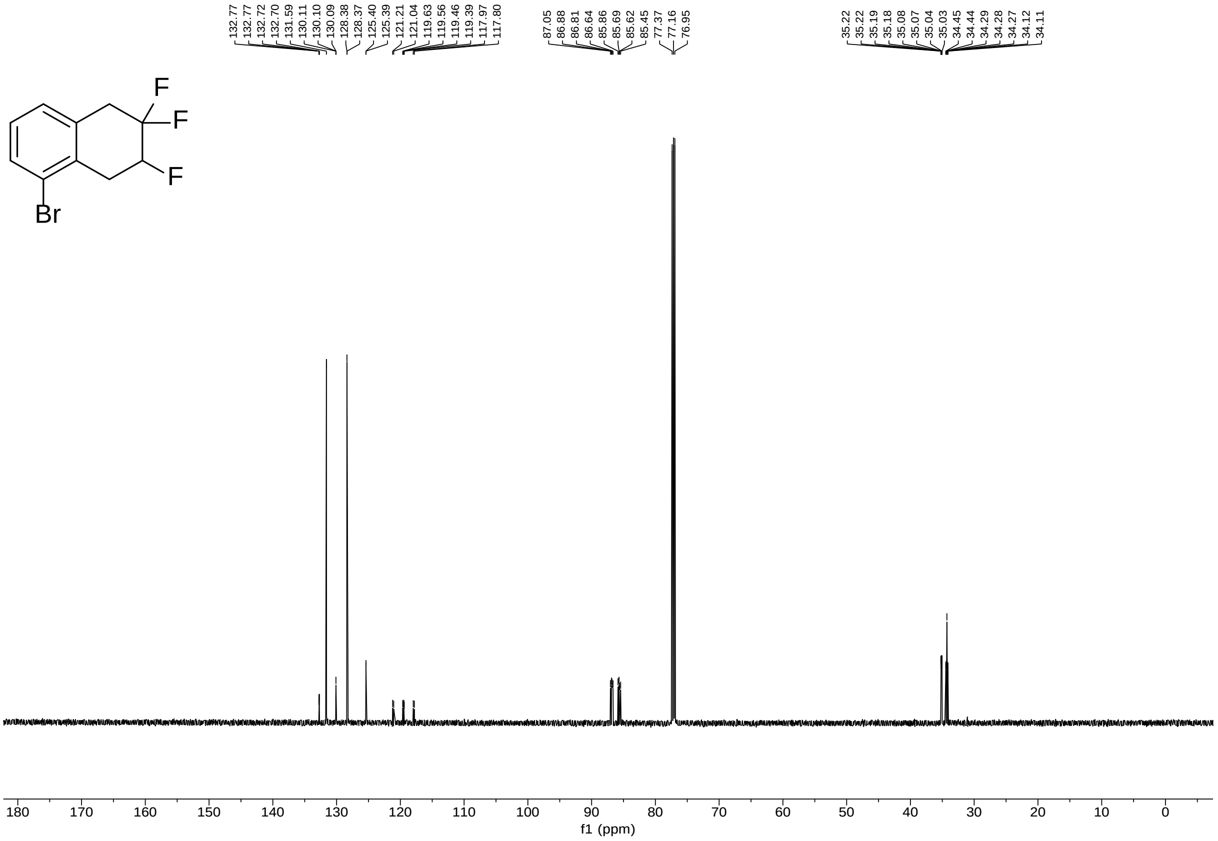

^{19}F NMR (564 MHz, $CDCl_3$, 299 K)

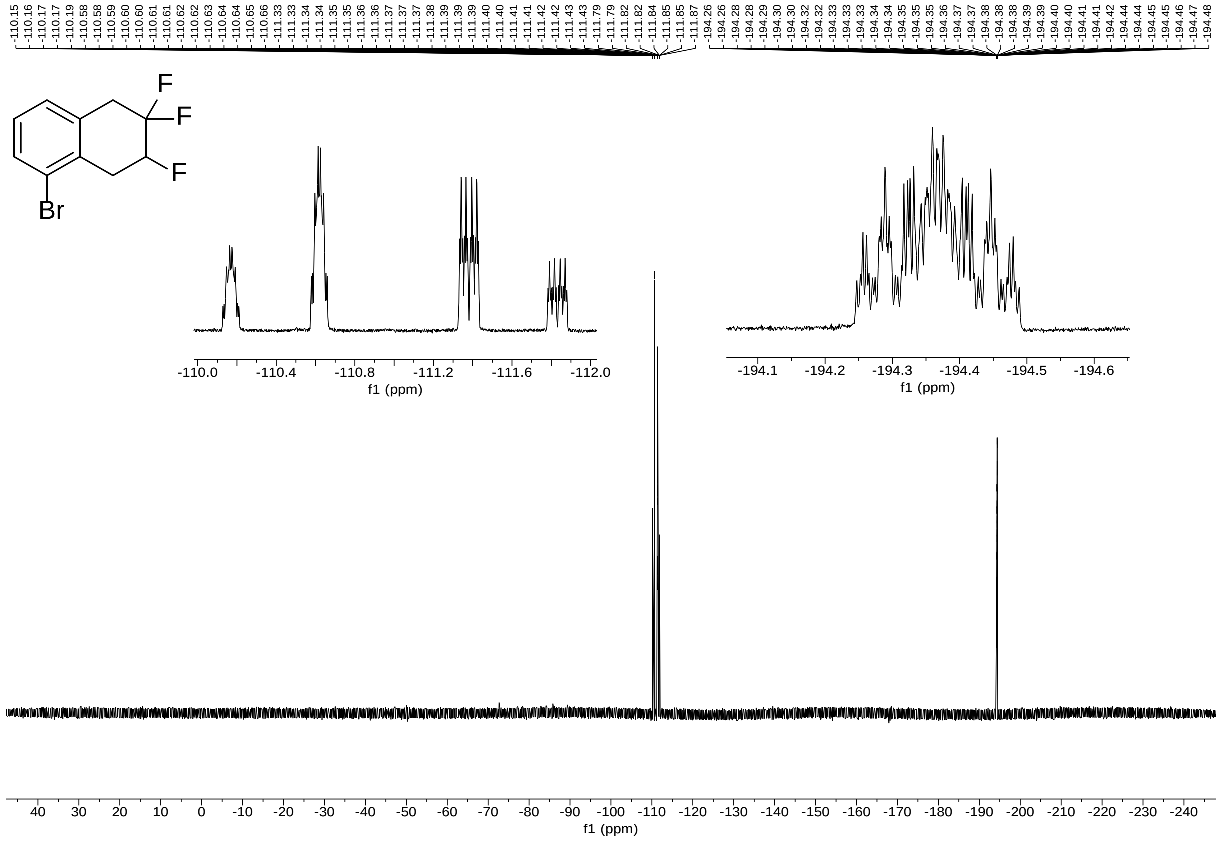

^{19}F{^{1}H} NMR (564 MHz, $CDCl_3$, 299 K)

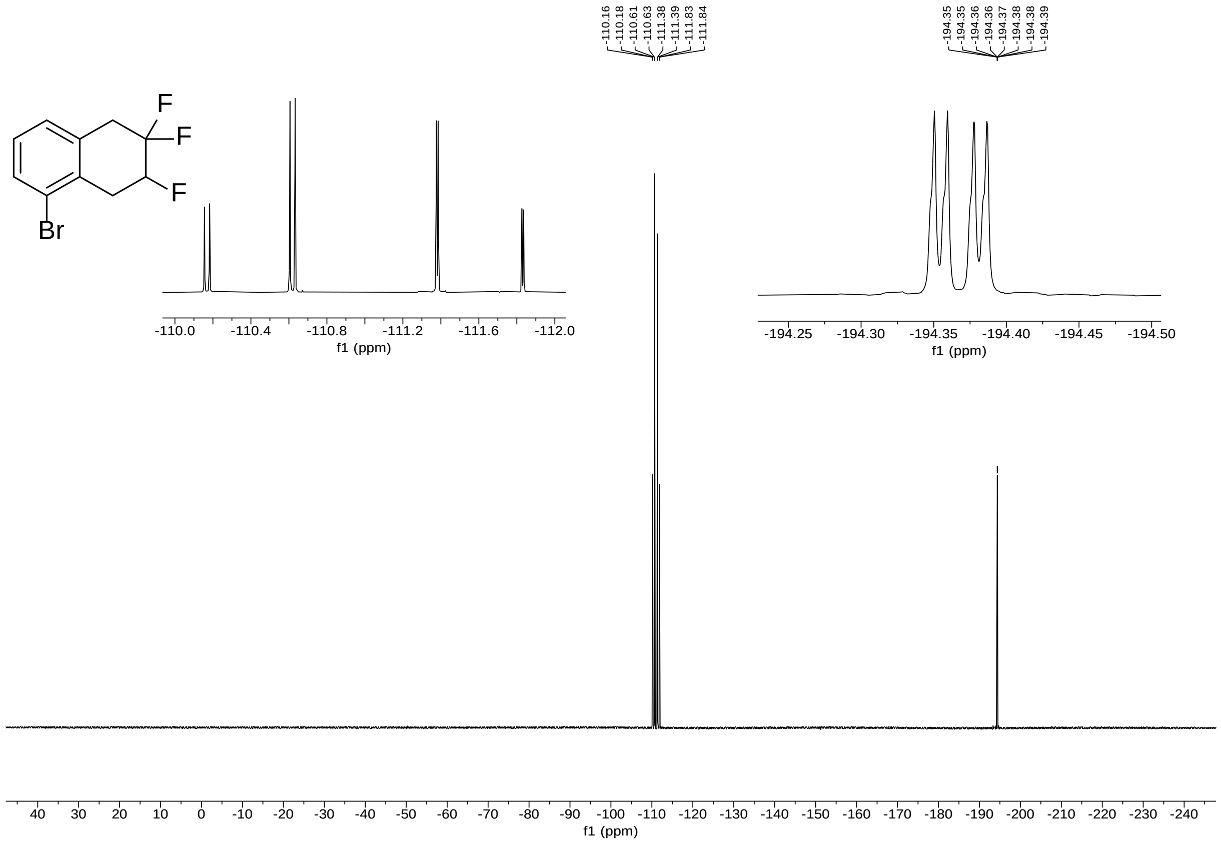

2,2,3-Trifluoro-6-methyl-1,2,3,4-tetrahydronaphthalene (15)

^{1}H NMR (599 MHz, $CDCl_3$, 299 K)

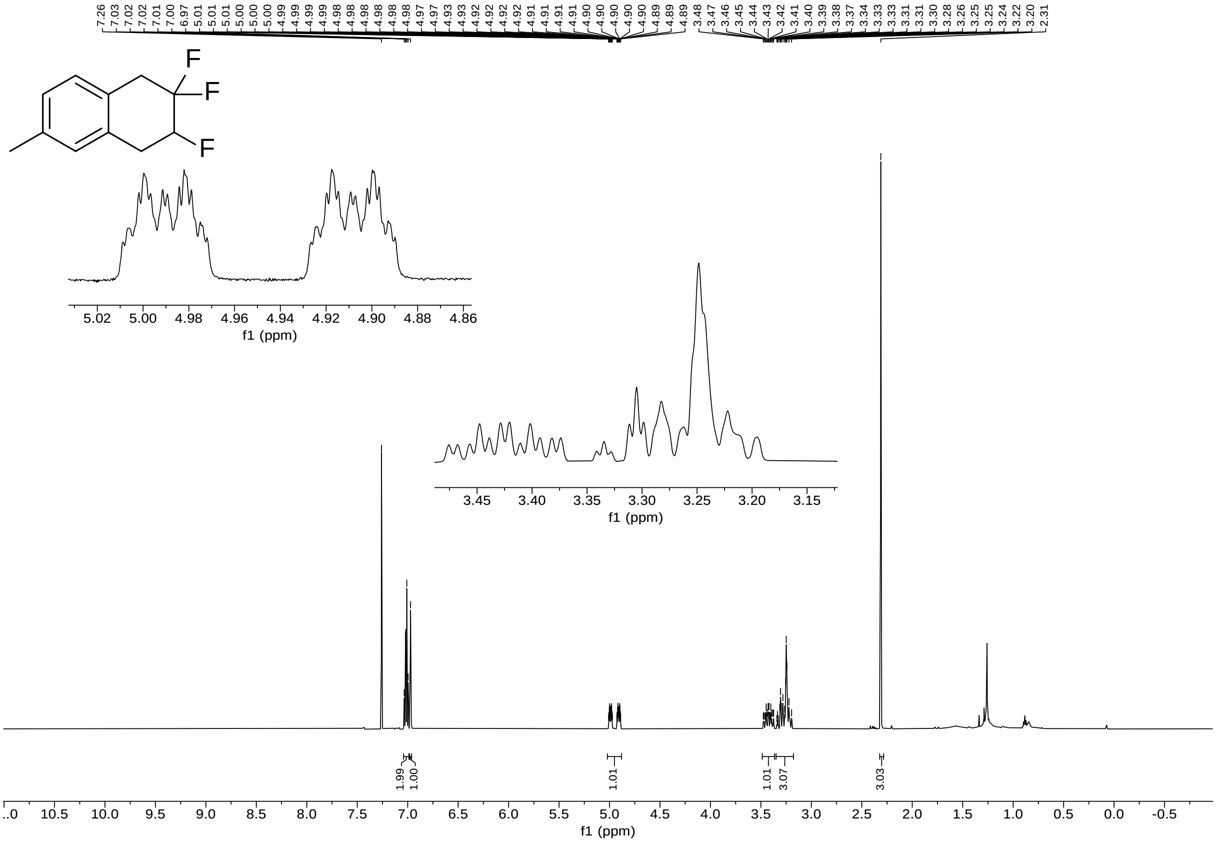

^{13}C NMR (151 MHz, $CDCl_3$, 299 K)

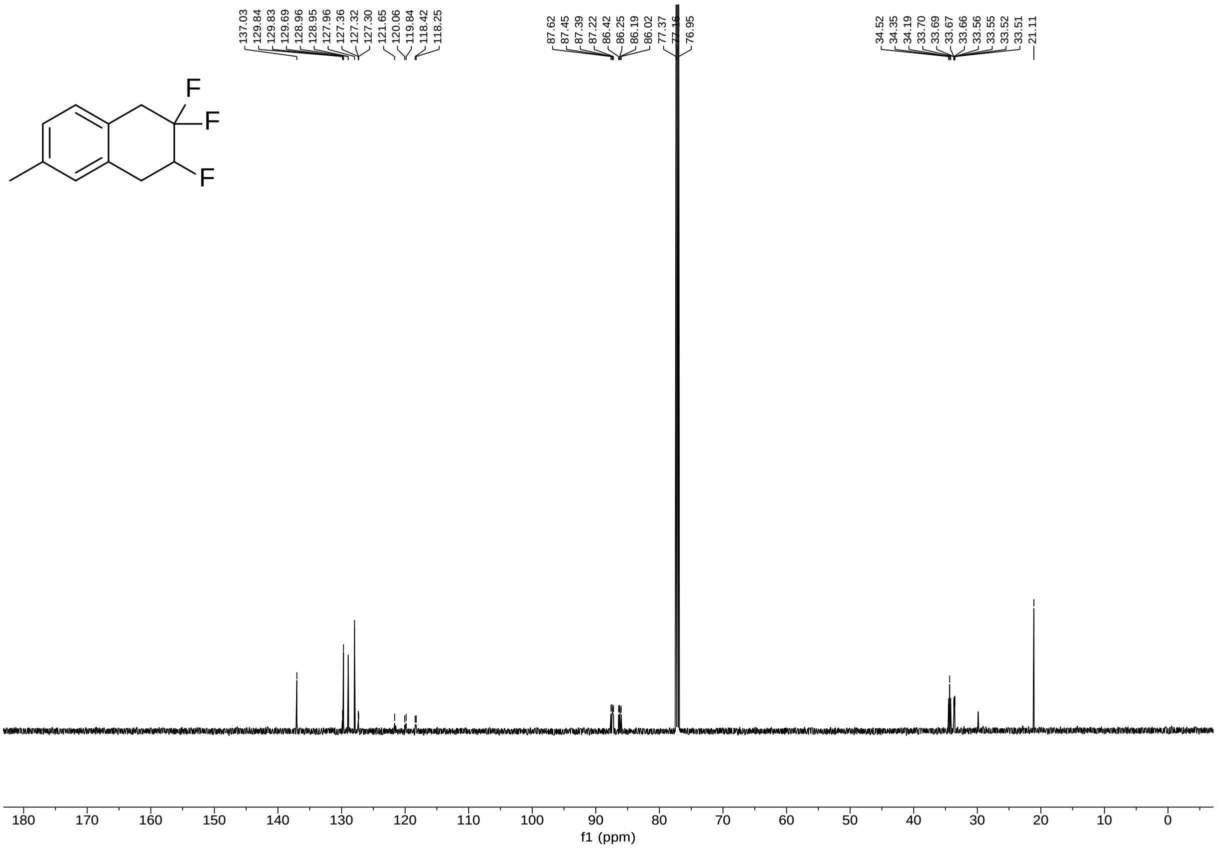

^{19}F NMR (564 MHz, $CDCl_3$, 299 K)

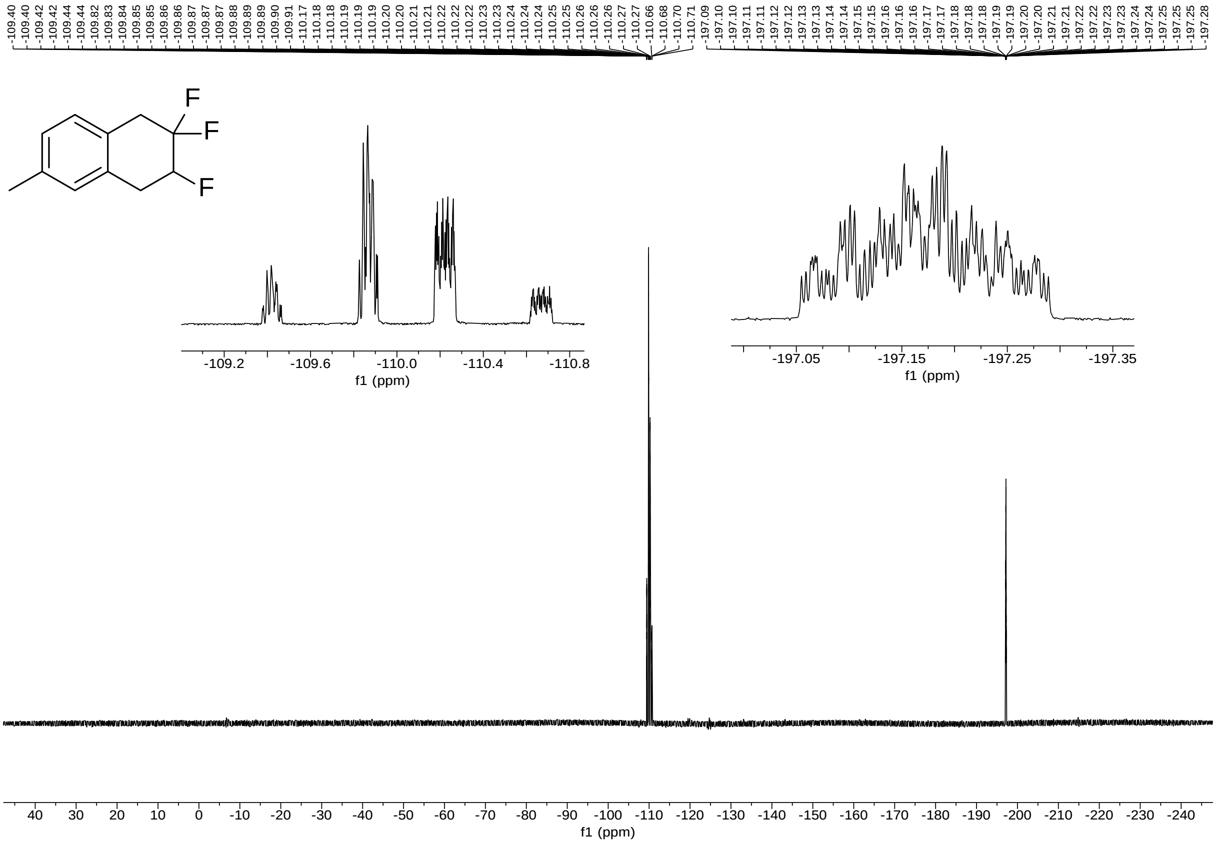

^{19}F{^{1}H} NMR (564 MHz, $CDCl_3$, 299 K)

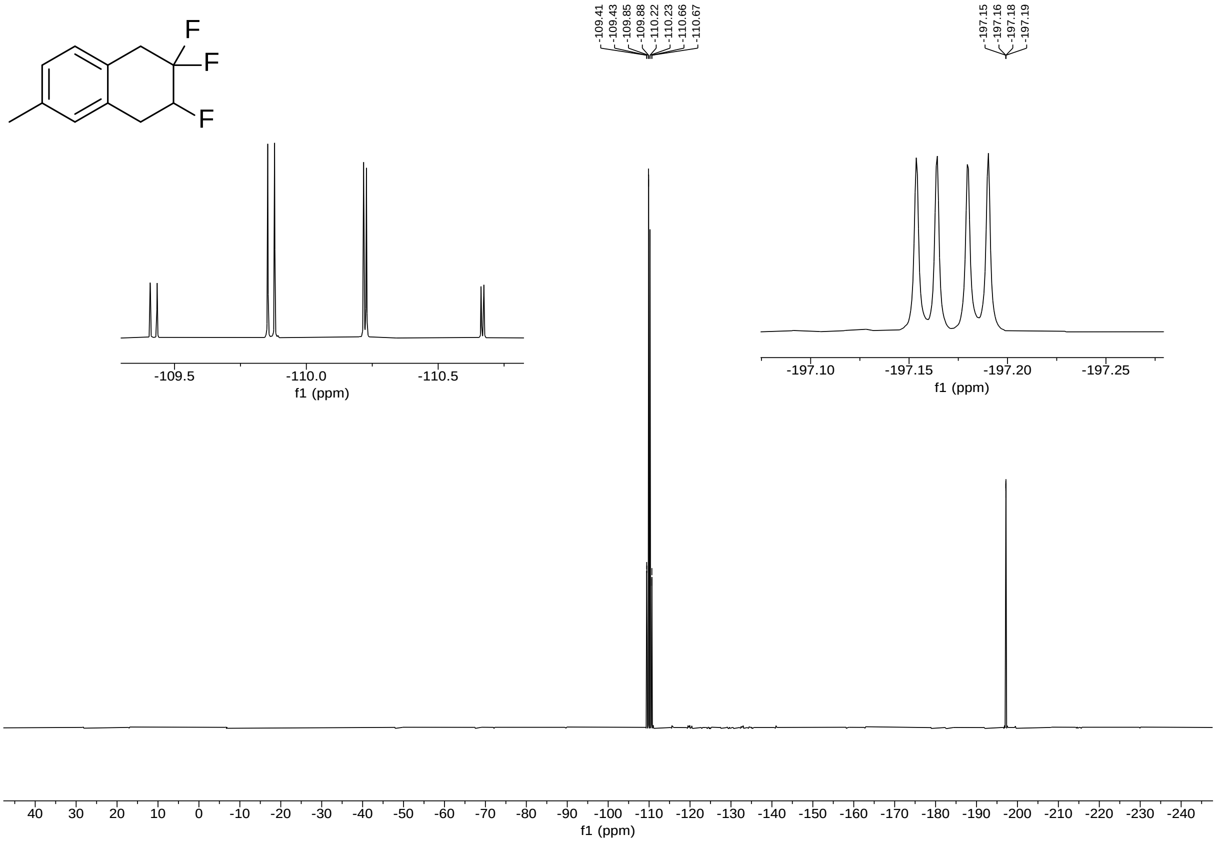

6,6,7-Trifluoro-5,6,7,8-tetrahydronaphthalen-2-yl trfluoromethanesulfonate (16)

^{1}H NMR (599 MHz, $CDCl_3$, 299 K)

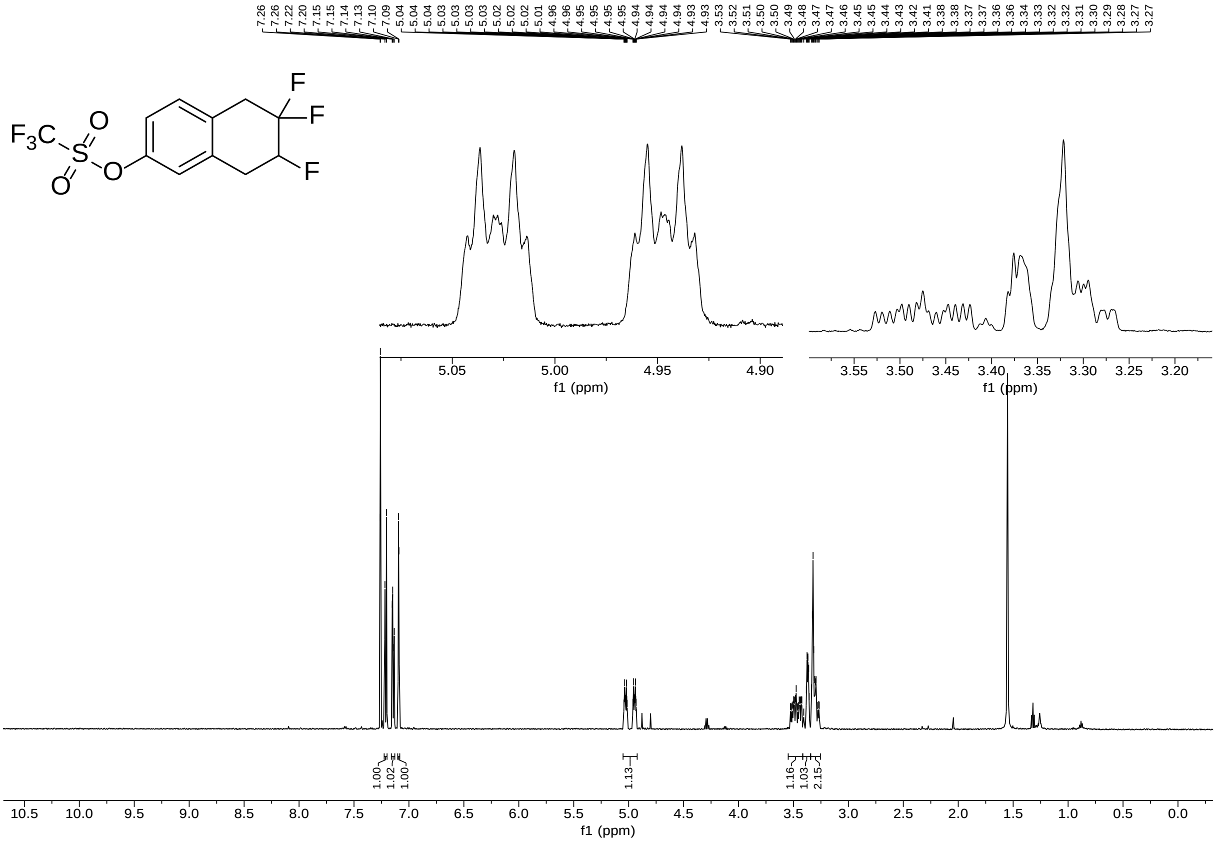

^{13}C NMR (151 MHz, $CDCl_3$, 299 K)

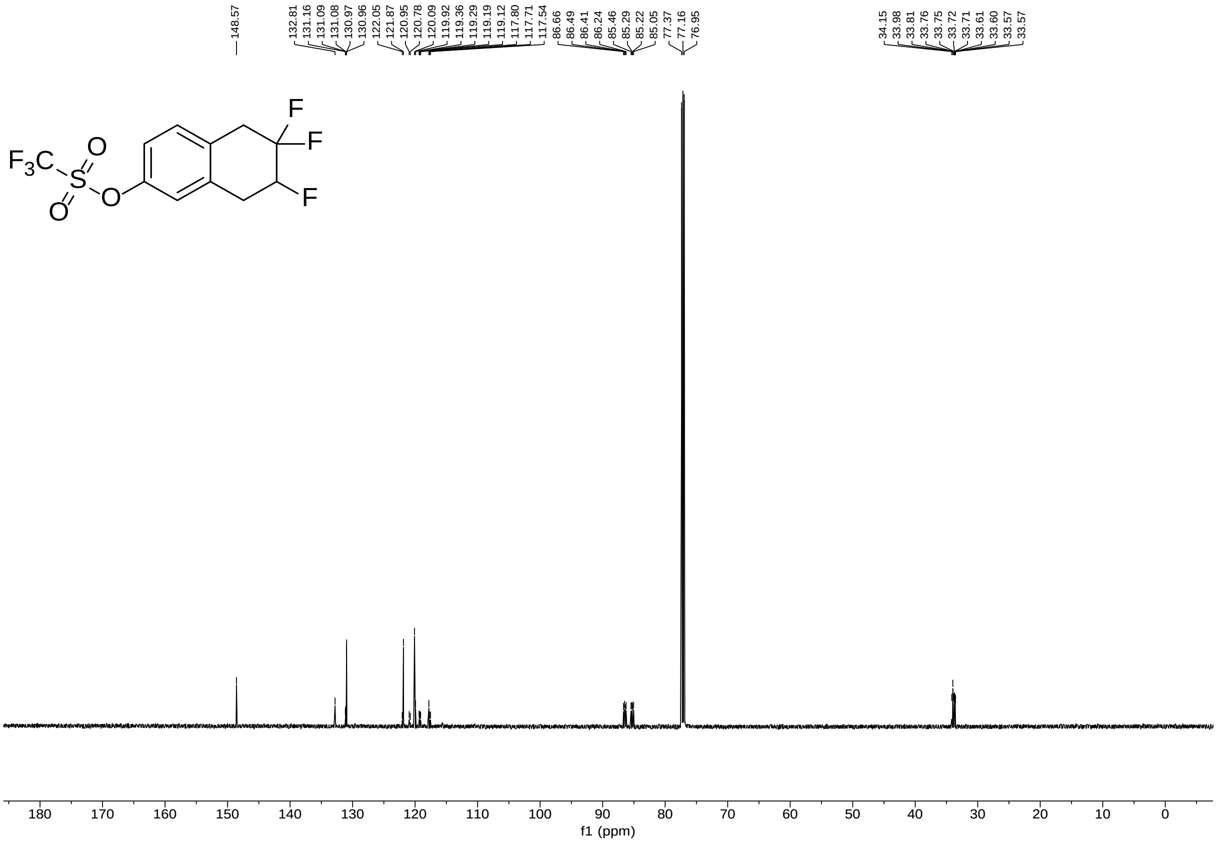

^{19}F NMR (564 MHz, $CDCl_3$, 299 K)

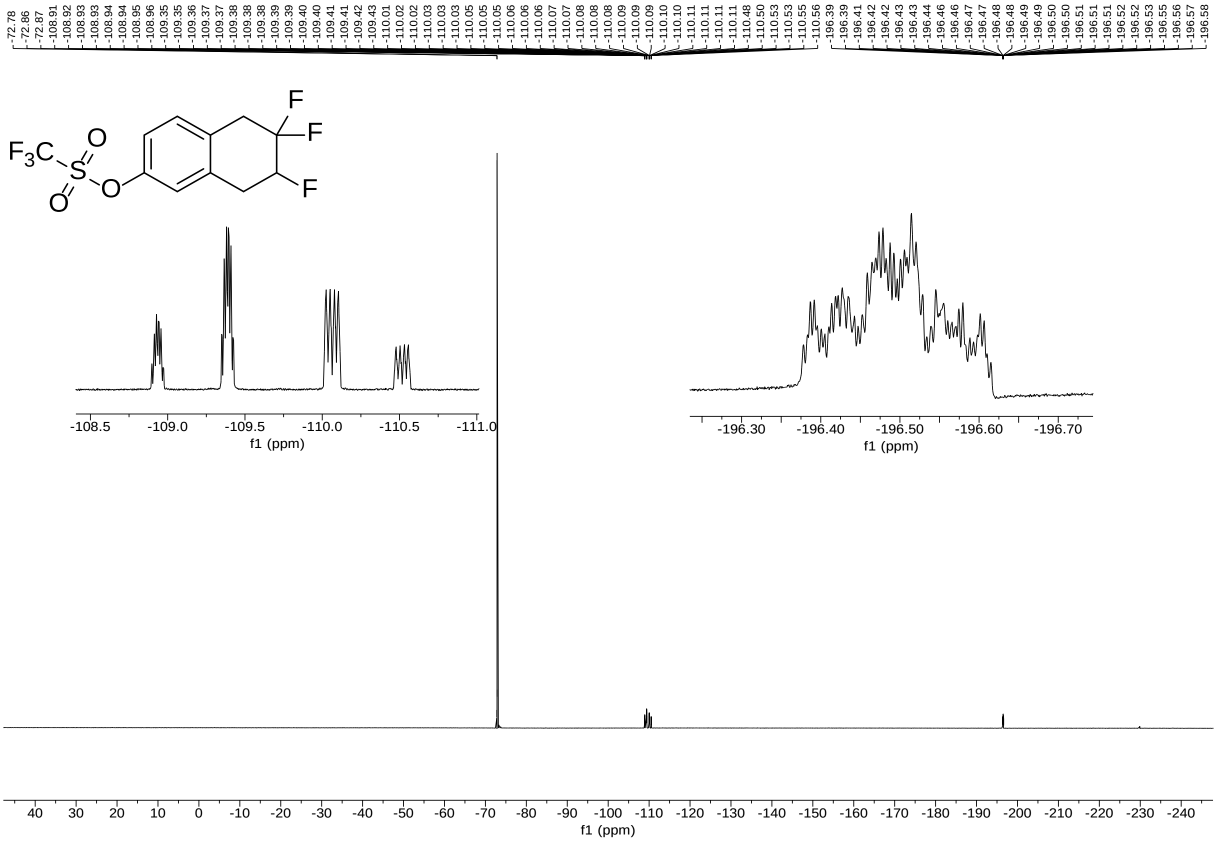

^{19}F{^{1}H} NMR (564 MHz, $CDCl_3$, 299 K)

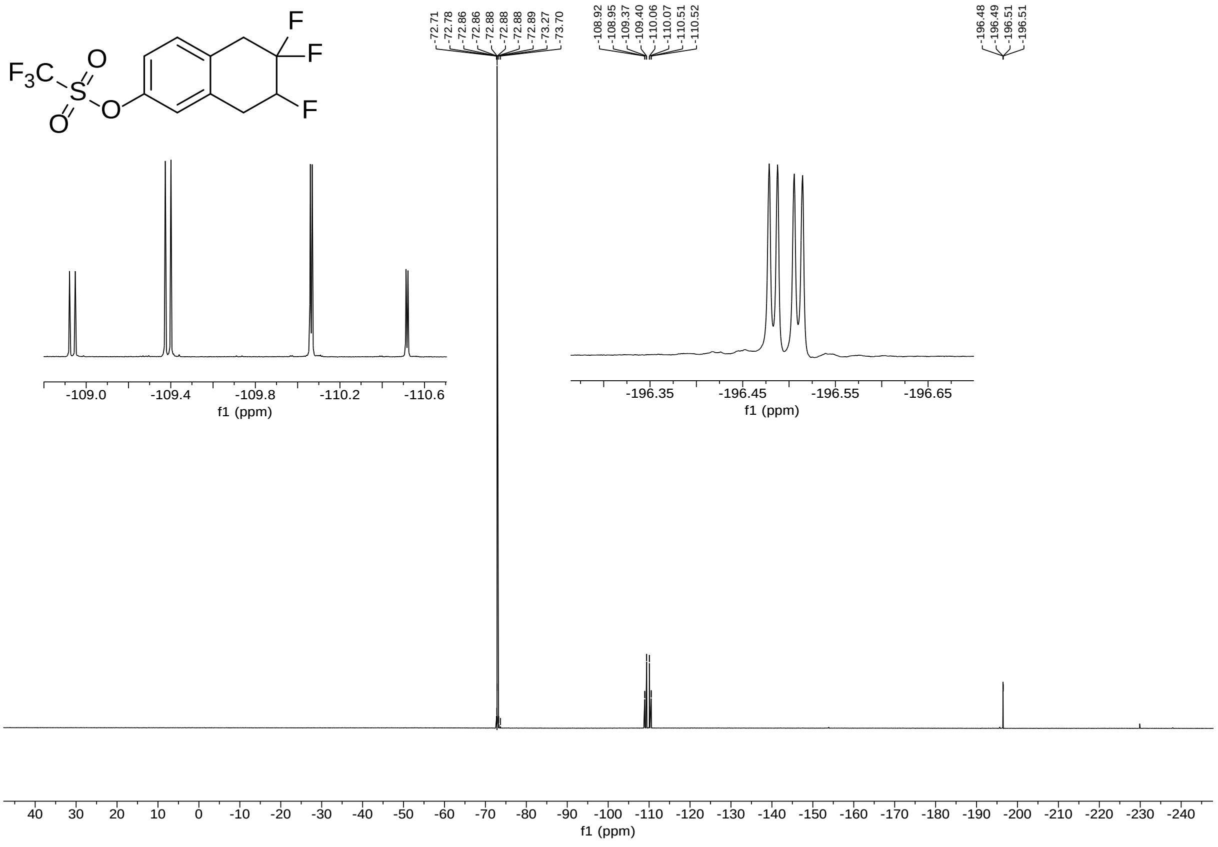

2,2,3Trifluoro-7-(trifluoromethyl)-1,2,3,4-tetrahydronaphthalene (17)

^{1}H NMR (500 MHz, CD_2Cl_2, 299 K)

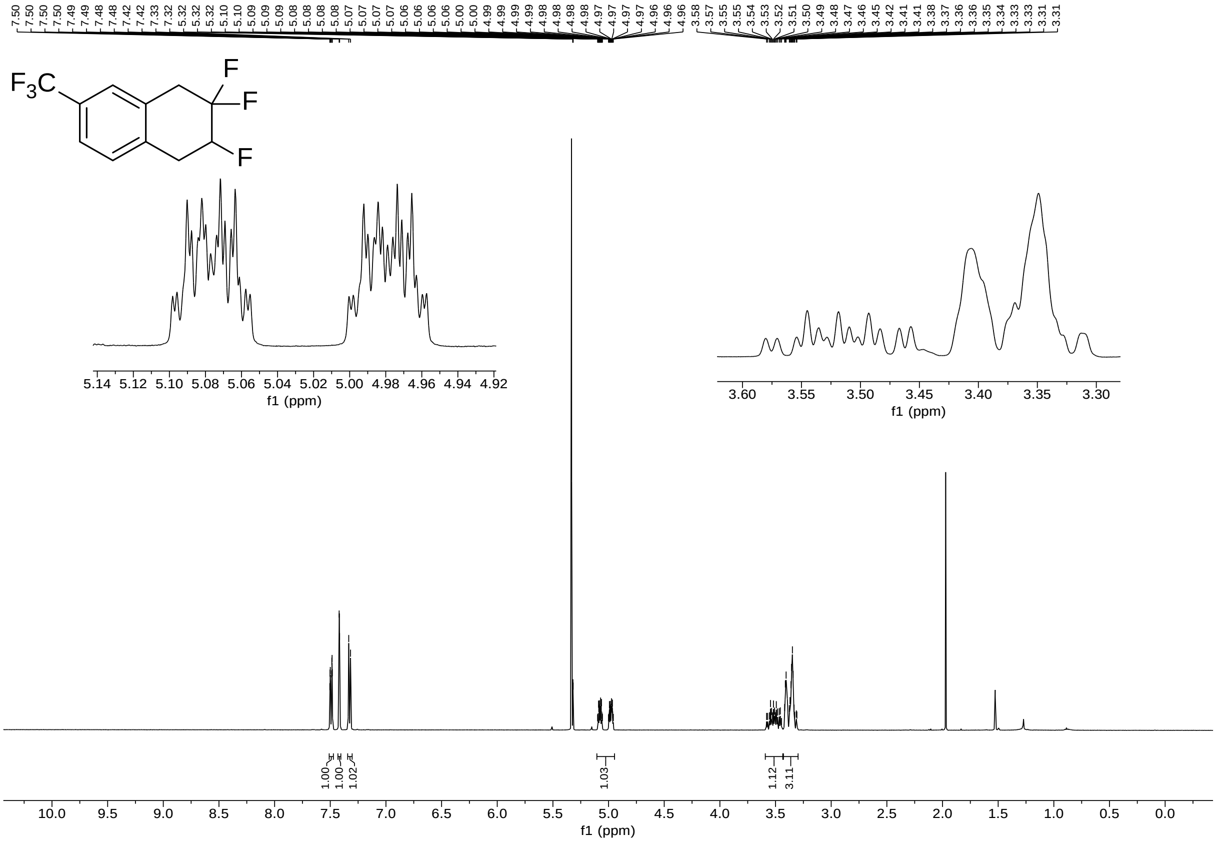

^{13}C NMR (126 MHz, CD_2Cl_2, 299 K)

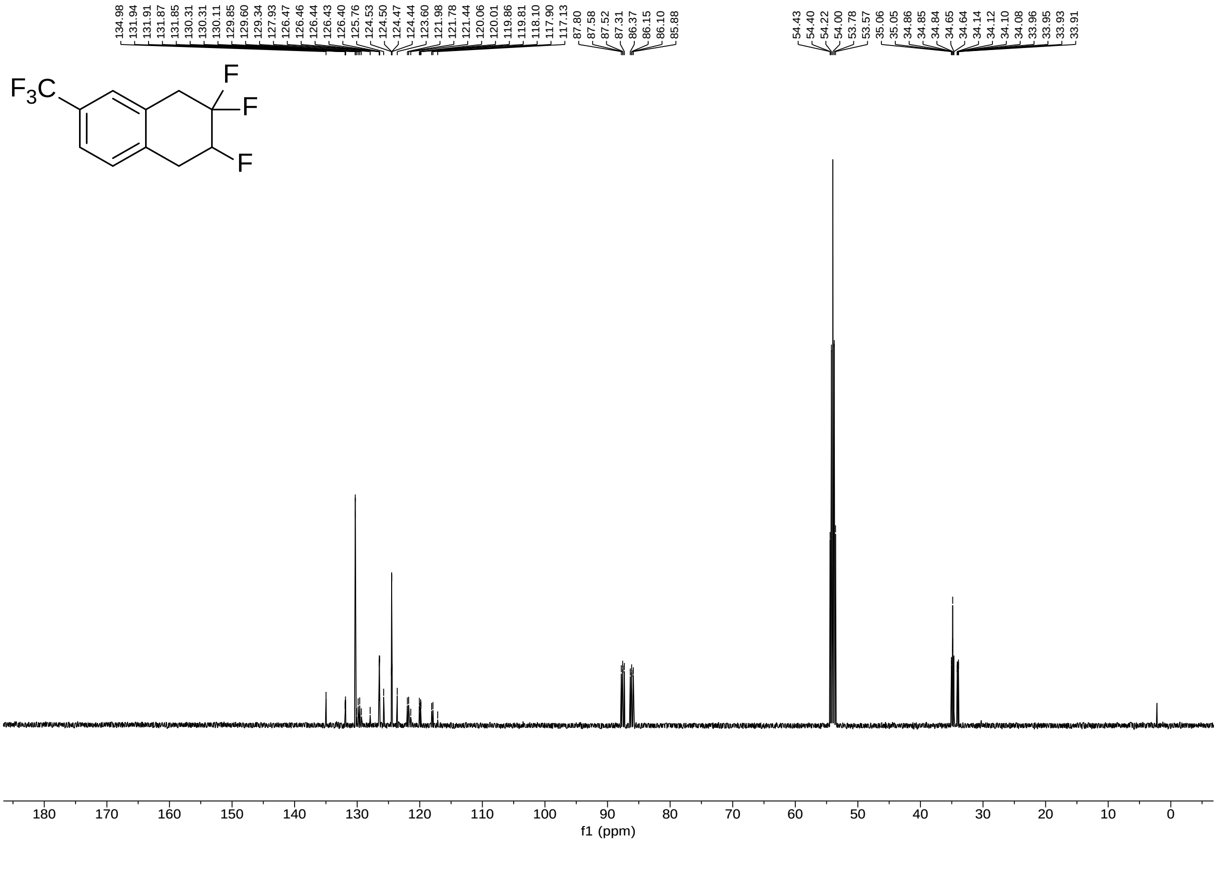

^{19}F NMR (470 MHz, CD_2Cl_2, 299 K)

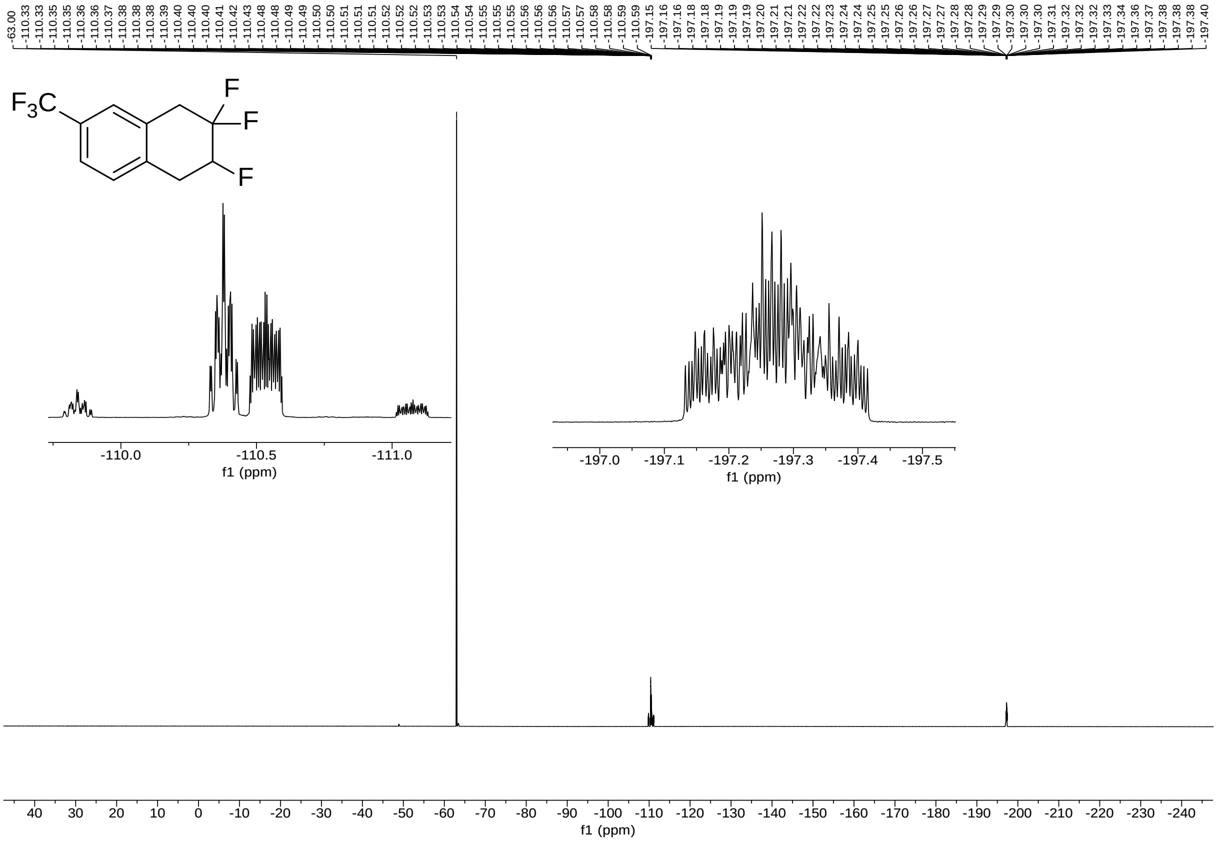

^{19}F{^{1}H} NMR (470 MHz, CD_2Cl_2, 299 K)

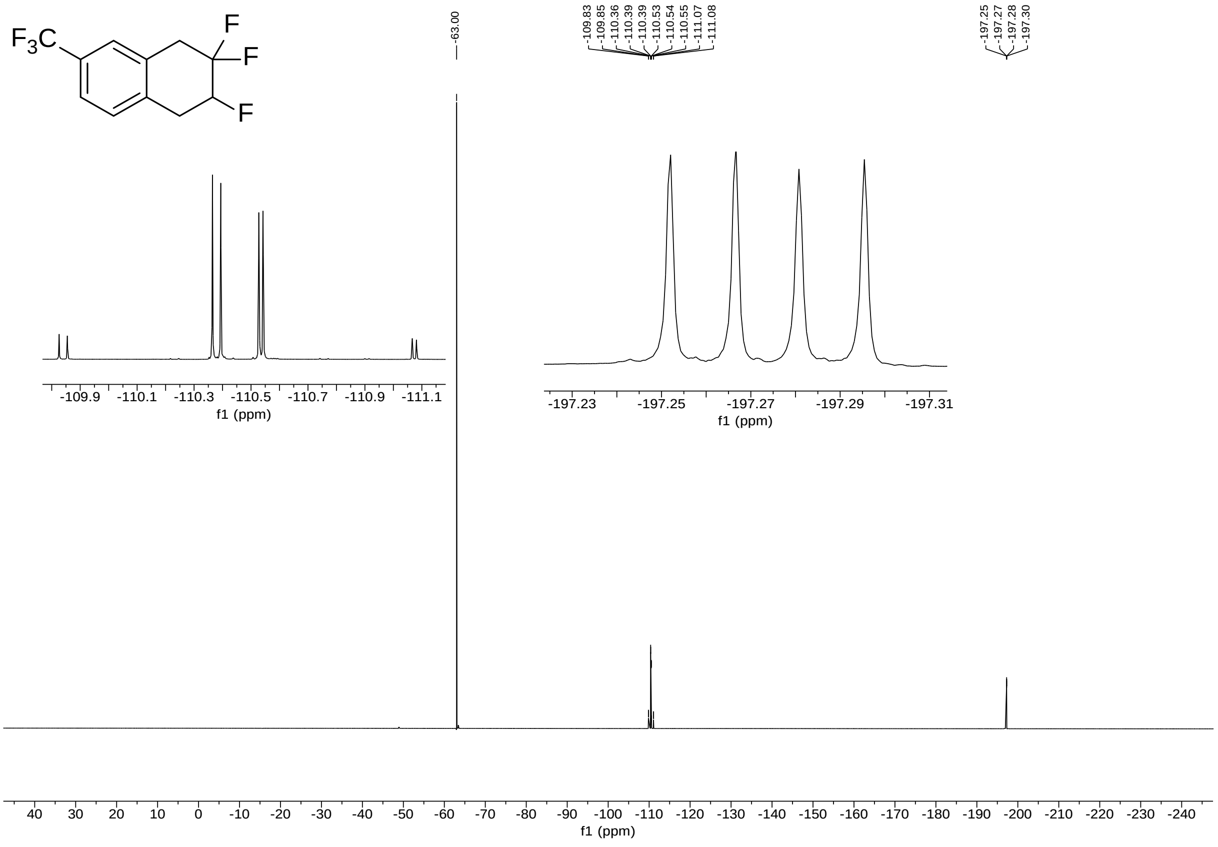

6,6,7-Trifluoro-5,6,7,8-tetrahydronaphthalene-2-carbonitrile (S18)

^{1}H NMR (599 MHz, CD_2Cl_2, 299 K)

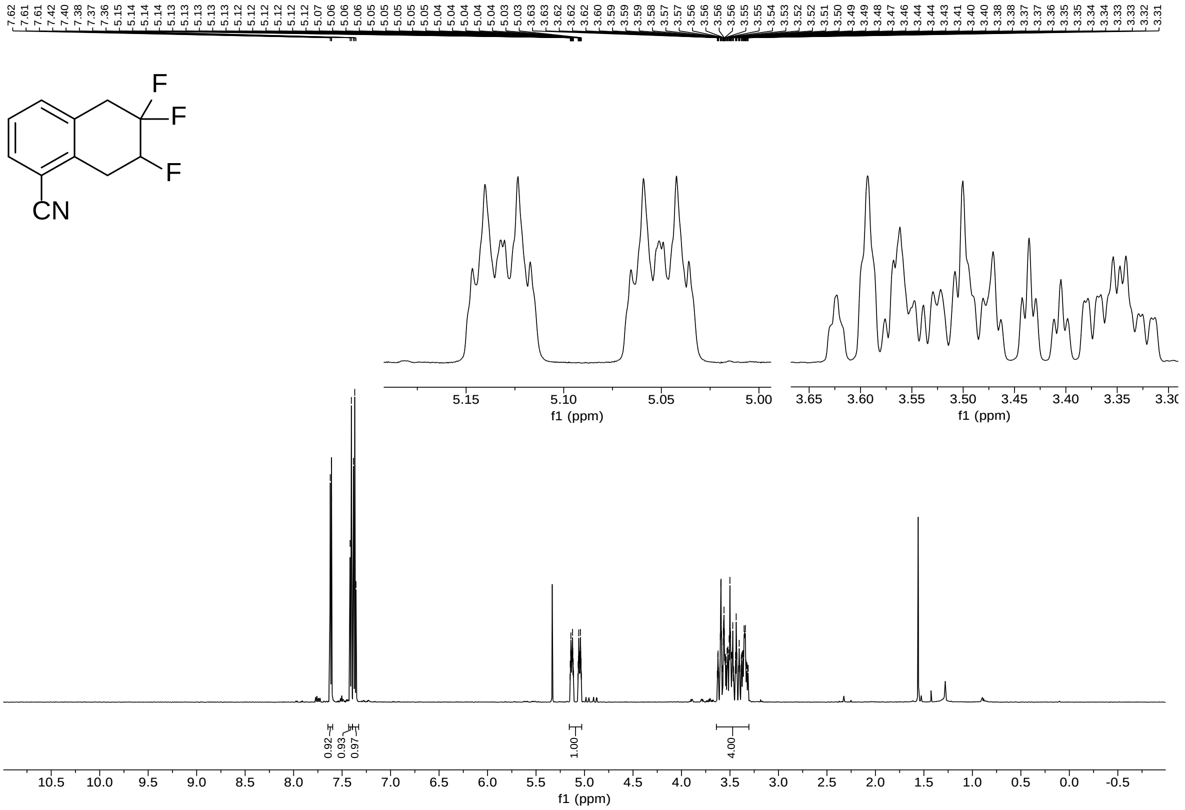

^{13}C NMR (151 MHz, CD_2Cl_2, 299 K)

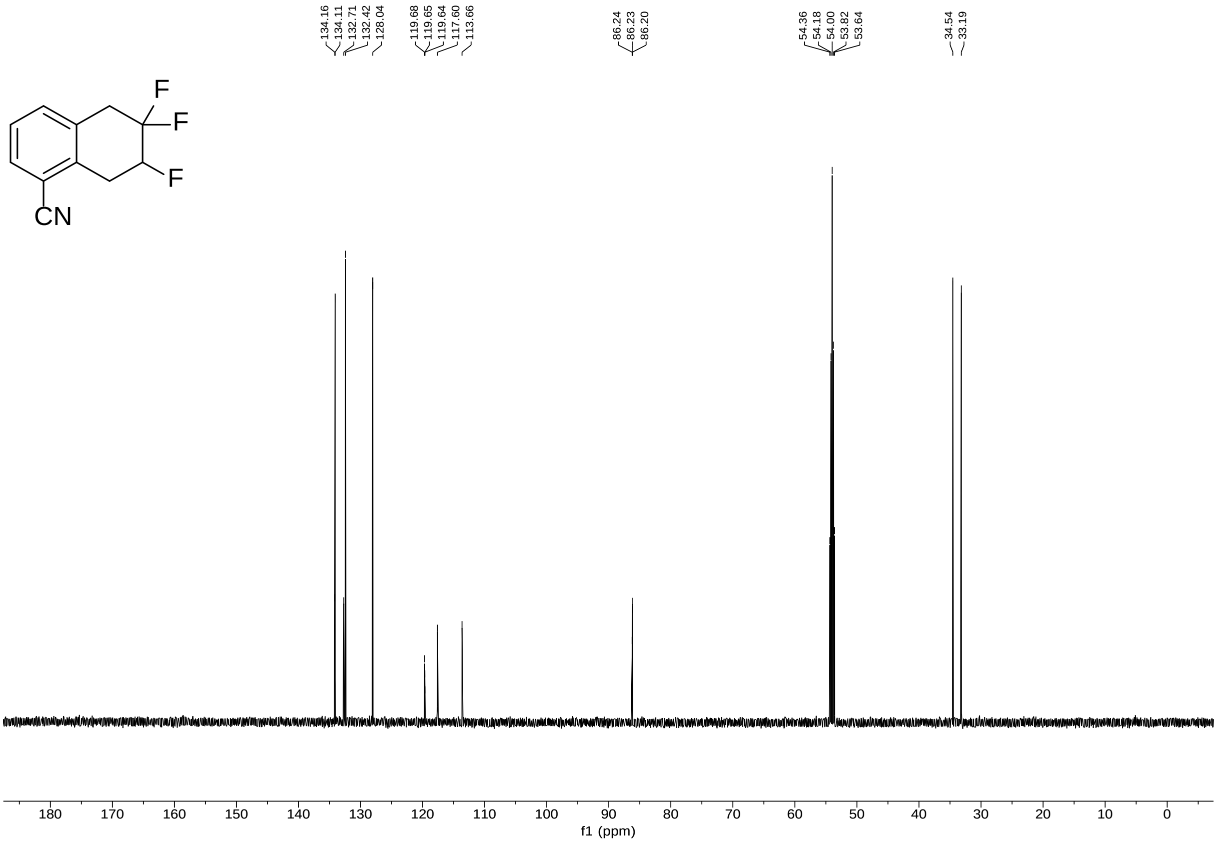

^{19}F NMR (564 MHz, CD_2Cl_2, 299 K)

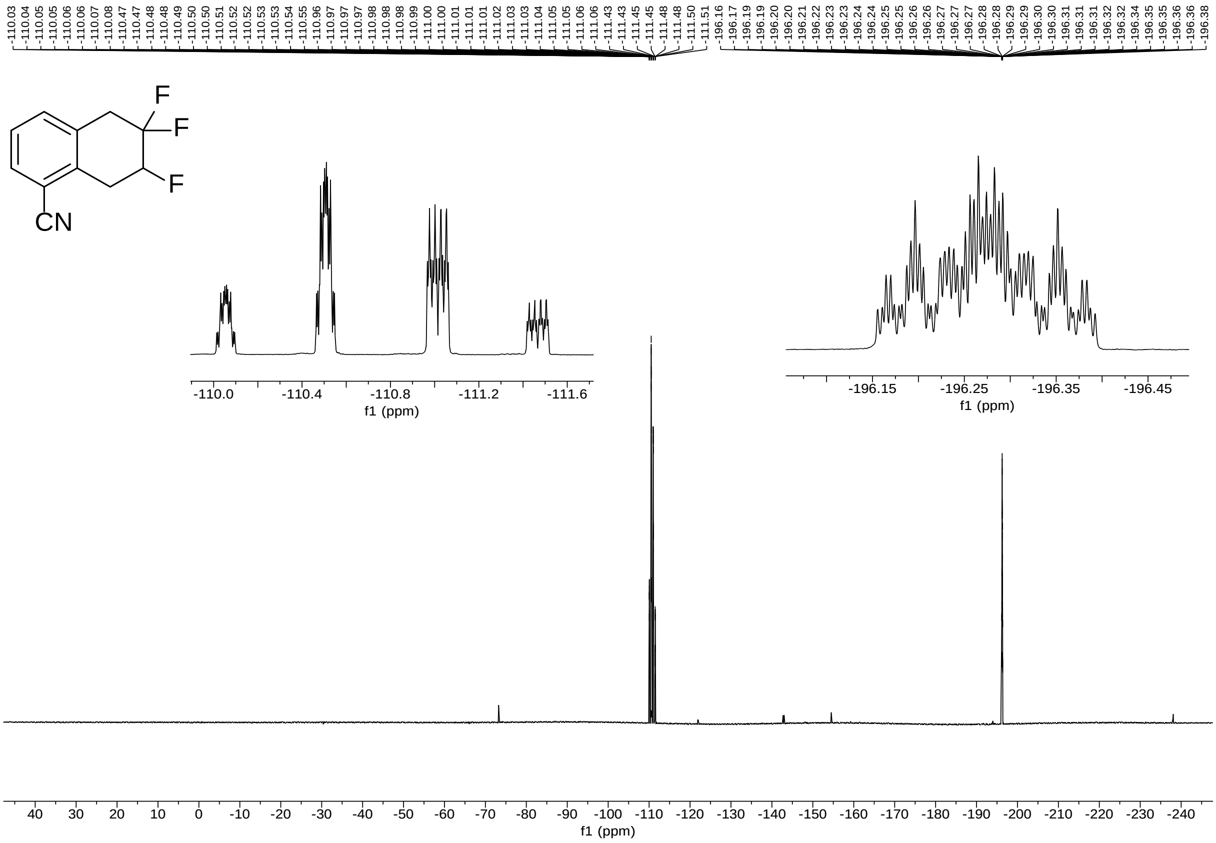

^{19}F{^{1}H} NMR (564 MHz, CD_2Cl_2, 299 K)

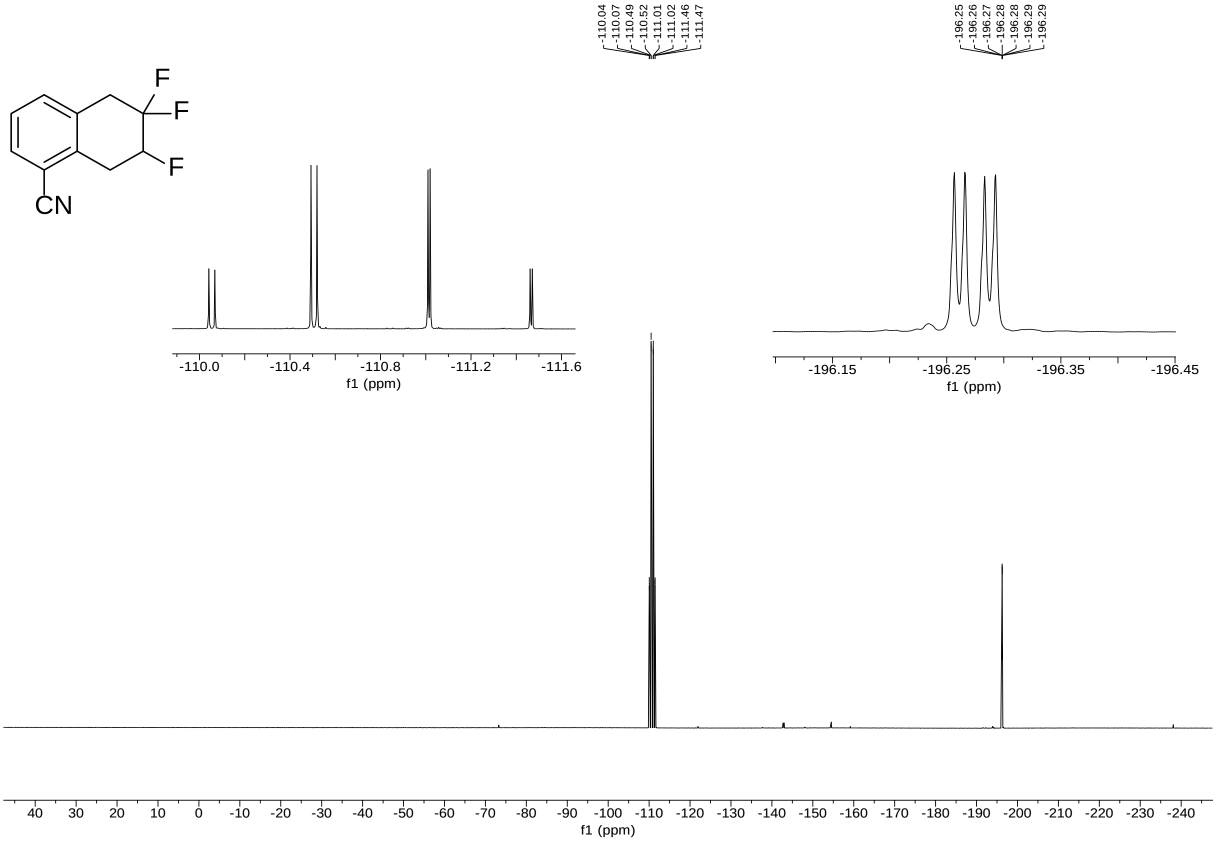

2-(6,7,7-Trifluoro-5,6,7,8-tetrahydronaphthalen-2-yl)isoindoline-1,3-dione (19)

^{1}H NMR (500 MHz, $CDCl_3$, 299 K)

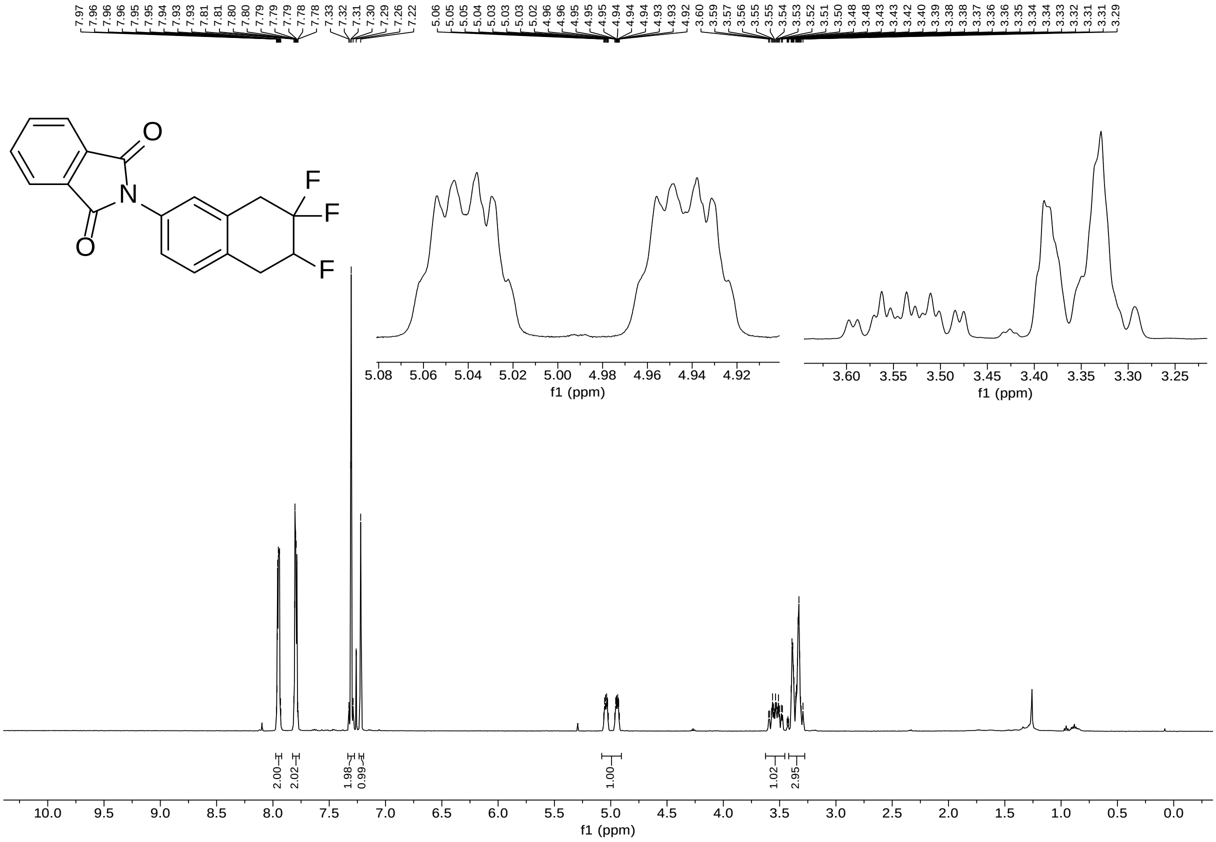

^{13}C NMR (126 MHz, $CDCl_3$, 299 K)

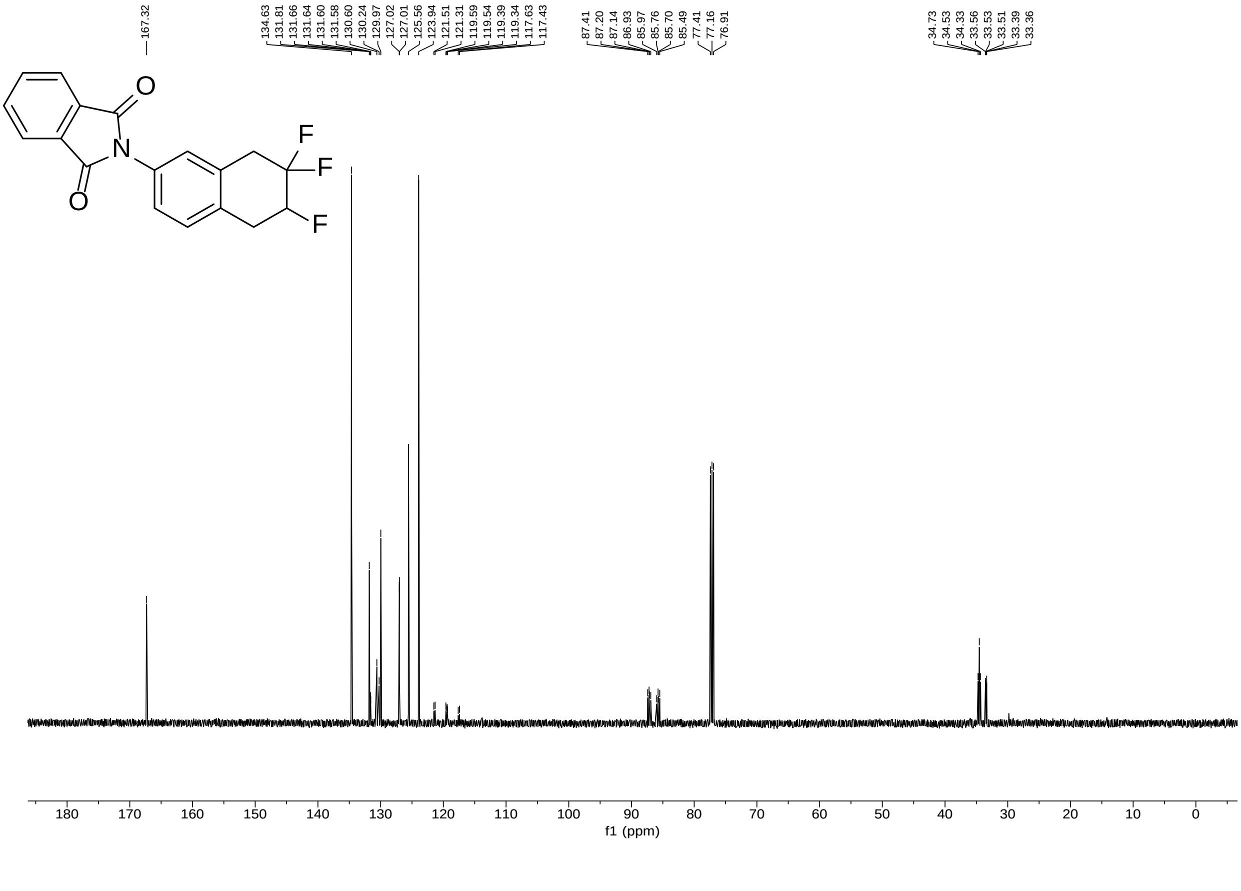

^{19}F NMR (470 MHz, $CDCl_3$, 299 K)

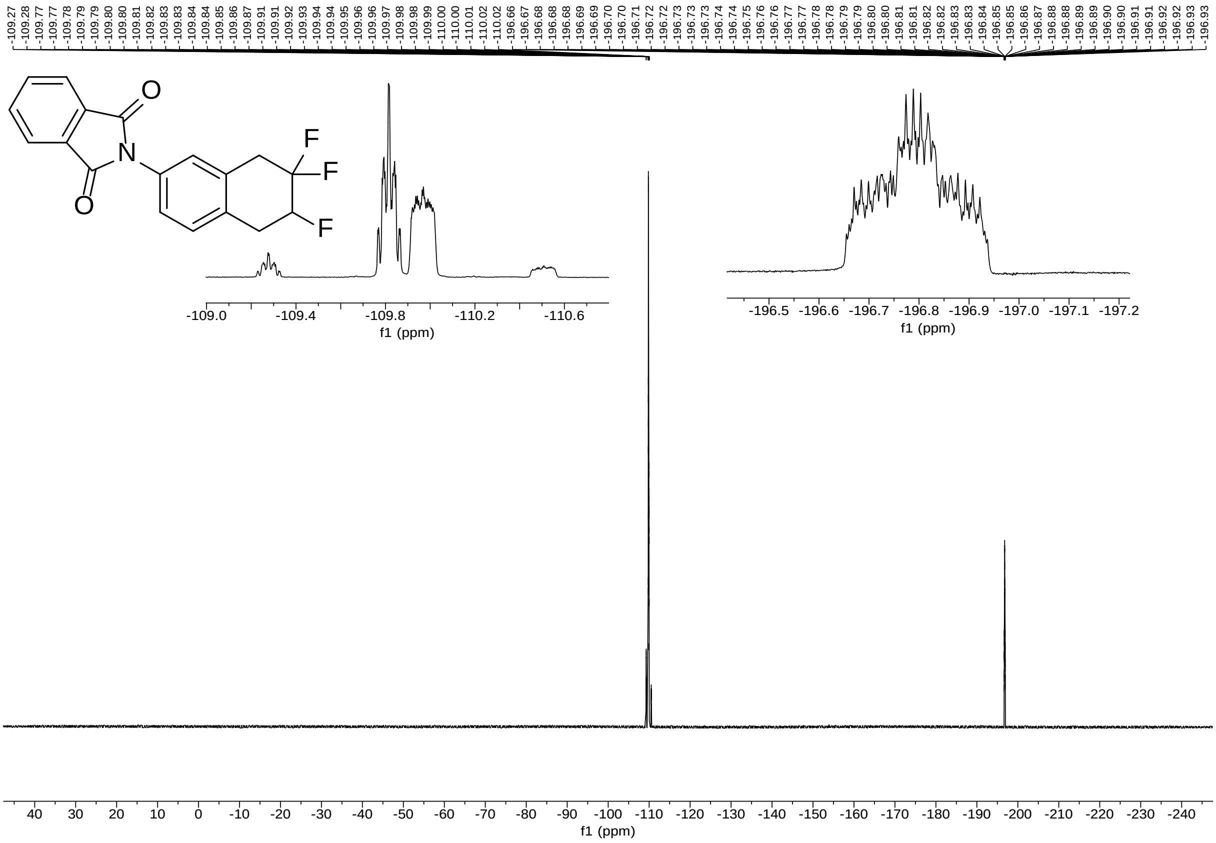

$^{19}F\{^1H\}$ NMR (470 MHz, $CDCl_3$, 299 K)

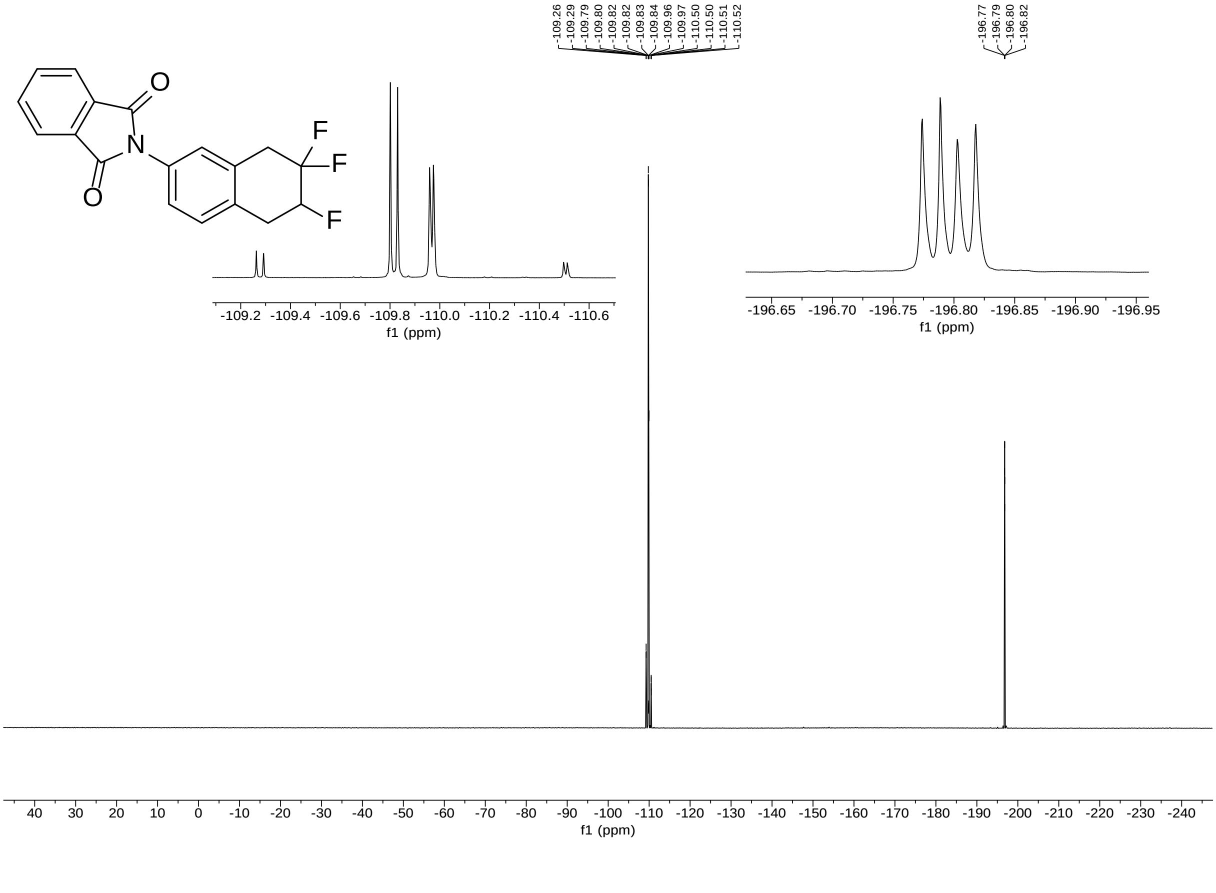

Methyl (*E*)-3-(6,6,7-trifluoro-5,6,7,8-tetrahydronaphthalen-2-yl)acrylate (20)

^{1}H NMR (500 MHz, $CDCl_3$, 299 K)

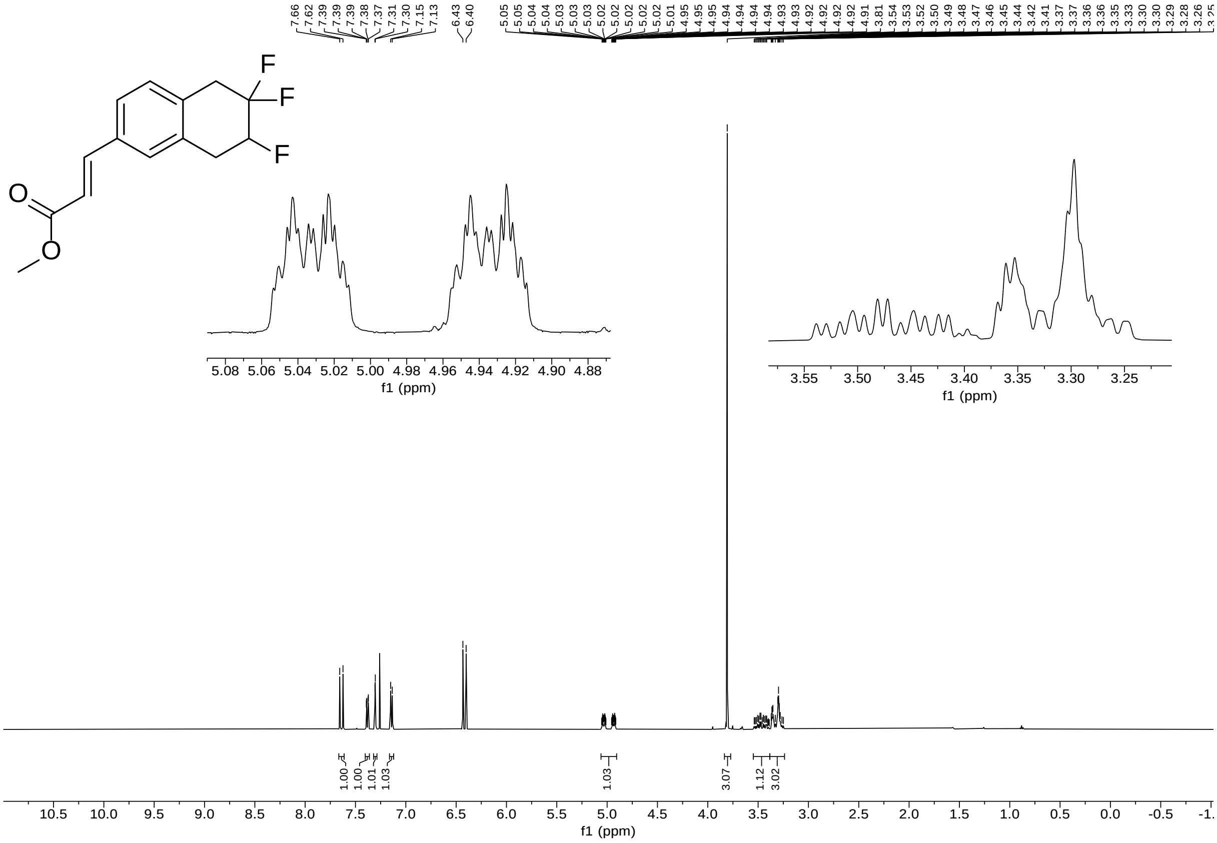

^{13}C NMR (126 MHz, $CDCl_3$, 299 K)

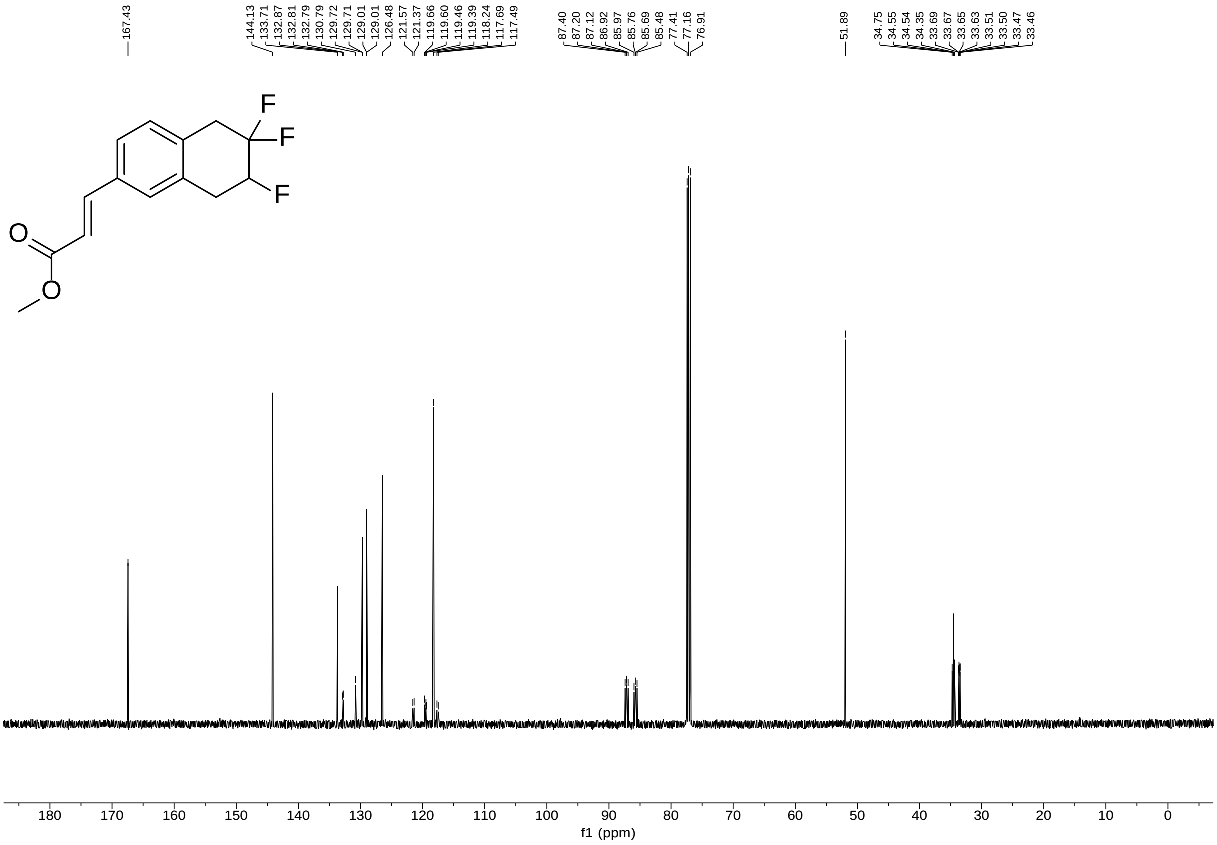

^{19}F NMR (470 MHz, $CDCl_3$, 299 K)

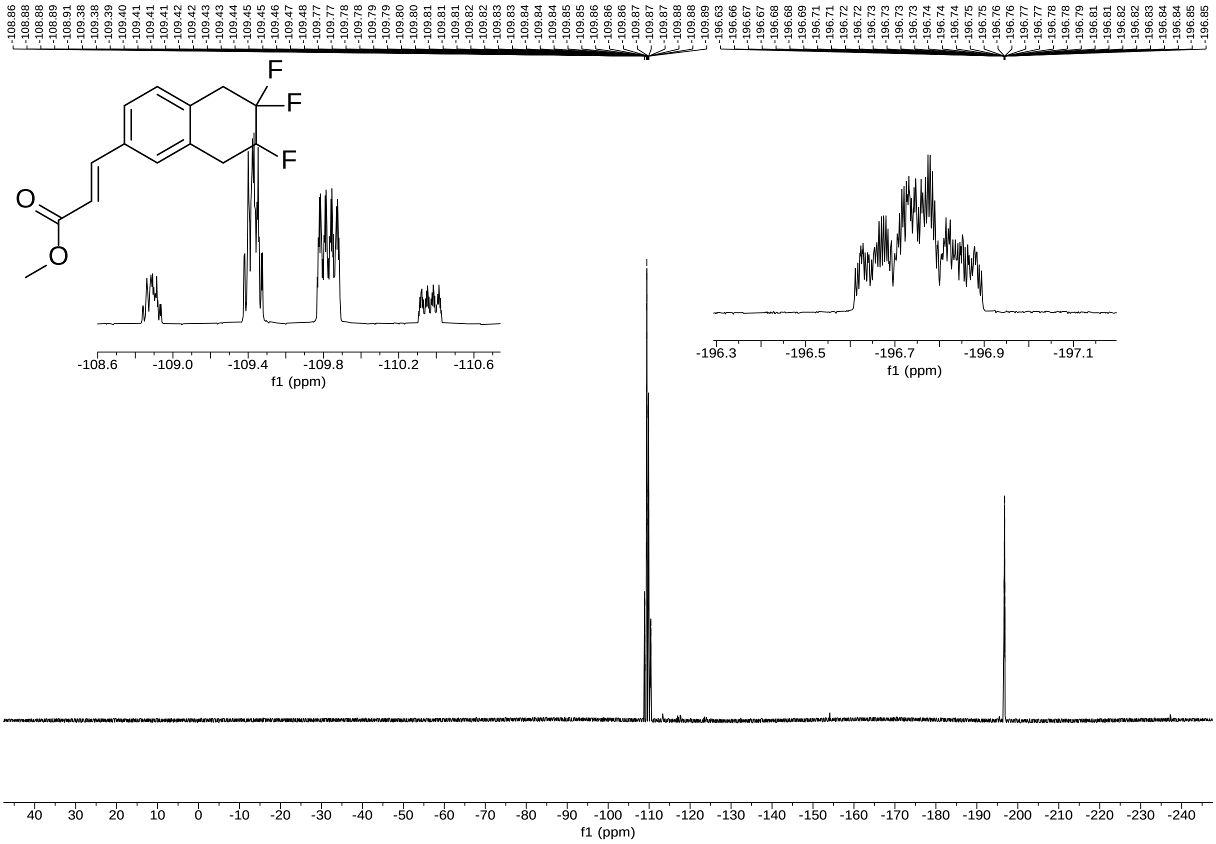

^{19}F{^{1}H} NMR (470 MHz, $CDCl_3$, 299 K)

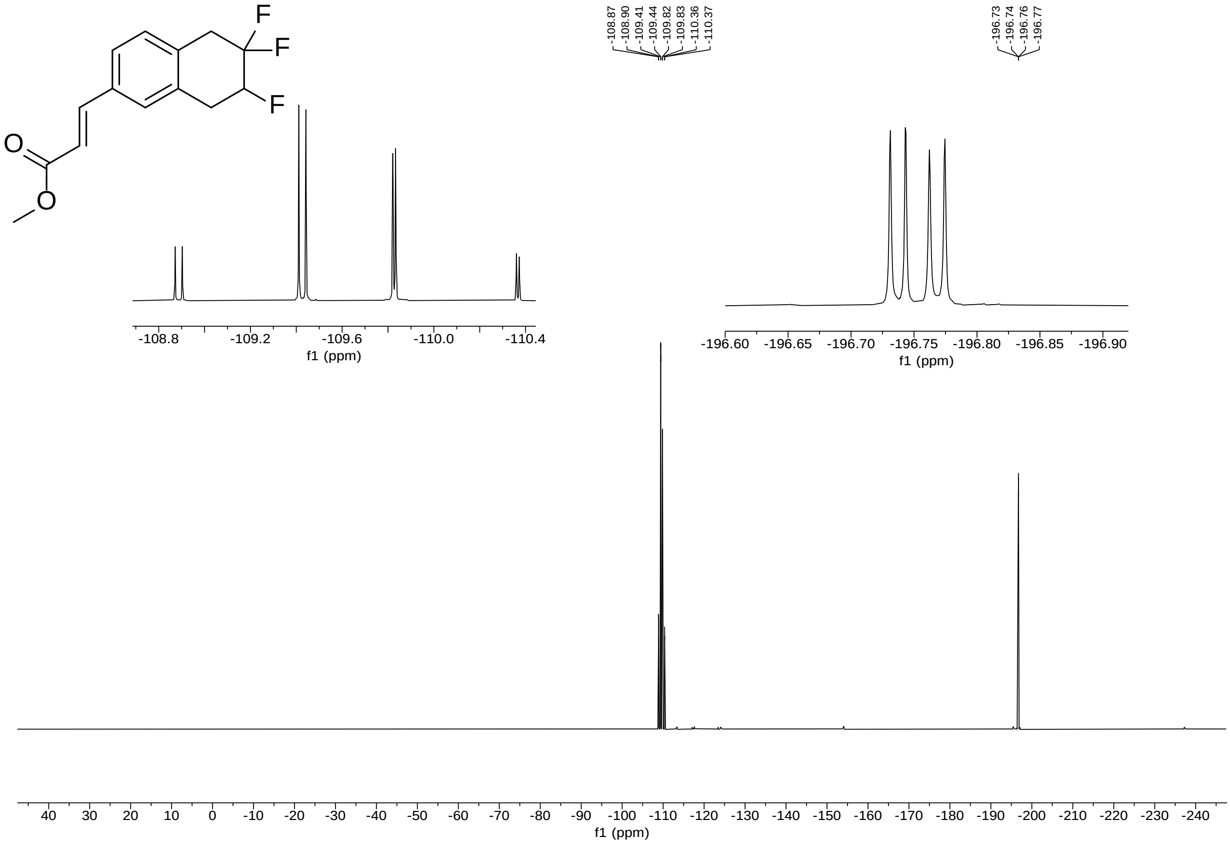

2,2,3-Trifluoro-1-phenyl-1,2,3,4-tetrahydronaphthalene (21)

^{1}H NMR (599 MHz, $CDCl_3$, 299 K)

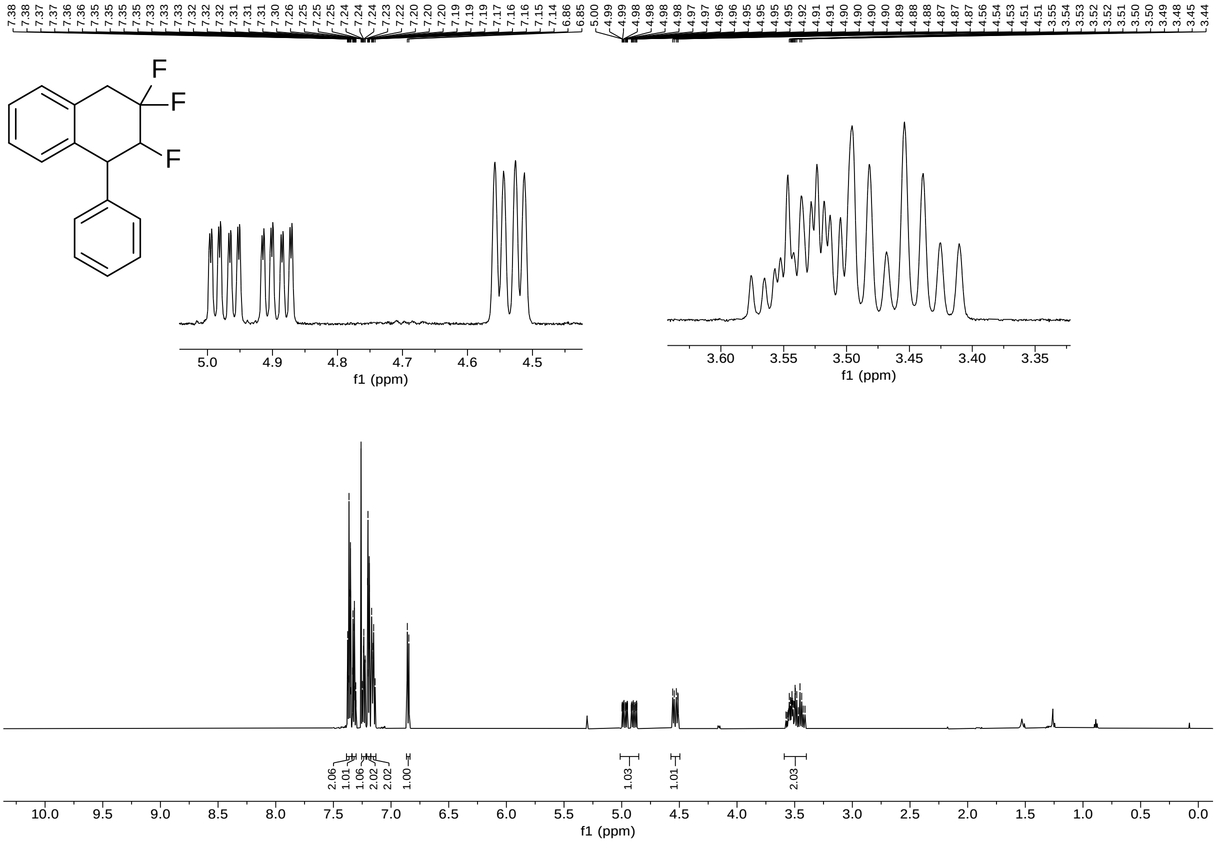

^{13}C NMR (151 MHz, $CDCl_3$, 299 K)

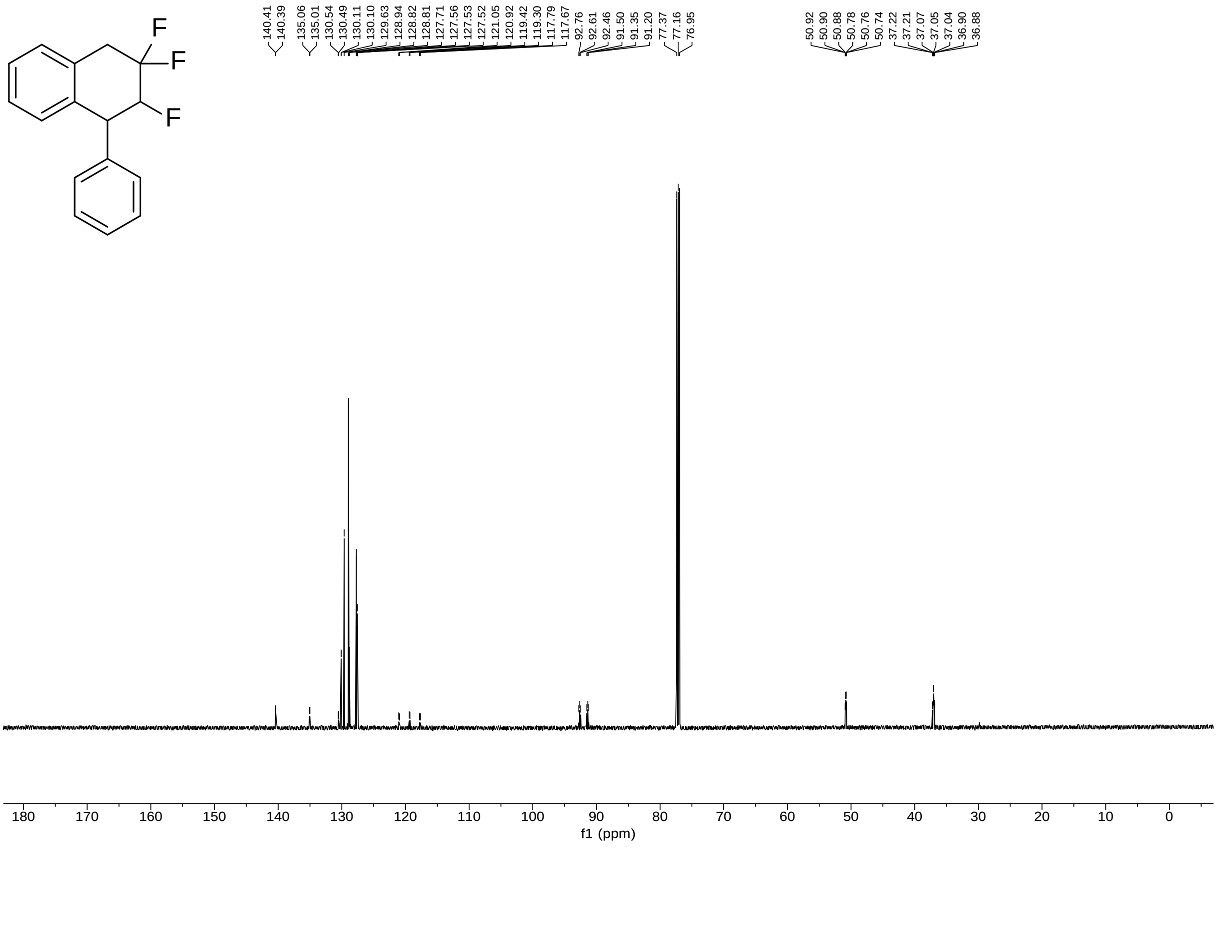

19F NMR (564 MHz, $CDCl_3$, 299 K)

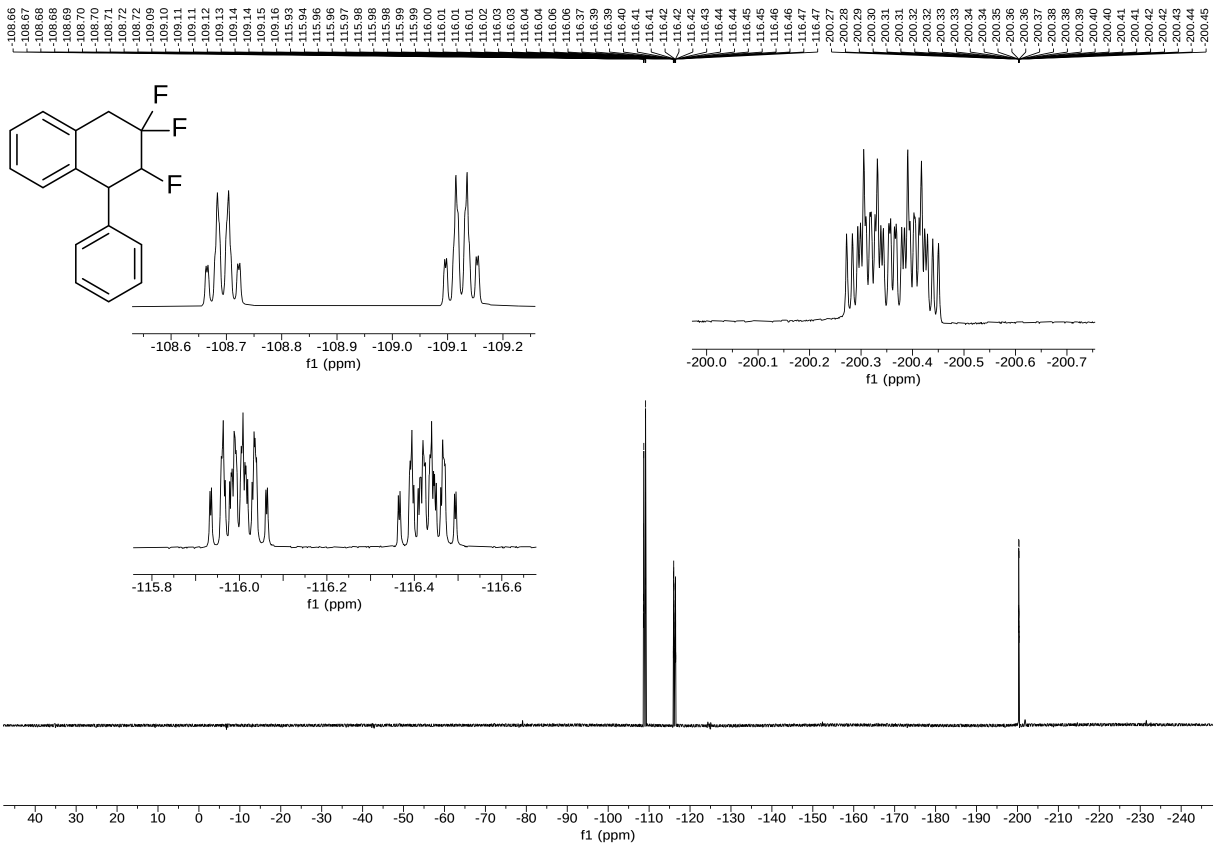

19F{1H} NMR (564 MHz, $CDCl_3$, 299 K)

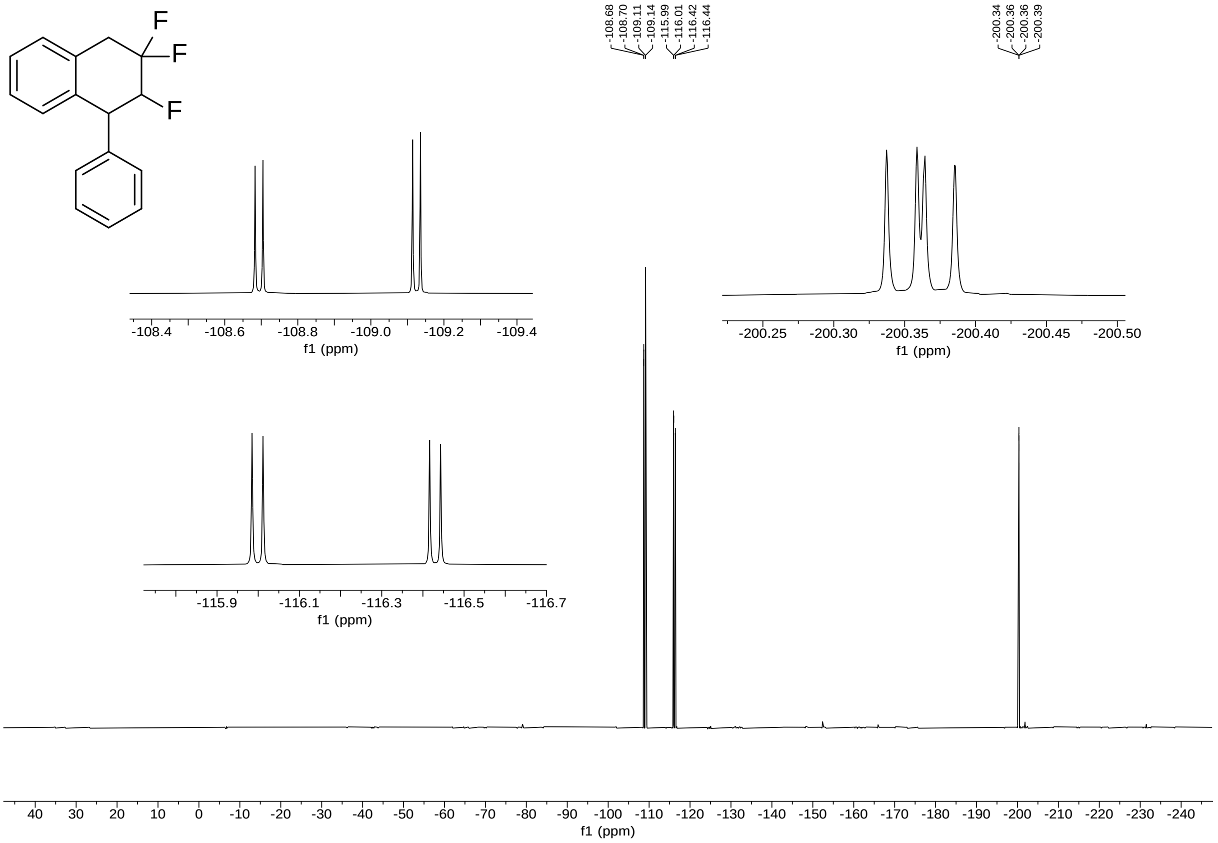

2,2,3-Trifluoro-1,1-dimethyl-1,2,3,4-tetrahydronaphthalene (22)

^{1}H NMR (500 MHz, $CDCl_3$, 299 K)

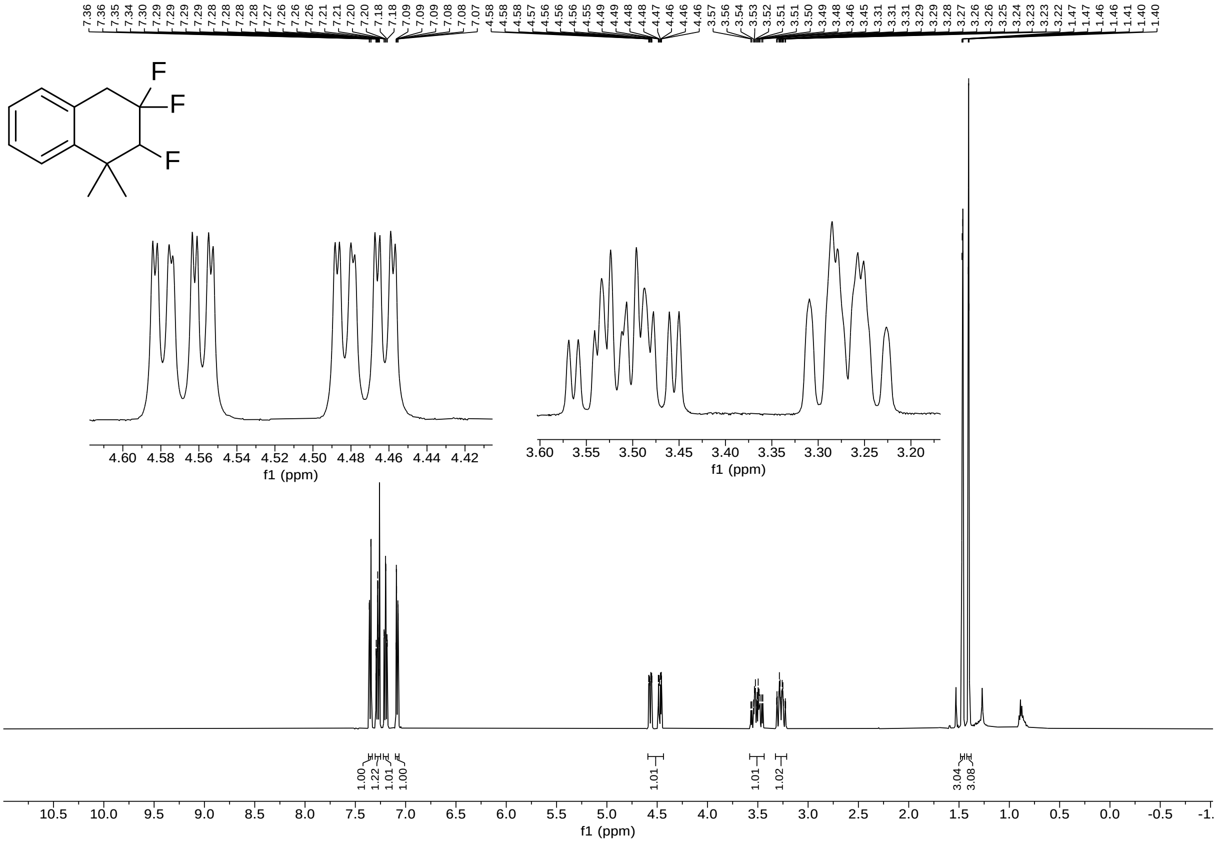

^{13}C NMR (126 MHz, $CDCl_3$, 299 K)

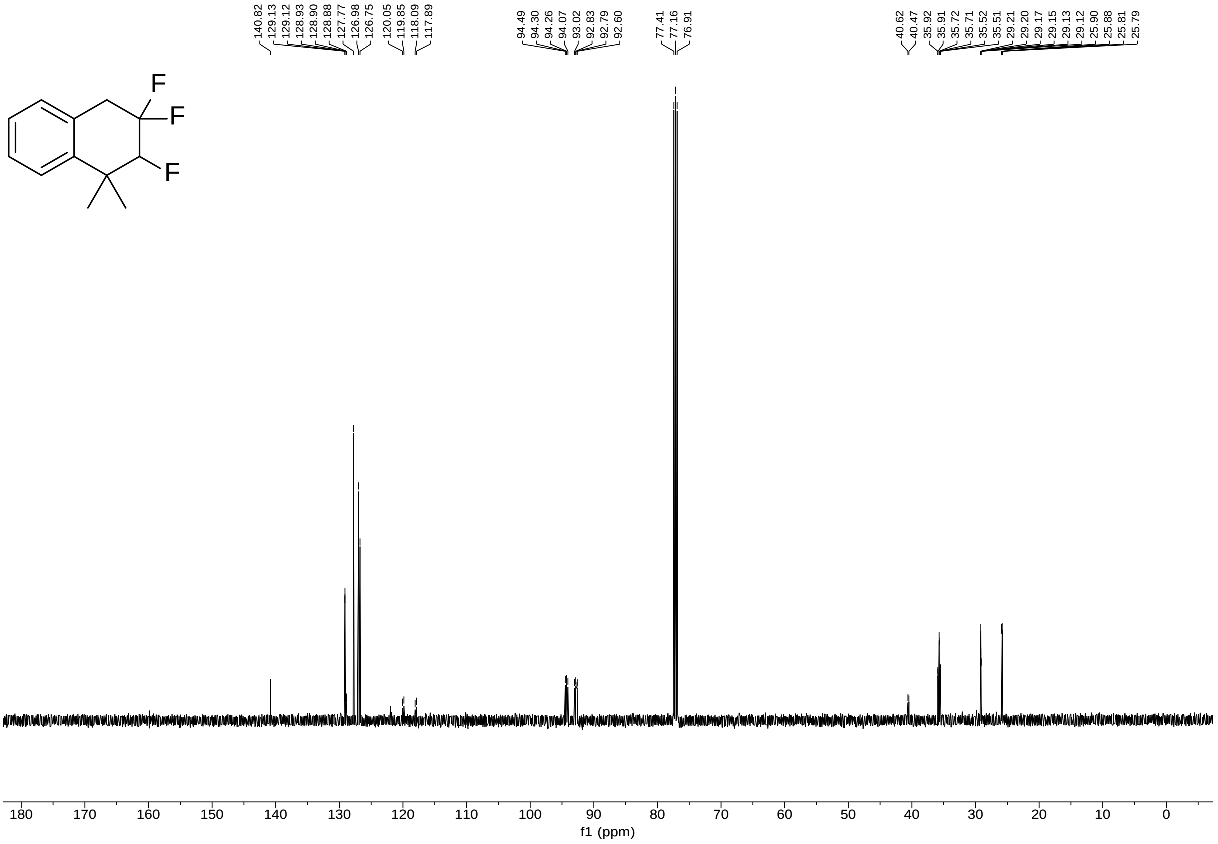

^{19}F NMR (470 MHz, $CDCl_3$, 299 K)

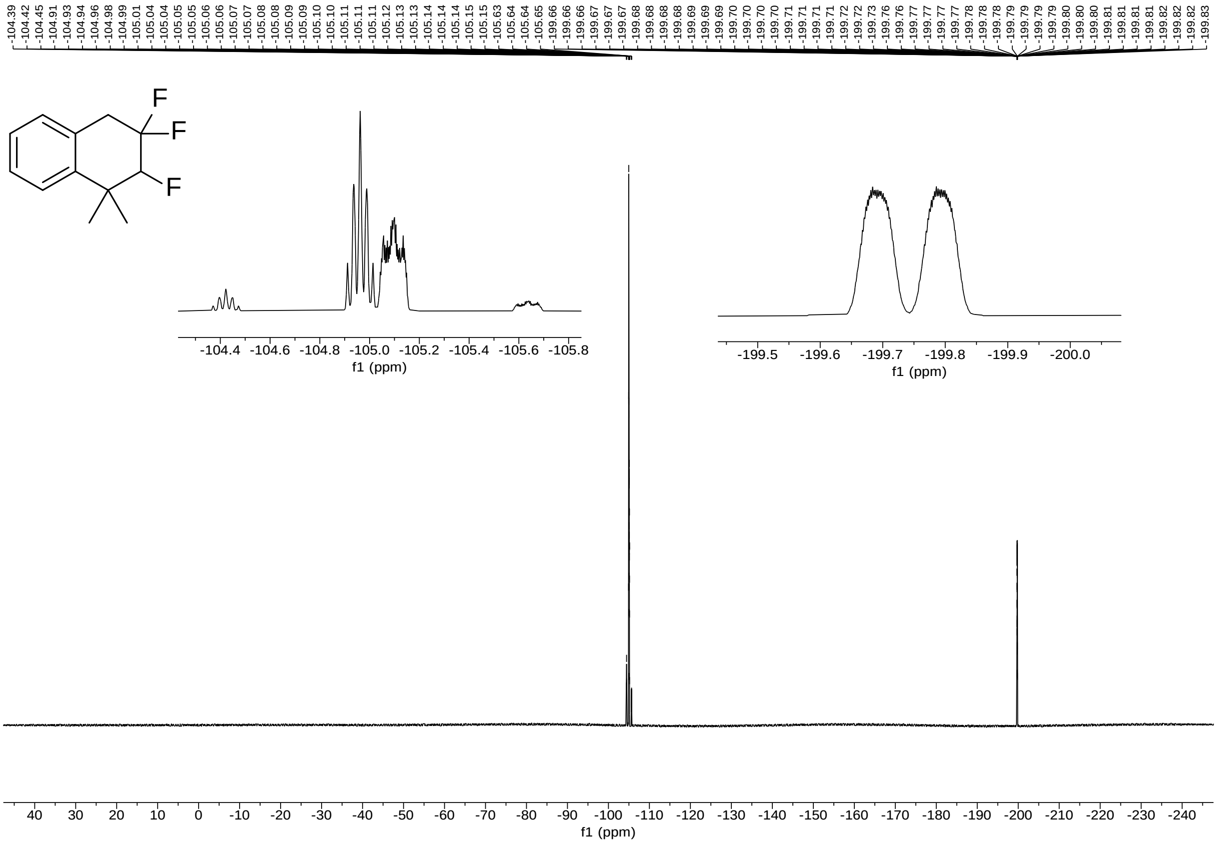

^{19}F{^{1}H} NMR (470 MHz, $CDCl_3$, 299 K)

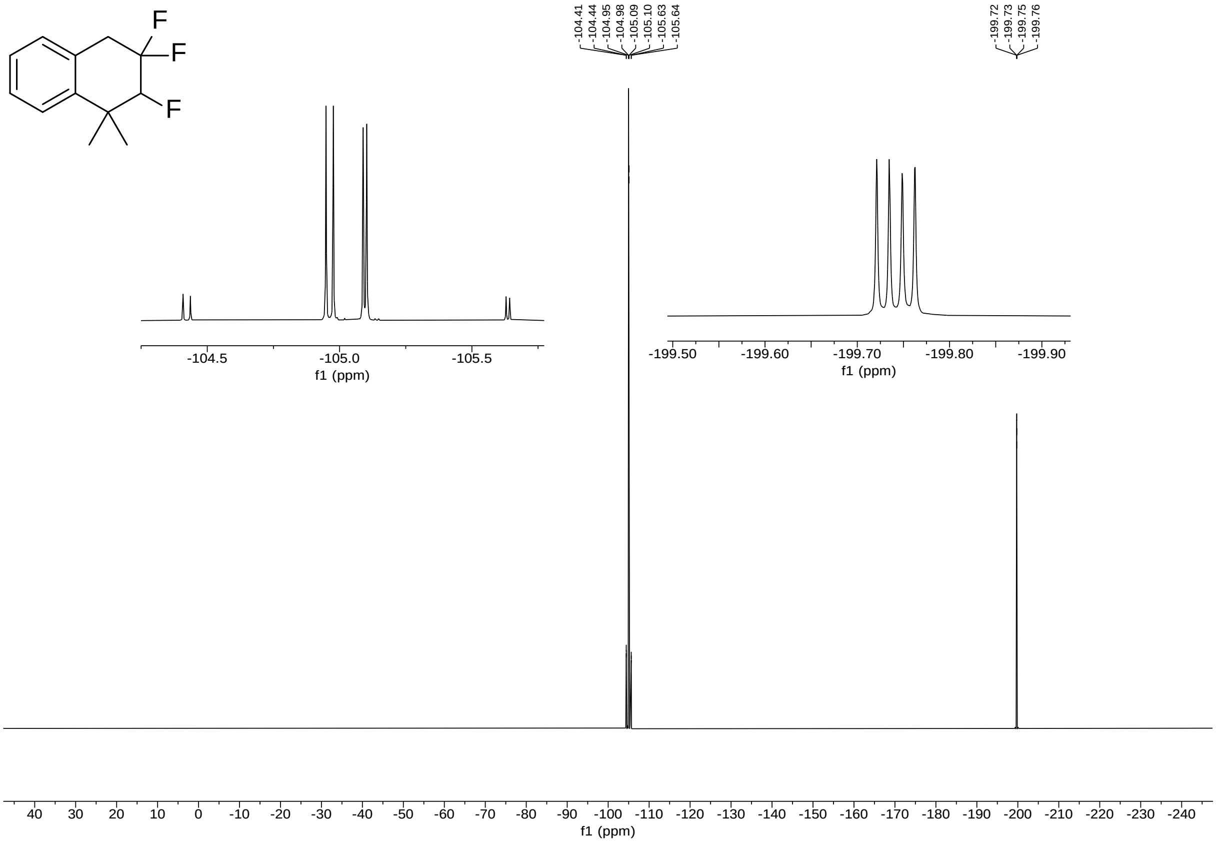

2,2,3,3-Tetrafluoro-1,2,3,4-tetrahydronaphthalene (23)

^{1}H NMR (599 MHz, $CDCl_3$, 299 K)

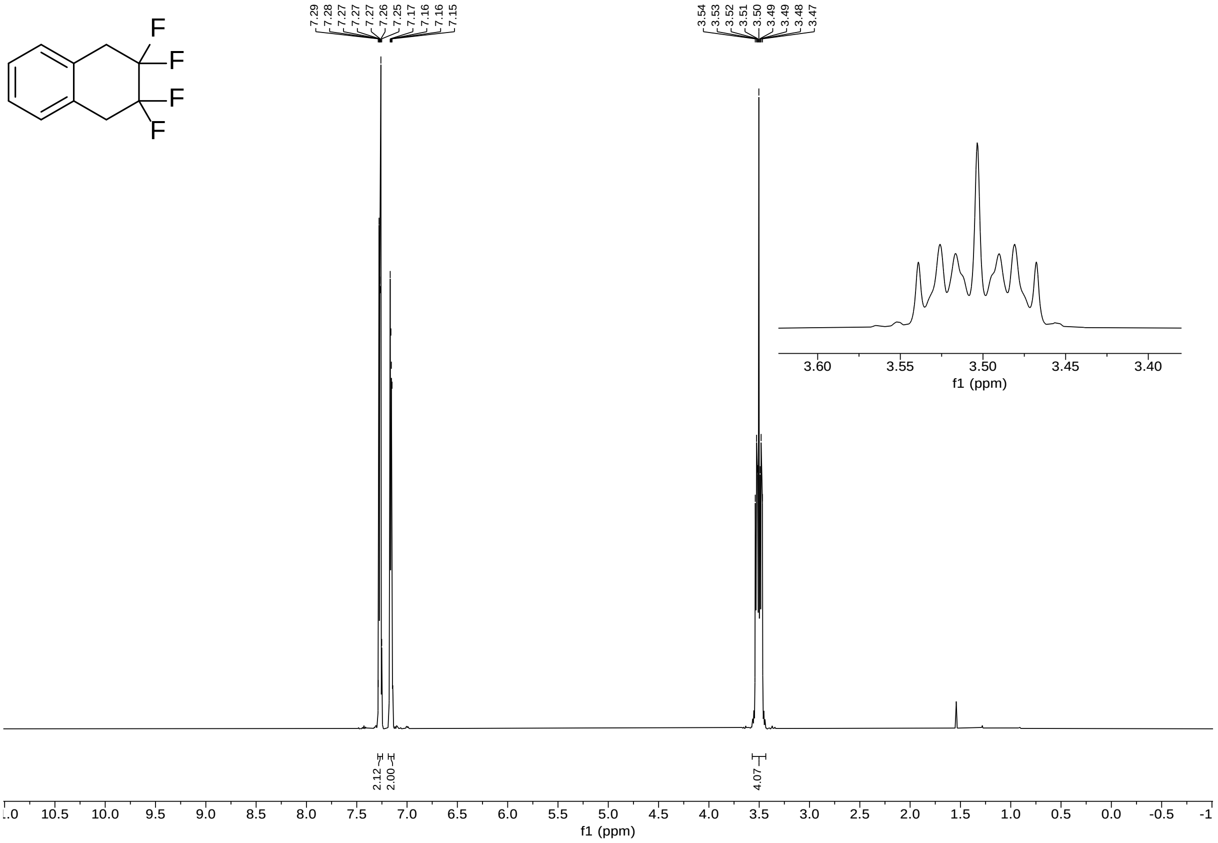

^{13}C NMR (151 MHz, $CDCl_3$, 299 K)

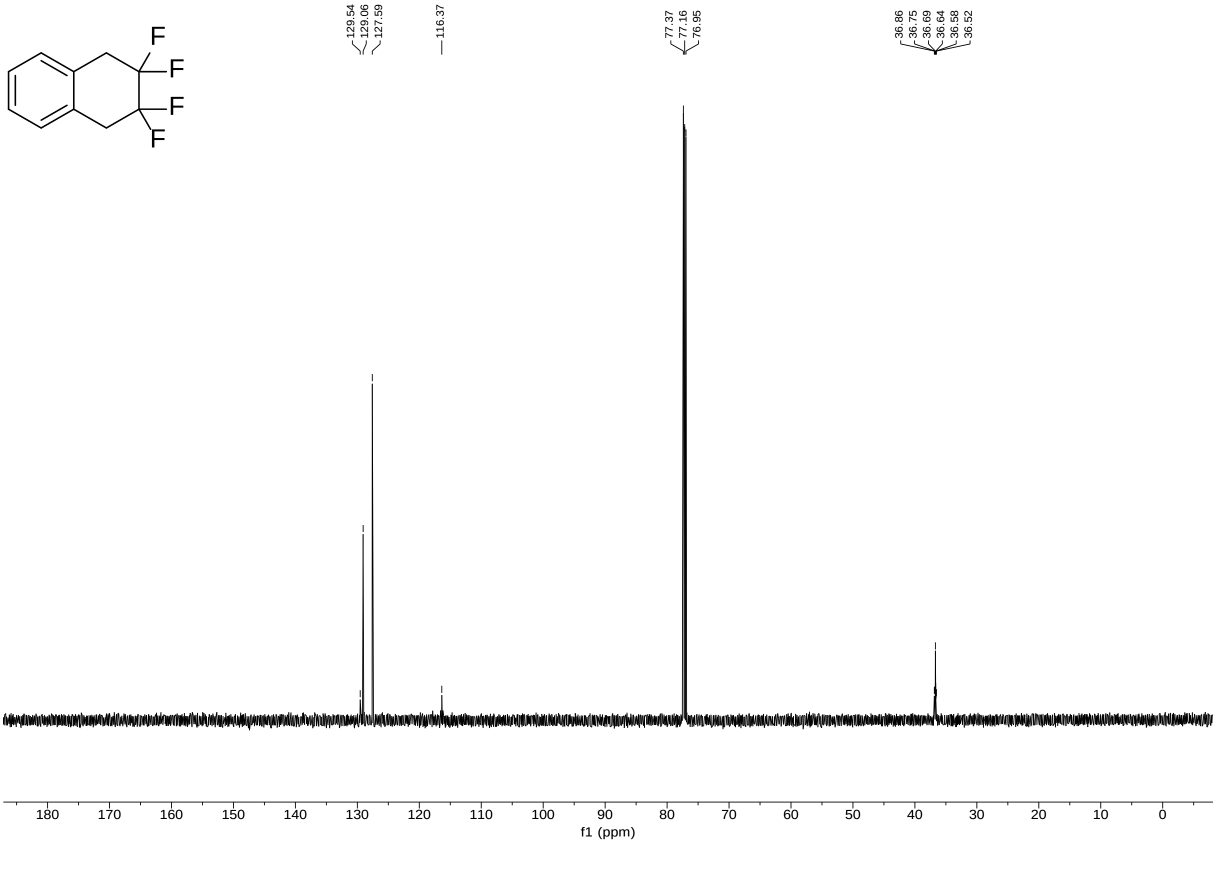

^{19}F NMR (564 MHz, $CDCl_3$, 299 K)

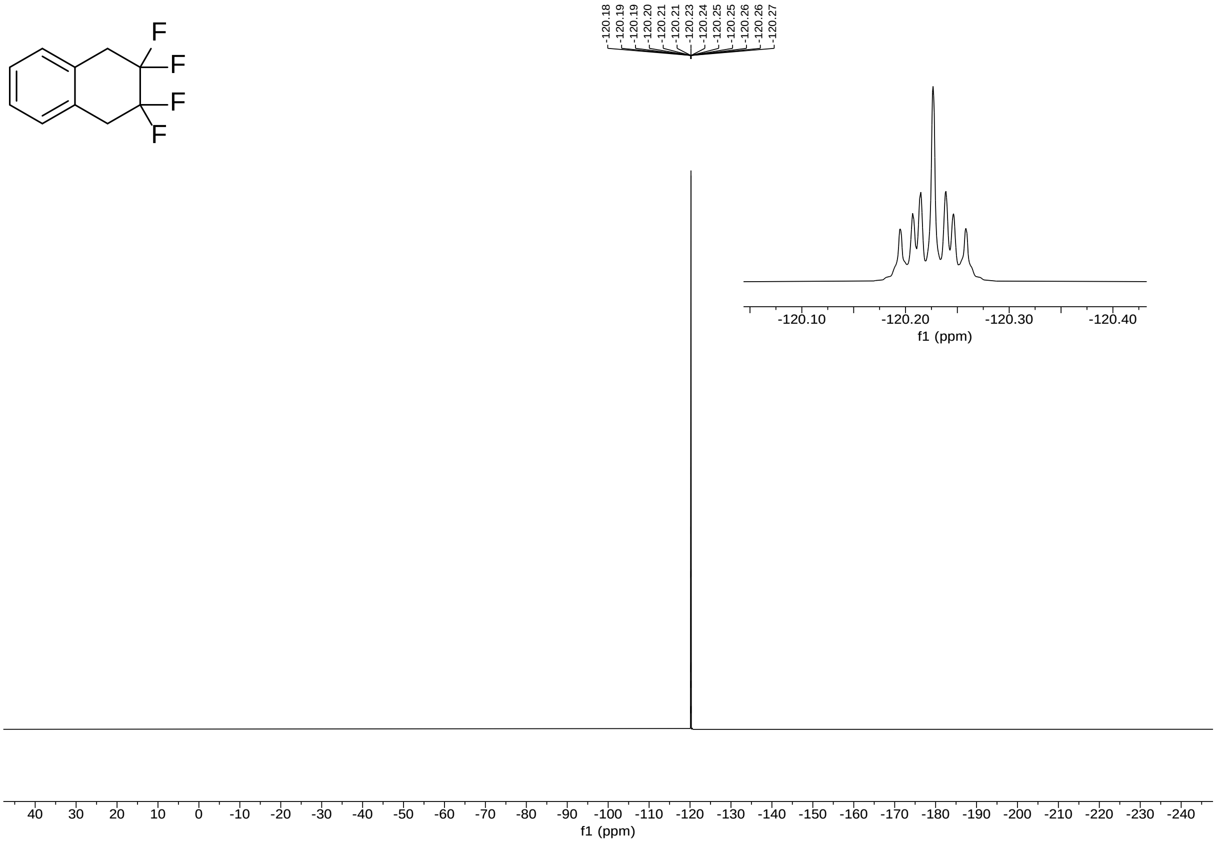

^{19}F{^{1}H} NMR (564 MHz, $CDCl_3$, 299 K)

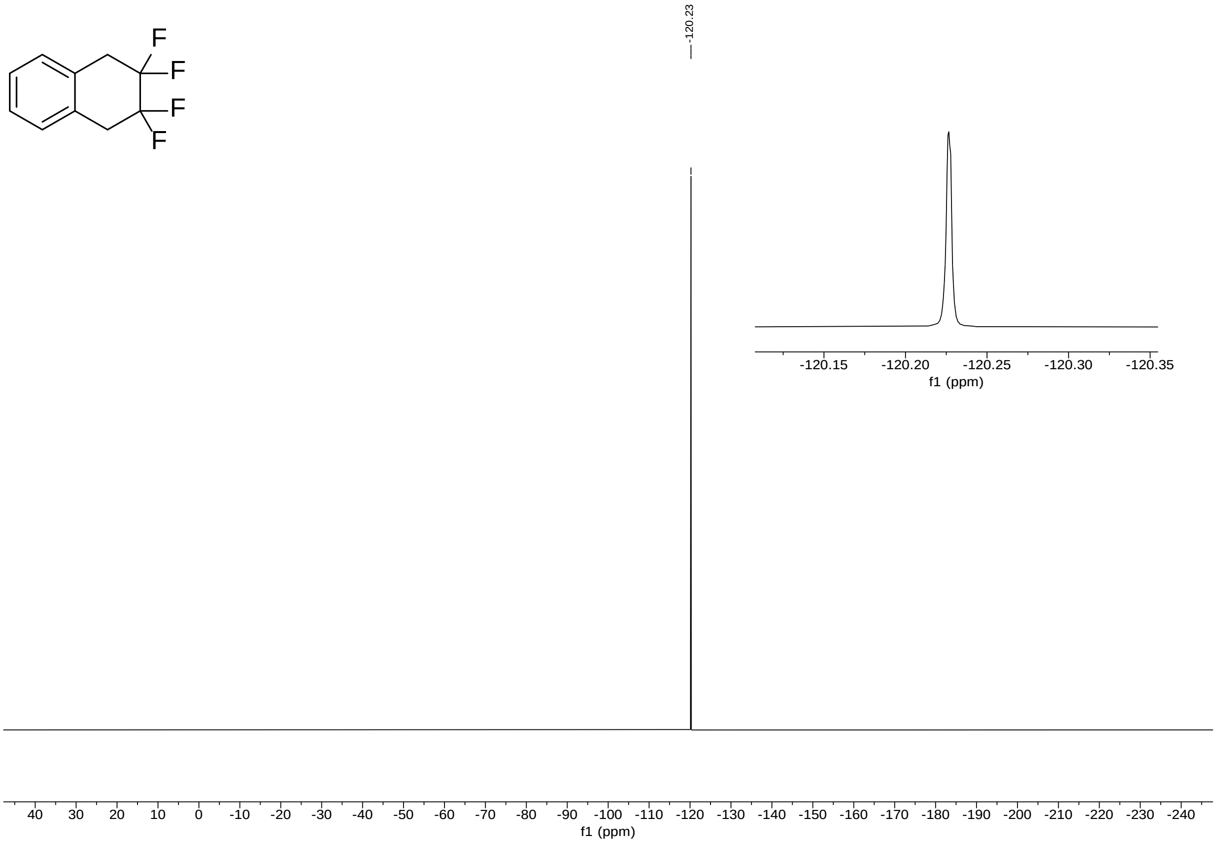

3-Chloro-2,2-difluoro-1,2,3,4-tetrahydronaphthalene (24)

^{1}H NMR (599 MHz, $CDCl_3$, 299 K)

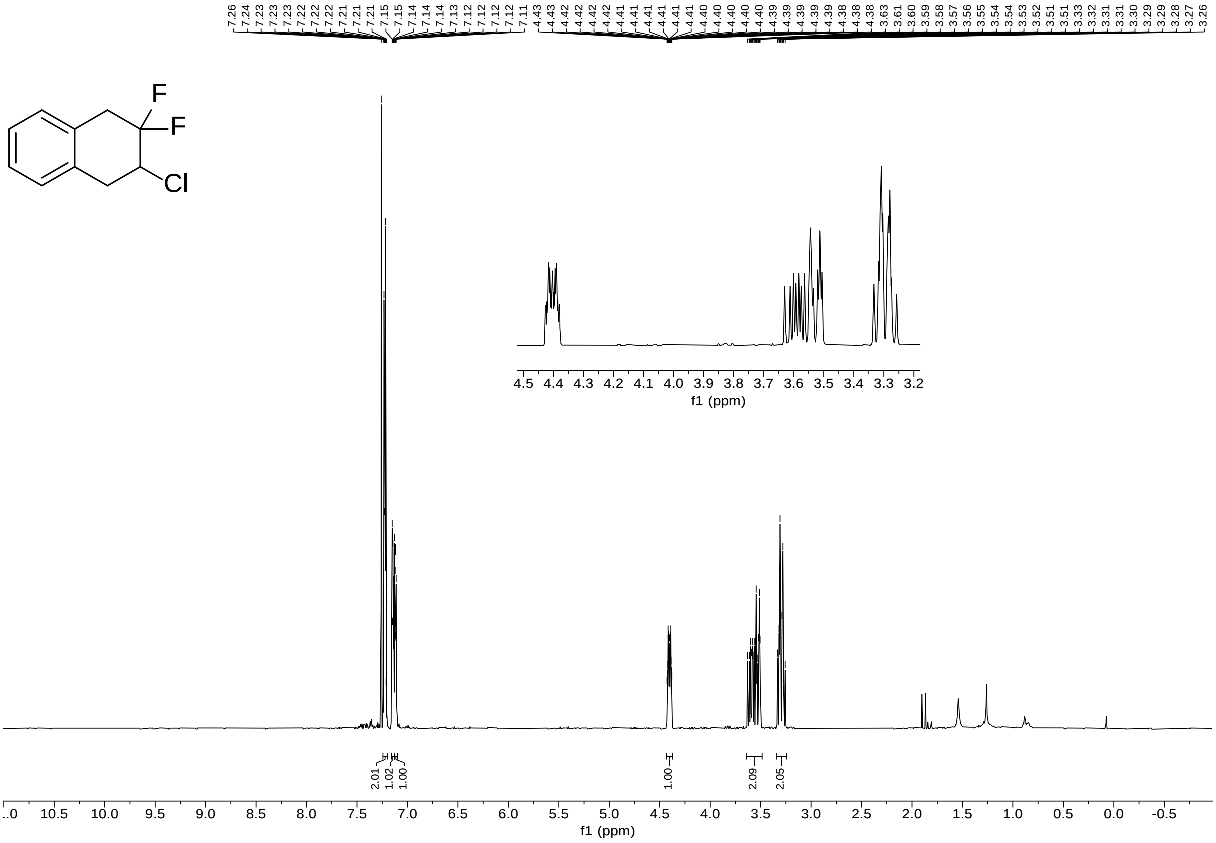

^{13}C NMR (151 MHz, $CDCl_3$, 299 K)

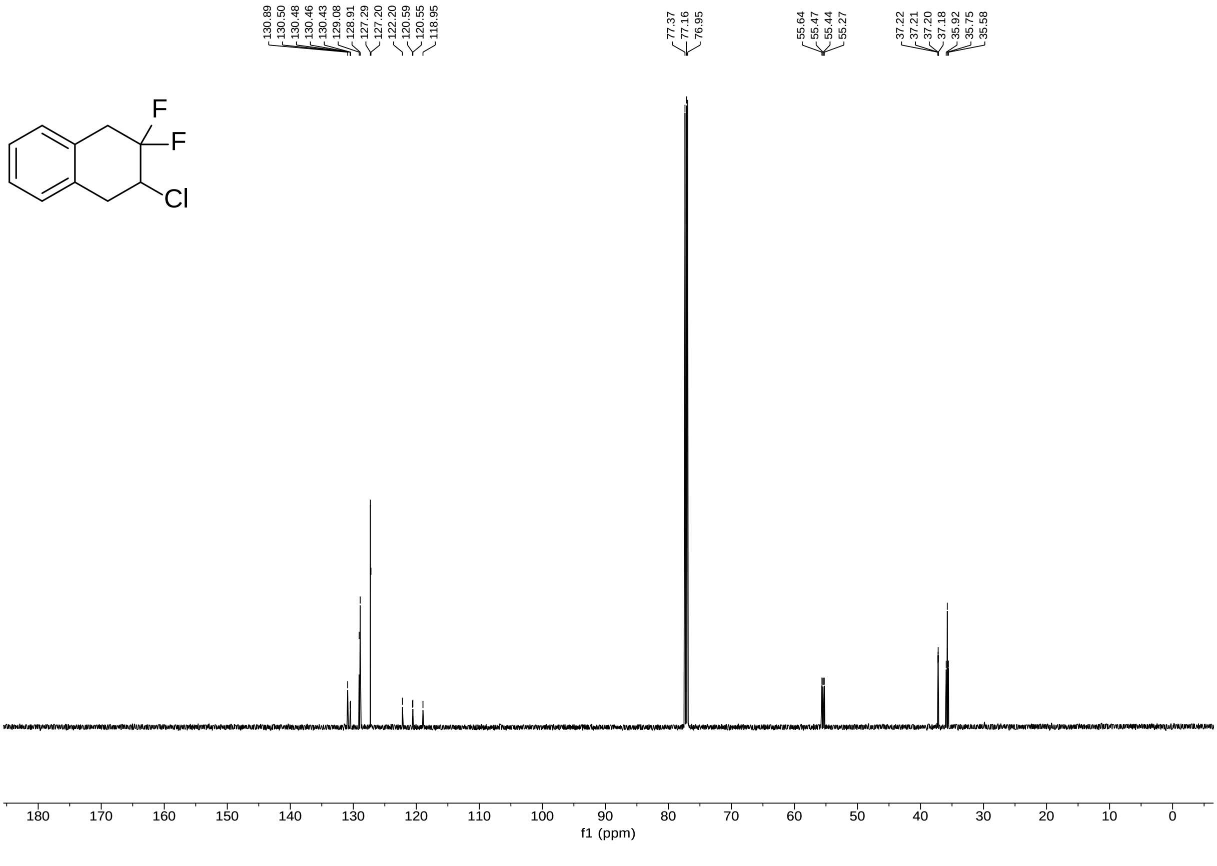

^{19}F NMR (564 MHz, $CDCl_3$, 299 K)

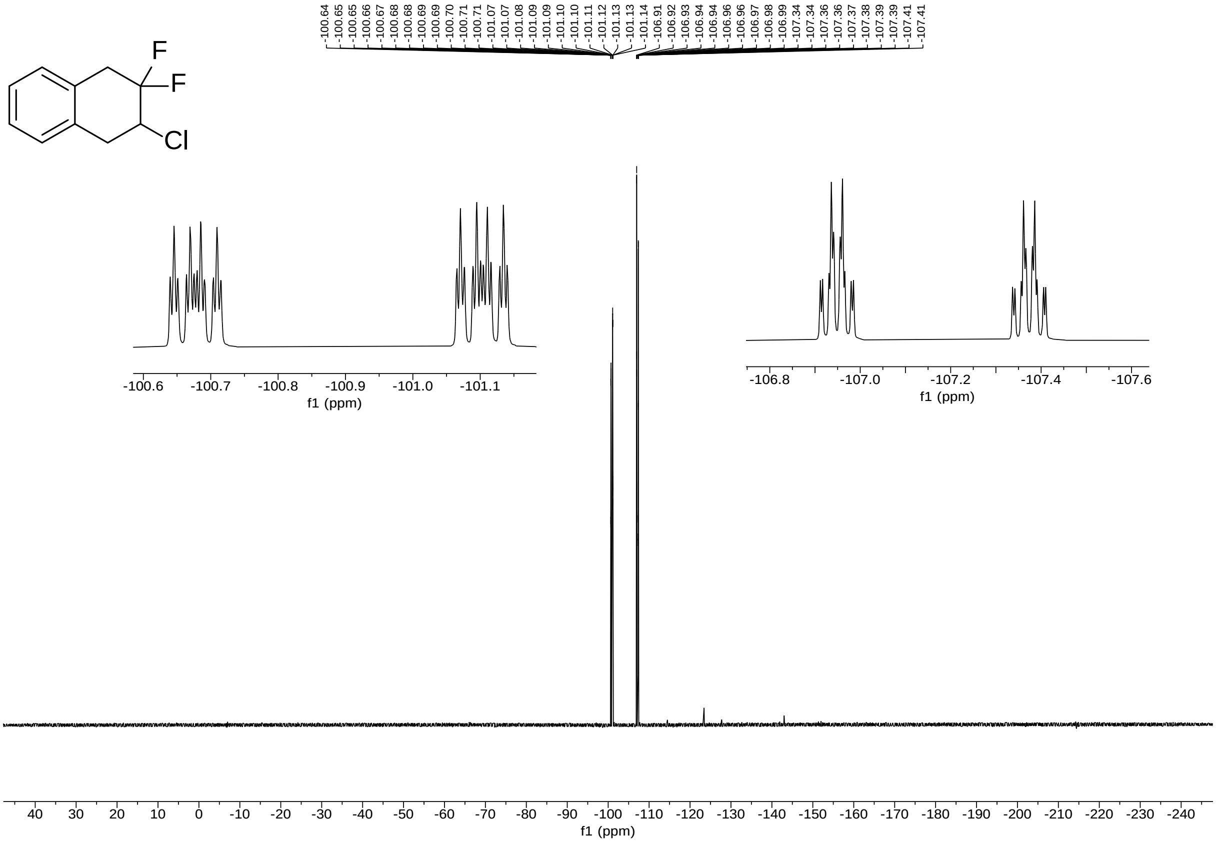

^{19}F{^{1}H} NMR (564 MHz, $CDCl_3$, 299 K)

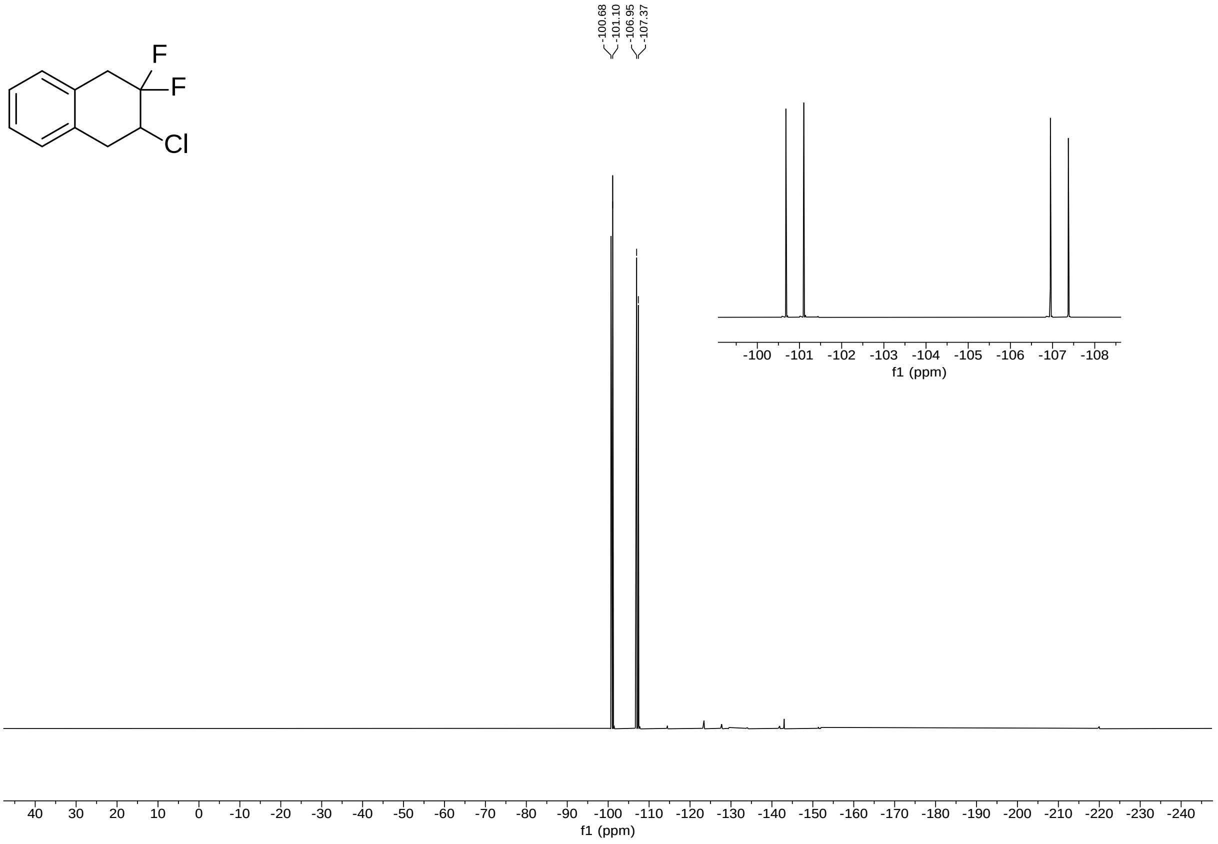

Methyl (*S*)-2,3,3,-trifluoro-1,2,3,4-tetrahydronaphthalene-2-carboxylate (28)

^{1}H NMR (599 MHz, $CDCl_3$, 299 K)

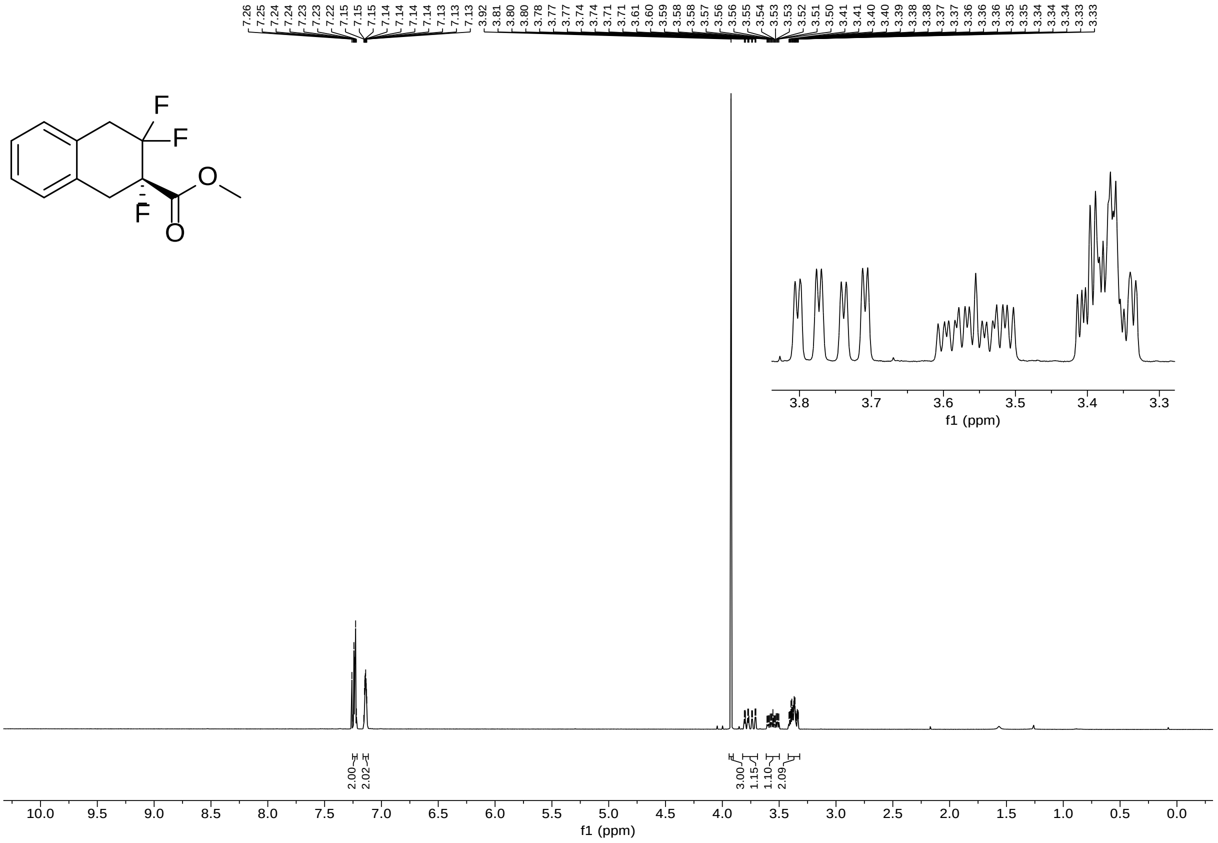

^{13}C NMR (151 MHz, $CDCl_3$, 299 K)

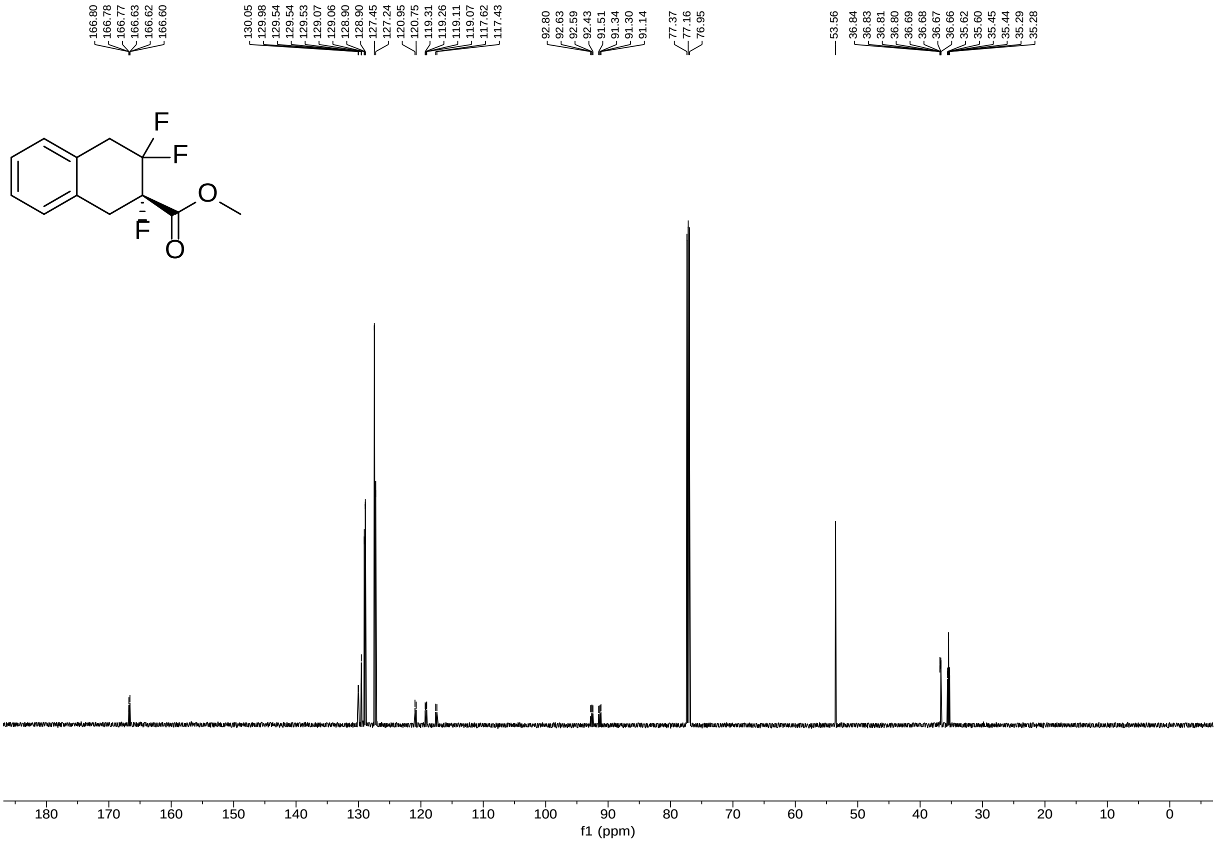

^{19}F NMR (564 MHz, $CDCl_3$, 299 K)

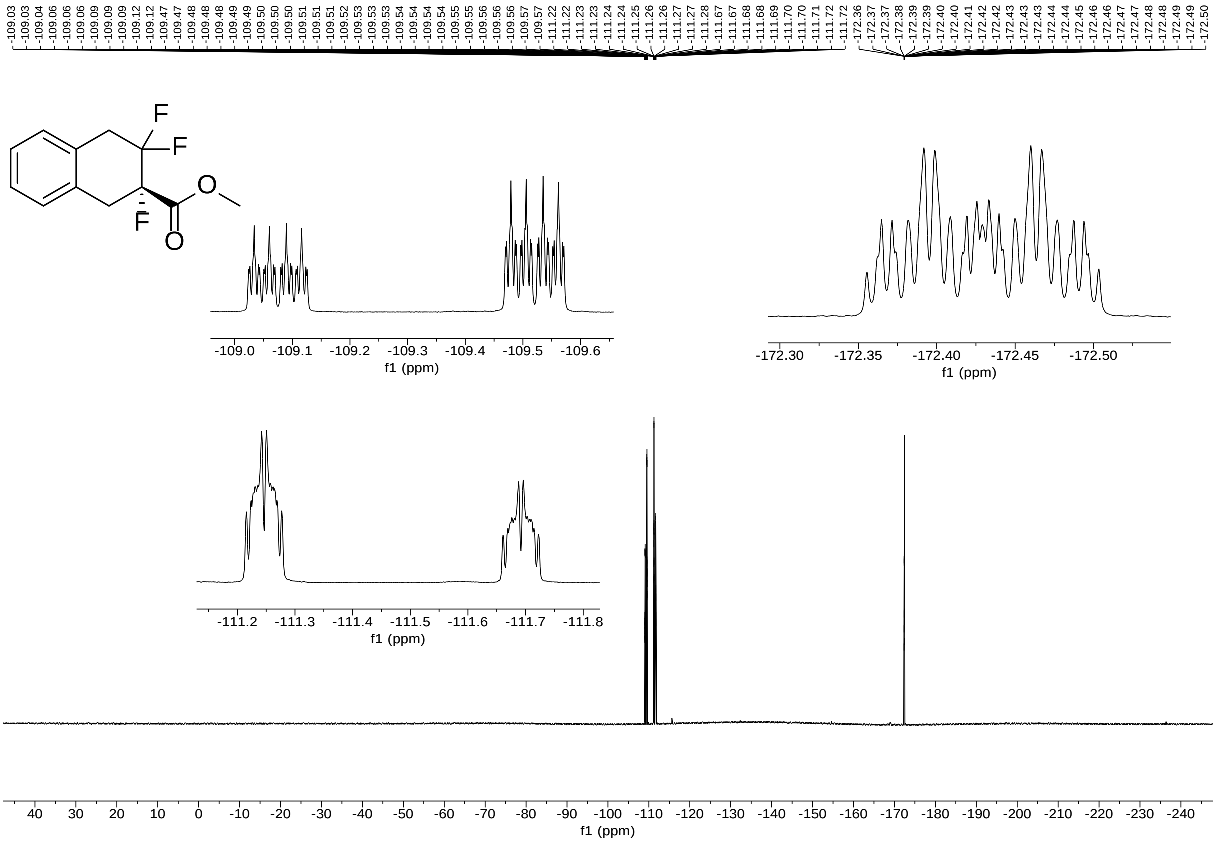

^{19}F{^{1}H} NMR (564 MHz, $CDCl_3$, 299 K)

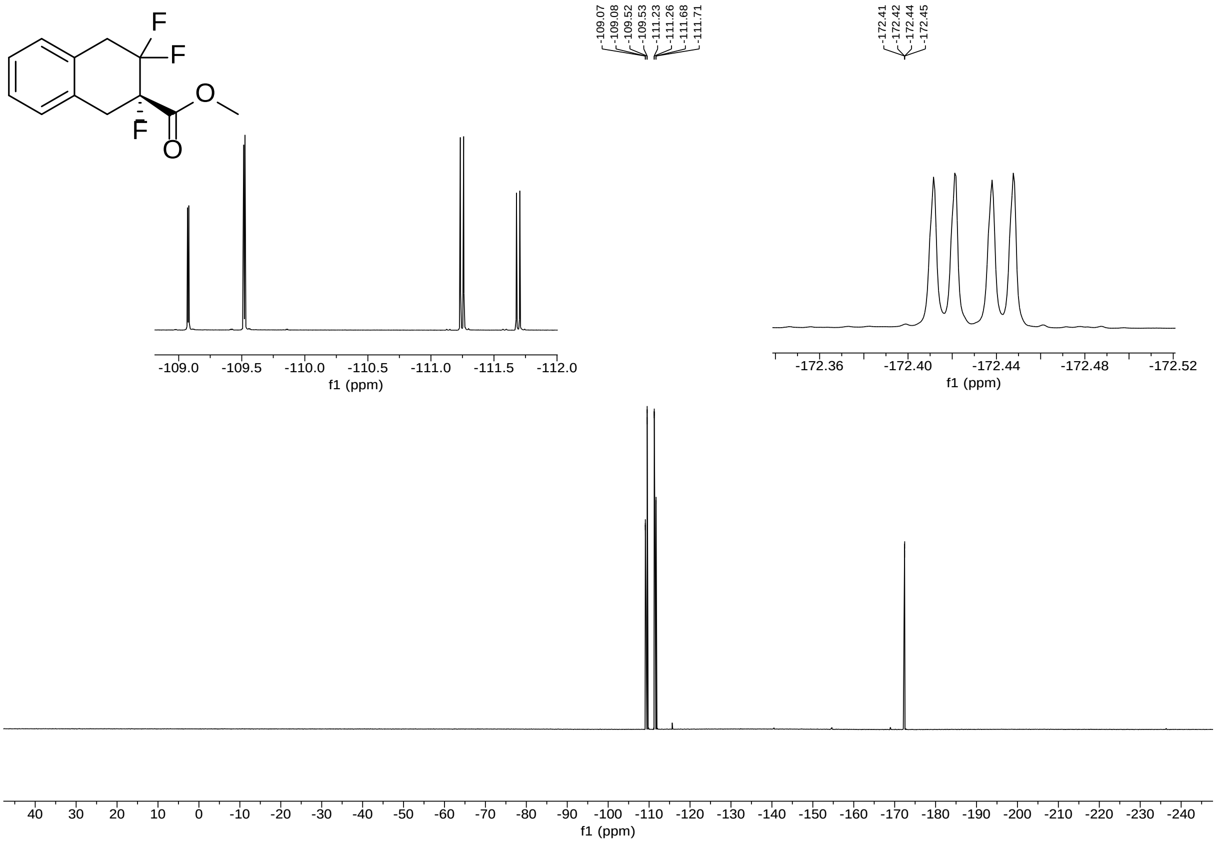

Ethyl (*S*)-2,3,3,-trifluoro-1,2,3,4-tetrahydronaphthalene-2-carboxylate (29)

^{1}H NMR (500 MHz, $CDCl_3$, 299 K)

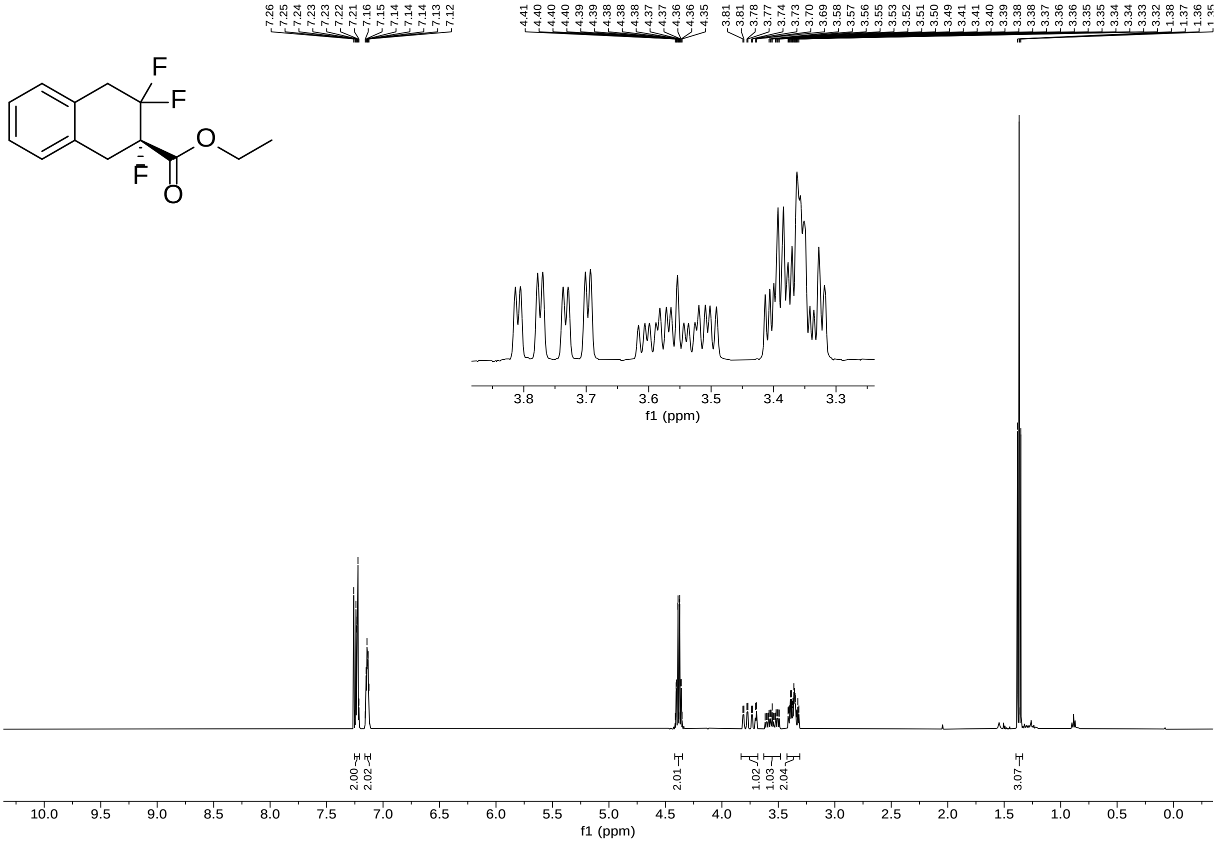

^{13}C NMR (126 MHz, $CDCl_3$, 299 K)

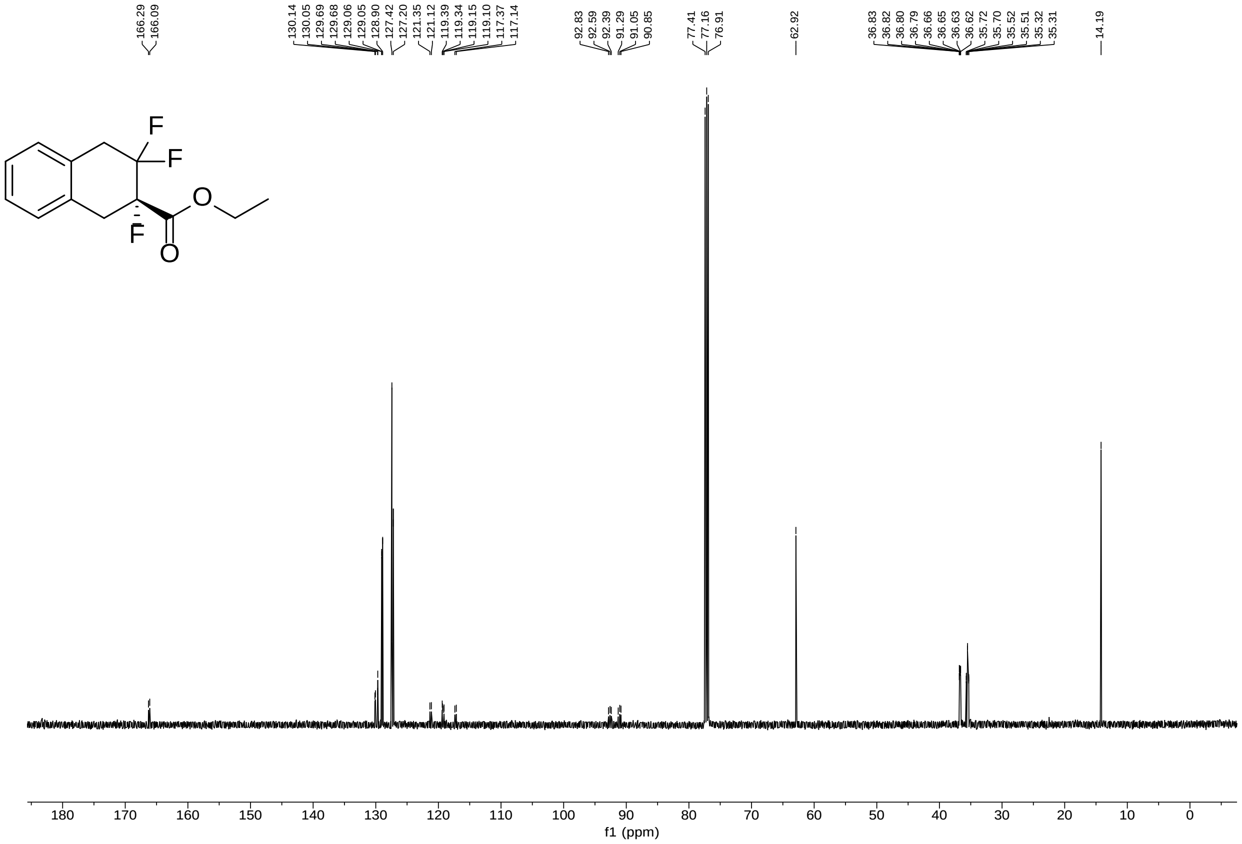

^{19}F NMR (470 MHz, $CDCl_3$, 299 K)

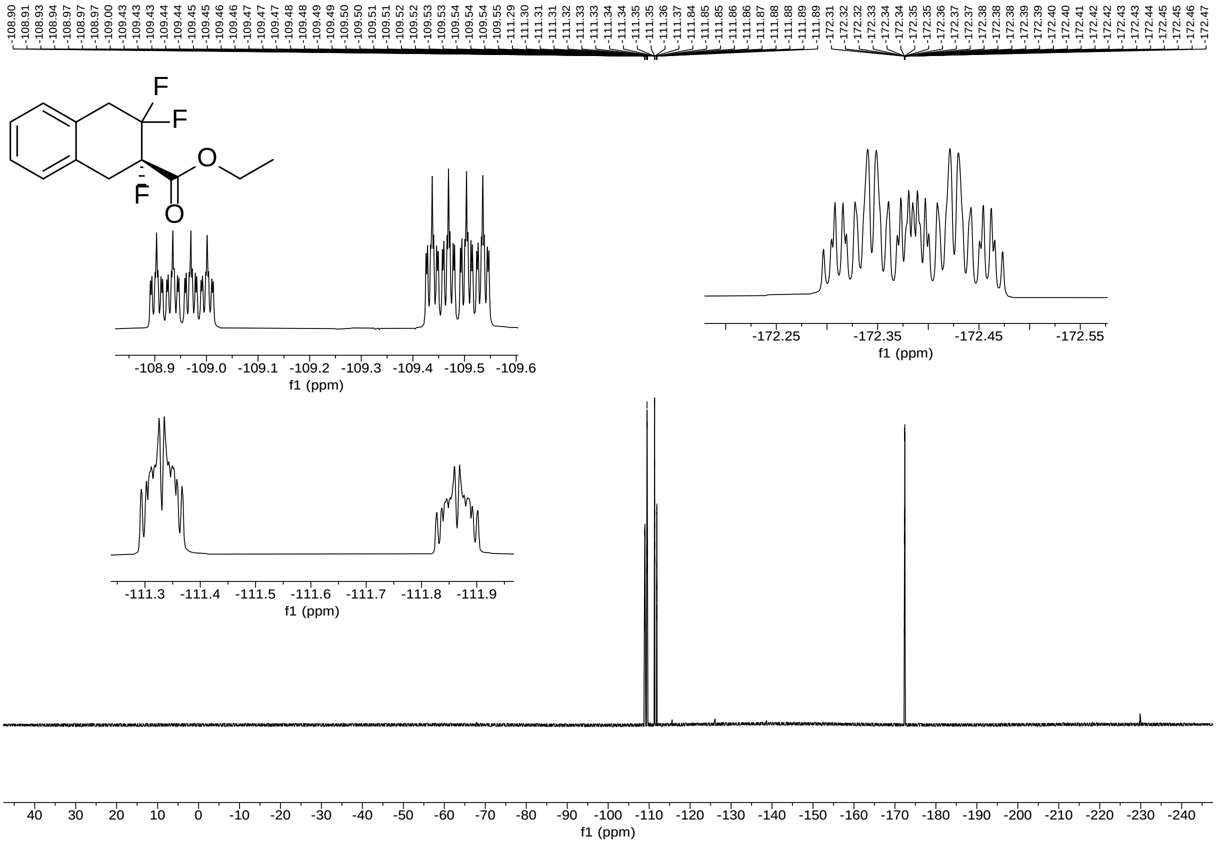

^{19}F{^{1}H} NMR (470 MHz, $CDCl_3$, 299 K)

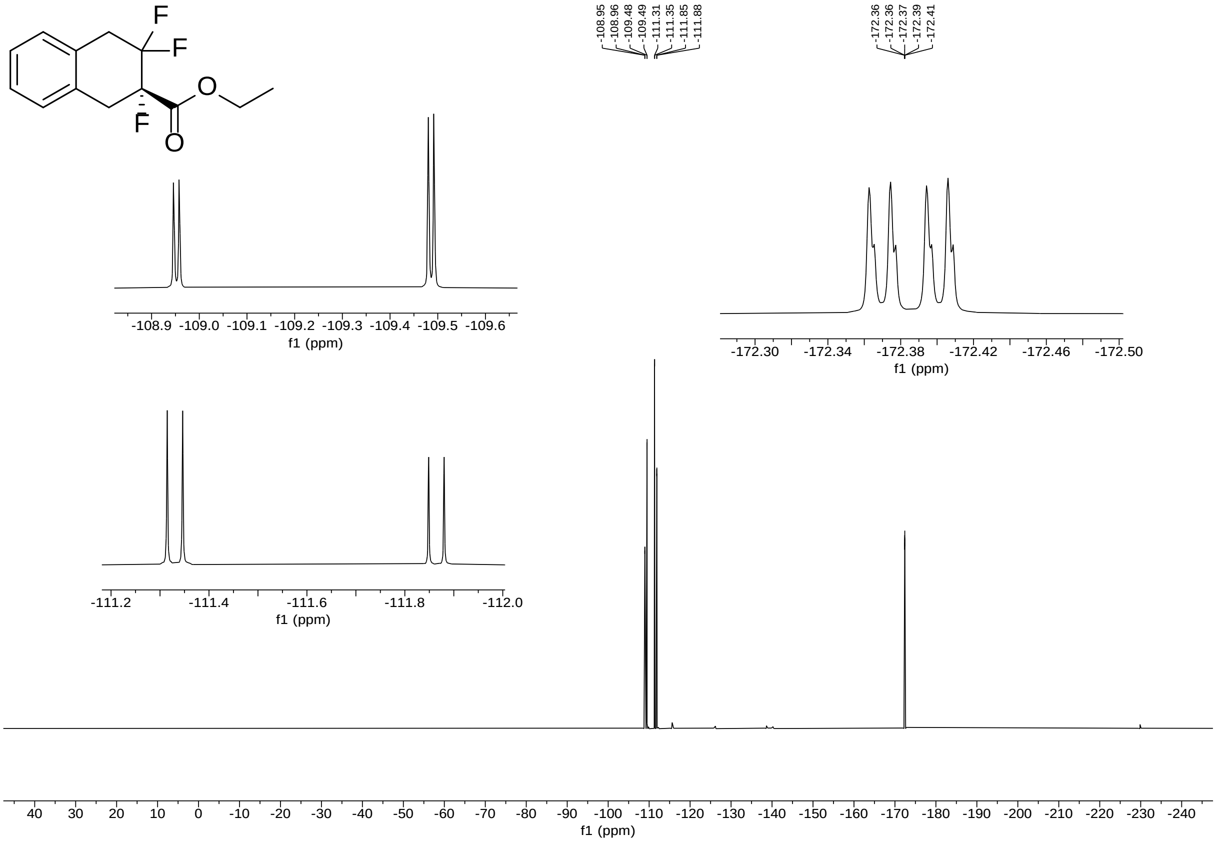

Ethyl (*S*)-7-bromo-2,3,3,-trifluoro-1,2,3,4-tetrahydronaphthalene-2-carboxylate (30)

^{1}H NMR (599 MHz, $CDCl_3$, 299 K)

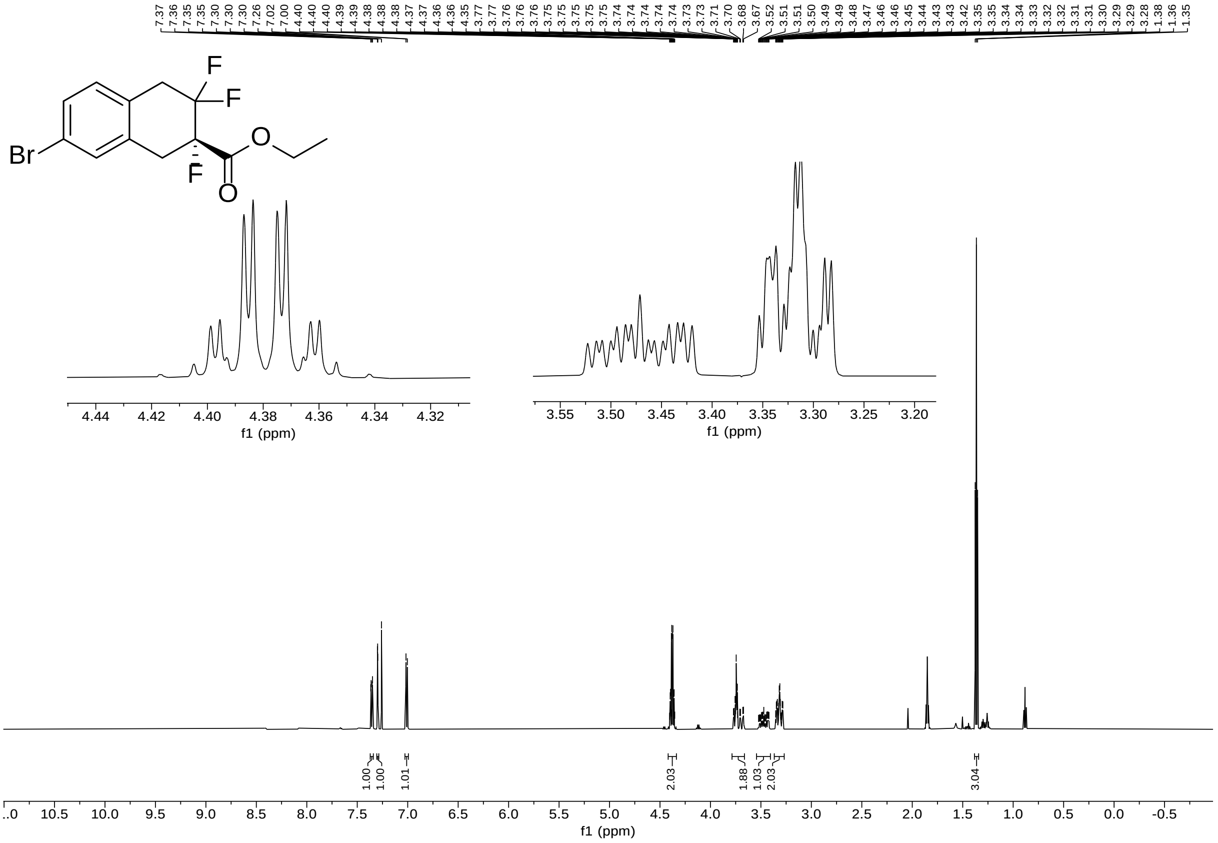

^{13}C NMR (151 MHz, $CDCl_3$, 299 K)

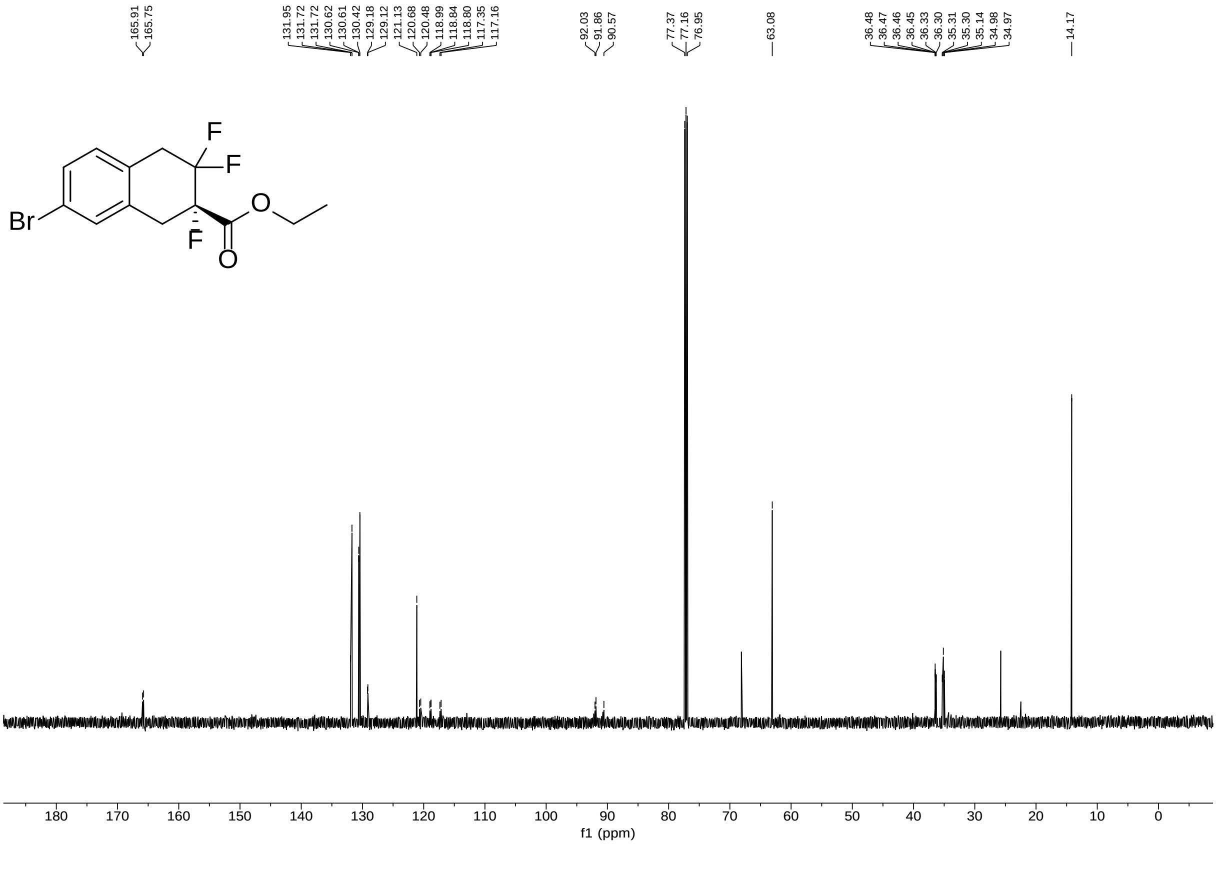

^{19}F NMR (564 MHz, $CDCl_3$, 299 K)

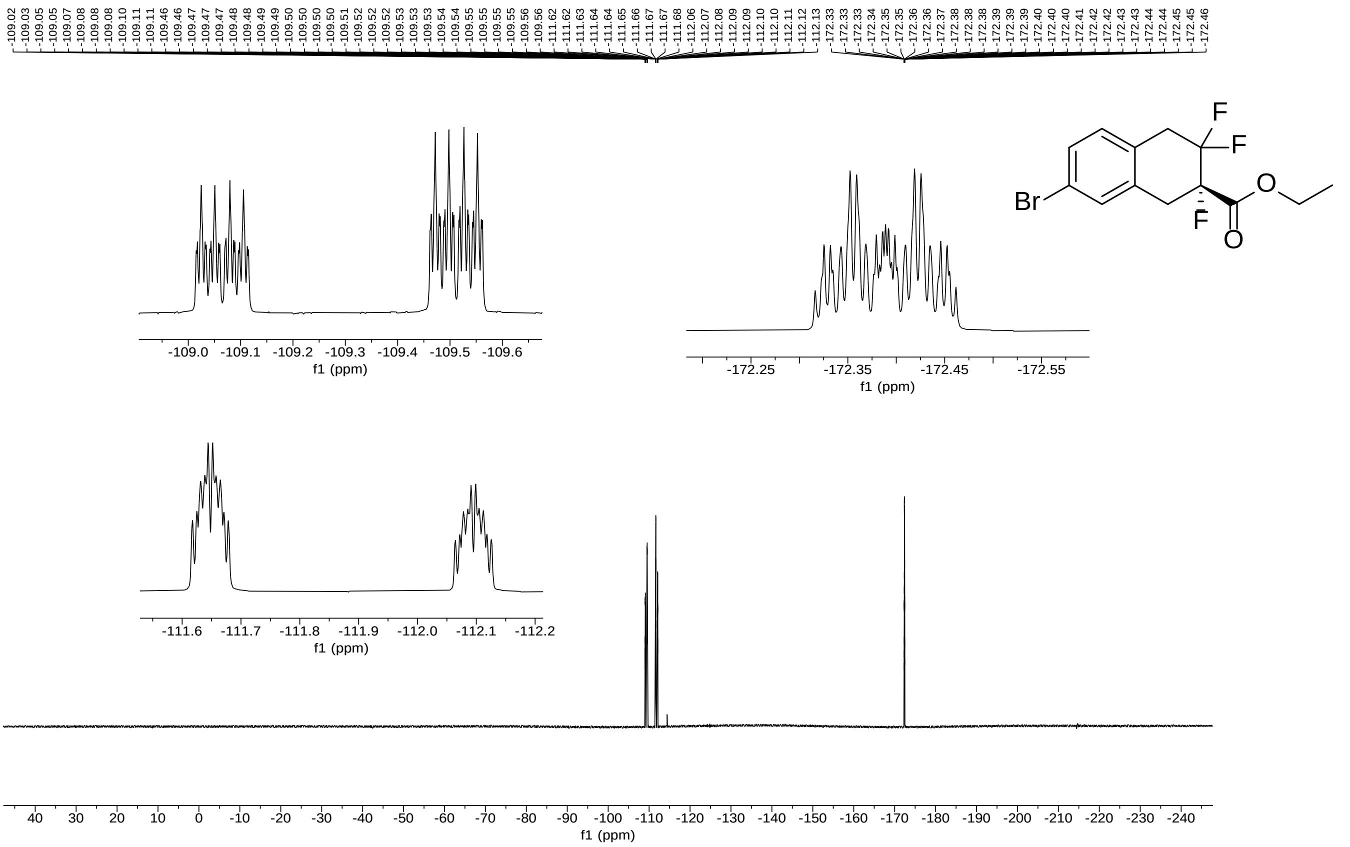

^{19}F{^{1}H} NMR (564 MHz, $CDCl_3$, 299 K)

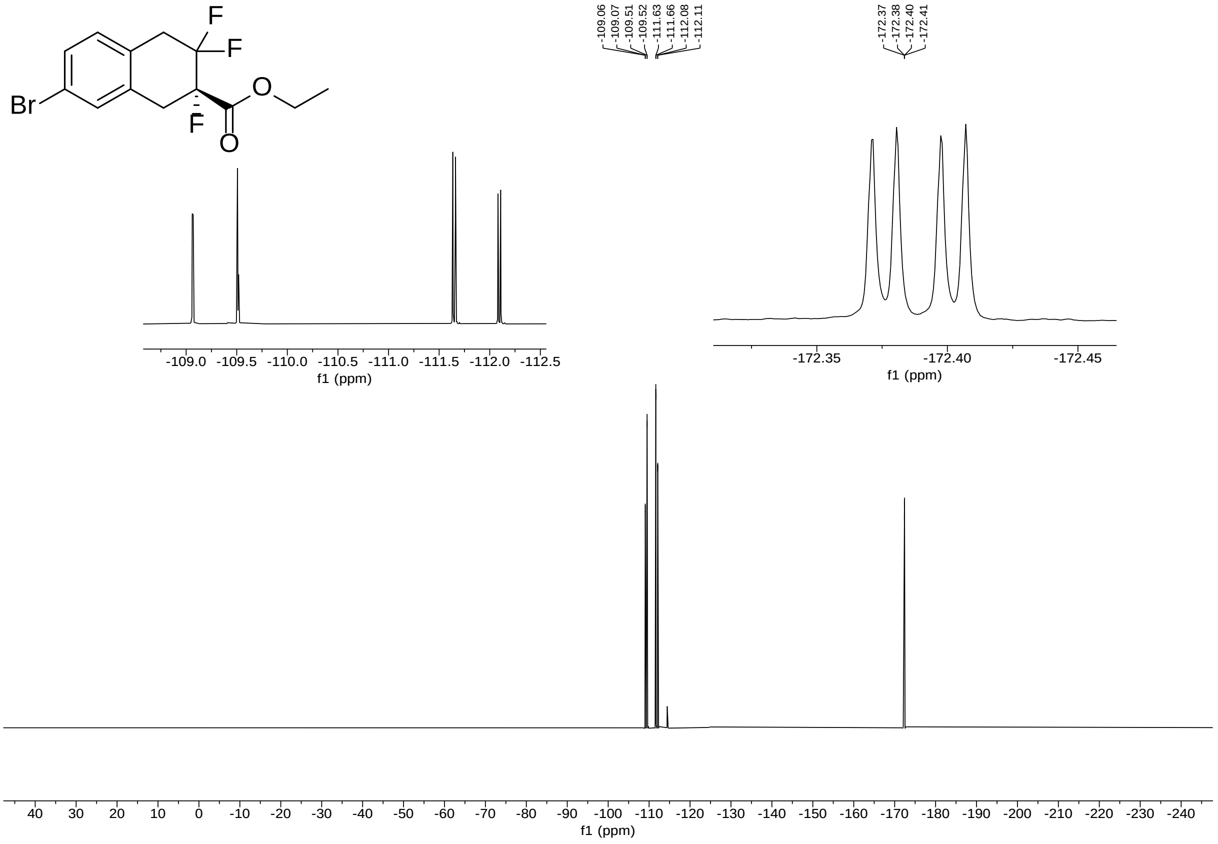

Ethyl (*S*)-7-chloro-2,3,3,-trifluoro-1,2,3,4-tetrahydronaphthalene-2-carboxylate (31)

^{1}H NMR (599 MHz, $CDCl_3$, 299 K)

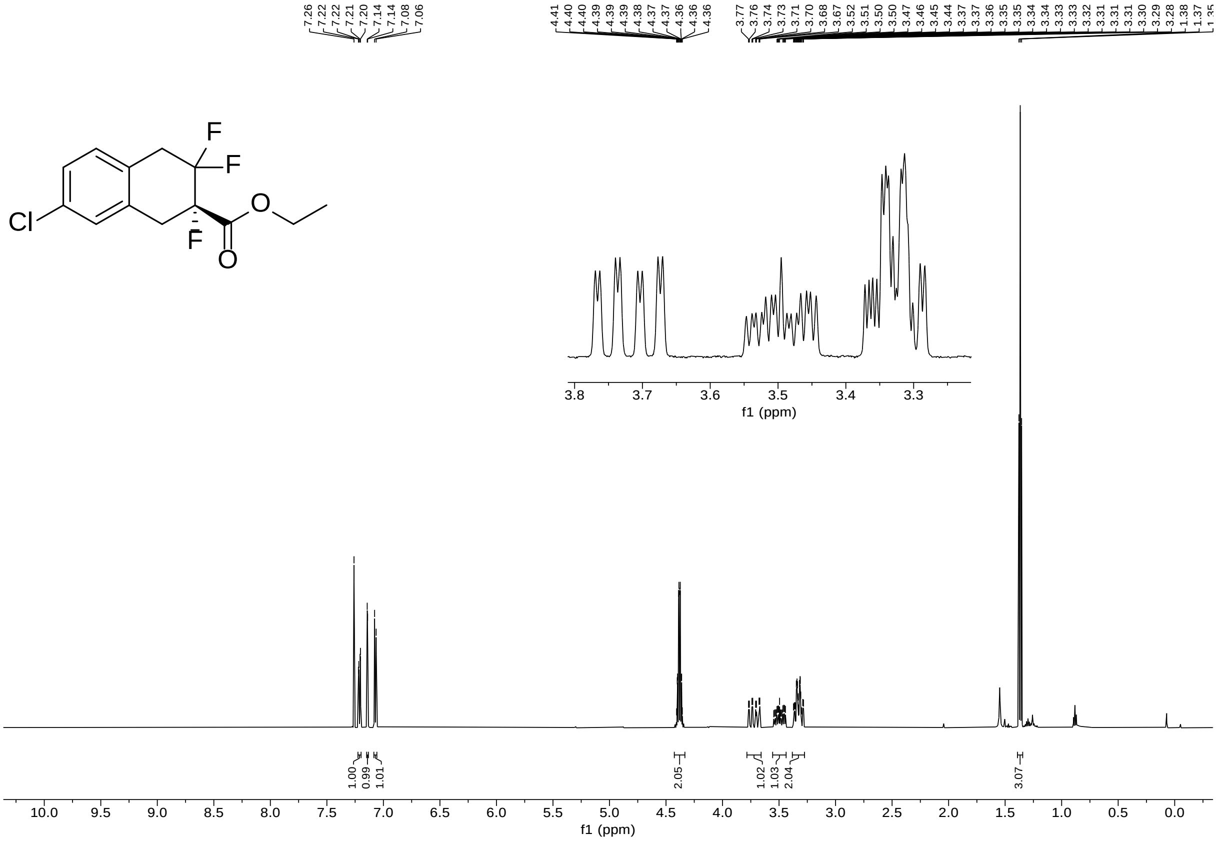

^{13}C NMR (151 MHz, $CDCl_3$, 299 K)

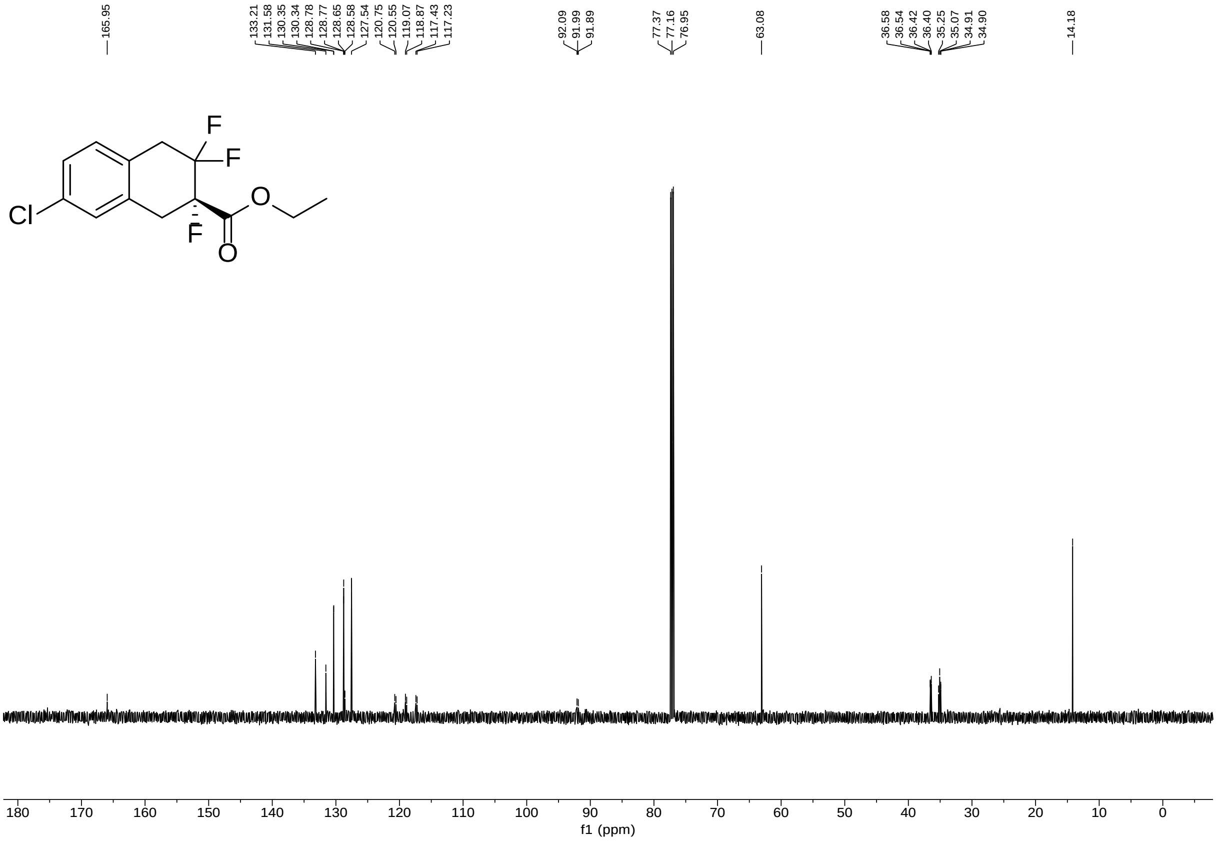

^{19}F NMR (564 MHz, $CDCl_3$, 299 K)

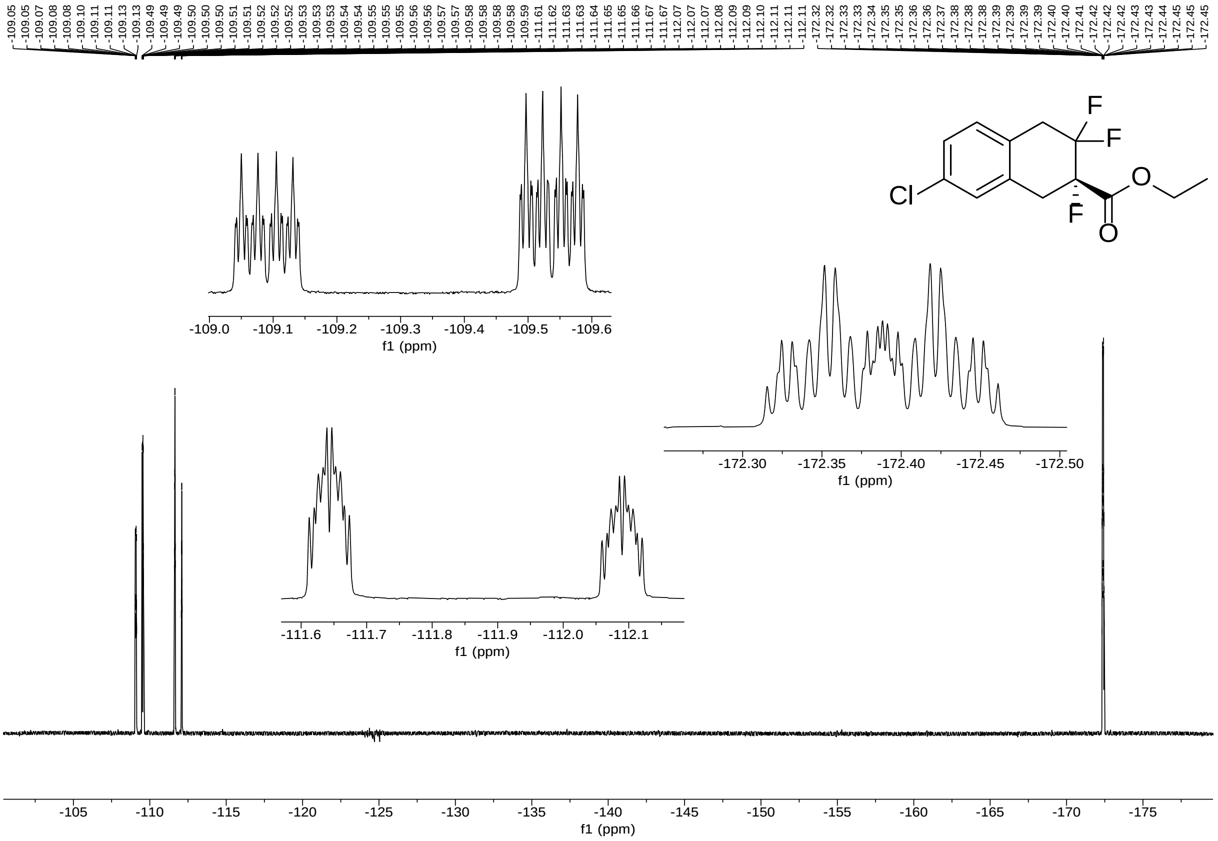

$^{19}F\{^1H\}$ NMR (564 MHz, $CDCl_3$, 299 K)

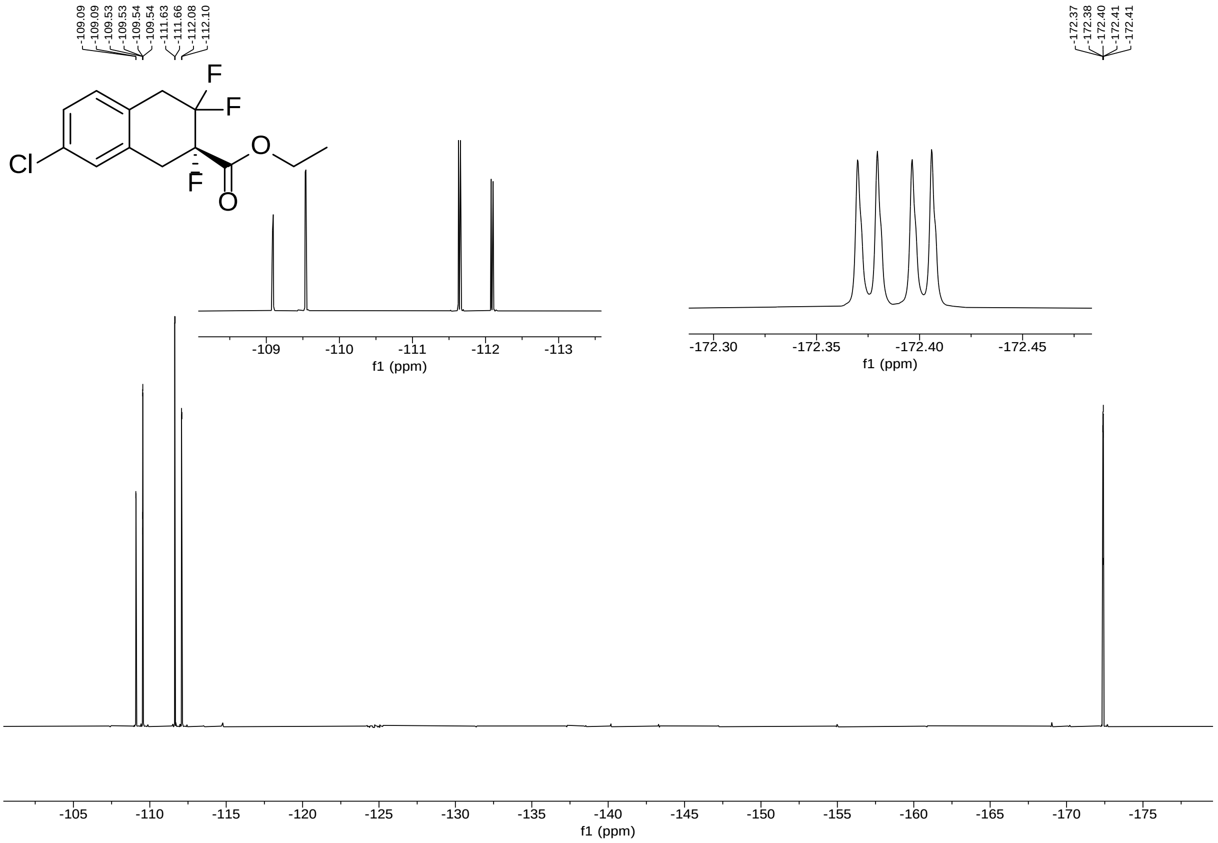

Ethyl (*S*)-2,3,3,-trifluoro-6-(trifluoromethyl)-1,2,3,4-tetrahydronaphthalene-2-carboxylate (32)

^{1}H NMR (599MHz, $CDCl_3$, 299 K)

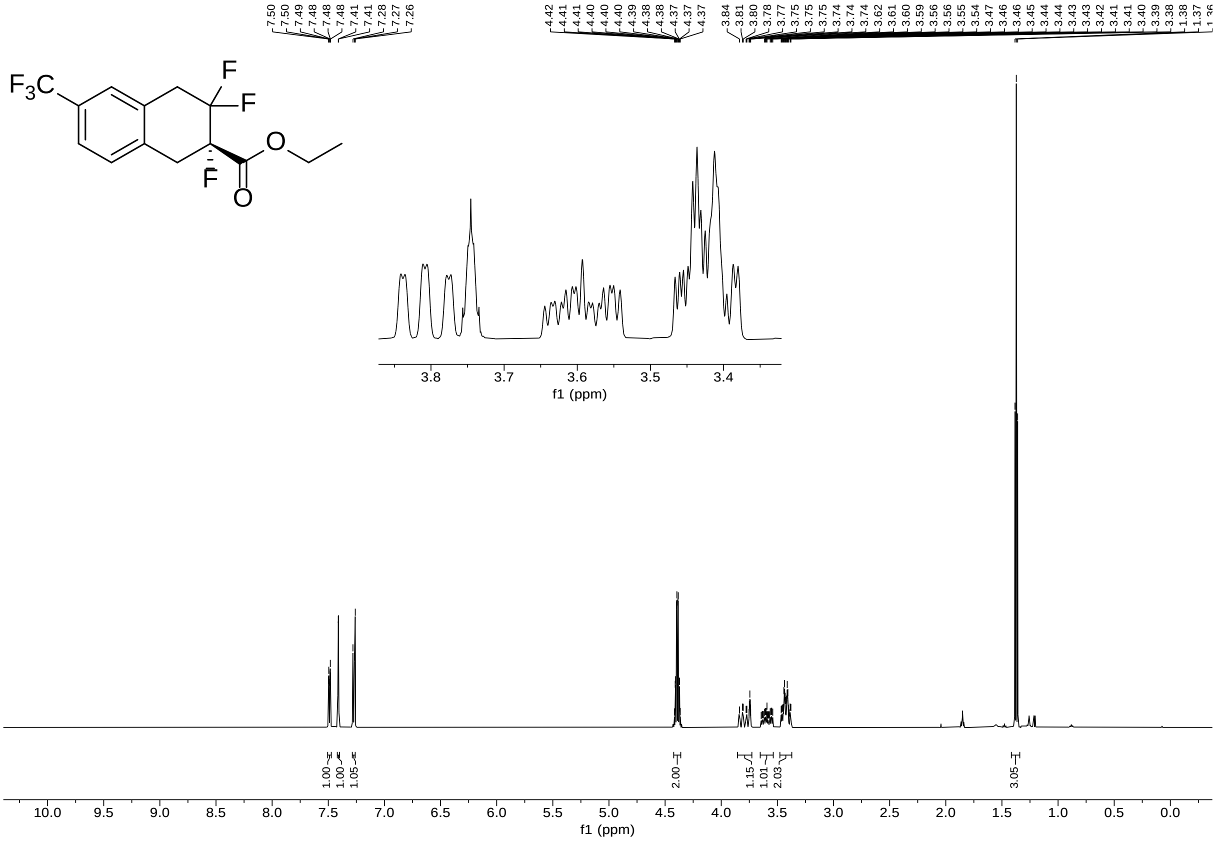

^{13}C NMR (151 MHz, $CDCl_3$, 299 K)

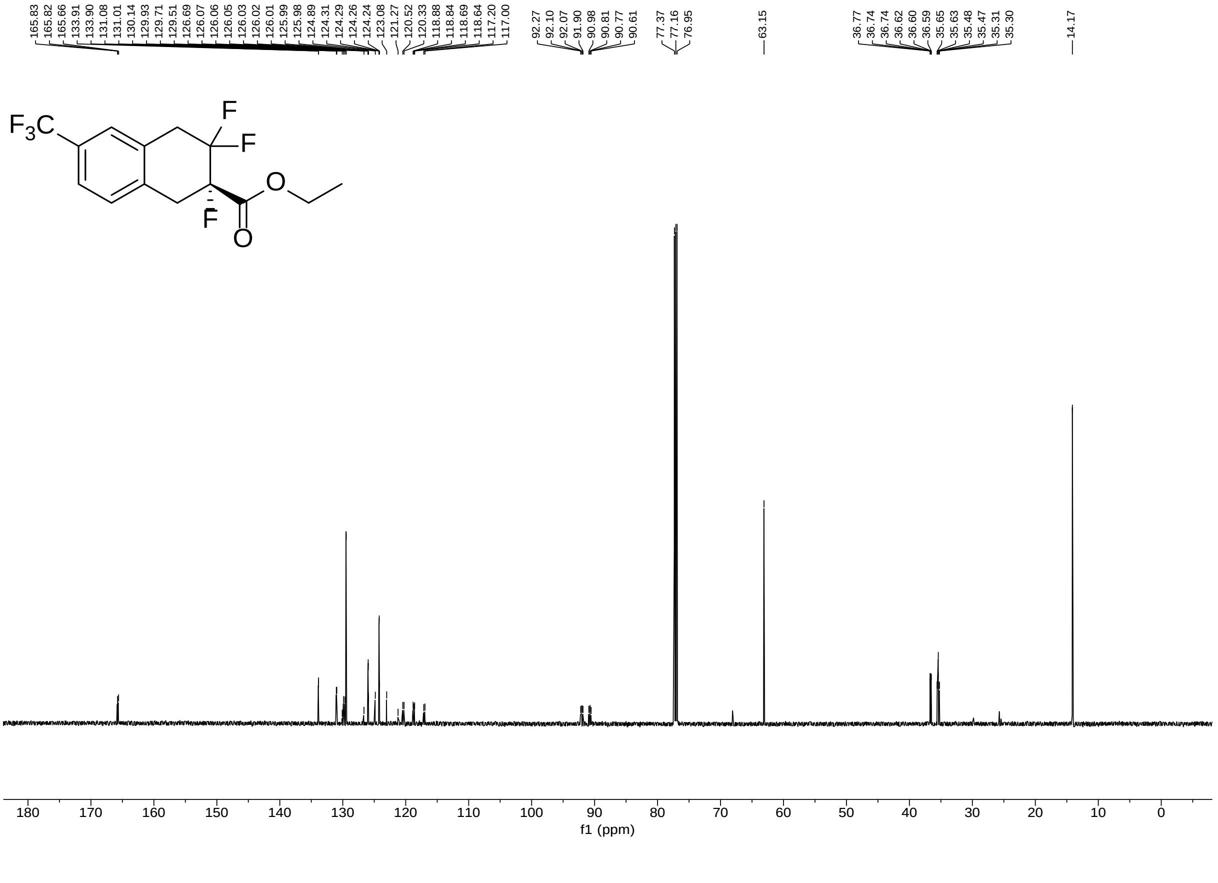

^{19}F NMR (564 MHz, $CDCl_3$, 299 K)

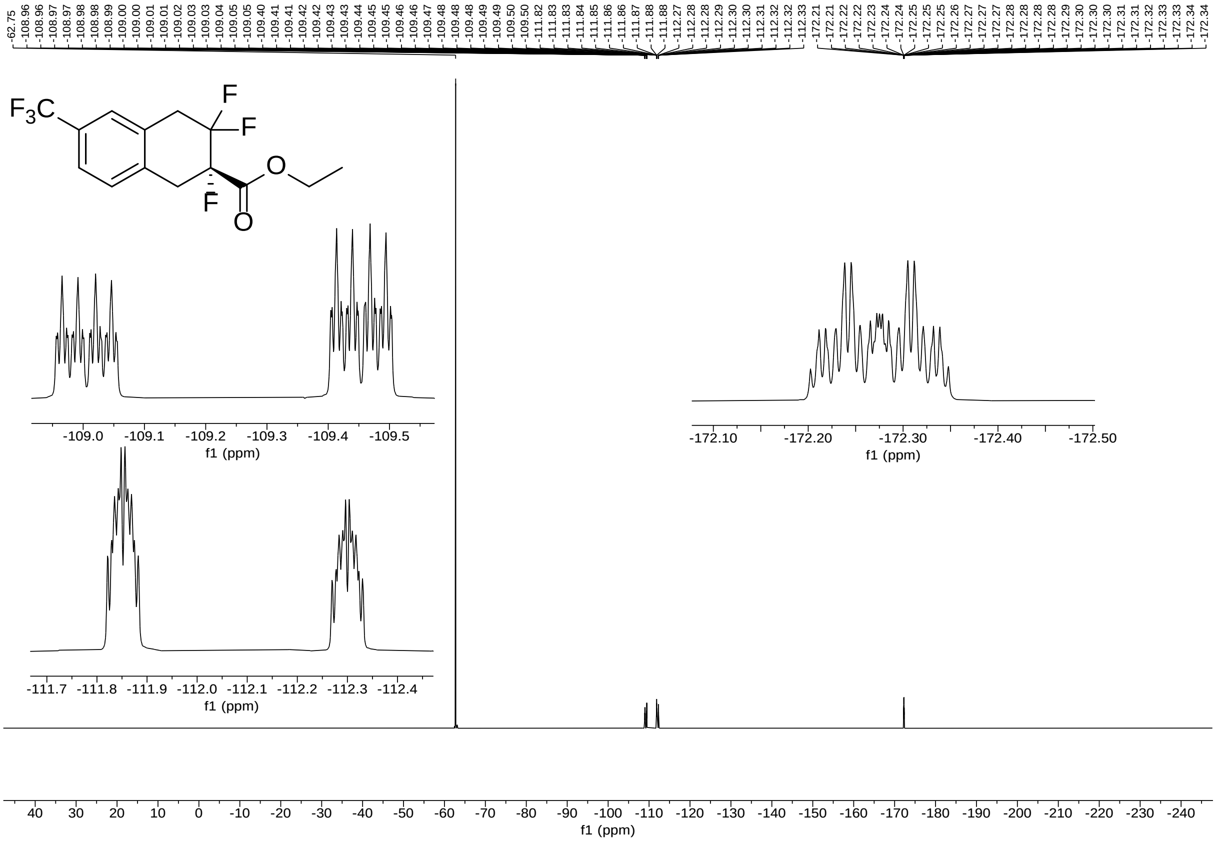

^{19}F{^{1}H} NMR (564 MHz, $CDCl_3$, 299 K)

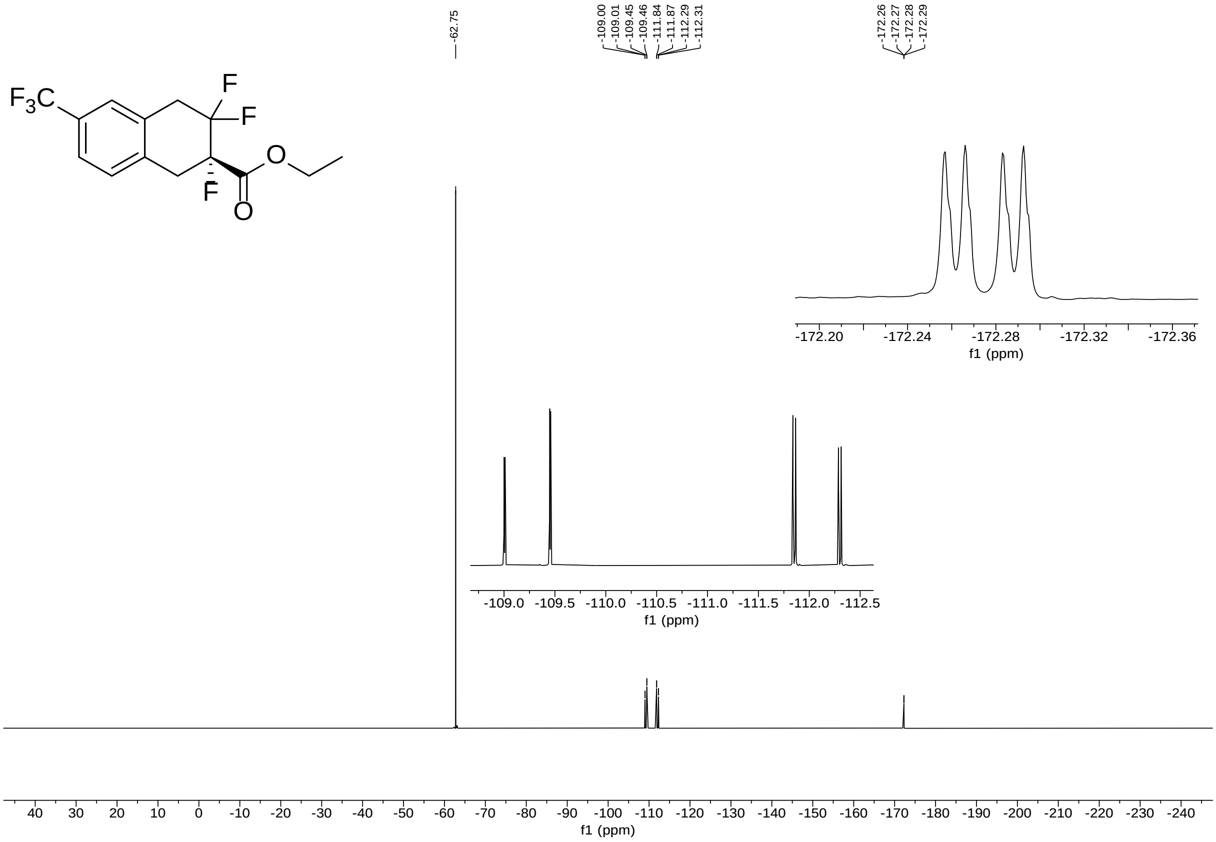

X. References

[1] Y. Zhao, Y. Pan, H. Liu, Y. Yang, Z. Jiang, C. – H. Tan, *Chem. Eur. J.* **2011**, *17*, 3571-3574.

[2] T. W. Liwosz, S. R. Chemler, *Chem. Eur. J.* **2013**, *19*, 12771-12777.

[3] G. Stavber, M. Zupan, M. Jereb, S. Stavber, *Org. Lett.* **2004**, *6*, 4973-4976.

[4] H. – Q. Luo, T. – P. Loh, *Tetrahedron Lett.* **2009**, *50*, 1554-1556.

[5] Z. Jiao, J. J. Beiger, Y. Jin, G. Shaozhong, J. S. Zhoi, J. F. Hartwig, *J. Am. Chem. Soc.* **2016**, *138*, 15980-15986.

[6] T. Asano, S. Kontani, M. Nakajima, *Org. Lett.* **2019**, *21*, 4192-4196.

[7] W. M. Seganish, P. J. DeShong, *J. Org. Chem*. *2004*, *69*, 1137–1143.

[8] S. Ulmschneider, U. Müller-Vieira, M. Mitrenga, R. W. Hartmann, S. Oberwinkler-Marchais, C. D. Klein, M. Bureik, R. Bernhardt, I. Antes, T. Lengauer, *J. Med. Chem.* **2005**, *48*, 1796-1805.

[9] J. Amitabh, T. – Y. Chou, Z. AlJaroudi, B. D. Ellis, T. S. Cameron, *Beilstein J. Org. Chem.* **2014**, *10*, 848–857.

[10] Z. Huang, Q. – Z. Yang, D. Khvostichenko, T. J. Kucharski, J. Chen, R. Boulatov, *J. Am. Chem. Soc.* **2009**, *131*, 1407–1409.

[11] I. Pravst, M. Zupan, S. Stavber, *Synthesis*, **2005**, *18*, 3140-3146.

[12] X. Pigeon, M. Bergeron, F. Barabé, P. Dubé, H. N. Frost, J. – F. Paquin, *Angew. Chem. Int. Ed.* **2010**, *49*, 1123-1127.

[13] Z. S. Zhou, L. Li, X. H. He, *Chinese Chemicals Letters* **2012**, *23*, 1213-1216.

[14] M. A. Kroc, A. Patil, A. Carlos, J. Ballantine, S. Aguilar, D. – L. Mo, H. – Y. Wang, D. S. Mueller, D. J. Wink, L. L. Anderson, *Tetrahedron*, **2017**, *73*, 4125-4137.

[15] J. C. Sarie, C. Thiehoff, J. Neufeld, C. G. Daniliuc, R. Gilmour, *Angew. Chem. Int. Ed.* **2020**, *59*, 15069-15075.

[16] J. C. Sarie, J. Neufeld, C. G. Daniliuc, R. Gilmour, *ACS Catal.* **2019**, *9*, 7232-7237.

[17] R. Pluta, P. E. Krach, L. Cavallo, L. Faliene, M. Rueping, *ACS Catal.* **2018**, *8*, 2582-2588.

[18] E. Bednarova, M. Dracinsky, S. Malatinec, I. Cisarova, F. Lamaty, M. Kotora, M. *Adv. Synth. Catal.* **2018**, *360*, 2869-2878.

[19] A. M. R. Smith, D. Billen, K. K. M. Hii, *Chem. Commun.* **2009**, 3925-3927.

[20] T. Niu, C. Xu, K. - H.Wang, D. Huang, Y. Hu, J. Xu, *Adv. Synth. Catal.* **2020**, *354*, 515-526.

[21] A. Dierks, M. Schmidtmann, J. Christoffers, *Chem. Eur. J.* **2019**, *25*, 5451-5462.

[22] Z. – Y. Zhang, Z.-Y.; Liu, R. – T. Guo, Y. – Q. Zhao, X. Li, X. – C. Wang, *Angew. Chem. Int. Ed.* **2017**, *56*, 4028-4032.
